# Microparticles from tumors exposed to radiation promote immune evasion in part by PD-L1

**DOI:** 10.1038/s41388-019-0971-7

**Published:** 2019-08-29

**Authors:** Michael Timaner, Ruslana Kotsofruk, Ziv Raviv, Ksenia Magidey, Dvir Shechter, Tal Kan, Alexander Nevelsky, Shahar Daniel, Elisabeth G. E. de Vries, Tongwu Zhang, Orit Kaidar-Person, Robert S. Kerbel, Yuval Shaked

**Affiliations:** 1 Cell Biology and Cancer Science, Rappaport Faculty of Medicine, Technion, Haifa, Israel; 2 Oncology Institute, Radiation Oncology Unit, Rambam Health Care Campus, Haifa, Israel; 3 Department of Medical Oncology, University of Groningen, University Medical Center Groningen, Groningen, The Netherlands; 4 Division of Cancer Epidemiology & Genetics, Integrative Tumor Epidemiology Branch, National Cancer Institute, National Institute of Health, Bethesda, MD USA; 5 Sunnybrook Health Sciences Centre, Toronto, ON Canada

**Keywords:** Cancer therapeutic resistance, Immunosurveillance

## Abstract

Radiotherapy induces immune-related responses in cancer patients by various mechanisms. Here, we investigate the immunomodulatory role of tumor-derived microparticles (TMPs)—extracellular vesicles shed from tumor cells—following radiotherapy. We demonstrate that breast carcinoma cells exposed to radiation shed TMPs containing elevated levels of immune-modulating proteins, one of which is programmed death-ligand 1 (PD-L1). These TMPs inhibit cytotoxic T lymphocyte (CTL) activity both in vitro and in vivo, and thus promote tumor growth. Evidently, adoptive transfer of CTLs pre-cultured with TMPs from irradiated breast carcinoma cells increases tumor growth rates in mice recipients in comparison with control mice receiving CTLs pre-cultured with TMPs from untreated tumor cells. In addition, blocking the PD-1-PD-L1 axis, either genetically or pharmacologically, partially alleviates TMP-mediated inhibition of CTL activity, suggesting that the immunomodulatory effects of TMPs in response to radiotherapy is mediated, in part, by PD-L1. Overall, our findings provide mechanistic insights into the tumor immune surveillance state in response to radiotherapy and suggest a therapeutic synergy between radiotherapy and immune checkpoint inhibitors.

## Introduction

Radiotherapy is currently one of the leading treatment options for cancer patients. Ionizing radiation applied locally to the tumor site causes DNA damage and subsequent cell death [1]. A growing number of studies have shown that in addition to the local effects at the irradiated tumor site, radiation activates several systemic biological responses, such as adaptive and innate immune-related activities that affect tumor progression [2–4]. For example, preclinical studies reported that macrophages exposed to radiation promote metastasis [5–7]. Accordingly, the elimination of macrophages from irradiated hosts inhibits tumor growth and metastasis [5]. In addition, Ahn et al. reported that myeloid cells in tumor-bearing mice home to the irradiated tumor site and contribute to tumor angiogenesis and subsequent re-growth [8]. Thus, there are systemic host responses following radiotherapy that contribute to tumor re-growth and subsequent resistance [9].

It has been shown that cells of the adaptive immune system play a role in modulating treatment efficacy in irradiated tumors. For example, following radiation, dying tumor cells stimulate dendritic and cytotoxic T-cell activity directed against viable tumor cells [10, 11]. Thus, tumors or metastatic sites that are outside of the irradiated field may respond positively to the radiation; however, such antitumor effects are rarely reported in the clinic. This phenomenon, termed the “abscopal effect”, is due to extensive inflammation and activation of immune cells as a result of radiation [12, 13]. Contrarily, radiotherapy can lead to myelosuppression that counteracts the benefit of activated immune cells [14, 15]. This cross-talk between immune cells and the irradiated site has provided a rationale for combining radiotherapy with immunotherapy, including immune checkpoint inhibitors [16–18]. Yet, the systemic effects generated in response to radiotherapy and immunotherapy are not fully explored.

The mobilization and trafficking of bone marrow-derived cells (BMDCs) are often affected by extracellular microvesicles (EVs) [19, 20]. EVs serve as a route of communication between cells at distant sites by transferring microRNA, proteins, and lipids, as demonstrated mainly in preclinical studies [20, 21]. EVs are classified according to their size and the mechanism by which they are formed. Exosomes, which are 30–120 nm in size, originate from the internal compartment of a cell, whereas microparticles (MPs), which are 0.1–1.0 µm in size, are generated from the outer cytoplasmic membrane blebbing or shedding, as was previously characterized by transmission electron microscopy [22]. In cancer, tumor-derived microparticles (TMPs) have been associated with therapy resistance. Specifically, they induce inflammation, angiogenesis, and even metastasis (for review see refs. [20, 23]). Moreover, TMPs and exosomes promote the mobilization of BMDCs from the bone marrow compartment and their homing to tumor or metastatic sites [19, 24], an effect that is enhanced in response to chemotherapy [19, 25]. Importantly, recent studies demonstrated that EVs may express programmed death-ligand 1 (PD-L1), thus contributing to immune evasion by PD-L1-dependent T-cell inhibition [26, 27]. However, the role of TMPs in response to radiation and their possible contribution to immune cell activity at the irradiated tumor sites has not been studied.

Here, we show that TMPs from irradiated breast carcinoma cells contain high levels of an array of immunosuppressive proteins in comparison with TMPs derived from untreated cells. These TMPs modulate the immune system, in part via PD-L1, resulting in enhanced tumor growth. Our study suggests that the expression of PD-L1 on TMPs could be useful as a possible biomarker to identify patients who are likely to benefit from the combination of radiotherapy with immune checkpoint therapy.

## Results

### TMPs from irradiated breast carcinoma cells are associated with immune modulation activity

We have previously shown that chemotherapy increases the production of TMPs originating from breast carcinoma cell lines, as well as from breast cancer patients [19]. To determine whether TMP production is similarly affected by radiotherapy, EMT/6, 4T1, PyMT, E0771, and DA3 breast carcinoma cell cultures were exposed to a single fraction of 2 Gy or 6 Gy radiation, in accordance with clinically used doses. Forty-eight hours later, TMPs were collected from the conditioned medium and quantified by flow cytometry, using a method previously described in ref. [19]. With the exception of E0771 cells exposed to 6 Gy radiation, TMP production did not significantly change in response to the tested radiation doses in all other cell lines (Fig. S1A).

Next, to rule out the possibility that apoptotic bodies are generated in response to a single-dose radiation, we assessed cell viability, early, and late apoptosis states in EMT6, 4T1, and PyMT cell lines 48 h after irradiation using Annexin V and 7-AAD staining. Cell viability did not significantly change at this time point, except for 4T1 cells that exhibited a slight increase in necrotic cells at a radiation dose of 6 Gy, but not 2 Gy (Fig. S1B). These results rule out the possibility that quantification of TMPs originating from the control and 2 Gy irradiated cells is affected by the presence of apoptotic bodies in our experimental setting.

We sought to determine whether the physical properties of TMPs are modified in response to radiotherapy. To this end, we assessed whether radiotherapy affects the size of TMPs using NanoSight NS300 analyzer. There were no substantial changes in TMP size between the different groups in any of the breast carcinoma cell lines tested (Fig. S1C). These results indicate that radiotherapy does not affect the physical properties of TMPs, at least in terms of size and therefore TMPs are probably characterized as previously described [28–33].

**Fig. 1:**
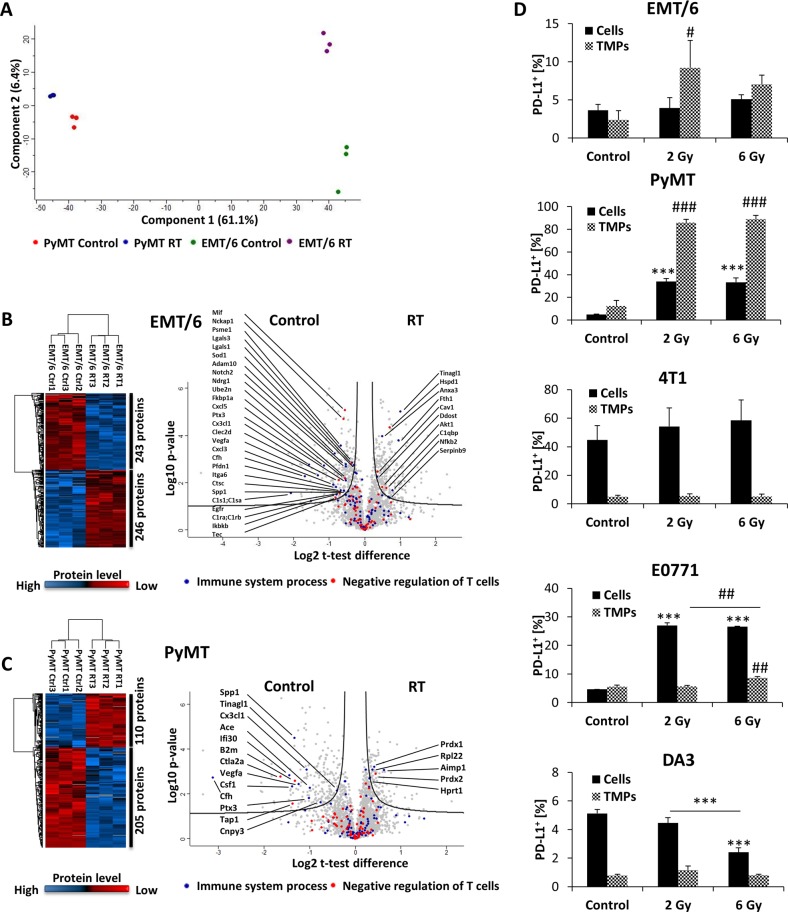
TMPs from cells exposed to radiation contain distinct immunomodulatory proteins. **a** TMPs were collected from untreated (control) or 2 Gy irradiated (RT) EMT/6 or PyMT cells. Protein content was characterized by mass spectrometry analysis. Principal component analysis shows clear separation between the control and irradiated samples. **b**, **c** Heatmap (left) and volcano plot (right) for the comparison between TMPs from the control and irradiated EMT/6 (**b**) or PyMT (**c**) cells. **d** EMT/6, PyMT, 4T1, E0771, and DA3 breast carcinoma cell cultures were irradiated once at the indicated radiation doses. Forty-eight hours later, the percentages of PD-L1-expressing cells and PD-L1-positive TMPs were assessed by flow cytometry (*n* = 3 biological repeats). ***p < 0.001 (for cells) and ^#^*p* < 0.05; ^##^*p* < 0.01; ^###^*p* < 0.001 (for TMPs) as assessed by two-tailed Student’s *t* test (for **b**, **c**) or one-way ANOVA followed by Tukey post hoc test (for **d**)

We next sought to determine whether radiotherapy affects the physiological properties of TMPs. To test this, we characterized the protein content of TMPs originating from the control or irradiated EMT/6 and PyMT cells using high-resolution mass spectrometry-based proteomic analysis, as described in the Materials and methods section. This analysis revealed an increased total protein content in TMPs from cells exposed to radiotherapy in comparison with TMPs from untreated cells in both cell lines tested (Table S1 for the entire proteomic data and Fig. S2). Principal component analysis further highlighted the large variation in protein content of TMPs from control and irradiated cells (Fig. 1a). Furthermore, comparisons between the proteomes of TMPs derived from the control and radiotherapy-exposed EMT/6 and PyMT cells revealed over 480 and 300 significantly altered proteins, respectively, as calculated by Student’s *t* test, FDR 0.05, and S0 = 0.1, as previously described [34] (Fig. 1b, c; Tables S2, S3). Among the immune-related proteins enriched in TMPs from irradiated cells were Hspd1, caveolin 1, AKT1, and complement component protein (C1qbp) in EMT/6 TMPs, and peroxiredoxin 2 in PyMT TMPs, all of which are associated with the suppression of T-cell activation and proliferation (Fig. 1b, c; Tables S4, S5). Furthermore, Fisher’s exact test demonstrated a significant enrichment of various distinct processes such as regulatory, biosynthetic, metabolic, and enzymatic processes in TMPs from radiotherapy-treated cells, in both cell types tested (Tables S6, S7). Altogether, these results suggest that the protein expression pattern in TMPs from radiated breast cancer cells is associated with immune modulation.

Recent studies have demonstrated that extracellular vesicles derived from cancer cells exhibit extensive immunosuppressive activity, an effect mediated by PD-L1 [26, 27]. We therefore investigated the expression level of PD-L1 in our system, comparing between untreated and radiated cells as well as TMPs derived from these cells. Since PD-L1 expression was below the detection threshold in our mass spectrometry analysis, we employed flow-cytometry analysis using anti-PD-L1 antibodies. In PyMT and E0771 cell lines, radiation resulted in an increase in the percentage of PD-L1-positive cells, an effect that was not apparent in DA3, 4T1, and EMT/6 cells. Importantly, there was an increase in the percentage of PD-L1-expressing TMPs derived from EMT/6, PyMT, and E0771 but not 4T1 and DA3 cells exposed to different doses of radiotherapy, when compared to TMPs from untreated cells (Figs. 1d and S3A). Notably, up to 80% of TMPs derived from radiated PyMT cells were positive for PD-L1. Importantly, although the percentage of TMPs expressing PD-L1 was increased, the expression intensity of PD-L1 on these TMPs was not elevated (Fig. S3B), indicating that it is more likely the distribution of PD-L1 on TMPs’s membrane rather than increased production of PD-L1. Consistently, in vivo analysis of TMPs in breast carcinoma tumor-bearing mice exposed to a single dose 2 Gy radiation revealed a significant increase in the percentage of PD-L1-expressing TMPs (Fig. S3C). Collectively, these results demonstrate that radiotherapy affects the percentage of PD-L1-expressing TMPs originating from different breast cancer cells both in vitro and in vivo.

### TMPs derived from irradiated breast carcinoma cells inhibit cytotoxic T-cell activity

**Fig. 2:**
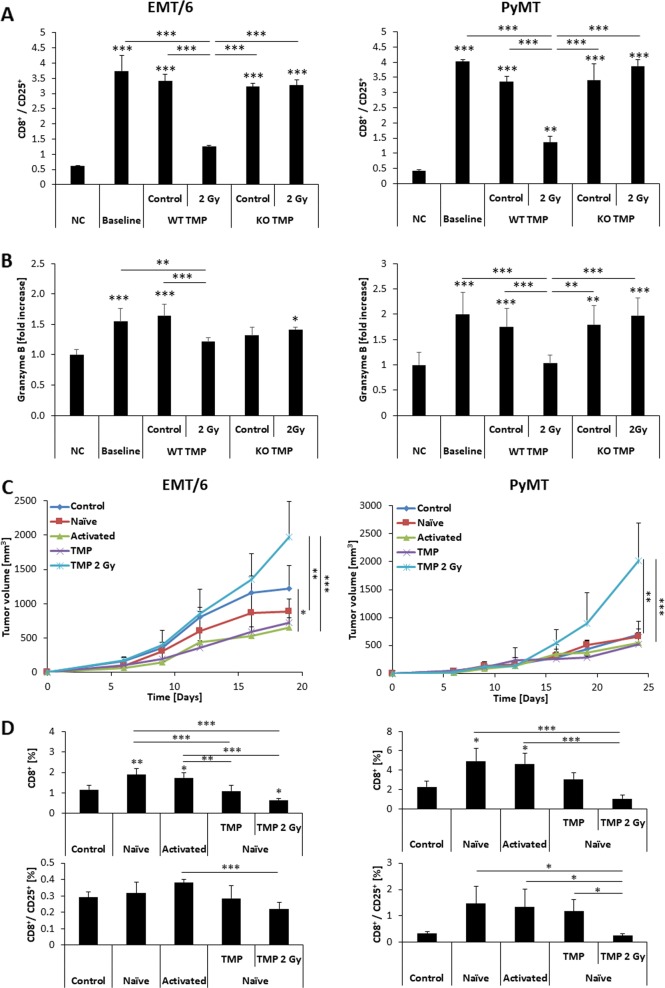
TMPs shed from irradiated EMT/6 and PyMT cells inhibit cytotoxic T-cell activity. **a** Splenocytes isolated from naïve 8–10-week-old BALB/c mice were cultured with CD3^+^/CD28^+^ T-cell activating beads in the absence (baseline) or presence of TMPs derived from the control or irradiated WT EMT/6 or PyMT cells or their PD-L1 KO counterparts as indicated. Negative control (NC) refers to cultures in the absence of activation beads. Twenty-four hours later, the splenocytes were harvested and the percentage of activated cytotoxic T cells (CD8^+^/CD25^+^) was evaluated by flow cytometry. **b** Granzyme B levels in the conditioned medium of the cultures described in (**a**) were assessed by specific ELISA. The results represent three biological repeats. **c**, **d** Eight to 10-week-old SCID and 6 Gy sub-lethally irradiated C57Bl/6 mice (*n* = 5 mice/group) were implanted with EMT/6 or PyMT cells mixed with splenocytes from untreated (naive) mice in a 1:100 ratio. In addition, in some groups, these samples were mixed with TMPs originated from control or 2 Gy irradiated cells, and subsequently injected into mice. A mixture of EMT/6 or PyMT cells with activated splenocytes from tumor-bearing mice were used as a positive control. EMT/6 and PyMT implanted to the mammary fat pad alone were used as negative control. Tumor growth was assessed twice weekly (**c**). At end point, tumors were extracted and the percentage of the total and activated CD8 cells (CD8^+^/CD25^+^) was evaluated by flow cytometry (**d**). *p < 0.05, **p < 0.01, ***p < 0.001, as assessed by one-way ANOVA followed by Tukey post hoc test

PD-L1 binds to PD-1 expressed by several types of immune cells and negatively regulates the activity of cytotoxic T cells [35]. Our proteomic characterization of TMPs originating from irradiated cells suggests that TMPs may play a role in immunomodulation following exposure to radiation. We therefore sought to investigate the immunomodulatory suppression activity of TMPs. We focused on EMT/6 and PyMT cells as they exhibited the highest percentage of TMPs expressing PD-L1 in response to radiotherapy. As a negative control, we chose to work with 4T1 cells, as they produced low levels of PD-L1-positive TMPs, regardless of radiation. PD-L1 knockout was performed in the three cell lines using CRISPR-Cas9 as described in the Materials and methods section. The lack of PD-L1 expression in EMT/6, PyMT, and 4T1 cells was verified by flow cytometry (Fig. S4). To evaluate the effect of PD-L1-positive TMPs on T-cell activation, TMPs were isolated from untreated or irradiated WT and PD-L1 KO EMT/6, PyMT, and 4T1 tumor cell cultures and mixed with splenocytes freshly extracted from spleens of non-tumor-bearing BALB/c or C57Bl/6 mice. The samples were then applied to a T-cell activation kit, and the activation of cytotoxic T cells was monitored by flow cytometry. TMPs isolated from 2 Gy irradiated WT EMT/6 or PyMT cultures inhibited cytotoxic T-cell activation in comparison with TMPs isolated from untreated cultures. However, this inhibitory effect was not apparent with TMPs isolated from PD-L1 KO EMT/6 or PyMT cultures (Fig. 2a). Of note, TMPs isolated from WT or PD-L1 KO 4T1 cells had no effect on T-cell activation, regardless of whether they were isolated from untreated or irradiated cells (Fig. S5A). These results can be further explained by a 6.56-fold increase in a ratio between PD-L1-positive EMT/6-derived TMPs and activated CD8 T cells (when comparing irradiated to untreated groups), whereas in 4T1 there was almost no change in the same ratio (Table S8). Lastly, the levels of granzyme B in the conditioned medium of splenocytes co-cultured with TMPs from WT or PD-L1 KO EMT/6, PyMT, and 4T1 cells were consistent with results obtained in the T-cell activation assay (Figs. 2b and S5B). Overall, these findings demonstrate that PD-L1-positive TMPs, which are generated from irradiated cells, play a role in suppressing cytotoxic T-cell activity in vitro.

Next, to evaluate the immunesuppressive effects of TMPs in vivo, a classic immunological WINN assay was performed, as described in the Materials and methods section. Briefly, splenocytes from naive mice were mixed with EMT/6 or PyMT cells in a 100:1 ratio and co-implanted into the mammary fat pad of naive mice. In some groups, TMPs from control or 2 Gy irradiated cells were mixed with tumor cells and splenocytes, and subsequently co-implanted into the mammary fat pad of naive mice. Mice implanted with EMT/6 or PyMT cells alone or mice co-implanted with activated splenocytes obtained from tumor-bearing mice were used as a negative and positive control, respectively. These experiments were performed in immune-compromised mice (SCID or sub-lethally irradiated mice) in order to eliminate the intrinsic immune responses that take place in immunocompetent mice. Mice co-injected with TMPs originating from 2 Gy irradiated cells exhibited the highest tumor growth rate in both EMT/6 and PyMT models, demonstrating that the TMPs may play a role in immune nodulation (Fig. 2c). Subsequent analysis of cytotoxic T cells in extracted tumors revealed that TMPs from 2 Gy irradiated cells significantly reduced the percentage of CD8-positive cells and their activation state (Fig. 2d). Notably, the percentage of MDSCs and macrophages in both EMT/6 and PyMT tumors remained unchanged, suggesting that they do not play a role in our experimental setting (Fig. S6). Taken together, TMPs originating from irradiated cells contribute to immunosuppression activity, in part by inhibiting cytotoxic CD8+ T cells.

### TMPs derived from irradiated breast carcinoma cells promote tumor growth and inhibit T-cell activity in vivo

**Fig. 3:**
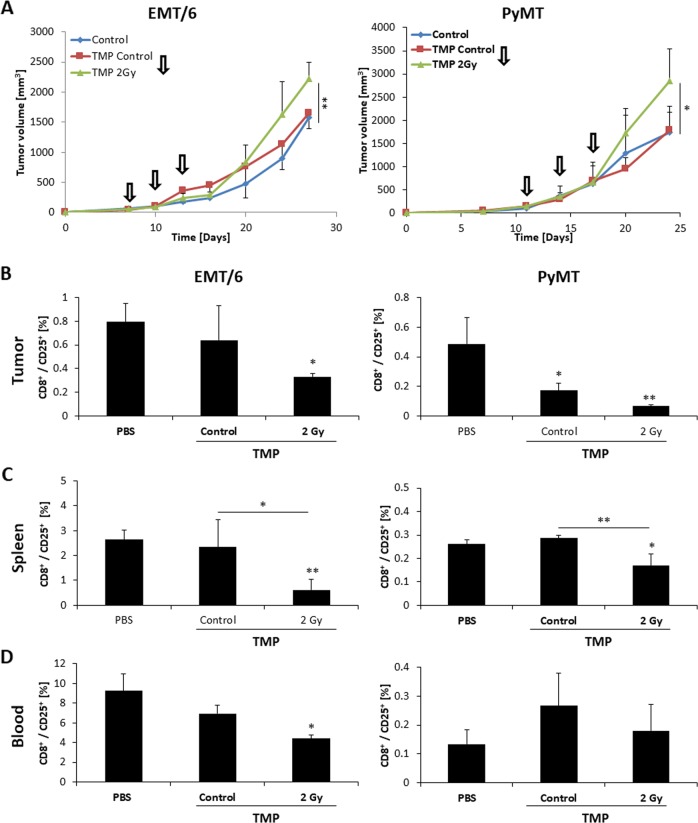
TMPs from irradiated EMT/6 or PyMT cells promote tumor growth and inhibit cytotoxic T-cell activity in vivo. **a** EMT/6 or PyMT tumor cells (5 × 10^5^/mouse) were orthotopically implanted into the mammary fat pad of 8–10-week-old female BALB/c and C57Bl/6 mice, respectively (*n* = 4–5 mice/group). When tumors reached ~100 mm^3^, mice were either intravenously injected with PBS (control) or TMPs derived from control or 2 Gy irradiated EMT/6 or PyMT cells every 3 days (indicated by arrows, 1 × 10^5^ TMPs/mouse). Tumor volume was monitored twice weekly. **b**–**d** At end point, blood was drawn, and tumors and spleens were harvested. The percentage of activated CD8 cells (CD8^+^/CD25^+^) was evaluated in tumors (**b**), spleens (**c**), and peripheral blood (**d**) by flow cytometry. *p < 0.05, **p < 0.01, as assessed by one-way ANOVA followed by Tukey post hoc test

To characterize the in vivo effects of TMPs, we first investigated whether TMPs derived from irradiated cultures affect tumor growth in orthotopic tumor models in mice. To this end, mice were orthotopically implanted with EMT/6, PyMT, or 4T1 cells. When tumors reached a size of ~100 mm^3^, the mice were intravenously injected with the respective TMPs (1 × 10^5^ per injection) derived from untreated or irradiated cultures. Tumor growth was monitored twice weekly until one of the groups reached end point. Tumor growth was enhanced in mice injected with TMPs derived from 2 Gy irradiated EMT/6 and PyMT cultures in comparison with all other groups, while not always significantly. In contrast, TMPs derived from untreated or irradiated 4T1 cultures had no noticeable effect on tumor growth (Figs. 3a and S7A). At end point, tumors were excised, prepared as single-cell suspensions and analyzed by flow cytometry to quantify the level of activated cytotoxic T cells. We found that injecting EMT/6 or PyMT tumor-bearing mice with TMPs derived from 2 Gy irradiated cell cultures reduced the number of activated cytotoxic T cells (CD8^+^/CD25^+^ cells) in tumors compared with control groups, whereas their numbers in 4T1 tumors were unaffected. The level of activated cytotoxic T cells was also reduced in the spleen and to a lesser extent in peripheral blood of EMT/6 and PyMT, but not 4T1 tumor-bearing mice in response to TMP injections (Figs. 3b–d and S7B–D). Taken together, these results suggest a possible link between TMP-mediated immunomodulation and tumor progression in vivo.

### TMPs derived from irradiated tumor cells are distributed in the spleen and liver, but not in lymph nodes and kidney

**Fig. 4:**
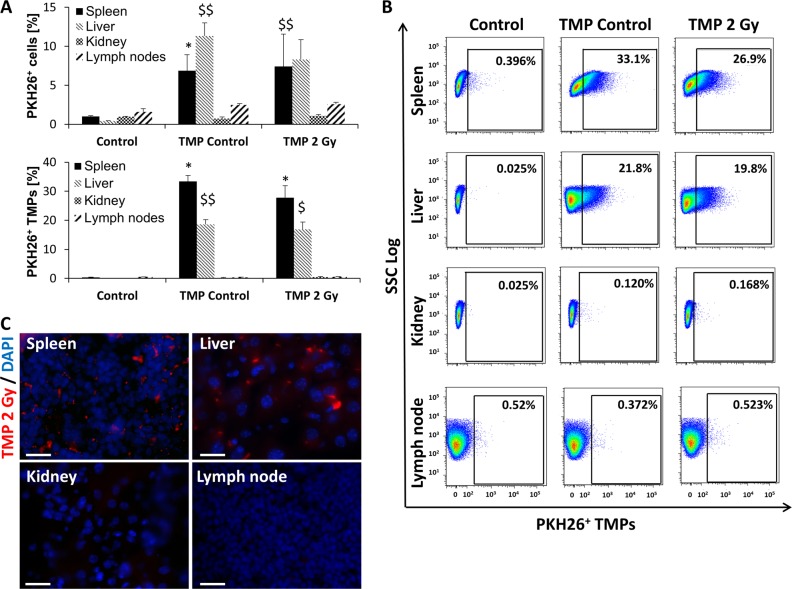
TMPs from irradiated breast carcinoma cells accumulate in spleen and liver. **a** TMPs shed from untreated (control) or 2 Gy irradiated EMT/6 cells were labeled with PKH26, a membrane fluorescent linker. The fluorescently labeled TMPs (1 × 10^5^ TMPs/mouse) were intravenously injected into naive 8–10-week-old BALB/c mice (*n* = 3–4 mice/group). Control mice were injected with PBS. After 24 h, the indicated organs were homogenized, and the percentage of PKH26^+^ cells (cells presumably fused with TMPs) and PKH26-labeled TMPs from the total microparticles (MPs) in the supernatant were evaluated by flow cytometry. **b** Shown are representative flow cytometry dot plots of PKH26-labeled TMPs from the total MPs in the spleen, liver, kidney, and axillary lymph nodes of mice injected with PBS (control) or TMPs from untreated (control) or irradiated cells. **c** Sections of the spleen, liver, kidney, and axillary lymph nodes from mice described in (**a**) after they were injected with TMPs from irradiated EMT/6 cells were stained with DAPI and analyzed by fluorescent microscopy. Representative images are shown. Magnification ×1000 field view; scale bar = 20 µm. *p < 0.05 (for spleen), ^$^*p* < 0.05 and ^$$^*p* < 0.01 (for liver), as assessed by one-way ANOVA followed by Tukey post hoc test

To gain further insight into which organs are involved in the immunomodulatory role of peripheral blood circulating TMPs in vivo, we focused on the distribution pattern of TMPs in various peripheral blood cell-infiltrating organs. To this end, 8–10-week-old BALB/c mice were intravenously injected with PKH26-fluorescently labeled TMPs derived from untreated or 2 Gy irradiated EMT/6 cultures. After 24 h, the mice were sacrificed, and spleens, livers, kidneys, and axillary lymph nodes were processed for immunostaining and flow-cytometry analysis. TMPs accumulated in large numbers in the spleen and liver, but were hardly detected in the kidney and lymph nodes (Fig. 4a–c). Importantly, neither the distribution pattern nor the accumulation of TMPs was affected by exposure to radiation. These results suggest that TMPs shed from cells located at the primary tumor act not only locally at the tumor site but also systemically when distributed through peripheral blood, but such effects are not modified in response to radiotherapy. By colonizing distant organs such as the spleen and liver, the TMPs have the potential to modulate the activity of immune cells at these remote organ sites. Yet, their immunomodulatory effects do not necessary involve lymph nodes.

### TMPs derived from irradiated cells counteract T cell-mediated inhibition of tumor growth

**Fig. 5:**
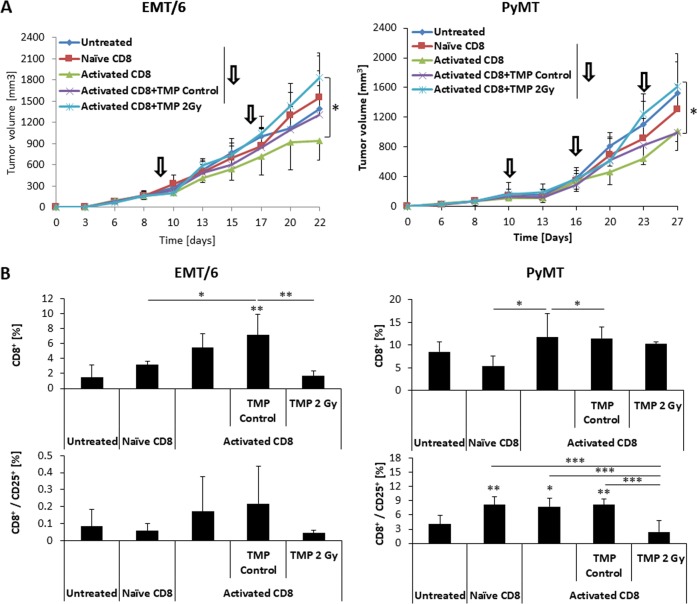
Pre-exposure of cytotoxic T cells to TMPs from irradiated cells counteracts T-cell-mediated inhibition of tumor growth. Spleens of 8–10-week-old naive or EMT/6 or PyMT tumor-bearing mice (BALB/c and C57Bl/6 mice, respectively) were harvested and prepared as single-cell suspensions from which CD8^+^ cells were isolated by negative selection. CD8^+^ cells originating from the tumor-bearing mice were cultured for 24 h either alone (activated CD8) or with TMPs from control EMT/6 or PyMT cells (activated CD8 + TMP control) or 2 Gy irradiated EMT/6 and PyMT cells (activated CD8 + TMP 2 Gy). CD8^+^ cells from each group were intravenously injected into EMT/6 tumor bearing 8–10-week-old female SCID mice or PyMT tumor-bearing 6 Gy sub-lethally irradiated C57Bl/6 mice (10^6^ cells/mouse, *n* = 5 mice/group). Control mice were injected with PBS (untreated). **a** Adoptive transfer of CD8^+^ cells was performed weekly at the indicated time points (arrows) until end point. Tumor growth was assessed every other day. **b** At end point, spleens were removed and prepared as single-cell suspensions for the evaluation of the total CD8^+^ cells and activated T cells (CD8^+^/CD25^+^) by flow cytometry. *p < 0.05, **p < 0.01, ***p < 0.001, as assessed by one-way ANOVA followed by Tukey post hoc test

To further explore the possible link between TMP-mediated immunomodulation and tumor progression, we performed a T-cell adoptive transfer experiment. To this end, CD8^+^ T cells were isolated from spleens of EMT/6 and PyMT tumor-bearing mice. The CD8^+^ cells were then cultured for 24 h either alone or in the presence of TMPs derived from untreated or irradiated EMT/6 or PyMT cultures. Subsequently, the CD8^+^ cells from each group were intravenously injected into SCID mice or sub-lethally irradiated C57Bl/6 mice bearing 200 mm^3^ EMT/6 and PyMT tumors, respectively. Of note, in the PyMT model, all mice including the control group were equally irradiated to avoid any possible additive effect of radiation. The adoptive transfer was repeated weekly, and tumor growth was monitored until one of the groups reached end point. As expected, mice that received CD8^+^ cells from tumor-bearing mice exhibited a slower tumor growth rate in comparison with mice that received T cells from naive mice. Importantly, tumor growth rate was accelerated in mice that received CD8^+^ cells that were pre-cultured with TMPs derived from irradiated EMT/6 or PyMT cultures. In contrast, mice that received CD8^+^ cells pre-cultured with TMPs derived from untreated cultures exhibited tumor growth rate similar to mice that received T cells from tumor-bearing mice (Fig. 5a). At end point, tumors and spleens were harvested and prepared as single-cell suspensions for the evaluation of CD8^+^ T cells by flow cytometry. The percentage of total CD8^+^ cells and the activated fraction of CD8^+^CD25^+^ cells were both reduced in the spleens of PyMT and EMT/6 tumor-bearing mice treated with TMPs from irradiated cells, although such results did not reach statistical significance in the EMT/6 model (Fig. 5b). Similarly, injection of TMPs from irradiated cells resulted in a significant reduction of activated CD8^+^ cells in PyMT tumors, whereas no significant differences were detected between any of the groups in EMT/6 tumors (Fig. S8). It is plausible that the nonsignificant results in T-cell activity found in the EMT/6 tumor model is due to the time gap between the last T-cell injection and the end point of the experiment. Specifically, in the EMT/6 model, analysis was performed 7 days after the last T-cell adoptive transfer, whereas in the PyMT tumor model the tumors were harvested 3 days after T-cell adoptive transfer. Collectively, our results demonstrate that TMPs originating from irradiated tumor cells inhibit cytotoxic T-cell activity, and as such accelerate tumor growth.

### Blocking the PD-1–PD-L1 axis alleviates TMP-mediated inhibition of cytotoxic T-cell activity

**Fig. 6:**
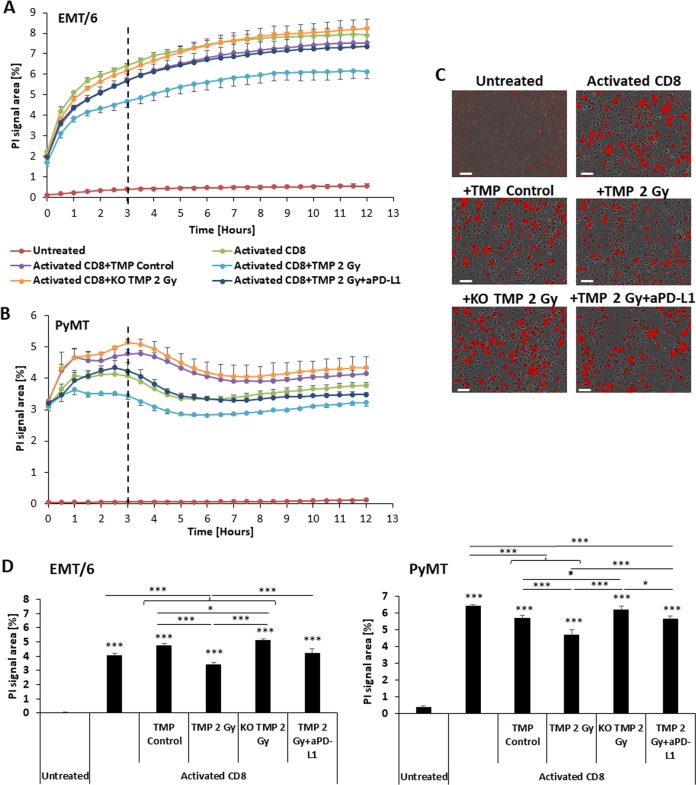
TMP-mediated inhibition of cytotoxic T-cell activity is partially dependent on the PD-1-PD-L1 axis. **a**–**d** CD8^+^ cells were isolated from the spleens of EMT/6 or PyMT tumor-bearing mice (activated CD8). The CD8^+^ cells were cultured with TMPs from untreated (activated CD8+TMP control) or 2 Gy irradiated WT or PD-L1 KO EMT/6 or PyMT cells (activated CD8+TMP 2 Gy and activated CD8+KO TMP 2 Gy groups, respectively). In addition, CD8^+^ cells were cultured with TMPs extracted from 2 Gy irradiated WT EMT/6 or PyMT cells, in the presence of anti-PD-L1 antibody (10 µg/ml) (activated CD8+TMP 2 Gy+αPD-L1). Twenty-four hours later, CD8^+^ cells were washed and then used in a T-cell killing assay performed on EMT/6 (**a**) or PyMT (**b**) cells, as a function of propidium iodide (PI) staining (*n* = 5 biological repeats). EMT/6 or PyMT cells cultured alone served as a negative control (untreated). Representative micrographs at the 12 h time point are shown (**c**). A T-cell killing assay at the 3 h time point is presented by a bar graph (**d**). *p < 0.05, ***p < 0.001 using one-way ANOVA followed by Tukey post hoc test

Immune checkpoint therapy has been shown clinically to dramatically induce cytotoxic activity of CD8^+^ T cells toward malignant cells in certain cancer types. Such therapy is currently standard of care for the treatment of several malignancies [35]. Furthermore, some early-phase clinical studies have revealed that the combination of radiation with immune checkpoint therapy improves therapeutic outcome [16, 17]. Our aforementioned results demonstrate that TMPs originating from irradiated tumor cells can inhibit cytotoxic T-cell activity, in part via PD-L1, whereas TMPs from untreated cells that minimally express PD-L1 have no such effect. We therefore asked whether blocking PD-L1 on irradiated TMPs would alleviate the inhibitory effect of TMPs on T-cell-mediated killing of tumor cells. To this end, CD8^+^ cells isolated from the spleens of EMT/6 or PyMT tumor-bearing mice were cultured with TMPs derived from WT and PD-L1 KO untreated or irradiated EMT/6 and PyMT tumor cells, respectively. In some groups, PD-L1 expressed on TMPs was pharmacologically neutralized with anti-PD-L1 antibody. The T cells were then washed to remove free TMPs and then used in a T-cell killing assay performed on EMT/6 or PyMT cells. As expected, and specifically at the 3 h time point, TMPs from 2 Gy irradiated cells significantly attenuated T-cell killing of cancer cells, compared with activated CD8 cells or T cells pre-cultured with control TMPs. In addition, PD-L1 KO TMPs from irradiated cells did not induce an inhibitory effect in both cell lines. Similarly, pre-culturing TMPs from irradiated cells with anti-PD-L1 antibodies partially restored the T-cell killing activity of CD8^+^ cells (Fig. 6a–d). Furthermore, in an in vivo experiment, the combination of anti-PD-1 therapy with a single dose 2 Gy radiation in mice-bearing EMT/6 tumors resulted in greater tumor growth delay than any of the monotherapy arms (Fig. S9). Overall, these results may provide a possible explanation for the improved therapeutic effect of blocking the PD-L1 immune checkpoint in combination with radiation. They suggest that such enhanced therapeutic activity might be the result of, at least in part, the expression of PD-L1 on TMPs, the effect of which leads to reduced T-cell cytotoxicity and subsequent tumor progression.

## Discussion

Immune checkpoint inhibitor therapy represents a major advancement in cancer treatment, leading to improved response in several cancer types. However, such therapy was found to be beneficial for only a small proportion of patients for reasons that are not fully clear [36]. In this study, we evaluate the immunomodulatory effects of radiation with a specific emphasis on TMPs. In our experimental setting, we used previous published methods to extract TMPs. Their physical characterization has been previously described [19, 28–33]. Next, we demonstrate that TMPs shed from cells exposed to radiotherapy comprise proteins with immunomodulatory and immunosuppressive activities when compared with TMPs from untreated cells. These results prompted us to evaluate the expression of known immunomodulatory proteins, such as PD-L1, on TMPs. Our study demonstrates that in response to radiation the percentage of TMPs expressing PD-L1 originating from breast carcinoma cells may increase; however, this effect is tumor dependent. We show that EMT/6 and PyMT cells shed a higher percentage of PD-L1-positive TMPs in response to radiation in comparison with TMPs of 4T1, E0771, and DA3 cells. The 2 Gy and 6 Gy radiation doses caused distinct effects in several cell lines tested. For example, the 6 Gy dose did not increase the number of PD-L1 expressing TMPs derived from EMT/6 cells. On the contrary, in E0771 cells, 6 Gy radiation increased the percentage of PD-L1-positive TMPs, an effect that was not observed after 2 Gy irradiation. These differential effects might be explained by differences in stress response mechanisms and activation of transcription machinery in the distinct cell types [37, 38]. Importantly, we demonstrate that these effects can modulate cytotoxic T-cell activity in distant organs such as the spleen, and therefore can systemically promote immune escape. Indeed, it has been shown that radiotherapy, in some cases, affects tumor growth in part by modulating the immune system, both within and outside of the irradiation field.

The abscopal effect—a systemic off target therapeutic effect of local radiation—may be explained in part by the presence of tumor cell debris that boosts the immune system, leading to improved therapeutic outcome [4, 10, 39]. In addition, radiotherapy induces the expression of pro-inflammatory cytokines, such as CXCL9, CXCL10, CXCL11, and CXCL16, which enhance the chemotaxis of T cells into tumors and increase their ability to mediate cytotoxicity [40, 41]. Our study demonstrates that radiation inhibits the activity of T cells by various immunomodulatory proteins, such as Hspd1, caveolin 1, AKT1, complement component 1 Q subcomponent-binding protein (C1qbp), and peroxiredoxin 2 (Tables S4, S5). In addition to these proteins, we found that also the percentage of TMPs from cells exposed to radiation express PD-L1. The finding that the distribution of PD-L1 expression is modified in response to radiation provides a rationale to combine radiation with immunomodulatory drugs, which is currently being evaluated in the clinical setting [16–18]. In this regard, in a prospective analysis of melanoma patients treated with CTLA-4 and radiation, a subset of patients benefited from the combinatorial therapy [42]. Furthermore, preclinical studies have indicated that the expression of PD-L1 on the cell surface of tumor cells is altered in response to radiation, thereby potentially contributing to the immunomodulation activity of radiotherapy [43, 44]. Thus, radiation may have both pro-tumorigenic and anti-tumorigenic activities within the tumor site, which may sometimes explain limited response to radiotherapy. In this regard, the basic immunosuppressive effects we report in this study were induced by a single dose 2 Gy radiation, which is considered to be a maximal daily dose in most hyperfractionation protocols. We chose to work with such a dose in our experimental setting in order to avoid tumor cell death and apoptotic bodies which could affect our TMP data. However, in the clinic, cancer patients may receive cumulative doses of 40–80 Gy in hyperfractionation radiotherapy. Therefore, immunosuppressive tumor activity should be further evaluated with repeatable doses of radiation in clinical samples.

EVs affect a variety of cellular and molecular pathways not only in cancer but also in other physiological and pathological conditions [20]. In cancer, tumor-derived EVs transfer oncogenes between tumor cells, mobilize cells that promote tumor growth and metastasis, and contribute to the formation of pre-metastatic niches [21, 22, 45], an effect which can be augmented in response to chemotherapy [25]. Moreover, it has been recently demonstrated that EVs possess an additional role of mediating immune evasion of the tumor through the PD-L1–PD-1 axis. Specifically, EVs originating from stem-like cells of glioblastoma overexpress PD-L1. A correlation with primary tumor volume was found to be associated with T-cell inhibition by such EVs, resulting in the increased aggressiveness of the tumor [26]. In another study, EVs from melanoma cells were found to express PD-L1. The percentage of exosomes expressing PD-L1 in peripheral blood of advanced melanoma patients correlated with disease progression [27]. Here, we specifically show that TMPs can modulate the immune response to radiotherapy. Proteomic analysis revealed that proteins such as Hspd1, caveolin 1, AKT1, complement component 1 Q subcomponent-binding protein (C1qbp), and peroxiredoxin 2 are highly expressed in TMPs from cells exposed to radiation in comparison with TMPs from control cells. While PD-L1 was under the detection level of the mass cytometry analysis in our experimental setting, we chose to focus on PD-L1 due to its known immunomodulatory properties [46]. It must be noted that proteins other than PD-L1 probably play significant roles in immunomodulation and should be further evaluated in additional studies. Indeed, the inhibition of PD-L1 by genetic or pharmacological approaches did not completely inhibit T-cell activity, further indicating that other immunomodulatory proteins expressed by TMPs affect T-cell activity in our experimental setting. Focusing on PD-L1, we demonstrate that the percentage of TMPs originating from certain irradiated breast cancer cells is increased. Furthermore, we demonstrate that these PD-L1-positive TMPs promote tumor growth due to the inhibition of cytotoxic T cells. Our results therefore explain, in part, the synergistic benefit of combining radiotherapy with PD-1–PD-L1 blockade.

In summary, the combination of radiotherapy with immunomodulatory drugs has been shown to improve therapeutic benefit at least in preclinical models and some clinical studies. Yet, the underlying mechanisms described thus far, have been mostly associated with abscopal effects. Here, we demonstrate that TMPs originating from irradiated cells express various immunomodulatory proteins, including PD-L1. These TMPs home to the spleen, and consequently reduce systemic cytotoxic T-cell activity. These effects can explain the pro-tumorigenic activity of TMPs following radiation, also described in other studies [5, 10]. Accordingly, we therefore suggest that evaluating the expression of PD-L1 specifically on circulating TMPs in cancer patients who undergo radiotherapy, may serve as a possible biomarker for successful combination of radiotherapy with immune checkpoint inhibition.

## Materials and methods

### Cell lines

EMT/6, 4T1, and E0771 murine breast carcinoma cell lines and MBA-MB-231 human breast carcinoma cells were purchased from the American Type Culture Collection (ATCC, Manassas, VA, USA) or CH3 Biosystems (Amherst, NY, USA), and were used within 6 months of resuscitation. PyMT murine breast carcinoma cell line was derived from primary tumor-bearing transgenic mice expressing polyoma middle T-antigen (PyMT) under the control of the murine mammary tumor virus (MMTV) promoter [47]. DA3 mammary adenocarcinoma cells were originally derived from the D1-DMBA-3 transplantable mammary tumors [48]. EMT/6, PyMT, DA3, and MDA-MB-231 cells were grown in the Dulbecco's modified Eagle's medium (DMEM, Sigma-Aldrich, Israel), and 4T1 and E0771 cells were grown in the Roswell Park Memorial Institute medium (RPMI, Sigma-Aldrich). All cell lines were supplemented with 10% fetal calf serum (FCS), 1% L-glutamine, 1% sodium pyruvate, and 1% penicillin–streptomycin–neomycin in solution (10 mg/ml, Biological Industries, Israel), and were cultured at 37 °C in 5% CO_2_. The cells were routinely tested as mycoplasma free.

### Generation of PD-L1 KO cells

A 24-base-pair gRNA sequence (CACCGGTCCAGCTCCCGTTCTACA) targeting exon 3 of PD-L1 was cloned into pSpCas9(BB)-2A-GFP(PX458) (Addgene plasmid #48138) according to Ran et al. [49] to obtain pSpCas9-GFP-PD-L1. Consequently, EMT/6, PyMT, and 4T1 cells were electroporated with pSpCas9-GFP-PD-L1. After 48 h, GFP-positive cells were sorted, and single-cells were plated in 96-well plates using FACSAria™ IIIu sorter instrument (BD Bioscience, San Jose, CA, USA). PD-L1 expression of expanded colonies was measured by flow cytometry. Clones showing complete loss of PD-L1 were further confirmed by Sanger sequencing of exon 3 of PD-L1. The original cell cultures were pooled and used as WT control.

### Generation and collection of TMPs

Tumor cells (as indicated in the text) were cultured until they reached 80% confluency, at which point, the medium was replaced with serum-free (SF) medium. Subsequently, cells were exposed to ionizing radiation, using a linear accelerator 6 MeV electron beam using Elekta Precise (Elekta, Sweden) at a dose rate of 40 cGy per minute, for a total dose of 2 Gy or 6 Gy at room temperature as a single fraction (Department of Radiotherapy, Rambam Medical Center, Haifa, Israel). Control cells were not exposed to radiation. To collect TMPs originating from the cells, conditioned medium (CM) was collected 48 h post radiation and centrifuged at 3300×*g* for 20 min at room temperature to remove floating cells. The cell-free supernatants were then centrifuged at 20,000 × *g* for 1 h at 4 °C to precipitate TMPs. The pellet was re-suspended in phosphate buffer solution (PBS), divided into small aliquots, and stored at −80 °C until further use. In some experiments, TMPs were extracted from blood as previously described [19].

### Mass spectrometry and proteomic analysis of TMPs

TMPs were subjected to proteomic analysis at the Smoler Protein research center (Technion). The extracted TMP pellets were re-suspended in 8 M Urea, 100 mM ammonium bicarbonate, and sonicated. The proteins were reduced with 3 mM DTT for 30 min at 60 °C, modified with 9 mM iodoacetamide in 100 mM ammonium bicarbonate in the dark for 30 min at room temperature, and digested in 2 M Urea, 25 mM ammonium bicabonate with modified trypsin (Promega, Madison, WI, USA) at a 1:50 enzyme-to-substrate ratio, overnight at 37 °C. An additional trypsinization was performed for 4 h. The resulting tryptic peptides were desalted using C18 tips (Harvard Apparatus, Holliston, MA, USA) dried and re-suspended in 0.1% formic acid. Samples were analyzed by LC–MS/MS using an Q Exactive plus mass spectrometer (ThermoFisher Scientific, Waltham, MA, USA) fitted with a capillary HPLC (easy nLC 1000, ThermoFisher Scientific). The peptides were loaded onto a C18 homemade capillary column (20 cm, 75 micron ID) packed with Reprosil C18-Aqua (Dr Maisch GmbH, Germany) in solvent A (0.1% formic acid in water). The peptides mixture was resolved with a (5–28%) linear gradient of solvent B (95% acetonitrile with 0.1% formic acid) for 120 min followed by 10 min gradient at 28–95% and 25 min at 95% acetonitrile with 0.1% formic acid in water at flow rates of 0.15 μl/min. Mass spectrometry was performed in a positive mode (m/z 350–1800, resolution 70,000) using repetitively full MS scan followed by collision induces dissociation (HCD, at 35 normalized collision energy) of the 10 most dominant ions (>1 charges) selected from the first MS scan. A dynamic exclusion list was enabled with exclusion duration of 20 s.

The mass spectrometry data were analyzed using the MaxQuant software 1.5.2.8 for peak picking identification and quantitation using the Andromeda search engine, searching against the mouse uniprot database with mass tolerance of 20 ppm and 4.5 ppm after calibration. Oxidation on methionine and protein N-terminus acetylation were accepted as variable modifications, and carbamidomethyl on cysteine was accepted as static modifications. Minimal peptide length was set to seven amino acids, and a maximum of two miscleavages were allowed. Peptide- and protein-level false discovery rates (FDRs) were filtered to 1% using the target-decoy strategy. Protein tables were filtered to eliminate the identifications from the reverse database, and common contaminants and single-peptide identifications. The data were quantified by label-free analysis using the same software, based on extracted ion currents (XICs) of peptides enabling quantitation from each LC/MS run for each peptide identified in any of experiments. The protein groups output was further analyzed using the Perseus 1.5.0.31 software [50]. Label-free quantification (LFQ) intensity values [51] were log_2_ transformed. Each comparison (EMT/6 control vs radiation or PyMT control vs radiation) was processed separately, following data filtration that kept only proteins that were quantified in at least two of the three replicates in at least one of the sample groups. Student’s *t* test was performed with permutation-based FDR correction with a cutoff of 0.05 and S0 correction of 0.1 [34]. Enrichment analysis was carried out using Fisher’s exact test with FDR cutoff of 0.02. PCA was performed following data imputation, by replacing the missing values with values that form a normal distribution with a downshift of 1.6 standard deviations and width of 0.4 of the original data distribution.

### Analysis of TMP number and size

TMPs quantification was performed using flow cytometry as previously described [31]. Briefly, 0.78 µm-sized beads (Bangs Laboratories, Inc., IN, USA) were used to gate the TMPs’ size, and the ratio between TMP number and counting beads (Bangs Laboratories, Inc.) was calculated. In some experiments, size characterization of TMPs was performed using NanoSight NS300 (Malvern, UK) and NanoSight NS300 NTA v2.3 software (Malvern, UK).

### Animal models

All animal studies and animal experimental protocols were approved by the Animal Care and Use Committee of the Technion. EMT/6 or 4T1 cells (5 × 10^5^) were orthotopically implanted into the mammary fat pad of 8–10-week-old female BALB/c mice (Envigo, Israel). PyMT cells (5 × 10^5^) were orthotopically implanted into the mammary fat pad of 8–10-week old female C57Bl/6 mice (Envigo, Israel). MDA-MB-231 cells (2 × 10^6^) were orthotopically implanted into the mammary fat pad of 8–10-week-old female SCID mice (Envigo, Israel). In most experiments, *n* = 5 mice/group were used, unless otherwise indicated. In all experiments, mice were randomly assigned to experimental groups. All irradiation procedures, tumor inoculations, and injections of TMPs were performed blindly. Tumor volume was measured using Vernier calipers and calculated by the formula width × width × length × 0.5. When tumors reached 100 mm^3^, the mice were intravenously injected with 1 × 10^5^ TMPs derived from EMT/6, PyMT, or 4T1 cells (in 100 μl of PBS). Control mice were injected with PBS. In some experiments, when tumors reach 100–200 mm^3^, the mice were exposed to a single dose 2 Gy radiation (locally) and were also treated with anti-PD-1 (100 μg/mouse, twice weekly), or its IgG control. Peripheral blood was collected 48 h following irradiation from retro-orbital sinus bleed, and TMPs were isolated for further analysis as described in ref. [19]. When tumors reached end point (usually 1000–1500 mm^3^) mice were killed, and tumors, spleens, and blood were collected for further analysis as indicated in the article.

For the T-cell adoptive transfer experiment, spleens were harvested from 500 mm^3^ EMT/6 and PyMT tumor-bearing or naive mice, and subsequently prepared as single-cell suspension to extract splenocytes, as previously described [52]. CD8^+^ cells were then isolated from the splenocyte suspensions by negative selection using MojoSort^TM^ mouse CD8 T-cell isolation kit (BioLegend, San Diego, CA, USA) in accordance with the manufacturer’s instruction. CD8^+^ cells from tumor-bearing mice were incubated for 24 h at 37 C° with or without TMPs obtained from 2 Gy irradiated or control EMT/6 or PyMT cells (1 × 10^5^ TMPs/1 × 10^6^ CD8^+^ cells), and afterwards were washed to remove free TMPs. Next, the CD8^+^ cells were intravenously injected into orthotopically implanted 10-week-old SCID mice-bearing 100–200 mm^3^ EMT/6 tumors or 10-week-old C57Bl/6 mice sub-lethally irradiated (6 Gy total body radiation, at a dose rate of 125 cGy per min) bearing 100–200 mm^3^ PyMT tumors. This adoptive T-cell transfer was repeated once a week. At end point, mice were killed, and tumors and spleens were harvested for further analysis as described below.

The WINN assay was performed as previously described [53, 54]. Briefly, spleens were collected from naive mice or 500 mm^3^ EMT/6 or PyMT tumor-bearing mice. Subsequently, they were prepared as single-cell suspension to obtain splenocytes. The splenocytes from naive mice were mixed with EMT/6 or PyMT cells in a ratio of 100:1 (50 × 10^6^ splenocytes/0.5 × 10^6^ EMT/6 or PyMT cells) as well as with TMPs (1 × 10^5^) extracted from naive or 2 Gy irradiated EMT/6 or PyMT cells. The mixture of cells and TMPs was subcutaneously injected to the flanks of 8–10-week old SCID mice (for EMT/6 tumors) and sub-lethally irradiated C57Bl/6 (for PyMT tumors). The mixture of activated splenocytes from tumor-bearing mice and tumor cells was used as a positive control. Tumor growth was monitored regularly. At end point, tumors were resected, processed for single-cell suspension, and analyzed for T-cell activity.

### Evaluation of cytotoxic T-cell activity and cancer cell killing

Cytotoxic T-cell activity was evaluated using a mouse T-cell activation kit (Miltenyi Biotec, Germany) in accordance with the manufacturer’s instructions. Briefly, splenocytes (10^6^) from naive BALB/c or C57Bl/6 mice were cultured for 24 h at 37 °C in the presence or absence of anti-mouse CD3ε, and CD28 biotinylated beads (10^6^ beads/sample) in the presence or absence of TMPs originating from EMT/6 or PyMT cells. The beads mimic antigen-presenting cells and activate resting T cells. Subsequently, cells were centrifuged at 470 × *g* for 5 min at room temperature. Cell pellets were re-suspended in PBS, and the levels of total CD8^+^ T cells and activated T cells (CD8^+^/CD25^+^) were quantified by flow cytometry as described below. In parallel, granzyme B secretion was evaluated by quantifying its level in the supernatants of the spleen and tumor cells using specific ELISA (R&D Systems, Minneapolis, MN, USA), in accordance with the manufacturer’s instructions.

For the T-cell killing assay, CD8^+^ cells were isolated by negative selection from splenocyte suspensions derived from the spleens of EMT/6 or PyMT tumor-bearing mice, using MojoSort^TM^ mouse CD8 T cell isolation kit (BioLegend, San Diego, CA, USA), in accordance with the manufacturer's instructions. The CD8^+^ cells were then cultured for 24 h at 37 °C in the presence or absence of TMPs from untreated or 2 Gy irradiated EMT/6 or PyMT cells (WT or PD-L1 KO cells). For pharmacological PD-L1 inhibition, 10 µg/ml anti-mouse PD-L1 antibody (BioXCell, West Lebanon, NH; cat. BP0101) was added. Subsequently, the T cells were washed with PBS to remove free TMPs and unbound antibodies and then cultured with EMT/6 or PyMT cells for 24 h in a ratio of 10:1 (25,000–2500 cells). Propidium iodide (PI, 500 nM) was added to cultures in order to identify dead cells. T-cell killing effect was monitored using Incucyte Zoom HD/2CLR system (Essen BioScience, Ann Arbor, MI). All experiments were performed in triplicates using at least three biological repeats.

### Flow-cytometry acquisition and analysis

Single-cell suspensions prepared from tumor, spleen, or peripheral blood cells as previously described [52], were immunostained with antibodies purchased from BioLegend (BLG, San Diego, CA) or BD Biosciences (BD, Franklin Lakes, NJ) in accordance with the manufacturers’ instruction. The following marker combinations were used to define total T cells (CD8^+^, cat. BLG-100714), activated cytotoxic T lymphocytes (CD8^+^, cat. BLG-100714/CD25^+^, cat. BD-102007), myeloid-derived suppressor cells (MDSC, CD11b^+^, cat. BLG-101230/Gr-1^+^, cat. BD-108408), and macrophages (F4/80^+^, cat. BLG-123116). TMPs derived from the control, or radiotherapy-treated murine breast cancer cells were immunostained and analyzed for PD-L1 expression. In peripheral blood samples obtained from mice, TMPs derived from MDA-MB-231 tumors were identified by immunostaining for human leukocyte antigen (HLA, cat. BD-311406) and analyzed for human PD-L1 expression (cat. BD-329706). Similarly, breast carcinoma cells and their derived TMPs were analyzed for PD-L1 expression (cat. BD-124312). Annexin V and 7-aminoactinomycin D (7-AAD) were used to distinguish between live, early apoptotic, late apoptotic, and dead cells, as previously described [55]. In some experiments, TMPs were labeled with PKH26 fluorescent cell linker (Sigma-Aldrich, St. Louis, MO, USA) in accordance with the manufacturer's instructions. These PKH26-tagged TMPs or cells with which they fused were identified as a positive event. At least 200,000 events were acquired using an LSRFortessa flow analyzer system (BD Bioscience, San Jose, CA, USA) or CyAN ADP flow cytometer (Beckman Coulter, CA, USA) followed by analysis using the FlowJo 7.6.1 software (FlowJo, LLC, Ashland, OR, USA).

### Immunostaining

Spleens, livers, kidneys and lymph nodes were embedded in OCT (Tissue-Tek, Sakura Finetek USA Inc., Randor, PA, USA) and stored at −80 °C until further processed. Next, tissues were cryosectioned (10 μm) using Leica CM1950 Clinical Cryostat (Leica Biosystems, Germany). Sections were fixed with 4% paraformaldehyde at 4 °C for 30 min, stained with 4'-6-Diamidino-2-phenylindole (DAPI) (Electron Microscopy Sciences, Hatfield, PA, USA) to stain nuclei and analyzed using an inverted fluorescent microscope Leica CTR 6000 microscope system (Leica Microsystems, Germany) per ×100 objective field.

### Statistical analysis

To ensure the adequate power of the results all experiments were performed at least in two technical triplicates and three biological repeats. In some experiments, mice that exhibited pathological conditions which are not related to the experiment were excluded from the analyses. All experiments were performed in a randomized manner. The analysis of the results was performed blindly. The data are presented as mean ± standard deviation (SD). Statistically significant differences were assessed by one-way ANOVA, followed by Tukey post hoc test (when comparing between more than two groups) using GraphPad Prism 4 software (La Jolla, CA, USA). When applicable, estimate of variance was performed and statistical significance comparing only two sets of data were determined by two-tailed Student’s *t* test. Significance was set at values of *p* < 0.05, and designated as follows: *p < 0.05; **p < 0.01; ***p < 0.001.

## Supplementary information

Supplemental Figures S1-S9

Supplemental Tables S1-S8
