# Supplementary material for: Microparticles from tumors exposed to radiation promote immune evasion in part by PD-L1
Source: Oncogene. 2019 Aug 29;39(1):187–203. doi: 10.1038/s41388-019-0971-7 (PMC6937213; doi:10.1038/s41388-019-0971-7)
Supplement: Supplementary file 1 — Supplemental Figures S1-S9 [file 41388_2019_971_MOESM1_ESM.pdf]

Supplemental Figures

Figure S1: Breast cancer cell viability as well as TMP number and size in response to radiation.

A-C. EMT/6, PyMT, 4T1, E0771, and DA3 breast carcinoma cell cultures were irradiated once at the indicated radiation doses. Forty-eight hours later, the number of TMPs present in the conditioned medium was evaluated by flow cytometry (**A**, n=5 biological repeats). Cell viability and apoptosis stages of EMT/6, PyMT, and 4T1 were evaluated by 7AAD and Annexin V using flow cytometry (**B**, n=3 biological repeats). The TMP fractions were analyzed for their size by Nanocyte NS300 system. Representative graphs of TMP size for each radiation dose are shown (**C**, n=3 biological repeats). *p<0.05, #p<0.05 as one-way ANOVA followed by Tukey post-hoc test.

Figure S2: Total number of proteins detected in TMPs from control or radiotherapy-exposed cells.

EMT/6 or PyMT cells were exposed to 2 Gy radiation (RT) or left untreated (control). TMPs were collected 48 h later and their protein content was analyzed by mass spectrometry. The total number of identified proteins was assessed using Perseus software. **p<0.001, as assessed by Student's t-test.

Figure S3: Expression of PD-L1 on breast carcinoma TMPs.

A-B. EMT/6, PyMT, 4T1, E0771, and DA3 breast carcinoma cell cultures were irradiated once at the indicated radiation doses. Forty-eight hours later, the percentage of TMPs expressing PD-L1 was assessed by flow cytometry. Representative dot-plots are provided for TMPs from EMT/6, PyMT and 4T1 cells (**A**). The intensity of PD-L1 expression in TMPs was assessed by flow cytometry using median fluorescence intensity (MFI) (**B**, n=3 biological repeats). **C**. MDA-MB-231 cells were implanted into the mammary fat pad of 8-10 week old female SCID mice (n=3/group). When tumors reached 100-200mm^3, the mice were exposed to a single dose of 2 Gy radiation (2 Gy) or left untreated (Control). Blood was drawn 48 hours later and TMPs were extracted. TMPs were immunostained for human HLA and PD-L1. The percentage of PD-L1 positive TMPs from total HLA$^+$ TMPs was evaluated by flow cytometry. Naïve non-tumor bearing mice served as a negative control (Naïve). ND – non-detectable. **p<0.01, as assessed by Student's t-test (between control and 2Gy radiation).

Figure S4: Validation of PD-L1 expression in PD-L1 KO breast cancer cells

EMT/6, PyMT, and 4T1 cells were electroporated with pSpCas9-GFP-PD-L1 to generate PD-L1 knock-out (KO) cells. After 48 hours, cells were expanded and sorted for GFP-positive clones. PD-L1 expression of expanded colonies was measured by flow cytometry. The original cell cultures were used as WT control.

Figure S5: CD8+ cell activity in the presence of TMPs originating from irradiated 4T1 breast carcinoma cells.

A. Splenocytes isolated from naïve 8-10 week old BALB/c mice were cultured with $CD3^+/CD28^+$ T cell activating beads in the absence (baseline) or presence of TMPs derived from control or irradiated WT or PD-L1 KO 4T1 cells as indicated in the Figure. Negative control (NC) refers to cultures in the absence of activation beads. Twenty-four hours later, the splenocytes were harvested and the percentage of activated cytotoxic T cells ($CD8^+/CD25^+$) was evaluated by flow cytometry. **B.** Granzyme B levels in conditioned medium of the cultures described in (A) were assessed by specific ELISA. The results represent 3 biological repeats. *$p<0.05$, **$p<0.01$, ***$p<0.001$, as assessed by one-way ANOVA followed by Tukey post-hoc test.

Figure S6: TMPs from irradiated cells do not affect innate immune cells.

Eight to ten-week-old SCID and 6 Gy sub-lethally irradiated C57Bl/6 mice (n=5/group) were injected with EMT/6 and PyMT cells, respectively (Control). In other groups, mice were co-injected with tumor cells mixed with splenocytes from untreated (Naïve) or tumor bearing mice (Activated) in a 100:1 ratio. In addition, a mixture of tumor cells with naive splenocytes was co-injected with TMPs originated from control or 2 Gy irradiated cells (TMPs' groups). At endpoint, the percentages of MDSCs (CD11b+/Gr-1+, **A**) and macrophages (F4/80+, **B**) were analyzed in tumors.

Figure S7: TMPs from 4T1 tumors do not affect immune cell activity.

A. 4T1 tumor cells ($5x10^5$/mouse) were orthotopically implanted into the mammary fat pad of 8-10 week old female BALB/c mice (n=4-5 mice/group). When tumors reached 100 mm^3, mice were either intravenously injected with PBS (control) or TMPs derived from control or 2 Gy irradiated EMT/6 or PyMT cells every three days (indicated by arrows, $1x10^5$ TMPs/mouse). Tumor volume was assessed twice weekly. **B–D.** At end point, blood was drawn and tumors and spleens were harvested. The percentage of activated CD8 cells (CD8+/CD25+) was evaluated in tumors (**B**), spleens (**C**) and peripheral blood (**D**) by flow cytometry. Statistical significance was assessed by one way ANOVA followed by Tukey post-hoc test.

Figure S8: T cell activity in tumors from mice that underwent adoptive transfer with CD8+ cells cultured with TMPs.

EMT/6 and PyMT tumors from the adoptive transfer experiment described in Figure 5, were harvested and prepared as single cell suspensions. The percentages of total $CD8^+$ cells and activated T cells ($CD8^+/CD25^+$) were assessed using flow cytometry. $^{**}p<0.01$, $^{***}p<0.001$, as assessed by one-way ANOVA followed by Tukey post-hoc test.

Figure S9: Tumor growth in mice receiving radiation and anti-PD-1 therapy.

EMT/6 cells were implanted to the mammary fat pad of 8-10 week old female BALB/c mice. When tumors reached 200 mm^3, treatment with anti-PD-1, a single dose of 2 Gy radiation or the combination of the two, was initiated. Anti-PD-1 was administered twice weekly at a dose of 100μg/mouse. Control mice were injected with IgG control. Tumor growth was assessed twice weekly.

Figure S1

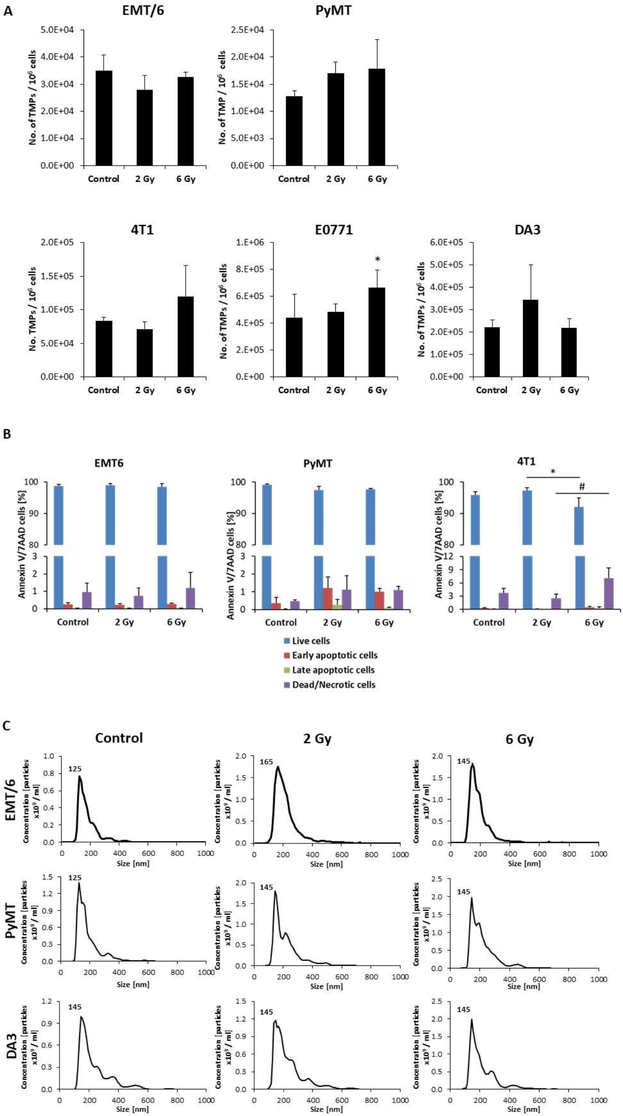
A
EMT/6
PyMT
4T1
E0771
DA3
No. of TMPs / 10^6 cells
No. of TMP / 10^6 cells
No. TMPs / 10^6 cells
Control
2 Gy
6 Gy
5.0E+04
4.0E+04
3.0E+04
2.0E+04
1.0E+04
0.0E+00
2.5E+04
2.0E+04
1.5E+04
1.0E+04
5.0E+03
2.0E+05
1.5E+05
1.0E+05
5.0E+04
1.E+06
8.E+05
6.E+05
4.E+05
2.E+05
0.E+00
6.0E+05
5.0E+05
4.0E+05
3.0E+05
2.0E+05
1.0E+05
*
B
EMT6
PyMT
4T1
Annexin V/7AAD cells [%]
*
#
Live cells
Early apoptotic cells
Late apoptotic cells
Dead/Necrotic cells
C
Control
2 Gy
6 Gy
EMT/6
PyMT
DA3
Concentration [particles x10^9 / ml]
Size [nm]
125
165
145
125
145
145
145
145
145

Figure S2

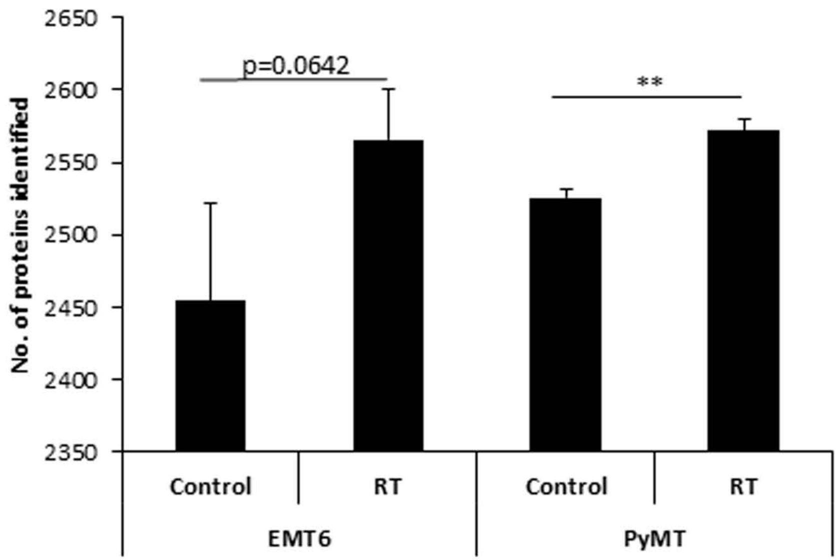
2650
2600
2550
2500
2450
2400
2350
No. of proteins identified
p=0.0642
**
Control
RT
Control
RT
EMT6
PyMT

Figure S3

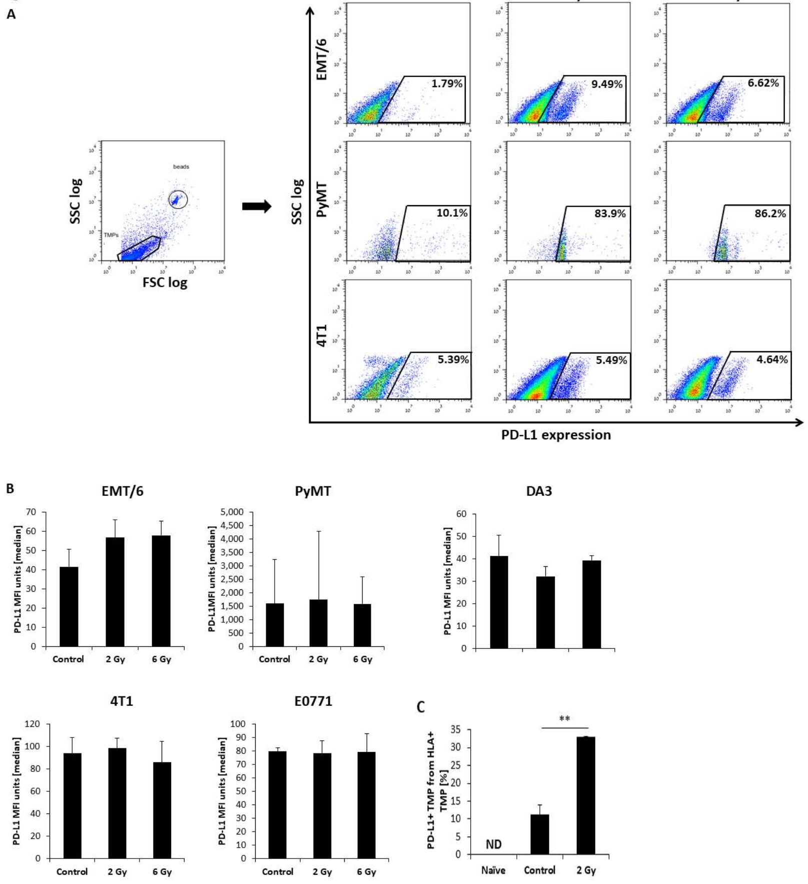
A
Control
2 Gy
6 Gy
EMT/6
PyMT
4T1
SSC log
FSC log
beads
TMPs
1.79%
9.49%
6.62%
10.1%
83.9%
86.2%
5.39%
5.49%
4.64%
PD-L1 expression
B
EMT/6
PyMT
DA3
4T1
E0771
PD-L1 MFI units [median]
PD-L1MFI units [median]
Control
2 Gy
6 Gy
C
**
PD-L1+ TMP from HLA+ TMP [%]
ND
Naïve
Control
2 Gy

Figure S4

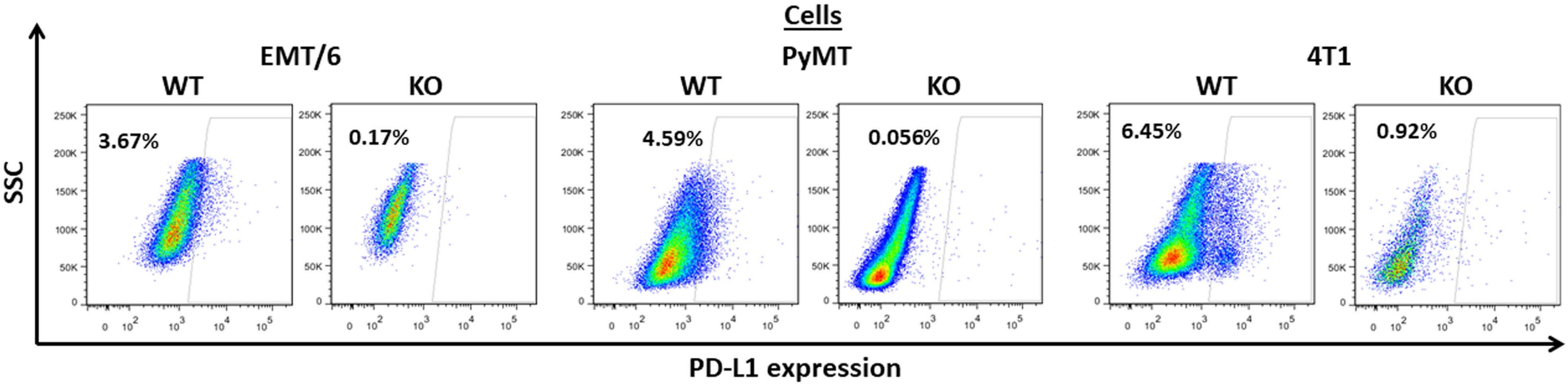
Cells
EMT/6
PyMT
4T1
WT
KO
WT
KO
WT
KO
3.67%
0.17%
4.59%
0.056%
6.45%
0.92%
SSC
PD-L1 expression

Figure S5

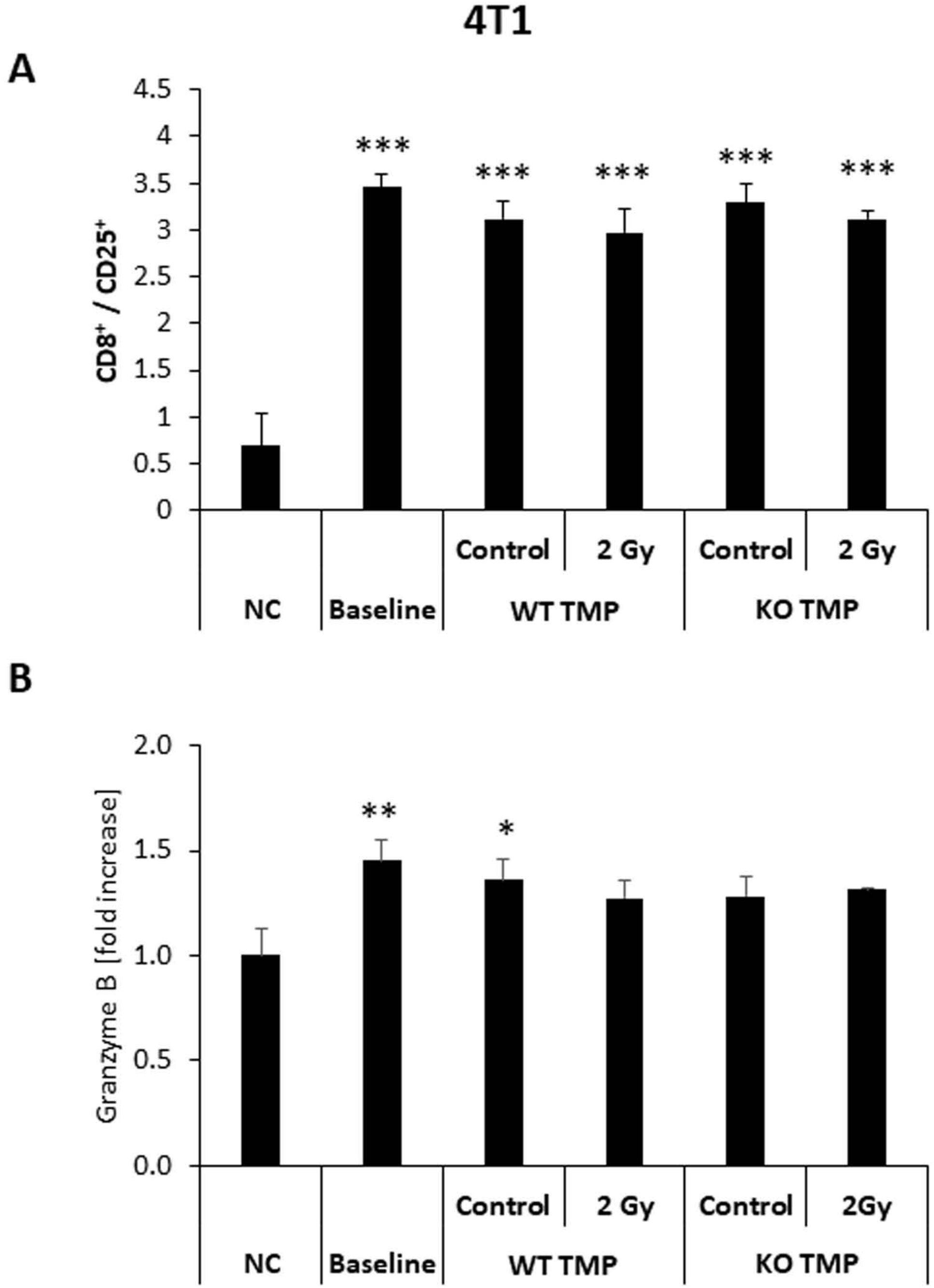
4T1
A
CD8⁺ / CD25⁺
4.5
4
3.5
3
2.5
2
1.5
1
0.5
0
***
***
***
***
***
Control
2 Gy
Control
2 Gy
NC
Baseline
WT TMP
KO TMP
B
Granzyme B [fold increase]
2.0
1.5
1.0
0.5
0.0
**
*
Control
2 Gy
Control
2Gy
NC
Baseline
WT TMP
KO TMP

Figure S6

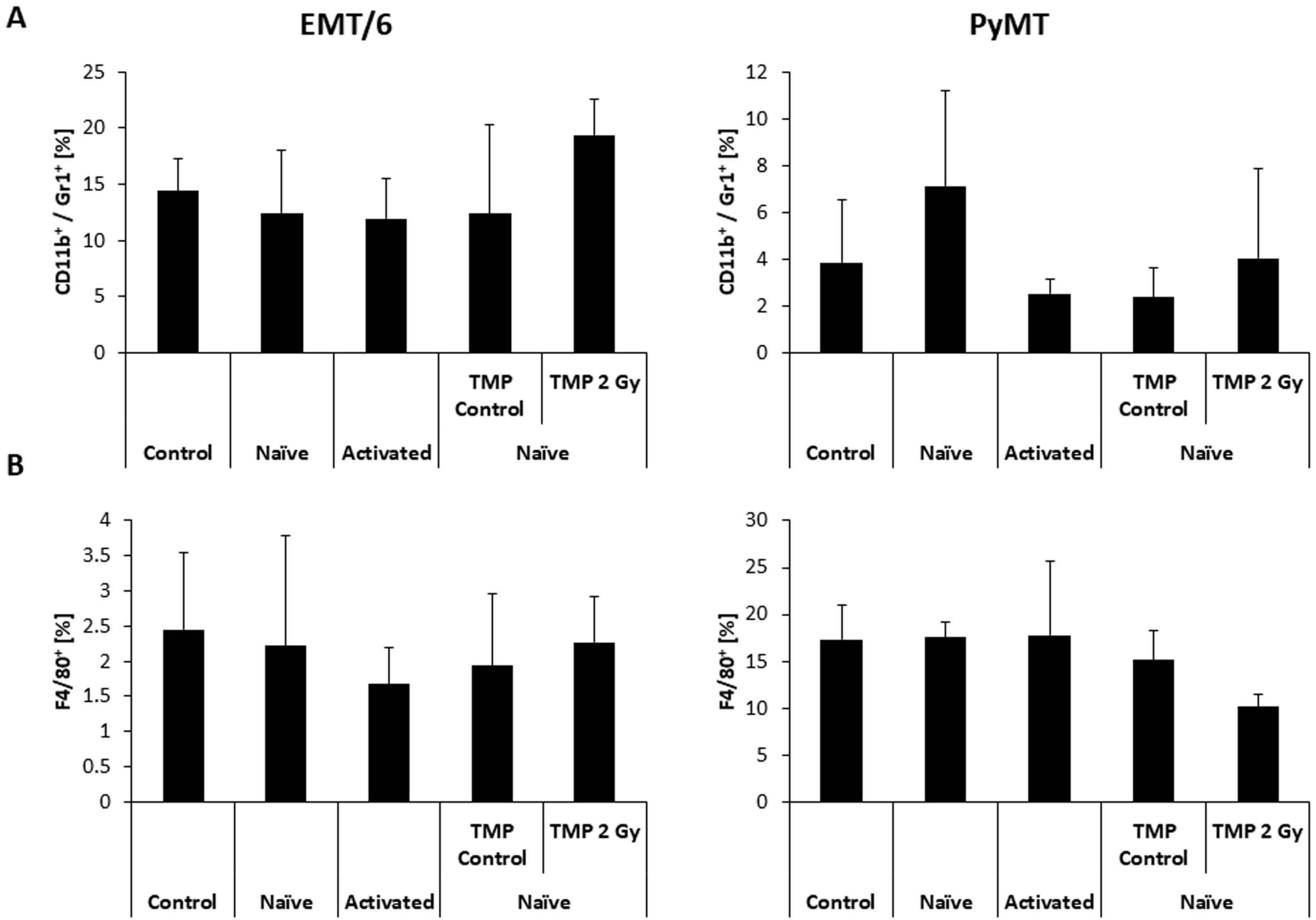

A
EMT/6
PyMT
CD11b+ / Gr1+ [%]
25
20
15
10
5
0
12
10
8
6
4
2
0
TMP Control
TMP 2 Gy
Control
Naïve
Activated
Naïve
B
F4/80+ [%]
4
3.5
3
2.5
2
1.5
1
0.5
0
30
25
20
15
10
5
0

Figure S7

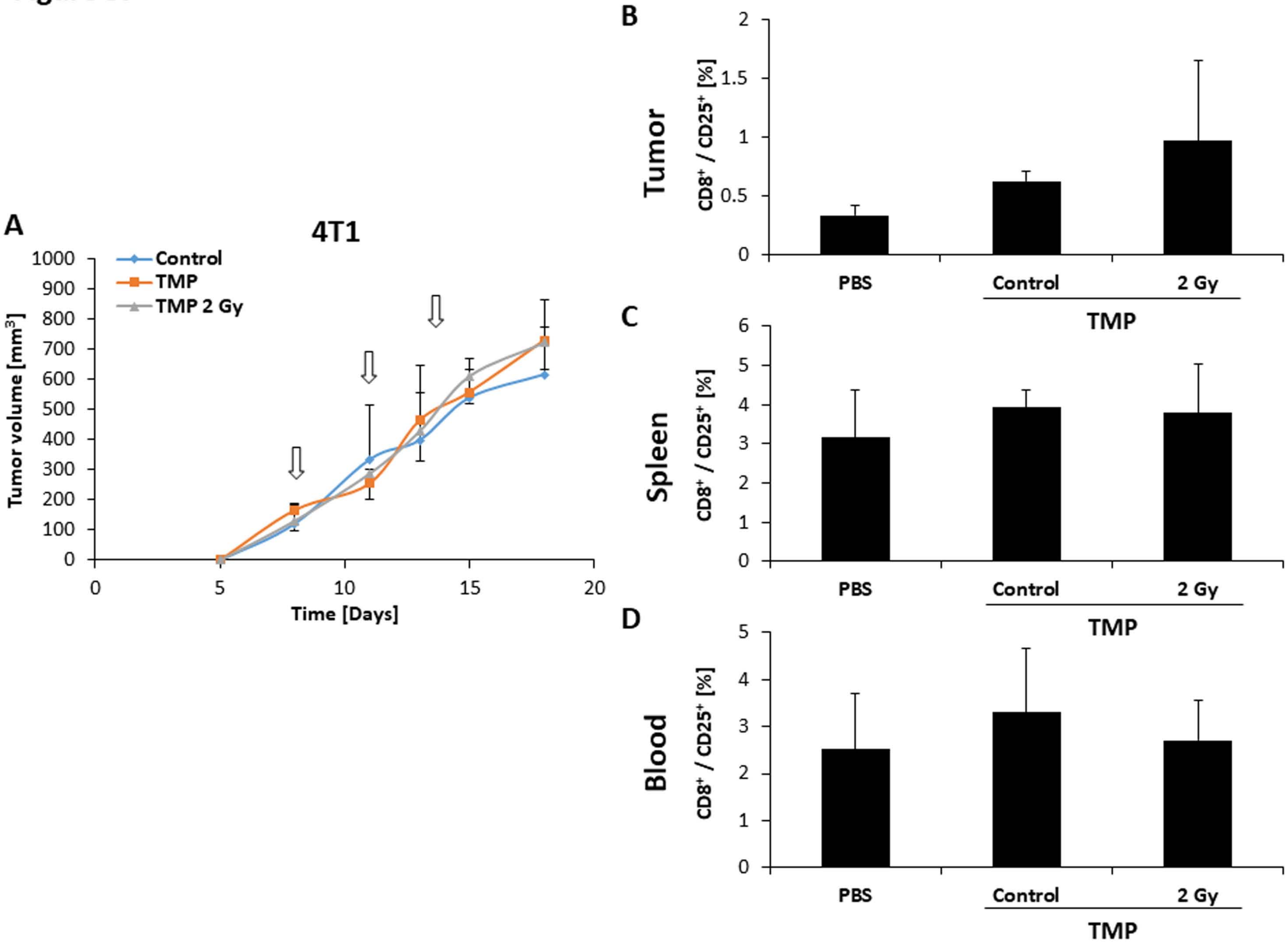
A
4T1
Control
TMP
TMP 2 Gy
Tumor volume [mm3]
1000
900
800
700
600
500
400
300
200
100
0
0
5
10
15
20
Time [Days]
B
Tumor
CD8+ / CD25+ [%]
2
1.5
1
0.5
0
PBS
Control
2 Gy
TMP
C
Spleen
CD8+ / CD25+ [%]
6
5
4
3
2
1
0
PBS
Control
2 Gy
TMP
D
Blood
CD8+ / CD25+ [%]
5
4
3
2
1
0
PBS
Control
2 Gy
TMP

Figure S8

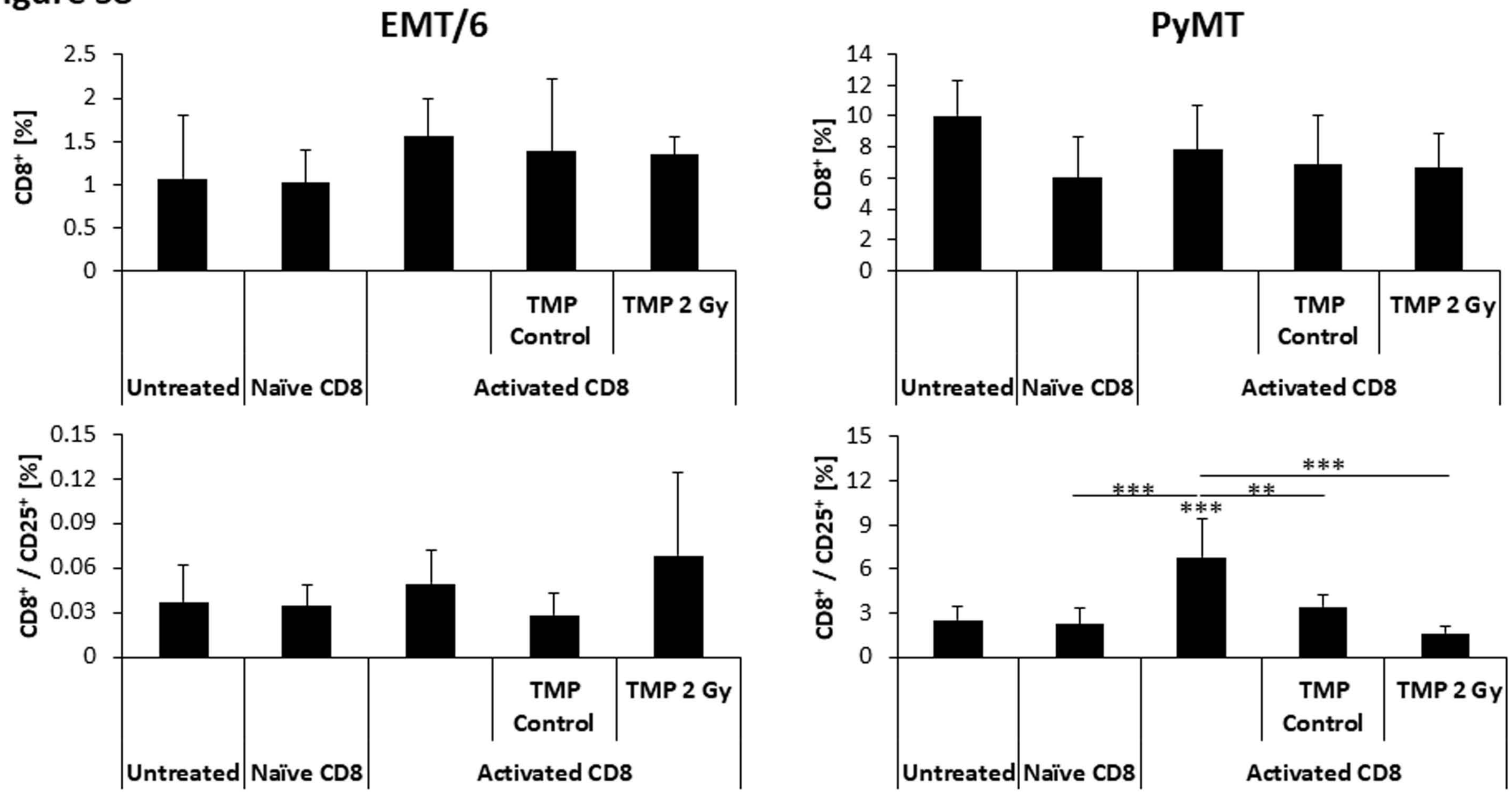

EMT/6
PyMT
CD8⁺ [%]
CD8⁺ / CD25⁺ [%]
2.5
2
1.5
1
0.5
0
14
12
10
8
6
4
2
0
0.15
0.12
0.09
0.06
0.03
0
15
12
9
6
3
0
***
**
***
***
TMP Control
TMP 2 Gy
Untreated
Naïve CD8
Activated CD8

Figure S9

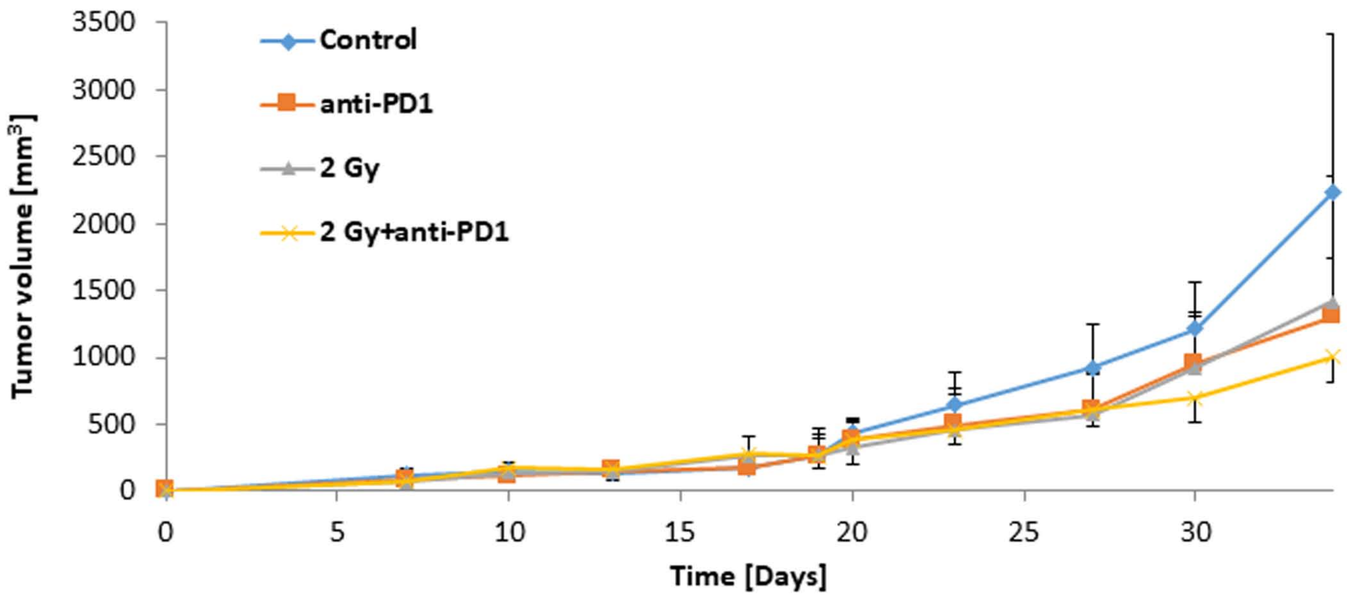
Control
anti-PD1
2 Gy
2 Gy+anti-PD1
Tumor volume [mm3]
3500
3000
2500
2000
1500
1000
500
0
0
5
10
15
20
25
30
Time [Days]
