# Supplementary material for: Microparticles from tumors exposed to radiation promote immune evasion in part by PD-L1
Source: Oncogene. 2019 Aug 29;39(1):187–203. doi: 10.1038/s41388-019-0971-7 (PMC6937213; doi:10.1038/s41388-019-0971-7)
Supplement: Supplementary file 2 — Supplemental Tables S1-S8 [file 41388_2019_971_MOESM2_ESM.pdf]

Supplemental table 1: Mass specrometry-based proteomic analysis of TMPs originated from EMT/6 and PyMT cells

LQF Intensity EMT6 Control1	LQF Intensity EMT6 Control2	LQF Intensity EMT6 Control3	LQF Intensity EMT6 RT1	LQF Intensity EMT6 RT2	LQF Intensity EMT6 RT3	LQF Intensity PYMT Control1	LQF Intensity PYMT Control2	LQF Intensity PYMT Control3
32.50142	32.57317	32.4775	32.78293	32.78098	32.75732	32.89374	32.91171	32.83281
34.38278	34.22647	34.38921	34.28967	34.30334	34.38278	35.31352	35.32697	35.26192
33.74535	33.80424	33.66289	34.09899	34.08322	34.14528	35.83045	35.8186	35.75782
31.68395	31.76525	31.62418	31.33031	31.25912	31.33031	31.98142	31.91883	31.92592
36.12809	36.19564	36.08124	36.69177	36.63882	36.79217	38.95535	39.01109	39.06477
32.04791	32.08639	32.03157	31.59779	31.55728	31.59335	31.44825	31.30878	31.39305
33.3956	33.56411	33.43341	33.27586	33.26192	33.26192	31.25912	31.23649	31.22504
32.92238	32.98651	32.95913	32.84221	32.8347	32.83658	32.89374	32.78488	32.82335
32.90992	32.96774	32.71088	32.3956	32.27864	32.52262	23.94949	23.67573	24.50446
33.64151	33.69437	33.58665	32.80424	32.81574	32.85712	32.82904	32.78098	32.7272
31.68813	31.87555	31.71702	31.71702	31.74935	31.72517	31.64581	31.62418	31.66289
31.03486	31.16639	31.03486	30.98821	30.9746	31.02168	31.64581	31.59335	31.60664
30.42342	30.64581	30.61105	30.41337	30.36202	30.34096	31.19601	31.13001	31.0927
34.52962	34.55842	35.20478	35.63612	34.41463	34.47629	20.17035	23.73892	22.54745
30.5389	30.71293	30.47267	30.38278	30.34096	30.37244	30.60222	30.54812	30.5389
31.81574	31.89735	31.84969	31.61105	31.61544	31.61982	31.74134	31.71293	31.71293
28.81421	28.87701	28.37451	28.80193	28.70965	28.61456	30.09899	30.17236	29.94
32.13001	32.28967	32.23934	31.74134	31.61982	31.80809	26.99766	25.98251	26.45356
33.11613	33.08163	33.2004	32.82525	32.85341	32.91528	32.79266	32.84782	32.80617
33.28967	33.31689	33.78488	33.45807	33.40831	33.40831	20.2535	22.55684	20.1946
33.63071	33.65224	33.75533	33.58665	33.58665	33.65224	33.65224	33.64151	33.61982
31.94	32.08639	31.91528	32.13614	32.14224	32.12694	32.90275	32.8792	32.89193
30.08639	30.27586	30.09899	30.19601	30.19601	30.18423	30.62854	30.57542	30.57542
31.82335	31.80424	31.84221	31.49667	31.54812	31.49667	32.75732	32.80424	32.89374
30.83092	30.9746	30.87555	30.48232	30.46295	30.56638	31.31959	31.29242	31.27031
32.92415	32.8347	32.96258	32.88649	32.94349	32.8792	33.72517	33.74535	33.80424
33.10368	33.01339	33.12848	33.26192	33.26192	33.34361	33.7751	33.79459	33.78488
33.20624	33.19601	33.26192	32.2106	32.2106	32.21639	30.75334	30.78488	30.7211
32.2814	32.26752	32.35678	32.58442	32.58889	32.57768	33.33031	33.33031	33.24785
33.87004	33.78488	33.91528	33.49429	33.50616	33.5527	32.88467	32.76327	32.79071
33.43341	33.5527	33.49429	33.50616	33.51794	33.5527	32.76327	32.73529	32.67556
30.00838	30.18423	29.92592	30.23078	30.13614	30.19601	30.12386	30.08639	30.02168
29.86821	29.71457	29.68479	29.31743	29.27808	29.44332	24.90885	23.0258	24.26069
30.44332	30.59335	30.41337	30.45317	30.40324	30.4334	29.55545	29.40121	29.55545
22.18253	23.00196	22.85567	22.78556	21.67573	22.90793	30.08639	29.91171	30.20769
33.35678	33.34361	33.40831	33.59779	33.69437	33.70471	33.35678	33.36984	33.38278
27.84446	26.77706	27.43936	26.78955	26.93157	24.85281	30.94	30.86083	31.04791
32.49905	32.53659	32.51559	31.90813	31.91171	31.9746	31.4919	31.57542	31.48712
31.94349	32.04466	32.06085	31.48712	31.51089	31.52496	31.20769	31.20769	31.25912
30.79266	31.0544	30.80039	30.94	30.89012	30.87555	30.73731	30.7692	30.65438
36.97417	36.82809	36.99538	36.6116	36.66554	36.63882	36.36496	36.38439	36.36169
33.05278	33.09428	33.08163	33.51794	33.52962	33.51794	33.24785	33.15587	33.23363
31.94698	32.12078	31.95046	32.03485	31.97117	32.04791	30.67135	30.55728	30.61105
34.77999	34.47629	34.93737	34.51794	34.64151	34.60885	34.50024	34.64151	34.43341
31.09899	31.26472	31.04791	31.03486	30.98821	31.00169	29.82335	29.89157	29.76129
31.80809	31.84969	31.84221	32.48712	32.50142	32.59557	32.48712	32.47024	32.45317
32.50142	32.62418	32.53659	32.61982	32.63504	32.64367	32.43092	32.4919	32.42091
30.33031	30.5389	30.27586	30.4334	30.37244	30.44332	30.2535	30.33031	30.14831
28.87408	28.69975	28.78955	29.92592	29.91171	29.99496	30.4334	30.41337	30.55728
33.0927	33.06085	33.0927	33.03321	32.98651	33.00001	33.2135	33.20624	33.20332
31.08005	31.17831	30.91883	31.16639	31.23649	31.23078	31.25912	31.20186	31.16639
29.1866	29.31959	29.13124	29.24672	29.26472	29.33245	29.75812	29.82941	29.84146
36.11458	35.98905	36.07328	36.07128	36.13958	36.10681	36.1107	36.23006	36.09703
27.78955	27.80809	27.75812	27.91456	27.76446	27.77077	27.80809	28.16039	27.91456
34.21205	34.31689	34.28967	34.76525	34.76525	34.75035	32.81	32.85341	32.88831
30.08639	30.28692	30.03486	30.42342	30.34096	30.41337	29.98142	29.92592	29.76762
31.40832	31.45807	31.52496	31.38792	31.28692	31.26472	31.86452	31.91528	31.86452
28.82639	29.08891	28.72598	28.98685	28.97869	29.05569	29.66966	29.57001	29.61456
29.61456	29.62331	29.42542	29.72598	29.75015	29.7162	30.59335	30.67976	30.59335
35.36659	35.38278	35.386	35.28278	35.32362	35.31689	35.19381	35.11846	35.15285
30.66289	30.77706	30.7211	30.55728	30.58441	30.61105	30.80039	30.67976	30.67976
30.44332	30.6372	30.50142	29.85192	29.69644	29.91171	29.77706	29.65096	29.6764
30.95394	31.06728	31.0414	31.14224	31.11148	31.11768	31.98821	31.91171	31.95046
28.91741	29.20303	28.98142	28.68979	28.68311	28.72923	29.58799	29.48424	29.51277
30.94	31.06085	30.9746	30.87555	30.91171	30.94698	28.4512	28.63201	28.22159
30.81574	30.93298	30.88285	30.88285	30.86083	30.81574	30.52028	30.52962	30.46295
29.04791	29.08891	28.94	29.18187	29.08385	29.15557	29.78644	29.86673	29.78019
31.66712	31.63288	31.81192	31.89374	31.89735	31.95394	31.86083	31.87555	31.91883
29.69146	29.91171	29.69644	29.4919	29.41337	29.3452	28.44332	28.34096	28.43539
30.77706	30.76129	30.72923	31.27031	31.27586	31.26472	31.44825	31.45317	31.40324
30.34096	30.35153	30.51089	30.44332	30.39305	30.44332	31.08639	31.38278	31.17831
28.1795	27.97596	27.92025	27.0296	27.39917	27.5969	30.80809	30.91171	30.89735
29.58082	29.56638	29.5389	29.46684	29.51277	29.40933	30.93298	30.95394	30.81574
30.52962	30.70471	30.52962	30.42342	30.41337	30.34096	30.61982	30.7211	30.44332
31.51559	31.44825	31.44332	32.14224	32.13919	32.15738	32.46782	32.48232	32.46539
33.38278	33.51794	33.54121	33.0927	33.16339	33.06085	33.81383	33.74535	33.78488
29.82487	29.89735	29.94	29.73731	29.85638	29.89446	30.33031	30.4919	30.31959
28.37865	28.52776	28.06085	29.02433	29.00571	28.76129	29.89735	29.94	29.86083
29.55179	29.66797	29.58262	29.40324	29.33458	29.30008	30.00838	29.87262	30.04791
30.03486	30.04791	29.84446	29.98142	29.99496	29.94	29.30443	29.54444	29.28692
30.33031	30.33031	30.4334	30.67135	30.71293	30.86821	25.45826	25.1488	25.78207
29.391	29.57001	29.35363	29.38895	29.38072	29.31095	29.509	29.54995	29.49762
29.54995	29.71457	29.54444	29.61105	29.60929	29.55728	30.61982	30.66289	30.62854
31.44825	31.45807	31.45807	31.28692	31.38792	31.40832	31.56638	31.55728	31.54352
31.00169	31.12386	31.17236	31.35153	31.32496	31.40832	29.49572	29.59335	29.60045

30.33031	30.34096	30.23078	29.80655	29.68311	29.67472	29.12386	28.95671	28.98956
31.19013	31.11768	31.08639	29.92592	29.31743	30.07368	NaN	22.8839	21.46762
30.61982	30.59335	30.54812	30.51089	30.46295	30.54812	29.70306	29.75653	29.81421
32.85527	32.91705	33.00336	32.4919	32.4007	32.44825	32.81955	32.83658	32.8347
30.34096	30.47267	30.40324	30.85341	30.73731	30.83846	31.56638	31.4334	31.37762
36.58385	36.47327	36.55556	36.65224	36.67871	36.70471	36.37794	36.34856	36.41148
31.76129	31.81574	31.90096	31.38792	31.31959	31.33565	29.41337	29.33031	29.48424
28.11148	28.15557	28.04531	28.27142	28.23535	28.12633	27.76446	27.90885	27.77706
29.13369	29.19366	28.96499	29.15074	29.01903	28.99226	28.93719	28.79885	28.82335
29.91171	30.11148	30.06085	30.2535	30.19601	30.23078	30.11148	30.02168	30.11148
29.60576	29.77706	29.65779	29.48616	29.57542	29.54444	29.258	29.13614	29.19835
31.74134	30.40324	31.44332	30.82335	31.34625	31.25912	23.18101	21.48738	23.57542
28.81726	28.84446	28.79576	29.12633	29.07112	29.03223	29.60222	29.82031	29.52776
29.258	29.51277	29.36202	29.20769	29.30008	29.27364	29.87408	29.88867	29.83696
31.94349	32.09899	32.08322	31.68395	31.66289	31.69229	31.7211	31.57993	31.61544
28.4073	28.7357	28.52028	28.99766	28.92592	28.89446	28.91741	29.3452	28.89446
32.98142	32.8792	33.07208	32.45807	32.48951	32.44825	32.06085	32.09899	32.07048
30.95394	31.16639	31.13614	30.95394	30.86083	30.88285	31.50616	31.5389	31.4919
31.0927	31.2135	31.14224	31.02828	30.95394	31.06728	31.25912	31.18423	31.18423
30.04791	30.23078	30.08639	30.28692	30.26472	30.24218	29.38895	29.48424	29.42342
28.49381	28.53519	28.59335	28.29351	28.22619	28.31527	27.66628	27.84446	28.19835
32.22791	32.20186	32.16039	31.96086	31.92238	31.96086	29.92592	29.89735	29.83394
28.98414	29.29789	29.08639	29.26249	29.2957	29.22159	29.391	29.27364	29.21002
31.61982	31.67976	31.6501	32.04466	32.01837	31.90455	31.5389	31.47267	31.52496
30.18423	30.28692	30.26472	30.23078	30.27586	30.24218	31.28692	31.29242	31.25912
28.19366	28.3452	28.20303	28.05051	28.01903	27.97049	27.77706	27.99226	27.98685
30.80039	30.77706	30.75334	30.66289	30.61105	30.67976	30.86821	30.85341	30.82335
27.72598	27.99766	27.63201	27.93157	27.95394	28.06599	28.20769	28.24445	28.20303
30.61982	30.80039	30.65438	30.41337	30.50142	30.52028	30.78488	30.75334	30.76129
31.50142	31.61982	31.54352	31.19601	31.20186	31.18423	30.65438	30.58441	30.73731
30.91883	30.94698	30.94698	30.80039	30.88285	30.89012	31.21928	31.0414	31.0927
30.70471	30.73731	30.60222	30.61105	30.66289	30.61982	30.99496	30.93298	30.88285
30.48232	30.68813	30.48232	31.16639	31.18423	31.12386	31.19601	31.20186	31.16639
30.78488	30.61982	30.81574	30.47267	30.42342	30.42342	30.89735	30.85341	30.98821
28.27142	28.55728	28.32817	28.20303	28.1795	28.15557	29.15316	29.08891	29.09648
33.63071	33.72517	33.68395	32.79653	32.7692	32.76525	29.81421	29.70965	29.87262
30.24218	30.46295	30.27586	30.61105	30.62854	30.64581	30.41337	30.30878	30.40324
30.36202	30.4919	30.28692	30.20769	30.34096	30.38278	30.00838	29.92592	29.95394
30.62854	30.82335	30.62854	29.88867	29.87555	29.99496	30.66289	30.67135	30.54812
30.62854	30.72923	30.6372	30.74535	30.74535	30.78488	30.19601	30.20769	30.28692
32.49429	32.5038	32.55728	32.30878	32.20478	32.25068	31.43837	31.59779	31.39816
30.30878	30.4334	30.24218	30.24218	30.27586	30.19601	29.46684	29.48808	29.34731
29.30226	29.56092	29.30878	28.23991	28.16039	28.24898	30.80809	30.78488	31.02828
27.47846	27.5389	27.45513	27.43141	27.49381	27.39917	27.83846	27.69975	27.64581
30.96774	31.0414	31.0544	30.70471	30.70471	30.67135	30.84595	30.76129	30.73731
30.29789	30.07368	30.28692	30.16039	30.07368	30.20769	30.65438	30.86821	30.76129
30.68813	30.81574	30.75334	30.58441	30.57542	30.65438	29.19601	29.14102	29.34942
30.30878	30.27586	30.35153	30.51089	30.58441	30.76129	29.71947	29.76288	29.66119
29.05827	29.09396	28.98956	29.09396	29.12386	29.06085	29.56819	29.38278	29.48232
28.74855	28.92875	28.42742	28.80809	28.81115	28.86231	27.42342	27.66628	28.45513
29.62506	29.64924	29.69644	29.03748	29.08639	29.06856	28.27586	28.258	28.29351
29.84146	29.56638	30.08639	28.23991	27.63893	28.54995	26.38772	25.67303	27.07112
33.78488	33.69437	33.71498	34.05924	34.12232	34.02663	33.79459	33.81383	33.71498
34.10681	33.95913	34.15285	34.16788	34.16788	34.24785	34.03485	34.00169	34.02663
31.14224	31.16039	31.08005	30.86821	30.89735	30.84595	30.69644	30.74535	30.65438
29.16997	29.4073	29.12879	29.43738	29.509	29.49762	29.1795	29.24218	29.27586
29.96774	30.19601	30.09899	30.61982	30.71293	30.71293	29.83092	29.82183	29.7162
33.47024	32.83092	33.47024	33.69437	33.7751	33.68395	33.56411	33.75533	33.54121
29.33031	29.48232	29.32817	29.6372	29.44134	29.59156	29.58441	29.44727	29.41941
29.35363	29.5389	29.41135	29.26472	29.21928	29.20303	29.09396	29.26919	29.12139
29.88577	30.06085	29.83244	29.92592	29.96774	29.95394	30.36202	30.26472	30.33031
28.60399	28.99226	28.54628	28.80193	28.68979	28.61456	29.58441	29.4073	29.28692
29.61632	29.54812	29.55179	29.80962	29.82487	29.71947	29.95394	29.99496	29.92592
28.52776	28.58978	28.43539	28.7162	28.66289	28.76446	28.63547	28.56456	28.57903
26.35448	26.43619	25.77706	26.90885	26.77706	26.61807	29.13369	28.74535	29.48999
30.51089	30.6372	30.57542	30.30878	30.31959	30.35153	31.14224	31.10525	31.0414
32.82904	32.9574	32.86267	32.76129	32.81383	32.77706	31.76525	31.7692	31.80039
33.33031	33.44579	33.43341	33.36984	33.24785	33.31689	31.91883	32.01505	31.87921
32.4775	32.57768	32.65651	33.11613	33.08797	33.10212	31.81192	31.74134	31.72517
29.84595	30.08639	29.94	30.19601	30.11148	30.14831	30.24218	30.14831	30.21928
29.22389	29.46879	29.20769	28.70965	28.62157	28.70636	29.34308	29.21002	29.391
28.44727	28.63893	28.48616	28.86231	28.79885	28.64924	27.94838	28.02433	27.9428
30.82335	31.01505	30.92592	31.16639	31.26472	31.20186	30.84595	30.84595	30.69644
32.36463	32.42592	32.49429	32.14224	32.18423	32.17236	32.54812	32.40578	32.55041
28.97596	29.00035	28.92592	28.86821	28.81726	28.94838	28.70636	28.5969	28.57181
30.41337	30.60222	30.52962	30.00838	29.95394	29.98142	28.72598	28.66966	28.85043
31.74134	31.91883	31.75732	31.31419	31.24785	31.28692	29.45317	29.3869	29.60929
29.37451	29.52402	29.22159	29.44134	29.33671	29.3452	29.98142	29.86083	29.92592
29.27586	29.43936	29.30661	29.77077	29.71129	29.60045	29.92592	29.7162	29.84745
28.05569	28.27142	28.08132	28.39509	28.21697	28.23535	28.47846	28.58262	28.31959
29.18423	29.16997	29.22159	29.06343	29.22849	29.13124	29.70471	29.66628	29.68479
28.75494	28.91171	28.72598	28.60399	28.58262	28.61456	28.52028	28.66628	28.44727
31.97117	32.06407	32.08322	32.2135	32.22791	32.2814	32.06728	32.04466	31.95394
29.35993	29.2535	29.2007	29.36411	29.48999	29.53148	29.75653	29.99496	29.83846
30.82335	30.93298	30.69644	31.38278	31.39305	31.39816	31.43837	31.34096	31.40324
31.48712	31.55728	31.57091	32.13919	32.26472	32.29242	32.61105	32.69851	32.53194
31.39305	31.34096	31.40324	31.77706	31.75732	31.84595	31.45807	31.56638	31.5389
32.79266	32.91883	32.82904	32.7272	32.81955	32.78098	31.67976	31.74935	31.73328

31.29242	31.28692	31.16039	31.16039	31.17831	31.14831	29.67472	29.64238	29.52776
28.84446	28.94559	28.83244	28.60399	28.51653	28.54259	29.30008	29.39305	29.41538
26.93157	26.23535	27.29789	27.34942	26.71293	26.76446	28.39509	29.04009	28.57903
29.46684	29.44727	29.35153	29.104	29.18187	29.13614	29.32603	29.13124	29.16997
27.34096	27.34096	26.86231	27.32389	27.13124	26.95394	27.96499	27.89157	27.92592
32.64796	32.3802	32.71088	32.9574	33.02663	32.66076	25.77706	25.93832	26.51803
30.68813	30.83092	30.52962	30.4919	30.52028	30.52962	28.45513	28.35363	28.31959
35.83281	36.01837	35.86544	35.72517	35.70985	35.66023	34.13001	34.04303	34.03485
31.78097	31.78488	31.77313	32.21928	32.2135	32.25068	31.86083	31.8347	31.88649
31.28692	31.35153	31.31959	31.61544	31.66289	31.69229	31.45317	31.38278	31.52962
27.3662	27.67976	27.29789	27.55362	27.43936	27.39917	28.89157	28.89157	28.90598
32.27309	32.37762	32.20186	32.12078	32.14224	32.19308	29.67472	29.66119	29.67472
30.09899	29.99496	30.04791	30.03486	29.89735	30.13614	30.08639	30.14831	30.02168
28.81726	29.01903	28.86526	29.33245	29.12633	29.19131	27.58978	27.85043	27.62506
28.37451	28.41941	28.42342	28.43141	28.47073	28.42742	26.9428	27.22619	27.75175
29.3452	29.37865	29.30878	29.1866	29.42542	29.2803	29.43936	29.65949	29.29351
29.02168	29.17236	29.01372	28.78955	28.80193	28.71293	29.27142	29.35363	29.38895
29.48999	29.59868	29.57723	29.76129	29.78176	29.77077	30.18423	30.13614	30.13614
31.82335	31.80809	31.7692	31.70882	31.68395	31.7211	31.34096	31.2535	31.41337
27.71947	26.80193	27.11148	26.83846	27.08132	26.89735	27.77077	27.71293	27.72598
31.61982	31.65864	31.54352	31.21928	31.28692	31.2814	31.52028	31.37762	31.37762
26.44095	23.56384	26.65949	26.30138	26.28207	26.30835	29.60222	29.33458	29.34096
29.89302	30.78488	29.96774	30.96086	29.72923	29.84296	19.92434	22.98815	21.78756
31.13614	31.27586	31.22504	31.4919	31.61544	31.5527	31.22504	31.25912	31.23078
30.08639	30.18423	30.12386	30.21928	30.33031	30.27586	28.09648	28.24445	28.11148
31.45317	31.30334	31.45807	31.81955	31.74535	31.80809	31.52496	31.46782	31.53427
28.82031	29.01903	28.83244	28.77392	28.96224	28.75494	29.69478	29.71457	29.76604
33.35678	33.38278	33.48232	33.05278	32.96086	32.90455	32.79071	32.98651	32.85712
31.29789	31.43837	31.37244	31.30334	31.19013	31.20769	31.08005	31.02828	31.0927
30.16039	30.11148	30.24218	29.78488	29.87262	29.89735	27.19835	27.82639	28.15557
29.53148	29.52962	29.66628	29.54628	29.69975	29.60399	30.14831	30.18423	30.17236
30.26472	30.45317	30.36202	29.92592	29.94	30.02168	29.85638	29.75812	29.89735
28.42742	28.72923	28.15557	28.43936	28.85043	28.39917	29.19601	28.69312	29.05051
28.37865	28.70965	28.06085	28.47846	28.51653	28.35783	27.72598	27.44727	27.34942
29.08132	29.48808	29.21466	29.55545	29.32389	29.45709	29.71293	29.71784	29.43539
30.62854	30.59335	30.46295	31.2814	31.34625	31.37244	31.46782	31.48712	31.53427
29.03223	28.86231	28.81726	29.35153	29.40527	29.52962	28.71293	28.88285	28.70965
28.11148	28.30226	28.10149	28.13124	28.18896	28.258	28.51653	28.56456	28.63201
29.08639	29.18896	28.87115	29.37865	29.47846	29.34942	32.03485	32.00169	32.02499
29.92592	30.06085	29.91171	29.72923	29.91171	29.86526	29.10899	29.07368	29.08639
29.1866	29.35993	29.0531	28.63547	28.65949	28.509	29.50332	29.26919	29.28692
31.64151	31.65864	31.71293	31.77313	31.87555	31.93298	31.62418	31.64151	31.65864
30.28692	30.44332	30.4334	30.98821	30.94698	30.91883	29.19131	29.24218	29.34308
29.18896	29.26919	29.21697	29.96774	29.91171	29.91171	29.88722	29.7911	29.81421
30.83846	30.94	31.0414	30.85341	30.83846	30.94	30.96086	31.0414	30.86821
30.02168	30.16039	30.02168	29.79885	29.80039	29.84146	28.74855	28.76129	28.87701
28.0296	28.41941	28.16997	27.83244	27.67976	27.83244	30.12386	30.04791	29.89735
30.44332	30.45317	30.46295	30.23078	30.2535	30.2535	29.99496	29.95394	29.84595
29.76129	29.78332	29.71457	29.63374	29.6372	29.73085	28.83244	28.62854	28.85638
30.18423	30.13614	30.2535	30.5389	30.46295	30.46295	30.87555	30.88285	30.86821
30.59335	30.62854	30.80039	30.27586	30.23078	30.24218	30.26472	30.35153	30.30878
28.01903	28.14102	28.09648	28.13614	28.23991	28.13614	28.43141	28.5389	28.68646
27.68646	27.18896	27.44727	27.13124	27.27142	27.43141	28.52028	28.41538	28.53519
28.84745	29.13858	29.03486	29.26696	29.27586	29.34096	29.42342	29.43141	29.45513
28.86821	29.08639	28.91741	28.88285	28.91741	28.85935	28.46684	28.48999	28.4746
27.66628	27.67303	27.90885	28.92875	29.0427	28.88867	29.64753	29.74214	29.55179
28.18423	28.21697	28.14589	28.38278	28.41135	28.56819	29.27586	29.21466	29.13614
34.23363	34.09113	34.52962	33.78488	33.82335	33.84221	32.67766	32.75334	32.81
34.42091	34.41463	34.48832	34.49429	34.58665	34.61982	33.89735	34.03485	34.00169
29.42342	29.59868	29.41135	29.4919	29.43539	29.5389	29.57001	29.47846	29.43141
33.68395	33.33031	33.73529	33.06728	33.07846	32.90992	31.36724	31.39816	31.40832
28.40324	28.36202	28.35783	28.5862	28.34942	28.33245	28.94559	28.78019	28.89735
28.48232	28.29789	28.19835	28.19366	28.16519	28.00838	28.73247	28.85638	28.64581
27.42342	28.34942	27.47073	28.22619	28.15074	28.29789	25.03591	25.33756	25.45199
27.92592	28.04531	28.04009	28.21697	28.36202	28.09144	28.64581	28.65267	28.75494
27.89735	28.13614	27.83244	28.19835	28.29789	28.16997	28.48616	28.46295	28.391
29.64924	29.4453	29.52962	29.3869	29.4512	29.40121	29.7973	29.74535	29.72923
27.98685	28.19366	27.95947	28.02433	27.88577	28.0296	29.4334	29.60929	29.41337
28.57542	28.93438	28.52776	28.62506	28.63547	28.65267	28.75175	28.4746	28.4746
29.33245	29.55362	29.41337	29.40527	29.43539	29.33884	28.75812	28.85638	28.81115
28.44332	28.43936	28.75175	28.65608	28.3869	28.29351	26.1776	27.14102	26.86231
31.18423	31.34096	31.16639	31.93649	31.92592	31.91528	31.56184	31.57091	31.50142
30.83092	31.08639	30.93298	30.72923	30.80809	30.67976	30.67976	30.61105	30.60222
28.27586	28.65949	28.3869	28.26696	28.31095	28.24445	27.91456	28.23991	27.93719
29.16997	29.44727	29.21928	28.87701	28.82941	28.95116	29.32817	29.45905	29.31095
31.34096	31.43837	31.40324	31.51089	31.50142	31.40324	30.4334	30.33031	30.4334
29.94	30.20769	29.91171	29.96774	29.86378	29.82639	31.41337	31.32496	31.30334
29.02168	28.99496	29.10899	28.7162	28.56092	28.68311	28.49762	28.54995	28.5389
30.84595	31.04791	30.89012	30.7211	30.7211	30.70471	30.83846	30.61982	30.6372
29.09396	29.01903	29.02697	28.81115	28.76762	28.72273	29.92592	29.92592	29.83394
25.85994	27.10149	26.73892	27.22619	27.21697	26.57542	28.45513	29.35153	28.7162
27.5389	27.77706	27.47846	27.80809	27.74535	28.02433	29.4174	29.17236	29.31095
NaN	23.1379	NaN	NaN	NaN	NaN	27.86821	27.9428	28.65267
32.37762	32.41589	32.34096	31.79266	32.00169	31.92945	32.0414	32.11148	32.2106
29.39305	29.59156	29.30226	29.20536	29.07623	28.98956	28.85935	28.90024	28.71947
32.08004	32.15134	32.08322	31.85341	31.93298	31.82714	31.88649	31.75732	31.84595
31.25912	30.83092	31.37244	31.12386	31.11768	31.20186	31.53427	31.60664	31.56638
28.86821	28.92309	28.76762	29.0531	29.18423	29.04531	29.11644	29.06343	29.10649

27.43936	27.66628	27.28912	27.69975	27.67303	27.57542	29.22849	29.31959	29.30226
27.90885	28.43539	28.14589	28.09648	28.22619	27.99226	28.08132	27.95947	28.00838
29.11148	29.07623	29.13369	28.83545	28.92592	28.96499	29.35993	29.4649	29.41538
29.02433	29.13369	28.86231	29.18187	29.35363	29.24445	29.13614	29.16039	29.06856
28.35363	28.51277	28.42742	28.90024	28.87701	29.01372	28.83545	28.93438	28.74535
28.56819	28.65949	28.61456	28.91171	28.91456	28.93438	29.84894	29.87115	29.83996
29.82487	30.04791	29.91171	29.69146	29.76288	29.91171	29.42142	29.53519	29.49381
29.88285	29.8814	29.70141	29.98142	30.00838	30.00838	30.35153	30.24218	30.29789
30.09899	30.17236	30.14831	31.0927	31.19013	31.15436	30.81574	30.57542	30.81574
29.80193	29.76762	29.83996	29.60929	29.65438	29.72273	29.63027	29.54075	29.52589
27.45513	27.80193	27.45513	27.52402	27.67303	27.61105	29.27808	29.25124	29.16279
26.57542	26.64581	26.85043	26.64581	26.92025	26.53297	27.30661	27.30661	27.13124
28.87115	29.37036	28.80809	28.92592	28.45513	28.15074	28.13614	27.22619	26.58978
28.4512	28.58262	28.29789	28.22619	28.43141	28.509	28.64581	29.08132	28.76129
27.47846	27.62506	27.50142	27.78955	27.80193	27.85638	28.08639	27.98142	28.11644
26.89735	27.18896	26.75175	27.2803	27.37451	27.22619	27.58978	27.48616	27.79576
29.7597	29.96774	29.7973	29.55728	29.56456	29.4334	29.0427	28.96224	29.13858
32.66076	32.69437	32.76722	32.68395	32.6372	32.79459	29.09144	28.53148	28.95947
29.06085	29.23535	29.09144	28.90885	28.88867	28.85638	28.35783	28.48232	28.51277
30.47267	30.58441	30.62854	31.20769	31.28692	31.24218	30.4334	30.42342	30.38278
31.69229	31.75732	31.82714	32.55728	32.56865	32.5038	31.57542	31.54352	31.60664
31.24218	31.23649	31.32496	30.83846	30.77706	30.71293	29.66458	29.40527	29.59335
30.29789	30.54812	30.42342	30.24218	30.34096	30.37244	30.31959	30.47267	30.35153
30.48232	30.54812	30.51089	30.4334	30.51089	30.4334	30.16039	30.27586	30.33031
31.0414	31.16639	30.98821	31.0927	31.09899	30.98142	31.26472	31.20769	31.12386
29.22159	29.30008	29.13614	29.2957	29.37036	29.37036	28.99226	29.02697	28.87701
28.51653	28.69644	28.40324	30.30878	30.45317	30.18423	31.73328	31.67556	31.6501
28.78644	29.19131	28.80809	28.37865	28.97869	28.68311	26.17379	25.68378	25.66221
30.44332	30.57542	30.52028	30.00838	30.02168	30.00838	29.92592	29.86673	29.91171
29.13369	29.26919	29.1866	29.26919	29.36202	29.31095	29.99496	29.86231	29.92592
28.75494	28.95671	28.68311	28.72923	28.69644	28.70636	29.30878	29.19131	29.20536
30.57542	30.81574	30.52028	30.45317	30.50142	30.35153	30.00838	30.02168	29.98142
28.18896	27.41538	27.16997	29.18423	29.13614	29.23991	22.52133	22.99162	23.04093
31.38792	31.44332	31.46782	31.61982	31.69644	31.68813	30.66289	30.61105	30.67976
28.83244	29.07623	28.87994	29.0531	29.19601	29.0531	29.09144	28.93157	28.90311
32.92592	32.90455	33.03321	32.74334	32.82714	32.81574	32.69851	32.74735	32.71088
28.04531	28.53519	28.09144	27.9428	28.06599	28.08132	28.41135	28.43936	28.20769
29.13858	29.11892	29.17712	29.46684	29.59513	29.56819	29.08132	29.04531	29.11396
28.34096	28.64581	28.49762	28.63201	28.62157	28.82941	28.75494	28.77392	28.78332
25.42342	26.26964	25.82396	21.91707	19.74878	21.98815	31.29789	31.22504	31.51089
29.83244	29.99496	29.84146	29.36411	29.27808	29.40324	28.12633	28.23078	28.23535
31.35153	31.2814	31.39816	30.69644	30.72923	30.87555	28.78019	28.91741	28.74535
31.22504	31.39305	31.33031	31.61544	31.61544	31.63288	31.42842	31.39305	31.52496
28.10649	27.98685	28.07623	28.07112	28.00838	28.14102	28.32817	28.90311	28.39917
28.08132	28.22619	27.83846	28.11148	28.18896	28.06599	28.68979	28.7357	28.61456
28.01372	28.15074	28.22619	28.03486	27.82639	27.78332	27.65267	27.91456	27.66628
29.03486	29.25124	29.06085	28.63201	28.6764	28.56092	28.15557	28.24445	28.11644
27.26249	27.29789	27.27142	27.21697	27.21697	27.48616	27.14102	27.13124	26.99766
29.61807	29.51841	29.54812	29.59513	29.5969	29.48808	29.98142	30.00838	29.91171
30.07368	30.28692	30.06085	30.82335	30.89735	30.81574	30.52028	30.44332	30.54812
28.34942	28.4512	28.2535	27.75175	27.65949	27.87994	28.04009	27.9428	28.10649
29.34731	29.42142	29.21002	29.43936	29.38895	29.40324	28.43936	28.31527	28.34942
27.56819	28.29789	28.16039	28.49381	28.68979	28.47846	24.58118	25.66763	24.06907
28.16519	28.2535	28.08132	27.98142	27.88577	27.79576	28.0296	28.09144	27.95947
32.01837	32.08639	32.15738	32.03157	31.88649	32.02168	31.76129	31.78097	31.90813
31.30334	31.47267	31.42842	31.22504	31.27031	31.01505	31.77313	31.67976	31.69644
28.31959	28.5389	28.47073	28.70965	28.73247	28.71947	29.30443	29.258	29.29351
27.33245	27.3662	27.10149	27.64581	27.77706	27.67976	28.11148	28.00303	28.12139
28.73247	28.75812	28.82335	28.87994	28.90024	29.00035	29.28692	29.16279	29.21002
29.461	29.5591	29.52776	29.30443	29.26024	29.32603	30.90455	31.00169	30.83092
29.73892	29.87555	29.80193	29.54444	29.57542	29.55545	30.26472	30.03486	29.96774
28.07623	28.41941	28.05569	28.21697	28.05569	28.04531	27.68646	27.97596	27.56092
29.45317	29.45905	29.40121	30.19601	30.30878	30.18423	29.96774	29.94	30.00838
28.06085	28.1795	28.08639	27.90885	28.23991	28.01903	28.29789	28.10649	28.391
31.54812	31.50142	31.51089	31.94349	31.87188	31.92592	31.65438	31.75334	31.65864
28.89157	29.01638	28.76446	29.19131	29.08639	29.13124	28.99226	29.02433	28.94838
28.82941	29.14346	28.89446	29.58441	29.48808	29.46879	28.50142	28.41941	28.49762
30.52028	30.56638	30.61982	30.94698	30.99496	31.03486	30.75334	30.76129	30.85341
24.65949	22.79749	22.37518	21.9061	22.73892	24.6811	28.08132	27.96499	27.91456
27.53148	28.13124	28.60752	26.85043	25.99981	26.92025	23.68646	22.97946	22.71188
22.84422	24.28207	NaN	22.92434	22.1047	NaN	29.62506	29.52962	29.47846
29.91171	29.96774	29.84296	29.78644	29.76446	29.84446	28.42342	28.49762	28.45905
27.1795	27.43141	27.21697	27.43936	27.33245	27.24445	27.75812	27.92592	27.79576
28.06085	28.26696	27.65949	28.31095	28.40324	28.30226	27.509	27.34942	27.391
27.69975	28.62506	28.22619	28.37036	27.83244	28.08132	30.48232	30.72923	30.57542
28.08639	28.21697	28.13614	28.88577	28.76129	28.79576	28.92875	29.09648	28.98956
28.97869	28.49381	28.97596	28.86231	28.84146	28.90885	29.15798	28.93719	29.08639
27.23535	27.13124	27.07112	27.52402	27.55362	27.56819	27.63201	27.52402	27.2803
28.69644	28.69975	28.51653	28.48232	28.5969	28.47846	28.54628	28.54628	28.63201
27.89157	28.04531	27.89735	27.31527	27.47073	27.55362	28.3869	28.21234	28.31527
28.51653	28.31527	28.33245	28.74214	28.78955	28.70965	28.63893	28.9428	28.68646
30.29789	30.29789	30.40324	30.79266	30.76129	30.69644	31.17831	31.24218	31.22504
30.67976	30.72923	30.75334	31.04791	31.17831	31.23649	30.91171	31.00838	30.88285
32.34625	32.35941	32.38278	31.25912	31.31419	31.33565	30.47267	30.52962	30.4919
30.40324	30.33031	30.40324	30.38278	30.18423	30.2535	30.74535	30.52962	30.62854
31.47267	31.59779	31.47267	31.8347	31.85341	31.88285	31.74935	31.66712	31.74535
28.55728	28.58978	28.48999	29.32174	29.27586	29.22849	28.90885	28.84745	28.81421
28.62157	28.67303	28.64924	28.87994	28.95116	28.98685	29.22159	29.09648	29.04531

29.98142	30.17236	30.14831	29.83545	29.88867	29.98142	29.58978	29.708	29.6372
31.7211	31.85712	31.71293	31.89374	31.87188	31.88649	31.52028	31.44332	31.50142
29.74053	29.66628	29.42142	30.44332	30.42342	30.38278	30.64581	30.51089	30.52028
31.57091	31.57993	31.52962	31.83846	31.80039	31.76129	31.51559	31.47267	31.61982
30.83846	30.95394	30.98142	31.01505	31.02168	30.99496	30.89012	30.78488	30.94698
30.16039	30.20769	30.06085	30.66289	30.56638	30.57542	30.94698	30.94698	31.11148
28.10149	28.29789	28.11148	28.11148	28.24445	28.10649	27.85638	27.89157	27.65949
28.86526	29.20769	28.99226	29.04791	28.96499	28.96499	28.71293	28.62157	28.5862
26.76446	27.16039	26.96499	26.93157	27.04009	26.90885	27.5389	27.57542	27.86231
28.76446	28.79885	28.70965	28.73892	28.75494	28.68311	28.69644	28.64238	28.76129
31.81192	31.88285	31.7692	31.34096	31.2535	31.30334	30.78488	30.82335	30.79266
30.87555	30.86083	30.80809	30.54812	30.60222	30.61982	30.40324	30.4919	30.61105
28.49762	28.5862	28.46684	28.83846	28.85043	28.75812	28.95947	28.80809	28.63893
30.16039	30.35153	30.11148	30.50142	30.57542	30.50142	30.44332	30.47267	30.28692
26.2021	25.85519	25.62923	25.85043	24.86231	25.79699	27.47073	27.66628	27.34096
29.98142	30.20769	30.17236	29.98142	30.03486	30.14831	30.18423	30.03486	30.00838
28.98414	29.2535	29.05569	28.90311	28.79576	28.74855	29.23991	29.2957	29.09144
29.4174	28.74214	28.73892	28.391	29.36411	29.64065	NaN	NaN	NaN
29.44332	29.56819	29.37865	28.77077	28.66966	28.64924	27.56092	27.47846	27.69975
29.89735	30.00838	30.08639	29.99496	29.98142	30.11148	29.62331	29.4073	29.52776
27.91456	27.99766	28.01372	28.03486	28.00838	28.05051	27.96499	28.02433	27.90885
30.80039	30.82335	30.68813	30.90455	30.83846	30.91171	31.15436	31.16039	31.04791
26.26785	27.391	26.34435	26.20769	25.80193	25.78955	26.77706	26.78955	26.99766
28.16039	28.04009	28.24898	27.87994	27.5389	27.82031	26.73892	26.82639	27.26249
26.99766	27.22619	27.2803	27.57542	27.83244	27.69312	28.72273	29.11396	28.62157
28.55362	29.01903	28.63201	28.50142	28.69975	28.6764	30.4919	30.39305	30.50142
29.06343	29.1866	29.26472	28.98414	29.05051	29.01638	29.68646	29.60752	29.82335
30.37244	30.58441	30.39305	30.59335	30.60222	30.57542	30.80809	30.80809	30.71293
27.43936	27.5969	27.26249	27.94838	27.83846	27.90311	27.90885	27.85638	27.95394
29.53705	29.6441	29.53334	29.48808	29.49762	29.61982	29.85638	29.88431	29.92592
27.99766	28.07112	28.08132	27.47073	27.75812	27.62506	28.65267	28.81726	28.54628
30.61105	30.78488	30.75334	30.52962	30.50142	30.42342	30.7211	30.60222	30.62854
28.59335	28.3869	28.37036	28.71947	28.79266	28.82941	28.44332	28.34942	28.29789
28.75494	29.05569	28.91456	29.10649	29.12879	29.11892	29.03223	28.78019	28.74535
31.06085	31.10525	31.06085	31.44825	31.40324	31.38278	31.19013	30.96774	31.06728
30.90455	30.95394	30.94	31.2814	31.22504	31.29242	30.74535	30.71293	30.72923
32.13614	31.88285	32.05116	31.86452	32.00169	31.9643	31.11148	31.35678	31.0414
27.9428	28.33245	28.12633	27.93719	28.19835	28.12139	28.77077	28.70965	28.69644
24.10149	22.53801	24.08537	21.7348	21.53564	22.09184	27.93157	27.48616	27.87408
27.39917	27.53148	27.16039	27.23535	27.2803	27.2535	27.11148	27.00838	26.88577
26.77706	26.75175	26.51051	26.06085	26.33586	26.37285	26.81421	26.20769	26.78955
31.91171	31.99833	31.86821	31.63288	31.65438	31.61544	NaN	NaN	21.68003
28.50522	28.72923	28.52776	28.55728	28.53148	28.50142	28.47073	28.41135	28.67303
26.14686	26.63201	26.48001	27.4073	27.5389	27.57542	27.35783	27.56819	27.26249
30.70471	30.55728	30.67976	31.08005	31.13614	31.18423	31.02168	31.02828	30.80039
28.52028	28.78955	28.57903	28.54259	28.37451	28.391	28.39917	28.35363	28.45513
27.2803	26.76446	27.34096	27.90311	28.20769	28.08639	28.53148	28.42342	28.50142
27.21697	27.44727	26.98685	27.65949	27.67976	27.82031	26.75175	26.82639	27.06085
26.86231	26.68646	25.82153	27.07112	27.34096	27.22619	27.29789	26.98685	27.509
27.42342	27.391	27.07112	27.08132	27.10149	26.95394	27.58262	27.68646	27.53148
27.73247	27.79576	27.63201	27.78955	27.95947	27.74535	28.29351	28.05569	28.06085
30.02168	29.77549	30.07368	30.4919	30.36202	30.51089	30.81574	30.79266	30.89735
28.04009	28.22159	28.04009	27.75175	27.80809	27.67976	26.89735	26.85043	26.68646
29.35993	29.36202	29.22389	29.19601	29.14589	29.18187	29.43738	29.4512	29.58799
25.05258	25.38113	24.47383	26.03591	26.24627	26.05051	27.90885	27.69312	27.98685
28.53519	28.5969	28.21697	28.74855	28.76762	28.74535	28.73247	28.72923	28.74535
28.42742	28.55728	28.37036	28.55362	28.61807	28.70636	28.10649	28.22619	27.99226
28.3662	28.77077	28.41538	28.67303	28.62854	28.66966	27.88577	28.09144	27.58978
29.07368	28.32817	28.88285	29.63201	29.57181	29.67303	29.00838	28.71293	28.96774
28.33671	28.31527	27.99226	28.47073	28.61105	28.82941	29.61807	29.66628	29.49572
30.04791	30.12386	29.96774	30.19601	30.17236	30.18423	32.23934	32.2106	32.2135
NaN	NaN	23.72858	NaN	NaN	NaN	29.23306	28.97596	29.13614
28.43539	28.72923	28.4073	28.30661	28.4073	28.391	28.45905	28.63547	28.46295
27.76446	28.21234	27.80809	28.391	28.5389	28.27586	28.258	28.23535	28.35363
29.10149	29.19131	29.15074	28.84745	28.83545	28.81421	28.29351	28.22159	28.13614
28.32817	28.89157	28.45905	28.36202	28.91171	28.57181	26.65949	25.59263	26.68646
26.65949	27.06085	26.61807	26.39754	26.33074	26.28912	27.57542	27.32389	27.51653
27.07112	26.54333	26.90885	27.2803	27.35783	27.16997	27.16039	26.96499	27.01903
26.42502	26.54038	26.55801	26.92025	26.77706	26.82639	27.58978	27.57542	27.44727
27.33245	27.56092	27.46295	27.49381	27.55362	27.41538	27.76446	27.84446	27.58978
26.26428	26.33415	26.07521	26.08942	25.47692	25.89041	25.93607	25.95172	26.07112
29.00571	28.79266	28.80193	28.79885	28.76446	28.74855	28.80501	28.95394	28.72923
27.26249	27.58262	27.35783	26.97596	27.13124	27.12139	27.05051	26.75175	27.16997
30.65438	30.78488	30.59335	30.93298	30.90455	31.03486	30.86083	30.80809	30.82335
30.81574	31.0414	31.00169	30.74535	30.70471	30.83092	31.02168	30.96774	31.13001
28.74855	28.93157	28.83244	29.60222	29.53148	29.52962	28.95394	28.93157	29.05827
30.12386	30.19601	29.98142	30.14831	30.24218	30.12386	30.36202	30.46295	30.41337
29.13124	29.04009	29.10649	29.80039	29.77392	29.89735	30.19601	30.08639	30.03486
27.48616	27.61105	27.53148	27.32389	27.34096	27.14102	26.06907	26.10749	25.97378
29.27142	29.37244	29.24445	29.104	29.13369	29.01372	29.2803	29.20303	29.14346
29.14589	29.23535	29.34731	28.80809	28.67976	28.89446	29.17712	29.13614	28.87115
29.8814	30.19601	30.14831	30.07368	29.96774	30.03486	31.80039	31.80809	31.80039
28.64924	28.63547	28.76762	28.77077	28.79576	28.53519	28.34942	28.16997	28.37451
28.76129	28.56819	28.78644	28.48999	28.49762	28.4746	27.92592	27.85043	28.09144
27.00838	27.1795	26.90885	26.87408	26.92025	26.89735	28.52028	28.68979	28.42742
29.8279	30.00838	29.80347	29.31527	29.45905	29.29351	27.97049	28.14589	28.3662
28.07623	28.10649	27.80809	28.15074	28.16997	28.04531	27.73247	27.72598	27.75812
28.01903	28.2535	28.10149	27.90885	27.92025	28.09144	28.69312	28.58262	28.71947

29.32389	29.50522	29.26024	29.30226	29.22159	29.15557	30.07368	30.08639	29.99496
29.65438	29.87408	29.73085	29.74053	29.77077	29.7357	28.56456	28.258	28.49381
28.18423	28.13614	28.08132	27.93157	27.83846	28.01372	28.50522	28.45905	28.57181
29.11892	29.2535	29.22389	29.41538	29.4334	29.37451	29.55179	29.44332	29.52589
28.24445	28.35783	28.3452	28.33671	28.26696	28.19366	27.97596	27.96499	28.14102
31.16639	30.76129	31.08005	31.35678	31.54352	31.39305	31.17236	31.28692	31.40832
30.55728	30.6372	30.5389	30.95394	30.89012	30.96774	30.68813	30.6372	30.67135
35.27239	35.20478	35.2931	34.38921	34.60885	34.48832	33.49429	33.51794	33.50616
31.83846	31.94	31.69229	31.51089	31.58441	31.44332	30.9746	30.95394	30.78488
27.67303	27.82031	27.54628	27.68646	27.56819	27.64581	28.47846	28.21697	28.56092
28.37451	28.15074	28.48616	28.53148	28.49381	28.45905	28.11644	28.13124	28.03486
27.1795	26.52999	26.93157	26.90885	27.10149	27.12139	27.95947	27.71947	27.91456
28.24445	28.49381	28.20303	27.80809	28.02433	28.0296	26.83846	26.86231	26.83846
29.69146	29.92592	29.80809	29.49953	29.50142	29.29351	29.62506	29.63547	29.6441
28.2803	28.54259	28.13124	28.19366	28.17474	28.2535	28.05051	28.11148	28.06599
29.98142	29.84296	29.96774	29.66458	29.65608	29.61456	29.52589	29.67808	29.67303
27.07112	27.33245	26.98685	26.51352	26.40568	26.55508	27.13124	26.42021	27.08132
27.68646	27.75175	27.71293	28.17474	28.14589	28.24898	28.4073	28.3452	28.45513
27.28912	27.55362	27.18896	27.35783	27.31527	27.2803	27.56092	27.34942	27.44727
28.05569	28.20769	27.94838	28.64924	28.59335	28.70636	28.44727	28.43539	28.51653
27.45513	27.49381	27.2535	27.94838	27.93719	28.08639	27.89735	27.85638	27.80193
29.4919	29.55362	29.48039	29.41135	29.3452	29.41941	29.72598	29.57542	29.56819
27.97049	27.94838	27.98685	27.77077	27.91456	28.09144	28.3869	28.23078	28.40324
27.44727	27.98685	27.54628	27.67976	27.77077	27.71293	26.92025	26.90885	26.61807
28.60045	28.70636	28.55362	28.74855	28.60045	28.77392	28.62854	28.80501	28.68979
27.09144	27.33245	27.16997	26.77706	27.01903	27.06085	26.2535	26.30313	26.04843
26.61807	26.75175	26.44253	26.55508	26.22619	26.01903	26.82639	26.85043	26.57542
27.83244	28.06085	27.97596	27.85043	27.80193	27.65949	28.20769	28.1795	28.21234
27.61105	27.82639	27.56092	27.89157	27.70636	27.89735	28.1795	28.07112	28.04531
27.05051	27.1795	27.04009	27.391	27.43141	27.42342	24.71293	25.09748	24.73376
28.34096	28.34096	28.34942	27.62506	27.54628	27.62506	27.05051	26.95394	27.00838
27.43141	27.43141	27.3662	27.55362	27.57542	27.63893	27.97596	28.32389	28.00838
29.14831	29.46879	29.22849	29.44924	29.63027	29.4649	29.37865	29.56819	29.4649
23.70767	24.39754	23.82881	25.48922	25.50142	25.41377	26.52103	26.57109	26.77706
24.47383	24.63755	23.78955	25.53445	25.60116	23.80931	26.97596	27.55362	26.80193
29.29789	29.35783	29.49572	29.20303	29.30226	29.42342	28.68311	28.509	28.63893
26.80193	26.96499	26.95394	27.2803	27.53148	27.33245	26.92025	27.18896	26.82639
NaN	NaN	NaN	NaN	NaN	NaN	26.82639	26.68646	26.69975
27.67303	28.4073	28.20303	27.58978	27.16997	27.07112	25.73892	25.1332	24.84805
28.13614	28.24898	28.01903	27.1795	27.31527	27.35783	28.04009	27.79576	27.95947
29.104	29.2007	29.0296	29.53148	29.55545	29.60929	29.69644	29.67303	29.79885
28.37036	28.37865	28.33245	27.87408	27.89157	27.94838	28.20303	28.01372	28.37036
30.12386	30.16039	30.08639	30.11148	30.20769	30.19601	30.81574	30.73731	30.76129
NaN	22.97771	24.04427	22.24771	22.58233	20.48738	29.28912	30.65438	29.24898
28.31959	28.43539	28.44727	28.47846	28.51277	28.31527	26.67303	27.12139	27.2535
29.99496	30.12386	30.04791	29.94	29.87408	29.84745	30.02168	29.99496	29.99496
30.51089	30.67135	30.57542	30.84595	30.80809	30.90455	30.47267	30.39305	30.42342
27.60399	27.58262	27.45513	27.80193	27.95394	27.90885	28.4512	28.24445	28.2535
28.4512	28.79266	28.34096	29.09648	29.07368	29.11644	28.13124	28.0296	27.95947
30.96086	31.19013	31.11768	30.92592	30.91171	30.94698	29.12386	29.03223	29.16279
28.45513	28.80809	28.39509	28.47073	28.57542	28.52402	29.04791	29.11644	28.80809
29.7973	29.95394	29.79421	29.41941	29.26024	29.30443	29.58978	29.41337	29.60576
27.54628	27.80193	27.68646	25.4008	25.67573	25.84086	21.50688	23.48001	21.66709
29.35363	29.43141	29.23078	29.44332	29.39305	29.4174	28.12633	27.98685	28.16519
27.70636	27.56092	27.50142	27.61807	27.67303	27.94838	28.41538	28.44332	28.33245
28.95671	29.18896	28.97049	28.45905	28.48616	28.56819	28.88867	29.13614	28.85638
29.7597	29.92592	29.92592	29.88577	29.98142	30.03486	30.03486	30.11148	30.18423
30.42342	30.67135	30.59335	30.60222	30.61105	30.55728	31.08639	31.02168	31.03486
30.86083	30.55728	30.59335	29.72923	29.23078	29.78176	24.24627	26.57542	24.98033
26.2553	26.20583	25.60399	26.31527	25.6513	25.55215	26.80193	26.50446	26.42502
28.60399	28.06599	28.24898	28.50522	28.61105	28.66628	28.54995	28.391	28.84146
NaN	NaN	NaN	23.35111	NaN	NaN	28.51277	28.5969	27.70636
29.61456	29.34096	29.63893	30.16039	30.23078	30.21928	29.66797	29.75015	30.02168
29.94	30.11148	29.94	29.59513	29.52402	29.60752	32.30334	32.34361	32.21928
28.01372	28.01372	27.70636	27.98142	27.99766	28.05569	27.46295	27.45513	27.22619
27.13124	27.22619	27.06085	27.57542	27.63201	27.62506	26.9428	26.60399	27.04009
28.92309	29.03486	28.76446	27.391	27.39917	27.33245	28.15557	28.28472	28.03486
26.72598	27.18896	26.77706	27.3662	26.68646	26.64581	27.11148	27.31527	27.19835
31.42842	31.67135	31.17236	31.70471	31.58889	31.6372	31.46295	31.46295	31.45317
30.00838	30.19601	30.20769	29.71457	29.68646	29.83846	29.99496	29.83244	29.78176
27.42342	26.52552	27.26249	27.75175	27.57542	27.69312	29.37865	29.40324	29.19366
27.64581	27.45513	27.15074	27.53148	27.46295	27.58262	28.01903	28.06599	27.97596
29.54995	29.79885	29.58082	29.04791	29.13858	29.07112	29.28251	29.18896	28.98142
27.47073	27.509	27.34942	27.3662	27.64581	27.35783	28.12139	28.01903	28.04009
27.97596	28.07623	27.88577	27.87408	27.96499	27.92592	28.34096	28.15557	28.07112
29.10899	29.13124	28.95394	28.88577	28.98956	28.85935	28.5389	28.39509	28.44332
29.31527	29.35363	29.36411	28.94	28.90885	28.96774	28.86821	28.99496	28.68979
28.0296	28.34096	27.84446	28.57903	28.55362	28.62854	27.99766	27.72598	27.84446
29.41538	29.60929	29.52589	29.4649	29.46295	29.46684	29.4453	29.5591	29.3869
25.62923	25.36786	24.83846	25.55508	25.62366	25.61526	26.28912	26.93157	26.1776
26.51352	26.61807	26.40568	26.18329	26.26607	26.3662	27.01903	26.60399	26.77706
28.43539	28.57903	28.65267	28.54259	28.57181	28.64924	28.22619	28.391	28.15557
27.62506	27.47846	27.73247	27.80809	27.14102	27.27142	25.38443	22.45262	24.85281
30.00838	30.26472	30.18423	30.02168	29.88577	29.8549	30.02168	30.12386	30.08639
27.91456	28.20769	27.97596	27.51653	27.72598	27.96499	24.03591	23.79947	23.85757
28.59335	28.7162	28.68311	28.85043	28.77077	28.97596	28.91741	28.86821	28.90311
29.01105	29.21234	29.17236	29.4453	29.49381	29.4919	29.34942	29.2535	29.3452
28.99496	28.61807	28.98956	27.54628	28.00838	27.77706	23.70767	23.63201	23.45513

31.0927	31.0414	31.50142	30.91171	30.88285	30.96774	32.31149	32.44825	32.36984
33.00838	33.05924	33.15285	32.66713	32.74535	32.70058	33.74535	33.70471	33.65224
32.5038	32.48472	32.42342	31.89735	32.08004	31.93649	32.16339	32.29789	32.09585
28.02433	28.0296	28.04009	27.95947	27.84446	28.08639	28.30661	28.3662	28.4512
30.23078	30.46295	30.35153	30.40324	30.39305	30.35153	30.4334	30.36202	30.41337
27.2803	27.2535	27.00838	27.47073	27.52402	27.83244	26.61807	26.58978	26.13712
22.61414	23.68646	21.57773	21.73066	22.48493	22.35917	28.44332	28.47846	28.80501
28.56819	28.62157	28.35363	28.60752	28.76762	28.66628	28.15557	28.16997	28.10649
30.52962	30.5389	30.44332	30.26472	30.20769	30.19601	30.00838	29.89735	29.99496
29.0296	29.16279	29.12633	29.15074	28.93438	28.87994	29.67472	29.7211	29.708
32.71702	31.28692	32.45562	32.86636	32.68186	33.03321	31.74535	31.60222	31.60222
28.30661	28.68646	28.54259	28.16519	28.24445	27.97049	23.86703	24.09346	24.15653
27.13124	27.18896	27.20769	27.68646	27.70636	27.56092	27.73892	27.53148	27.60399
28.85341	28.81115	28.79885	28.5862	28.509	28.50522	26.98685	26.88577	27.04009
26.76446	26.96499	27.24445	27.27142	27.77706	27.43141	28.07623	28.35783	27.99226
27.51653	27.96499	27.9428	27.41538	27.0296	26.85043	24.1332	25.61807	24.30313
28.12139	28.2535	28.258	27.87994	27.94838	28.13614	28.16519	27.95394	28.24898
NaN	23.391	NaN	24.42342	22.11108	23.37782	26.82639	27.15074	26.95394
28.06085	25.07316	28.10649	28.65949	28.01372	28.29351	28.97323	28.57903	29.09144
26.13907	25.99766	26.06085	25.9157	26.36453	24.72858	26.76446	27.3662	27.06085
27.38278	27.391	27.09144	27.95947	27.95394	27.90885	28.11148	27.97596	28.14589
25.96719	25.88577	25.56092	26.18139	25.73892	25.49838	26.24989	26.85043	26.43936
27.53148	27.70636	27.391	27.97049	28.04531	27.97049	27.16997	27.31527	27.43936
27.80193	27.74535	27.58978	27.63893	27.70636	27.76446	27.96499	27.78332	27.73892
28.4073	28.48616	28.32389	28.5389	28.65267	28.56456	28.3662	28.33671	28.37036
27.00838	27.27142	27.33245	26.76446	27.37451	26.80193	27.38278	27.26249	27.43141
29.49953	29.30443	29.31527	29.85341	29.91171	29.91171	28.78955	28.98142	28.65949
27.67303	28.04531	27.73247	27.95394	27.82031	27.75812	28.41538	28.39509	28.52028
28.61105	28.63547	28.80193	29.0296	28.85935	28.91456	28.82335	28.72598	28.84446
26.31354	26.42982	26.32389	27.13124	27.16039	27.63201	28.24898	28.26696	28.31095
27.47073	27.75812	27.01903	27.92592	28.10649	27.93157	27.93157	28.04531	27.92592
26.87408	26.83846	26.44884	27.07112	26.9428	26.98685	27.27142	27.37451	27.26249
30.71293	30.52962	30.64581	30.62854	30.61105	30.64581	28.35783	28.45513	28.63201
30.60222	30.5389	30.70471	30.67135	30.60222	30.52962	30.12386	30.29789	30.11148
27.63201	27.73247	27.62506	27.95394	27.80193	27.73247	26.75175	26.82639	26.81421
27.48616	27.35783	27.4073	27.92025	28.03486	27.90311	27.92025	27.85638	28.0296
28.76762	28.84146	28.84146	28.18423	28.10149	28.20303	29.05051	28.94559	29.05827
29.22389	29.34096	29.3452	29.24445	29.28912	29.22849	29.31311	29.21466	29.19835
26.47228	27.0296	26.55069	26.89735	26.89735	26.90885	27.69312	27.24445	27.28912
26.64581	27.05051	26.87408	25.75175	25.73376	25.92025	25.48922	25.28912	26.18896
28.43936	28.47073	28.19366	28.08639	28.27586	28.63547	25.81665	26.12139	25.21697
26.85043	27.00838	26.60399	27.00838	26.80193	26.87408	25.65676	25.97596	26.13516
26.45199	27.00838	26.61807	26.98685	26.92025	27.12139	25.58405	26.43778	26.0296
28.57903	28.7357	28.68646	28.75494	28.57542	28.63547	29.33031	29.07623	29.4334
27.76446	28.07112	28.06085	27.23535	27.30661	27.61105	22.40406	22.86703	24.2535
26.4186	26.55069	25.77957	26.17188	26.239	26.27853	25.38772	25.65949	25.65949
26.09949	26.40406	26.63201	26.77706	26.78955	26.68646	26.85043	26.68646	26.85043
26.64581	26.87408	26.72598	26.39917	26.4008	26.55508	26.41538	26.46607	26.33245
26.31008	26.63201	26.43301	26.43778	26.50142	26.24264	26.78955	26.80193	26.64581
31.17236	31.27031	31.2814	31.60664	31.50616	31.48232	31.17831	31.09899	31.31419
26.93157	27.18896	26.90885	26.95394	26.72598	26.82639	27.43936	27.24445	27.391
27.13124	26.73892	27.16997	27.13124	26.93157	26.88577	27.10149	26.88577	26.99766
27.67976	28.01903	27.80809	27.66628	27.509	27.35783	26.86231	27.13124	27.01903
32.9574	32.96086	33.16788	32.92415	33.03485	33.08955	30.4919	30.40324	30.33031
26.64581	26.43619	26.47846	26.36453	26.03171	26.08335	25.62366	25.30313	25.40406
26.82639	26.9428	26.89735	26.55655	26.61807	26.82639	27.44727	27.43141	27.42342
28.50142	29.11644	28.90311	28.86821	28.85341	29.02433	29.26249	29.45905	28.82335
29.31743	29.43141	29.24218	29.3869	29.42142	29.53519	29.10149	28.85638	29.01638
30.88285	30.85341	30.80809	30.74535	30.68813	30.92592	29.75175	29.99496	29.99496
30.60222	30.72923	30.66289	30.37244	30.37244	30.30878	29.74214	29.51277	29.63374
28.13614	28.59335	28.09144	27.95947	27.85638	27.64581	26.72598	26.68646	26.78955
27.71293	27.97049	27.63201	27.54628	27.66628	27.5389	27.84446	27.89157	28.15074
28.70965	29.04791	28.72598	28.62157	28.64238	28.63893	29.00838	28.80193	28.92592
28.52028	28.45513	28.48232	28.78644	29.0296	28.85341	28.3869	28.26696	28.41941
28.4746	28.7357	28.48616	28.79576	28.69312	28.72273	29.23078	29.14346	29.18423
29.55728	29.87555	29.52776	29.94	30.03486	30.03486	29.64238	29.46295	29.54628
28.81726	29.14589	28.83545	29.24898	29.07112	29.03748	28.05051	27.93157	27.96499
31.27031	31.38792	31.16639	30.70471	30.70471	30.74535	30.30878	30.41337	30.21928
30.21928	30.06085	30.20769	30.64581	30.62854	30.56638	30.39305	30.08639	30.28692
30.54812	30.4334	30.61982	30.79266	30.82335	31.02168	30.66289	30.89012	30.52028
29.76762	29.33671	29.77077	30.13614	30.18423	30.31959	29.87848	29.87262	29.75494
30.35153	29.67976	30.37244	30.00838	29.94	30.11148	29.73247	29.95394	29.7692
30.90455	30.89012	30.88285	30.9746	30.94	30.96774	30.06085	30.03486	30.04791
28.68646	28.88577	28.72923	28.37451	28.21697	28.28472	28.76129	28.6764	28.7357
26.39754	26.33756	25.91114	25.75685	25.51953	25.2535	26.0041	26.77706	26.15074
27.20769	27.38278	27.28912	27.09144	27.07112	26.97596	26.85043	26.64581	26.78955
27.45513	27.69312	27.5389	27.57542	27.65949	27.98142	26.35111	26.98685	26.54333
29.99496	29.98142	30.16039	29.64065	29.6981	29.71947	29.57903	29.65267	29.77706
24.79947	25.53445	23.96719	25.7024	26.01903	25.88344	27.09144	27.19835	27.16039
25.56092	26.04009	25.09346	26.01478	25.62087	25.73376	26.0874	26.16615	26.27853
30.08639	30.26472	30.34096	29.98142	29.94	29.88431	29.62854	29.4512	29.57903
30.04791	30.03486	30.26472	30.16039	30.36202	30.35153	30.68813	30.52028	30.47267
27.30661	27.43141	27.11148	26.85043	26.99766	27.11148	27.27142	26.98685	26.99766
27.70636	27.49381	27.78955	27.71293	27.83244	28.06599	27.91456	28.14589	28.07623
30.29789	29.7973	30.36202	30.52962	30.52962	30.46295	29.06599	28.92025	29.19835
28.18423	28.07623	28.00303	28.19835	28.17474	28.07623	28.37036	28.2803	28.34096
26.34773	26.86231	26.32903	26.71293	26.56819	26.72598	26.55947	26.60399	26.38772
26.24264	27.19835	26.36786	26.96499	27.04009	26.60399	26.88577	27.15074	26.64581

26.01478	26.65949	26.01265	26.38772	26.72598	26.64581	27.80809	27.73892	27.81421
28.77077	28.53519	28.50522	28.37451	28.44332	28.54995	28.5389	28.81115	28.45513
26.92025	27.10149	26.9428	26.90885	26.98685	27.00838	26.49228	26.78955	26.69975
27.19835	27.3662	27.24445	27.42342	27.61807	27.53148	26.82639	26.83846	26.9428
27.05051	27.34942	27.34942	27.39917	27.47846	27.45513	28.06085	28.14589	28.05569
27.48616	27.5969	27.64581	27.22619	27.47846	27.27142	27.16039	27.5389	27.10149
28.5969	29.19835	28.87408	28.65267	28.48616	28.60045	27.65949	27.63893	27.90311
28.81726	29.06343	28.87994	28.82941	28.42342	28.63201	26.77706	26.9428	25.9428
30.09899	29.96774	30.08639	30.29789	30.35153	30.52028	29.66966	29.88867	29.55545
26.1488	25.82153	26.11347	26.34604	26.34266	26.38936	26.60399	26.50142	26.21512
30.35153	30.35153	30.40324	30.03486	30.03486	29.87848	30.7211	30.82335	30.61982
28.50142	28.68311	28.52028	28.20769	28.16039	28.02433	26.4346	26.20769	26.23169
26.03171	25.95394	25.83123	25.82639	26.14686	25.38443	27.05051	27.81421	27.37451
30.18423	30.11148	30.2535	30.42342	30.38278	30.52028	30.34096	30.21928	30.31959
28.15557	28.30226	28.05569	27.75175	27.56819	27.56819	28.15557	28.07623	28.18896
26.37782	26.86231	26.56092	27.55362	27.74535	27.61807	28.52028	28.4073	28.52776
27.15074	27.71293	27.32389	27.67976	27.63893	27.75812	28.391	28.40324	28.4512
29.91171	30.06085	29.83092	29.8549	29.99496	30.03486	28.66966	28.78332	28.68646
25.94056	26.06291	27.11148	26.85043	27.30661	28.06599	23.71816	23.88577	22.82104
27.1795	27.05051	27.0296	27.13124	26.64581	27.12139	26.35783	27.01903	26.16615
27.3662	27.75812	27.48616	27.60399	27.66628	27.5969	27.42342	27.70636	27.29789
27.66628	28.24898	27.76446	27.49381	27.33245	27.65949	27.76446	27.71947	27.65267
26.13712	26.27675	26.42982	26.13907	26.21697	26.14492	28.12139	28.07623	27.96499
26.33415	26.57109	26.18518	26.12928	26.35616	26.36119	NaN	NaN	NaN
26.391	26.9428	26.09346	27.00838	26.98685	26.53297	24.98902	24.77455	25.86703
26.80193	26.96499	27.27142	26.76446	26.95394	26.68646	26.81421	26.63201	26.64581
26.18139	26.72598	27.08132	25.80931	26.32732	26.46451	24.8811	25.24264	24.90426
28.45905	28.54259	28.5389	28.43936	28.33245	28.41135	29.21234	29.06343	29.15557
26.72598	26.75175	26.71293	27.16039	27.05051	27.37451	26.56529	26.54038	26.83846
25.47692	26.09748	25.4073	26.49228	26.60399	27.0296	24.10149	25.02749	24.21697
27.66628	27.89735	27.70636	27.32389	27.32389	27.2803	27.00838	27.3662	26.90885
26.63201	27.0296	26.93157	26.92025	26.75175	26.77706	27.77706	28.43141	27.90885
27.12139	27.43141	27.15074	27.30661	27.45513	27.48616	27.95947	27.86821	28.00303
29.29351	28.85935	29.60752	28.84745	29.05827	29.27142	22.84613	23.42982	24.79452
30.45317	30.4919	30.55728	30.42342	30.4919	30.55728	30.36202	30.21928	30.31959
27.1795	27.52402	27.0296	26.87408	26.78955	26.99766	27.34942	27.31527	27.45513
29.91171	30.08639	29.91171	30.2535	30.27586	30.37244	29.89446	29.82941	29.92592
25.30661	26.03171	25.84086	25.56674	25.61526	26.1035	26.26785	26.75175	26.55947
27.22619	27.2535	26.90885	27.08132	27.16997	27.14102	27.18896	27.56092	27.16997
28.58978	28.85043	28.86821	28.96499	28.82639	28.95947	29.37451	29.33671	29.28912
NaN	NaN	NaN	NaN	NaN	24.28912	29.5969	28.60752	29.56275
30.17236	30.16039	30.23078	30.61105	30.52962	30.76129	30.28692	30.06085	30.17236
29.13614	29.23763	29.27808	28.66289	28.51653	28.86526	26.27853	26.32732	25.85519
27.21697	27.23535	27.31527	27.19835	27.23535	27.1795	NaN	NaN	NaN
29.64238	29.80193	29.68646	29.41135	29.31095	29.33458	27.84446	27.98142	27.94838
29.81574	29.70471	29.78176	30.06085	29.95394	30.21928	29.7692	29.73731	29.75653
29.92592	29.77863	29.83092	29.96774	30.06085	30.13614	30.14831	30.08639	30.14831
28.58262	28.62854	28.58262	28.41941	28.4746	28.4073	29.14831	29.02433	29.22849
25.77706	26.16231	25.70504	26.09949	26.36453	26.2021	25.9428	25.86703	26.02538
26.63201	27.15074	26.81421	26.87408	26.83846	26.58978	27.2803	27.39917	27.18896
27.43141	24.6971	27.53148	23.62087	25.56964	27.75812	26.05258	26.73892	25.95394
26.89735	26.71293	26.73892	26.55069	26.4186	26.28383	27.509	27.00838	27.16039
27.07112	27.0296	27.28912	27.06085	27.2803	27.20769	27.09144	27.24445	27.08132
30.19601	30.33031	30.36202	31.00169	31.0544	31.0927	30.4919	30.47267	30.51089
26.58978	26.38113	26.65949	26.85043	26.75175	27.07112	27.07112	26.99766	26.96499
27.51653	26.73892	27.16039	27.61105	28.14102	26.90885	26.82639	26.54038	26.24989
28.28472	28.2535	28.32817	28.39917	28.13124	28.258	28.07623	27.95947	27.90885
29.88577	29.98142	30.09899	30.03486	30.19601	29.94	30.7211	30.7692	30.73731
29.61281	29.4746	29.64924	29.70965	29.76604	29.75175	29.96774	30.02168	29.95394
25.28912	26.04635	26.09346	26.2553	25.94726	26.29964	26.9428	26.77706	27.06085
26.22803	25.77706	25.7543	25.72338	25.14492	24.98902	25.94949	26.22435	26.1488
28.79576	28.58262	28.71293	28.61807	29.00838	28.97323	26.9428	27.00838	27.04009
27.19835	26.96499	27.16997	27.24445	27.47073	27.57542	27.509	27.52402	27.29789
28.29351	28.42742	28.35783	28.70636	28.67976	28.61456	28.31095	28.31527	28.26696
27.12139	27.09144	26.96499	27.09144	27.05051	26.89735	27.47073	27.38278	27.27142
29.75334	29.80655	29.95394	29.76446	29.61632	29.66458	28.74214	29.13369	28.78019
29.11644	29.35363	29.29132	29.71947	29.75812	29.80347	29.59335	29.53334	29.31959
25.51051	24.02749	25.87877	25.73376	26.09346	25.92025	26.55215	26.86231	26.93157
21.40145	21.3538	21.25926	21.29894	20.9812	23.25061	28.42342	28.4073	28.35783
25.94056	25.97596	26.16039	25.88809	25.73376	25.80931	26.00838	25.32732	25.71293
28.18423	28.42742	28.31959	27.99226	28.2803	28.12139	28.14589	28.22619	28.26696
27.47846	27.83846	27.30661	27.63201	27.77077	27.81421	26.82639	26.86231	26.11148
26.23535	26.46762	26.30138	26.80193	26.76446	26.57542	26.51953	26.19835	25.69975
28.37036	28.70306	28.43539	28.42742	28.48232	28.55362	27.28912	27.16997	26.80193
26.92025	26.82639	27.04009	27.29789	27.42342	27.35783	27.70636	27.61105	27.75175
28.31095	28.34096	28.11148	28.76129	29.00303	28.87408	28.23078	28.35783	27.90311
23.29614	22.26499	24.47383	25.50749	25.26069	25.62645	26.83846	26.72598	26.75175
27.67303	27.98685	27.89735	27.50142	27.30661	27.3662	27.16039	27.12139	27.11148
28.01372	28.18423	27.86231	28.28912	28.16997	28.19835	27.77706	27.47846	27.49381
27.34942	27.45513	27.15074	27.2535	27.3662	27.34942	27.89157	27.93157	27.99766
29.08385	29.2535	29.22159	29.04791	29.02697	28.91741	29.07877	28.89735	29.20536
29.29351	29.34096	29.2535	29.61807	29.55728	29.53148	28.83545	28.66628	28.98685
29.24672	29.33671	29.4174	29.14831	29.24898	29.24672	29.62157	29.52776	29.509
28.51277	28.2535	28.41538	28.23078	28.39917	28.41538	28.4746	28.71293	28.57542
28.32389	28.57181	28.47073	28.81421	28.509	28.56819	29.30661	29.20536	29.30661
26.89735	26.76446	26.87408	26.88577	26.88577	26.85043	26.99766	26.67303	26.88577
28.05051	28.12139	28.08639	27.82639	27.97049	27.99226	28.44727	28.41538	28.43936
27.91456	27.78332	27.76446	27.43141	27.39917	27.29789	27.63201	27.37451	27.63201

29.70306	29.85638	29.5862	28.52402	28.68311	28.60752	28.55362	28.5969	28.5389
26.58978	26.26785	26.33586	26.56092	26.65949	26.57542	24.45513	24.59833	NaN
27.28912	27.39917	27.29789	27.23535	27.21697	27.16039	27.0296	27.05051	27.21697
26.4186	26.77706	26.317	26.239	26.16231	26.25169	27.00838	27.05051	26.67303
25.59263	25.98251	25.66763	25.81176	25.65403	25.84805	26.29964	26.1757	26.16039
27.19835	27.20769	27.50142	26.82639	27.08132	27.01903	25.54628	25.73892	24.55801
26.64581	26.86231	26.51352	26.34266	26.80193	26.49838	27.73247	28.31527	27.41538
28.94838	29.17712	28.97869	28.82031	28.77706	28.89157	29.02697	28.87408	29.01105
NaN	25.01903	24.93157	24.35111	25.28207	24.59263	25.12139	26.50294	25.87408
26.75175	25.67842	26.9428	27.07112	26.55801	26.61807	27.509	27.16039	26.01903
28.87701	29.05051	28.94838	28.31527	28.23535	28.29351	28.67976	28.57903	28.65608
27.09144	27.19835	26.93157	27.12139	27.23535	27.1795	29.04791	29.09648	29.25575
27.2803	27.35783	27.39917	28.11148	28.07623	28.11644	28.35783	28.16039	28.2535
28.10649	28.24898	28.14102	28.05569	27.99766	28.01903	27.08132	27.00838	27.15074
26.89735	27.10149	26.61807	27.35783	27.5389	27.31527	26.44884	26.61807	26.67303
29.69975	29.53334	29.89735	29.48039	29.35153	29.46879	29.34942	29.2803	29.2007
29.68979	29.68144	29.87262	30.14831	30.19601	30.2535	29.71784	29.6268	29.98142
NaN	NaN	NaN	NaN	NaN	NaN	29.16758	28.95394	29.16997
29.08132	29.44134	29.71947	28.89735	29.01903	28.82639	27.33245	27.3662	27.29789
28.00303	28.12633	28.02433	28.95947	28.92592	29.11148	28.32817	28.4073	28.24898
28.22159	28.61807	28.391	28.28912	28.21234	28.05051	28.84446	28.87994	28.75494
28.53519	28.68979	28.60399	28.4073	28.60045	28.35783	28.82031	28.52776	28.65608
26.48462	26.71293	26.58978	26.35279	26.15267	26.29088	25.30313	25.26428	24.78457
27.89735	27.85638	27.84446	27.94838	27.71947	27.82639	27.39917	27.34942	27.42342
28.95394	28.99766	28.67976	28.86231	28.5389	28.75175	25.31008	25.60682	25.65676
29.87408	29.8814	29.8814	29.65438	29.64238	29.57181	NaN	NaN	21.32663
29.22389	29.08639	29.18423	29.29132	29.21697	29.26919	29.2803	29.33884	28.96224
27.12139	26.99766	26.83846	26.69975	26.56529	26.83846	27.38278	27.47073	27.65267
26.85043	26.57109	26.93157	27.34096	27.61807	27.53148	27.32389	27.31527	27.16039
26.61807	27.06085	26.58978	27.00838	26.90885	26.81421	27.37451	27.29789	27.16039
27.07112	27.19835	26.98685	26.72598	27.23535	26.82639	26.23535	26.71293	26.57254
30.45317	30.45317	30.36202	29.78332	29.73409	29.79266	NaN	NaN	NaN
27.61807	27.80809	27.63893	27.28912	27.23535	27.2803	26.90885	27.32389	26.93157
30.58441	30.58441	30.60222	30.4919	30.38278	30.4919	30.52962	30.35153	30.61105
27.48616	27.27142	27.04009	26.92025	26.68646	26.60399	27.13124	27.71293	27.66628
30.42342	30.42342	30.47267	30.73731	30.7692	30.79266	30.64581	30.27586	30.42342
28.77392	29.00035	28.98414	29.33671	29.26696	29.4073	29.62331	29.39917	29.61281
31.55728	31.57091	31.38278	31.37244	31.44332	31.50616	30.29789	30.28692	30.20769
30.00838	29.39917	30.12386	30.28692	30.12386	30.34096	29.99496	30.08639	30.02168
26.1035	24.54628	26.1035	26.1946	26.27853	25.87643	27.33245	27.47846	27.26249
27.96499	28.2803	27.24445	27.61807	27.45513	27.29789	28.33671	28.66628	28.43539
29.48616	29.31959	29.41538	29.461	29.4512	29.64753	30.28692	30.36202	30.12386
28.01903	28.13614	28.05051	27.86821	27.87408	27.78955	28.16997	27.9428	28.20769
27.58978	27.91456	27.84446	27.67976	27.89735	27.32389	28.14102	28.19366	28.45513
NaN	NaN	NaN	NaN	24.81421	NaN	27.509	26.67303	26.77706
26.9428	26.81421	26.90885	26.22803	26.28207	26.01903	24.10949	23.62087	24.33074
29.81268	29.98142	29.87994	29.46295	29.50142	29.6981	29.37036	29.32174	29.27808
28.20769	28.258	28.12139	27.80193	27.68646	27.80809	26.61807	26.65949	26.56384
27.34942	26.98685	27.5389	26.317	27.26249	26.57109	NaN	25.01052	23.19761
28.54259	28.42742	28.62157	28.06085	28.04009	27.90885	26.65949	26.85043	26.54481
25.89504	25.85519	26.21512	26.52701	26.54922	26.69975	25.84086	25.56674	25.77706
27.06085	26.26428	26.92025	26.9428	26.89735	26.98685	27.73247	28.13124	27.73247
27.07112	27.57542	26.9428	26.78955	26.52103	26.88577	26.48769	26.54185	26.42822
30.19601	30.26472	30.11148	29.83996	29.80655	29.84446	31.08639	30.83092	31.0414
27.14102	27.39917	27.11148	27.74535	27.83846	27.90311	28.35783	28.26696	28.28472
27.89735	28.28472	28.10149	27.80809	27.9428	27.71293	27.07112	26.44727	26.69975
25.36453	25.17188	26.08132	26.44095	26.1332	26.03171	26.86231	26.46139	26.60399
26.61807	26.57542	26.60399	26.69975	26.76446	26.55362	26.53148	26.51051	26.24082
27.51653	27.29789	26.68646	26.00196	26.97596	27.39917	26.89735	26.52103	26.46139
26.67303	27.00838	26.88577	26.52701	26.83846	26.65949	26.50749	26.52253	26.31872
27.80193	27.76446	27.82031	28.07623	27.93719	27.93719	28.1795	28.1795	28.391
27.81421	27.9428	27.87994	27.47846	27.45513	27.29789	27.47846	27.92025	27.38278
26.68646	26.99766	26.48769	26.27142	26.14297	26.26607	26.30835	26.2553	26.31008
28.20303	27.32389	28.06599	27.38278	27.15074	27.74535	25.89735	25.74663	25.90656
27.82031	27.98142	27.80193	27.87408	27.81421	27.83244	27.85043	28.00838	27.71947
27.41538	28.08639	27.43141	27.0296	27.07112	26.67303	27.0296	27.41538	27.0296
28.89735	28.98956	28.95671	28.96774	29.08132	29.18896	28.15074	28.09648	28.24898
28.16997	28.2803	28.11644	28.2803	27.9428	28.09648	26.73892	26.38607	26.71293
27.50142	27.83846	27.57542	27.61807	27.4073	27.56092	27.11148	26.83846	26.87408
26.33245	26.05672	25.60682	26.39917	27.1795	26.77706	25.16423	25.09748	25.04427
NaN	NaN	24.84805	24.15653	NaN	24.48001	25.16806	25.63755	25.34096
27.26249	27.15074	26.85043	27.69975	27.60399	27.51653	26.93157	27.15074	26.96499
33.65224	33.40831	33.7751	33.33031	33.35678	33.43341	33.64151	33.86083	33.81383
27.5969	27.53148	27.68646	28.14589	28.15074	28.20769	28.06085	27.80193	27.66628
31.90813	31.9574	31.99496	31.88285	31.91883	31.92592	32.27586	32.44332	32.19308
26.26964	26.17379	25.96058	26.07112	25.97158	26.26249	27.20769	26.95394	27.23535
25.70767	25.61807	25.32389	25.1795	25.48616	25.45199	25.03591	25.07316	24.08537
22.90242	NaN	24.33074	NaN	NaN	23.391	24.44253	24.74919	24.56384
26.07928	26.61807	26.14686	27.20769	26.89735	26.63201	25.2021	24.01052	25.20583
26.317	26.61807	26.29964	26.43141	26.46451	26.64581	25.89735	26.03381	25.77957
26.21327	26.22803	26.15074	25.56384	26.05051	25.49534	26.4186	26.24082	26.07725
27.39917	27.58262	27.41538	27.1795	27.509	27.3662	26.80193	26.31008	26.64581
27.58262	27.86821	27.77077	27.09144	27.27142	27.15074	27.91456	27.90311	27.89735
24.59263	24.64306	24.84326	26.76446	26.1946	25.90656	25.83605	25.83123	25.93832
25.9955	26.07928	26.2021	24.33074	24.30313	23.51653	26.67303	26.2856	26.19648
NaN	25.317	22.75735	25.89966	25.54333	25.66221	25.33415	26.09346	25.52253
26.17379	25.56092	25.74663	25.82639	25.76192	25.72078	26.83846	26.47383	26.52103
28.94	28.37451	29.0296	28.03486	27.51653	28.14102	24.08537	22.89874	21.83461

26.69975	27.07112	26.77706	27.07112	27.00838	27.10149	27.85638	27.65949	27.83244
28.55362	29.00303	28.86231	28.70636	28.74855	28.81421	28.65949	28.66289	28.53148
24.15653	23.63201	23.91342	25.05258	25.22803	23.94056	26.00624	27.16997	26.64581
25.99335	25.15653	26.29789	25.11347	24.60399	24.7543	26.54922	26.97596	27.01903
25.87173	25.04843	26.1795	26.2553	25.47073	25.54922	25.29614	25.73117	25.4073
29.67135	30.18423	29.72598	29.62506	29.62157	29.60399	29.43141	29.61105	29.68646
28.37036	28.45905	28.3452	28.31959	28.37036	28.44332	NaN	NaN	NaN
27.4073	27.05051	27.16039	27.16997	27.13124	27.44727	25.73892	25.57254	25.81421
22.37518	22.95482	24.21697	24.01052	23.42982	27.83846	27.85043	27.47073	28.05569
28.391	28.6764	28.44332	28.61105	28.67303	28.55362	28.37036	28.46295	28.35783
26.83846	27.21697	26.97596	26.60399	26.5285	26.60399	26.45199	26.28912	26.29789
26.60399	26.33586	26.02538	26.85043	26.85043	26.87408	26.16039	26.04218	25.73376
27.32389	27.1795	27.26249	27.05051	26.85043	26.83846	27.15074	27.00838	26.73892
27.11148	26.56092	27.13124	26.86231	27.00838	26.89735	27.90885	27.93157	27.86231
27.12139	26.55215	27.72598	26.86231	26.89735	26.92025	NaN	23.32389	NaN
NaN	NaN	21.04926	NaN	NaN	NaN	27.47073	28.61105	28.80809
32.73933	32.52729	32.81765	31.86083	32.0414	32.03813	30.86083	30.86821	30.89735
31.81192	31.68813	31.98142	31.73731	31.81192	31.64151	31.25912	31.17236	31.17236
27.19835	27.18896	26.82639	26.77706	26.60399	26.57542	26.02327	25.8044	26.18707
29.64753	29.66119	29.73085	29.58082	29.52402	29.29351	28.55728	28.49381	28.65267
27.2803	27.20769	27.24445	27.49381	27.19835	27.18896	25.49228	25.45199	25.59833
26.23352	25.9157	26.43778	26.72598	26.61807	26.69975	26.96499	26.82639	26.93157
26.95394	27.10149	27.07112	26.58978	26.64581	26.83846	26.98685	26.65949	26.75175
29.62157	29.92592	29.68311	29.7911	29.78332	29.66458	30.27586	30.06085	30.03486
30.51089	30.71293	30.59335	29.58441	29.54995	29.49572	28.7162	28.78644	28.65949
21.43237	24.79452	24.63201	23.12218	24.65949	23.36453	28.51653	27.92592	28.12633
26.86231	27.19835	26.88577	26.63201	26.64581	26.65949	26.98685	27.21697	27.09144
27.5389	27.86231	27.56819	27.46295	27.35783	27.28912	26.23169	26.61807	26.24445
28.52776	28.75494	28.40324	29.22849	29.23991	29.34308	29.60222	29.44727	29.7211
27.2535	27.29789	26.86231	27.44727	27.56819	27.84446	28.20303	28.1795	28.12633
28.87701	28.84146	28.92592	28.88577	28.57903	28.79266	28.22159	28.24898	28.1795
29.15557	29.3662	29.22619	29.4453	29.50142	29.43141	29.87408	29.82335	29.89157
27.83846	27.76446	27.49381	27.66628	27.50142	27.75175	27.65949	27.62506	27.90885
29.15316	29.14102	29.18896	29.02168	28.97869	28.98685	29.14102	29.18896	29.10649
27.76446	27.99226	27.73247	27.34096	27.34096	27.13124	28.01903	27.68646	27.73892
29.67135	29.87555	29.65096	29.99496	29.87262	30.04791	29.77235	29.6981	29.64238
29.80501	29.73409	29.94	30.17236	30.41337	30.37244	29.73409	29.66458	29.61632
27.9428	28.10149	27.98142	27.69312	27.73247	27.82639	28.09144	28.08132	28.14102
28.16519	28.42342	28.48999	28.21234	28.09648	28.1795	27.29789	27.43936	27.22619
27.43141	27.48616	27.16039	27.06085	26.95394	27.20769	26.77706	26.63201	27.06085
29.32603	29.3662	29.31095	29.06343	29.17236	28.99226	28.65608	28.83244	28.68646
25.82881	26.02327	25.69445	25.88577	25.83846	25.46139	26.08537	26.77706	25.98033
25.22435	25.84326	25.21327	25.21327	24.39754	24.14102	24.49838	24.60399	25.33415
26.97596	26.73892	27.00838	26.73892	26.57398	26.98685	27.22619	27.08132	27.15074
27.44727	27.55362	27.87994	27.56819	27.72598	27.89735	25.73635	25.29614	25.44253
30.89735	30.93298	30.72923	30.57542	30.55728	30.61982	29.50711	29.43141	29.64753
24.81909	25.21327	25.06085	24.84326	24.65403	24.95394	26.43936	26.88577	26.3662
27.12139	27.31527	27.0296	27.05051	26.96499	26.97596	26.68646	26.49076	26.99766
26.44411	26.69975	26.85043	26.95394	27.18896	27.19835	26.47846	26.08132	25.15267
27.11148	27.20769	26.88577	26.93157	26.80193	26.71293	27.41538	27.2803	27.30661
25.11744	25.2571	25.11744	25.46139	25.22066	25.32732	27.52402	27.391	27.39917
28.34942	28.5389	28.68311	28.54259	28.69312	28.68646	28.31959	28.50522	28.31095
26.06702	26.30835	26.35951	26.05879	26.35783	26.08942	26.98685	26.61807	27.04009
27.90885	28.12139	27.97596	27.38278	27.47846	27.51653	27.44727	27.58262	27.2535
27.78332	28.15074	27.75812	27.83846	27.73247	27.85043	NaN	NaN	NaN
26.37948	26.2803	26.04009	26.36786	26.98685	26.72598	25.33415	25.82639	25.52552
26.12731	26.09144	25.92931	26.42662	25.99766	26.2535	27.28912	27.16997	27.00838
26.40892	26.30835	26.05879	26.29263	25.93382	26.34096	25.92931	26.19084	25.73892
29.54075	29.78799	29.51465	29.06343	28.75175	28.75494	27.98142	27.88577	27.74535
NaN	NaN	NaN	NaN	NaN	NaN	25.75175	26.24989	26.38443
27.4073	27.61807	27.15074	27.32389	27.38278	27.33245	27.57542	27.49381	27.60399
27.22619	NaN	27.08132	26.41054	26.87408	25.84805	27.60399	27.47073	28.2803
27.72598	27.71947	27.5969	27.01903	27.06085	26.93157	25.75175	25.11744	25.38113
29.3662	29.61632	29.391	30.03486	29.95394	30.02168	29.43141	29.3869	29.6981
27.29789	27.2803	27.44727	27.76446	27.73892	27.80809	27.74535	27.68646	28.05569
27.2535	27.62506	27.27142	26.56674	26.54628	26.71293	24.75939	24.99766	24.28207
28.00303	27.30661	27.87994	28.53148	28.29351	27.69312	NaN	22.2006	NaN
26.92025	27.08132	27.04009	27.24445	27.13124	27.44727	27.63893	27.66628	27.45513
25.32045	26.96499	26.14492	25.99766	26.33415	26.16039	27.30661	26.97596	27.35783
23.2535	24.91798	22.38574	23.94056	25.65403	NaN	26.33245	26.92025	26.71293
27.63201	27.69975	27.48616	27.82639	27.57542	27.50142	27.42342	27.4073	27.54628
26.95394	26.75175	26.78955	26.50749	26.32732	26.29789	23.76951	23.59833	23.64306
26.28383	25.71816	25.56384	25.65676	25.38443	25.65949	26.52999	26.41538	26.4073
27.41538	27.38278	27.18896	28.11644	28.06599	28.07623	27.35783	27.35783	27.23535
27.23535	27.49381	27.06085	26.95394	26.71293	26.80193	27.56092	27.20769	27.2803
27.66628	28.3869	27.99226	27.69312	27.24445	27.77706	28.32389	28.64238	28.19366
25.32732	25.73117	25.24627	25.86231	25.53742	25.54922	25.77706	25.7415	25.45826
29.66797	29.46879	29.68646	29.94	30.12386	30.06085	29.70141	29.6372	29.63201
27.53148	27.58978	27.53148	27.99226	28.04009	28.42342	28.33671	28.38278	28.47846
26.15267	25.86231	25.7024	26.10949	25.92025	25.68913	26.57542	26.48155	26.55801
26.09346	26.1776	25.85043	26.52701	26.67303	26.76446	26.99766	27.0296	26.92025
27.509	27.53148	27.63201	25.7543	25.37782	26.2021	23.09668	21.6584	21.75532
28.46295	28.258	28.49381	29.11644	29.08132	29.258	29.31527	29.28912	29.16279
25.6811	25.89735	25.87408	25.38443	25.83605	NaN	26.60399	26.90885	26.58978
25.65949	25.42662	24.98033	25.71555	26.11148	25.63201	25.98033	25.92479	26.15074
26.16423	26.19084	25.98468	26.2553	26.17379	26.34942	26.67303	26.53742	26.83846
32.59779	32.29789	32.49429	32.28967	30.82335	32.06407	32.25068	32.63287	31.81955
28.56092	28.3869	28.55362	28.33245	28.45513	28.48999	27.27142	27.16039	27.14102

29.26472	29.49953	29.42542	29.26472	29.32174	29.22849	28.27142	28.35363	28.17474
24.35783	23.60964	24.84805	24.60964	NaN	23.59833	27.35783	26.57398	27.53148
28.32817	28.09144	28.29789	28.39509	28.15074	28.70965	28.23991	28.07112	27.85638
27.77077	27.15074	27.9428	26.50749	26.90885	27.16997	24.27498	24.72858	25.53445
28.5969	28.41941	28.66628	28.36202	28.41941	28.28912	28.63893	28.4746	28.57903
29.78019	29.82941	29.83244	29.80193	29.83394	29.92592	29.7162	29.95394	29.89446
29.89157	30.38278	30.24218	30.19601	30.03486	30.09899	28.63547	28.66628	28.82941
26.04427	25.55801	25.36786	26.56238	26.49076	26.44884	26.82639	27.15074	26.40568
35.16414	35.14148	35.08718	34.05924	34.09899	34.15285	33.21928	33.30334	33.24785
25.72078	25.88577	25.9428	25.96719	25.28207	25.62645	25.64031	25.50446	25.65676
27.50142	27.80809	27.56819	28.13124	28.24445	26.93157	25.38443	25.81421	25.49838
24.73376	25.56384	25.09748	25.89041	25.90656	25.71293	25.93382	25.30661	25.71816
28.95116	29.04791	28.99496	29.19131	29.21002	29.33458	29.37865	29.56092	29.39305
26.81421	26.97596	26.61807	26.75175	26.97596	27.05051	27.38278	27.58978	27.37451
26.77706	26.80193	26.49381	27.16997	26.64581	26.86231	26.4186	26.18518	26.23169
26.63201	26.85043	26.38278	26.26785	26.45199	26.75175	25.92931	26.13124	25.22803
27.14102	26.78955	27.2535	27.2535	27.1795	27.3662	26.68646	26.96499	26.67303
26.49534	26.93157	26.71293	26.63201	26.50597	26.58978	25.89504	26.317	26.49076
26.317	26.48922	27.09144	26.28383	25.88344	25.88577	24.85757	24.47383	NaN
25.52253	25.83605	26.21697	26.32903	26.61807	26.63201	25.06085	24.46762	24.49838
29.31095	29.42342	29.26696	28.12139	28.32817	28.2803	27.90885	28.00838	28.00838
27.43936	27.27142	27.20769	27.41538	27.33245	27.43141	28.1795	28.24445	28.18423
26.22803	26.04635	26.37617	26.15653	25.98902	26.13124	25.98468	26.1757	26.12337
26.23717	26.47073	25.91798	26.22803	25.90426	25.92025	26.20396	26.72598	26.28207
25.02327	25.32045	24.09346	24.18707	24.92705	24.72338	25.05672	25.67842	25.60964
23.04427	23.32389	24.61526	24.67034	25.00624	25.05672	27.63893	27.72598	27.43936
29.82941	29.7973	29.83996	30.33031	30.31959	30.29789	30.04791	29.92592	29.95394
32.15738	31.02828	32.20478	31.36202	31.25912	31.28692	29.05051	28.95394	29.12139
28.72923	29.00303	28.93719	29.04531	28.99766	29.04009	28.73247	28.5969	28.52776
25.98468	26.2856	26.3256	26.15267	26.02115	26.10949	28.19835	27.78332	28.41538
24.98033	25.40406	25.29964	25.01052	24.94056	25.02327	25.79203	24.97596	25.5285
28.28912	28.391	28.29789	28.16997	27.93719	28.16997	26.90885	26.73892	26.55655
27.91456	27.22619	27.44727	26.20955	27.06085	26.60399	23.58692	24.36453	NaN
29.18187	29.31959	29.17474	29.12386	29.01903	29.04531	29.16279	29.19835	29.2803
26.12928	NaN	25.22066	25.9955	26.22251	25.51653	27.2535	27.38278	27.56092
31.07368	31.0927	31.00838	31.31419	31.17831	31.27586	31.14224	31.09899	31.14831
NaN	NaN	NaN	24.6971	28.49762	NaN	25.44253	26.20023	25.86938
26.81421	26.95394	26.78955	27.06085	27.23535	27.43936	26.86231	26.239	26.65949
28.85638	29.0296	28.71293	29.12879	29.06856	29.26024	28.69312	28.62506	28.56092
25.56384	26.18707	25.85281	25.96939	25.48308	25.67303	25.9955	26.2856	26.09949
29.2803	29.38278	29.22389	29.68144	29.5862	29.69312	27.16997	27.69312	26.95394
24.6971	23.33756	24.1332	26.44095	26.4567	26.239	26.96499	27.05051	26.96499
26.07521	24.77957	26.60399	25.60964	24.73376	25.45826	27.64581	27.24445	27.12139
26.80193	26.80193	26.87408	26.31008	26.54333	26.60399	27.52402	27.3662	27.42342
25.23169	25.88344	25.86231	26.69975	26.01691	26.34096	24.44884	24.54628	24.99766
28.71947	29.01105	28.45513	28.36202	28.51277	28.52402	26.89735	27.00838	26.63201
29.81879	29.88867	29.92592	30.28692	30.2535	30.29789	30.02168	30.00838	30.11148
28.08132	28.34942	28.09648	28.19366	28.10149	28.12633	28.90885	28.80501	28.87994
26.25169	26.13516	26.46295	26.06907	25.90885	25.92252	26.42342	26.31181	26.87408
28.29789	28.16997	28.11644	28.09648	28.16997	28.16039	28.54259	28.64581	28.65608
23.42982	23.6971	23.391	NaN	22.27355	25.11347	25.61526	25.54333	27.07112
31.39816	31.75334	31.40832	31.43837	31.37244	31.52028	30.7211	31.02168	30.87555
27.49381	27.82031	27.46295	27.55362	27.44727	27.16039	27.13124	26.99766	27.34942
26.58978	27.00838	26.47228	26.67303	26.60399	26.46139	26.42342	26.49838	26.15267
23.92252	24.23169	23.81909	21.18253	21.66275	21.65403	25.98251	26.78955	25.90426
27.63893	27.63201	27.62506	27.11148	27.24445	27.28912	26.9428	26.61807	26.71293
28.33245	28.43141	28.24898	28.62157	28.63547	28.57903	27.05051	28.03486	27.47073
26.72598	26.07316	26.81421	27.04009	27.01903	26.85043	26.87408	27.2803	27.39917
28.81726	28.91741	28.86526	28.62506	28.77706	28.70965	28.64238	28.66289	28.63201
26.86231	27.06085	26.77706	27.08132	26.92025	26.86231	26.60399	26.87408	26.73892
26.55215	26.83846	26.51202	26.75175	26.36786	26.35783	26.73892	27.0296	26.87408
25.74919	25.68913	25.10949	25.85994	25.7024	26.03591	25.85043	26.30487	25.74919
28.64238	28.94	28.70965	28.16519	27.98142	28.23535	26.71293	26.37285	26.73892
35.09899	35.02663	35.17905	34.22647	34.21928	34.38278	33.21928	33.33031	33.13155
27.5969	27.74535	27.69312	27.83846	27.71947	27.83244	27.91456	27.57542	27.93719
26.83846	25.64581	26.49686	26.69975	26.391	26.56384	27.16997	26.90885	27.57542
26.99766	27.22619	27.00838	27.0296	26.80193	26.89735	NaN	NaN	NaN
26.86231	26.87408	26.97596	26.41538	26.31354	26.22803	26.85043	26.61807	26.83846
25.04009	25.1946	25.36786	26.50446	25.97378	26.38278	25.88344	25.65949	26.00196
27.10149	27.07112	26.80193	27.06085	27.04009	26.96499	27.34096	27.5389	27.68646
27.2803	27.10149	27.18896	26.81421	26.97596	26.9428	26.56092	26.40892	26.63201
29.30878	28.92592	29.06599	28.71947	28.78332	28.9428	26.72598	27.82031	27.19835
26.86231	27.3662	27.1795	27.43141	27.61105	27.509	22.58003	24.42982	24.417
25.82396	26.57398	26.02327	26.22066	26.58978	26.78955	27.27142	27.19835	27.20769
26.65949	27.19835	26.49686	27.61807	26.58978	27.15074	26.31354	25.75175	25.8811
27.00838	26.97596	27.15074	26.86231	26.68646	26.63201	26.69975	26.95394	26.85043
26.38113	25.12139	26.08335	26.26249	26.20583	26.78955	25.62087	25.06497	25.31354
26.20769	26.20769	25.93832	26.58978	26.61807	26.61807	26.51051	26.48155	26.57542
28.33671	28.52402	28.20303	28.34942	28.10649	28.34096	27.57542	27.75175	27.73247
26.78955	26.83846	27.2803	26.32732	26.37948	26.58978	26.51352	26.96499	26.61807
25.45199	25.39428	25.34773	25.52552	25.39428	25.71555	24.41054	24.91798	24.67034
27.67976	27.85043	27.67303	27.95947	27.85043	28.06599	27.95947	27.87408	28.03486
28.22619	28.38278	28.27142	28.23078	27.92025	28.00838	28.05051	27.99226	27.83846
25.54628	25.8811	24.87643	26.22435	26.23169	26.00838	26.96499	26.75175	26.61807
27.69312	28.0296	27.95947	28.18896	28.05051	28.04009	27.34942	27.14102	27.13124
27.41538	27.43141	27.57542	27.2535	27.43936	27.47846	27.52402	27.51653	27.45513
26.76446	26.24264	26.75175	26.45199	26.23535	26.61807	27.20769	26.61807	26.71293
27.93157	28.18423	28.07112	28.43936	28.4746	28.44332	28.46684	28.24898	28.32817

NaN	23.6971	24.60964	24.98468	NaN	24.01903	26.32732	26.20583	26.23535
27.11148	27.65949	27.05051	27.52402	27.35783	27.45513	27.43141	27.5969	27.509
27.5389	27.78332	27.62506	27.71947	27.73247	27.87994	26.80193	26.48769	26.86231
27.67303	27.96499	25.65403	25.64856	26.42822	26.27142	26.37119	26.86231	26.14297
28.08132	28.63547	28.258	28.16039	28.22159	28.20769	27.63201	27.77077	27.73892
25.51953	24.71293	24.49228	25.84326	25.38772	24.76446	26.81421	26.92025	26.53297
26.16039	25.97378	26.15653	26.60399	26.27142	27.06085	25.44884	25.01478	25.53742
28.29351	28.33671	28.49762	28.4512	28.62854	28.41135	28.3662	28.27142	28.47846
29.53705	29.71293	29.509	29.04009	29.104	29.27586	29.07623	29.16279	28.91456
27.23535	27.58978	27.16997	27.27142	27.19835	27.04009	26.41538	26.61807	26.64581
25.83364	25.65949	25.56092	25.75685	26.68646	26.72598	25.9955	26.69975	26.2589
27.11148	27.30661	27.12139	27.16039	26.96499	27.19835	27.3662	27.2803	27.391
27.55362	28.59335	27.61807	27.31527	27.27142	26.95394	NaN	NaN	NaN
27.57542	27.65267	27.81421	27.87994	27.78332	27.85638	27.91456	27.82031	27.99766
26.57542	26.23535	26.58978	26.61807	26.49228	26.43301	25.04009	24.98468	24.71816
23.51653	23.85757	23.82881	24.47383	23.48001	24.1946	25.51653	25.08537	25.59263
27.52402	27.73247	27.45513	26.97596	26.97596	27.09144	27.14102	27.04009	27.05051
28.82941	29.04009	29.06856	27.89157	28.48999	28.01903	27.47073	27.78332	27.77077
27.13124	27.28912	26.89735	27.56819	27.91456	27.05051	24.35111	23.32389	24.54038
25.1332	25.43301	25.12534	25.24627	25.37119	25.05672	25.59548	25.99118	25.76446
29.15798	29.40933	29.06599	29.20303	29.14589	29.1866	28.95394	28.78955	28.93438
29.19131	29.23535	29.06599	28.89446	29.08385	29.02168	28.95394	29.15557	28.81726
26.24808	26.54185	26.88577	27.16039	27.06085	27.20769	25.17188	25.45513	25.37119
28.71947	28.60399	28.52402	28.45513	28.42342	28.27586	26.90885	26.58978	26.54775
24.90426	25.51352	24.76446	24.89041	25.08132	24.84805	25.21697	24.97596	25.50142
25.98685	25.58118	25.44569	25.53148	25.67573	25.77706	25.17188	26.02538	25.75939
25.08132	25.02749	25.18329	24.91798	24.83846	24.90885	25.20955	25.07316	25.391
24.04427	24.34435	24.10149	23.63201	24.11744	23.50446	24.63201	24.78457	24.31008
27.34942	27.0296	27.30661	27.04009	27.2803	27.33245	27.15074	27.28912	27.1795
25.49838	25.52253	25.53148	25.30661	NaN	24.62087	NaN	NaN	NaN
27.48616	27.43141	27.19835	27.51653	27.5389	27.61807	27.80193	27.82639	27.78332
27.43936	27.35783	27.53148	27.81421	27.81421	28.19366	27.79576	27.14102	26.98685
26.61807	26.75175	26.4346	26.64581	26.82639	26.76446	27.5969	27.42342	27.42342
26.49534	25.9428	26.29439	26.85043	25.54922	26.35951	26.78955	26.65949	26.54628
25.24989	24.84326	24.65403	25.03171	24.86703	24.93157	25.31354	24.85281	25.239
27.06085	26.99766	26.95394	26.61807	26.53297	26.64581	26.21697	26.12731	26.07316
26.75175	26.58978	26.78955	26.38936	27.07112	27.45513	25.01052	24.85281	24.94949
26.61807	26.72598	26.39428	26.42982	26.86231	26.47846	27.47073	27.26249	27.391
NaN	24.99766	25.05258	25.04009	24.98033	25.29614	25.60682	25.47692	25.27142
23.57542	23.56384	NaN	22.69285	22.9812	NaN	25.98033	26.12534	26.1795
25.22435	26.05258	25.96939	25.54922	25.83605	25.50749	25.76699	25.74919	25.76192
25.08942	25.1757	25.01903	24.50446	24.51653	24.67034	24.35783	NaN	24.68646
28.04531	28.41941	28.31959	28.60045	28.63547	28.61456	28.94	28.94838	28.67303
26.65949	26.80193	26.67303	26.48769	26.44253	26.41216	26.76446	26.78955	26.78955
27.21697	26.72598	26.86231	26.53445	26.9428	26.63201	24.2535	23.92252	24.46139
28.58262	28.52776	28.27142	26.60399	26.65949	26.42982	21.8423	NaN	NaN
26.69975	26.67303	25.85519	26.24627	26.21141	26.17379	29.12386	29.15798	28.66628
27.69975	27.88577	27.34942	27.75812	27.47846	27.63893	27.74535	27.5969	27.5389
27.12139	27.13124	27.04009	26.51051	26.51503	26.67303	27.07112	27.08132	27.12139
23.93157	NaN	24.17188	25.33415	25.54038	25.04009	26.42502	26.52402	26.26607
24.10949	24.08537	24.44884	NaN	23.42982	23.87643	23.84805	24.06907	NaN
32.16639	31.42842	32.11458	31.78097	31.6501	31.90096	30.40324	30.38278	30.91171
NaN	NaN	19.54321	NaN	NaN	NaN	29.14346	28.84446	28.49381
28.4512	28.05569	28.509	27.83846	27.95947	28.05569	28.96224	28.72598	28.81726
29.31527	29.65438	29.48999	30.11148	29.92592	29.77863	28.85935	28.86231	28.83244
27.08132	26.60399	27.16039	27.47073	27.391	27.19835	25.7024	26.1757	26.1055
30.03486	30.21928	30.03486	30.26472	30.11148	30.16039	29.41337	29.3662	29.48999
26.68646	26.58978	26.60399	26.86231	26.55508	26.55801	26.51352	26.1946	26.31527
26.36786	26.55655	26.24445	26.86231	26.68646	26.69975	26.96499	26.80193	26.82639
28.37451	28.26249	28.391	27.29789	27.55362	27.57542	22.37782	23.57542	22.08537
26.72598	27.08132	26.97596	27.11148	26.55215	25.63201	25.83123	26.44411	25.87643
29.66628	29.5969	29.64581	29.43539	29.4073	29.32817	29.14589	29.07877	29.14831
28.63201	28.76762	28.69644	28.67976	28.52776	28.50522	28.77706	28.78019	28.82335
23.29614	24.41054	25.1055	23.71816	24.54038	24.07725	26.03171	25.83123	26.2856
25.32045	25.07316	25.73635	25.51352	25.84805	25.73892	24.91342	25.01903	25.05258
27.63201	27.56819	27.51653	28.02433	28.09144	28.31527	26.64581	26.56092	26.73892
26.09346	26.22619	26.41377	26.18329	25.9955	26.20955	25.36119	25.28207	25.32045
27.1795	26.83846	27.24445	26.77706	26.75175	26.45199	27.16997	27.34942	27.45513
25.45826	25.45826	25.91114	25.71555	25.99766	26.10949	24.80931	25.07316	24.51653
29.87115	29.54075	29.86378	29.61281	29.96774	29.7911	29.53334	29.7211	29.98142
27.0296	27.33245	27.09144	27.91456	27.58262	27.60399	26.41538	27.26249	26.69975
32.7751	32.6501	33.02168	32.36724	32.45807	32.55728	31.45807	31.80039	31.61982
26.43936	25.88577	26.04843	26.19084	26.63201	26.15846	NaN	27.21697	24.54628
27.11148	26.99766	27.11148	27.391	27.56819	27.46295	27.14102	27.10149	27.19835
26.69975	26.83846	26.58978	26.72598	26.78955	26.42021	26.95394	26.95394	26.69975
27.2535	27.0296	27.12139	27.09144	27.10149	27.10149	27.5389	27.63201	27.53148
29.77549	29.77235	30.00838	30.28692	30.27586	30.4919	29.87555	29.87115	30.07368
25.36119	25.82396	25.73117	25.88809	25.43936	25.03171	26.15653	25.93607	26.24989
27.58262	28.0296	27.92592	28.60752	28.33245	28.2803	28.43539	28.41941	28.4746
30.48232	30.61982	30.4334	30.69644	30.51089	30.68813	31.16039	30.78488	30.9746
29.02168	29.13124	29.54995	29.16039	29.00035	29.21234	29.99496	29.98142	29.83394
24.97596	NaN	24.42982	23.88577	NaN	24.33756	26.12337	26.20583	27.09144
27.13124	27.09144	27.21697	26.63201	26.77706	27.0296	27.49381	27.32389	27.38278
26.67303	26.12139	26.12337	26.39591	26.4073	26.2856	25.4073	25.19084	24.91342
26.55508	27.19835	26.57542	27.67976	27.3662	27.07112	26.30835	27.13124	26.16806
26.69975	27.00838	27.39917	27.56819	28.08639	28.22619	28.28912	28.46684	28.00838
27.11148	26.9428	27.3662	26.83846	27.10149	27.0296	27.15074	27.16039	27.10149
27.20769	27.62506	27.33245	27.15074	27.34096	27.04009	27.35783	27.34096	27.34942

26.50446	26.93157	26.98685	26.63201	26.55215	26.61807	27.35783	27.0296	27.19835
28.27142	28.29351	28.07623	26.77706	27.07112	27.391	NaN	24.27498	NaN
28.58978	26.90885	28.37036	28.86821	28.76129	28.95116	NaN	NaN	NaN
24.98902	24.06907	24.35111	NaN	24.33074	24.23169	26.11942	25.44253	25.53148
25.53148	25.317	25.24627	25.26428	25.8811	25.61807	26.20023	25.20955	25.22435
26.52402	27.15074	26.18518	26.38772	26.31527	26.37285	NaN	NaN	NaN
27.42342	27.55362	27.37451	27.57542	27.1795	27.16997	26.97596	27.53148	27.66628
24.6971	25.6971	26.1035	26.00838	25.49228	25.30661	25.58692	26.65949	26.35616
25.12534	25.48922	25.239	25.12139	25.33756	25.03591	25.84566	25.9955	25.7103
28.37451	28.65267	28.06085	28.43141	28.5969	27.98142	29.11644	28.98956	29.34942
26.52552	26.04635	26.06085	26.33756	26.04843	25.90656	25.80193	26.63201	26.42982
26.52402	24.64306	26.36953	25.92705	25.88577	26.85043	27.2535	27.22619	27.31527
25.86938	26.05466	25.55215	26.1488	26.77706	26.97596	24.79452	24.55801	24.80931
26.77706	26.89735	27.24445	26.36786	26.36119	26.1757	NaN	NaN	NaN
25.54038	24.89041	25.239	24.59263	25.12928	24.83846	25.14492	24.54628	24.07725
25.06085	25.53445	25.53742	NaN	25.02749	25.54333	25.32045	25.54922	26.50294
27.80193	27.87408	27.78332	27.27142	27.2535	27.39917	27.01903	26.85043	26.68646
25.44884	25.67842	25.34773	25.56964	25.12534	25.38443	25.56674	25.48001	25.58692
29.0296	29.13124	28.85341	28.61807	28.57181	28.69644	27.00838	26.9428	27.01903
27.14102	27.09144	27.10149	27.28912	27.20769	27.43936	27.04009	26.85043	27.07112
27.26249	27.47073	27.22619	27.72598	27.66628	27.69975	26.85043	26.78955	26.86231
24.40406	24.23169	25.50446	24.2535	24.00196	23.391	25.57254	25.06085	24.50446
NaN	NaN	NaN	24.91798	NaN	23.94949	24.91342	25.32732	24.87643
25.65949	25.38443	25.22435	26.15846	25.93157	25.8811	26.72598	26.82639	26.90885
25.47383	25.72858	25.53742	25.52552	25.26785	25.49838	25.83364	26.08537	25.75685
26.41216	26.14686	26.21512	26.20583	26.29964	26.03171	24.45513	25.51953	25.22803
27.64581	NaN	24.44253	22.78157	24.42342	26.63201	25.27853	23.66492	24.72858
25.58692	23.13946	25.22803	25.55508	25.56092	25.46139	26.03591	25.54628	26.05879
27.14102	26.53148	27.07112	26.04427	25.89041	26.4073	25.21327	24.82881	25.06907
26.45199	26.30835	26.37285	26.69975	26.88577	27.0296	26.11545	26.23352	25.72338
25.51352	25.70504	25.64581	25.97378	25.75175	25.72598	25.24627	25.16039	25.40406
25.94503	25.67573	25.61246	25.46451	25.51653	25.1332	25.81665	26.04635	25.86938
27.97596	27.97596	27.93157	28.16997	28.27142	28.1795	28.17474	28.39917	28.31527
26.23352	26.61807	26.03171	26.49381	26.39917	25.81909	26.18707	25.97596	26.05466
26.69975	27.04009	26.64581	27.1795	27.08132	27.14102	27.92592	27.83846	27.91456
25.64581	24.76446	26.56964	24.95394	26.26964	26.44884	NaN	22.25638	21.26499
26.00624	25.96279	25.98902	26.27853	26.1035	26.00624	27.26249	26.93157	27.1795
26.27498	26.37782	26.27853	26.07725	26.19272	26.07521	26.35279	27.09144	25.95837
27.61807	27.77077	27.4073	25.33756	25.18329	25.81176	NaN	22.70345	NaN
28.60399	28.5969	28.53519	28.36202	28.33671	28.5862	22.47754	NaN	NaN
26.07725	25.74663	26.31008	26.391	26.41216	26.60399	27.24445	27.06085	27.05051
29.95394	29.88722	29.86231	30.2535	30.37244	30.45317	29.5591	29.391	29.3452
26.4008	25.85519	26.37285	26.69975	26.44884	26.54922	NaN	22.38311	24.20955
26.77706	26.54775	26.41377	26.65949	26.55508	26.68646	27.2535	27.0296	27.35783
27.18896	27.08132	27.12139	27.43141	27.42342	27.50142	26.48155	26.73892	26.07928
25.1055	25.52253	25.1795	25.82153	25.66221	25.90426	26.46607	25.67573	26.29263
NaN	25.35783	NaN	24.98033	NaN	NaN	28.7162	28.6764	28.23078
28.72598	28.32389	28.52402	28.84146	28.79885	28.95671	28.53519	28.46295	28.4746
29.32603	29.33031	29.36202	29.61456	29.60399	29.7597	29.4453	29.23306	29.42542
25.58405	25.43619	25.79699	26.13124	26.98685	27.16997	NaN	23.07235	NaN
25.17188	25.50749	25.28912	25.04843	25.94726	25.73117	24.67034	24.97596	25.00624
26.63201	26.77706	26.83846	26.78955	26.75175	26.78955	27.29789	27.26249	27.19835
28.05051	28.01903	27.58978	27.47846	27.1795	27.43141	NaN	NaN	NaN
26.24082	24.57542	24.32389	26.82639	26.99766	26.64581	27.52402	26.89735	27.55362
26.07725	26.45356	25.38443	26.63201	26.63201	26.30138	26.16997	26.53742	25.51051
25.90426	25.97596	25.76951	25.19084	25.28207	25.42021	25.45513	25.48922	25.66763
26.04218	26.00838	26.05879	25.79947	25.58692	25.85757	24.86703	25.42982	25.46762
25.45513	25.33756	25.46451	24.76446	NaN	24.60964	25.27142	25.20583	25.63478
26.30487	25.78457	26.01903	26.18896	25.82396	26.0041	26.57254	26.32045	26.39754
26.22435	26.36453	26.12337	26.54333	26.61807	26.40406	26.26428	25.60682	25.97596
23.56384	24.42982	NaN	25.08537	24.99335	25.07725	25.93157	25.83605	25.77455
25.88577	26.20583	25.85281	25.81909	25.4008	25.03171	26.13124	25.80686	26.04843
25.38772	25.79947	25.97378	25.96499	25.12534	25.1757	24.72338	24.31008	23.60964
23.32389	24.06907	24.79452	23.49228	23.44253	21.66275	26.46762	26.46295	26.55801
26.88577	27.33245	27.10149	26.88577	26.65949	26.58978	24.26069	24.01052	24.91798
25.08132	25.38113	24.84326	25.00624	24.92252	24.98033	NaN	NaN	NaN
26.80193	27.12139	26.98685	26.82639	26.87408	26.86231	27.11148	27.391	27.11148
26.76446	27.12139	26.78955	27.19835	27.26249	27.14102	26.09346	26.04427	25.41054
25.73635	25.7024	26.03591	26.05879	26.038	26.05258	26.49686	26.55508	26.41216
24.62087	24.34435	NaN	24.8044	23.67573	25.44253	24.32389	25.94056	25.69975
26.02538	24.59263	24.11744	24.1795	NaN	24.58692	23.94949	24.33756	NaN
24.79452	24.57542	23.2535	23.85757	26.38607	24.18707	25.30661	25.86467	25.90426
NaN	NaN	NaN	NaN	NaN	NaN	24.83846	24.81421	24.67573
24.93157	25.16423	24.37782	25.01052	24.76951	25.14492	25.84805	25.7543	25.69179
29.06343	29.68979	28.61456	28.5862	28.79885	28.35783	29.53148	29.07368	29.03486
23.71816	25.61807	23.99335	25.10149	25.26069	25.26069	26.34266	25.86231	26.01478
27.77077	28.15074	26.19272	25.16039	25.53742	25.16806	23.55215	21.61863	NaN
27.67303	27.73892	27.65267	27.71293	27.64581	27.90885	27.28912	27.0296	27.42342
25.76446	25.61246	25.52253	26.29439	26.56092	26.05258	25.78706	26.34266	26.1488
26.44095	26.29263	26.45042	26.42502	26.45042	26.52999	26.48616	26.23352	26.30835
25.75175	27.30661	26.37285	26.12139	26.01478	27.30661	24.58692	24.54038	25.81909
25.09748	25.94503	24.97158	24.417	24.55215	25.42982	NaN	NaN	NaN
26.48308	26.02749	26.78955	26.08132	27.12139	26.39591	24.81421	24.15653	24.07725
26.20396	26.0041	26.24808	26.44727	26.44727	26.72598	26.21327	26.82639	26.35279
25.30313	24.51051	25.2571	25.82153	25.46139	25.32045	25.4073	24.87173	25.69179
24.69179	24.94056	24.42982	24.94503	24.85281	24.88577	24.50446	24.44884	24.79452
25.62087	26.60399	25.37119	25.08942	25.54038	25.61807	25.35111	25.80193	25.42982
27.65267	27.19835	27.05051	26.58978	26.99766	27.15074	25.39428	25.8044	NaN

28.34096	28.53519	28.3452	28.44332	28.31095	28.56819	NaN	NaN	NaN
27.76446	27.80193	27.47073	28.00303	28.06599	27.96499	26.99766	26.95394	26.9428
25.82153	24.7543	24.99766	24.8044	25.10949	25.75685	24.03591	23.31008	22.55918
25.16806	25.74919	25.92705	26.12139	26.54038	25.81665	24.1488	23.74919	23.95837
26.27142	26.44727	26.23535	26.69975	26.89735	26.72598	25.53445	25.77957	25.75685
29.04791	28.87701	28.92592	28.74535	28.87701	28.82335	28.92592	28.86526	28.78644
28.07112	28.41538	27.93157	28.07112	28.22159	28.26249	27.0296	27.13124	26.99766
24.79452	24.33074	24.57542	24.58692	24.97596	25.09748	24.56384	25.06907	25.16806
25.63755	25.95615	24.95394	25.07725	26.65949	25.08942	23.56384	23.72858	NaN
NaN	NaN	NaN	NaN	NaN	NaN	28.48232	28.509	28.16997
26.21882	25.98902	26.19835	26.47228	26.55069	26.03171	26.12139	26.07521	25.87408
25.88577	26.00196	25.72598	25.95615	25.82639	25.92252	25.04427	24.87173	25.28912
31.08005	31.07368	31.16639	29.88577	30.03486	30.00838	29.21466	29.23763	29.14346
25.11347	25.6971	25.54628	25.71816	25.65949	25.50749	25.9955	26.48308	25.89966
29.57542	29.66966	29.40933	28.87408	28.60045	28.7357	27.98685	27.92025	28.0296
26.61807	26.61807	27.11148	26.239	26.25169	26.37948	25.24989	25.71816	25.56384
26.09748	26.0874	26.03591	26.08942	25.91114	26.16423	26.50294	26.27142	26.35616
26.47537	26.89735	26.35616	26.30487	26.24445	26.34435	25.77203	25.64306	25.69975
29.63027	29.76288	29.74855	29.71784	29.64924	29.59156	NaN	22.87267	19.17035
25.71293	26.29964	25.81176	26.54481	26.19648	26.21882	27.28912	26.95394	27.29789
27.56092	27.58978	27.52402	27.35783	27.51653	27.61807	27.35783	27.05051	27.34096
27.75175	28.09648	27.54628	27.49381	27.71947	27.90311	27.31527	27.38278	27.21697
26.78955	27.08132	26.89735	26.60399	26.48616	26.2856	26.4567	26.01691	26.31527
27.96499	28.01903	28.19835	28.14589	28.21697	28.12139	27.86821	28.02433	27.90311
34.19014	34.36984	34.36333	33.38278	33.31689	33.44579	32.3802	32.51559	32.33565
NaN	NaN	25.1946	25.01478	24.94949	24.96279	NaN	24.28912	24.29614
28.02433	28.05569	27.9428	27.33245	27.60399	27.31527	28.34096	28.5389	28.04009
27.48616	27.11148	29.00303	28.0296	26.28736	28.12633	24.83364	24.64306	25.79452
26.85043	27.27142	26.81421	26.54481	26.65949	26.75175	26.239	26.00838	26.09547
28.57903	28.72923	28.87701	28.46295	28.41135	28.32389	28.81421	28.74535	28.87115
31.29242	31.09899	31.27586	31.64581	31.86452	31.86821	31.00169	31.22504	31.09899
27.94838	28.39509	28.0296	27.96499	27.83846	27.80193	27.72598	27.90885	27.92025
30.20769	30.13614	30.39305	30.42342	30.38278	30.54812	30.27586	30.06085	30.38278
25.87408	25.75939	26.4008	25.72338	26.43619	27.08132	25.02749	25.56674	24.78457
23.99335	NaN	NaN	NaN	NaN	NaN	25.56674	25.10949	25.65676
28.01372	27.75812	28.19366	28.05051	28.00838	27.99226	27.61807	27.65267	27.78332
24.91342	25.43619	25.08537	25.33756	25.13712	25.11347	25.94056	25.84326	25.7543
26.09144	26.06702	26.11545	26.2021	26.19084	26.04843	26.04009	26.0296	25.96719
26.26785	26.71293	26.10749	26.85043	26.78955	26.64581	25.74663	25.35783	25.96939
26.9428	27.09144	26.83846	26.93157	27.14102	26.88577	26.82639	26.69975	26.75175
26.16615	25.24264	25.27142	24.59833	23.50446	24.48616	24.76951	24.14102	23.72858
25.48922	26.41054	26.19835	26.05051	26.01903	26.26607	24.46139	23.23169	24.02749
27.23535	27.63201	27.84446	27.24445	27.01903	26.99766	24.49228	NaN	NaN
26.97596	27.10149	27.13124	27.2803	27.24445	27.34942	26.81421	26.73892	26.78955
27.0296	26.44095	26.69975	26.81421	26.57542	26.58978	25.22803	24.95837	24.36453
25.62087	25.57831	25.58978	23.87643	NaN	NaN	27.04009	26.99766	27.20769
27.78332	27.87408	27.82031	27.75175	27.99766	27.73892	27.34096	27.44727	27.39917
27.55362	27.33245	27.58262	26.88577	26.71293	26.71293	24.98033	24.10949	25.60399
27.07112	27.39917	27.21697	27.27142	26.88577	26.81421	25.89273	26.58978	26.44884
24.2535	23.07561	23.74919	23.96719	25.08537	24.00196	26.13124	26.07316	25.81909
25.82396	25.58692	25.76699	25.22803	25.03171	25.24989	24.56964	24.14102	24.64856
26.57254	26.83846	25.54922	26.63201	26.09949	26.18518	27.30661	27.06085	27.2535
26.78955	26.49838	26.98685	26.37617	26.73892	26.63201	26.39264	26.46762	26.317
27.30661	27.1795	27.28912	27.5969	27.41538	27.48616	26.97596	27.08132	26.96499
NaN	NaN	NaN	25.42982	25.13712	24.99766	26.13516	26.34942	26.20955
26.04427	26.08335	26.16423	26.9428	27.14102	27.11148	27.11148	27.06085	27.29789
26.04009	25.04009	25.04843	24.83364	24.47383	25.43936	24.64856	23.07235	24.239
25.36119	25.24627	24.72338	25.52552	25.38772	24.80931	24.49838	25.35448	24.79452
27.16997	26.83846	26.93157	26.73892	26.60399	26.87408	27.45513	27.11148	27.43141
24.66492	25.59263	26.58978	25.79452	26.4567	27.08132	27.00838	26.93157	26.58978
27.05051	26.43141	27.391	28.04531	27.19835	27.26249	23.05755	22.47259	NaN
24.2021	24.97596	23.77957	24.33756	24.34435	24.1488	25.30661	26.00838	25.317
24.72858	24.94503	24.5285	25.06907	24.88577	24.71293	26.09547	25.55215	26.00624
NaN	NaN	NaN	24.06907	24.16423	23.79947	24.6811	24.48001	24.6811
28.01903	28.43936	28.15557	28.02433	28.10649	27.97596	27.63201	27.63201	27.3662
NaN	NaN	NaN	NaN	NaN	NaN	28.53148	28.30661	28.52028
24.53445	24.47383	24.50446	24.29614	23.07235	24.58692	24.55215	24.26785	23.78955
26.60399	25.90196	26.80193	27.60399	27.42342	27.47073	26.82639	26.47228	26.75175
26.60399	26.52999	26.49228	26.24445	26.06085	25.95172	25.7543	25.87408	25.47383
NaN	NaN	NaN	NaN	24.01052	NaN	25.16423	25.18707	25.74919
25.96499	26.27498	26.2803	25.87173	25.76192	25.7024	25.34435	26.36119	26.02538
26.03591	25.96058	25.7543	24.42342	24.74919	25.04427	NaN	NaN	NaN
29.16997	29.09144	29.34731	28.61456	28.74855	28.89446	29.4919	29.47653	29.57001
26.52999	26.86231	26.11148	26.87408	27.10149	26.99766	25.54922	25.89273	25.85281
21.85377	NaN	21.21252	24.5285	23.391	23.87643	27.26249	26.72598	26.77706
25.53742	25.91798	24.92252	25.317	26.04009	25.59548	NaN	NaN	NaN
27.77706	27.65949	27.3662	26.98685	27.21697	27.12139	26.63201	26.48616	26.43141
27.3662	27.77706	27.31527	27.58262	27.27142	27.391	28.01372	27.75812	27.61105
27.0296	27.2803	27.20769	26.69975	25.62366	25.79699	25.76192	25.14102	25.44569
26.11942	26.28912	26.28736	25.89735	25.71555	26.08537	25.94503	25.98685	26.21697
28.53148	28.58978	28.5389	28.73892	28.60045	28.64238	27.83244	27.89735	27.86821
25.55508	25.61246	25.98685	25.78207	25.8044	25.67303	NaN	23.417	23.86703
25.66221	26.04009	25.77455	26.10749	26.16806	26.22435	26.16423	25.57542	25.7415
27.07112	26.68646	26.67303	26.02749	26.17188	25.57542	24.47383	24.54628	24.17188
27.30661	27.31527	27.35783	27.42342	27.61105	27.54628	27.33245	27.30661	27.21697
27.38278	26.78955	27.2803	27.78955	27.06085	27.30661	27.29789	27.10149	27.5389
25.96939	26.04843	26.37285	26.4008	25.90656	26.29439	25.41054	26.038	25.54922
23.98468	24.51051	24.47383	25.19084	24.93607	24.57542	24.82396	25.01052	24.94056

27.86231	26.90885	28.0296	28.73247	28.41538	28.00303	28.53148	29.08385	28.84745
25.6513	25.08537	25.01478	25.64031	25.60964	25.58405	26.01691	26.28383	26.23717
28.76762	28.41135	28.57181	28.85341	28.65267	28.86231	29.11396	28.95394	29.30661
25.76192	26.06291	25.85043	25.46139	25.65949	25.77455	26.05879	26.01903	25.8044
24.97596	25.77455	25.81176	25.62087	25.39428	25.61526	25.77706	25.82639	25.58692
27.97049	28.14102	27.91456	27.71947	27.66628	27.75175	27.2535	26.75175	27.12139
25.93607	25.53742	25.82153	25.68646	25.4008	25.37451	26.34773	25.81665	26.12534
26.50749	26.27142	26.75175	25.28912	25.74407	25.92252	25.44253	26.14297	25.97158
26.52253	27.11148	27.58262	25.86938	24.28912	25.70767	28.12139	29.96774	29.104
27.84446	27.73892	27.79576	27.73892	27.78332	28.00838	26.82639	26.86231	26.9428
26.44569	26.22251	26.68646	26.85043	26.56238	26.75175	26.58978	26.75175	26.54333
24.79947	24.67573	24.92705	24.89041	24.95394	24.62087	24.91798	25.2856	25.31008
26.68646	27.05051	27.4073	27.08132	27.13124	26.73892	27.07112	27.95394	28.09144
26.86231	25.7024	26.88577	27.08132	26.22251	26.88577	19.83923	19.88911	22.84995
25.48616	25.63201	25.09346	25.13712	25.03171	25.04009	24.83364	NaN	24.90885
28.96224	29.17474	28.95947	28.46684	28.54628	28.47073	27.47073	27.86821	27.97049
25.72598	25.78955	25.78955	25.72338	25.70767	25.80686	26.19272	25.65949	26.09346
24.97596	24.57542	25.08132	25.76446	25.65403	26.20396	24.94949	25.05258	25.23535
25.1795	25.48922	25.03171	25.33756	25.31008	25.30313	25.7024	25.37451	25.98033
24.55215	24.98033	24.49838	25.6971	25.1946	25.46139	24.55801	25.22435	24.51051
NaN	NaN	24.05258	23.93157	24.21697	24.37782	24.28912	24.43619	24.82881
25.55508	25.60399	25.64856	26.41054	26.73892	26.21512	26.75175	26.65949	26.58978
26.1795	26.47846	26.55508	26.4008	25.80686	26.53594	27.15074	27.05051	27.06085
25.51653	25.29614	25.41054	25.16423	25.20955	25.59833	25.82396	25.96719	25.7103
28.5969	28.54995	28.70965	29.01372	28.88285	28.98685	28.60399	28.38278	28.58262
27.11148	27.08132	26.61807	27.10149	26.81421	27.05051	26.06702	26.05051	26.23352
25.42021	26.08942	25.51953	25.94949	26.23352	26.13124	26.09144	26.11744	26.06907
26.58978	26.86231	27.60399	26.90885	27.07112	26.67303	24.98033	26.18139	26.30661
24.23169	24.61526	24.49838	24.79947	24.67034	24.239	23.64306	24.71293	NaN
26.53148	26.85043	26.71293	26.71293	26.16423	26.20955	26.9428	27.08132	27.07112
25.2535	26.90885	27.06085	27.2803	27.53148	27.48616	22.46762	22.50446	NaN
25.79947	26.03381	25.94056	26.26607	25.77455	25.16423	25.36786	25.33756	25.59833
28.57542	28.69975	28.55728	28.73892	28.47846	28.54995	27.50142	27.3662	27.61105
24.51653	24.78457	24.91798	24.77957	25.11744	25.2571	26.50597	25.99118	26.48001
26.40243	26.23352	26.21327	26.60399	25.92025	26.04427	24.90426	25.58118	24.76446
27.16039	27.12139	27.01903	26.83846	27.00838	26.87408	24.69179	25.06085	25.44884
25.29614	23.31008	24.89504	25.6513	25.33415	25.59263	NaN	23.6971	NaN
25.23169	22.13006	24.77957	24.98902	25.01478	23.97596	23.91342	24.59833	25.417
24.04427	24.37782	23.79947	24.67573	24.34435	24.10949	25.7103	25.87173	25.18329
24.51051	26.01478	24.11744	25.03591	25.79203	25.54038	25.78457	25.47073	26.16615
23.36453	24.08537	NaN	24.27498	24.18707	24.73376	25.58692	25.02327	24.96719
29.73731	29.72923	29.77706	29.47846	29.29789	29.10899	24.14102	23.6971	22.01903
26.4186	24.87643	27.10149	26.03381	25.77706	NaN	26.82639	28.57903	NaN
24.42982	23.89504	24.72858	24.1946	23.51653	25.15653	22.80341	22.3073	22.1795
26.41054	25.77455	25.81665	25.96939	26.08537	25.62923	22.6971	NaN	NaN
26.06291	26.28383	26.03381	25.84566	26.038	26.08942	25.76699	25.5285	25.38113
27.93157	28.06085	27.83846	27.58978	27.71293	27.71293	27.92025	27.77706	27.87994
27.47073	27.47846	27.85043	27.39917	27.63201	27.71947	27.77706	27.34096	27.4073
25.31008	26.78955	26.20955	26.64581	24.2021	26.19272	26.99766	26.86231	27.07112
27.65949	26.68646	27.18896	27.26249	26.68646	26.47846	27.56819	27.27142	27.3662
NaN	NaN	NaN	NaN	NaN	NaN	25.92025	25.79452	25.71555
NaN	23.32389	24.75939	23.73892	NaN	NaN	26.19272	26.86231	26.57542
23.97596	24.14102	24.35783	23.92252	23.93157	24.41054	23.66492	24.06085	24.44253
NaN	NaN	NaN	NaN	NaN	NaN	27.57542	27.46295	27.51653
25.55508	25.72858	25.1332	25.23535	24.97596	25.1946	25.90885	25.78207	25.68913
26.06907	26.45199	25.94726	25.86938	25.92252	25.79452	25.37119	25.41377	25.58692
26.47228	26.72598	26.30487	26.71293	26.68646	26.55069	26.04843	25.76699	26.13124
NaN	24.58118	NaN	25.1795	24.70767	NaN	24.55801	25.9157	25.76446
30.65438	30.59335	30.64581	30.06085	30.07368	30.06085	30.38278	30.52962	30.41337
30.69644	30.78488	31.01505	30.58441	30.7692	30.41337	30.21928	30.4334	30.35153
26.90885	26.81421	26.80193	26.40243	26.60399	26.60399	27.61105	27.85043	27.55362
26.92025	26.92025	26.77706	27.06085	26.98685	26.61807	26.14297	26.391	26.82639
24.58692	24.67573	24.51051	25.26785	25.06085	25.23169	25.2535	24.81909	25.40406
29.29132	29.41538	29.37036	29.36828	29.33884	29.25575	29.30226	29.15074	29.33245
27.76446	28.29789	27.84446	29.28251	28.54628	28.63201	27.57542	27.65949	28.258
26.88577	26.96499	26.68646	26.07725	26.68646	25.62923	26.50749	26.28912	26.13712
26.75175	26.38607	26.58978	26.65949	26.63201	26.58978	27.28912	27.76446	27.32389
24.97158	25.31354	25.23535	24.86231	24.83846	25.06497	25.42662	25.46451	25.13712
27.75175	27.58978	28.51653	27.44727	27.85638	27.87408	27.09144	26.95394	27.53148
25.77203	25.34773	25.39428	25.87877	25.65949	25.56092	25.27853	24.91342	25.2856
26.33245	26.4346	26.24627	26.50749	26.64581	26.5389	26.509	26.13124	26.44095
NaN	NaN	NaN	25.09748	NaN	25.32732	25.83123	25.54038	25.87877
26.05672	25.97596	25.62923	25.32045	25.6811	25.90196	25.97158	26.11347	25.8044
25.73117	26.04009	25.95837	25.71816	25.83123	25.78955	25.51051	25.30313	25.2571
25.44569	25.26785	25.33756	24.76446	25.19084	25.1946	NaN	24.26069	NaN
NaN	23.66492	NaN	24.73376	24.55215	24.30313	25.03591	25.51051	25.14492
25.12928	24.94503	24.90885	25.61246	25.52552	25.53148	24.68646	25.05258	24.5285
30.04791	30.17236	30.04791	29.96774	30.02168	30.24218	29.89446	29.77077	29.98142
26.24082	26.45513	26.21512	25.82639	25.97158	26.18329	25.89273	25.7543	25.92252
24.51051	25.46762	24.97596	25.03591	25.16806	24.68646	25.58405	26.06702	25.62087
25.89966	26.38936	25.86703	26.17188	26.29263	25.73376	25.99118	25.85757	25.78706
28.2803	28.41941	28.51653	28.58978	28.51277	28.10149	27.22619	27.23535	27.30661
26.9428	26.65949	26.92025	26.29964	26.82639	26.9428	27.79576	27.81421	27.92592
26.97596	27.28912	26.81421	26.73892	26.64581	26.55947	26.80193	26.35279	26.24082
29.41337	29.32389	29.51653	29.27364	29.4649	29.19131	28.24445	28.04531	28.30226
26.92025	26.96499	26.64581	26.37617	26.26249	26.46918	26.17188	26.20955	26.0041
25.46139	26.17188	25.46139	26.85043	26.18707	27.27142	26.63201	26.93157	27.1795
25.04009	24.62645	25.19084	26.20583	26.30487	26.16997	26.04635	27.13124	26.46139

28.3452	28.70636	28.49762	29.11148	28.80501	28.74214	27.71947	27.61807	27.75175
26.03591	25.70767	26.24989	25.90656	25.82153	25.35448	26.1332	25.38443	26.07725
25.6811	25.7103	25.32389	25.317	25.23169	25.44253	25.39754	25.4073	25.50749
28.03486	26.57542	27.71293	28.45513	28.46295	28.43539	28.74535	28.55362	28.70306
24.8044	24.6971	24.91798	23.87643	23.50446	23.1946	24.20955	24.42982	25.64581
26.21327	26.29789	26.1795	26.34773	26.55362	26.53297	24.67034	24.41054	25.1795
NaN	24.98902	24.1488	23.88577	NaN	23.92252	25.36119	25.27853	25.56964
25.78207	26.05258	26.0041	25.86703	25.83605	25.91114	25.7415	25.94949	25.88344
24.82396	NaN	23.23023	NaN	25.11347	25.24264	NaN	26.038	24.82396
25.70504	25.80686	25.46762	25.74407	26.11347	25.79947	25.77706	26.55362	25.85281
25.15653	25.26069	24.97596	25.39754	25.2535	27.69312	25.04843	24.73892	25.01052
23.75939	24.15653	23.56384	23.91342	24.01052	24.21697	25.84086	26.04218	25.63478
26.36453	26.317	25.87408	27.21697	26.72598	26.71293	26.83846	27.09144	27.1795
23.80931	23.68646	23.50446	23.67573	24.03591	23.21104	25.22435	25.09346	24.5285
NaN	NaN	NaN	NaN	NaN	NaN	25.91114	26.16039	25.87408
24.63755	25.41054	25.35111	25.26428	24.87643	24.86231	25.04427	24.8811	25.18707
26.57254	26.65949	26.19648	26.64581	26.38278	26.29789	25.59548	25.53148	25.21327
25.62645	26.28912	26.13712	25.53445	25.28912	24.56384	NaN	NaN	NaN
25.14492	25.32389	24.93157	24.96719	25.90426	25.21327	23.73892	NaN	NaN
24.89504	NaN	24.60964	24.83846	24.93607	27.16039	27.20769	26.77706	26.93157
23.07887	24.06907	24.1946	24.317	24.35111	23.79947	25.83605	24.55801	25.84086
25.19084	NaN	NaN	24.45513	24.1946	24.91798	24.91798	25.1757	24.82396
26.16231	25.94056	25.99981	25.68913	26.44727	26.509	26.49534	26.39428	26.39264
26.61807	26.45042	26.32045	27.44727	27.1795	27.4073	26.49381	26.47228	26.31354
26.87408	26.83846	26.90885	26.73892	26.96499	26.63201	26.63201	26.75175	27.22619
26.97596	27.08132	26.98685	27.07112	27.06085	27.09144	28.03486	27.73247	28.06085
26.49076	26.88577	26.37782	26.41377	26.09144	26.11744	26.63201	26.48308	26.68646
25.93157	26.01052	25.34773	26.38772	26.05051	26.3256	26.08537	26.23717	26.04218
27.58978	28.1795	28.12633	27.68646	27.68646	27.71293	25.28912	24.67573	25.64306
25.31008	25.30313	25.14492	25.27498	25.61807	25.12534	25.67573	25.80686	25.43301
27.19835	27.77077	27.00838	27.56819	27.42342	27.43141	27.2803	27.07112	27.00838
26.1035	25.42982	25.2535	25.43936	26.02327	26.23352	25.98685	25.75939	25.64581
25.12534	25.239	25.47383	25.45199	25.42662	25.78706	24.96279	25.13712	25.21697
26.80193	27.16039	26.55655	26.58978	26.56674	26.55069	26.35951	26.61807	26.37451
25.20955	23.91342	25.53148	23.59833	24.417	24.01052	25.59548	25.24989	25.1055
27.07112	27.41538	27.26249	27.43141	27.44727	27.55362	27.56819	27.56092	27.58978
30.40324	30.55728	30.47267	30.70471	30.60222	30.69644	30.61105	30.41337	30.46295
28.39917	28.23535	28.67976	27.20769	27.1795	26.34435	24.62087	NaN	25.52253
28.30226	27.89735	28.16519	29.0296	28.43539	28.56456	28.52028	28.04531	28.16997
NaN	23.66492	23.37782	22.69073	22.11108	23.23169	NaN	NaN	24.94503
26.92025	26.69975	27.32389	NaN	26.92025	25.32732	26.63201	26.24989	26.05258
23.81909	24.20955	23.92252	24.55215	24.59263	24.78457	24.50446	24.31008	24.42982
26.57254	26.76446	26.77706	26.81421	26.60399	26.55362	26.05051	26.44884	26.03591
26.40892	27.47073	26.56674	25.99981	26.05672	26.12337	26.50446	26.53445	26.49686
25.79203	26.18518	25.85757	26.04427	26.20955	26.46139	25.58692	25.38113	25.06907
25.52552	25.24627	25.2571	26.90885	26.57109	26.64581	27.42342	27.29789	27.62506
27.82031	28.06599	27.91456	27.79576	27.76446	27.69312	NaN	NaN	NaN
26.35279	26.72598	26.10749	26.21512	26.31354	26.29439	25.92705	26.15074	25.88344
28.10149	27.69975	28.11644	28.44332	28.49762	28.55728	28.16997	28.13614	28.15557
26.50294	26.04218	25.60964	26.61807	26.43778	26.38936	25.88577	26.72598	26.239
26.07112	23.04926	26.37285	25.97378	25.64031	26.04218	NaN	25.15653	25.79203
27.61105	27.07112	27.28912	27.86821	27.67303	27.69975	27.04009	26.9428	26.65949
23.88577	24.26785	23.42982	25.04009	25.21697	24.39754	22.16116	25.10949	NaN
25.48922	25.89966	25.8044	24.66492	25.07316	24.74919	25.79452	25.41054	25.49228
24.53445	24.391	24.44253	24.54038	24.51653	24.417	25.7415	26.41377	25.89504
25.19084	24.29614	NaN	NaN	24.82396	NaN	25.51051	25.7415	25.93157
25.95394	26.10949	25.94726	26.51953	26.20769	25.89504	26.83846	26.4999	26.49381
25.24264	23.71816	25.92931	26.69975	25.6971	26.58978	22.97596	NaN	23.89504
24.09346	NaN	24.43619	24.01052	24.04427	23.91342	24.06907	24.36453	24.239
24.84326	25.17188	24.67573	24.62645	24.42342	23.90426	25.48001	25.60964	25.44569
25.27498	24.239	25.38443	25.63201	25.40406	25.61807	24.79452	25.18329	24.54628
25.9955	26.37948	25.98033	25.54038	25.38113	25.82153	NaN	NaN	NaN
26.92025	27.0296	26.87408	26.93157	26.68646	26.64581	26.37451	26.01478	25.84805
27.76446	27.83846	27.85043	27.77706	27.82031	27.5969	27.54628	27.66628	27.85043
NaN	NaN	24.84805	NaN	24.01052	24.40406	24.59833	25.50446	25.2571
27.04009	27.07112	27.11148	27.4073	27.18896	27.08132	27.0296	26.80193	26.83846
26.48308	26.58978	26.4346	26.97596	26.95394	26.89735	26.2732	26.17379	26.56384
NaN	NaN	22.93517	23.51653	23.42982	23.77957	25.1757	24.59263	24.8044
25.59833	25.83605	25.84086	25.23535	25.36119	25.34773	25.85043	26.0874	25.61807
25.2856	25.38113	25.2021	26.13712	26.07928	25.95394	25.34773	25.78706	25.62923
23.26785	NaN	23.86703	23.35111	23.01052	23.94056	24.09346	24.16423	23.60964
25.06085	24.77957	25.01903	25.27853	25.06907	25.08132	25.43301	25.15267	25.42662
26.65949	26.76446	26.82639	26.39428	26.41377	26.48155	27.10149	27.19835	27.01903
27.83244	27.55362	28.0296	27.45513	27.22619	27.48616	25.48616	25.16806	26.81421
24.391	24.26785	24.6971	24.2021	24.68646	24.83846	25.07316	24.81909	25.06907
28.26696	28.32817	28.3452	28.21697	28.16519	28.11644	27.11148	27.28912	27.1795
27.20769	26.96499	26.78955	26.78955	26.67303	25.87643	26.97596	26.9428	26.61807
24.78457	25.09346	24.76951	24.59263	24.58692	25.37451	24.98902	24.80931	25.317
26.01265	26.48155	25.93382	26.04635	25.72078	26.09748	26.42342	26.22435	26.417
25.1757	25.34096	24.85281	25.2535	25.35448	25.00624	25.1946	25.26785	25.30313
NaN	NaN	23.48001	23.98468	NaN	NaN	24.33074	24.28912	24.36453
25.31354	25.80193	25.30313	25.26785	25.49838	25.09346	24.92252	24.94056	25.06497
25.91798	26.35951	26.22803	25.71293	26.22435	26.14297	26.67303	27.06085	26.51352
23.50446	NaN	23.55215	23.72858	23.42982	24.1332	24.5285	24.89041	24.62087
26.20955	26.26069	26.64581	25.77957	25.04843	25.76699	25.35783	24.96279	25.54038
23.97596	24.26069	23.86703	23.76951	24.54628	23.93157	NaN	23.24771	NaN
25.5285	27.89157	25.7543	25.70767	25.24989	25.24989	24.95837	24.54038	25.01052
25.13712	25.317	24.95837	25.60682	25.62923	25.56384	25.16806	25.42021	25.1757

24.48616	26.33245	24.81421	26.37948	26.00624	25.79452	25.62087	25.93157	26.33245
24.27498	24.2535	23.88577	24.04427	24.28912	23.65403	NaN	NaN	NaN
24.42982	NaN	24.11744	24.03591	24.5285	24.56964	25.03591	25.79452	24.51051
23.65403	23.84805	23.65403	23.88577	23.67573	24.07725	24.5285	24.18707	24.37782
28.74535	28.91456	28.75494	28.52402	28.41941	28.41538	28.34942	28.32389	28.35363
23.44253	24.18707	23.94056	24.08537	24.1332	24.1488	24.2535	NaN	23.76951
28.06599	27.97049	28.02433	28.13124	28.16997	28.07112	28.83244	28.69312	28.81726
NaN	24.58692	NaN	24.35783	23.81909	NaN	24.46139	24.30313	24.35783
NaN	NaN	24.65949	24.86703	24.79947	24.95837	25.09346	24.60964	24.95394
25.24627	25.97158	25.65949	25.53148	25.82881	25.92479	25.86231	25.89504	25.81421
NaN	24.16423	NaN	NaN	24.1946	NaN	25.26428	25.03171	24.85757
NaN	NaN	NaN	NaN	NaN	NaN	NaN	24.43619	24.18707
26.2021	25.19835	25.11744	24.71293	24.79947	24.84805	24.51051	24.37119	24.73376
24.75939	24.58692	24.66492	25.42982	25.08537	25.00624	24.5285	NaN	24.77957
NaN	NaN	NaN	NaN	NaN	NaN	24.03591	24.35111	23.96719
25.26785	25.97158	25.65949	25.67034	24.97158	26.20583	25.08537	25.11347	25.29263
NaN	NaN	NaN	23.6971	NaN	NaN	24.22435	25.04009	23.83846
27.5389	27.29789	27.58262	27.68646	27.49381	27.62506	27.65949	27.67303	28.14102
25.04427	24.391	24.95394	24.50446	24.06907	24.15653	25.54038	24.69179	25.00624
24.15653	24.45513	24.86703	24.33074	24.33074	24.42982	23.80931	24.48001	24.60964
27.97049	28.06085	27.78955	27.49381	27.61807	27.79576	27.98142	28.16039	27.81421
NaN	NaN	NaN	NaN	NaN	NaN	26.31181	26.317	26.14492
25.40406	25.88577	25.37451	25.19835	24.98033	25.66492	24.90885	24.74919	24.99335
27.78332	28.05051	27.72598	27.48616	27.24445	27.45513	27.82031	27.66628	27.73247
24.48001	24.09346	24.10949	25.16039	24.65949	25.72598	NaN	NaN	NaN
25.28912	25.41054	24.97158	25.41377	24.27498	25.31008	26.21697	26.07112	26.00624
22.69498	22.58463	NaN	22.98642	22.51653	NaN	22.92252	NaN	22.85186
24.15653	24.73892	24.26785	24.56964	23.2535	24.51051	26.46918	26.32045	26.46918
25.98685	25.7103	25.87643	26.44253	26.53594	26.58978	25.2021	25.42342	25.11347
23.55215	NaN	23.82881	23.56384	23.88577	23.77957	24.77957	25.15267	24.28207
NaN	NaN	NaN	25.81176	24.63201	25.54038	24.76446	NaN	NaN
26.82639	NaN	26.69975	26.29439	26.57398	26.75175	26.71293	27.35783	27.20769
25.54922	25.60116	25.37451	26.1546	25.89273	25.42662	25.94503	26.2553	26.86231
26.76446	24.97158	26.97596	27.63893	27.509	27.81421	27.95947	27.96499	28.06085
24.90885	24.82396	25.04843	24.54628	24.59263	24.43619	28.13614	28.65608	28.37865
NaN	NaN	24.51653	25.13712	25.39428	25.2535	NaN	NaN	NaN
28.68646	28.69975	28.90885	27.67303	27.48616	27.5969	27.53148	27.21697	27.34942
25.82881	25.35448	26.40568	27.09144	26.9428	26.95394	27.07112	27.70636	27.16039
26.98685	25.14492	27.11148	27.11148	26.80193	27.16997	24.51653	23.28207	24.31008
25.72858	25.30661	25.73117	26.56529	26.48462	27.07112	27.12139	27.13124	27.12139
26.67303	27.08132	26.51202	26.41377	26.68646	26.78955	25.85281	26.07521	26.1035
28.04009	27.41538	28.33671	27.00838	27.31527	27.19835	24.317	NaN	NaN
27.81421	27.76446	27.63893	26.50749	26.60399	26.89735	NaN	NaN	NaN
25.81665	26.30313	25.92931	25.95172	26.01052	26.00624	25.1757	25.14102	24.84805
27.29789	27.15074	27.2803	27.05051	26.85043	26.98685	NaN	NaN	NaN
25.16806	25.317	24.78955	24.87643	25.39428	24.8811	24.87643	25.46762	25.22066
28.69312	29.01372	28.82941	28.58262	28.68646	28.66289	28.08132	28.20769	27.98142
23.29614	NaN	NaN	NaN	NaN	NaN	26.2553	25.76951	26.28383
32.18719	31.90813	32.34625	31.8347	31.49667	31.70058	31.98482	32.0927	32.14832
27.78332	27.74535	27.93157	26.21141	26.64581	26.60399	24.98033	24.14102	24.67573
26.46607	26.95394	26.06497	25.83123	25.84566	25.84326	26.53445	26.42662	25.82881
29.8279	30.28692	29.91171	30.54812	30.11148	30.11148	29.87701	30.88285	30.14831
24.01903	24.54038	23.87643	24.53445	24.48001	24.63201	25.90196	25.30661	25.42982
25.47383	25.48922	25.21327	25.48308	25.46762	24.90426	25.78207	26.0296	26.04843
25.92705	25.77203	26.00838	26.56674	26.48616	26.391	24.64856	26.02327	25.54038
28.42742	28.29789	28.63201	28.78019	28.71947	28.90024	28.74855	28.66289	28.76446
27.35783	27.66628	27.3662	27.27142	27.16039	27.26249	27.2535	27.15074	27.19835
25.7103	25.97378	25.68378	25.68913	25.78706	25.75685	25.2571	25.391	25.46451
28.42342	28.31527	28.83545	28.38278	28.26249	28.30661	28.42742	28.42742	28.56819
29.05827	27.38278	29.13858	29.4919	29.4334	29.47267	29.15798	29.39305	29.25124
29.77392	29.91171	29.64238	30.2535	30.47267	30.28692	30.16039	30.18423	30.31959
24.31008	24.98902	25.48922	25.79947	24.69179	23.57542	25.04009	26.68646	24.2021
26.58978	26.89735	27.06085	27.46295	27.24445	27.32389	27.31527	27.32389	27.30661
25.97378	26.22986	25.67842	25.97815	25.74919	26.03171	25.05672	25.04843	25.18329
24.87643	25.04427	24.94503	24.42342	24.42342	24.64856	24.1946	NaN	24.14102
29.28472	29.57903	29.07623	28.69975	29.07112	28.86821	27.87994	28.23535	28.509
27.26249	27.20769	27.2803	26.05879	26.18707	26.40568	22.00196	NaN	NaN
24.70767	23.42982	24.95837	NaN	NaN	NaN	24.55215	25.15653	25.77957
25.82153	25.71293	25.72078	25.76446	26.0296	25.88577	25.93832	25.94503	25.92479
NaN	23.64306	23.82881	23.72858	23.22435	24.391	25.76699	25.01052	25.58405
26.03591	25.69445	26.18139	25.92931	26.05051	25.98251	24.12534	25.1055	24.72858
25.83364	26.26785	26.39591	26.23169	25.73892	25.58978	25.65676	25.317	25.79452
26.16423	26.3662	25.96279	26.40243	26.13712	25.97596	25.79947	25.57254	24.82881
24.1795	23.79947	24.5285	23.98468	24.31008	24.84326	24.53445	24.22435	24.18707
25.20583	24.95394	24.10149	25.43619	26.40406	NaN	27.76446	28.06599	27.77077
NaN	NaN	NaN	NaN	NaN	NaN	26.43301	25.57542	26.06291
NaN	NaN	NaN	NaN	NaN	NaN	NaN	NaN	NaN
23.66492	24.2535	23.45513	23.77957	22.81127	23.75939	25.03171	24.40406	24.15653
27.99226	28.27142	28.04009	28.15074	28.07623	28.23991	28.21697	28.09144	28.15557
28.40324	27.12139	28.20303	28.27142	27.97049	27.97049	26.72598	26.57542	26.87408
25.24264	25.33756	25.65949	25.02327	25.06085	25.1488	26.02115	26.60399	26.1055
NaN	25.98468	25.47073	25.68646	24.67034	NaN	26.01478	25.06907	26.0041
NaN	NaN	NaN	24.44253	24.417	NaN	24.91342	25.07316	25.05258
NaN	24.34435	24.52253	25.15267	25.31354	24.92252	26.33756	26.33074	25.82639
24.26069	NaN	23.80931	24.70767	24.67034	24.95837	25.15267	25.1055	25.08537
NaN	24.59263	24.5285	23.83846	23.86703	23.78955	24.59833	24.58118	24.20955
28.47846	28.67303	28.45905	28.77706	28.7357	28.58262	28.89157	28.84745	28.92309
25.87408	25.89735	26.01478	24.98468	25.6971	25.42021	24.80931	24.98468	24.62087

26.71293	26.42982	26.2803	26.51653	26.51653	26.96499	26.10749	26.04218	25.81176
24.28912	24.75939	24.63755	23.95837	24.33074	24.14102	24.35111	NaN	24.31008
26.038	NaN	26.13124	25.34096	25.56092	26.40568	22.01903	22.9968	23.44253
27.06085	27.52402	27.0296	26.52552	26.26607	26.15074	25.91342	26.24627	25.71555
25.55215	25.53445	25.37451	26.24989	25.34773	26.36286	26.35783	26.27675	25.78955
24.54628	25.27853	25.50749	25.37782	25.53445	25.8811	25.64031	26.61807	25.99766
26.60399	26.85043	26.81421	26.46762	26.54922	26.49076	26.47537	26.63201	26.73892
23.12376	23.86703	23.35111	22.60964	22.52373	NaN	26.13907	24.86231	24.94949
NaN	NaN	NaN	NaN	NaN	NaN	26.31354	26.16039	26.31181
24.93607	25.60399	25.19835	24.92252	25.15653	25.20583	25.89504	25.95837	25.96939
24.77455	24.55801	24.34435	25.16806	25.33074	25.317	24.74407	24.76446	24.65949
25.43619	25.61807	25.61526	25.04009	25.14102	25.54038	26.23352	26.24445	26.09949
23.85757	24.35783	23.74919	24.55215	23.81909	24.37782	23.96719	24.10949	24.28207
NaN	NaN	NaN	NaN	NaN	NaN	NaN	NaN	NaN
24.08537	24.00196	23.94949	24.2021	24.60964	24.239	23.57542	23.5285	24.41054
26.16615	25.98468	26.37451	26.64581	26.67303	26.78955	26.37451	26.78955	26.35448
27.01903	27.34096	27.14102	27.23535	27.37451	27.37451	25.76446	25.99335	26.23535
26.317	26.52999	26.48001	26.67303	26.30661	26.63201	25.55215	25.08537	25.1757
26.15653	24.58118	25.84566	25.42982	25.37119	25.41377	NaN	NaN	NaN
27.27142	27.73892	27.46295	26.54628	26.13907	26.26249	25.64581	25.22435	25.42342
30.77706	30.57542	30.91171	30.38278	30.26472	30.68813	28.84446	28.81115	28.86526
25.74919	25.78207	26.23717	27.22619	26.75175	26.85043	27.92592	27.41538	28.35363
NaN	NaN	NaN	NaN	NaN	NaN	26.07316	26.06085	25.85281
NaN	NaN	NaN	NaN	25.41054	24.38443	24.94949	24.44253	24.20955
23.65403	NaN	24.03591	23.57542	NaN	24.29614	24.55215	25.11744	24.38443
NaN	NaN	23.07398	24.317	23.50446	NaN	24.17188	24.30313	24.17188
23.98468	NaN	24.04427	25.12139	23.79947	24.56384	24.55215	24.79947	24.99335
26.73892	27.22619	26.58978	27.10149	27.04009	26.97596	27.54628	27.3662	27.26249
26.35111	26.06907	26.2535	26.88577	26.58978	27.0296	27.00838	26.80193	26.75175
NaN	25.89273	NaN	NaN	NaN	NaN	25.54922	25.87643	25.61246
25.37782	25.29964	25.37782	25.96279	25.94056	25.26428	25.44569	25.18707	25.22803
26.06497	25.72598	26.04427	25.84805	25.93832	25.48922	24.95394	NaN	25.68913
NaN	NaN	NaN	24.58118	24.65403	24.83364	25.79699	25.43301	25.60399
24.52253	25.02327	24.63755	25.36119	24.96719	24.99335	24.56384	24.44884	24.64306
25.72078	NaN	25.7103	26.03381	26.54775	26.09949	24.92705	25.48616	26.24264
24.2021	24.48616	24.26785	24.58118	25.57254	24.1332	24.28207	23.68646	24.38443
25.09748	25.03591	25.35783	25.417	25.03171	25.33074	25.42982	24.96279	25.32732
26.33074	26.38936	26.09346	26.32045	25.85519	26.12337	25.9428	25.34435	26.05051
24.94503	25.21327	24.82396	24.85757	24.22435	24.87643	25.06907	25.27498	25.12139
25.13712	25.16806	24.90426	25.82881	25.86938	26.00624	26.23535	25.51953	25.64306
24.41054	24.69179	24.391	25.76446	24.60399	24.94503	25.64031	26.03381	25.55215
25.37119	25.82881	25.79947	26.22986	26.20583	26.1055	26.73892	26.95394	26.85043
NaN	24.06907	23.84805	24.03591	23.83846	24.02749	NaN	24.29614	23.97596
24.417	24.54628	NaN	24.17188	23.97596	25.26428	23.83846	24.37119	23.58692
24.67573	24.50446	NaN	24.66492	24.84326	24.91798	26.01478	26.19648	25.6971
23.94056	23.88577	23.54038	23.91342	24.00196	24.16423	23.29614	24.00196	23.417
27.00838	27.16039	27.4073	27.64581	27.42342	27.68646	27.4073	27.54628	27.15074
27.16039	25.33074	26.00196	25.78706	26.43619	26.69975	25.30313	25.20583	25.81176
NaN	NaN	NaN	NaN	NaN	NaN	NaN	27.90311	NaN
27.67976	26.45356	27.71947	27.31527	27.12139	27.391	25.97158	27.34942	26.71293
24.7543	25.06907	24.01903	24.8811	24.28912	24.44253	24.27498	24.84326	24.79947
28.72273	26.82639	28.78644	28.96224	29.14589	29.22159	29.13858	29.09144	29.28251
27.04009	27.48616	27.26249	27.65949	27.80193	27.65267	26.93157	26.93157	26.61807
25.35448	26.71293	25.33756	26.22435	26.68646	26.13516	27.46295	27.28912	26.95394
25.96719	26.4073	25.94726	26.43778	26.14297	25.7103	26.04635	26.0041	26.06907
NaN	NaN	NaN	NaN	NaN	NaN	24.8811	24.89966	24.85757
23.87643	23.93157	23.95837	23.16116	23.79947	NaN	23.37782	23.46762	24.50446
27.42342	27.44727	27.31527	27.56819	27.80809	27.65267	26.57398	26.65949	26.71293
25.92931	26.20023	25.95615	25.70767	25.77957	25.85281	25.42021	25.72858	25.79699
26.72598	27.01903	26.95394	26.07316	26.61807	26.87408	26.82639	26.13712	26.31872
25.61807	25.85994	25.54628	26.15267	25.75685	25.67842	26.51202	26.46139	25.93607
28.45905	28.29351	28.80501	28.70306	28.73247	28.61807	28.37036	28.06599	28.21697
25.13712	25.08942	24.50446	26.11545	25.92705	25.76192	25.73892	25.68378	25.81421
27.14102	26.61807	27.38278	27.54628	27.56092	27.58978	27.28912	27.06085	26.9428
26.98685	27.19835	27.01903	26.45826	26.57542	27.05051	25.69445	25.78207	25.70504
26.04843	26.22435	26.02327	26.04009	26.35279	26.23535	22.65403	NaN	NaN
25.08942	25.02327	24.46139	24.83364	25.17188	24.97158	25.33756	25.22435	26.57109
NaN	29.44332	28.99496	28.85638	27.95394	28.94838	28.28912	28.39509	28.51653
NaN	NaN	23.5285	NaN	NaN	NaN	24.01903	24.01903	NaN
NaN	NaN	22.1673	NaN	NaN	NaN	NaN	NaN	NaN
24.59263	24.7024	24.7543	24.42982	24.90426	25.12534	25.13712	25.78706	25.89504
24.39754	25.63478	24.38443	NaN	24.18707	NaN	24.28207	24.80931	24.5285
NaN	24.42342	24.00196	23.97596	NaN	NaN	23.98468	24.34435	NaN
NaN	23.94056	NaN	NaN	NaN	24.37119	24.65403	NaN	NaN
24.94503	24.88577	25.38113	24.84805	24.89966	24.7543	24.27498	24.56964	24.46762
NaN	NaN	24.33074	NaN	NaN	25.47383	NaN	NaN	NaN
26.08335	26.23717	26.18707	24.92252	25.70504	25.73892	25.28912	25.12928	25.10149
24.89504	24.86231	24.6811	25.00624	23.95837	24.48001	NaN	NaN	NaN
26.06497	25.71816	25.9955	25.55801	25.51051	25.80193	25.60682	26.00624	25.37451
25.36786	25.78955	25.1488	24.91798	25.2535	24.92705	25.67303	25.46762	25.64581
25.06907	NaN	23.81909	23.89504	22.46762	22.77354	24.59263	24.34435	24.38443
25.86231	25.85043	25.6811	26.32389	25.93832	26.36453	24.73376	24.65403	24.86703
25.77957	26.35783	26.33586	26.77706	27.05051	26.82639	26.06291	26.57542	25.92025
24.54628	24.83846	24.96279	24.10149	24.1795	24.11744	23.94949	24.03591	24.46762
25.54038	25.21327	25.04427	24.98902	24.66492	25.15653	24.7024	23.29614	25.14492
23.06579	NaN	22.17035	25.22803	24.26069	23.96719	24.87173	24.26069	24.16423
27.1795	27.42342	27.22619	26.55947	26.61807	26.64581	NaN	NaN	NaN
24.96279	26.26964	24.72338	25.1795	25.10949	25.46451	24.391	24.30313	24.11744

27.33245	27.56092	27.28912	27.55362	27.74535	27.54628	27.97049	27.85043	27.85638
NaN	22.66926	NaN	22.75532	21.71816	NaN	23.15499	23.54038	22.25638
25.94949	25.91798	26.1946	25.93382	25.78207	26.15074	24.83846	24.26069	24.48616
24.55801	NaN	24.28207	24.64856	24.1332	24.99335	25.09346	24.72338	24.69179
25.07725	26.04009	25.74407	25.31008	25.02749	25.28207	25.1946	25.14492	24.94056
25.31008	25.48308	25.03591	25.18707	25.48001	25.43936	25.08132	24.74919	25.13712
23.88577	24.31008	24.17188	24.76446	24.54628	24.65949	25.47383	25.32389	25.4073
NaN	25.43301	25.34773	24.64856	24.391	24.56964	NaN	25.17188	24.34435
24.76446	25.47383	25.4008	24.77455	25.4008	25.36119	26.52253	26.30313	26.12337
23.81909	24.18707	23.18101	22.93337	23.37782	23.51653	24.06085	24.06907	23.85757
24.1795	NaN	24.72858	NaN	NaN	NaN	24.54628	24.86231	24.44253
NaN	23.68646	24.31008	24.44253	22.50203	23.57542	25.21327	25.59548	24.85757
23.89504	NaN	23.79947	23.89504	23.93157	24.1332	NaN	24.09346	24.16423
NaN	NaN	NaN	NaN	NaN	NaN	NaN	24.22435	23.93157
NaN	24.35783	23.65403	24.78457	24.79947	24.02749	23.87643	24.417	24.82396
NaN	25.239	24.01903	NaN	NaN	24.7543	24.1795	NaN	NaN
24.29614	24.14102	23.60964	24.50446	23.65403	25.26785	25.1055	24.97158	24.49228
24.71816	23.99335	23.82881	24.02749	24.31008	24.51051	25.45826	24.26785	24.04427
NaN	NaN	NaN	23.81909	23.48001	NaN	24.8811	24.56384	24.85757
NaN	NaN	NaN	NaN	24.47383	NaN	25.38772	25.1055	25.29263
24.94056	24.89966	25.22803	24.59833	24.89504	24.80931	24.05258	24.35111	24.29614
NaN	NaN	NaN	23.04093	NaN	23.49228	23.24191	NaN	NaN
24.78955	25.40406	25.36786	24.64856	25.21697	24.00196	NaN	NaN	NaN
23.42982	24.51653	24.06907	24.01052	NaN	NaN	24.65949	NaN	24.61526
26.58978	26.51653	27.64581	26.2803	26.04427	27.49381	26.60399	26.2732	27.41538
29.29789	28.52402	29.28692	29.78644	29.66628	29.54628	29.65267	29.53148	29.73085
25.62087	24.79947	24.35783	25.21697	25.02749	23.93157	25.64581	25.11347	25.29964
NaN	25.26069	NaN	24.82881	23.62087	NaN	24.91342	24.96719	25.08537
26.02538	25.62645	25.72858	26.2571	26.31181	26.27853	25.30313	26.1946	25.63201
NaN	26.391	24.94503	24.87643	NaN	25.27853	24.79947	25.09748	24.70767
24.01052	24.23169	23.91342	24.317	24.02749	24.44884	24.04427	24.38443	24.22435
23.51653	24.10149	22.85567	23.6971	23.63201	24.69179	23.75939	24.54628	23.33756
25.58405	NaN	24.82396	24.50446	24.8811	25.23169	24.6971	24.81909	24.2535
25.60964	26.30661	25.96499	25.21697	25.38772	25.60399	NaN	24.68646	NaN
NaN	NaN	NaN	NaN	NaN	NaN	25.14102	NaN	NaN
24.85757	25.27142	24.6811	25.66763	25.20583	25.27853	25.01478	25.26785	24.95837
NaN	NaN	NaN	NaN	NaN	NaN	23.65403	23.62087	23.15653
NaN	24.77957	24.37782	24.48616	25.01052	24.91798	NaN	NaN	NaN
23.88577	NaN	24.59263	23.97596	23.73892	23.64306	25.03591	25.31008	24.70767
24.239	24.317	24.05258	23.62087	23.79947	23.72858	23.45513	22.79352	23.32389
NaN	NaN	NaN	22.94949	NaN	NaN	NaN	NaN	NaN
24.00196	24.32389	24.04427	23.76951	24.1795	24.23169	24.37119	25.45199	23.84805
25.08132	25.1055	24.27498	23.6971	23.64306	24.26785	NaN	NaN	23.31008
25.54333	25.68913	25.64581	25.72858	25.35111	25.63201	25.50446	26.56384	NaN
23.6971	NaN	24.17188	23.72858	23.5285	23.93157	NaN	NaN	NaN
23.05424	23.46762	NaN	23.0592	22.85567	NaN	25.14492	24.84326	24.31008
23.98468	24.14102	23.06415	NaN	25.13712	23.5285	25.42662	24.43619	24.00196
24.21697	NaN	24.1795	NaN	NaN	NaN	NaN	24.6811	24.72338
23.91342	24.71816	24.11744	24.58692	24.11744	24.37119	NaN	23.82881	NaN
24.63201	24.54628	24.14102	24.52253	24.79452	24.77455	NaN	24.36453	NaN
27.07112	26.93157	27.52402	25.44569	25.55215	25.81176	23.75939	23.74919	23.92252
26.46295	26.75175	26.01265	25.88344	26.34942	26.16039	26.20583	26.21327	26.15267
25.00624	25.06497	24.66492	24.72858	25.10149	25.08537	NaN	NaN	NaN
NaN	NaN	NaN	25.07725	24.52253	25.49534	NaN	23.94056	24.51051
26.54775	26.56964	24.95394	25.80193	26.33074	26.01265	NaN	23.31008	23.62087
24.49838	24.65403	24.71293	25.76446	25.66492	25.91342	27.81421	27.71293	27.74535
26.24445	25.85519	25.47692	25.08132	25.63755	25.50446	25.68913	26.13712	25.8044
26.11148	26.39428	26.1546	25.84086	26.01903	26.33756	26.78955	26.56674	26.65949
NaN	NaN	NaN	24.65403	23.50446	24.20955	25.10149	24.85281	25.24989
29.34942	29.28692	29.02697	28.65608	28.08639	29.14102	29.07877	29.00571	28.06085
24.80931	25.10149	24.73892	24.74919	25.02749	24.99335	24.99335	24.64856	24.73892
27.70636	27.43141	27.86821	27.75812	27.83846	28.05569	28.05569	28.12633	28.13614
26.88577	23.65403	27.20769	25.08942	27.43936	27.13124	25.417	26.73892	24.97158
NaN	24.65403	24.86703	25.48308	25.98251	25.76446	24.68646	24.85757	24.76951
24.85281	30.04791	24.81909	24.89504	24.91798	24.98902	24.83364	24.1795	24.99335
NaN	24.72338	24.88577	23.95837	23.84805	24.00196	27.08132	26.47692	27.0296
24.98902	25.18707	24.85281	25.1055	24.62645	24.94503	25.31354	24.95837	25.18329
NaN	NaN	NaN	NaN	NaN	NaN	24.34435	24.239	NaN
25.55801	25.96499	24.94949	25.68378	25.48308	26.05672	25.27142	25.37782	25.07725
25.54333	25.63478	25.12928	25.29964	25.39754	24.81421	25.56092	25.65403	25.50749
25.03171	25.01903	25.03591	25.33415	25.19084	25.09748	24.70767	24.42342	24.26069
27.19835	26.97596	26.86231	26.76446	26.60399	26.71293	25.53445	25.57542	24.92252
24.82396	24.71816	24.28912	24.72858	24.63755	24.35783	25.48001	25.37119	25.22066
24.09346	NaN	NaN	24.28207	23.62087	23.84805	24.97596	25.04843	25.13712
25.32732	25.66492	25.2535	24.47383	23.82881	24.32389	27.3662	27.2803	27.2803
23.62087	24.16423	23.82881	24.26785	24.26069	24.18707	26.05879	25.91114	25.81665
NaN	NaN	NaN	NaN	NaN	NaN	25.05258	24.74407	25.37451
27.34942	27.22619	27.391	26.69975	26.90885	27.07112	26.63201	26.46139	26.55069
27.49381	27.20769	27.52402	26.92025	26.85043	26.82639	27.53148	27.54628	27.35783
26.20583	26.33415	26.03381	NaN	26.60399	26.52253	24.41054	23.6971	23.92252
24.79947	24.31008	24.35111	24.69179	24.05258	23.99335	23.48001	23.71816	23.07071
22.94235	NaN	23.01052	23.08699	NaN	22.59605	23.03423	22.86325	22.89319
27.34942	27.35783	27.39917	27.01903	26.92025	26.92025	26.82639	26.81421	26.81421
24.60964	NaN	24.35111	24.63201	24.74407	24.24627	24.12534	23.86703	NaN
26.53594	25.84086	26.32732	26.63201	26.21141	25.9157	26.64581	26.29614	26.4346
25.18329	25.26785	25.57542	24.34435	23.99335	24.239	NaN	NaN	NaN
25.64031	25.88809	25.57254	25.43301	25.51953	25.54333	24.71293	25.37782	25.29614
26.20583	26.07112	26.417	25.58978	25.60399	25.2535	25.34773	24.7024	25.10149

23.66492	23.46762	23.76951	NaN	NaN	22.95305	24.57542	24.32389	23.94056
26.63201	26.5389	26.60399	26.87408	27.30661	27.3662	26.2589	26.76446	26.239
25.84326	24.81421	24.20955	NaN	24.391	NaN	24.74919	24.89966	24.91798
26.17379	26.57542	26.08942	26.3662	25.89273	25.71816	26.77706	26.37617	26.34266
27.88577	28.07623	28.15074	28.05569	27.90311	28.01372	27.56819	27.45513	27.53148
27.55362	27.82639	27.56092	27.29789	27.38278	27.22619	26.90885	27.12139	26.97596
25.11744	25.91798	25.59263	25.12139	25.48308	25.35111	26.61807	26.9428	26.81421
24.74919	25.16806	24.1795	25.02749	25.07316	24.94949	24.64856	24.77455	24.79452
24.11744	24.90885	23.89504	24.44884	25.33415	23.31008	24.94056	25.76699	24.59833
24.98902	24.88577	25.32045	25.64581	24.95394	25.51953	24.57542	25.07316	24.78457
27.49381	27.509	27.3662	27.9428	28.01372	28.01372	27.79576	27.73892	27.90885
23.33756	24.66492	24.06085	24.69179	24.66492	25.18707	22.54981	22.23023	22.82687
25.41377	25.86703	25.37782	24.94056	24.94949	25.02327	NaN	NaN	23.15345
24.74919	24.95837	24.83364	24.40406	24.79452	24.37782	24.36453	25.01478	24.86703
24.16423	24.27498	23.29614	23.72858	23.42982	23.68646	23.32389	23.84805	24.41054
25.29614	25.24264	24.75939	24.76951	24.93607	24.58118	24.63755	24.77957	24.28207
25.79947	25.91342	25.87173	26.2571	26.27675	26.49228	26.82639	26.41054	26.88577
22.35111	22.26213	22.79154	NaN	NaN	22.44	22.52373	23.0592	22.81127
26.10149	26.71293	26.09748	26.73892	26.69975	26.1488	26.13712	26.44095	26.19272
24.2021	24.23169	22.96719	23.73892	23.90426	23.31008	24.391	24.06085	24.55215
22.42726	NaN	22.04593	22.73273	NaN	22.51171	22.83461	22.21845	NaN
29.42542	29.56456	29.40324	28.70636	28.45905	28.45905	28.22159	28.28912	28.29351
25.22066	25.00196	25.20955	24.83846	24.01903	24.93157	25.04427	NaN	NaN
24.6811	24.86703	24.82396	24.5285	24.42342	24.07725	NaN	24.97596	23.73892
23.84805	24.14102	23.87643	23.73892	23.88577	23.75939	24.17188	24.2535	24.51653
24.74407	24.54628	24.34435	24.86703	24.90885	24.65403	24.88577	24.36453	24.46139
23.86703	23.87643	24.10149	24.02749	24.391	23.95837	NaN	24.42342	23.64306
NaN	NaN	NaN	NaN	NaN	NaN	24.71816	24.65403	24.52253
24.28912	24.06907	23.417	24.00196	23.89504	23.79947	NaN	NaN	NaN
24.81909	NaN	24.08537	25.62645	24.63201	25.21697	NaN	24.86231	NaN
25.92479	26.39754	25.67573	26.00624	25.91798	25.24264	26.00624	26.53742	25.89041
26.43301	26.73892	26.33756	26.11942	25.7415	26.038	NaN	NaN	NaN
24.40406	24.27498	24.55801	24.98033	24.85281	24.46762	NaN	NaN	NaN
26.34096	25.62645	26.11942	26.05672	25.83364	25.5285	24.59833	25.2535	25.34096
25.90885	25.76192	25.87877	25.44884	25.27853	25.22435	24.94056	25.12534	24.69179
25.24627	25.56964	25.42021	25.239	25.12534	25.43936	24.64856	24.43619	24.40406
23.11744	22.42214	NaN	23.59833	23.70767	24.24627	NaN	NaN	NaN
NaN	28.04531	24.2021	25.81909	25.92705	26.30487	25.32045	30.00838	25.2571
23.96719	24.317	24.24627	24.70767	24.5285	24.08537	NaN	NaN	23.32389
24.95837	NaN	23.78955	25.34773	24.44884	24.14102	25.32389	25.03171	25.44569
23.44253	NaN	NaN	NaN	NaN	NaN	23.40406	NaN	23.50446
24.81909	24.95837	24.63201	24.2535	23.91342	24.84326	24.05258	24.29614	24.33756
NaN	NaN	23.46762	NaN	23.33756	23.03591	23.32389	24.48616	23.92252
26.47383	26.69975	26.15653	25.79452	25.34096	25.51953	24.63755	NaN	NaN
24.51051	25.2856	24.95837	24.29614	25.00624	24.87173	24.98468	24.05258	24.74919
27.58262	27.19835	27.83846	27.89735	27.81421	28.01903	27.04009	26.92025	27.11148
28.92592	28.78644	28.85638	28.88867	28.57903	28.73892	27.77706	27.58978	27.61105
26.01478	26.33074	25.85994	26.23169	26.11744	26.15074	26.78955	26.81421	27.04009
NaN	NaN	NaN	NaN	NaN	NaN	24.60399	24.69179	24.84805
27.79576	27.90311	27.87994	27.58978	27.45513	27.52402	26.40568	25.84086	26.18329
22.72025	23.49228	NaN	NaN	23.68646	23.20658	24.07725	NaN	23.78955
25.64856	25.97815	25.9428	25.45199	NaN	25.31008	NaN	NaN	25.43619
NaN	23.35111	23.15499	23.70767	23.12849	23.21845	23.87643	NaN	23.46762
27.04009	26.64581	27.0296	26.40406	26.26964	26.33586	25.16039	25.34435	25.23535
26.65949	27.00838	26.57398	26.60399	26.68646	26.71293	26.33245	26.27853	26.30835
23.94056	NaN	NaN	22.94592	23.67573	24.54628	27.58978	26.98685	27.53148
NaN	23.1031	NaN	NaN	NaN	23.45513	25.53742	24.8044	25.65949
26.61807	25.20955	26.11347	25.73892	25.66221	25.62923	25.65949	NaN	NaN
25.51051	25.60116	25.47383	26.06291	25.99981	26.13712	25.52253	25.98902	25.85757
24.92705	24.50446	25.28912	24.37119	25.1332	24.05258	23.92252	24.00196	24.03591
NaN	23.70767	23.62087	23.94949	23.81909	23.42982	23.93157	23.66492	23.89504
NaN	NaN	NaN	NaN	NaN	NaN	24.22435	23.96719	23.05258
25.06907	NaN	24.75939	24.81909	NaN	25.37782	NaN	NaN	NaN
23.83846	NaN	NaN	24.01052	24.26785	24.21697	NaN	NaN	NaN
NaN	NaN	NaN	23.49228	22.81127	22.40925	NaN	NaN	23.15808
NaN	24.29614	24.417	24.89041	24.72338	24.18707	24.391	25.05258	25.1055
24.89504	24.47383	24.65949	25.69975	25.42021	24.99335	24.74919	24.72858	24.60399
25.56674	25.07316	25.54922	25.77203	25.7103	25.66221	24.20955	23.72858	24.09346
25.77957	26.06702	25.7024	25.1946	24.89966	24.66492	24.05258	25.12928	24.1332
24.03591	26.09144	24.06907	24.07725	24.04427	24.12534	25.48922	24.85757	25.61526
25.16806	25.73635	25.32389	25.58405	25.59548	25.52552	25.67842	25.52552	25.94056
24.52253	24.08537	24.39754	24.01052	23.48001	23.66492	23.6971	24.86703	24.24627
24.26069	23.98468	23.6971	24.57542	24.97596	24.31008	22.53564	23.391	NaN
25.83846	25.69445	25.70504	25.41054	25.20583	25.51352	23.65403	NaN	NaN
24.94949	24.71816	24.62645	24.76446	24.5285	24.68646	24.47383	24.85757	23.91342
25.83846	25.51051	25.46139	25.47383	25.46139	25.78207	NaN	NaN	NaN
25.61526	26.05466	25.85757	25.59548	25.63755	26.05258	NaN	NaN	NaN
26.00838	26.04218	25.95394	26.29614	26.28207	25.80193	24.46762	24.93607	25.03591
26.05879	NaN	26.38443	26.2589	26.11942	26.22986	24.92705	25.02749	25.14102
24.92705	25.04843	24.71293	24.47383	24.50446	24.46762	24.83364	24.55801	24.6811
26.09346	24.63201	24.7543	24.317	25.87408	25.16806	25.57831	25.59833	25.83123
25.33415	25.55801	25.79699	25.18707	25.29614	25.52253	23.86703	25.68913	25.02749
24.43619	24.66492	NaN	25.55801	25.75685	24.55215	26.64581	27.3662	26.60399
NaN	24.43619	NaN	24.7543	NaN	NaN	24.48616	24.82396	24.32389
22.52373	NaN	22.9566	22.45513	22.68646	23.00024	23.97596	24.33756	23.48001
25.96279	26.64581	25.05258	25.34096	25.44884	25.45826	24.56964	24.1946	23.88577
23.59833	23.96719	24.21697	23.33756	23.62087	23.35111	24.42342	24.52253	24.239
NaN	NaN	NaN	21.2535	22.70345	NaN	23.88577	23.08537	NaN

24.6971	25.05672	23.91342	24.74407	24.33074	25.26069	26.33245	26.38278	26.38607
24.53445	24.64306	24.56384	24.98033	24.7543	24.74919	24.89041	24.51051	24.87643
26.21512	26.15267	26.06702	26.13124	26.28912	26.417	NaN	25.80931	25.50446
25.56092	25.66221	25.6513	26.52103	26.60399	26.33586	27.38278	27.00838	27.22619
25.24627	NaN	25.39754	25.75685	25.52253	25.59548	26.35616	26.47692	26.63201
24.98902	25.20583	25.57542	26.42502	26.14492	26.4008	26.2589	26.37617	26.71293
27.69312	27.79576	27.65949	27.94838	28.06085	27.80193	27.71293	27.20769	27.63893
27.43936	27.14102	27.50142	27.83846	28.04009	27.92025	28.37036	28.20769	28.59335
26.15267	26.46139	26.19272	26.19835	26.06702	26.2589	26.55508	26.5285	26.20396
24.65403	25.33756	24.64856	24.83364	25.33756	24.91342	24.52253	25.04843	24.66492
26.1946	26.09346	26.2535	26.10149	25.92931	25.94726	26.12731	26.39754	27.2803
26.54922	26.01265	26.87408	25.82153	25.12534	26.038	25.62366	25.54628	25.36119
NaN	25.18707	24.79452	25.50446	25.62923	25.11347	25.29964	24.79452	26.0041
NaN	NaN	NaN	23.83846	NaN	NaN	25.74407	26.08132	26.00838
25.88809	25.98468	25.87643	25.39428	25.62923	25.317	25.68646	25.391	25.34435
25.78457	24.89041	25.99118	26.99766	27.16997	27.28912	28.71947	28.57903	28.97049
23.63201	NaN	24.06907	23.62087	24.1795	23.67573	24.26785	23.45513	23.28207
26.27498	26.88577	26.65949	27.12139	27.19835	26.98685	26.03381	26.0296	26.24627
24.87643	25.19084	25.09748	25.15267	25.317	25.48308	24.89966	24.89041	25.1332
26.36119	27.11148	27.13124	26.93157	26.56819	26.60399	23.11744	NaN	22.95305
24.28912	23.65403	23.92252	24.04427	24.18707	24.39754	24.21697	24.42982	24.317
27.82639	28.00838	27.96499	28.24898	28.47073	28.29351	27.71293	27.80193	28.21234
24.57542	25.27142	24.09346	25.10949	24.94949	24.82396	25.12928	25.38113	24.99766
NaN	NaN	NaN	NaN	NaN	NaN	25.10149	24.99766	24.97158
25.1055	24.51653	24.83364	25.01052	25.11744	24.85757	24.96279	24.99335	24.92252
24.27498	NaN	24.60399	24.6971	24.45513	24.49228	25.85757	25.2021	24.85757
25.78457	25.85281	25.32732	25.05672	25.42982	25.27498	25.62366	25.78955	25.50142
24.5285	24.1488	23.89504	24.28207	23.65403	23.62087	24.47383	24.00196	24.28207
24.7024	24.49228	24.2021	25.1055	24.58118	24.317	24.60399	NaN	24.55215
26.37285	26.06702	26.15074	26.4567	26.15653	26.63201	25.56384	25.95615	25.68913
23.28207	24.6811	24.50446	24.21697	24.391	NaN	NaN	NaN	NaN
26.60399	26.21141	26.1776	25.72078	25.31008	26.1946	26.61807	25.97378	25.77957
25.04843	NaN	24.76446	25.29263	25.68378	25.239	26.36286	26.35616	26.35951
24.92705	25.4008	24.96719	24.93157	24.94503	25.46762	23.84805	24.27498	23.72858
24.35783	24.28207	24.239	23.96719	24.15653	24.42982	24.42342	24.49228	23.83846
27.56092	28.15074	27.80193	27.18896	27.1795	27.05051	NaN	NaN	NaN
NaN	NaN	NaN	25.45826	24.38443	24.31008	NaN	NaN	23.24771
NaN	NaN	24.77455	24.98468	25.08942	25.26428	25.52552	24.1332	23.68646
25.86703	25.76699	26.1055	25.7415	25.95394	25.6971	25.56964	25.79947	25.45513
23.74919	25.1795	24.28912	24.01903	24.42342	25.14102	24.72338	24.81421	24.70767
26.57254	26.34266	26.60399	26.86231	26.47073	26.61807	26.04009	26.45199	26.22066
25.30661	24.78955	25.2856	25.38113	24.68646	25.67573	26.13124	25.03171	25.46139
25.76951	26.26607	26.00838	25.76446	25.56964	25.86703	24.66492	24.58118	24.5285
29.29351	29.258	29.59156	29.04531	28.82941	28.56092	28.09144	28.22159	28.57181
24.96279	25.44884	25.04843	25.56964	25.67034	25.6811	25.66492	25.73635	25.49228
24.43619	24.6971	23.96719	24.37119	24.45513	24.50446	24.10949	NaN	24.17188
24.23169	24.24627	24.14102	24.44253	24.40406	24.391	24.58118	24.46139	24.62645
24.91342	24.37782	NaN	24.51653	24.31008	24.6971	25.1488	25.317	25.23169
25.1332	25.2571	25.89504	25.02327	25.89966	25.91798	25.72858	25.50446	25.86467
28.42742	28.51277	28.4746	28.2535	28.22159	28.23991	29.94	29.95394	29.99496
26.72598	26.80193	26.47537	25.69975	25.92931	25.69179	NaN	NaN	NaN
26.42021	26.46918	26.80193	26.07112	26.47537	26.18707	26.26428	26.48155	26.46139
26.36119	26.82639	26.67303	27.10149	27.04009	27.20769	26.1795	25.80686	26.2535
27.66628	27.98142	27.69312	28.10649	28.32817	28.37451	28.23535	28.07623	27.97049
25.86938	26.35783	26.12139	26.01052	26.02749	25.9157	25.73635	25.6811	25.80931
23.94056	24.10949	24.2535	24.35783	25.04009	24.239	24.417	24.85281	23.91342
NaN	NaN	NaN	NaN	NaN	NaN	NaN	NaN	NaN
22.17646	20.5545	NaN	23.56384	23.18404	22.11426	23.22729	23.60964	22.41957
28.16039	28.23535	28.52776	28.42742	28.36202	28.46684	NaN	NaN	NaN
25.1757	25.62366	25.16423	24.95837	24.54038	24.35783	23.54038	NaN	NaN
24.58692	24.87643	24.6811	24.2021	24.10949	24.46139	23.80931	22.85186	24.04427
23.76951	24.10949	23.83846	24.1488	23.73892	24.39754	23.96719	24.41054	23.99335
24.51653	24.65403	24.74407	24.48001	24.70767	24.98033	23.68646	23.74919	23.93157
25.16423	24.96719	25.08942	24.40406	25.317	25.00196	24.41054	24.15653	24.5285
NaN	NaN	22.48493	23.40406	NaN	NaN	NaN	NaN	23.51653
24.82396	24.317	24.76446	24.417	24.64306	24.63201	23.77957	23.72858	23.28207
22.69922	21.93876	22.45764	22.66058	NaN	22.41183	NaN	NaN	22.20658
NaN	NaN	NaN	NaN	NaN	NaN	24.83364	24.52253	25.59263
27.37451	27.49381	27.24445	26.65949	26.98685	27.08132	24.65403	25.2535	24.65949
NaN	NaN	23.78955	23.73892	23.49228	23.62087	23.98468	24.239	24.12534
22.90242	23.07398	23.14414	22.97246	23.19761	23.64306	22.34299	22.48738	22.23608
25.04009	24.76446	24.58692	24.43619	24.85757	24.37782	NaN	NaN	NaN
23.65403	23.71816	NaN	NaN	23.06415	NaN	23.33756	23.1673	23.1379
22.13634	22.1795	21.62756	22.2535	22.78955	21.83461	23.0559	22.61863	22.58003
24.65403	25.27142	24.71293	25.78955	25.6811	25.39754	23.82881	24.22435	24.33756
23.76951	NaN	23.98468	23.26785	24.27498	24.1795	23.83846	23.88577	23.79947
NaN	NaN	NaN	NaN	NaN	NaN	23.37782	NaN	23.96719
NaN	NaN	NaN	23.10149	23.2535	NaN	24.42982	25.09346	25.26069
25.51653	24.93607	25.55801	25.70767	25.28207	25.78207	25.69445	25.23169	25.19835
23.29614	23.74919	23.07071	22.83653	23.03758	22.67142	24.00196	24.02749	23.89504
25.66492	25.66221	26.46918	26.64581	26.69975	26.83846	26.12337	24.35783	25.9955
22.92071	23.17035	22.74098	23.35111	22.99162	23.31008	NaN	NaN	20.14569
24.49838	24.46139	24.60399	24.72858	NaN	25.38113	25.37119	25.71293	25.61526
24.63755	22.82687	24.317	NaN	24.56384	24.2021	24.26785	24.26069	24.1946
24.29614	24.56964	24.66492	24.2535	24.35111	24.46762	23.68646	23.88577	24.03591
25.2535	NaN	24.98468	NaN	NaN	NaN	NaN	NaN	NaN
NaN	NaN	24.23169	24.06907	23.79947	NaN	NaN	NaN	NaN
25.23535	26.98685	25.64856	25.32732	25.08942	24.69179	24.88577	25.04843	24.80931

25.96719	26.19272	25.71816	25.55801	25.86467	25.60399	26.75175	26.75175	26.48769
28.52776	24.35783	28.391	27.32389	25.99118	27.27142	28.01372	28.60399	27.4073
24.82396	24.23169	25.7543	24.01052	NaN	23.79947	NaN	NaN	NaN
NaN	NaN	21.82299	NaN	NaN	NaN	25.239	25.08132	24.92252
NaN	NaN	22.95305	NaN	NaN	NaN	24.01903	23.96719	24.06085
24.57542	24.72858	24.1488	24.51653	24.49838	24.55215	24.99766	24.8044	24.76446
23.66492	24.06907	24.00196	24.1795	24.47383	25.24627	24.18707	24.67034	24.48616
26.06085	26.1757	26.82639	26.85043	26.24264	26.51953	25.81176	25.96719	25.42021
NaN	NaN	NaN	NaN	NaN	NaN	24.39754	24.55801	NaN
NaN	NaN	NaN	NaN	NaN	NaN	23.77957	23.54038	23.83846
NaN	NaN	NaN	NaN	NaN	NaN	NaN	NaN	NaN
22.9812	24.07725	24.1488	24.72338	22.79352	NaN	23.12534	24.62645	NaN
NaN	22.83074	NaN	24.79947	22.93876	NaN	21.79947	22.98815	NaN
24.63201	24.46762	24.391	23.98468	23.0559	24.54628	22.38837	24.239	24.00196
NaN	NaN	NaN	NaN	NaN	NaN	25.50749	25.4073	25.36119
NaN	21.97071	22.35111	NaN	NaN	NaN	22.74919	NaN	23.96719
24.1488	24.67573	23.68646	23.85757	24.54038	23.98468	NaN	NaN	24.07725
NaN	NaN	NaN	23.46762	23.84805	23.65403	23.83846	22.92252	24.36453
NaN	NaN	25.80686	26.29964	26.18707	NaN	NaN	25.34096	26.4346
23.0426	23.75939	23.54038	23.95837	24.14102	23.23169	24.28912	24.85757	24.04427
NaN	NaN	NaN	22.71398	NaN	NaN	NaN	NaN	NaN
24.57542	24.31008	24.10949	24.317	24.18707	24.33756	25.27142	25.87643	25.39428
NaN	NaN	NaN	24.05258	24.80931	27.0296	25.16039	24.18707	23.36453
NaN	NaN	25.36786	NaN	NaN	NaN	NaN	NaN	NaN
24.33756	25.2535	24.14102	24.46139	24.417	24.39754	23.93157	23.66492	24.46762
23.78955	24.93607	24.62645	25.02327	24.52253	24.42342	NaN	24.90885	23.26785
27.69975	26.98685	27.32389	27.39917	27.13124	27.47073	27.31527	27.37451	27.46295
25.47692	25.48922	25.26785	25.76446	25.41054	25.32045	23.94949	24.96719	25.33415
22.48493	22.79947	22.239	22.23316	22.18858	NaN	22.67573	22.82493	22.97771
23.85757	24.15653	23.73892	23.98468	24.15653	24.49228	24.97596	24.1488	24.17188
26.21141	25.86703	26.69975	26.11347	26.88577	26.51202	26.2732	26.47228	25.92252
23.55215	NaN	NaN	NaN	NaN	23.08861	NaN	23.35111	23.86703
NaN	24.38443	24.16423	24.15653	24.10949	24.06085	NaN	NaN	NaN
24.35783	24.5285	24.48616	24.29614	24.417	24.77957	23.68646	NaN	23.28207
27.24445	27.0296	27.35783	26.93157	26.52552	26.98685	NaN	NaN	NaN
24.64856	NaN	24.83846	24.58118	24.92252	24.67573	24.84805	25.51352	24.90426
NaN	22.81714	23.22582	22.84805	NaN	NaN	NaN	NaN	22.39885
25.81176	26.29614	25.04843	25.63478	25.82396	26.25169	25.95837	25.68913	25.95172
24.69179	23.94056	25.36786	24.29614	23.66492	25.56964	NaN	23.6971	23.07887
NaN	NaN	NaN	NaN	NaN	NaN	23.81909	23.80931	23.93157
26.1757	26.11744	26.02115	26.39264	26.50597	25.91342	25.73635	25.91798	26.61807
24.60399	NaN	24.48616	24.23169	24.43619	24.45513	25.08132	24.85757	24.45513
26.77706	27.3662	26.82639	26.48308	26.52552	26.75175	26.4186	26.3662	26.0874
26.60399	25.98685	25.86231	26.28207	26.27675	26.05879	26.11744	25.87173	25.82881
NaN	25.35111	NaN	23.66492	24.37782	23.32389	NaN	24.59263	23.28207
25.91798	24.48001	25.95172	25.98033	25.78457	26.22619	25.65676	25.26069	25.62645
23.93157	24.2021	NaN	24.42982	24.55801	27.13124	24.6971	24.67034	24.21697
23.78955	24.38443	24.54038	25.81665	24.2021	25.1946	24.48616	NaN	24.6811
23.55215	24.02749	NaN	23.76951	23.20508	23.86703	NaN	23.81909	NaN
22.92615	NaN	NaN	23.46762	24.64306	23.5285	24.06085	22.70345	NaN
NaN	23.17798	23.31008	23.24336	23.42982	23.23608	22.86136	NaN	22.97246
NaN	NaN	NaN	NaN	NaN	NaN	NaN	24.54038	NaN
23.97596	NaN	NaN	NaN	23.90426	23.87643	NaN	NaN	NaN
NaN	23.90426	24.50446	23.81909	24.59833	24.77957	24.16423	24.96279	24.68646
23.92252	NaN	24.2021	24.00196	24.06907	NaN	24.05258	NaN	23.94056
25.16039	24.58118	25.31008	23.83846	23.58692	23.65403	24.23169	23.95837	24.49838
NaN	NaN	NaN	NaN	NaN	NaN	NaN	NaN	NaN
24.91798	25.42342	25.04843	25.239	25.00624	25.11744	25.29964	25.417	25.14102
NaN	NaN	NaN	23.87643	24.47383	24.06907	NaN	NaN	NaN
25.27853	25.36786	24.77455	24.36453	24.48616	23.86703	24.45513	NaN	NaN
23.49228	22.35917	24.30313	23.86703	NaN	23.88577	25.33756	25.19084	24.93157
24.80931	22.49228	NaN	25.06497	25.27853	25.35783	25.23169	25.43936	24.87173
22.44758	NaN	21.60513	21.6231	NaN	21.18858	NaN	NaN	NaN
23.76951	24.41054	23.58692	24.04427	24.12534	23.80931	24.06907	24.98902	24.59263
22.80931	23.77957	22.81714	24.43619	NaN	NaN	22.84613	22.68859	22.9812
23.37782	24.2535	23.32389	23.94056	NaN	24.08537	23.48001	23.31008	23.12218
24.00196	23.80931	24.12534	23.1488	24.05258	24.1946	25.14102	24.94503	24.73376
24.11744	24.48001	23.58692	23.89504	23.82881	24.28207	23.66492	22.23023	23.09828
26.86231	26.82639	26.96499	26.51202	26.54628	26.42021	26.67303	26.50597	26.98685
24.79452	24.90885	24.48001	25.23535	25.23169	25.42982	24.77455	25.10949	24.81421
26.45199	26.22986	26.48308	26.41377	26.20769	26.60399	25.35783	25.11347	25.7415
24.28207	24.73376	25.68646	24.95837	24.91342	24.98033	23.37782	25.18707	24.55215
NaN	NaN	NaN	22.95305	24.10949	24.29614	NaN	NaN	NaN
24.63755	24.83846	24.31008	24.49228	24.41054	24.37782	24.7024	24.37782	24.46762
22.49717	22.94235	21.79947	23.26785	22.66492	22.95127	22.5708	23.1931	23.19009
23.6971	23.97596	23.36453	23.91342	23.82881	23.90426	24.24627	23.92252	24.53445
25.17188	25.391	25.37782	24.8811	25.08942	24.76446	26.15074	25.69445	25.81909
24.27498	24.40406	24.92705	23.6971	23.77957	23.64306	NaN	NaN	NaN
24.24627	24.26785	23.92252	24.01903	24.2021	23.58692	23.64306	23.55215	23.70767
23.29614	23.58692	22.95482	23.48001	23.56384	23.56384	NaN	NaN	NaN
24.20955	24.43619	24.33074	24.17188	24.68646	24.5285	NaN	23.391	23.33756
22.32389	22.29894	NaN	22.16423	22.63423	22.35917	22.36185	22.49228	NaN
24.71816	24.92705	24.24627	24.88577	24.22435	25.00624	NaN	NaN	NaN
26.71293	26.81421	26.82639	26.27675	26.09748	26.29614	NaN	NaN	NaN
24.7024	24.74919	24.75939	25.33756	25.00624	24.89504	24.66492	NaN	24.5285
27.9428	28.15557	28.32817	27.98685	27.61807	28.12633	28.10149	28.19366	28.06599
26.11347	25.68378	26.44253	24.56964	25.51653	24.64306	26.72598	27.1795	27.2535
NaN	NaN	NaN	NaN	NaN	NaN	26.26249	26.64581	26.42822

NaN	NaN	NaN	NaN	NaN	NaN	23.94056	23.78955	NaN
NaN	NaN	NaN	NaN	NaN	NaN	NaN	NaN	NaN
22.92796	23.26785	22.27923	23.59833	23.32389	23.417	NaN	23.46762	23.94949
24.11744	24.08537	24.40406	24.08537	24.47383	23.89504	23.45513	24.06085	23.73892
30.27586	29.91171	30.47267	30.54812	30.40324	29.61807	30.44332	30.70471	30.39305
22.59149	22.49473	22.63201	22.52133	21.87643	22.28771	NaN	NaN	NaN
NaN	NaN	NaN	23.21401	NaN	NaN	NaN	NaN	NaN
25.391	25.93382	25.76951	26.11545	25.61526	25.87173	24.28912	24.86231	24.239
25.7415	26.01478	25.34435	24.99766	25.05258	25.37782	25.91342	25.16806	25.83123
25.89735	25.89041	25.99981	27.01903	26.24082	26.61807	26.78955	26.71293	26.9428
26.06907	26.15074	26.34266	26.35616	26.50142	26.4073	26.61807	26.77706	27.21697
22.74098	22.83461	23.01563	23.29614	22.85377	23.68646	23.36453	23.40406	22.59377
23.88577	NaN	23.77957	22.63644	23.88577	23.73892	23.77957	24.20955	23.20955
26.17188	25.97815	26.4073	24.82396	24.92252	24.33756	26.55362	26.44411	26.76446
26.07725	25.87643	25.90196	26.68646	26.38607	26.46295	26.87408	26.67303	26.82639
NaN	25.4073	24.33756	24.73892	NaN	24.18707	24.81909	25.47383	NaN
24.89966	25.28912	24.82881	25.39428	25.27142	25.65403	25.54333	25.57254	25.36786
25.13712	25.43936	25.49228	24.84326	24.60399	24.87173	25.11744	25.85043	25.02327
26.10149	26.1546	26.09346	25.67034	25.65403	25.98468	24.60964	25.59833	24.7024
25.92931	26.07928	25.68913	26.87408	26.46607	26.58978	26.24082	26.05051	26.51352
24.52253	25.27498	24.91798	24.77957	24.51051	24.93157	24.87643	25.2856	24.87173
23.24627	NaN	NaN	22.53564	22.88948	23.20658	NaN	NaN	23.57542
27.58262	25.26785	27.22619	27.43141	27.47846	27.47846	27.29789	27.16039	27.19835
25.33756	24.71293	25.391	24.85757	24.98033	25.42982	25.72338	25.78457	25.87643
23.44253	23.91342	23.5285	23.48001	23.31008	23.81909	24.52253	24.46139	24.59833
26.47692	24.88577	26.52552	26.61807	26.24808	26.30661	25.61526	25.62923	25.27142
27.89157	27.5389	27.63893	27.2803	27.44727	27.48616	27.13124	27.38278	26.97596
NaN	NaN	23.48001	NaN	NaN	23.88577	NaN	23.65403	24.11744
24.03591	24.6811	24.10149	24.00196	24.09346	23.75939	24.28207	24.239	24.33756
26.69975	26.43301	26.58978	26.40243	26.57109	26.82639	25.94949	26.4999	26.30138
26.71293	27.28912	26.85043	26.60399	26.80193	27.12139	26.10949	26.00196	25.94726
29.86673	29.0531	29.53705	29.82487	30.36202	30.14831	30.52028	30.36202	30.42342
24.1332	24.22435	23.84805	24.24627	24.26785	24.31008	24.33756	24.63201	24.37782
26.71293	27.97049	26.75175	26.65949	26.43619	26.88577	27.33245	26.64581	26.90885
NaN	NaN	NaN	24.06907	23.81909	24.14102	24.41054	24.8811	24.46762
25.27498	25.8044	25.89735	24.72858	24.73892	24.66492	24.43619	24.72858	24.24627
27.01903	27.31527	26.81421	26.80193	26.80193	26.67303	26.75175	26.69975	26.86231
25.90196	25.77706	25.6513	25.98251	25.83123	25.79947	24.47383	NaN	24.35783
26.17188	26.63201	26.06497	27.09144	26.9428	26.92025	27.16997	26.81421	26.98685
NaN	NaN	NaN	22.19761	NaN	NaN	NaN	23.67573	22.77957
23.07235	NaN	25.11744	24.11744	24.46762	NaN	NaN	NaN	NaN
NaN	23.75939	23.94056	23.54038	NaN	NaN	24.20955	23.60964	23.99335
NaN	NaN	21.8839	22.9812	22.95305	23.56384	23.96719	23.56384	23.86703
24.391	24.417	24.09346	23.98468	23.78955	23.98468	24.53445	23.73892	23.26785
25.06907	25.38772	25.47073	24.62087	24.85281	25.50749	24.42342	24.14102	24.42342
NaN	23.89504	24.239	23.76951	23.97596	NaN	25.32732	NaN	26.37451
NaN	NaN	NaN	NaN	NaN	21.80735	NaN	NaN	NaN
24.43619	NaN	24.60399	24.1488	24.26069	23.91342	NaN	NaN	NaN
24.05258	24.51051	24.70767	23.93157	24.07725	24.22435	NaN	24.39754	24.46139
24.78955	24.49228	NaN	NaN	NaN	23.79947	NaN	NaN	23.13946
23.82881	NaN	23.95837	24.07725	24.1488	23.85757	24.08537	24.07725	24.35111
NaN	23.42982	23.0476	23.24627	23.40406	23.54038	22.9566	NaN	22.80144
23.46762	NaN	23.391	23.55215	NaN	23.68646	24.1332	NaN	NaN
25.38443	25.19835	24.1488	24.65949	24.52253	25.08132	NaN	NaN	NaN
23.01563	22.68432	21.43746	23.46762	23.12534	22.99335	22.09828	22.50446	22.34028
NaN	NaN	NaN	23.2535	23.71816	23.417	24.04427	24.07725	23.77957
24.96279	25.48001	25.73117	25.52253	25.51051	25.56674	NaN	NaN	NaN
NaN	NaN	NaN	NaN	NaN	NaN	24.92252	25.33074	25.13712
24.90885	24.71293	24.60399	24.74407	24.239	23.96719	25.33756	25.2571	25.02749
25.35783	25.31008	25.49534	25.60682	25.84566	25.79947	25.47692	25.49534	25.78706
NaN	NaN	23.49228	NaN	23.66492	NaN	NaN	NaN	NaN
23.91342	NaN	23.71816	23.81909	24.08537	24.44884	23.45513	23.32389	NaN
23.46762	NaN	23.417	23.391	23.45513	23.5285	NaN	23.82881	23.65403
25.72078	24.317	NaN	24.06907	25.05258	24.54038	23.0592	24.30313	23.29614
24.7024	24.64306	24.60399	24.75939	24.96279	24.1488	23.88577	23.84805	24.00196
24.74919	NaN	24.32389	NaN	23.37782	23.94056	NaN	23.06579	NaN
23.29614	NaN	23.46762	22.96367	23.63201	23.48001	22.97771	23.29614	NaN
27.23535	26.77706	27.32389	27.08132	26.83846	26.78955	25.64581	25.43619	25.42662
NaN	NaN	NaN	24.34435	25.19084	NaN	24.45513	25.47383	24.83846
22.81127	NaN	23.01733	22.17646	23.1206	22.95482	22.97071	24.1795	23.21549
NaN	NaN	NaN	22.82104	NaN	22.76142	24.49228	24.33074	24.26069
25.23169	24.43619	26.07316	24.28207	NaN	NaN	NaN	24.83846	24.09346
NaN	NaN	22.68432	22.74714	NaN	23.33756	NaN	NaN	22.90793
22.86136	NaN	NaN	NaN	NaN	NaN	NaN	NaN	NaN
22.10789	22.23608	24.73892	24.39754	24.10149	24.57542	26.2571	27.15074	25.96939
26.52402	26.98685	27.21697	26.40568	26.81421	26.9428	26.54922	27.23535	26.77706
30.23078	30.33031	30.2535	29.34942	29.12139	29.32603	28.16997	28.14102	27.54628
24.05258	24.51051	24.54038	23.80931	NaN	NaN	NaN	NaN	NaN
23.58692	23.75939	23.00539	23.83846	23.93157	23.79947	NaN	22.76344	22.47507
27.0296	29.71129	26.36286	27.46295	26.92025	27.14102	27.11148	26.99766	27.19835
24.84326	24.20955	NaN	24.23169	NaN	NaN	25.04009	24.91342	24.79452
23.31008	23.72858	23.23754	23.83846	23.49228	23.42982	23.48001	23.59833	23.67573
28.37036	NaN	28.29351	26.44884	26.57542	NaN	26.35951	26.46139	NaN
NaN	NaN	NaN	NaN	NaN	NaN	25.71555	24.76446	25.37451
24.51653	24.93607	24.48616	24.56964	24.1795	24.6971	23.71816	23.07398	23.40406
23.37782	23.86703	22.89134	22.92615	NaN	23.1673	NaN	NaN	NaN
24.6811	25.22803	25.09748	25.23535	25.35783	25.31354	25.85519	25.78706	25.88344
25.46451	25.92479	25.39754	25.78706	25.92025	25.1795	26.55069	26.68646	25.87173

24.91342	NaN	24.1488	23.92252	24.12534	24.59263	NaN	NaN	24.26069
22.36453	NaN	22.04593	NaN	NaN	23.16576	NaN	NaN	NaN
NaN	24.21697	NaN	NaN	25.96719	NaN	26.82639	25.81665	26.17188
25.33415	23.80931	24.53445	24.56964	24.44253	25.04843	23.59833	NaN	NaN
23.86703	24.09346	23.40406	23.50446	23.68646	23.09184	24.2021	23.90426	24.93607
NaN	NaN	21.52133	21.84613	NaN	NaN	23.12534	NaN	22.98989
25.80686	25.75685	26.18139	25.73635	25.44884	25.60682	25.48308	25.53445	25.63755
NaN	24.85757	NaN	23.84805	23.83846	23.6971	NaN	NaN	NaN
NaN	NaN	NaN	NaN	NaN	NaN	24.16423	24.98468	NaN
23.71816	23.75939	23.95837	23.76951	24.10149	24.1946	24.74919	24.98902	24.72338
NaN	NaN	NaN	NaN	24.32389	24.46762	25.53148	25.45513	25.65949
NaN	23.84805	NaN	23.98468	24.47383	24.76446	22.97421	23.391	23.64306
24.48616	24.51051	24.42982	24.34435	23.391	24.317	23.97596	24.05258	24.39754
23.97596	24.03591	23.2006	24.00196	24.17188	22.82299	24.1946	23.73892	24.68646
22.6584	23.33756	22.39885	23.42982	22.74919	22.88762	NaN	NaN	23.32389
22.95127	23.78955	23.63201	24.64856	23.65403	23.59833	22.53801	23.36453	23.49228
NaN	NaN	NaN	NaN	NaN	NaN	24.7543	25.14492	24.69179
NaN	24.63755	NaN	24.35783	24.79452	NaN	24.97596	25.00624	25.19835
NaN	NaN	NaN	NaN	NaN	NaN	25.73635	26.76446	25.71293
NaN	NaN	NaN	NaN	23.46762	NaN	23.64306	23.68646	23.66492
NaN	NaN	NaN	21.47754	NaN	23.82881	23.50446	23.12849	22.77354
24.1946	24.80931	24.09346	23.97596	23.77957	23.6971	22.37518	22.79749	23.59833
23.99335	24.46762	24.14102	24.36453	24.59263	24.47383	24.67573	24.48616	24.73376
24.5285	24.86231	24.48001	24.8811	24.82396	24.94503	24.35111	24.37119	24.76951
25.38443	24.52253	25.40406	25.43936	25.60399	NaN	25.87173	26.16039	26.57254
22.47754	21.79551	22.58921	NaN	NaN	22.73892	NaN	NaN	NaN
28.37036	NaN	28.91741	NaN	NaN	28.55362	29.31743	28.48999	28.81115
24.84805	24.5285	24.93607	23.96719	24.32389	24.22435	23.75939	23.60964	24.33074
23.05258	23.74919	22.94949	23.49228	23.89504	24.04427	23.09507	22.42982	23.26785
NaN	NaN	NaN	NaN	NaN	NaN	23.66492	23.36453	22.98815
NaN	24.24627	23.24771	24.31008	24.20955	24.38443	23.49228	23.86703	24.06085
23.26785	22.87455	23.2006	23.44253	23.391	23.1961	NaN	NaN	NaN
25.92025	23.97596	25.66763	25.87643	26.46295	26.38772	25.87408	25.98251	26.06497
NaN	NaN	NaN	23.50446	23.24916	24.18707	23.28207	24.01903	23.55215
NaN	NaN	NaN	NaN	NaN	NaN	NaN	27.94838	27.99226
NaN	NaN	NaN	NaN	NaN	NaN	23.2535	NaN	NaN
NaN	24.14102	24.1946	24.11744	24.45513	24.15653	24.65403	24.91342	24.42342
23.99335	24.23169	24.14102	22.88017	23.40406	23.37782	NaN	23.99335	23.44253
NaN	NaN	NaN	22.39624	22.239	NaN	23.05755	22.92615	22.78157
24.78955	24.61526	24.77455	24.8044	24.2535	24.52253	25.24989	25.30661	25.16039
26.53148	27.37451	25.58118	25.37782	NaN	24.94056	25.21697	25.06497	24.93607
24.8811	25.38113	26.24627	24.33074	24.2535	NaN	NaN	NaN	NaN
NaN	25.4008	NaN	24.96279	24.87173	NaN	NaN	NaN	NaN
25.76699	NaN	25.26069	NaN	25.50446	NaN	25.04009	25.8044	25.32045
25.63201	25.32389	24.56384	25.30313	26.09144	25.39428	26.52552	26.71293	26.81421
25.27142	25.44569	25.12928	24.86231	25.04427	25.1488	24.51051	24.83846	24.49838
23.98468	25.08942	24.6971	24.04427	24.64306	24.85757	24.07725	24.391	24.06085
24.44884	23.83846	24.33756	25.2535	25.02749	25.2535	25.15653	24.73376	25.41377
24.04427	23.6971	24.00196	23.37782	23.07071	23.09184	NaN	NaN	NaN
26.39917	26.1776	25.94503	25.76699	25.80931	26.01478	26.89735	26.57109	26.65949
22.74714	NaN	23.50446	23.70767	23.65403	23.6971	23.89504	23.79947	23.6971
NaN	NaN	NaN	NaN	NaN	NaN	NaN	NaN	NaN
23.66492	24.60399	24.16423	24.71293	23.90426	24.10149	23.19761	24.62087	23.18101
22.58463	NaN	22.59605	NaN	23.51653	23.26785	23.91342	23.74919	23.73892
24.78955	24.8044	24.26069	24.71816	24.33756	24.92705	24.98468	NaN	24.7024
23.88577	24.09346	24.10949	23.12218	24.32389	NaN	25.69179	24.49228	25.00624
26.04843	NaN	26.4186	26.88577	26.27498	26.20023	26.05258	26.038	25.7543
25.88577	25.89735	25.67573	26.35111	25.56964	25.73117	24.87173	25.16039	24.85757
23.417	23.54038	23.417	23.21104	23.51653	23.44253	23.75939	23.57542	23.50446
NaN	26.26428	NaN	25.72338	26.1488	NaN	26.02327	26.08942	26.40406
24.84805	25.16039	25.71816	25.02749	25.27498	25.42662	25.22066	24.49228	24.50446
NaN	NaN	NaN	23.46762	22.89319	NaN	23.87643	24.39754	24.56964
24.64306	24.45513	24.39754	24.18707	23.70767	24.03591	24.08537	24.07725	23.81909
24.79947	24.97158	25.08537	25.1488	25.27853	25.1332	25.19084	25.60116	25.15653
24.49228	NaN	24.12534	24.49838	25.49534	24.73892	25.60399	25.19084	25.68646
26.07521	25.73376	26.23169	26.12928	25.83364	26.15267	26.35448	25.93382	26.24627
24.54038	25.19084	24.89966	24.18707	24.00196	24.01903	24.56964	24.52253	24.2021
21.76344	22.1047	21.9061	22.64306	22.1916	22.90242	22.60738	NaN	22.72442
25.01478	24.88577	26.12139	25.93157	25.66221	25.29263	NaN	NaN	NaN
NaN	NaN	NaN	NaN	NaN	NaN	24.88577	NaN	24.391
24.51051	25.04843	24.91342	24.37119	24.62087	24.49228	24.91798	24.89966	24.71816
23.5285	23.45513	NaN	22.20658	NaN	23.92252	24.39754	25.16423	24.89041
24.45513	NaN	24.34435	NaN	NaN	NaN	25.67034	25.82396	25.89504
25.03591	24.96279	24.94056	25.32732	25.36453	25.16806	24.20955	24.74407	24.51653
NaN	24.68646	24.98902	25.04009	25.417	25.42982	25.34435	25.20583	25.62645
23.58692	23.2535	NaN	24.1946	23.57542	24.18707	25.77455	25.61526	26.29263
NaN	NaN	23.25205	NaN	NaN	NaN	24.239	24.71293	24.06085
22.98989	NaN	23.45513	23.77957	NaN	21.76344	NaN	23.73892	NaN
24.47383	24.18707	24.74407	24.83364	25.38113	25.18707	24.24627	24.15653	24.21697
28.06085	27.78955	27.57542	27.93719	28.11644	27.71947	26.34773	26.73892	26.33756
24.95394	24.95837	24.87643	24.78457	24.95837	24.98033	24.79452	25.24989	25.18707
27.3662	NaN	27.35783	26.81421	26.39264	27.09144	26.80193	27.92025	26.57542
25.76446	24.40406	25.60682	26.35111	26.82639	26.81421	27.00838	26.9428	27.27142
25.14492	24.98902	25.22066	25.09346	25.1332	25.29263	24.21697	23.57542	23.50446
NaN	26.58978	NaN	25.14102	25.2856	25.27142	25.92705	25.48001	25.61807
25.93832	26.87408	26.04218	26.48769	26.67303	26.26069	25.85281	25.92252	25.94949
NaN	NaN	NaN	NaN	NaN	NaN	24.1946	NaN	NaN
23.20359	23.28207	NaN	23.84805	24.63755	24.18707	23.75939	23.391	22.98294

NaN	NaN	23.29614	24.15653	24.06907	24.39754	24.65403	24.42342	24.04427
25.33074	24.95837	25.10149	25.87877	25.92479	26.10149	26.31181	26.24264	25.98033
24.96279	25.76951	25.63201	23.93157	23.99335	24.72338	23.29614	23.32389	23.24916
24.95837	25.32389	25.11744	24.83364	24.6971	24.85281	24.44884	24.2535	24.84805
25.08942	24.75939	24.51653	24.27498	24.417	24.44253	25.85757	25.43936	25.9157
NaN	NaN	NaN	NaN	NaN	NaN	24.37119	NaN	24.54038
24.07725	23.94949	24.34435	25.62366	23.84805	NaN	24.32389	24.23169	NaN
26.07725	25.46139	26.15074	25.87877	25.92705	25.95837	25.09748	25.27498	25.08132
24.33756	NaN	23.5285	23.391	NaN	23.81909	NaN	NaN	NaN
25.98685	25.06085	25.82881	25.65676	25.65676	25.82396	NaN	NaN	NaN
23.68646	NaN	24.239	24.29614	24.1488	24.20955	24.15653	23.97596	24.58118
25.01052	25.06907	25.19084	25.54333	25.19835	24.72338	25.07725	25.04843	25.12139
23.74919	23.93157	23.0625	22.42726	NaN	23.2021	24.56964	26.49534	23.75939
23.81909	23.36453	23.49228	23.35111	22.76547	22.80931	NaN	NaN	NaN
NaN	NaN	NaN	NaN	NaN	NaN	24.49838	24.55801	24.50446
NaN	24.14102	NaN	NaN	23.82881	23.94949	25.01903	25.26428	24.75939
23.37782	NaN	23.77957	24.1488	23.97596	24.37119	23.75939	NaN	NaN
25.73117	NaN	25.81665	25.18329	25.11744	25.06907	24.40406	24.94949	24.96279
23.87643	24.20955	23.86703	24.61526	23.95837	24.49228	24.56384	24.34435	24.52253
22.239	24.44253	24.239	24.62087	24.14102	23.94056	24.83846	24.40406	25.29964
23.76951	23.89504	23.0258	24.317	NaN	24.29614	NaN	22.12376	NaN
24.95394	24.91342	25.24264	25.20955	25.19835	24.81909	25.93382	25.15267	25.29614
24.39754	24.83364	23.81909	NaN	24.18707	23.07561	24.74407	24.76446	24.60964
NaN	23.78955	NaN	NaN	NaN	NaN	NaN	NaN	23.88577
NaN	NaN	23.28207	NaN	NaN	NaN	24.1332	NaN	22.97071
24.63201	24.91798	24.55801	24.98902	24.42342	24.84326	24.37782	24.05258	24.1332
NaN	NaN	NaN	NaN	NaN	NaN	NaN	NaN	NaN
NaN	NaN	NaN	NaN	NaN	23.55215	23.93157	23.81909	23.80931
23.81909	24.91342	24.2021	23.88577	24.98033	25.1488	23.95837	23.75939	NaN
24.63755	NaN	24.51051	24.57542	24.8044	24.85757	23.21697	23.08861	23.35111
NaN	NaN	NaN	NaN	NaN	24.35783	NaN	NaN	NaN
24.65403	25.1946	24.82396	25.54922	25.03171	25.2856	NaN	NaN	NaN
25.38113	24.8811	25.28207	24.6811	22.68432	24.03591	24.6811	25.27142	24.68646
NaN	NaN	NaN	20.70556	NaN	NaN	NaN	24.10149	22.66492
22.31562	NaN	22.40145	21.89134	NaN	23.13006	NaN	22.54038	22.6584
NaN	NaN	23.71816	NaN	NaN	NaN	NaN	NaN	NaN
26.98685	27.09144	26.76446	26.72598	26.55508	26.75175	27.06085	27.13124	27.1795
23.15808	NaN	NaN	NaN	NaN	NaN	26.16423	22.39362	23.28207
NaN	NaN	NaN	NaN	NaN	NaN	22.33211	NaN	NaN
NaN	23.32389	23.391	23.417	23.13946	23.59833	24.317	23.78955	23.63201
NaN	NaN	NaN	NaN	NaN	22.93157	NaN	22.27923	NaN
23.89504	NaN	23.55215	23.35111	23.83846	NaN	23.81909	NaN	NaN
NaN	NaN	22.67788	NaN	NaN	23.6971	NaN	NaN	NaN
24.34435	NaN	24.6971	24.6971	24.33074	24.98033	24.06085	NaN	24.17188
22.61414	23.42982	23.10789	23.63201	23.55215	23.28207	24.16423	23.89504	24.12534
NaN	23.22582	NaN	NaN	NaN	NaN	NaN	NaN	NaN
NaN	NaN	NaN	NaN	NaN	24.34435	NaN	NaN	NaN
23.2535	23.49228	23.28207	22.9812	22.97946	23.5285	NaN	NaN	NaN
NaN	23.73892	NaN	23.82881	NaN	NaN	NaN	NaN	NaN
NaN	23.391	NaN	23.49228	24.06085	23.36453	23.91342	22.43746	NaN
NaN	NaN	NaN	NaN	NaN	NaN	NaN	NaN	NaN
NaN	NaN	23.19009	NaN	23.11585	NaN	NaN	23.71816	23.75939
22.46513	22.41442	NaN	22.80931	23.03255	22.92976	22.67357	23.59833	23.23316
24.7543	NaN	24.40406	24.1946	24.07725	NaN	NaN	NaN	24.76951
NaN	NaN	NaN	21.95305	NaN	23.33756	23.32389	22.91159	NaN
24.56964	NaN	24.79452	NaN	NaN	25.13712	25.58118	25.55215	25.24989
23.49228	23.1931	NaN	23.57542	22.94592	24.28207	23.59833	23.62087	23.45513
26.39917	27.07112	26.63201	26.78955	26.72598	26.73892	26.239	26.29439	26.13907
25.22803	24.89504	NaN	24.76446	24.86231	25.21697	NaN	NaN	24.06085
NaN	NaN	NaN	NaN	23.33756	NaN	NaN	22.92976	22.73066
22.80735	NaN	23.04926	NaN	NaN	23.02411	NaN	22.58921	22.99852
25.42982	25.12534	25.18329	24.66492	24.54038	24.74919	NaN	NaN	NaN
NaN	NaN	NaN	23.57542	23.73892	23.33756	22.75124	NaN	21.89874
23.58692	NaN	23.5285	24.1946	23.66492	23.51653	24.15653	23.54038	24.09346
NaN	NaN	NaN	NaN	NaN	NaN	NaN	NaN	NaN
21.81127	NaN	20.95305	22.97421	22.13946	22.33756	NaN	NaN	NaN
22.96543	23.2214	23.391	22.49228	22.36453	23.44253	NaN	NaN	NaN
25.97815	24.62645	24.65403	24.2535	24.50446	24.22435	24.67034	26.35279	25.20955
23.92252	23.82881	23.417	23.85757	23.85757	24.60399	23.29614	NaN	NaN
NaN	NaN	NaN	NaN	NaN	23.72858	NaN	NaN	NaN
21.90242	22.87643	22.94414	22.94056	23.04093	22.87455	22.28489	23.33756	22.45513
NaN	NaN	NaN	NaN	NaN	NaN	25.14102	NaN	NaN
24.20955	NaN	24.03591	24.34435	24.12534	24.05258	24.6811	24.97158	24.64306
24.21697	23.85757	23.49228	23.5285	NaN	23.17798	22.74919	22.00539	NaN
23.26785	24.94503	24.86231	23.75939	23.76951	NaN	24.03591	24.16423	24.2021
24.94056	NaN	24.74919	24.80931	24.82396	25.02749	23.88577	23.07235	23.73892
23.94949	NaN	23.63201	23.40406	23.84805	23.80931	23.64306	23.46762	23.60964
NaN	24.26069	NaN	23.28207	23.1991	23.24045	22.63423	22.70978	22.77555
23.33756	23.45513	23.46762	23.60964	23.76951	23.48001	23.60964	24.15653	23.64306
NaN	NaN	NaN	23.20508	23.93157	NaN	23.66492	23.5285	22.47011
24.87173	25.24264	25.5285	27.45513	26.76446	25.56674	25.73376	25.10949	26.49686
27.56819	26.73892	26.89735	26.71293	26.81421	26.61807	27.30661	26.97596	27.06085
23.23169	24.06907	23.40406	NaN	NaN	NaN	NaN	NaN	NaN
23.74919	24.56964	24.35111	23.94056	23.86703	23.98468	23.391	NaN	22.32937
22.63423	23.14414	NaN	22.40145	NaN	23.31008	22.3538	NaN	22.05258
21.6971	24.84805	22.49717	25.1488	25.65676	24.10149	24.45513	26.34435	25.81176
NaN	NaN	NaN	NaN	NaN	23.63201	NaN	NaN	NaN
23.20359	NaN	23.40406	23.79947	23.31008	NaN	NaN	NaN	23.15499

NaN	23.51653	NaN	23.1673	23.28207	NaN	22.2764	23.44253	22.68217
26.89735	27.04009	26.89735	26.43141	26.31527	26.54185	26.90885	25.97158	26.54185
NaN	NaN	NaN	NaN	NaN	22.7348	22.98468	NaN	22.64306
22.32937	NaN	22.00539	21.90976	21.89134	21.68859	21.9566	NaN	22.61863
26.92025	24.7543	25.82639	25.97158	25.99981	25.60399	26.01478	25.62366	25.6811
24.09346	NaN	NaN	NaN	NaN	NaN	21.30452	21.23608	NaN
28.24898	28.5389	27.86231	28.13614	28.26696	28.2803	27.0296	27.61105	27.90311
NaN	22.74919	22.26785	22.20658	22.71188	22.60964	NaN	NaN	NaN
25.01903	25.54333	24.95837	24.60399	24.72338	24.34435	23.87643	24.26069	24.35783
NaN	22.69498	23.03591	22.59149	22.64086	22.72234	NaN	NaN	NaN
27.85043	28.3662	26.14492	NaN	NaN	26.63201	26.80193	28.16039	26.23535
NaN	NaN	21.27071	NaN	22.51893	22.82493	23.09507	22.76142	NaN
24.31008	NaN	24.7024	24.96279	24.92705	25.16039	24.75939	25.06497	25.34773
21.89134	NaN	22.3672	22.0426	NaN	22.26499	22.27355	NaN	22.3538
NaN	NaN	NaN	NaN	NaN	NaN	22.70345	23.12692	22.63865
23.48001	NaN	23.28207	23.6971	23.48001	23.57542	23.31008	23.84805	22.98989
NaN	24.391	NaN	25.06497	25.01478	24.98902	23.71816	24.45513	24.02749
NaN	NaN	NaN	NaN	NaN	NaN	23.96719	23.77957	23.90426
24.16423	22.24771	24.89966	24.42982	24.71816	24.23169	25.02749	25.64306	25.09748
23.74919	NaN	NaN	NaN	24.10949	23.91342	23.82881	23.2535	23.66492
23.74919	23.81909	23.417	23.90426	23.51653	23.71816	23.65403	23.33756	23.40406
NaN	NaN	23.45513	NaN	NaN	NaN	NaN	NaN	23.75939
NaN	NaN	NaN	NaN	NaN	NaN	24.35111	25.80931	24.90426
NaN	NaN	NaN	NaN	NaN	NaN	NaN	NaN	NaN
NaN	23.33756	24.46139	23.50446	23.73892	23.44253	24.06907	23.86703	NaN
22.20658	22.27355	22.1673	22.14258	22.18253	22.34841	22.67573	22.32937	22.1673
NaN	NaN	NaN	NaN	NaN	NaN	NaN	NaN	NaN
23.97596	23.93157	NaN	23.42982	23.44253	23.48001	24.22435	23.71816	23.76951
NaN	NaN	NaN	NaN	NaN	NaN	23.88577	23.85757	23.86703
23.6971	24.11744	23.95837	23.98468	24.49228	24.28912	22.58921	22.64746	NaN
23.42982	23.71816	22.87079	23.48001	23.33756	23.62087	24.62645	24.90426	25.34435
23.87643	23.63201	22.89504	23.55215	23.59833	23.391	23.56384	NaN	24.1795
22.46014	NaN	NaN	NaN	NaN	NaN	24.05258	24.28912	24.36453
23.82881	23.64306	23.32389	23.90426	23.58692	24.20955	22.37252	24.46762	23.14569
NaN	NaN	NaN	NaN	NaN	NaN	23.11744	23.33756	23.33756
26.20023	25.78706	25.88809	26.24082	25.69445	27.46295	26.38278	26.96499	26.93157
24.98033	25.56964	24.83364	25.29964	25.2535	25.54333	24.57542	24.79947	25.14102
22.35649	NaN	21.84995	21.97071	21.36986	21.9061	NaN	NaN	21.00881
NaN	29.52776	28.81726	28.99766	28.96499	28.391	28.98685	28.78955	28.73247
24.2535	NaN	24.60964	25.28912	24.54628	25.4008	NaN	24.2021	NaN
NaN	NaN	NaN	NaN	NaN	NaN	NaN	NaN	28.57542
23.0592	23.31008	23.04593	22.88577	NaN	23.37782	22.76142	23.18101	NaN
NaN	NaN	NaN	NaN	NaN	NaN	NaN	NaN	22.68217
25.38443	25.93607	25.2856	25.27142	25.26785	25.19835	25.70767	25.49838	25.51051
NaN	NaN	NaN	NaN	22.80144	NaN	NaN	NaN	23.09184
24.44884	24.64856	24.26785	24.61526	24.81421	24.28912	24.77455	24.55215	24.46762
NaN	22.30452	23.17493	23.42982	22.85186	22.73686	23.49228	23.86703	23.09989
24.37119	24.7543	24.65403	24.85757	25.06085	25.11347	NaN	NaN	NaN
23.36453	NaN	23.391	23.88577	23.66492	23.82881	23.2535	NaN	23.08213
NaN	NaN	NaN	NaN	NaN	22.73686	24.01903	23.6971	23.76951
NaN	21.24191	NaN	21.6584	NaN	NaN	21.73066	22.1916	22.1916
NaN	23.58692	23.417	23.76951	22.56848	NaN	22.91707	NaN	NaN
NaN	NaN	NaN	NaN	NaN	NaN	22.66058	22.98468	23.36453
25.46451	25.62087	25.00196	25.48001	24.97596	25.26428	24.46762	24.20955	24.417
NaN	NaN	NaN	NaN	NaN	NaN	22.1916	NaN	24.09346
25.92025	NaN	22.88017	25.13712	NaN	NaN	26.96499	25.47383	26.60399
NaN	NaN	NaN	NaN	NaN	NaN	NaN	NaN	NaN
NaN	NaN	NaN	NaN	NaN	NaN	NaN	NaN	NaN
NaN	NaN	NaN	NaN	NaN	NaN	NaN	NaN	NaN
NaN	NaN	24.8044	23.76951	NaN	NaN	NaN	NaN	NaN
25.72338	24.97158	25.24627	24.68646	24.77455	25.08942	24.30313	24.01052	24.54038
25.27853	25.24989	25.08132	24.94056	24.96279	24.97596	25.34096	25.30313	25.45513
24.06085	NaN	24.08537	25.09346	24.98033	24.58118	NaN	24.64306	25.33756
NaN	NaN	NaN	NaN	NaN	NaN	NaN	26.72598	NaN
24.26785	24.60399	24.5285	24.33074	24.33756	24.32389	24.8811	24.87173	25.00624
NaN	NaN	NaN	NaN	NaN	NaN	NaN	22.67788	22.04926
23.94949	NaN	NaN	NaN	NaN	NaN	22.22729	23.92252	NaN
23.73892	23.84805	23.78955	24.28912	23.75939	23.36453	24.98033	24.90885	24.73892
NaN	NaN	NaN	NaN	NaN	NaN	NaN	NaN	NaN
24.2021	NaN	24.22435	24.93157	24.1488	24.59263	23.72858	NaN	NaN
24.33756	NaN	24.68646	NaN	24.62645	25.90885	NaN	24.06085	NaN
23.04926	23.17493	23.2535	23.26785	22.92976	23.239	NaN	NaN	NaN
NaN	NaN	NaN	NaN	NaN	NaN	NaN	24.31008	23.6971
NaN	NaN	NaN	NaN	NaN	NaN	NaN	NaN	NaN
NaN	NaN	NaN	NaN	NaN	NaN	NaN	NaN	NaN
NaN	NaN	NaN	NaN	22.99335	22.54274	23.68646	23.35111	24.01903
23.65403	24.02749	23.75939	23.55215	23.40406	23.26785	24.93157	25.08537	25.07725
NaN	NaN	NaN	24.73376	NaN	NaN	NaN	NaN	NaN
NaN	NaN	NaN	22.39362	22.31562	22.5285	22.97421	NaN	22.91889
NaN	NaN	NaN	22.83653	22.50203	22.77555	23.23608	23.14569	23.18101
NaN	NaN	NaN	NaN	NaN	NaN	NaN	NaN	NaN
NaN	NaN	24.01903	NaN	23.35111	22.93517	NaN	NaN	NaN
25.239	25.53148	25.42982	26.14686	25.92025	25.96279	26.30661	26.45199	26.06702
NaN	NaN	NaN	22.29894	NaN	21.86891	21.68003	21.38047	NaN
25.91342	25.98685	25.77957	25.54922	25.68646	25.73635	24.18707	23.391	24.06085
27.51653	27.12139	27.30661	27.2535	27.12139	26.67303	27.05051	27.05051	27.39917
25.80193	25.71555	NaN	25.76951	25.6971	26.26249	25.54038	25.42021	23.90426
22.85946	23.07887	22.58463	22.87267	22.8783	NaN	22.51653	22.28489	NaN

25.72078	25.83364	25.99766	25.50142	25.95394	25.92025	26.1035	26.01052	25.77706
24.89504	24.54038	24.33756	24.45513	24.87173	24.10149	25.58118	24.99766	25.24627
24.06907	NaN	24.35783	NaN	23.417	NaN	NaN	23.12849	22.34571
23.50446	23.36453	23.40406	23.391	22.88762	22.96543	NaN	NaN	NaN
NaN	NaN	NaN	NaN	24.96719	24.87643	24.93157	24.78955	25.26069
NaN	24.59263	NaN	22.63201	NaN	26.13124	NaN	NaN	NaN
NaN	23.11267	NaN	NaN	21.36986	NaN	23.04093	23.42982	23.26785
24.78457	24.87643	24.86703	24.35783	24.60964	24.15653	NaN	NaN	NaN
NaN	NaN	23.97596	23.56384	24.11744	24.22435	24.35111	24.26069	24.1946
NaN	NaN	NaN	NaN	NaN	NaN	24.49228	24.391	24.01903
23.09184	23.29614	22.85377	23.09828	23.79947	23.80931	23.56384	NaN	23.88577
22.98294	NaN	22.97946	23.83846	23.32389	23.55215	24.03591	24.98468	24.06085
NaN	NaN	NaN	NaN	NaN	NaN	NaN	24.10949	24.7024
22.87267	NaN	23.84805	NaN	NaN	NaN	NaN	NaN	NaN
26.82639	26.77706	27.19835	27.28912	28.18423	27.88577	26.76446	27.509	26.49534
NaN	NaN	NaN	NaN	NaN	NaN	25.04009	NaN	NaN
NaN	NaN	NaN	NaN	NaN	NaN	25.96939	25.98033	26.20023
22.73686	23.42982	22.52133	23.31008	23.417	NaN	21.8839	20.79551	NaN
23.99335	24.2535	23.83846	24.17188	24.21697	24.56384	24.01052	24.16423	23.81909
24.23169	24.51051	24.62087	25.01052	25.39428	25.39754	24.91342	24.37119	24.46762
NaN	NaN	NaN	NaN	NaN	22.83461	23.93157	23.54038	23.59833
24.78955	25.01903	24.54038	24.60964	24.54628	NaN	NaN	23.73892	24.64856
24.23169	22.31285	24.1488	22.24771	22.00539	23.44253	NaN	22.35111	22.20658
NaN	21.66275	NaN	NaN	20.76344	NaN	23.04093	22.33211	23.04593
24.69179	24.27498	23.88577	23.66492	23.85757	24.05258	23.17188	23.99335	23.62087
22.78357	22.58233	22.18858	23.09507	22.65622	22.87267	24.59263	24.18707	23.42982
NaN	NaN	23.93157	24.12534	23.78955	23.44253	23.26785	NaN	23.40406
23.37782	NaN	23.0559	23.04427	NaN	NaN	NaN	NaN	NaN
NaN	NaN	NaN	24.98033	25.32732	24.76951	NaN	24.84805	23.90426
23.57542	NaN	23.23169	23.63201	23.62087	23.67573	NaN	NaN	NaN
NaN	NaN	NaN	23.11426	23.11267	23.1047	NaN	23.46762	23.51653
NaN	NaN	23.75939	22.37518	23.23608	23.45513	23.71816	23.45513	23.5285
24.68646	25.15653	24.77957	24.99335	24.54038	24.28207	25.09346	24.89966	25.01903
23.93157	23.90426	NaN	23.35111	23.46762	23.16423	NaN	NaN	NaN
23.1031	24.87173	23.71816	24.10149	23.49228	23.97596	22.53801	24.10949	23.49228
25.56384	25.7024	25.69975	26.28912	26.38113	26.44253	26.39917	26.30835	26.60399
25.77455	24.56384	25.85757	25.07725	25.2856	25.56384	23.79947	23.84805	23.65403
24.07725	NaN	22.80735	24.50446	24.35111	24.08537	24.22435	24.31008	24.08537
28.63547	25.02749	28.52402	28.08132	28.62506	28.10649	NaN	NaN	NaN
25.7415	26.36953	25.2571	25.1946	24.82881	25.65949	25.27498	24.44884	25.06497
NaN	NaN	NaN	NaN	NaN	NaN	24.32389	24.34435	24.1795
NaN	NaN	NaN	NaN	NaN	22.88762	25.239	24.22435	24.23169
24.89504	24.91798	24.96279	25.46139	25.90656	25.59548	26.36119	26.06085	26.33586
NaN	NaN	NaN	NaN	24.43619	NaN	24.26069	NaN	23.92252
NaN	23.26785	NaN	NaN	NaN	NaN	NaN	NaN	NaN
26.77706	26.57542	26.97596	26.80193	26.73892	26.38607	23.31008	23.32389	NaN
26.75175	26.64581	25.94726	25.64581	25.39754	25.82881	26.19835	26.23352	25.87643
NaN	NaN	NaN	NaN	NaN	NaN	NaN	NaN	NaN
25.29263	NaN	25.37451	25.18707	NaN	NaN	NaN	NaN	NaN
25.85994	26.20396	25.89735	26.90885	26.87408	26.63201	25.51352	25.07316	25.34773
24.57542	24.51653	24.83846	24.15653	24.38443	24.48616	24.77957	25.24989	24.89504
26.97596	26.71293	27.04009	26.9428	26.65949	26.52253	26.4999	25.39754	25.59263
23.92252	24.45513	23.80931	23.12692	22.68003	21.98468	22.83074	22.78756	22.87079
25.54333	25.66221	25.53148	25.36786	NaN	25.00196	NaN	NaN	NaN
28.87994	28.89735	29.14831	27.42342	27.58978	27.67976	26.85043	26.99766	26.78955
NaN	NaN	24.28912	23.36453	24.33074	24.58118	25.06497	25.317	25.00624
NaN	23.20807	NaN	NaN	NaN	NaN	25.55801	NaN	22.62979
NaN	NaN	23.11902	22.80538	23.73892	23.417	NaN	NaN	23.12218
24.83846	24.83364	24.76446	25.47692	25.48616	25.53445	25.79699	25.60964	25.82396
24.20955	23.51653	23.55215	NaN	24.63201	23.64306	22.5545	23.58692	22.66492
NaN	23.56384	NaN	22.87455	22.93337	25.16039	22.09507	23.59833	23.391
24.06907	24.76446	24.46762	24.93607	24.74919	24.94949	23.44253	24.317	23.64306
24.86703	25.06497	24.99766	24.84326	24.86703	24.98468	25.49534	24.89504	25.44884
24.03591	23.90426	23.6971	23.64306	23.48001	23.19761	22.2214	22.11108	NaN
25.01478	22.65185	24.89504	25.6513	25.88577	25.73376	26.07521	25.60116	25.94949
24.42342	25.78457	24.2535	24.09346	23.99335	23.97596	23.58692	23.26785	23.21401
22.37518	22.40925	22.04926	NaN	22.09184	22.20359	22.18556	22.2535	NaN
23.11744	NaN	NaN	22.98815	NaN	NaN	24.89504	24.23169	NaN
22.99507	NaN	NaN	NaN	22.52133	NaN	NaN	NaN	NaN
25.32732	25.48001	25.92025	25.18329	25.20955	25.58978	25.26069	25.08537	25.19835
NaN	NaN	NaN	24.06907	NaN	23.89504	23.59833	23.6971	NaN
24.64856	24.71293	24.76446	24.23169	24.12534	24.46762	24.88577	24.37119	24.62087
24.1488	24.83364	23.85757	NaN	NaN	NaN	25.44569	25.44884	25.05258
NaN	NaN	NaN	24.94949	25.12534	25.08537	24.86231	25.29263	25.51051
24.77957	23.92252	24.57542	24.22435	24.02749	24.58692	24.37119	24.46139	23.33756
NaN	NaN	NaN	NaN	NaN	NaN	23.42982	24.64856	23.56384
NaN	NaN	NaN	21.55918	NaN	NaN	NaN	NaN	22.40406
24.00196	22.85567	23.76951	23.71816	NaN	23.54038	23.77957	23.50446	23.32389
22.43237	NaN	NaN	21.7265	22.47507	22.58003	21.22435	23.54038	23.56384
24.54628	23.77957	24.35783	25.71293	25.93607	26.26249	26.57398	27.07112	26.67303
21.3538	19.38785	21.59149	20.98815	21.86891	21.23608	23.87643	22.9566	24.72858
24.45513	24.04427	24.74407	24.42982	22.78756	24.84326	22.04593	24.68646	21.89134
24.58692	25.16806	24.417	25.04009	24.92252	24.71816	NaN	NaN	NaN
22.38574	NaN	20.53564	NaN	NaN	NaN	NaN	NaN	NaN
22.87643	23.391	NaN	22.36185	22.25061	NaN	NaN	NaN	NaN
24.51051	23.70767	NaN	24.239	24.68646	NaN	24.46762	25.22803	25.24264
25.49534	25.89966	25.46451	25.06085	25.56964	25.35111	24.6811	25.29263	25.08132
NaN	NaN	NaN	NaN	NaN	NaN	23.96719	NaN	NaN

NaN	NaN	NaN	NaN	NaN	NaN	NaN	NaN	NaN
NaN	NaN	23.95837	23.01052	NaN	23.72858	22.9968	23.49228	NaN
22.46513	22.77957	22.33484	22.31285	22.20658	22.48247	22.80144	22.65403	22.85757
23.73892	NaN	23.94949	23.98468	24.391	23.94949	23.95837	24.09346	24.15653
24.64856	25.00196	24.59833	24.48616	24.89504	24.8044	24.78955	25.1946	25.00196
22.63644	NaN	NaN	NaN	NaN	NaN	NaN	23.17035	22.92615
NaN	NaN	NaN	23.05755	22.79154	23.16576	NaN	NaN	NaN
NaN	NaN	22.53326	21.81909	NaN	22.41957	NaN	NaN	21.88017
NaN	NaN	23.59833	23.92252	24.51653	23.92252	NaN	NaN	NaN
23.62087	23.87643	23.65403	24.10949	23.77957	23.24191	23.49228	23.23316	23.14569
23.67573	23.56384	23.86703	22.91159	23.20658	23.35111	22.42726	22.9812	22.51653
NaN	NaN	NaN	NaN	NaN	NaN	23.70767	NaN	22.66275
23.37782	NaN	23.29614	NaN	21.90242	NaN	24.64856	24.51653	24.41054
24.67573	24.35783	NaN	24.02749	24.96719	24.01052	24.21697	24.08537	24.03591
23.42982	23.35111	22.94235	22.91342	23.40406	22.74304	NaN	NaN	NaN
24.96279	25.03171	24.6811	25.06085	24.98468	24.8811	24.83846	24.54628	24.56964
23.29614	NaN	22.93337	22.69922	23.03255	22.94235	NaN	NaN	NaN
NaN	NaN	NaN	NaN	NaN	NaN	NaN	NaN	23.93157
26.1055	NaN	25.1488	NaN	NaN	NaN	NaN	NaN	NaN
NaN	NaN	NaN	NaN	NaN	NaN	NaN	24.00196	NaN
26.99766	29.04009	27.19835	28.29351	27.66628	27.64581	28.97869	30.36202	30.40324
NaN	NaN	NaN	NaN	NaN	NaN	NaN	23.56384	NaN
23.73892	23.24045	23.48001	23.29614	23.57542	23.26785	23.45513	23.50446	23.23169
24.317	24.2535	23.86703	24.20955	24.46139	24.33756	NaN	NaN	NaN
23.67573	23.68646	23.6971	23.57542	23.44253	23.58692	23.51653	23.57542	23.23608
NaN	NaN	22.79154	NaN	23.26785	23.04093	NaN	NaN	NaN
24.74407	24.7024	24.58692	24.09346	24.09346	24.26069	24.10149	24.08537	24.12534
NaN	NaN	24.11744	24.41054	NaN	NaN	23.32389	23.50446	NaN
24.06907	25.49228	24.72338	NaN	23.51653	23.75939	24.98033	25.09346	24.03591
23.05092	23.2535	23.16116	23.11585	22.91159	23.16116	24.04427	23.83846	23.86703
NaN	NaN	NaN	NaN	NaN	23.54038	24.2535	24.2535	24.48001
NaN	NaN	22.52373	22.66709	22.90976	NaN	23.88577	23.86703	23.87643
23.98468	23.01052	25.01478	24.06907	24.05258	23.59833	24.91342	NaN	25.12534
NaN	NaN	NaN	24.38443	23.97596	NaN	24.07725	24.86231	24.88577
24.23169	24.11744	24.42982	24.01903	24.24627	24.26069	24.42982	24.02749	24.22435
28.36202	26.87408	28.55362	28.01372	28.11148	28.11148	27.34942	27.41538	27.86231
23.59833	23.44253	23.40406	23.2214	24.10149	23.80931	23.87643	24.01903	23.99335
NaN	NaN	NaN	24.17188	NaN	23.87643	25.07725	24.34435	24.26785
NaN	NaN	23.76951	23.239	23.10149	NaN	24.12534	23.91342	23.10629
23.24481	23.50446	23.48001	23.13163	22.85377	22.97596	23.12849	23.0071	22.65185
23.09507	23.65403	24.1332	23.44253	23.62087	24.01052	24.59833	24.71816	23.89504
24.26785	24.44884	23.63201	NaN	24.39754	23.66492	24.04427	24.89504	24.417
23.75939	24.48001	23.82881	23.5285	23.82881	23.58692	24.07725	NaN	23.96719
24.52253	23.55215	23.48001	24.46762	24.92252	24.18707	21.1332	NaN	22.78157
24.59263	24.94503	24.52253	24.86231	25.38772	24.76951	25.97158	26.01052	26.04843
NaN	NaN	NaN	NaN	NaN	NaN	NaN	NaN	NaN
NaN	NaN	NaN	NaN	NaN	23.5285	NaN	NaN	NaN
23.77957	23.45513	24.38443	22.82104	23.33756	23.417	22.93517	22.91707	22.4501
23.71816	NaN	24.27498	24.06085	24.08537	23.98468	NaN	NaN	NaN
NaN	NaN	NaN	NaN	NaN	NaN	NaN	NaN	NaN
NaN	NaN	NaN	NaN	NaN	NaN	NaN	NaN	NaN
27.12139	NaN	26.44569	26.57542	27.87994	27.06085	23.85757	25.36786	NaN
23.70767	24.05258	23.83846	23.68646	23.44253	24.05258	23.56384	23.33756	23.07235
23.92252	24.01903	24.44884	24.05258	23.04593	NaN	NaN	NaN	22.48001
NaN	NaN	NaN	24.39754	24.41054	24.24627	25.06085	25.14102	24.60964
22.07235	22.95127	22.49717	22.14258	22.42982	22.1673	23.29614	23.33756	23.0805
26.10149	26.01052	26.52402	26.09547	26.36119	26.49838	28.08639	27.67303	27.97049
NaN	NaN	NaN	23.55215	NaN	NaN	NaN	NaN	NaN
NaN	NaN	NaN	22.76547	NaN	NaN	NaN	NaN	NaN
NaN	NaN	NaN	NaN	NaN	NaN	21.44253	NaN	21.71816
NaN	NaN	NaN	NaN	NaN	NaN	NaN	NaN	NaN
23.81909	24.1488	24.41054	23.94056	24.239	23.93157	24.59263	24.54038	24.93157
NaN	NaN	NaN	22.49473	23.06415	NaN	23.31008	23.6971	23.14258
22.73066	23.22729	22.83846	22.93157	23.63201	23.08861	22.78756	22.74509	22.96543
NaN	NaN	23.17341	NaN	NaN	NaN	22.78756	22.92976	22.92252
23.79947	NaN	23.71816	22.94414	22.86136	23.49228	NaN	NaN	NaN
23.78955	23.79947	23.82881	23.67573	23.79947	NaN	NaN	NaN	23.2006
NaN	NaN	23.81909	24.11744	24.31008	24.52253	24.11744	23.99335	24.42342
25.59548	NaN	26.90885	26.0874	26.56529	26.65949	27.20769	25.74407	27.0296
25.00196	24.77455	24.76951	24.87643	24.55801	24.56964	23.79947	24.81421	24.90426
NaN	NaN	22.93517	23.03926	NaN	23.40406	NaN	NaN	NaN
24.63755	25.17188	NaN	25.1488	25.84566	23.29614	24.76446	23.94056	NaN
NaN	NaN	NaN	NaN	NaN	NaN	NaN	NaN	NaN
NaN	NaN	NaN	NaN	NaN	NaN	NaN	NaN	NaN
NaN	23.0592	NaN	NaN	NaN	NaN	NaN	NaN	NaN
25.23169	25.89041	24.76446	25.51051	22.9566	24.99335	22.98989	25.34096	NaN
25.7024	NaN	24.89504	25.15653	25.43301	25.59833	25.61807	25.98685	26.36453
23.77957	NaN	23.50446	NaN	23.29614	23.70767	24.23169	NaN	23.92252
NaN	NaN	25.06497	NaN	NaN	NaN	NaN	NaN	NaN
24.14102	23.98468	23.91342	25.10149	24.34435	24.40406	23.58692	NaN	26.37617
NaN	21.391	NaN	21.62756	NaN	NaN	21.66275	21.8423	21.85377
NaN	23.73892	23.68646	23.60964	NaN	23.70767	NaN	NaN	NaN
23.64306	23.84805	24.96719	24.51051	24.15653	24.46139	24.28207	24.28207	24.78955
24.60399	23.64306	23.33756	23.32389	23.40406	22.56384	24.5285	24.51653	24.82396
21.2764	NaN	24.57542	NaN	NaN	NaN	21.18253	NaN	20.73892
NaN	NaN	NaN	NaN	NaN	NaN	26.18139	25.98251	26.06497
24.06085	24.7024	24.15653	23.93157	24.54038	24.1946	NaN	NaN	NaN
NaN	NaN	NaN	NaN	NaN	NaN	NaN	NaN	NaN

NaN	NaN	22.4247	22.67573	22.94235	23.42982	NaN	NaN	NaN
23.87643	24.03591	23.08213	NaN	24.04427	24.08537	NaN	NaN	23.29614
22.79352	23.79947	23.78955	23.76951	23.6971	23.71816	24.06085	NaN	NaN
NaN	NaN	NaN	NaN	NaN	22.1332	22.99335	23.05424	22.63201
24.95837	24.98468	24.78955	24.62645	24.63755	25.23169	24.62087	24.317	24.40406
NaN	NaN	NaN	22.48738	22.44506	22.68432	NaN	NaN	NaN
25.68913	25.76699	25.2856	24.96719	25.2535	25.16039	25.12534	25.02749	24.88577
23.60964	NaN	NaN	23.5285	23.86703	23.84805	NaN	NaN	NaN
20.32113	22.38837	21.73066	23.78955	22.30452	21.45262	NaN	20.6971	NaN
21.89134	21.95305	21.68859	22.22435	NaN	20.97421	23.13477	22.63201	22.77555
NaN	24.417	NaN	24.10949	23.98468	23.92252	24.1946	24.53445	24.46139
23.45513	23.391	23.15808	22.73686	NaN	NaN	NaN	NaN	NaN
23.03926	23.40406	22.91342	23.28207	22.80735	22.94592	22.2006	22.92434	23.00367
24.60964	NaN	NaN	NaN	NaN	NaN	NaN	NaN	NaN
22.48493	NaN	22.61639	22.49717	22.39624	22.32663	22.71816	NaN	23.74919
22.55918	23.46762	22.59377	23.31008	24.2021	23.20508	NaN	NaN	NaN
NaN	NaN	NaN	NaN	22.96719	NaN	23.60964	23.54038	23.48001
24.33756	24.79947	24.417	24.90885	25.64031	25.239	26.32045	25.98902	25.91114
22.24481	23.1206	21.1332	NaN	NaN	NaN	NaN	21.18858	21.0426
NaN	24.24627	24.28912	24.1946	24.29614	24.1488	23.72858	23.32389	23.59833
NaN	24.2535	NaN	24.28912	26.35111	NaN	NaN	NaN	NaN
NaN	NaN	24.04427	23.24916	23.81909	23.78955	NaN	NaN	23.37782
20.94592	21.61863	20.81127	NaN	20.56384	NaN	21.63201	NaN	21.44253
NaN	NaN	22.57311	23.28207	23.18858	22.90426	NaN	NaN	NaN
NaN	22.60738	23.40406	23.09507	23.81909	24.1946	NaN	23.28207	22.51653
28.66628	28.68979	28.69312	NaN	28.19835	28.97049	29.27586	29.08132	NaN
23.72858	23.80931	NaN	23.70767	23.81909	23.95837	24.06907	24.04427	24.1488
23.67573	NaN	NaN	NaN	NaN	NaN	NaN	NaN	NaN
23.03255	24.48001	NaN	NaN	23.68646	23.50446	24.239	24.39754	23.75939
23.81909	NaN	NaN	23.94949	23.62087	23.75939	NaN	NaN	NaN
28.09144	28.9428	28.01372	26.89735	26.82639	27.41538	26.5285	25.85757	26.40892
NaN	NaN	NaN	NaN	NaN	NaN	24.38443	24.51051	24.10149
NaN	NaN	NaN	NaN	21.96014	NaN	NaN	NaN	NaN
25.28207	25.55215	25.45826	25.27142	25.22066	25.15267	24.49228	24.44884	24.22435
21.87267	22.39624	21.96014	21.9812	21.83461	NaN	21.68003	21.64966	NaN
NaN	NaN	NaN	NaN	23.84805	23.67573	24.41054	24.76446	24.81421
25.89273	25.85043	25.45199	25.35111	25.57542	25.73635	23.35111	23.35111	23.32389
24.92705	25.72338	24.2535	23.24045	23.23169	23.54038	NaN	NaN	NaN
27.88577	28.01372	25.09346	24.74407	25.6811	24.77455	27.33245	24.26785	26.67303
22.54038	23.09184	22.47259	22.34571	22.11744	22.4247	24.11744	24.78955	24.03591
NaN	NaN	NaN	23.85757	NaN	NaN	26.04218	26.12928	26.0874
25.82639	26.1035	25.87877	26.13124	NaN	NaN	25.90656	25.66492	24.06085
NaN	NaN	NaN	NaN	NaN	NaN	NaN	NaN	NaN
22.51171	22.58233	22.91342	22.77354	22.65403	22.51171	22.13946	22.46762	22.95482
NaN	23.62087	NaN	NaN	23.60964	23.56384	24.95394	25.34773	24.77455
21.2006	NaN	NaN	21.42726	NaN	21.32113	21.34299	NaN	21.38574
24.85757	23.33756	23.55215	23.23608	23.94056	23.77957	NaN	22.44758	22.67573
24.1488	24.317	23.73892	24.1795	25.04843	24.93157	24.50446	24.79452	24.45513
27.08132	26.75175	27.31527	26.61807	26.69975	26.61807	26.42662	26.22251	26.64581
NaN	NaN	NaN	NaN	NaN	NaN	25.19084	24.56964	25.391
21.71816	NaN	NaN	NaN	NaN	NaN	NaN	NaN	NaN
NaN	23.04926	23.40406	22.1673	NaN	NaN	22.37252	23.21697	NaN
NaN	NaN	NaN	NaN	NaN	NaN	NaN	23.16269	22.97071
NaN	NaN	NaN	23.57542	NaN	NaN	NaN	NaN	NaN
24.97158	25.1946	24.8044	25.24264	25.11744	25.4073	25.54628	25.63201	25.44884
NaN	NaN	NaN	NaN	NaN	NaN	NaN	NaN	23.93157
23.97596	23.93157	23.94949	23.87643	23.80931	23.88577	23.36453	23.31008	23.83846
23.12534	NaN	23.6971	23.82881	23.66492	23.56384	22.69922	23.50446	22.84805
25.36453	25.43619	25.49534	25.6513	25.34096	24.99766	24.33074	25.62087	24.98033
NaN	23.72858	23.09507	NaN	23.17493	23.85757	NaN	NaN	NaN
22.93337	23.46762	23.00024	24.01052	23.391	24.239	23.48001	24.48001	22.81519
NaN	23.417	23.49228	24.33756	24.26785	24.48001	22.77354	NaN	NaN
NaN	NaN	NaN	NaN	NaN	NaN	NaN	22.74098	21.58233
NaN	23.62087	22.78556	23.14258	23.63201	23.74919	24.51051	24.31008	24.57542
21.90242	22.45764	21.76749	22.32113	22.40145	21.8423	22.20955	22.28771	22.62756
NaN	NaN	NaN	22.24771	NaN	NaN	24.32389	25.57542	24.62087
23.77957	NaN	NaN	23.13006	NaN	22.69073	NaN	NaN	23.25061
24.78457	24.59833	26.26428	27.19835	26.30835	26.29263	NaN	26.01265	25.39428
NaN	NaN	NaN	24.01052	23.74919	NaN	24.09346	24.417	23.73892
23.01733	23.1991	22.89874	24.01903	23.77957	24.01903	24.85281	24.51051	NaN
26.09144	26.22619	26.02538	26.1546	25.93157	26.06085	25.74663	25.75939	25.77455
24.48616	NaN	22.85946	24.48001	25.14492	24.71816	24.68646	24.79947	24.51051
NaN	NaN	NaN	NaN	NaN	NaN	NaN	NaN	NaN
NaN	NaN	23.89504	NaN	NaN	NaN	NaN	23.6971	NaN
24.96719	24.82396	24.35783	26.1488	26.04635	25.15653	25.34773	25.1488	25.43936
23.87643	24.57542	23.95837	24.417	24.52253	24.23169	24.87643	25.01903	25.02749
24.33756	24.64306	24.70767	25.5285	24.55801	24.96719	25.71293	26.57542	26.02749
NaN	24.01052	23.50446	23.67573	24.06085	23.83846	24.82396	24.55801	24.71293
NaN	NaN	22.94949	22.71188	NaN	NaN	NaN	NaN	NaN
23.09346	23.32389	23.391	23.67573	23.76951	23.94949	NaN	NaN	NaN
24.08537	NaN	24.04427	24.09346	24.7543	NaN	NaN	NaN	NaN
24.09346	23.93157	23.88577	23.21549	24.08537	23.74919	23.65403	23.88577	23.57542
22.98294	23.37782	22.9061	22.90793	22.99852	22.89689	22.55918	22.71188	22.78556
23.94949	NaN	24.42982	23.97596	23.72858	24.06907	NaN	NaN	NaN
NaN	NaN	NaN	NaN	NaN	NaN	NaN	NaN	NaN
26.82639	27.09144	27.24445	26.47228	26.71293	26.51653	26.69975	26.58978	26.39428
21.61414	NaN	22.26785	22.08861	20.08213	NaN	23.73892	22.94592	NaN
NaN	NaN	NaN	NaN	NaN	NaN	NaN	NaN	NaN

25.07316	24.96719	23.70767	NaN	23.67573	23.66492	24.30313	24.31008	24.28207
24.49838	23.46762	23.99335	23.79947	23.65403	22.77957	23.79947	23.42982	24.09346
NaN	24.00196	23.80931	23.21993	23.97596	23.80931	24.64856	25.28912	24.71816
22.1519	NaN	NaN	22.6006	NaN	NaN	NaN	NaN	NaN
24.41054	NaN	24.08537	NaN	22.70556	NaN	NaN	21.74304	NaN
NaN	NaN	NaN	NaN	NaN	NaN	25.02749	25.28207	24.55215
28.87994	29.17712	28.84146	28.76762	28.83244	NaN	29.1795	28.93719	28.86231
26.76446	26.44727	26.43619	26.07928	27.2535	27.07112	27.43936	27.67976	27.54628
NaN	NaN	NaN	NaN	NaN	NaN	NaN	NaN	NaN
NaN	22.18858	21.6584	22.09184	23.00881	22.63201	NaN	NaN	NaN
23.73892	23.24916	22.84805	23.99335	NaN	NaN	24.27498	24.54038	NaN
NaN	NaN	NaN	NaN	NaN	NaN	22.56384	22.69073	22.44506
NaN	NaN	NaN	NaN	NaN	NaN	NaN	NaN	NaN
NaN	NaN	21.75124	22.18556	22.38047	NaN	NaN	22.56384	22.38574
NaN	NaN	NaN	NaN	22.70767	22.22729	22.36986	22.58003	NaN
22.13634	NaN	22.18556	21.83074	23.07071	NaN	22.64966	22.33756	22.52612
25.89735	NaN	25.61526	26.20023	26.12731	26.07928	25.50446	NaN	NaN
NaN	NaN	NaN	NaN	NaN	NaN	NaN	22.40406	22.69285
21.26499	NaN	21.98815	23.40406	21.51653	NaN	23.40406	25.1332	23.35111
23.35111	23.01563	23.29614	23.57542	24.00196	24.16423	22.74714	23.00024	22.53564
NaN	NaN	NaN	NaN	NaN	NaN	NaN	28.43141	NaN
25.98033	26.08942	26.41216	26.1776	26.43141	26.61807	25.76699	25.74919	25.58118
NaN	NaN	NaN	NaN	22.79551	NaN	NaN	NaN	NaN
NaN	NaN	23.32389	23.36453	23.70767	NaN	24.02749	23.93157	NaN
22.08537	22.41442	21.88762	22.22729	21.48738	22.18253	22.32389	NaN	22.0426
NaN	NaN	NaN	23.51653	23.55215	23.44253	23.80931	23.28207	23.75939
23.28207	23.81909	NaN	23.84805	23.64306	24.06085	NaN	23.36453	23.85757
NaN	NaN	NaN	NaN	NaN	NaN	NaN	NaN	NaN
23.51653	22.1047	NaN	22.75939	23.40406	22.85946	23.46762	23.50446	23.391
NaN	24.26069	NaN	24.48001	24.32389	23.67573	NaN	23.96719	NaN
NaN	NaN	23.45513	NaN	NaN	NaN	NaN	NaN	NaN
NaN	NaN	23.26785	NaN	NaN	NaN	NaN	NaN	NaN
24.239	24.53445	24.28912	23.40406	23.73892	23.63201	22.73066	22.70556	NaN
22.1795	21.92796	22.08213	22.68003	NaN	22.1946	NaN	NaN	NaN
26.38443	25.96279	26.39917	26.55362	26.44411	26.52552	25.42662	25.34773	25.28912
24.11744	24.47383	23.91342	24.42342	24.64856	23.99335	24.89966	25.26428	24.90885
26.2535	26.24264	26.2571	26.17379	26.09346	26.4186	27.89735	27.96499	27.76446
23.48001	23.78955	23.66492	23.74919	23.79947	23.84805	NaN	NaN	NaN
NaN	NaN	NaN	NaN	24.09346	NaN	NaN	NaN	NaN
NaN	NaN	23.417	23.66492	NaN	NaN	NaN	NaN	NaN
NaN	NaN	22.4996	NaN	NaN	NaN	NaN	NaN	NaN
NaN	NaN	21.66275	NaN	22.13946	NaN	22.39885	22.2006	NaN
NaN	NaN	23.85757	NaN	NaN	NaN	25.1332	24.91798	25.15267
NaN	NaN	22.91889	23.78955	23.59833	NaN	24.08537	24.91798	24.52253
25.10949	NaN	25.61807	26.22803	26.1055	26.18707	24.23169	NaN	25.34773
NaN	NaN	NaN	NaN	NaN	NaN	NaN	NaN	NaN
NaN	NaN	NaN	NaN	NaN	NaN	NaN	NaN	NaN
NaN	NaN	NaN	24.17188	24.23169	NaN	24.38443	25.1757	25.53148
28.88867	29.104	28.95116	28.97596	29.08639	28.98685	28.43141	28.11644	28.58262
NaN	NaN	NaN	NaN	NaN	NaN	NaN	NaN	NaN
NaN	NaN	NaN	NaN	23.14258	NaN	NaN	NaN	NaN
NaN	NaN	NaN	NaN	NaN	NaN	NaN	NaN	NaN
26.2803	24.37782	25.80193	25.74919	25.89504	25.62366	25.46762	25.12928	25.29614
NaN	NaN	NaN	23.54038	NaN	23.42982	23.09184	23.1488	23.46762
25.81909	25.28207	25.56674	25.92025	25.7103	25.92479	26.1795	26.16231	26.2553
23.88577	24.2535	24.00196	23.23169	23.54038	23.55215	23.417	23.68646	23.45513
25.239	25.45826	24.79947	25.35111	25.16039	25.1055	24.93157	24.7024	24.21697
24.23169	24.70767	24.74919	24.1795	25.1055	24.21697	NaN	NaN	NaN
NaN	NaN	NaN	NaN	NaN	NaN	25.97815	26.30661	25.89504
NaN	NaN	NaN	NaN	23.67573	23.36453	NaN	NaN	NaN
23.6971	NaN	23.42982	23.70767	23.58692	23.89504	26.11942	24.76446	24.85281
NaN	NaN	NaN	22.67573	23.33756	NaN	23.57542	23.26785	23.21104
23.87643	NaN	23.60964	24.01903	24.04427	24.09346	23.83846	24.35783	23.96719
NaN	NaN	NaN	NaN	NaN	NaN	23.23608	22.97246	22.68432
25.60682	25.07725	23.74919	24.87643	24.77455	24.63755	23.89504	NaN	NaN
23.81909	24.62645	24.26069	24.37119	22.55684	24.59263	23.26785	NaN	23.11426
23.67573	24.11744	22.93517	23.05755	23.42982	23.417	23.94056	NaN	23.82881
26.90885	NaN	NaN	NaN	NaN	NaN	NaN	NaN	NaN
21.92071	NaN	21.47259	21.97071	22.07235	NaN	NaN	NaN	NaN
NaN	NaN	NaN	NaN	NaN	NaN	NaN	NaN	NaN
25.88344	25.6811	25.317	25.23535	25.08132	25.50142	NaN	23.87643	NaN
25.9955	26.54922	25.63201	25.53445	24.90426	25.22803	26.92025	26.95394	26.85043
NaN	NaN	NaN	NaN	NaN	22.7265	23.80931	23.59833	NaN
NaN	NaN	21.60964	NaN	NaN	NaN	21.99507	22.8839	NaN
NaN	NaN	NaN	NaN	NaN	NaN	21.6584	NaN	NaN
NaN	NaN	23.91342	24.67573	24.66492	NaN	25.28207	25.41377	25.42342
25.85994	NaN	26.65949	26.17188	25.91798	26.14686	26.20023	25.66763	26.67303
23.97596	24.03591	24.1488	25.12534	25.02327	25.20583	27.47073	27.48616	28.51277
24.38443	25.22066	24.66492	NaN	NaN	NaN	25.23169	24.65949	NaN
NaN	NaN	NaN	NaN	22.57542	22.46513	NaN	NaN	NaN
25.34773	25.38443	24.75939	26.16231	26.1055	25.48308	25.08132	25.53445	25.13712
24.41054	24.12534	24.80931	25.1055	24.98902	25.58405	24.16423	24.91342	25.317
NaN	NaN	24.89504	25.33756	NaN	NaN	25.03171	NaN	NaN
23.49228	23.64306	23.28207	NaN	22.92252	NaN	NaN	NaN	NaN
NaN	NaN	NaN	NaN	23.91342	NaN	NaN	NaN	NaN
NaN	NaN	22.64526	NaN	NaN	NaN	NaN	NaN	NaN
24.35111	23.36453	23.00024	23.391	23.391	23.32389	22.89504	22.70134	22.82687
NaN	21.64526	NaN	21.29894	NaN	NaN	21.81909	22.47507	22.25638

NaN	NaN	21.26499	NaN	23.90426	NaN	NaN	NaN	23.21401
26.02538	26.14492	25.72338	25.71816	25.55801	25.86703	24.239	23.21697	24.21697
NaN	NaN	21.1332	NaN	NaN	NaN	21.45262	NaN	22.01563
NaN	NaN	NaN	NaN	NaN	NaN	NaN	NaN	NaN
20.53564	21.52612	20.96719	NaN	20.6006	20.67142	20.61863	21.89134	21.17035
25.15653	25.27142	25.35111	24.417	24.5285	24.58118	24.76951	NaN	24.51653
NaN	NaN	NaN	NaN	NaN	NaN	NaN	NaN	NaN
26.72598	26.73892	26.96499	27.28912	27.07112	27.20769	26.92025	26.65949	26.98685
NaN	NaN	NaN	NaN	NaN	NaN	22.7265	NaN	22.87079
NaN	NaN	NaN	NaN	NaN	NaN	22.08861	22.94414	NaN
23.73892	NaN	23.90426	23.85757	24.21697	24.42342	23.99335	23.95837	23.76951
23.96719	24.21697	23.85757	23.79947	NaN	NaN	23.80931	NaN	23.87643
24.39754	24.1946	23.49228	23.18556	23.58692	NaN	23.21252	22.79947	22.61863
22.25926	22.97071	22.73892	22.8839	23.15345	22.74098	NaN	NaN	NaN
23.62087	22.54038	NaN	23.00367	NaN	NaN	23.66492	23.49228	22.88762
23.73892	23.18404	23.65403	23.60964	23.36453	23.80931	22.31008	24.2535	22.88762
24.06907	23.78955	22.65622	23.91342	23.90426	23.66492	NaN	22.79947	22.74919
NaN	NaN	NaN	NaN	NaN	NaN	NaN	22.41442	NaN
NaN	NaN	23.88577	NaN	24.07725	24.22435	NaN	NaN	NaN
NaN	NaN	NaN	NaN	NaN	22.41442	NaN	NaN	NaN
NaN	24.42342	NaN	24.99766	24.90426	NaN	NaN	NaN	NaN
23.28207	23.32389	22.42726	20.32113	NaN	22.1795	NaN	22.26499	22.47754
23.391	22.99507	NaN	NaN	NaN	23.70767	23.1031	23.42982	21.89134
NaN	NaN	NaN	23.84805	NaN	NaN	23.26785	NaN	NaN
NaN	NaN	NaN	NaN	NaN	NaN	23.2535	22.74509	22.67357
NaN	23.37782	22.47507	23.77957	23.59833	23.55215	NaN	20.66275	NaN
24.56964	24.97158	24.2535	24.37119	24.24627	24.39754	24.93157	24.62645	24.37782
NaN	NaN	NaN	22.81909	NaN	22.7265	NaN	NaN	NaN
NaN	NaN	NaN	NaN	NaN	21.25926	NaN	NaN	NaN
NaN	22.04926	20.1946	19.65927	NaN	NaN	NaN	NaN	NaN
22.6231	NaN	23.26785	23.81909	24.06085	23.44253	NaN	22.92976	23.09184
NaN	25.71555	NaN	24.239	NaN	NaN	NaN	NaN	NaN
NaN	NaN	NaN	21.70556	NaN	NaN	NaN	NaN	22.77756
22.25638	NaN	NaN	22.58692	22.64526	NaN	NaN	NaN	NaN
NaN	NaN	NaN	NaN	NaN	NaN	20.62756	NaN	NaN
NaN	24.2535	NaN	24.32389	NaN	24.72858	23.37782	24.37782	NaN
NaN	NaN	NaN	NaN	NaN	NaN	NaN	NaN	NaN
NaN	NaN	NaN	NaN	NaN	22.62756	NaN	23.98468	NaN
24.02749	22.1795	22.44758	23.55215	21.54981	24.2535	NaN	NaN	NaN
22.81519	22.54274	NaN	22.14258	22.89504	NaN	NaN	22.74714	NaN
NaN	23.60964	NaN	23.33756	23.36453	23.391	NaN	NaN	NaN
22.417	NaN	NaN	22.1673	NaN	22.32389	22.90242	NaN	23.42982
24.50446	24.75939	24.65403	24.98468	25.03171	24.99766	25.37451	25.27498	25.44253
24.21697	NaN	24.86703	24.07725	NaN	24.04427	NaN	25.22066	24.46139
31.70471	29.78019	31.84969	31.26472	30.21928	30.59335	27.509	30.52028	27.77077
NaN	21.6006	NaN	NaN	NaN	NaN	NaN	20.75532	NaN
NaN	NaN	NaN	22.58692	NaN	NaN	22.70767	22.61414	23.07887
NaN	23.67573	NaN	23.88577	24.23169	24.14102	NaN	22.94949	22.58921
NaN	NaN	NaN	NaN	NaN	NaN	NaN	NaN	NaN
NaN	NaN	NaN	NaN	NaN	NaN	22.62533	NaN	22.32663
NaN	NaN	23.03926	NaN	22.32389	NaN	NaN	NaN	NaN
NaN	NaN	NaN	23.71816	23.2535	NaN	NaN	22.58233	NaN
26.75175	25.89735	27.01903	26.47537	26.86231	26.97596	27.0296	27.29789	27.2535
23.77957	24.01052	23.29614	23.59833	23.29614	23.50446	23.74919	23.26785	23.03758
NaN	NaN	NaN	22.09184	NaN	NaN	NaN	23.40406	NaN
NaN	24.417	23.51653	23.74919	23.59833	21.0625	22.70556	21.97071	22.09507
23.60964	NaN	NaN	NaN	NaN	23.23608	NaN	NaN	NaN
NaN	NaN	NaN	21.60513	21.53564	21.5451	22.60738	23.28207	22.86514
NaN	22.98815	NaN	23.417	23.22876	23.33756	NaN	NaN	NaN
NaN	NaN	NaN	NaN	NaN	NaN	NaN	NaN	NaN
21.93876	NaN	22.48001	21.21252	22.09507	22.57311	NaN	NaN	NaN
NaN	NaN	NaN	NaN	NaN	NaN	23.09023	22.86136	22.91525
NaN	NaN	NaN	NaN	NaN	22.91342	23.6971	23.86703	23.87643
NaN	NaN	NaN	NaN	NaN	NaN	NaN	NaN	NaN
22.33756	22.20955	22.21549	NaN	21.67573	21.54981	22.6006	22.47507	NaN
NaN	23.2535	NaN	NaN	NaN	NaN	NaN	NaN	NaN
NaN	NaN	NaN	NaN	NaN	NaN	NaN	NaN	NaN
23.37782	23.70767	22.99162	23.1931	23.02073	23.5285	NaN	NaN	NaN
23.25061	23.11902	NaN	23.24627	23.72858	23.89504	24.99335	25.06085	25.06907
NaN	NaN	NaN	NaN	NaN	NaN	NaN	NaN	NaN
NaN	NaN	NaN	NaN	NaN	NaN	NaN	NaN	NaN
24.37119	NaN	24.65949	23.87643	23.65403	23.75939	23.83846	24.16423	23.31008
NaN	NaN	NaN	24.44253	24.66492	NaN	NaN	24.57542	24.21697
23.37782	23.64306	23.28207	NaN	NaN	22.81323	NaN	NaN	NaN
23.29614	23.31008	23.00539	22.86325	23.06415	22.62533	NaN	23.15808	23.01223
NaN	NaN	NaN	NaN	NaN	22.24771	NaN	NaN	NaN
24.46762	NaN	24.52253	NaN	NaN	NaN	NaN	NaN	NaN
NaN	NaN	NaN	NaN	NaN	NaN	NaN	NaN	NaN
24.84805	24.80931	24.97596	25.02327	25.02327	24.98902	23.58692	23.79947	23.66492
NaN	NaN	22.18556	22.61639	22.80735	23.03086	23.15653	23.55215	23.09507
22.55918	23.00367	22.75328	22.37518	NaN	22.25638	21.98468	21.53564	21.32113
23.37782	24.77455	23.14258	24.74919	25.05672	24.23169	24.08537	24.71293	24.69179
22.98989	NaN	NaN	NaN	NaN	22.62087	24.38443	22.07235	23.62087
23.32389	23.78955	23.44253	23.93157	23.92252	23.59833	23.67573	23.48001	23.15962
NaN	NaN	NaN	NaN	NaN	NaN	NaN	NaN	NaN
NaN	NaN	22.03926	NaN	NaN	21.96367	NaN	NaN	22.56151
NaN	NaN	NaN	22.88204	NaN	NaN	23.16269	23.40406	23.391
22.35111	NaN	21.03591	NaN	NaN	NaN	22.84613	22.84805	22.69285

NaN	NaN	23.5285	24.35783	24.50446	NaN	24.51051	24.72338	24.85281
23.29614	23.68646	23.58692	23.57542	23.04593	NaN	22.53089	23.12692	23.33756
NaN	NaN	21.70556	22.0625	NaN	21.88017	NaN	22.18858	21.40145
23.57542	23.72858	23.83846	22.95837	22.79154	23.09989	NaN	22.79749	NaN
NaN	NaN	NaN	NaN	NaN	NaN	NaN	NaN	NaN
NaN	NaN	NaN	23.62087	NaN	NaN	24.391	24.26069	24.64856
22.90426	23.92252	NaN	24.90426	23.66492	24.11744	21.61863	21.51171	21.79551
NaN	NaN	NaN	NaN	NaN	NaN	NaN	22.75328	NaN
23.01733	22.9968	22.96895	NaN	NaN	NaN	NaN	NaN	NaN
NaN	NaN	NaN	NaN	NaN	NaN	22.34841	23.14569	22.07235
30.58441	30.69644	31.08639	30.48232	29.21928	29.61982	28.50142	29.42142	27.83846
NaN	NaN	NaN	NaN	NaN	NaN	NaN	NaN	NaN
NaN	NaN	22.47507	NaN	NaN	21.92071	NaN	NaN	NaN
26.27142	26.16806	26.41054	26.54922	25.7024	25.80193	26.29614	25.47383	27.63201
23.391	NaN	24.1795	NaN	NaN	24.55801	NaN	NaN	NaN
23.09989	23.80931	23.07561	23.25205	23.32389	23.28207	23.07398	22.98815	23.0476
NaN	NaN	23.54038	23.72858	24.65403	24.27498	NaN	NaN	23.5285
NaN	NaN	NaN	NaN	NaN	NaN	NaN	NaN	NaN
NaN	NaN	NaN	NaN	NaN	NaN	NaN	NaN	NaN
NaN	NaN	NaN	NaN	NaN	NaN	26.04009	NaN	NaN
24.93157	23.42982	24.95837	24.03591	23.95837	24.08537	23.87643	22.84995	23.03926
22.2006	21.64966	21.70978	23.50446	22.66275	23.63201	21.53089	21.98815	NaN
22.53801	NaN	NaN	21.09507	NaN	NaN	NaN	NaN	NaN
23.04093	23.417	22.90426	NaN	23.29614	NaN	23.88577	NaN	23.36453
NaN	NaN	NaN	NaN	NaN	22.32389	NaN	NaN	NaN
25.18707	NaN	NaN	NaN	NaN	NaN	NaN	NaN	25.15267
NaN	NaN	NaN	NaN	NaN	NaN	NaN	NaN	NaN
23.10949	23.37782	23.26785	22.77957	23.06085	24.01052	23.51653	23.5285	23.32389
NaN	NaN	NaN	NaN	NaN	NaN	23.86703	24.49838	23.26785
20.92434	22.1916	22.52612	21.8839	22.0426	21.8839	NaN	NaN	21.96014
NaN	NaN	NaN	NaN	NaN	23.12376	NaN	NaN	NaN
NaN	NaN	NaN	NaN	NaN	NaN	NaN	NaN	24.44884
24.20955	23.32389	24.48001	23.417	21.94235	22.09184	21.0426	22.28489	NaN
27.26249	27.37451	27.07112	27.24445	27.16039	26.88577	24.72858	24.78955	24.8811
22.02242	NaN	22.12692	21.80341	21.77153	21.92796	26.07521	25.64031	26.04218
NaN	23.55215	NaN	22.86325	22.86891	22.59377	NaN	NaN	NaN
NaN	NaN	NaN	NaN	NaN	NaN	22.35111	NaN	NaN
NaN	NaN	NaN	NaN	NaN	NaN	NaN	NaN	NaN
NaN	NaN	NaN	NaN	23.29614	NaN	NaN	NaN	NaN
NaN	20.53564	NaN	NaN	19.44758	NaN	NaN	22.00881	NaN
NaN	NaN	NaN	NaN	NaN	NaN	24.02749	23.70767	23.96719
NaN	NaN	NaN	21.75939	21.68432	21.0559	23.21252	22.81519	23.10789
NaN	NaN	24.04427	NaN	NaN	NaN	NaN	NaN	NaN
24.29614	24.35783	24.54628	24.59263	24.45513	24.89041	24.2021	24.48616	24.39754
23.33756	23.46762	23.40406	23.45513	23.16883	23.2535	23.5285	23.1519	23.37782
23.26785	NaN	22.79749	NaN	22.417	22.97246	23.37782	22.83074	NaN
23.04593	23.65403	23.49228	24.16423	24.33074	24.24627	24.21697	23.96719	23.70767
NaN	23.65403	NaN	23.92252	24.05258	NaN	23.14258	23.42982	23.68646
NaN	NaN	21.18253	NaN	NaN	NaN	NaN	NaN	20.91707
25.24627	25.59833	25.54038	24.98033	23.83846	25.1946	24.14102	24.48616	24.30313
22.73686	23.35111	23.51653	23.70767	22.73686	23.09989	20.53564	21.18858	21.33211
19.93157	NaN	20.93876	NaN	20.23023	20.9812	21.28771	22.68003	NaN
22.2214	NaN	NaN	NaN	NaN	NaN	22.79352	NaN	23.70767
NaN	NaN	NaN	NaN	NaN	NaN	NaN	22.68217	NaN
24.98468	25.13712	25.00624	23.94949	23.86703	24.06907	22.68646	23.14414	22.25638
NaN	NaN	NaN	NaN	NaN	NaN	NaN	NaN	NaN
24.89504	25.06085	24.97596	25.27142	24.67034	25.01478	25.45826	24.40406	25.1946
23.0258	NaN	NaN	23.18404	NaN	NaN	NaN	23.5285	NaN
NaN	NaN	NaN	NaN	NaN	22.74304	NaN	NaN	NaN
NaN	NaN	NaN	NaN	NaN	NaN	NaN	NaN	NaN
NaN	NaN	19.67487	21.59605	NaN	22.68859	19.98815	22.6006	21.94592
NaN	23.01733	22.96543	NaN	NaN	NaN	23.91342	23.92252	24.1488
23.5285	24.09346	24.06085	23.46762	23.31008	22.99335	23.85757	23.70767	23.75939
NaN	NaN	NaN	23.29614	NaN	24.50446	NaN	NaN	NaN
NaN	NaN	NaN	23.85757	NaN	NaN	24.41054	24.417	NaN
NaN	NaN	NaN	NaN	NaN	NaN	23.05424	NaN	22.78157
NaN	22.84038	NaN	22.44	23.01223	NaN	NaN	24.49838	22.84995
24.00196	24.44884	23.71816	23.18253	23.1961	23.45513	24.48001	24.55801	23.81909
27.29789	27.51653	27.49381	27.55362	27.509	27.67976	28.27586	27.95394	28.21234
22.63644	22.53326	23.37782	23.35111	NaN	NaN	23.1031	22.97946	NaN
25.79452	24.98468	25.8044	24.8044	25.82881	25.62645	25.94726	25.41054	25.83605
NaN	NaN	23.55215	23.76951	23.73892	NaN	23.11902	23.75939	NaN
NaN	NaN	NaN	NaN	NaN	NaN	23.68646	23.55215	23.70767
22.98642	23.40406	22.98468	22.82881	NaN	22.63201	22.69285	NaN	22.46513
NaN	22.09184	NaN	NaN	NaN	NaN	NaN	22.55918	NaN
NaN	24.62645	NaN	24.07725	NaN	NaN	24.54628	NaN	NaN
NaN	21.77957	22.31838	21.89134	NaN	21.86891	NaN	21.70556	NaN
NaN	NaN	27.27142	27.0296	27.63893	27.42342	25.95394	26.82639	26.01478
NaN	NaN	NaN	NaN	NaN	NaN	NaN	NaN	23.78955
24.49228	22.80931	24.33074	24.39754	24.35111	24.23169	24.08537	24.35111	25.86703
24.01052	24.05258	23.91342	23.6971	24.06085	24.29614	NaN	NaN	NaN
NaN	26.97596	NaN	NaN	NaN	NaN	NaN	NaN	NaN
NaN	NaN	NaN	NaN	NaN	NaN	27.69975	27.23535	27.47846
25.27498	NaN	24.85281	NaN	NaN	NaN	NaN	NaN	25.87173
NaN	NaN	NaN	NaN	NaN	NaN	NaN	NaN	22.94592
29.14346	28.19835	29.61982	29.19366	28.55362	29.65096	28.63201	28.61105	29.48808
23.79947	24.48001	23.55215	24.34435	24.04427	24.28912	24.10149	23.50446	23.55215
NaN	25.47692	25.11744	25.87173	25.53445	25.71555	24.05258	NaN	NaN

NaN	NaN	21.86514	NaN	NaN	23.92252	NaN	NaN	NaN
NaN	NaN	NaN	NaN	NaN	NaN	25.89966	25.55801	26.12337
NaN	NaN	21.75124	21.8423	22.20955	22.09828	22.97596	23.58692	23.76951
24.62087	NaN	24.23169	24.59833	25.08942	25.44884	24.94503	25.71293	24.54038
21.89504	22.23316	21.90242	22.43237	21.83846	NaN	21.55918	22.58003	21.51653
NaN	NaN	NaN	NaN	NaN	NaN	NaN	NaN	NaN
23.64306	23.417	24.09346	23.17646	23.36453	NaN	24.67034	24.80931	24.58692
23.86703	23.96719	24.42342	24.01903	23.71816	24.14102	NaN	NaN	NaN
NaN	NaN	NaN	NaN	NaN	NaN	22.51412	23.23023	22.42726
25.20583	NaN	25.06085	25.24627	24.72338	25.24627	22.42214	NaN	23.91342
28.59335	26.21327	28.52402	28.98414	29.18896	29.1866	28.63547	29.00838	28.66966
23.36453	NaN	23.06579	23.64306	23.68646	23.6971	23.64306	23.05755	23.14414
22.22435	NaN	22.56151	22.42214	22.57542	NaN	23.28207	23.29614	22.79154
26.32903	26.64581	26.22986	26.71293	26.55215	26.49228	24.10949	23.35111	23.417
24.01052	NaN	23.97596	24.1795	24.04427	24.14102	24.21697	24.1795	24.11744
NaN	NaN	NaN	NaN	NaN	23.88577	24.94503	24.417	24.5285
NaN	NaN	NaN	NaN	NaN	NaN	24.17188	24.08537	23.48001
25.35111	NaN	25.65403	26.01691	26.01691	25.87643	25.53742	25.62087	25.37782
23.6971	NaN	23.85757	23.67573	23.51653	23.62087	NaN	NaN	NaN
NaN	NaN	20.1332	18.08472	24.97596	24.09346	NaN	20.6971	NaN
NaN	NaN	NaN	NaN	NaN	NaN	NaN	NaN	NaN
NaN	NaN	NaN	NaN	NaN	NaN	24.01052	NaN	NaN
24.62087	24.55801	24.41054	24.35111	23.99335	24.26785	24.76951	24.63201	25.17188
22.17035	22.84613	22.94414	23.29614	23.40406	23.21549	20.36453	20.86514	NaN
25.43301	25.60116	NaN	26.38607	NaN	25.03591	26.01478	25.26069	25.24989
NaN	NaN	NaN	NaN	23.36453	23.80931	23.1488	24.24627	23.96719
NaN	NaN	25.06497	25.49534	25.391	25.80931	NaN	NaN	NaN
23.63201	24.08537	24.22435	24.44884	24.26785	24.33756	23.70767	23.57542	23.95837
24.38443	24.1488	24.07725	24.60399	25.05258	24.2535	NaN	NaN	NaN
25.21327	25.45199	25.30313	25.12139	25.01478	25.36786	26.37617	26.09748	26.55801
NaN	NaN	22.31562	22.57311	22.47259	NaN	22.72858	22.33756	NaN
NaN	NaN	NaN	NaN	NaN	NaN	NaN	NaN	NaN
NaN	NaN	NaN	24.78457	25.99335	25.62366	23.66492	NaN	NaN
NaN	NaN	NaN	NaN	23.24336	NaN	NaN	NaN	NaN
23.5285	23.09346	23.36453	NaN	NaN	NaN	22.65622	23.12849	22.63423
22.73892	23.2535	22.73273	21.99162	NaN	NaN	22.09507	NaN	22.15499
NaN	NaN	NaN	NaN	NaN	NaN	21.92434	NaN	NaN
23.15962	NaN	22.32937	23.1946	23.95837	23.31008	NaN	21.79154	NaN
NaN	NaN	NaN	NaN	NaN	NaN	NaN	NaN	NaN
25.29263	NaN	25.89966	24.50446	25.36453	25.21327	NaN	23.56384	NaN
NaN	23.51653	NaN	NaN	NaN	NaN	23.0625	23.06415	NaN
21.99852	22.9968	23.33756	23.35111	23.01052	22.95305	21.85757	22.31285	22.56384
23.36453	23.55215	23.46762	23.45513	23.54038	23.37782	23.54038	NaN	23.80931
19.2764	20.81127	20.73066	21.99162	20.82687	21.56848	19.8469	22.31008	NaN
23.19761	NaN	NaN	NaN	23.35111	23.1795	NaN	NaN	NaN
23.71816	23.66492	23.51653	23.01563	23.42982	22.92252	23.85757	23.0559	23.48001
NaN	NaN	NaN	NaN	NaN	NaN	NaN	NaN	NaN
NaN	NaN	NaN	NaN	NaN	NaN	NaN	NaN	24.92252
22.84038	23.02749	23.04427	22.65185	22.72442	22.70556	NaN	NaN	NaN
NaN	NaN	NaN	NaN	NaN	NaN	NaN	NaN	24.04427
NaN	NaN	NaN	NaN	NaN	NaN	NaN	NaN	NaN
23.17188	NaN	23.08213	NaN	22.90426	NaN	NaN	NaN	NaN
NaN	NaN	NaN	NaN	NaN	NaN	NaN	NaN	NaN
25.77706	25.89966	25.85757	26.57254	NaN	26.31527	25.9428	25.74663	26.05051
NaN	NaN	NaN	NaN	NaN	NaN	26.11545	25.44569	27.14102
24.02749	24.27498	24.06907	24.32389	24.28912	24.51653	23.90426	23.54038	23.59833
23.48001	NaN	23.86703	23.1332	23.21401	22.90426	23.48001	23.17341	23.18404
NaN	21.7348	NaN	22.27071	21.61863	21.39624	NaN	21.53089	21.49228
NaN	23.60964	NaN	23.84805	24.60964	NaN	22.35649	NaN	NaN
25.11744	25.18707	24.99766	25.70767	25.36786	25.30661	24.81421	23.24191	23.417
NaN	NaN	NaN	NaN	NaN	NaN	24.37782	NaN	NaN
NaN	NaN	NaN	NaN	NaN	NaN	25.28207	25.11744	25.29614
NaN	24.77957	NaN	24.98902	25.19084	23.13006	25.32389	25.58978	26.46762
NaN	NaN	23.50446	NaN	NaN	24.1795	NaN	NaN	NaN
25.29263	25.60682	24.79947	24.91342	NaN	24.72338	24.57542	NaN	24.05258
NaN	NaN	NaN	NaN	NaN	NaN	24.54038	24.1795	24.09346
NaN	NaN	NaN	22.89689	22.81323	NaN	NaN	NaN	NaN
NaN	NaN	NaN	21.40145	21.29334	NaN	NaN	21.64966	NaN
NaN	NaN	NaN	NaN	NaN	NaN	22.62087	23.20359	22.18858
22.65403	21.79947	NaN	22.84995	24.10149	NaN	22.73066	22.88204	22.75328
NaN	24.20955	NaN	22.85186	21.94949	NaN	22.78556	23.83846	NaN
NaN	24.54628	21.83846	NaN	NaN	NaN	22.68646	22.06579	NaN
NaN	22.31562	NaN	20.36453	NaN	NaN	NaN	21.86891	NaN
22.07887	22.57542	22.08537	22.69285	22.02918	23.02242	22.67142	22.78955	22.07561
NaN	NaN	NaN	NaN	NaN	NaN	NaN	NaN	NaN
NaN	NaN	NaN	NaN	NaN	NaN	NaN	NaN	NaN
NaN	NaN	NaN	NaN	NaN	NaN	NaN	NaN	NaN
24.48616	24.5285	24.92705	24.35783	24.26069	24.04427	25.19835	25.23535	25.58692
NaN	NaN	NaN	NaN	NaN	NaN	24.59833	24.16423	24.42982
NaN	24.46762	NaN	22.46264	22.29614	NaN	21.9812	23.1031	22.20658
NaN	24.94503	NaN	24.34435	NaN	24.73892	24.08537	23.85757	NaN
NaN	NaN	23.09023	NaN	NaN	NaN	NaN	NaN	NaN
NaN	21.5451	NaN	NaN	NaN	NaN	NaN	NaN	NaN
NaN	NaN	NaN	NaN	NaN	NaN	NaN	NaN	NaN
24.28912	24.41054	NaN	24.39754	24.38443	24.46762	24.47383	24.59263	24.69179
24.00196	24.08537	NaN	23.40406	NaN	24.09346	23.50446	NaN	NaN
23.20508	23.03758	22.57542	23.2535	NaN	23.24627	22.37782	22.76749	22.83653
NaN	NaN	NaN	NaN	NaN	NaN	23.02918	22.67357	22.51171

NaN	NaN	NaN	NaN	NaN	NaN	NaN	NaN	NaN
28.67976	26.92025	29.02433	29.08639	29.05569	29.08132	28.75812	28.96224	28.7357
22.0559	22.91525	21.79154	23.33756	23.04926	23.15962	NaN	NaN	20.60964
NaN	22.8783	NaN	22.74919	22.96719	NaN	25.86703	24.01052	24.01052
NaN	22.76142	NaN	22.85377	23.07561	23.48001	23.50446	23.417	23.417
NaN	22.47754	22.75735	23.46762	23.14414	23.97596	22.59377	22.24191	22.2535
NaN	23.83846	NaN	NaN	NaN	NaN	24.40406	23.85757	23.70767
NaN	21.46762	NaN	22.00539	21.36986	21.48738	21.78357	22.57542	22.29334
NaN	NaN	NaN	NaN	NaN	24.28207	NaN	NaN	NaN
NaN	NaN	NaN	NaN	NaN	NaN	NaN	NaN	25.93832
21.81127	22.55215	22.27923	21.96367	22.0559	22.31838	22.44506	22.57773	22.1519
22.9968	NaN	22.47754	22.81909	22.87643	22.92434	NaN	23.62087	NaN
NaN	NaN	23.44253	23.391	NaN	23.79947	23.71816	NaN	NaN
NaN	21.11426	NaN	21.2006	21.38574	NaN	21.23608	21.31562	21.49717
28.52402	27.98685	27.55362	27.45513	28.76762	28.41538	27.67976	27.90885	27.30661
NaN	NaN	NaN	NaN	NaN	NaN	NaN	NaN	NaN
NaN	NaN	NaN	NaN	NaN	NaN	NaN	NaN	NaN
NaN	NaN	NaN	NaN	NaN	NaN	NaN	NaN	NaN
NaN	NaN	23.14258	22.99162	22.87079	22.74304	23.21697	22.84995	23.31008
22.84805	22.80144	NaN	22.67357	NaN	NaN	23.391	24.28912	22.8783
22.68646	NaN	NaN	22.98815	NaN	22.82299	NaN	NaN	NaN
NaN	22.65622	21.99507	23.1031	22.51653	21.6971	NaN	21.61414	21.96014
NaN	NaN	NaN	NaN	NaN	NaN	NaN	22.65403	22.94771
23.23754	23.11902	23.417	23.35111	23.0592	22.93697	NaN	NaN	NaN
20.68859	NaN	NaN	NaN	NaN	NaN	23.1931	23.0426	23.15499
23.1047	23.93157	23.88577	24.14102	24.02749	23.63201	24.06085	24.06907	24.10149
23.44253	22.06907	23.68646	23.68646	24.05258	24.01052	25.13712	24.417	24.60399
NaN	NaN	NaN	NaN	NaN	NaN	NaN	NaN	NaN
NaN	23.75939	22.21845	22.37252	22.5545	22.28207	NaN	22.4996	NaN
NaN	NaN	NaN	NaN	NaN	NaN	NaN	NaN	NaN
NaN	NaN	NaN	NaN	22.82687	NaN	NaN	NaN	NaN
22.68859	NaN	22.75328	NaN	22.96543	23.08699	24.40406	24.31008	23.417
NaN	22.05258	24.33756	23.98468	24.21697	24.46762	23.17341	NaN	23.55215
NaN	NaN	NaN	NaN	23.12849	NaN	23.63201	23.51653	23.45513
22.21252	NaN	22.33211	21.70134	22.16423	NaN	NaN	22.55684	22.89689
NaN	22.38311	NaN	23.14414	22.76749	23.15808	NaN	NaN	NaN
NaN	NaN	NaN	NaN	NaN	NaN	NaN	NaN	NaN
NaN	NaN	NaN	NaN	NaN	NaN	NaN	NaN	NaN
NaN	NaN	NaN	NaN	NaN	NaN	NaN	NaN	NaN
NaN	NaN	NaN	NaN	NaN	NaN	NaN	NaN	NaN
25.02749	23.84805	23.6971	24.1332	24.02749	24.28912	24.54628	24.08537	24.1795
NaN	22.83653	NaN	NaN	NaN	NaN	NaN	23.46762	NaN
24.76446	24.7543	24.85757	24.93607	25.06085	25.1055	NaN	25.01052	25.19835
23.80931	23.70767	23.46762	NaN	NaN	23.46762	23.07235	23.28207	23.59833
NaN	NaN	24.23169	NaN	NaN	NaN	NaN	NaN	NaN
20.33211	NaN	NaN	NaN	NaN	NaN	20.82687	20.95305	20.91707
NaN	NaN	21.97071	23.33756	NaN	22.96014	23.45513	NaN	NaN
NaN	NaN	NaN	NaN	NaN	NaN	22.72858	22.25638	NaN
25.47073	25.97378	25.69179	25.89504	25.89041	25.16039	26.09346	25.26428	25.06085
NaN	NaN	NaN	NaN	NaN	NaN	NaN	NaN	NaN
25.01052	24.86703	24.8044	24.63755	24.6811	24.48616	NaN	NaN	NaN
NaN	NaN	NaN	NaN	NaN	NaN	NaN	NaN	NaN
25.23169	25.24264	NaN	25.47383	24.95837	25.23535	24.82881	24.239	24.64306
23.74919	NaN	23.98468	NaN	NaN	NaN	NaN	NaN	NaN
NaN	NaN	NaN	NaN	NaN	NaN	NaN	24.35111	23.83846
NaN	23.37782	NaN	24.26069	23.17646	NaN	NaN	NaN	NaN
23.07398	NaN	NaN	NaN	21.72234	22.29614	23.01052	22.54745	22.95127
22.59605	23.09828	22.32389	NaN	22.00196	22.28207	NaN	NaN	NaN
24.46762	25.09346	24.6971	24.5285	24.62087	23.94056	24.31008	23.94056	24.02749
NaN	NaN	NaN	25.42982	24.36453	NaN	24.10149	24.57542	23.96719
24.52253	24.97158	24.37119	25.14492	24.44253	24.91342	25.86467	25.71816	25.63755
NaN	NaN	22.5545	NaN	NaN	NaN	23.76951	NaN	23.1961
22.72234	23.54038	23.17035	22.85757	NaN	22.66926	24.07725	23.32389	24.20955
NaN	25.08942	NaN	22.7348	22.78357	NaN	NaN	NaN	NaN
NaN	NaN	NaN	22.62087	NaN	22.84613	22.42214	22.33756	22.40925
NaN	NaN	22.82687	24.23169	24.23169	23.51653	NaN	NaN	NaN
NaN	NaN	NaN	NaN	NaN	NaN	NaN	28.51277	NaN
NaN	NaN	NaN	NaN	NaN	24.1946	NaN	NaN	NaN
24.86703	NaN	24.34435	NaN	NaN	NaN	NaN	NaN	NaN
28.48616	26.71293	25.98902	25.27853	25.14492	25.32732	25.76951	25.83123	25.27498
24.79452	24.28207	24.86231	24.69179	25.27498	NaN	25.93832	25.44569	25.87643
NaN	NaN	NaN	NaN	NaN	NaN	NaN	NaN	NaN
NaN	NaN	NaN	NaN	NaN	NaN	23.96719	NaN	NaN
NaN	NaN	NaN	NaN	NaN	NaN	NaN	NaN	NaN

LQF Intensity PYMT RT1	LQF Intensity PYMT RT2	LQF Intensity PYMT RT3	Protein IDs	Majority protein IDs	Protein names	Gene names
33.13614	33.18719	33.21928	Q9JHU4;F6ZX84	Q9JHU4	Cytoplasmic dynein 1 heavy chain 1	Dync1h1
35.47327	35.39241	35.42405	Q8VDD5;Q5SV64;Q61879;F2Z494;Q8BX	Q8VDD5	Myosin-9	Myh9
35.39879	35.4427	35.36006	E9PZ16	E9PZ16	Basement membrane-specific heparan sulfa	Hspg2
31.90455	31.9574	31.96774	Q9QXS1;E9Q3W4;F6R059;A0A0R4J218;	Q9QXS1;E9Q3W4	Plectin	Plec
38.50468	38.59432	38.51206	A0A087WR50;Q3UHL6;A0A087WS56;Q	A0A087WR50;Q3UHL6;A0	Fibronectin	Fn1
31.16039	31.12386	31.00838	E9Q616;G5E8K8	E9Q616	AHNAK nucleoprotein (desmoyokin)	Ahnak
31.13614	31.23649	31.20186	A0A286YDF5;Q69ZN7;E9Q390;A0A286Y	A0A286YDF5;Q69ZN7;E9Q	Myoferlin	Myof
33.07368	33.09428	33.06728	B7FAU9;Q8BTM8;B7FAV1;F6XC15;J3JS9	B7FAU9;Q8BTM8;B7FAV1	Filamin, alpha;Filamin-A;Filamin, alpha (Frag	Flna
24.17188	23.70767	22.35111	E9PX70;Q60847;F6WIM8	E9PX70;Q60847	Collagen alpha-1(XII) chain	Col12a1
32.85155	32.85712	32.84782	Q5SXR6;Q68FD5;F6Z1R4	Q5SXR6;Q68FD5	Clathrin heavy chain;Clathrin heavy chain 1	Cltc
31.77313	31.84595	31.81192	P19096;A0A0U1RNJ1;A0A0U1RPP5	P19096;A0A0U1RNJ1	Fatty acid synthase	Fasn
31.59779	31.6501	31.58441	Q80X90	Q80X90	Filamin-B	Flnb
31.41337	31.45807	31.42342	P26039;A2AIM2;F6S1V7;F6SX70	P26039	Talin-1	Tln1
23.83846	23.48001	21.55918	Q01149;E0CXI2	Q01149	Collagen alpha-2(I) chain	Col1a2
30.4919	30.57542	30.48232	A0A0R4J0I9;Q91ZX7;Q3U5J2;D3Z5M3	A0A0R4J0I9;Q91ZX7	Low density lipoprotein receptor-related pr	Lrp1
31.87188	31.86821	31.89735	Q9JKF1;A0A0U1RNG5;A0A0U1RPI2;A0A	Q9JKF1	Ras GTPase-activating-like protein IQGAP1	Iqgap1
30.17236	30.19601	30.07368	A0A171EBL2;E9Q555;Q3U0U2	A0A171EBL2;E9Q555	E3 ubiquitin-protein ligase RNF213	Rnf213
23.94056	24.23169	23.71816	M0QWP1;Z4YK85;A2ASQ1;Z4YJS5	M0QWP1;Z4YK85;A2ASQ1	Agrin	Agrn
33.08322	33.02333	33.02828	P26041	P26041	Moesin	Msn
21.72234	18.73066	21.89134	P11087;CON__Q862S4;Q3U962	P11087	Collagen alpha-1(I) chain	Col1a1
33.82335	33.83281	33.74535	P11499;E9Q3D6;E9PX27;D3Z1R1;E9Q0	P11499	Heat shock protein HSP 90-beta	Hsp90ab1
32.91883	32.91883	32.95913	Q7TPR4;A1BN54;O88990	Q7TPR4;A1BN54	Alpha-actinin-1;Alpha actinin 1a	Actn1
30.83846	30.94	30.95394	Q8CGC7;A0A0A6YWA4;A0A0A6YWH3	Q8CGC7	Bifunctional glutamate/proline--tRNA ligase	Eprs
32.47266	32.51559	32.46782	F8WIT2	F8WIT2	Annexin	Anxa6
31.52496	31.56184	31.58441	Q64727;A0A286YDJ4	Q64727	Vinculin	Vcl
34.25491	34.26192	34.22647	P58252	P58252	Elongation factor 2	Eef2
33.1916	33.15436	33.21784	Q01853	Q01853	Transitional endoplasmic reticulum ATPase	Vcp
30.82335	30.84595	30.77706	Q9EQK5	Q9EQK5	Major vault protein	Mvp
33.01339	33.01172	32.93473	P08113;F7C312	P08113;F7C312	Endoplasmin;Endoplasmin (Fragment)	Hsp90b1
32.63071	32.65438	32.5527	P20152;A0A0A6YWC8;A2AKJ2;P31001;	P20152;A0A0A6YWC8	Vimentin	Vim
32.65864	32.69437	32.56638	Q8VDN2;D3YYN7;Q6PIE5;Q91WH7;E9Q	Q8VDN2	Sodium/potassium-transporting ATPase sub	Atp1a1
30.18423	30.28692	30.24218	E9QAI5;B2RQC6;G3UWN2;E9QAT6;F6S	E9QAI5;B2RQC6;G3UWN2	CAD protein	Cad
25.83605	26.34435	26.35783	F2Z4A3;A0A087WRT4;A0A1L1SQU7	F2Z4A3;A0A087WRT4;A0A	FAT atypical cadherin 1	Fat1
29.509	29.55728	29.55728	Q8VHX6	Q8VHX6	Filamin-C	Flnc
30.7211	30.65438	30.90455	P14873;A2ARP8;Q9QYR6	P14873	Microtubule-associated protein 1B	Map1b
33.07368	33.02993	32.96258	P20029	P20029	78 kDa glucose-regulated protein	Hspa5
30.96774	30.6372	30.89735	A2A863;A2A864	A2A863;A2A864	Integrin beta-4;Integrin beta	Itgb4
30.64581	30.68813	30.7211	G3X9T8;G3X8Q5;G3UXG1;G3UWP5;G3U	G3X9T8;G3X8Q5	Ceruloplasmin	Cp
31.2135	31.31959	31.23649	Q9WU78	Q9WU78	Programmed cell death 6-interacting protein	Pdcd6ip
30.72923	30.74535	30.7211	Q9WTI7;Q5ND45	Q9WTI7	Unconventional myosin-Ic	Myo1c
36.0852	36.11846	36.09113	P07356;B0V2N5;B0V2N7;B0V2N8;REV_	P07356;B0V2N5;B0V2N7	Annexin A2;Annexin (Fragment)	Anxa2
33.11458	33.10992	33.06246	P27773;F6Q404	P27773	Protein disulfide-isomerase A3	Pdia3
31.04791	31.08639	31.08639	P35441;E9PWF0;Q05895	P35441	Thrombospondin-1	Thbs1
34.83752	34.67871	34.64151	P52480;A0A1L1SU37;A0A1L1SQV8;A0A	P52480	Pyruvate kinase PKM	Pkm
29.7357	29.77863	29.80039	B2RXS4;Q9QZC2;Q3UH93;Q8CJH3	B2RXS4	Plexin-B2	Plxnb2
32.42342	32.40831	32.33031	P63038;D3Z2F2;D3Z7J9	P63038	60 kDa heat shock protein, mitochondrial	Hspd1
32.29789	32.22216	32.19894	P09103;E9Q8G8	P09103	Protein disulfide-isomerase	P4hb
30.47267	30.54812	30.4334	Q3V117;Q91V92;Q3TS02	Q3V117;Q91V92	ATP-citrate synthase	Acly
30.87555	30.80809	30.81574	E9Q5G3;A0A1L1SRP4	E9Q5G3;A0A1L1SRP4	Kinesin-like protein KIF23;Kinesin-like protei	Kif23
33.31689	33.30334	33.26192	P63017;Q504P4;D3Z5E2	P63017;Q504P4	Heat shock cognate 71 kDa protein	Hspa8
30.91883	30.92592	30.98142	Q8BHN3	Q8BHN3	Neutral alpha-glucosidase AB	Ganab
30.00838	30.09899	30.08639	Q8BMJ2	Q8BMJ2	Leucine--tRNA ligase, cytoplasmic	Lars
35.98269	35.9656	35.93077	P48036;A0A0G2JGQ0	P48036	Annexin A5	Anxa5
27.98142	28.02433	28.20303	A2AN08;F6SSP6;Z4YMA7;Z4YLP1	A2AN08	E3 ubiquitin-protein ligase UBR4	Ubr4
32.57542	32.66501	32.62636	O35639;Q3TET3;A0A0G2JGL7;A0A0G2J	O35639	Annexin A3	Anxa3
29.89591	29.96774	29.94	Q8VDM4;E9Q2S8	Q8VDM4	26S proteasome non-ATPase regulatory sub	Psmd2
31.98482	32.04791	31.9746	P07901;B7ZC50;A2A6A2;B7ZC49	P07901	Heat shock protein HSP 90-alpha	Hsp90aa1
29.94	29.98142	30.11148	Q61768;E9QAK5;P33175	Q61768;E9QAK5	Kinesin-1 heavy chain;Kinesin-like protein (F	Kif5b
30.67976	30.66289	30.64581	O55143;Q8R429;B1ATS4;B1ATS5;E9Q5	O55143	Sarcoplasmic/endoplasmic reticulum calciu	Atp2a2
34.98057	34.91972	34.89284	P10107;E9QAQ7;A2BH40	P10107	Annexin A1	Anxa1
30.91883	30.9746	30.93298	P42932;H3BL49;H3BJB6;H3BKG2;H3BL	P42932;H3BL49	T-complex protein 1 subunit theta	Cct8
28.98414	28.93157	29.1795	P48678;D3YUF7	P48678	Prelamin-A/C	Lmna
32.01505	32.03157	31.94698	P57780;A0A1L1SV25;E9Q2W9;A0A1L1S	P57780;A0A1L1SV25;E9Q	Alpha-actinin-4;Alpha-actinin-4 (Fragment)	Actn4
29.56275	29.71129	29.64753	Q64514;A0A087WRC0;Q05DJ9;A0A087	Q64514;A0A087WRC0	Tripeptidyl-peptidase 2	Tpp2
28.10649	28.14589	28.26249	Q7TQD7;P46735;E9QNH6;E9Q580;D3Y	Q7TQD7;P46735;E9QNH6	Myo1b protein;Unconventional myosin-Ib	Myo1b
30.80039	30.83092	30.69644	Q8BGQ7	Q8BGQ7	Alanine--tRNA ligase, cytoplasmic	Aars
29.96774	30.02168	30.02168	Q8BU30;E9Q866;F6Q6R1	Q8BU30	Isoleucine--tRNA ligase, cytoplasmic	Iars
31.7211	31.79266	31.79653	Q9D379;E9PWK1;F6YTS6;D3Z4M3	Q9D379;E9PWK1	Epoxide hydrolase 1;Epoxide hydrolase	Ephx1
28.41135	28.31959	28.2803	O35379;E9Q1I5;D3YZY1	O35379	Multidrug resistance-associated protein 1	Abcc1
31.39305	31.36724	31.34096	P38647	P38647	Stress-70 protein, mitochondrial	Hspa9
31.50142	31.33565	31.45807	P70168	P70168	Importin subunit beta-1	Kpnb1
30.93298	30.91171	30.78488	Q61739	Q61739	Integrin alpha-6	Itga6
31.17831	31.27031	31.16639	A0A1C7CYV0;Q99K51;B1AX58;Q61233;	A0A1C7CYV0;Q99K51;B1A	Plastin-3 (Fragment);Plastin-3	Pls3
30.76129	30.80039	30.71293	Q02053;P31254	Q02053	Ubiquitin-like modifier-activating enzyme 1	Uba1
32.45317	32.38792	32.40831	Q03265;D3Z6F5;D6RJ16	Q03265;D3Z6F5	ATP synthase subunit alpha, mitochondrial;	Atp5a1
34.09899	34.01837	33.99328	P17182;Q6PHC1;B0QZL1;B1ARR7;B1AR	P17182;Q6PHC1	Alpha-enolase	Eno1
30.55728	30.57542	30.66289	Q3U2G2;Q61316	Q3U2G2;Q61316	Heat shock 70 kDa protein 4	Hspa4
29.8549	29.94	29.96774	Q3V3R1	Q3V3R1	Monofunctional C1-tetrahydrofolate syntha	Mthfd1l
30.27586	30.4334	30.34096	Q60864	Q60864	Stress-induced-phosphoprotein 1	Stip1
29.51465	29.54995	29.54075	Q78PY7;Q3TJ56;E9Q3E9	Q78PY7;Q3TJ56	Staphylococcal nuclease domain-containing	Snd1
24.87643	26.10149	25.89273	Q8BMK4	Q8BMK4	Cytoskeleton-associated protein 4	Ckap4
29.55728	29.69312	29.66289	Q9Z1Q9;G3UY93;G3UZ22;G3UYW2;G3U	Q9Z1Q9;G3UY93	Valine--tRNA ligase;Valine--tRNA ligase (Frag	Vars
30.46295	30.52962	30.48232	G5E829;A0A1W2P7C7;Q3UHH0;F8WHB	G5E829	Plasma membrane calcium-transporting ATP	Atp2b1
31.80424	31.72517	31.78097	P80314;A0A1W2P7B7;A0A1W2P828;A0	P80314	T-complex protein 1 subunit beta	Cct2
29.45317	29.62331	29.65608	Q62351;Q8C872	Q62351;Q8C872	Transferrin receptor protein 1	Tfrc

28.97596	28.96774	29.06599	Q7TMB8;A0A0R4J119;A0A0U1RQ05	Q7TMB8;A0A0R4J119;A0A	Cytoplasmic FMR1-interacting protein 1	Cyfip1
19.34082	21.82299	20.49717	Q99K41	Q99K41	EMILIN-1	Emilin1
29.73085	29.68311	29.79576	Q9WVK4	Q9WVK4	EH domain-containing protein 1	Ehd1
33.09428	33.08797	32.98142	P05064;A6ZI44;D3YWI1;Q9CPQ9;A6ZI4	P05064;A6ZI44;D3YWI1;Q	Fructose-bisphosphate aldolase A;Fructose-I	Aldoa
31.50142	31.58889	31.51089	P35564	P35564	Calnexin	Canx
36.55556	36.53543	36.53832	P63260;B1ATY1;G3UYG0	P63260	Actin, cytoplasmic 2	Actg1
28.56456	28.71947	28.51277	Q02819;A0A1C7CYU3;A0A1B0GR41;A0A	Q02819;A0A1C7CYU3;A0A	Nucleobindin-1;Nucleobindin 1, isoform CRA	Nucb1
27.68646	27.63201	27.80809	Q5SWU9;V9GWS0;V9GXY1;D3Z0B3	Q5SWU9	Acetyl-CoA carboxylase 1	Acaca
28.86821	28.87408	28.86821	Q8CIE6;F8WHL2;F6XJN3	Q8CIE6;F8WHL2	Coatomer subunit alpha	Copa
29.71293	29.71293	29.62506	A0A0R4J0Z1;P08003	A0A0R4J0Z1;P08003	Protein disulfide-isomerase A4	Pdia4
29.13858	29.22159	29.36202	P43406;A2AKI5;B7ZCI9;F6W6Q6	P43406;A2AKI5	Integrin alpha-V	Itgav
22.94235	22.40665	23.417	P97449;A0A0U1RNS3	P97449	Aminopeptidase N	Anpep
29.48232	29.39509	29.50332	Q6P5E4;G3UYG7;G3UZU8;G3UY73;G3U	Q6P5E4	UDP-glucose:glycoprotein glucosyltransfera	Uggt1
30.09899	30.23078	30.16039	Q9QUR6	Q9QUR6	Prolyl endopeptidase	Prep
31.81192	31.68813	31.69229	D3Z2H9;D3YVR0	D3Z2H9	Tropomyosin 3, related sequence 7	Tpm3-rs7
28.87701	28.92309	28.75175	E9PVA8	E9PVA8	eIF-2-alpha kinase activator GCN1	Gcn1
32.27309	32.31149	32.26472	P09411;S4R2M7	P09411;S4R2M7	Phosphoglycerate kinase 1;Phosphoglycerat	Pgk1
31.87921	31.81955	31.79266	P40124;B1ARS0	P40124	Adenylyl cyclase-associated protein 1	Cap1
31.45317	31.41337	31.29789	P80313;A0A0N4SV00;A0A0N4SUI8	P80313;A0A0N4SV00	T-complex protein 1 subunit eta	Cct7
29.42742	29.4746	29.34308	Q61699;E9Q0U7;D3Z3I9;D3Z027;A0A0J	Q61699;E9Q0U7	Heat shock protein 105 kDa	Hsph1
28.29789	28.17474	28.60045	Q62261;A0A0A0MQG2	Q62261;A0A0A0MQG2	Spectrin beta chain, non-erythrocytic 1;Spec	Sptbn1
29.83244	29.89735	29.69146	Q62470;F6UD16	Q62470	Integrin alpha-3	Itga3
29.51841	29.54628	29.53148	Q922D8;A0A1W2P733;A0A1W2P7L5	Q922D8;A0A1W2P733	C-1-tetrahydrofolate synthase, cytoplasmic	Mthfd1
31.64581	31.73731	31.65864	Q9D8E6	Q9D8E6	60S ribosomal protein L4	Rpl4
31.50616	31.55728	31.46295	O88342;A0A0J9YU05	O88342	WD repeat-containing protein 1	Wdr1
27.96499	28.07623	28.11148	P16546;A3KGU5;A3KGU7;E9Q447;A3KG	P16546;A3KGU5;A3KGU7;	Spectrin alpha chain, non-erythrocytic 1	Sptan1
31.06728	31.01505	31.0544	P80316;E0CZA1	P80316	T-complex protein 1 subunit epsilon	Cct5
28.61807	28.61105	28.70306	E9Q586;E9Q3M3;O08788;D3YX34;D3Z2	E9Q586;E9Q3M3;O08788;	Dynactin subunit 1	Dctn1
30.96086	30.90455	30.86821	P11983;F2Z483	P11983	T-complex protein 1 subunit alpha	Tcp1
30.61105	30.64581	30.62854	P40142;A0A286YE28;E0CY51	P40142;A0A286YE28	Transketolase;Transketolase (Fragment)	Tkt
31.32496	31.27586	31.26472	P80315;G5E839;G3UYW5;G3UXF3;G3U	P80315;G5E839	T-complex protein 1 subunit delta	Cct4
31.02168	31.11768	31.0414	P80318;E9Q133;Q3U0I3;F6Q609;F6ZVG	P80318;E9Q133;Q3U0I3;F	T-complex protein 1 subunit gamma	Cct3
31.11768	31.0544	31.02168	Q3TFD0;Q9CZN7	Q3TFD0;Q9CZN7	Serine hydroxymethyltransferase	Shmt2
31.19601	31.09899	31.17236	Q61598;A0A1Y7VL99;A0A1Y7VLG4	Q61598;A0A1Y7VL99	Rab GDP dissociation inhibitor beta;Rab GDI	Gdi2
29.37451	29.35993	29.23991	Q8BKC5	Q8BKC5	Importin-5	Ipo5
29.71293	29.77706	29.69312	Q8C4U8;O35474;Q8C8K0	Q8C4U8;O35474;Q8C8K0	EGF-like repeat and discoidin I-like domain-c	Edil3
30.04791	30.16039	30.07368	Q91YQ5;A0A0N4SUJ8	Q91YQ5	Dolichyl-diphosphooligosaccharide--protein	Rpn1
30.14831	30.12386	30.11148	Q9CWJ9	Q9CWJ9	Bifunctional purine biosynthesis protein PUF	Atic
30.42342	30.59335	30.45317	Q9WV91	Q9WV91	Prostaglandin F2 receptor negative regulato	Ptgfrn
29.95394	30.07368	30.02168	F8VPU2;E9Q805	F8VPU2	FERM, ARHGEF and pleckstrin domain-conta	Farp1
31.49667	31.5527	31.62854	P09055	P09055	Integrin beta-1	Itgb1
29.82031	29.81268	29.84296	Q00612;A3KG36;G3UWD6;P97324;REV_	Q00612;A3KG36	Glucose-6-phosphate 1-dehydrogenase X;Gl	G6pdx
30.6372	30.76129	30.74535	Q04857	Q04857	Collagen alpha-1(VI) chain	Col6a1
27.80193	27.90885	27.82031	Q4FE56;P70398;E9PWA9;F8VPU6;G3UY	Q4FE56;P70398	Probable ubiquitin carboxyl-terminal hydrola	Usp9x
30.91883	30.99496	31.09899	Q6IRU2	Q6IRU2	Tropomyosin alpha-4 chain	Tpm4
31.00838	31.0414	30.96774	Q76MZ3;G3UWL2;G3UXQ1	Q76MZ3	Serine/threonine-protein phosphatase 2A 6	Ppp2r1a
29.21928	29.30661	29.23306	Q8C129	Q8C129	Leucyl-cystinyl aminopeptidase	Lnpep
28.88285	28.76762	28.88577	Q8VEK3	Q8VEK3	Heterogeneous nuclear ribonucleoprotein U	Hnrnpu
29.77077	29.89591	29.74053	Q9D0I9	Q9D0I9	Arginine--tRNA ligase, cytoplasmic	Rars
27.391	27.32389	27.43141	Q9QY06	Q9QY06	Unconventional myosin-IXb	Myo9b
28.34096	28.31095	28.27142	A2AS98;P28660;Q8K1X4	A2AS98;P28660	Nck-associated protein 1	Nckap1
23.06579	23.51653	23.17798	E9Q8I0;P06909;D6RGQ0;E9Q8H9;A0A0	E9Q8I0;P06909;D6RGQ0;E	Complement factor H	Cfh
33.86083	33.88831	33.81383	P05213	P05213	Tubulin alpha-1B chain	Tuba1b
34.38921	34.33031	34.31013	P10126;D3YZ68;D3Z3I8;P62631	P10126;D3YZ68;D3Z3I8;P	Elongation factor 1-alpha 1;Elongation facto	Eef1a1
30.80039	30.82335	30.71293	P12382;A0A1W2P7T1	P12382	ATP-dependent 6-phosphofructokinase, live	Pfkl
29.34096	29.35153	29.28472	P46471;Q8BVQ9	P46471;Q8BVQ9	26S proteasome regulatory subunit 7	Psmc2
29.67303	29.64753	29.65608	P50516;D3Z1B9;D3YWH3;D3YZ23	P50516	V-type proton ATPase catalytic subunit A	Atp6v1a
33.67346	33.63071	33.66289	P68372;Q9D6F9	P68372;Q9D6F9	Tubulin beta-4B chain;Tubulin beta-4A chain	Tubb4b;Tubb4a
29.54259	29.64924	29.4919	Q3TXS7;J3QN38;D6RGR5	Q3TXS7	26S proteasome non-ATPase regulatory sub	Psmd1
29.15798	29.22619	29.11644	Q3TZZ7;A0A1Y7VM39;A0A1Y7VNA1	Q3TZZ7	Extended synaptotagmin-2	Esyt2
30.67976	30.67976	30.64581	Q9CZD3	Q9CZD3	Glycine--tRNA ligase	Gars
29.74535	29.80501	29.80501	Q9D0R2;Q8BLY2	Q9D0R2	Threonine--tRNA ligase, cytoplasmic	Tars
29.57001	29.59156	29.72923	Q9JKR6;F6TRP3;A0A1L1SQ34;E0CYZ2	Q9JKR6	Hypoxia up-regulated protein 1	Hyou1
28.09648	27.96499	27.87994	A0A087WPL5;E9QNN1;O70133;Q3UR42	A0A087WPL5;E9QNN1;O7	ATP-dependent RNA helicase A	Dhx9
27.39917	27.07112	27.38278	E9Q035;P47758;F7CJN9;F7BAE9;A0A1W	E9Q035	Predicted gene 20425	Gm20425
31.37244	31.36724	31.2535	P06745;A0A0U1RP97;A0A0U1RQ72;A0A	P06745	Glucose-6-phosphate isomerase	Gpi
31.72517	31.65864	31.63288	P08752;A0A0A6YWA9;B2RSH2;A0A0A6	P08752;A0A0A6YWA9	Guanine nucleotide-binding protein G(i) sub	Gnai2
31.89735	31.99159	32.01172	P10852;A0A0U1RP98;A0A0U1RPL8;A0A	P10852	4F2 cell-surface antigen heavy chain	Slc3a2
31.6372	31.61982	31.57091	P14211	P14211	Calreticulin	Calr
30.28692	30.39305	30.40324	P37040;Q05DV1;E9Q997;E9PVT9;F6R7H	P37040;Q05DV1	NADPH--cytochrome P450 reductase	Por
28.71293	28.81726	28.91171	P37889	P37889	Fibulin-2	Fbln2
27.98685	28.14589	28.08132	P47856;Q9Z2Z9;D3YYD9;D3YYE0	P47856	Glutamine--fructose-6-phosphate aminotran	Gfpt1
30.93298	30.98821	30.90455	P60843	P60843	Eukaryotic initiation factor 4A-I	Eif4a1
32.60664	32.65864	32.74134	P63101;D3YXN6;D3YXF4;D3YW45	P63101	14-3-3 protein zeta/delta	Ywhaz
28.46295	28.5969	28.67976	Q3UPL0;S4R2A9;S4R192;S4R256;S4R1T	Q3UPL0;S4R2A9	Protein transport protein Sec31A;Protein tra	Sec31a
28.82335	28.89157	28.74855	Q3UW53;E9PYV4;D3Z233;D3YYZ9	Q3UW53;E9PYV4	Protein Niban	Fam129a
29.14831	29.14102	29.17474	Q61508;D3Z0U9;D3Z038;D3YV22;D3YZ	Q61508	Extracellular matrix protein 1	Ecm1
30.09899	30.20769	30.12386	Q922B2;Q8BJY7	Q922B2	Aspartate--tRNA ligase, cytoplasmic	Dars
29.69312	29.80501	29.79421	Q99KI0	Q99KI0	Aconitate hydratase, mitochondrial	Aco2
28.62854	28.7162	28.65608	E9QB02;Q68FL6;F6W0G8	E9QB02;Q68FL6	Methionine--tRNA ligase, cytoplasmic	Mars
29.81268	29.91171	29.86821	O08529	O08529	Calpain-2 catalytic subunit	Capn2
28.56456	28.52028	28.60045	O55029	O55029	Coatomer subunit beta	Copb2
31.70058	31.74535	31.70471	P19324;A0A140LHR4;A0A140LHK0;A0A	P19324	Serpin H1	Serpinh1
29.86083	29.91171	29.94	P46935;Q3V335;V9GWV8;V9GXK3;V9G	P46935	E3 ubiquitin-protein ligase NEDD4	Nedd4
31.33031	31.31959	31.33031	P47738;A0A0G2JEU1;A0A0G2JFQ0;A0A	P47738;A0A0G2JEU1	Aldehyde dehydrogenase, mitochondrial	Aldh2
32.622	32.52028	32.49667	P56480	P56480	ATP synthase subunit beta, mitochondrial	Atp5b
31.75732	31.76525	31.64151	P97351	P97351	40S ribosomal protein S3a	Rps3a
31.48232	31.45807	31.57993	P97429;A0A0N4SW89;D3Z0S1;A0A0N4	P97429;A0A0N4SW89;D3	Annexin A4;Annexin (Fragment)	Anxa4

29.87555	29.89446	29.87262	Q61553;A0A0G2JDU7;F7BDR1	Q61553;A0A0G2JDU7	Fascin	Fscn1
29.47073	29.43539	29.46684	Q6ZQ38;D3YWC5	Q6ZQ38	Cullin-associated NEDD8-dissociated protein	Cand1
28.97869	28.78644	28.81726	Q9JJ28	Q9JJ28	Protein flightless-1 homolog	Flii
29.08639	29.17236	29.14831	E9QPE2;E9QN12;P05622	E9QPE2;E9QN12;P05622	Platelet-derived growth factor receptor beta	Pdgfrb
28.14589	27.98142	27.75175	G3X922;D4AFX7;A0A087WRC9;A0A087	G3X922;D4AFX7	DnaJ heat shock protein family (Hsp40) mem	Dnajc13
24.94949	24.33074	26.07316	P01902;F6TIX5;Q9BCZ1;A7VMS2;Q3161	P01902	H-2 class I histocompatibility antigen, K-D al	H2-K1
28.16039	28.31959	28.36202	P17879;Q61696	P17879;Q61696	Heat shock 70 kDa protein 1B;Heat shock 70	Hspa1b;Hspa1a
34.09113	34.09899	34.03485	P21956	P21956	Lactadherin	Mfge8
32.05116	32.08955	32.13001	P62702;V9GWY0	P62702	40S ribosomal protein S4, X isoform	Rps4x
31.6501	31.61982	31.59335	P62908;D3YV43;A0A140LI77	P62908;D3YV43;A0A140LI	40S ribosomal protein S3	Rps3
28.66966	28.75494	28.71947	P98063;E9Q5B9;Q9WVM6;G3X9F5;Q62	P98063	Bone morphogenetic protein 1	Bmp1
28.98685	28.97049	29.01903	Q61398;D3YUE2;A0A0G2JE29;D3YZE3;D	Q61398;D3YUE2;A0A0G2J	Procollagen C-endopeptidase enhancer 1	Pcolce
30.08639	30.16039	30.08639	Q62167;P16381;Q62095;Q3V086;Q614	Q62167;P16381;Q62095	ATP-dependent RNA helicase DDX3X;Putativ	Ddx3x;D1Pas1
26.83846	27.10149	26.99766	Q7TPV4	Q7TPV4	Myb-binding protein 1A	Mybbp1a
26.93157	27.43936	27.41538	Q7TT50;A0A1Y7VKD5;A0A1Y7VLI0;A0A1	Q7TT50	Serine/threonine-protein kinase MRCK beta	Cdc42bpb
29.62506	29.52589	29.51653	Q8C605;Q9WUA3;D3YUA3;F6YL81	Q8C605;Q9WUA3	ATP-dependent 6-phosphofructokinase;ATP-	Pfkp
29.40324	29.45317	29.40933	Q8R422	Q8R422	CD109 antigen	Cd109
30.38278	30.27586	30.44332	Q91VI7;A0A1B0GSG5;A0A1B0GRG4;A0A	Q91VI7;A0A1B0GSG5	Ribonuclease inhibitor	Rnh1
31.40324	31.49667	31.35678	Q9R118	Q9R118	Serine protease HTRA1	Htra1
27.85638	27.82031	27.86231	A0A0U1RNK7;A2A9M5;E9PX48;A2A9M4	A0A0U1RNK7;A2A9M5;E9	Dedicator of cytokinesis protein 7	Dock7
31.20769	31.21928	31.15436	A0A2C9F2D2;Q07076;A0A286YCW4	A0A2C9F2D2;Q07076	Annexin A7	Anxa7
29.50711	29.75334	29.52216	E9PWE8;Q3TT92;Q62188;D3YUS0;D3Z5	E9PWE8;Q3TT92;Q62188	Dihydropyrimidinase-related protein 3	Dpysl3
22.70978	22.239	20.90976	P08121;A0A087WPJ5;A0A087WPS3	P08121	Collagen alpha-1(III) chain	Col3a1
31.47267	31.35153	31.4334	P12970;A0A140T8L1;A0A140T8L3;V9GX	P12970;A0A140T8L1;A0A1	60S ribosomal protein L7a;Ribosomal protei	Rpl7a;Rpl7a-ps3
27.80809	28.00838	27.91456	P24270;A2AL20	P24270	Catalase	Cat
31.7211	31.77313	31.69644	P27659;A0A087WQK0;A0A087WNS0;Q	P27659	60S ribosomal protein L3	Rpl3
30.12386	30.13614	30.07368	Q3TGU7;P50580	Q3TGU7;P50580	Proliferation-associated 2G4;Proliferation-as	Pa2g4
33.13461	33.13155	33.02993	Q564E2;P06151;A0A1B0GSX0;A0A1B0G	Q564E2;P06151;A0A1B0G	L-lactate dehydrogenase;L-lactate dehydrog	Ldha
31.31959	31.44825	31.41337	Q61753;F6ZSB7	Q61753	D-3-phosphoglycerate dehydrogenase	Phgdh
27.84446	27.65267	27.48616	Q8BH64;D3Z7U7	Q8BH64	EH domain-containing protein 2	Ehd2
30.42342	30.50142	30.45317	Q8BP47	Q8BP47	Asparagine--tRNA ligase, cytoplasmic	Nars
29.66628	29.61105	29.72598	Q8BT60;Q9D6C8;Q3UYN2;Q9Z140;Q8B	Q8BT60	Copine-3	Cpne3
29.00571	29.08132	29.08385	Q99104;D3YZ62;D3Z4J3;F6TDE5;B8JK0	Q99104;D3YZ62;D3Z4J3	Unconventional myosin-Va	Myo5a
27.43141	27.37451	27.391	Q9EPU0	Q9EPU0	Regulator of nonsense transcripts 1	Upf1
29.70141	29.57362	29.61982	Q9EQH3	Q9EQH3	Vacuolar protein sorting-associated protein	Vps35
31.0927	31.19013	31.14224	Q9QZF2	Q9QZF2	Glypican-1	Gpc1
28.57181	28.75175	28.70306	Q9Z110;D3Z0B4;H3BKJ8;H3BLE8;H3BKK	Q9Z110	Delta-1-pyrroline-5-carboxylate synthase	Aldh18a1
28.77077	28.98142	28.88577	A2A7S7;Q91WQ3;F6VXZ2	A2A7S7;Q91WQ3	Tyrosine--tRNA ligase;Tyrosine--tRNA ligase,	Yars
32.19601	32.29789	32.29515	E9QPX1;P39061;A0A1W2P6N8;D3Z556	E9QPX1;P39061	Collagen alpha-1(XVIII) chain	Col18a1
28.60752	28.62854	28.76762	P09405	P09405	Nucleolin	Ncl
29.39917	29.4174	29.461	P11688	P11688	Integrin alpha-5	Itga5
31.78877	31.72517	31.72923	P14148;F6XI62	P14148;F6XI62	60S ribosomal protein L7;60S ribosomal prot	Rpl7
29.24445	29.4174	29.258	P16460	P16460	Argininosuccinate synthase	Ass1
29.73247	29.80809	29.68144	P26443;F7CFA5	P26443	Glutamate dehydrogenase 1, mitochondrial	Glud1
31.2814	31.16639	31.13001	P80317	P80317	T-complex protein 1 subunit zeta	Cct6a
28.57542	28.48616	28.64924	Q60715	Q60715	Prolyl 4-hydroxylase subunit alpha-1	P4ha1
29.80039	29.78332	29.73085	Q61503	Q61503	5-nucleotidase	Nt5e
30.35153	30.33031	30.41337	Q8C483;P26638;A2AFS0;A2AFS1	Q8C483;P26638;A2AFS0	Serine--tRNA ligase, cytoplasmic;Serine--tRN	Sars
28.83244	28.69644	28.78644	Q8CI51;D9J2Z9;D9J300;D9J301;D9J302;	Q8CI51;D9J2Z9;D9J300;D	PDZ and LIM domain protein 5;ENH isoform	Pdlim5
30.80809	30.81574	30.86083	Q99JY9;A0A087WRA1;A0A087WQ14;A	Q99JY9	Actin-related protein 3	Actr3
30.41337	30.52028	30.52028	Q9DCD0	Q9DCD0	6-phosphogluconate dehydrogenase, decarb	Pgd
28.50142	28.52402	28.62506	Q9EP69	Q9EP69	Phosphatidylinositide phosphatase SAC1	Sacm1l
28.99226	28.99496	28.99766	Q9ER72;A0A140LIB6;A0A140LII7	Q9ER72	Cysteine--tRNA ligase, cytoplasmic	Cars
29.47846	29.57181	29.56456	Q9QUJ7	Q9QUJ7	Long-chain-fatty-acid--CoA ligase 4	Acsl4
28.24898	28.37036	28.41135	Q9WTM5;A0A1B0GSR4;A0A1B0GRW3;	Q9WTM5	RuvB-like 2	Ruvbl2
29.88867	29.76762	29.85341	Q9WVM1;E9Q9N9;E9Q851;F6WD40	Q9WVM1	Rac GTPase-activating protein 1	Racgap1
29.13614	29.13369	29.04009	G3UVV4;P17710;D3YYR4;D3Z365;D3Z1	G3UVV4;P17710	Hexokinase 1, isoform CRA_f;Hexokinase-1	Hk1
31.51559	31.36202	31.36202	P10923;F8WIP8;D3Z4N2;D3Z513;D3Z11	P10923;F8WIP8	Osteopontin	Spp1
34.42091	34.29652	34.19014	P16858;A0A0A0MQF6;S4R1W1;A0A1D	P16858;A0A0A0MQF6;S4	Glyceraldehyde-3-phosphate dehydrogenase	Gapdh
29.59335	29.64924	29.54444	P29341;Q9D4E6;Q62029;F6ZAX1;Q8C7	P29341;Q9D4E6	Polyadenylate-binding protein 1;Polyadenyla	Pabpc1;Pabpc6
31.12386	31.10525	31.0544	Q07797;E9Q5X5	Q07797	Galectin-3-binding protein	Lgals3bp
28.72923	28.76762	28.89157	Q3T9X3;F8WIV5;P39054;Q3TCR7;G3X9	Q3T9X3;F8WIV5;P39054;	Dynamin-2	Dnm2;D
28.78644	28.71947	28.66289	Q4VA93;P20444;Q3TQ39;P68404;Q2NK	Q4VA93;P20444	Protein kinase C;Protein kinase C alpha type	Prkca
24.417	25.01478	23.59833	Q60790;D6RFE0	Q60790	Ras GTPase-activating protein 3	Rasa3
28.61456	28.7357	28.69644	Q80UE5;O70318;Q80UE4;A0A1W2P6I5;	Q80UE5;O70318;Q80UE4;	Band 4.1-like protein 2	Epb41l2
28.27586	28.4073	28.37036	Q8K009;D3Z6B9	Q8K009;D3Z6B9	Mitochondrial 10-formyltetrahydrofolate de	Aldh1l2
29.98142	30.14831	30.02168	Q9DCL9;D3Z6P1;D6RCU8	Q9DCL9	Multifunctional protein ADE2	Paics
29.63547	29.76288	29.72273	Q9EPL8;A0A140LHD0	Q9EPL8	Importin-7	Ipo7
28.57181	28.64924	28.28472	Q9JIF7	Q9JIF7	Coatomer subunit beta	Copb1
28.94	28.95394	29.14589	O70475;D3YXP9	O70475	UDP-glucose 6-dehydrogenase	Ugdh
27.04009	26.95394	27.2535	P21447;P21440;A0A0G2JDM4;F6SGP4	P21447	Multidrug resistance protein 1A	Abcb1a
31.61544	31.61982	31.55728	P48962;Q3V132	P48962	ADP/ATP translocase 1	Slc25a4
30.33031	30.28692	30.18423	P97384;D3Z7U0	P97384;D3Z7U0	Annexin A11;Annexin	Anxa11
28.22619	28.04009	28.21697	Q00519;G3X982;Q3TYQ9	Q00519	Xanthine dehydrogenase/oxidase	Xdh
29.46879	29.45317	29.57001	Q3U7R1;A0A1W2P784	Q3U7R1	Extended synaptotagmin-1	Esyt1
30.00838	30.04791	30.02168	Q3U9N4;P28798;H3BJE0;H3BJ90;H3BL	Q3U9N4;P28798;H3BJE0	Granulins;Granulins (Fragment)	Grn
31.79653	31.88649	31.73731	Q62465	Q62465	Synaptic vesicle membrane protein VAT-1 h	Vat1
28.75175	28.80193	28.78332	Q8VCQ8;E9QA16;S4R1T7;E9Q0M9;D3Z	Q8VCQ8;E9QA16;S4R1T7;	Caldesmon 1	Cald1
30.85341	30.83092	30.87555	Q9D8N0	Q9D8N0	Elongation factor 1-gamma	Eef1g
30.03486	30.14831	30.00838	E9PYT3;Q91YH5;Q8BH66	E9PYT3;Q91YH5	Atlastin-3	Atl3
29.2007	29.13369	28.89157	E9QLA5;Q0GNC1;A0A1Y7VM80	E9QLA5;Q0GNC1;A0A1Y7	Inverted formin-2;Inverted formin-2 (Fragm	Inf2
29.87994	29.89735	29.73892	F7DBB3;Q3URZ6	F7DBB3	AHNAK nucleoprotein 2 (Fragment)	Ahnak2
28.76446	27.75812	28.82941	P10493	P10493	Nidogen-1	Nid1
32.48712	32.42842	32.52262	P17751;H7BXC3	P17751;H7BXC3	Triosephosphate isomerase	Tpi1
28.99226	29.00035	28.96774	P26040	P26040	Ezrin	Ezr
32.25912	32.2535	32.20478	P35700;B1AXW5;B1AXW6;B1AXW4	P35700;B1AXW5;B1AXW6	Peroxiredoxin-1;Peroxiredoxin-1 (Fragment)	Prdx1
31.78488	31.75732	31.88285	P62259;D6REF3;F6WA09	P62259;D6REF3;F6WA09	14-3-3 protein epsilon;14-3-3 protein epsilo	Ywhae
29.18423	29.27586	29.43738	P62334	P62334	26S proteasome regulatory subunit 10B	Psmc6

29.1866	29.13124	29.22849	Q03145;E9PUK8;A2BDQ4;G1K381;Q8CE	Q03145	Ephrin type-A receptor 2	Epha2
27.91456	28.07623	27.99766	Q3U1J4	Q3U1J4	DNA damage-binding protein 1	Ddb1
29.68646	29.64924	29.77706	Q61024;D3Z0C2;D3YYR0;D3Z028	Q61024	Asparagine synthetase [glutamine-hydrolyzi	Asns
29.11148	29.15798	29.23535	Q8BG32;G3UYH2;G3UYI4;G3UYL3;G3UV	Q8BG32	26S proteasome non-ATPase regulatory subu	Psmd11
28.50522	28.34096	28.4746	Q8CGK3	Q8CGK3	Lon protease homolog, mitochondrial	Lonp1
29.81115	29.70965	29.82487	Q8K2B3;A0A1Y7VJ55	Q8K2B3	Succinate dehydrogenase [ubiquinone] flavo	Sdha
29.07623	29.21928	29.0296	Q8R180;A0A1Y7VJM4;A0A1Y7VNF4;Q8	Q8R180	ERO1-like protein alpha	Ero1a
30.38278	30.44332	30.35153	Q91Z25;Q9WV32;F6VVE6;F6THG2;D3Z6	Q91Z25;Q9WV32	Actin-related protein 2/3 complex subunit;A	Arpc1b
30.31959	30.33031	30.34096	Q99JR5;H3BJ97;H3BKM8	Q99JR5;H3BJ97	Tubulointerstitial nephritis antigen-like	Tinagl1
29.73085	29.66458	29.61456	Q9R1P4;A0A1B0GS70	Q9R1P4;A0A1B0GS70	Proteasome subunit alpha type-1;Proteasom	Psma1
29.26696	29.42142	29.37244	Q9WUM4;E9PX03;E9PZJ0;E9PVJ1;B9EIZ	Q9WUM4	Coronin-1C	Coro1c
27.2803	27.26249	27.54628	A0A1L1SV73;Q8BY87	A0A1L1SV73;Q8BY87	Ubiquitin carboxyl-terminal hydrolase 47	Usp47
27.71947	28.28912	28.03486	E9PYK3	E9PYK3	Poly [ADP-ribose] polymerase	Parp4
28.16997	28.00838	28.09648	E9Q668;O08665;O88632	E9Q668;O08665	Semaphorin-3A (Fragment);Semaphorin-3A	Sema3a
28.3452	28.31095	28.53519	F8VQC1;E9Q740	F8VQC1;E9Q740	Signal recognition particle subunit SRP72	Srp72
27.81421	27.78955	27.83244	F8WIJ0;Q9JIS8;A2AGJ9;Q6P6P5;Q3V0N8	F8WIJ0;Q9JIS8	Solute carrier family 12 member 4	Slc12a4
29.16039	29.32603	29.2957	O88844;A0A087WPT4;A0A087WRS9;D3	O88844	Isocitrate dehydrogenase [NADP] cytoplasm	Idh1
28.48616	28.49762	28.21697	P01900;P01895	P01900;P01895	H-2 class I histocompatibility antigen, D-D al	H2-D1
28.76762	28.66628	28.82941	P05201;F7ALS6	P05201	Aspartate aminotransferase, cytoplasmic	Got1
30.39305	30.44332	30.40324	P05202	P05202	Aspartate aminotransferase, mitochondrial	Got2
31.67976	31.60222	31.59779	P08249;A0A0G2JF23;A0A0G2JGY4	P08249	Malate dehydrogenase, mitochondrial	Mdh2
29.44332	29.65438	29.67472	P10649;A2AE89;F6WHQ7;A2AE91;D3YV	P10649;A2AE89;F6WHQ7	Glutathione S-transferase Mu 1;Glutathione	Gstm1
30.61982	30.61982	30.60222	P47962;D3YYV8	P47962;D3YYV8	60S ribosomal protein L5;60S ribosomal prot	Rpl5
30.06085	30.11148	30.14831	P62137	P62137	Serine/threonine-protein phosphatase PP1-a	Ppp1ca
31.5527	31.55728	31.59335	P68040	P68040	Receptor of activated protein C kinase 1	Rack1
29.07623	29.11148	29.10899	Q3TW96;A0A1B0GST7;A0A1B0GR64;A0	Q3TW96	UDP-N-acetylhexosamine pyrophosphorylas	Uap1l1
31.9574	31.91528	31.86821	Q64337;D3YZJ1;F6VD69	Q64337;D3YZJ1	Sequestosome-1	Sqstm1
25.84805	25.98685	26.14492	Q8BKG3	Q8BKG3	Inactive tyrosine-protein kinase 7	Ptk7
30.23078	30.23078	30.17236	Q99K85;Q3U6K9;E9Q6P1	Q99K85;Q3U6K9	Phosphoserine aminotransferase	Psat1
29.89735	29.86231	29.84894	Q9CVB6;D3YXG6;A0A087WRT2	Q9CVB6;D3YXG6	Actin-related protein 2/3 complex subunit 2	Arpc2
29.58978	29.64238	29.66966	Q9Z0N1;A2AAW9;Q9Z0N2	Q9Z0N1;A2AAW9;Q9Z0N2	Eukaryotic translation initiation factor 2 sub	Eif2s3x
29.69975	29.81268	29.64581	Z4YKV1;P63094;Q6R0H7;Q66L47;Q8CG	Z4YKV1;P63094;Q6R0H7	Guanine nucleotide-binding protein G(s) sub	Gnas
22.40406	23.04427	24.37782	E9Q600;Q924C6;E9PY86	E9Q600;Q924C6	Lysyl oxidase homolog 4	Loxl4
30.20769	30.36202	30.21928	O35887;G3UWV3;G3UWR0;G3UY49;G3	O35887;G3UWV3;G3UWR	Calumenin;Calumenin (Fragment)	Calu
29.09144	29.15557	28.99496	P14685;F7B7L8	P14685	26S proteasome non-ATPase regulatory subu	Psmd3
33.14224	33.02003	33.02828	P18760;F8WGL3	P18760;F8WGL3	Cofilin-1	Cfl1
28.29789	28.28472	28.1795	P48722;E0CY23;A0A0N4SW76;A0A0N4S	P48722;E0CY23	Heat shock 70 kDa protein 4L;Heat shock 70	Hspa4l
28.9428	29.06343	29.11396	P54071;D6RIL6;A0A0U1RP68;A0A0U1R	P54071	Isocitrate dehydrogenase [NADP], mitochon	Idh2
29.0531	29.20303	29.26249	P70698	P70698	CTP synthase 1	Ctps1
27.60399	28.14102	28.27142	P97298;B7ZC25;F6S1M4;Q5ND37;E9PW	P97298	Pigment epithelium-derived factor	Serpinf1
27.71947	27.92025	27.97596	Q00493	Q00493	Carboxypeptidase E	Cpe
28.391	28.3452	28.28472	Q60963;E9Q330;E9Q6J0;E9Q4T5	Q60963;E9Q330	Platelet-activating factor acetylhydrolase;Pla	Pla2g7
31.54352	31.64151	31.52496	Q6ZWN5;F7CJS8;D3YWH9;Q9CXW7;D3	Q6ZWN5;F7CJS8;D3YWH9	40S ribosomal protein S9;40S ribosomal prot	Rps9
28.87408	28.77077	28.98142	Q6ZWX6	Q6ZWX6	Eukaryotic translation initiation factor 2 sub	Eif2s1
28.83846	28.87115	28.85638	Q8BLN5;F7BJL0	Q8BLN5	Lanosterol synthase	Lss
27.73892	27.87994	27.85043	Q8CI94;Q9WUB3;E9PUM3;Q9ET01;Q3U	Q8CI94	Glycogen phosphorylase, brain form	Pygb
28.34942	28.47073	28.4073	Q8R1F1;A2ARS6	Q8R1F1	Niban-like protein 1	Fam129b
26.97596	27.10149	27.04009	Q8VDJ3;A0A087WP83;A0A087WS92;A0	Q8VDJ3;A0A087WP83	Vigilin	Hdlbp
30.31959	30.27586	30.34096	Q99JZ4;P36536;A0A1W2P869;A0A1W2	Q99JZ4;P36536	GTP-binding protein SAR1a	Sar1a
30.39305	30.4919	30.40324	Q9CZU6;Q80X68	Q9CZU6	Citrate synthase, mitochondrial	Cs
28.08132	28.20303	28.20769	Q9D0F9;A2CEK3	Q9D0F9;A2CEK3	Phosphoglucomutase-1;Phosphoglucomutas	Pgm1;Pgm2
28.41941	28.56456	28.48616	Q9D2R0	Q9D2R0	Acetoacetyl-CoA synthetase	Aacs
24.16423	23.28207	23.32389	Q9D6Z1;E0CXZ0;F7CHP9;F7CHQ7;A2AP	Q9D6Z1;E0CXZ0	Nucleolar protein 56	Nop56
28.04531	28.06085	28.08132	Q9DBG3;H3BIY9;H3BKM0;H3BJ06;Q5SV	Q9DBG3;H3BIY9;H3BKM0	AP-2 complex subunit beta;AP complex subu	Ap2b1
31.31419	31.39305	31.45807	E9PZ00;Q8BFQ1;K3W4L3;J3QPG5;Q612	E9PZ00;Q8BFQ1;K3W4L3;	Prosaposin	Psap
32.01837	32.0544	31.95394	E9PZF0;Q01768	E9PZF0;Q01768	Nucleoside diphosphate kinase;Nucleoside d	Gm20390;Nme2
29.23763	29.26472	29.34731	E9Q1W0;E9Q1T1;F8WIS9;P11798;F6WH	E9Q1W0;E9Q1T1	Calcium/calmodulin-dependent protein kina	Camk2d
27.90311	28.03486	28.11148	E9Q8Z4;E9Q8Z9;D3Z2H2;E9Q901;E9Q9	E9Q8Z4;E9Q8Z9;D3Z2H2;	Catenin delta-1	Ctnnd1
28.49762	28.77077	28.81115	E9QN70;P02469	E9QN70;P02469	Laminin subunit beta-1	Lamb1
30.28692	30.26472	30.21928	J3QNY1;B2RXM2;A2AL12;Q8BG05;A2AL	J3QNY1;B2RXM2;A2AL12;	Predicted pseudogene 9242;EG627828 prote	Gm9242;Gm679
30.18423	30.19601	30.11148	O08553	O08553	Dihydropyrimidinase-related protein 2	Dpysl2
27.84446	28.01372	28.15557	P06801;Q3TQP6	P06801;Q3TQP6	NADP-dependent malic enzyme;Malic enzym	Me1
29.24672	29.32603	29.34308	P18242;F8WIR1;F6Y6L6;A0A1B0GT66	P18242;F8WIR1;F6Y6L6;A	Cathepsin D;Uncharacterized protein (Fragm	Ctsd
28.3662	28.33245	28.37865	P23116	P23116	Eukaryotic translation initiation factor 3 sub	Eif3a
31.84595	31.95046	31.96774	P47911	P47911	60S ribosomal protein L6	Rpl6
29.11892	29.13369	29.0296	P54775;A0A140LIZ5	P54775;A0A140LIZ5	26S proteasome regulatory subunit 6B	Psmc4
28.20769	28.2535	28.391	P62814;Q91YH6;A0A0U1RNU9	P62814	V-type proton ATPase subunit B, brain isofor	Atp6v1b2
30.98821	30.98142	31.06085	P62918	P62918	60S ribosomal protein L8	Rpl8
27.84446	27.98685	27.87408	Q3TZS3;G3X9Q1;Q61738	Q3TZS3;G3X9Q1;Q61738	Integrin alpha-7	Itga7
23.94056	24.79947	24.03591	Q3UHK6;B7ZNJ5;G3X907;A2ANL9;Q9W	Q3UHK6	Teneurin-4	Tenm4
28.76129	28.06085	28.04531	Q61391;A0A0A6YY36;A0A0A6YWB9;A0	Q61391	Neprilysin	Mme
27.75175	27.87408	27.87994	Q640N1;A0A0R4J0C4;Q9D2L5	Q640N1	Adipocyte enhancer-binding protein 1	Aebp1
27.91456	28.03486	28.07623	Q8C3V4;A0A087WSP5;Q99K94;P42225;	Q8C3V4;A0A087WSP5;Q9	Signal transducer and activator of transcripti	Stat1
27.06085	27.28912	26.80193	Q9JHF5;F6XRE6;F6ZFB8	Q9JHF5	V-type proton ATPase subunit a	Tcirg1
30.48232	30.54812	30.46295	Q9JHZ2	Q9JHZ2	Progressive ankylosis protein	Ankh
28.95947	28.89735	28.98956	A0A1L1STE6;Q9D6R2	A0A1L1STE6;Q9D6R2	Isocitrate dehydrogenase [NAD] subunit, mi	Idh3a
29.25124	29.16758	29.23078	A2AMW0;F7CAZ6	A2AMW0;F7CAZ6	Capping protein (Actin filament) muscle Z-lin	Capzb
27.42342	27.15074	27.19835	E9Q800;Q8CAQ8;E9QAY6;A0A0U1RQ14	E9Q800;Q8CAQ8;E9QAY6	MICOS complex subunit MIC60;MICOS comp	Immt
28.84745	28.74214	28.83545	G3UZI2;A0A0R4J259;G3V018;Q7TMK9;	G3UZI2;A0A0R4J259;G3V0	Heterogeneous nuclear ribonucleoprotein Q	Syncrip
27.67976	27.66628	27.61105	O88325	O88325	Alpha-N-acetylglucosaminidase	Naglu
28.88285	28.77706	29.0531	O88685;A2AGN7;B7ZCF1;F6Q2E3	O88685;A2AGN7;B7ZCF1	26S proteasome regulatory subunit 6A	Psmc3
30.02168	30.07368	30.07368	P10605	P10605	Cathepsin B	Ctsb
31.06728	31.00838	30.98142	P14131	P14131	40S ribosomal protein S16	Rps16
29.3869	29.47267	29.36828	P15864	P15864	Histone H1.2	Hist1h1c
29.74695	29.72273	29.91171	P20060	P20060	Beta-hexosaminidase subunit beta	Hexb
31.94698	31.97801	31.81192	P25444;A0A140T8L5;D3YVC1;D3YWJ3;F	P25444;A0A140T8L5;D3YV	40S ribosomal protein S2;Ribosomal protein	Rps2;Rps2-ps6
29.00035	29.00303	28.99766	P29758	P29758	Ornithine aminotransferase, mitochondrial	Oat
29.3452	29.30008	29.27586	P61161	P61161	Actin-related protein 2	Actr2

29.11892	29.13614	29.12139	P61979;B2M1R6;A0A286YDM3;H3BK96	P61979;B2M1R6;A0A286Y	Heterogeneous nuclear ribonucleoprotein K	Hnrnpk
31.65438	31.70882	31.65438	P62242	P62242	40S ribosomal protein S8	Rps8
30.48232	30.50142	30.57542	P67778;Q5SQG5	P67778;Q5SQG5	Prohibitin;Prohibitin (Fragment)	Phb
31.27031	31.22504	31.16639	Q3TML0	Q3TML0	Protein disulfide-isomerase A6	Pdia6
31.09899	31.20769	31.19013	Q5SW88;Q5SW87;Q5SW86	Q5SW88;Q5SW87	RAB1A, member RAS oncogene family	Rab1a
30.96086	31.04791	30.98821	Q60930;G3UX26;A0A286YCR8;D3YZT5;I	Q60930;G3UX26;A0A286Y	Voltage-dependent anion-selective channel	Vdac2
28.0296	27.94838	27.94838	Q64737;D6RCG1	Q64737	Trifunctional purine biosynthetic protein ade	Gart
28.5969	28.5969	28.45905	Q6PE70;G5E8F8;O70309	Q6PE70;G5E8F8;O70309	Integrin beta;Integrin beta-5	Itgb5
27.63893	27.61807	27.55362	Q6Q899;A2AP29	Q6Q899;A2AP29	Probable ATP-dependent RNA helicase DDX5	Ddx58
29.04009	29.0531	29.0296	Q99MN1;Q8R2P8	Q99MN1;Q8R2P8	Lysine--tRNA ligase	Kars
31.11148	31.08639	31.0927	Q9DBJ1;O70250	Q9DBJ1	Phosphoglycerate mutase 1	Pgam1
30.77706	30.74535	30.75334	Q9Z1Q5	Q9Z1Q5	Chloride intracellular channel protein 1	Clic1
28.65608	28.64238	28.75494	A0A0R4J0G0;Q8BH04;Q9Z2V4	A0A0R4J0G0;Q8BH04	Phosphoenolpyruvate carboxykinase [GTP],	Pck2
30.02168	30.20769	29.95394	A2ACG7;Q9DBG6	A2ACG7;Q9DBG6	Dolichyl-diphosphooligosaccharide--protein	Rpn2
27.69975	27.48616	27.5969	E9PYG6;A0A286YDU0	E9PYG6;A0A286YDU0	RAS p21 protein activator 1;RAS p21 protein	Rasa1
30.14831	30.12386	30.08639	E9Q452;Q8BSH3;E9Q454;P58771;S4R2U	E9Q452;Q8BSH3;E9Q454;	Tropomyosin alpha-1 chain	Tpm1
29.4334	29.4334	29.4073	F8WIV2;Q60854;K7E6F1;E9Q108;E9Q0F	F8WIV2;Q60854;K7E6F1;E	Serine (or cysteine) peptidase inhibitor, clad	Serpinb6a;Serpi
23.70767	23.58692	22.87643	G3XA35;E9PYH0;Q62059;E9QMK3;E9QN	G3XA35;E9PYH0;Q62059;I	MCG116562, isoform CRA_a;Versican core p	Vcan
27.15074	27.13124	27.13124	G5E8D6;Q9WVJ9;E9Q3N9;E9Q3F3;F6Z1	G5E8D6;Q9WVJ9;E9Q3N9	EGF-containing fibulin-like extracellular matr	Efemp2
29.5591	29.54628	29.59335	O09131;D3Z1Q9;Q8K2Q2	O09131	Glutathione S-transferase omega-1	Gsto1
28.07623	28.11148	28.16519	O55222;D3YZA5;A0A1B0GRF6	O55222;D3YZA5	Integrin-linked protein kinase;Integrin-linked	Ilk
31.37244	31.30334	31.30334	P14206;A0A1L1SUK3;A0A1L1SRW0	P14206;A0A1L1SUK3	40S ribosomal protein SA	Rpsa
26.77706	26.86231	26.85043	P24527	P24527	Leukotriene A-4 hydrolase	Lta4h
27.08132	27.0296	27.14102	P26043;Q7TSG6	P26043;Q7TSG6	Radixin	Rdx
29.02697	29.19601	29.13858	P32921	P32921	Tryptophan--tRNA ligase, cytoplasmic	Wars
30.87555	30.91883	30.88285	P35279	P35279	Ras-related protein Rab-6A	Rab6a
29.73892	29.77235	29.72436	P50247;A2ALT5	P50247	Adenosylhomocysteinase	Ahcy
30.78488	30.79266	30.64581	P51150;A0A0N4SVG9;A0A0N4SVR6	P51150	Ras-related protein Rab-7a	Rab7a
27.65267	27.73247	27.61105	P51660	P51660	Peroxisomal multifunctional enzyme type 2	Hsd17b4
30.12386	30.13614	30.00838	P53994;G3UXQ7;G3V022;A0A1D5RMH1	P53994	Ras-related protein Rab-2A	Rab2a
28.90024	28.98414	28.90885	P54822;E9Q242;E9Q3T7;A0A140LHU1;E	P54822;E9Q242;E9Q3T7	Adenylosuccinate lyase	Adsl
30.78488	30.83092	30.72923	P61982	P61982	14-3-3 protein gamma	Ywhag
28.42342	28.45513	28.40324	P62192	P62192	26S proteasome regulatory subunit 4	Psmc1
29.02433	29.2007	29.15316	P62196;Q8K1K2	P62196;Q8K1K2	26S proteasome regulatory subunit 8	Psmc5
31.2135	31.30334	31.25912	P62281;A0A1B0GRR3;A0A1B0GSE8	P62281;A0A1B0GRR3;A0A	40S ribosomal protein S11;40S ribosomal pr	Rps11
30.96774	31.08639	31.04791	P62754	P62754	40S ribosomal protein S6	Rps6
30.94698	30.94	30.95394	P62874;H3BKR2;H3BLF7	P62874;H3BKR2	Guanine nucleotide-binding protein G(I)/G(S	Gnb1
28.88577	28.82335	28.87701	Q3U4W8;P56399;D3YYA5;D3Z4K7	Q3U4W8;P56399	Ubiquitinyl hydrolase 1;Ubiquitin carboxyl-te	Usp5
28.48999	28.58978	28.43141	Q3UZ39;A0A087WSF5;E9Q9T1;A0A087V	Q3UZ39	Leucine-rich repeat flightless-interacting pro	Lrrfip1
26.98685	26.69975	26.9428	Q5SYD0	Q5SYD0	Unconventional myosin-Id	Myo1d
24.72858	25.80193	25.87877	Q61001	Q61001	Laminin subunit alpha-5	Lama5
NaN	21.01563	NaN	Q64277;A0A0R4J190	Q64277;A0A0R4J190	ADP-ribosyl cyclase/cyclic ADP-ribose hydrol	Bst1
28.39509	28.57542	28.37865	Q6P9J9	Q6P9J9	Anoctamin-6	Ano6
27.22619	27.11148	27.50142	Q6PB66	Q6PB66	Leucine-rich PPR motif-containing protein, m	Lrpprc
31.00838	31.02828	31.04791	Q6ZWV3;I7HLV2;P86048;A0A1B0GXC3	Q6ZWV3;I7HLV2;P86048	60S ribosomal protein L10;60S ribosomal pr	Rpl10;Rpl1
27.84446	27.76446	27.80809	Q8BFR4;A0A1W2P8D3;A0A1W2P6W9	Q8BFR4	N-acetylglucosamine-6-sulfatase	Gns
28.42742	28.41538	28.46295	Q8BFR5;A0A0U1RPC4;A0A0U1RNQ6	Q8BFR5	Elongation factor Tu, mitochondrial	Tufm
26.97596	26.95394	26.75175	Q8BH59;V9GXX9	Q8BH59	Calcium-binding mitochondrial carrier prote	Slc25a12
26.75175	27.00838	26.78955	Q8C2E7	Q8C2E7	WASH complex subunit 5	Washc5
27.83846	27.75812	27.78955	Q8JZQ9;A0A0G2JG48	Q8JZQ9	Eukaryotic translation initiation factor 3 sub	Eif3b
28.41941	28.4746	28.48999	Q8R1Q8	Q8R1Q8	Cytoplasmic dynein 1 light intermediate cha	Dync1li1
30.88285	30.82335	30.82335	Q8VEM8;G5E902	Q8VEM8;G5E902	Phosphate carrier protein, mitochondrial;M	Slc25a3
26.51352	26.68646	26.75175	Q91V35;P18052;A0A1B0GRH2;A0A1B0	Q91V35;P18052	Receptor-type tyrosine-protein phosphatase	Ptpra
29.52962	29.6764	29.60576	Q91V41;A2AL34	Q91V41;A2AL34	Ras-related protein Rab-14	Rab14
28.12139	27.75175	27.75812	Q921L6;Q60598	Q921L6;Q60598	Cortactin, isoform CRA_a;Src substrate corta	Cttn
28.74535	28.85341	28.83244	Q99JI4;A0A286YDW8	Q99JI4	26S proteasome non-ATPase regulatory sub	Psmd6
28.05569	28.16039	28.13124	Q9CZW4;E9PUC2;D3Z4I4	Q9CZW4;E9PUC2	Long-chain-fatty-acid--CoA ligase 3	Acsl3
27.06085	27.10149	26.75175	Q9D0E1;B8JK33;B8JK32;F6W322;F7C9U	Q9D0E1;B8JK33;B8JK32	Heterogeneous nuclear ribonucleoprotein M	Hnrnpm
28.90311	28.91456	28.94838	Q9D0K2;Q3UJQ9	Q9D0K2;Q3UJQ9	Succinyl-CoA:3-ketoacid coenzyme A transfe	Oxct1
29.53519	29.54259	29.50142	Q9DBS1	Q9DBS1	Transmembrane protein 43	Tmem43
32.36202	32.49667	32.43092	Q9DCN2;F2Z456;A0A0A0MQM3	Q9DCN2;F2Z456;A0A0A0I	NADH-cytochrome b5 reductase 3;NADH-cyt	Cyb5r3
27.58978	27.07112	27.1795	Q9QUR8	Q9QUR8	Semaphorin-7A	Sema7a
28.4746	28.3869	28.34942	Q9QZE5;Q7TNQ1;Q9QXK3	Q9QZE5	Coatomer subunit gamma-1	Copg1
28.37451	28.49762	28.20303	Q9R1J0	Q9R1J0	Sterol-4-alpha-carboxylate 3-dehydrogenase	Nsdhl
28.39509	28.47846	28.39917	A0A0A6YY47;P13595;A0A0A6YY91;E9Q	A0A0A6YY47;P13595;A0A	Neural cell adhesion molecule 1	Ncam1
26.58978	26.42982	26.93157	A0A0R4J1G9;E9QN92;Q8CI59;D3YTP0	A0A0R4J1G9;E9QN92;Q8C	Metalloreductase STEAP3	Steap3
27.5389	27.71293	27.45513	B1AXN9;P18654;B1AXP0;G3UZI3;E9PW	B1AXN9;P18654	Ribosomal protein S6 kinase alpha-3	Rps6ka3
27.2535	27.43936	27.13124	D3Z158;Q8BML9;A0A140LJH2;A0A0A6Y	D3Z158;Q8BML9	Glutamine--tRNA ligase	Qars
27.49381	27.56819	27.61105	E9Q175;E9Q3L1;E9PVU0;E9Q174;V9GX	E9Q175;E9Q3L1;E9PVU0;E	Unconventional myosin-VI	Myo6
27.58978	27.77077	27.71293	E9Q634;A0A1L1STM1	E9Q634	Unconventional myosin-Ie	Myo1e
25.70767	25.57831	25.85994	E9Q6R7;A0A1W2P7C0;Q61636;A0A1W2	E9Q6R7;A0A1W2P7C0;Q6	Utrophin;G-utrophin (Predicted protein)	Utrn
28.87115	28.80809	28.94838	E9Q9F5;E9Q1G8;O55131	E9Q9F5;E9Q1G8;O55131	Septin-7	Sept7
27.19835	27.04009	27.00838	E9QNX9;Q60751	E9QNX9;Q60751	Tyrosine-protein kinase receptor;Insulin-like	Igf1r
30.96774	31.0544	30.96086	F6YVP7;P62270;S4R1N6;A0A1Y7VKY1	F6YVP7;P62270;S4R1N6;A	Predicted gene 10260;40S ribosomal protein	Gm10260;Rps18
31.24785	31.23649	31.15436	H3BKH6;Q9R0P3;H3BLJ9;H3BJL6;H3BJP	H3BKH6;Q9R0P3;H3BLJ9;H	S-formylglutathione hydrolase	Esd
28.87408	28.82941	28.97596	O08749	O08749	Dihydrolipoyl dehydrogenase, mitochondria	Dld
30.21928	30.20769	30.11148	O08807;B1AZS9	O08807;B1AZS9	Peroxiredoxin-4;Peroxiredoxin-4 (Fragment)	Prdx4
30.06085	30.09899	29.98142	O35129;F6QPR1;F6Q8V7	O35129;F6QPR1	Prohibitin-2;Prohibitin-2 (Fragment)	Phb2
25.99335	25.92705	25.98033	O35375	O35375	Neuropilin-2	Nrp2
29.13369	29.21234	29.10899	O70435;E0CX62;E0CZ34;F8WH02;E0CYL	O70435;E0CX62	Proteasome subunit alpha type-3;Proteasom	Psma3
27.72598	28.14589	27.95947	O88569;A0A0N4SUM2	O88569;A0A0N4SUM2	Heterogeneous nuclear ribonucleoproteins A	Hnrnpa2b1
30.12386	30.21928	30.18423	P06797;A0A1Y7VNM3;A0A1Y7VNQ4;A0	P06797;A0A1Y7VNM3;A0A	Cathepsin L1;Cathepsin L1 (Fragment)	Ctsl
27.98142	28.02433	28.16519	P21279;P30677	P21279	Guanine nucleotide-binding protein G(q) sub	Gnaq
28.41538	28.35363	28.48616	P21550;Q5SX59;J3QPZ9;Q5SX60;Q5SX6	P21550	Beta-enolase	Eno3
28.24898	28.4073	28.4512	P21981;G3UXE8	P21981	Protein-glutamine gamma-glutamyltransfera	Tgm2
27.46295	27.53148	27.63893	P23780;A0A1L1SSJ7	P23780	Beta-galactosidase	Glb1
28.02433	28.12633	27.98685	P24547;A0A0A6YY72;A0A0A6YXS4	P24547;A0A0A6YY72	Inosine-5-monophosphate dehydrogenase 2	Impdh2
28.75494	28.94	28.88285	P30416;F6S2D5;F7CAT1	P30416;F6S2D5;F7CAT1	Peptidyl-prolyl cis-trans isomerase FKBP4;Pe	Fkbp4

30.21928	30.34096	30.19601	P35293	P35293	Ras-related protein Rab-18	Rab18
28.61807	28.68311	28.77077	P45376;D3YVJ7	P45376;D3YVJ7	Aldose reductase;Aldo-keto reductase family	Akr1b1;Akr1b3
28.36202	28.68979	28.47846	P47713;Q9DBX5;A0A087WPN8	P47713;Q9DBX5	Cytosolic phospholipase A2;Phospholipase A	Pla2g4a
30.02168	29.98142	30.02168	P61164	P61164	Alpha-centractin	Actr1a
28.13124	28.43539	28.10149	P61222	P61222	ATP-binding cassette sub-family E member 1	Abce1
31.61105	31.61544	31.69644	P62082;F6SVV1	P62082;F6SVV1	40S ribosomal protein S7	Rps7;Gm9493
30.84595	30.89012	30.82335	P62717;A0A1D5RLW5;A0A1D5RM85;A0	P62717;A0A1D5RLW5;A0A	60S ribosomal protein L18a;60S ribosomal p	Rpl18a
32.44085	32.43589	32.35941	P62806	P62806	Histone H4	Hist1h4a
30.81574	30.86821	30.91171	P62835;A0A0G2JE52;A0A0G2JDL9;A0A0	P62835;A0A0G2JE52;A0A0	Ras-related protein Rap-1A;MCG10748, isofo	Rap1a;Ra
28.88867	29.06856	29.05827	P63005;Q5SW16	P63005	Platelet-activating factor acetylhydrolase IB	Pafah1b1
28.10149	28.13614	28.29351	P63037;B1AXY1;B1AXY0;B1AXX9	P63037;B1AXY1;B1AXY0	DnaJ homolog subfamily A member 1;DnaJ h	Dnaja1
28.29789	28.26696	28.48616	Q5UE59;E9Q7C9;Q7TNF4;Q8CD76;O884	Q5UE59;E9Q7C9;Q7TNF4;	Kinesin light chain 1	Klc1
26.57542	26.43301	26.33756	Q60770;A0A0G2JEI5;A2AEC9;A0A0G2JE	Q60770	Syntaxin-binding protein 3	Stxbp3
29.87408	29.79885	29.85043	Q6GT24;O08709;D3Z0Y2;Q8BG37;A0A0	Q6GT24;O08709;D3Z0Y2	Peroxiredoxin-6	Prdx6
28.00303	28.06599	27.97596	Q8BJY1;F7BA91	Q8BJY1	26S proteasome non-ATPase regulatory sub	Psmd5
29.33671	29.30443	29.42142	Q8C166;B7ZCP4;E9Q806;B7ZCP6;B7ZCP	Q8C166;B7ZCP4;E9Q806;B	Copine-1;Copine-1 (Fragment)	Cpne1
26.49686	26.56092	26.56529	Q8CG19;B1B1E2	Q8CG19;B1B1E2	Latent-transforming growth factor beta-bind	Ltbp1
28.39917	28.33245	28.31527	Q8R0X7;D6REF7;D3YZT4;D3YY13;D3Z1Z	Q8R0X7;D6REF7	Sphingosine-1-phosphate lyase 1	Sgpl1
27.56819	27.5969	27.54628	Q8R1B4;M0QWV3	Q8R1B4	Eukaryotic translation initiation factor 3 sub	Eif3c
28.50142	28.54628	28.56092	Q99LC5	Q99LC5	Electron transfer flavoprotein subunit alpha,	Etfa
27.62506	27.91456	27.78955	Q9CQN1	Q9CQN1	Heat shock protein 75 kDa, mitochondrial	Trap1
29.92592	29.99496	29.92592	Q9CZX8;D3YUT3;D3Z5R8;D3Z722;D3YU	Q9CZX8;D3YUT3;D3Z5R8;I	40S ribosomal protein S19;40S ribosomal pr	Rps19
28.61105	28.69312	28.66628	Q9DB05;P28663	Q9DB05	Alpha-soluble NSF attachment protein	Napa
26.93157	26.73892	26.45199	Q9EQP2	Q9EQP2	EH domain-containing protein 4	Ehd4
28.77706	28.91456	28.79266	Q9WVJ2;E9Q5I9;E9Q0U1;F6ZQQ3;F6PX	Q9WVJ2;E9Q5I9;E9Q0U1	26S proteasome non-ATPase regulatory sub	Psmd13
25.72858	25.68378	25.95394	A0A067XG53;O70589;F6ZQR2;F6Y9I5;B	A0A067XG53;O70589	Peripheral plasma membrane protein CASK	Cask
27.01903	26.97596	26.85043	A0A0J9YUS5;E9PVC6;E9Q9E1;E9PVC5;Q	A0A0J9YUS5;E9PVC6;E9Q	Eukaryotic translation initiation factor 4 gam	Eif4g1
28.32389	28.28912	28.22619	A0A171KXD3;Q9JIF0;A0A140LHF7;A0A1	A0A171KXD3;Q9JIF0;A0A1	Protein arginine N-methyltransferase 1	Prmt1
28.45905	28.20303	28.57542	A2AAN2;Q8BMA6	A2AAN2;Q8BMA6	Signal recognition particle subunit SRP68	Srp68
25.07316	25.34773	24.44884	A2AL50;Q8C0I1;H3BKN2;A2AL49	A2AL50;Q8C0I1;H3BKN2;A	Alkylglycerone-phosphate synthase;Alkyldih	Agps
26.85043	27.14102	27.09144	B7ZCU2;B7ZCU5;B7ZCU4;B7ZCU3;B7ZC	B7ZCU2;B7ZCU5;B7ZCU4;	Abl interactor 1	Abi1
28.09144	28.23535	28.35363	E9PUD2;Q8K1M6	E9PUD2;Q8K1M6	Dynamin-1-like protein	Dnm1l
29.31311	29.258	29.35363	E9PXX7;Q91W90;A0A0R4J1Y7	E9PXX7;Q91W90;A0A0R4J	Thioredoxin domain-containing protein 5	Txndc5
26.75175	27.2803	26.73892	E9Q3T3;L0N7N1;A0A0J9YUN5	E9Q3T3;L0N7N1	Kinesin-like protein KIF14	Kif14
27.67976	27.58262	27.07112	F6SBR5;E9QL53;P49025;D3Z1U0;D3YU8	F6SBR5;E9QL53;P49025;D	Citron Rho-interacting kinase	Cit
27.43936	27.70636	27.64581	G3X8T3;P16675;A2A5J9;A2A5J8;A2A5J6	G3X8T3;P16675	Carboxypeptidase;Lysosomal protective pro	Ctsa
26.55655	26.29439	26.29439	G3XA21;E0CZ22;D3Z624;F6UUF6;E9PYI5	G3XA21;E0CZ22;D3Z624	MCG134445, isoform CRA_a;Maestro heat-li	Mroh1
26.71293	26.83846	26.83846	L7N451;Q80SU7	L7N451;Q80SU7	Interferon-induced very large GTPase 1	Gvin1
23.03758	22.94414	23.55215	O09159	O09159	Lysosomal alpha-mannosidase	Man2b1
27.71293	27.87994	27.81421	O35598;E9PYF2;D3Z1E6	O35598;E9PYF2	Disintegrin and metalloproteinase domain-c	Adam10
30.03486	30.09899	30.02168	O70503	O70503	Very-long-chain 3-oxoacyl-CoA reductase	Hsd17b12
27.73892	27.69975	27.71293	P12023	P12023	Amyloid-beta A4 protein	App
31.06085	31.03486	30.98821	P14869;D3YVM5;S4R1N1	P14869;D3YVM5;S4R1N1	60S acidic ribosomal protein P0;60S acidic ri	Rplp0
29.73731	28.52402	28.26249	P28654	P28654	Decorin	Dcn
26.97596	27.00838	27.05051	P36371	P36371	Antigen peptide transporter 2	Tap2
30.14831	30.24218	30.21928	P46638;G3UY29;E9Q3P9;F8WGS1;P624	P46638;G3UY29;E9Q3P9;F	Ras-related protein Rab-11B;MCG22989, iso	Rab11b;Rab11a
30.66289	30.61982	30.62854	P47963	P47963	60S ribosomal protein L13	Rpl13
28.07623	28.20303	28.24445	P51655	P51655	Glypican-4	Gpc4
28.01903	28.05569	28.02433	P51863	P51863	V-type proton ATPase subunit d 1	Atp6v0d1
28.87408	28.9428	28.85935	P53986	P53986	Monocarboxylate transporter 1	Slc16a1
29.2535	29.23078	29.21002	P63330	P63330	Serine/threonine-protein phosphatase 2A ca	Ppp2ca
29.72923	29.75812	29.78644	P70296;D3Z1V4;D6RHS6;E9QLE5;Q8VIN	P70296;D3Z1V4;D6RHS6	Phosphatidylethanolamine-binding protein 1	Pebp1
23.33756	NaN	22.27071	P97499	P97499	Telomerase protein component 1	Tep1
27.85638	28.06599	27.86231	Q3TCU5;Q9R233;G3UZZ2	Q3TCU5;Q9R233	Tapasin	Tapbp
28.60045	28.61807	28.65608	Q3ULB1;Q921W7;P47226;D6RH72;A2AI	Q3ULB1;Q921W7;P47226	Testin	Tes
27.79576	27.78955	27.5969	Q5EBP8;P49312	Q5EBP8;P49312	Heterogeneous nuclear ribonucleoprotein A	Hnrnpa1
30.13614	30.04791	30.02168	Q60931;J3QMG3	Q60931;J3QMG3	Voltage-dependent anion-selective channel	Vdac3
31.0544	31.0414	30.98821	Q60932;F2Z471	Q60932;F2Z471	Voltage-dependent anion-selective channel	Vdac1
24.62087	24.58118	25.33415	Q62000	Q62000	Mimecan	Ogn
26.92025	27.15074	26.78955	Q62448;F7CBP1;G3XA17;F6TW20;F7DA	Q62448;F7CBP1;G3XA17	Eukaryotic translation initiation factor 4 gam	Eif4g2
28.78019	28.85341	28.94838	Q64282	Q64282	Interferon-induced protein with tetratricope	Ifit1
27.15074	27.01903	27.34942	Q64449;A2AAA9	Q64449;A2AAA9	C-type mannose receptor 2	Mrc2
30.17236	29.94	30.03486	Q6ZWZ7;Q9CPR4;B2RY53	Q6ZWZ7;Q9CPR4	60S ribosomal protein L17	Rpl17
32.61325	32.58889	32.36984	Q8BH78	Q8BH78	Reticulon	Rtn4
26.16997	26.47692	26.71293	Q8BTS0;Q61656;S4R1I6;B1ARB9;B1ARC	Q8BTS0;Q61656;S4R1I6	DEAD (Asp-Glu-Ala-Asp) box polypeptide 5;P	Ddx5
27.0296	26.81421	27.31527	Q8BWT1	Q8BWT1	3-ketoacyl-CoA thiolase, mitochondrial	Acaa2
28.07623	28.00303	28.09648	Q8R366;G3UYZ1;A0A0R4J117;A0A0A6Y	Q8R366;G3UYZ1;A0A0R4J	Immunoglobulin superfamily member 8	Igsf8
27.43936	27.29789	27.00838	Q8VI75;Q5U4C5;D6RES6	Q8VI75;Q5U4C5	Importin-4	Ipo4
31.6372	31.48712	31.53427	Q91V55;D3YYM6;D3Z1S8;P97461	Q91V55;D3YYM6;D3Z1S8;	40S ribosomal protein S5;40S ribosomal pro	Rps5;
30.12386	30.09899	30.23078	Q920E5;A0A0G2JEA5;A0A0G2JDJ5;A0A0	Q920E5;A0A0G2JEA5	Farnesyl pyrophosphate synthase;Predicted	Fdps;Gm43738
29.46879	29.44924	29.42342	Q922F4;CON__ENSEMBL:ENSBTAP0000	Q922F4	Tubulin beta-6 chain	Tubb6
28.4073	28.45905	28.41135	Q99KJ8	Q99KJ8	Dynactin subunit 2	Dctn2
29.57903	29.49381	29.39305	Q99LB4;D3YTL5;P24452;D3YZN3;D3YU7	Q99LB4;D3YTL5;P24452	Capping protein (Actin filament), gelsolin-lik	Capg
27.99226	28.04531	27.98685	Q9CQF9;F7CIP8;A0A0N4SWD3;D3Z275	Q9CQF9	Prenylcysteine oxidase	Pcyox1
28.63547	28.67303	28.79576	Q9D819	Q9D819	Inorganic pyrophosphatase	Ppa1
28.64238	28.4512	28.71947	Q9JII6;B1AXW3	Q9JII6;B1AXW3	Alcohol dehydrogenase [NADP(+)];Alcohol d	Akr1a1
29.13124	29.05051	28.98142	Q9QYB1;E0CY42;Q8BXK9;Q8BHB9	Q9QYB1	Chloride intracellular channel protein 4	Clic4
28.20303	28.22159	28.10649	Q9R045;A0A0A6YXQ1;Q8R0Z6;Q640P2	Q9R045	Angiopoietin-related protein 2	Angptl2
29.58441	29.45905	29.42142	Q9R1P0;E9PW69;E9Q0X0	Q9R1P0;E9PW69	Proteasome subunit alpha type-4;Proteason	Psma4
26.49381	26.72598	25.78955	A0A0R4J0H7;Q8K2Z4;A0A087WNQ1;A0	A0A0R4J0H7;Q8K2Z4	Condensin complex subunit 1	Ncapd2
26.93157	27.0296	26.9428	A6PWC3;A2A9Q2;Q8BHG1;Q3V3G9	A6PWC3;A2A9Q2;Q8BHG1	Nardilysin, N-arginine dibasic convertase, NF	Nrd1
28.57903	28.21697	28.34096	E9PWY9;Q8C0C7;D6RIJ2	E9PWY9;Q8C0C7	Phenylalanine--tRNA ligase alpha subunit	Farsa
24.97596	24.98033	24.78955	E9Q718;Q9R0B9	E9Q718;Q9R0B9	Procollagen-lysine,2-oxoglutarate 5-dioxyge	Plod2
30.40324	30.42342	30.33031	F6YY69;F6VW30;P68254	F6YY69;F6VW30;P68254	14-3-3 protein theta (Fragment);14-3-3 prot	Ywhaq
24.55801	23.60964	23.01733	F8WGM5;Q64324;A0A140LJH9;A0A140	F8WGM5;Q64324	Syntaxin-binding protein 2 (Fragment);Synta	Stxbp2
28.61105	28.68979	28.69644	O08795	O08795	Glucosidase 2 subunit beta	Prkcsh
29.12386	29.15798	29.04531	O54734	O54734	Dolichyl-diphosphooligosaccharide--protein	Ddost
22.97071	23.42982	25.10149	P05063	P05063	Fructose-bisphosphate aldolase C	Aldoc

32.85155	32.69851	32.73933	P14069	P14069	Protein S100-A6	S100a6
34.06728	34.02663	33.90634	P16045	P16045	Galectin-1	Lgals1
32.27864	32.2106	32.11768	P17742;A0A1L1SST0;F8VPN3;A0A1L1SR	P17742;A0A1L1SST0	Peptidyl-prolyl cis-trans isomerase A;Peptidy	Ppia
28.48616	28.53519	28.46295	P22437	P22437	Prostaglandin G/H synthase 1	Ptgs1
29.78644	29.82639	29.68479	P24369	P24369	Peptidyl-prolyl cis-trans isomerase B	Ppib
25.46139	25.56964	25.73117	P25206	P25206	DNA replication licensing factor MCM3	Mcm3
28.90885	28.52028	29.15798	P47739;B1ATI6	P47739	Aldehyde dehydrogenase, dimeric NADP-pre	Aldh3a1
27.99226	28.05569	28.06085	P60122;D3YW60	P60122	RuvB-like 1	Ruvbl1
30.04791	30.13614	30.03486	P62827;Q14AA6;Q61820	P62827;Q14AA6	GTP-binding nuclear protein Ran	Ran;1700009N1
30.00838	29.95394	29.81115	P63242;A0A0A0MQM0;J3QPS8;Q8BGY2	P63242;A0A0A0MQM0	Eukaryotic translation initiation factor 5A-1;	Eif5a
31.98821	31.87921	32.16639	P68033;P68134;A0A1D5RM20	P68033;P68134	Actin, alpha cardiac muscle 1;Actin, alpha sk	Actc1;Acta1
23.80931	22.19761	22.94235	P97333	P97333	Neuropilin-1	Nrp1
27.68646	27.85043	27.85043	P97807	P97807	Fumarate hydratase, mitochondrial	Fh
26.57398	26.65949	26.69975	Q06890;E9PUU2;E9PXG5;E9Q8Y5;E9Q9	Q06890;E9PUU2;E9PXG5;	Clusterin;Clusterin (Fragment)	Clu
27.94838	27.82639	27.95394	Q3TDN2;D3Z2H4;D3YUP4;D6RE51;D3Z4	Q3TDN2;D3Z2H4	FAS-associated factor 2;FAS-associated facto	Faf2
25.24264	25.29614	24.56384	Q3UH60;B2RQC7;D3Z5G8	Q3UH60;B2RQC7;D3Z5G8	Disco-interacting protein 2 homolog B;DIP2	Dip2b
28.31527	28.3869	28.15557	Q543K9;P23492;Q9D8C9	Q543K9;P23492;Q9D8C9	Purine nucleoside phosphorylase	Pnp;Pnp2
27.65267	27.89735	28.10149	Q5PRF0	Q5PRF0	HEAT repeat-containing protein 5A	Heatr5a
29.15798	29.04791	29.27364	Q61335	Q61335	B-cell receptor-associated protein 31	Bcap31
27.33245	27.26249	27.08132	Q6PDI5;A2ALV7;A2ALV6	Q6PDI5;A2ALV7	Proteasome-associated protein ECM29 hom	Ecm29;AI31418
27.87994	27.87408	27.95947	Q8BMF4	Q8BMF4	Dihydrolipoyllysine-residue acetyltransferas	Dlat
26.4567	26.33245	26.83846	Q8CG48;B1AWH6	Q8CG48;B1AWH6	Structural maintenance of chromosomes pro	Smc2
27.45513	27.37451	27.24445	Q8QZT1	Q8QZT1	Acetyl-CoA acetyltransferase, mitochondrial	Acat1
28.05051	28.11148	28.10149	Q8QZY1	Q8QZY1	Eukaryotic translation initiation factor 3 sub	Eif3l
28.76446	28.80809	28.5969	Q8R050;Q149F3;F7CE88	Q8R050	Eukaryotic peptide chain release factor GTP-	Gspt1
27.38278	27.57542	27.42342	Q8VCT3;E9PYF1	Q8VCT3;E9PYF1	Aminopeptidase B	Rnpep
28.69312	28.56092	28.80193	Q9CPX4;P29391;A0A1B0GR60;P49945;A	Q9CPX4;P29391;A0A1B0G	Ferritin;Ferritin light chain 1	Ftl1
28.52028	28.53519	28.61105	Q9CPY7;A0A0G2JEM7	Q9CPY7	Cytosol aminopeptidase	Lap3
29.09144	29.04791	28.96774	Q9D8W5;B1AT36;Q3TRH2	Q9D8W5;B1AT36;Q3TRH2	26S proteasome non-ATPase regulatory sub	Psmd12
28.21697	28.45905	28.30661	Q9DB77;A0A140LI98	Q9DB77	Cytochrome b-c1 complex subunit 2, mitoch	Uqcrc2
27.98685	28.01903	27.73247	Q9DCW4;A0A0U1RNP5;A0A0N4SVE0;A0	Q9DCW4;A0A0U1RNP5;A0	Electron transfer flavoprotein subunit beta;E	Etfb
26.83846	26.85043	27.23535	Q9EQH2;A0A1Y7VMT6;A0A1Y7VNY4	Q9EQH2	Endoplasmic reticulum aminopeptidase 1	Erap1
28.28472	28.32389	28.3452	Q9ESU7;P51912	Q9ESU7;P51912	Amino acid transporter;Neutral amino acid t	Slc1a5
30.29789	30.40324	30.23078	Q9QUI0;A0A0A6YXF6;Q9CR99;A0A0A6Y	Q9QUI0;A0A0A6YXF6	Transforming protein RhoA;Transforming pr	Rhoa
26.10149	26.36286	26.67303	Q9R0E1;F6W3Q8	Q9R0E1	Procollagen-lysine,2-oxoglutarate 5-dioxyge	Plod3
27.86231	27.86821	27.80193	Q9R0X4;Q32MW3	Q9R0X4;Q32MW3	Acyl-coenzyme A thioesterase 9, mitochond	Acot9;Acot10
29.2535	29.33884	29.33671	Q9WVA4;A0A0A6YXG6;Q9R1Q8	Q9WVA4;A0A0A6YXG6	Transgelin-2;Transgelin-2 (Fragment)	Tagln2
29.33458	29.37244	29.26024	Q9Z2U0;Q9CWH6	Q9Z2U0	Proteasome subunit alpha type-7	Psma7
27.34096	27.3662	27.31527	Z4YJV4;Q60597;E9Q7L0;Q5SVY1;Q5SVY	Z4YJV4;Q60597	2-oxoglutarate dehydrogenase, mitochondri	Ogdh
25.53445	25.43301	25.49838	A0A087WNM1;Q91Z67;A0A087WSQ1;A	A0A087WNM1;Q91Z67;A0	SLIT-ROBO Rho GTPase-activating protein 2	Srgap2
24.89966	25.98468	25.56964	A0A0R4J1D0;P59108;A0A1D5RLP0	A0A0R4J1D0;P59108	Copine-2	Cpne2
26.06497	26.33926	26.48922	A0A0R4J1Q0;G5E896;Q3UJB9;D6RE33	A0A0R4J1Q0;G5E896;Q3U	Enhancer of mRNA-decapping protein 4;Enh	Edc4
26.13712	26.28207	26.16039	A0A140T8I9;E9Q3L2;D6RJ66;F6XSW4;F6	A0A140T8I9;E9Q3L2	Phosphatidylinositol 4-kinase alpha	Pi4ka
29.58799	29.72436	29.76446	A0A140T8M7;P62751;D3YTY6	A0A140T8M7;P62751	Ribosomal protein L23A, pseudogene 3;60S	Rpl23a-ps3;Rpl2
24.24627	24.1488	22.44	A0A1W2P6L9;Q3UQ28;D3Z5M7	A0A1W2P6L9;Q3UQ28	Peroxidasin homolog	Pxdn
25.34096	25.10949	25.35783	A2AH85;O08810;G3UZ34;G3UXK8	A2AH85;O08810;G3UZ34	116 kDa U5 small nuclear ribonucleoprotein	Eftud2
26.4073	26.73892	26.72598	A2AL85;Q8BSY0;Q8CBM2;A2AL83;A2AL	A2AL85;Q8BSY0;Q8CBM2	Aspartyl/asparaginyl beta-hydroxylase	Asph
26.52402	26.61807	26.52552	A2AW86;Q60767	A2AW86;Q60767	Lymphocyte antigen 75	Ly75
26.75175	26.77706	26.71293	D3Z061;Q8C7R4	D3Z061;Q8C7R4	Ubiquitin-like modifier-activating enzyme 6	Uba6
31.34096	31.47267	31.42842	E9QAZ2;Q9CZM2;B8JKK2	E9QAZ2;Q9CZM2	Ribosomal protein L15;60S ribosomal protei	Gm10020;Rpl15
26.61807	26.61807	26.37451	F8VQJ3;P02468;F6TLW1	F8VQJ3;P02468	Laminin subunit gamma-1	Lamc1
27.26249	27.22619	27.26249	G3X8Y3;Q80UM3;A0A0A6YW80;A0A0A	G3X8Y3;Q80UM3;A0A0A6	N-alpha-acetyltransferase 15, NatA auxiliary	Naa15
25.52253	26.23535	26.21327	G5E866;Q99NB9	G5E866;Q99NB9	Splicing factor 3B subunit 1	Sf3b1
30.34096	30.26472	30.20769	K3W4Q8;P18572;J3QP71	K3W4Q8;P18572;J3QP71	Basigin;Basigin (Fragment)	Bsg
25.83123	26.1035	25.74407	O35350	O35350	Calpain-1 catalytic subunit	Capn1
27.62506	27.66628	27.52402	O88544;D3YV99;F6QTS1;D3Z1R9	O88544;D3YV99;F6QTS1;D	COP9 signalosome complex subunit 4;COP9	Cops4
29.51841	29.1795	28.93719	O89051	O89051	Integral membrane protein 2B	Itm2b
28.73247	28.91171	28.75175	P01899;V9GXI3;G3UX20;G3UZP7;F6VXI	P01899	H-2 class I histocompatibility antigen, D-B al	H2-D1
30.08639	29.96774	30.00838	P08030;A0A1D5RLR6	P08030	Adenine phosphoribosyltransferase	Aprt
29.51465	29.461	29.54628	P08228	P08228	Superoxide dismutase [Cu-Zn]	Sod1
25.90426	26.06085	25.87173	P12265;Q99KJ6;D3YY48	P12265	Beta-glucuronidase	Gusb
27.41538	27.46295	27.07112	P29416	P29416	Beta-hexosaminidase subunit alpha	Hexa
28.87701	28.98414	28.98685	P42208;E9Q3V6;F6WYM0;D3YYB1;D3Z3	P42208;E9Q3V6;F6WYM0	Septin-2;Septin-2 (Fragment)	Sept2
27.97049	28.15557	28.00303	P46978;D3YZN5	P46978	Dolichyl-diphosphooligosaccharide--protein	Stt3a
29.15798	29.24898	29.38072	P47754;D6RCW7;A0A0N4SVM0	P47754;D6RCW7	F-actin-capping protein subunit alpha-2	Capza2
29.91171	29.86231	29.56638	P51410;A0A0G2JES3;A0A140T8T4;D3Z6	P51410;A0A0G2JES3;A0A1	60S ribosomal protein L9;60S ribosomal prot	Rpl9
27.65267	27.86231	27.89735	P57716	P57716	Nicastrin	Ncstn
30.52028	30.48232	30.40324	P60766;G3UZM2;D3Z3L1;F2Z463;D3YX6	P60766	Cell division control protein 42 homolog	Cdc42
30.61105	30.51089	30.35153	P62245;F8WJ41;D3Z712;D3YVB4	P62245;F8WJ41;D3Z712	40S ribosomal protein S15a;40S ribosomal p	Rps15a
30.86821	30.91883	30.94698	P62301;Q921R2;A0A0U1RQ71	P62301;Q921R2	40S ribosomal protein S13	Rps13
30.24218	30.13614	30.09899	P62911;P17932	P62911	60S ribosomal protein L32	Rpl32
29.80347	29.86821	29.67135	P84078;P61205;Q8BSL7;A2A6T9	P84078;P61205;Q8BSL7	ADP-ribosylation factor 1;ADP-ribosylation f	Arf1;Arf3;Arf2
30.04791	30.07368	30.00838	P97370	P97370	Sodium/potassium-transporting ATPase sub	Atp1b3
29.14831	28.98685	28.95116	P97372;G3X9V0;E0CZ90	P97372;G3X9V0	Proteasome activator complex subunit 2;MC	Psme2
26.46607	26.56092	26.33415	Q01279;Q9WVF5;B2KGF7;P70424;Q615	Q01279;Q9WVF5	Epidermal growth factor receptor	Egfr
26.63201	26.69975	27.16039	Q04736;A0A0J9YUV2;F6UND7;A0A087V	Q04736	Tyrosine-protein kinase Yes	Yes1
26.45983	26.32903	27.08132	Q05769	Q05769	Prostaglandin G/H synthase 2	Ptgs2
29.509	29.64753	29.55179	Q3TMX0;O08992;H3BLG5;A2AKJ9;A2AK	Q3TMX0;O08992;H3BLG5	MCG4375, isoform CRA_b;Syntenin-1;Synten	Sdcbp
27.26249	27.82031	27.33245	Q3UKC1;D3Z715;D3Z213;D3Z152	Q3UKC1	Tax1-binding protein 1 homolog	Tax1bp1
25.60116	25.74919	26.1332	Q3UMB9;A0A1W2P6V9	Q3UMB9	WASH complex subunit 4	Washc4
28.80809	28.88867	28.92025	Q5NCU4;A0A1L1SSH9;P07214;Q5NCU3	Q5NCU4;A0A1L1SSH9;P07	SPARC	Sparc
30.73731	30.64581	30.60222	Q5XJF6;P53026;D6RE43	Q5XJF6;P53026	Ribosomal protein;60S ribosomal protein L1	Rpl10a
27.23535	27.30661	27.1795	Q5XJY5	Q5XJY5	Coatomer subunit delta	Arcn1
27.98685	28.01372	28.37036	Q60737;A2ANR6;Q6NSS6	Q60737	Casein kinase II subunit alpha	Csnk2a1
28.33671	28.51653	28.45905	Q61937;Q9DAY9;Q5SQB0;Q5SQB5	Q61937;Q9DAY9;Q5SQB0;	Nucleophosmin	Npm1
28.60752	28.65949	28.50522	Q64674	Q64674	Spermidine synthase	Srm
26.50294	26.37451	26.65949	Q6A0A9	Q6A0A9	Constitutive coactivator of PPAR-gamma-like	FAM120A
26.21141	26.63201	26.61807	Q6PE80;Q00993;F6YPR4	Q6PE80;Q00993	Axl protein;Tyrosine-protein kinase receptor	Axl

27.68646	27.91456	27.65949	Q8BXZ1	Q8BXZ1	Protein disulfide-isomerase TMX3	Tmx3
28.3662	28.49762	28.79266	Q8CAY6;G3XA25;Q80X81;F2Z459	Q8CAY6;G3XA25;Q80X81	Acetyl-CoA acetyltransferase, cytosolic	Acat2
26.80193	26.76446	26.69975	Q8CIN4;Q61036;A3KGC3;A3KGC4;A3KG	Q8CIN4	Serine/threonine-protein kinase PAK 2	Pak2
26.96499	27.08132	27.12139	Q8JZR0;A0A286YD68;A0A286YCF3;A0A2	Q8JZR0	Long-chain-fatty-acid--CoA ligase 5	Acsl5
28.15557	28.09648	28.2535	Q8K0C4	Q8K0C4	Lanosterol 14-alpha demethylase	Cyp51a1
27.33245	27.32389	27.2535	Q91VH2	Q91VH2	Sorting nexin-9	Snx9
27.5389	27.5389	27.51653	Q920A5	Q920A5	Retinoid-inducible serine carboxypeptidase	Scpep1
26.85043	26.30661	26.61807	Q921M7;Q8BHZ0	Q921M7	Protein FAM49B	Fam49b
29.54812	29.58441	29.69312	Q922Q8	Q922Q8	Leucine-rich repeat-containing protein 59	Lrrc59
26.1055	26.35448	25.96939	Q99K70;B1AWT2;B1AWT3;Q7TT45;B1A	Q99K70;B1AWT2;B1AWT3	Ras-related GTP-binding protein C;Ras-relate	Rragc;Rragd
30.91883	31.00169	30.90455	Q99PT1	Q99PT1	Rho GDP-dissociation inhibitor 1	Arhgdia
26.4008	26.63201	27.00838	Q9CPU0	Q9CPU0	Lactoylglutathione lyase	Glo1
27.15074	27.1795	27.38278	Q9CR16	Q9CR16	Peptidyl-prolyl cis-trans isomerase D	Ppid
30.52028	30.5389	30.51089	Q9CXW4;E9PYL9;A2BH06	Q9CXW4;E9PYL9;A2BH06	60S ribosomal protein L11;Predicted gene 10	Rpl11;Gm10036
28.65949	28.82639	28.88867	Q9CY58;A0A0N4SV32;Q3UMP4;A0A0N4	Q9CY58;A0A0N4SV32;Q3U	Plasminogen activator inhibitor 1 RNA-bindi	Serbp1
28.58262	28.50142	28.5969	Q9CZ13;A0A0A6YW82;A0A0A6YWX6	Q9CZ13	Cytochrome b-c1 complex subunit 1, mitoch	Uqcrc1
28.52776	28.60399	28.54628	Q9DBC7;A2AI69	Q9DBC7	cAMP-dependent protein kinase type I-alpha	Prkar1a
28.62157	28.59335	28.5969	Q9DC51	Q9DC51	Guanine nucleotide-binding protein G(k) sub	Gnai3
23.66492	24.02749	24.20955	Q9JIK5	Q9JIK5	Nucleolar RNA helicase 2	Ddx21
25.85043	25.74919	26.07725	Q9R0E2;A8Y5E6;F6WNR1	Q9R0E2	Procollagen-lysine,2-oxoglutarate 5-dioxyge	Plod1
27.62506	27.64581	27.98142	Q9WUA2;A0A087WS80;A0A087WPV4;A	Q9WUA2	Phenylalanine--tRNA ligase beta subunit	Farsb
27.82639	27.69975	27.62506	A0A0J9YTY0;A0A0J9YUL3;Q8C1B7;A0A0	A0A0J9YTY0;A0A0J9YUL3;	Septin-11;Septin 11, isoform CRA_b	Sept11
27.69975	27.95394	28.0296	B1AV77;B1ATI0;P47740;A0A140LJF9	B1AV77;B1ATI0;P47740	Aldehyde dehydrogenase;Fatty aldehyde de	Aldh3a2
NaN	NaN	NaN	E9QL12;E9PXU9;A0A0N4SUJ2;E9Q423;A	E9QL12;E9PXU9;A0A0N4S	Dysferlin	Dysf
24.72858	25.03591	24.58118	E9QM38;P55012	E9QM38;P55012	Solute carrier family 12 member 2	Slc12a2
26.46139	26.81421	26.87408	F6RPJ9;Q9JHR7	F6RPJ9;Q9JHR7	Insulin-degrading enzyme (Fragment);Insulin	Ide
25.417	25.1795	25.239	F8VPR2;A2APV2;E9PXE6;F6XR20;A2AQV	F8VPR2;A2APV2;E9PXE6;F	Formin-like protein 2	Fmnl2
29.35363	29.41538	29.31311	F8WJK8;Q99L47;E9Q1V0;E9Q1X9	F8WJK8;Q99L47	Hsc70-interacting protein	St13
26.14297	26.81421	26.81421	H3BJU7;H3BJX8;H3BJ45;H3BJ40;H3BKH	H3BJU7;H3BJX8;H3BJ45;H	Rho guanine nucleotide exchange factor 2	Arhgef2
23.77957	22.9566	22.85567	H3BKN0;Q1HFZ0	H3BKN0;Q1HFZ0	tRNA (cytosine(34)-C(5))-methyltransferase	Nsun2
26.99766	26.93157	27.11148	O08917;G3UYU4;G3UZZ5;G3UWW8;G3	O08917;G3UYU4	Flotillin-1	Flot1
28.27142	28.14589	28.01903	O35343;A0A0B4J1E7;D3YTN1	O35343;A0A0B4J1E7	Importin subunit alpha-3	Kpna4
27.89157	28.0296	28.00303	P11440;D3Z2T9;Q80YP0;P97377;E3W99	P11440;D3Z2T9	Cyclin-dependent kinase 1;Cyclin-dependent	Cdk1
23.77957	23.42982	23.35111	P15626;D3YX76;F6Y363	P15626;D3YX76	Glutathione S-transferase Mu 2;Glutathione	Gstm2
30.04791	30.04791	29.86378	P17047	P17047	Lysosome-associated membrane glycoprote	Lamp2
27.2803	27.38278	27.39917	P17427;A0A0J9YUA7;A0A140LHG0	P17427	AP-2 complex subunit alpha-2	Ap2a2
30.02168	30.12386	30.04791	P19253;A0A1B0GSQ6;A0A1B0GS68;A0A	P19253;A0A1B0GSQ6;A0A	60S ribosomal protein L13a;60S ribosomal p	Rpl13a
26.60399	26.39264	26.69975	P26231;Q65CL1	P26231	Catenin alpha-1	Ctnna1
27.28912	27.45513	27.58978	P28271;Q811J3	P28271	Cytoplasmic aconitate hydratase	Aco1
29.44727	29.63027	29.55545	P35282;A0A1W2P6Z4	P35282	Ras-related protein Rab-21	Rab21
29.50711	28.72273	28.68646	P40936	P40936	Indolethylamine N-methyltransferase	Inmt
30.41337	30.42342	30.42342	P41105;F6Z0X0	P41105	60S ribosomal protein L28	Rpl28
25.37119	24.74407	25.30313	P48759	P48759	Pentraxin-related protein PTX3	Ptx3
NaN	NaN	NaN	P58022	P58022	Lysyl oxidase homolog 2	Loxl2
27.78955	28.01372	27.82639	P58242	P58242	Acid sphingomyelinase-like phosphodiestera	Smpdl3b
29.92592	29.92592	29.96774	P61255;B1ARA3;B1ARA5	P61255;B1ARA3;B1ARA5	60S ribosomal protein L26;60S ribosomal pr	Rpl26
30.42342	30.65438	30.59335	P63325;Q3UW83	P63325;Q3UW83	40S ribosomal protein S10	Rps10
29.4073	29.4649	29.48039	P68510	P68510	14-3-3 protein eta	Ywhah
26.02749	25.98251	26.02115	P70227;P11881	P70227	Inositol 1,4,5-trisphosphate receptor type 3	Itpr3
27.77077	27.67976	27.41538	Q3TPJ8;A2BFF9;O88487;A2BFF8;A2BFF	Q3TPJ8;A2BFF9;O88487;A	Cytoplasmic dynein 1 intermediate chain 2	Dync1i2
26.90885	26.54481	26.95394	Q45VK7;F6QLS2;A0A1L1SRI6;A0A1L1SS	Q45VK7	Cytoplasmic dynein 2 heavy chain 1	Dync2h1
27.11148	27.44727	27.64581	Q61035;G5E823;Q99KK9	Q61035	Histidine--tRNA ligase, cytoplasmic	Hars
27.32389	27.27142	27.2803	Q61792;A2A6G9;A2A6H0;A2A6G6;A2A6	Q61792;A2A6G9;A2A6H0;	LIM and SH3 domain protein 1;LIM and SH3	Lasp1
30.39305	30.4334	30.31959	Q64433	Q64433	10 kDa heat shock protein, mitochondrial	Hspe1
26.77706	26.58978	26.96499	Q64521;A2AQR0	Q64521;A2AQR0	Glycerol-3-phosphate dehydrogenase, mitoc	Gpd2
26.22435	26.97596	25.33074	Q6A099;Q6DFZ1;H3BLN1;H3BIX5;H3BL	Q6A099;Q6DFZ1	Golgi-specific brefeldin A-resistance factor 1	Gbf1
27.87408	27.90885	27.89735	Q6PHN9;S4R1W7	Q6PHN9	Ras-related protein Rab-35	Rab35
30.76129	30.74535	30.87555	Q6ZWQ9;D3YV37;D3Z249	Q6ZWQ9	MCG5400	Myl12a
30.52962	30.45317	30.45317	Q6ZWZ6;F7AEH4;A0A1W2P7A1;P63323	Q6ZWZ6;F7AEH4;A0A1W2	40S ribosomal protein S12	Rps12-ps3;Rps1
27.2535	27.35783	27.29789	Q7TQE2;Q62523;A0A0N4SVD2;A0A0N4	Q7TQE2;Q62523	Zyx protein;Zyxin	Zyx
26.33415	26.39428	26.10149	Q7TT37	Q7TT37	Elongator complex protein 1	Elp1
27.11148	27.04009	27.52402	Q80SW1;F7ATQ6;D3Z2Q0	Q80SW1	S-adenosylhomocysteine hydrolase-like prot	Ahcyl1
27.50142	27.39917	27.43141	Q80W54;I7HIP5	Q80W54	CAAX prenyl protease 1 homolog	Zmpste24
28.22159	27.99766	28.09648	Q80ZP8;Q3TMX5;Q9CXI5;F6USD5;F6T4	Q80ZP8;Q3TMX5;Q9CXI5;	Armet protein;Arginine-rich, mutated in earl	Manf
27.46295	27.48616	27.45513	Q8BFY9;Q3TKD0;J3QMX2	Q8BFY9;Q3TKD0	Transportin-1;Transportin-1 (Fragment)	Tnpo1
28.45513	28.26249	28.61456	Q8BGJ5;Q922I7;Q8CB58;P17225;E9QM	Q8BGJ5;Q922I7;Q8CB58;P	MCG13402, isoform CRA_a;MCG13402, isofo	Ptbp1
29.71129	29.61807	29.63893	Q8BP67;F6RSK3	Q8BP67	60S ribosomal protein L24	Rpl24
27.13124	26.75175	26.80193	Q8BU33;A0A1W2P8E1;A0A1W2P727;A0	Q8BU33;A0A1W2P8E1;A0	Acetolactate synthase-like protein	Ilvbl
28.6764	28.88577	29.0531	Q8C522;A0A1L1SSA0	Q8C522	Endonuclease domain-containing 1 protein	Endod1
25.89735	25.69179	26.61807	Q8C8U0;F7CUU8;A0A0N4SUZ5;G3X957	Q8C8U0	Liprin-beta-1	Ppfibp1
28.32817	28.32817	28.4512	Q8CDN6	Q8CDN6	Thioredoxin-like protein 1	Txnl1
26.64581	26.36953	26.1332	Q8K297	Q8K297	Procollagen galactosyltransferase 1	Colgalt1
26.64581	26.75175	26.35616	Q8K411	Q8K411	Presequence protease, mitochondrial	Pitrm1
26.60399	26.27498	26.16806	Q8VDW0;D6RHT5	Q8VDW0;D6RHT5	ATP-dependent RNA helicase DDX39A;DEAD	Ddx39a;Ddx39
27.67303	28.08132	27.97596	Q91XV3	Q91XV3	Brain acid soluble protein 1	Basp1
28.30226	28.16997	28.23078	Q99N15;A2AFQ2;O08756	Q99N15;A2AFQ2;O08756	17beta-hydroxysteroid dehydrogenase type	Hsd17b10
27.19835	26.89735	27.22619	Q9CXF4	Q9CXF4	TBC1 domain family member 15	Tbc1d15
27.28912	27.33245	27.13124	Q9D1C8	Q9D1C8	Vacuolar protein sorting-associated protein	Vps28
27.13124	27.18896	27.2535	Q9DBH5	Q9DBH5	Vesicular integral-membrane protein VIP36	Lman2
28.11148	28.00303	28.0296	Q9JMH6;A0A1W2P6U1;Q9D8I4;A0A0U1	Q9JMH6	Thioredoxin reductase 1, cytoplasmic	Txnrd1
29.16997	29.26249	29.14589	Q9QUM9;E0CXB1;E0CYT2	Q9QUM9;E0CXB1	Proteasome subunit alpha type-6;Proteason	Psma6
28.95394	28.95394	29.14346	Q9QYJ0	Q9QYJ0	DnaJ homolog subfamily A member 2	Dnaja2
29.94	30.04791	29.95394	Q9R0P5	Q9R0P5	Destrin	Dstn
28.38278	28.31527	28.46295	Q9WV54;D3Z505	Q9WV54	Acid ceramidase	Asah1
29.66119	29.43539	29.53148	Q9WV55	Q9WV55	Vesicle-associated membrane protein-assoc	Vapa
27.07112	27.16997	27.23535	Q9Z1Z2	Q9Z1Z2	Serine-threonine kinase receptor-associated	Strap
28.72598	28.70306	28.76446	A0A0R4IZW8;O88456;A0A0R4J1C2;Q9D	A0A0R4IZW8;O88456;A0A	Calpain small subunit 1	Capns1
26.71293	26.80193	26.80193	A0A1B0GR11;Q93092	A0A1B0GR11;Q93092	Transaldolase	Taldo1

28.62157	28.78955	28.77706	B1AWE0;Q6PFA2;B1AWE1;B1AWD8;B1	B1AWE0;Q6PFA2;B1AWE1	Clathrin light chain	Clta
22.16423	24.44253	25.391	D3Z030;Q6EDY6;F7AI27;E9Q4H6	D3Z030;Q6EDY6;F7AI27	F-actin-uncapping protein LRRC16A	Carmil1
27.35783	27.44727	27.43936	E9PYI8;Q9JMA1	E9PYI8;Q9JMA1	Ubiquitin carboxyl-terminal hydrolase 14	Usp14
27.05051	26.89735	27.0296	E9Q197;Q9CPV4;F6ZTG3;E9Q055;E9Q2	E9Q197;Q9CPV4;F6ZTG3	Glyoxalase domain-containing protein 4	Glod4
26.31872	26.21882	26.4008	E9Q2X6;Q8CG47;A0A0A6YXY3;Q3U763	E9Q2X6;Q8CG47	Structural maintenance of chromosomes pro	Smc4
25.33756	25.56384	25.71555	E9Q638;A0A140LHU0;Q61072;A0A1B0	E9Q638;A0A140LHU0;Q61	Disintegrin and metalloproteinase domain-c	Adam9
28.04531	27.72598	27.46295	E9QKZ2;Q91YE6;E0CXB2;F6TLX3;E0CY4	E9QKZ2;Q91YE6;E0CXB2	Importin-9	Ipo9
29.02168	29.12139	29.04009	E9QN08;Q80T06;P57776;Q91VK2;A0A0	E9QN08;Q80T06;P57776;	Elongation factor 1-delta (Fragment);Elonga	Eef1d
25.7415	25.70767	26.15653	F8VPK5;A0A1Y7VMN0;P70336;P70335;	F8VPK5;A0A1Y7VMN0;P7	Rho-associated protein kinase;Rho-associate	Rock2
26.81421	25.14102	26.90885	F8WJE0;Q60710;F6TVP2;E9Q0K6;E9PY	F8WJE0;Q60710;F6TVP2;	Deoxynucleoside triphosphate triphosphohy	Samhd1
28.81115	28.86526	28.97049	G3UXZ5;P97371;G3UXY0;G3X9K9;G3U	G3UXZ5;P97371;G3UXY0;	Proteasome activator complex subunit 1 (Fr	Psme1
29.32389	29.66628	29.33458	G3X8R0;Q60870	G3X8R0;Q60870	Receptor expression-enhancing protein;Rec	Reep5
28.29351	28.3452	28.47846	G3X9L6;Q9DCX2;B1ASE2	G3X9L6;Q9DCX2;B1ASE2	ATP synthase subunit d, mitochondrial	Gm10250;Atp5
26.56384	26.49686	26.34604	G5E924;Q8R081;G3UY38;G3UY56	G5E924;Q8R081;G3UY38	Heterogeneous nuclear ribonucleoprotein L	Hnrnpl
26.54628	26.63201	26.46295	K3W4T3;Q9Z1G4;A2A599	K3W4T3;Q9Z1G4	V-type proton ATPase subunit a;V-type prot	Atp6v0a1
29.15316	29.12879	29.02168	O35566;A0A1B0GSK8;A0A1B0GRG3;A0	O35566;A0A1B0GSK8	CD151 antigen;Tetraspanin (Fragment)	Cd151
30.06085	29.99496	30.03486	O55142	O55142	60S ribosomal protein L35a	Rpl35a
29.29351	29.2803	29.25575	O70326;O88273	O70326	Gremlin-1	Grem1
26.40406	25.59263	25.84086	P07141;D3Z090;D3YTW1;F6RNW8	P07141;D3Z090	Macrophage colony-stimulating factor 1;Ma	Csf1
28.43936	28.47073	28.44332	P09528	P09528	Ferritin heavy chain	Fth1
28.58262	28.63893	28.52028	P10833;A0A1B0GRG1	P10833	Ras-related protein R-Ras	Rras
29.01638	28.97323	28.99496	P14152;B1ATQ3	P14152	Malate dehydrogenase, cytoplasmic	Mdh1
NaN	24.44253	23.05258	P14733	P14733	Lamin-B1	Lmnb1
26.81421	26.85043	26.85043	P17439;A0A0G2JDK2	P17439;A0A0G2JDK2	Glucosylceramidase	Gba
25.57542	25.58405	26.46451	P18872;D3Z2M7;F7BLT7	P18872;D3Z2M7	Guanine nucleotide-binding protein G(o) su	Gnao1
21.29894	21.94949	21.27071	P24472;A0A1L1SS61	P24472	Glutathione S-transferase A4	Gsta4
29.37244	29.18187	29.35153	P26516	P26516	26S proteasome non-ATPase regulatory sub	Psmd7
27.87994	28.05569	28.12633	P28474;A0A0G2JGS3	P28474	Alcohol dehydrogenase class-3	Adh5
27.11148	27.28912	27.27142	P35486	P35486	Pyruvate dehydrogenase E1 component sub	Pdha1
27.12139	27.18896	27.04009	P40336;A0A1W2P7Z9;A0A1W2P7R7	P40336	Vacuolar protein sorting-associated protein	Vps26a
26.64581	26.52701	26.33586	P47857	P47857	ATP-dependent 6-phosphofructokinase, mu	Pfkm
NaN	NaN	NaN	P47880	P47880	Insulin-like growth factor-binding protein 6	Igfbp6
27.31527	27.34942	27.3662	P50396;B7FAU8;D6RI86	P50396	Rab GDP dissociation inhibitor alpha	Gdi1
30.47267	30.67135	30.82335	P61027;D3YUS4	P61027	Ras-related protein Rab-10	Rab10
27.24445	27.49381	27.43936	P61202;A2AQE4	P61202;A2AQE4	COP9 signalosome complex subunit 2	Cops2
30.61982	30.60222	30.5389	P61358;A2A4Q0	P61358;A2A4Q0	60S ribosomal protein L27;60S ribosomal pr	Rpl27
29.8814	29.81115	29.80193	P62852;A0A1L1SQA8	P62852;A0A1L1SQA8	40S ribosomal protein S25	Rps25
30.07368	29.95394	29.99496	P62880;E9QKR0;D3YZX3;D3Z1M1;D3Z1	P62880;E9QKR0;D3YZX3;	Guanine nucleotide-binding protein G(I)/G(S	Gnb2
30.33031	30.11148	30.20769	P63276	P63276	40S ribosomal protein S17	Rps17
27.11148	27.06085	27.61105	Q02248;E9Q6A9;F7BAC9;F7CRC6;F6QZ	Q02248;E9Q6A9	Catenin beta-1	Ctnnb1
28.56819	28.77077	28.58262	Q11011;F6QYF8;E9Q039;E9Q6F4;F6V7	Q11011;F6QYF8;E9Q039	Puromycin-sensitive aminopeptidase;Amino	Npepps
30.52028	30.52962	30.62854	Q3TF41;Q8BSH9;E9PW66;P28656	Q3TF41;Q8BSH9;E9PW66;	Nucleosome assembly protein 1-like 1	Nap1l1
27.91456	28.11148	28.08639	Q3TVK3;Q9Z2W0;Q8BPW9;A0A087WS3	Q3TVK3;Q9Z2W0;Q8BPW	Aspartyl aminopeptidase	Dnpep
28.40324	28.62854	28.51277	Q3TXV4;Q921E2;D6RFT6	Q3TXV4;Q921E2	Rab22B;Ras-related protein Rab-31	Rab31
26.80193	26.30138	26.10149	Q3UPN1;P29533;A0A0G2JFP9	Q3UPN1;P29533;A0A0G2	Vascular cell adhesion protein 1	Vcam1
23.74919	23.50446	24.99766	Q5FWI3	Q5FWI3	Cell surface hyaluronidase	Tmem2
29.7162	29.7162	29.85935	Q61171;D3Z4A4	Q61171;D3Z4A4	Peroxiredoxin-2;Peroxiredoxin-2 (Fragment)	Prdx2
26.09144	26.07928	26.26428	Q61235;B7ZNU9	Q61235;B7ZNU9	Beta-2-syntrophin	Sntb2
24.417	25.24627	25.239	Q62165	Q62165	Dystroglycan	Dag1
26.58978	26.67303	26.2732	Q62433;E9Q514;E9Q5I8;E9Q3F9;E9PVF	Q62433	Protein NDRG1	Ndrg1
24.98468	25.20955	25.49534	Q6GQT9	Q6GQT9	Nodal modulator 1	Nomo1
28.29351	28.06599	28.13124	Q6P1F6;Q9CWU3;Q925E7;F6RV17;Q6Z	Q6P1F6;Q9CWU3	Serine/threonine-protein phosphatase 2A 5	Ppp2r2a;Ppp2
25.02327	25.59833	25.77455	Q8BK67;A2AWQ2	Q8BK67	Protein RCC2	Rcc2
31.06085	31.00169	31.06085	Q8C253;P16110	Q8C253;P16110	Galectin;Galectin-3	Lgals3
28.27586	28.35783	28.23078	Q8C2Q8;Q91VR2;A2AKU9;A2AKV1;A2A	Q8C2Q8;Q91VR2;A2AKU9	ATP synthase subunit gamma;ATP synthase	Atp5c1
26.40892	26.65949	26.71293	Q8K4Q8	Q8K4Q8	Collectin-12	Colec12
26.61807	26.42662	26.80193	Q91VD9;A0A087WQ77;A0A087WR47;A	Q91VD9	NADH-ubiquinone oxidoreductase 75 kDa su	Ndufs1
26.27675	26.07316	26.16423	Q91VR5;A0A1Y7VM48	Q91VR5	ATP-dependent RNA helicase DDX1	Ddx1
25.98685	26.14492	25.90426	Q921M3	Q921M3	Splicing factor 3B subunit 3	Sf3b3
26.60399	26.67303	26.85043	Q99KK7	Q99KK7	Dipeptidyl peptidase 3	Dpp3
28.55728	28.48232	28.47073	Q99L45;E0CXJ3	Q99L45	Eukaryotic translation initiation factor 2 sub	Eif2s2
27.97049	27.63893	27.53148	Q9CQ60;Q8CBG6;D3Z4X1;F6X8L5	Q9CQ60;Q8CBG6;D3Z4X1	6-phosphogluconolactonase	Pgls
26.22435	26.16231	26.16423	Q9D662;A2ANA0;A2AN97;A2AN98;A2A	Q9D662	Protein transport protein Sec23B	Sec23b
26.3256	25.92931	26.14686	Q9D6X6;A0A140LJH8;A0A140LHF1;A0A	Q9D6X6;A0A140LJH8	Serine protease 23;Serine protease 23 (Frag	Prss23
28.05569	28.09648	27.85638	Q9DCH4	Q9DCH4	Eukaryotic translation initiation factor 3 sub	Eif3f
26.52103	26.68646	26.69975	Q9QWR8	Q9QWR8	Alpha-N-acetylgalactosaminidase	Naga
28.30661	28.05569	27.93157	Q9R0Y5;Z4YN97;A0A0A6YXW8	Q9R0Y5	Adenylate kinase isoenzyme 1	Ak1
25.33074	NaN	24.33074	Q9WUU7	Q9WUU7	Cathepsin Z	Ctsz
26.038	25.97378	26.1035	Q9Z204;A0A1D5RLK6;A0A1D5RMF0;A0	Q9Z204	Heterogeneous nuclear ribonucleoproteins	Hnrnpc
25.58405	25.30313	24.82396	Q9Z2I8;A0A0N4SWD1	Q9Z2I8	Succinate--CoA ligase [GDP-forming] subuni	Suclg2
25.30661	25.60399	25.83123	Z4YL78;A0A0R4J0K2;K3W4R5;A2AGT5	Z4YL78;A0A0R4J0K2;K3W	Cytoskeleton-associated protein 5	Ckap5
26.68646	26.63201	26.72598	A0A0A6YX18;Q8BVE3;A0A0A6YWP6;A0	A0A0A6YX18;Q8BVE3;A0A	V-type proton ATPase subunit H	Atp6v1h
33.9763	33.88831	34.01837	A0A0G2JGD2;P07091	A0A0G2JGD2;P07091	Protein S100-A4 (Fragment);Protein S100-A	S100a4
27.78955	27.79576	27.77706	A0A0R4J083;P51174	A0A0R4J083;P51174	Long-chain-specific acyl-CoA dehydrogenase	Acadl
32.5038	32.35941	32.37244	A0A1W2P6F6;A0A1W2P7Q9;Q60605;Q	A0A1W2P6F6;A0A1W2P7	Myosin light polypeptide 6	Myl6;My
27.0296	27.08132	27.12139	A2A6U3;Q80UG5;A2A6U5;A8Y5D3	A2A6U3;Q80UG5;A2A6U5	Septin-9;Septin-9 (Fragment)	Sept9
24.417	24.48001	24.417	A2AVJ7;Q99PL5	A2AVJ7;Q99PL5	Ribosome-binding protein 1	Rrbp1
24.64306	25.94503	25.73892	A2AWA9;A2AWA7;A2AWB0;A0A0A6YW	A2AWA9	Rab GTPase-activating protein 1	Rabgap1
24.49838	23.63201	23.77957	A6PW84;A2A7Q5;Q3V1T4;F2Z499	A6PW84;A2A7Q5;Q3V1T4	Prolyl 3-hydroxylase 1	P3h1
25.9428	26.15653	25.35448	B1AU25;Q9Z0X1	B1AU25;Q9Z0X1	Apoptosis-inducing factor 1, mitochondrial	Aifm1
25.81909	25.83605	25.99335	B2RRF0;A0A1W2P7Y1;P35822;A0A1W2	B2RRF0;A0A1W2P7Y1;P3	Ptprk protein;Receptor-type tyrosine-prote	Ptprk
26.11942	26.45513	26.2803	D3Z3B8;H7BWY4;E9Q9H0;Q811D0;F6U	D3Z3B8;H7BWY4;E9Q9H0	Disks large homolog 1	Dlg1
27.97596	28.09144	28.01372	D3Z3G6;Q63844;A0A0U1RPX4;A0A0U1	D3Z3G6;Q63844;A0A0U1	Mitogen-activated protein kinase;Mitogen-a	Mapk3
25.90196	25.96279	26.00196	D3Z7P3;D3Z7P4;F7B327;F6U529;F6RD	D3Z7P3;D3Z7P4	Glutaminase kidney isoform, mitochondrial	Gls
25.9428	26.09346	27.33245	E9Q7U2;Q8CGU1	E9Q7U2;Q8CGU1	Calcium-binding and coiled-coil domain-con	Calcoco1
25.47073	26.53297	26.72598	F8VQ29;Q3UQP1;A0A0A6YXJ2	F8VQ29;Q3UQP1	IQ motif-containing GTPase-activating prote	Iqgap3
26.63201	26.73892	26.67303	F8WGG3;Q9JK81;F7A3N3	F8WGG3;Q9JK81;F7A3N3	UPF0160 protein MYG1, mitochondrial (Frag	Myg1
20.47754	20.68859	NaN	F8WH23;E9Q5D9;Q61581	F8WH23;E9Q5D9;Q61581	Insulin-like growth factor-binding protein 7	Igfbp7

27.53148	27.58978	27.63893	J7NUP1;Q60766	J7NUP1;Q60766	Immunity-related GTPase family M protein 1	Irgm1
28.86821	28.66628	28.55362	O09061	O09061	Proteasome subunit beta type-1	Psmb1
27.95394	27.28912	27.87408	O35239;A0A1L1STP0	O35239	Tyrosine-protein phosphatase non-receptor	Ptpn9
27.63201	27.26249	27.2535	O35295	O35295	Transcriptional activator protein Pur-beta	Purb
25.38772	25.57831	25.67034	O35382;Q8C391;Q9CXE1	O35382;Q8C391	Exocyst complex component 4	Exoc4
29.91171	29.86821	29.80809	O35682;A0A0N4SW94	O35682	Myeloid-associated differentiation marker	Myadm
NaN	NaN	NaN	O54724	O54724	Caveolae-associated protein 1	Cavin1
24.23169	23.51653	25.32045	O54782;F6TMZ3;F6Z025	O54782	Epididymis-specific alpha-mannosidase	Man2b2
28.27142	28.88285	28.56819	O55234	O55234	Proteasome subunit beta type-5	Psmb5
28.7357	28.73247	28.58978	O70251;A0A087WS46;G3UX43;G3UZ47	O70251;A0A087WS46	Elongation factor 1-beta;Eukaryotic translati	Eef1b;Eef1b2
26.73892	26.68646	26.53594	O70400;S4R1V0	O70400;S4R1V0	PDZ and LIM domain protein 1;PDZ and LIM	Pdlim1
25.44884	25.43301	25.68913	O88986;E9PWY6	O88986;E9PWY6	2-amino-3-ketobutyrate coenzyme A ligase,	Gcat
27.33245	27.33245	27.2803	O89079;D3Z315;F6YFR7;E9Q6I5;F6XIG5	O89079;D3Z315;F6YFR7	Coatomer subunit epsilon;Coatomer subunit	Cope;Co
28.14589	28.27142	28.24898	P00493	P00493	Hypoxanthine-guanine phosphoribosyltrans	Hprt1
23.09828	NaN	24.50446	P06869	P06869	Urokinase-type plasminogen activator	Plau
28.65949	28.71293	28.54995	P08122;B1AVK5	P08122	Collagen alpha-2(IV) chain	Col4a2
30.94	30.5389	30.39305	P08207	P08207	Protein S100-A10	S100a10
31.32496	31.37762	31.29242	P0DP28;P0DP27;P0DP26;Q3UKW2;Q9D	P0DP28;P0DP27;P0DP26;	Calmodulin-3;Calmodulin-2;Calmodulin-1	Calm3;Calm2;Ca
25.62087	26.01903	25.90196	P11928;Q8K469;Q05BJ7;Q8VI95;Q924S	P11928;Q8K469	2-5-oligoadenylate synthase 1A;2-5 oligoade	Oas1a;Oas1g
28.33671	28.3869	28.31959	P12815	P12815	Programmed cell death protein 6	Pdcd6
25.48922	24.54628	25.2571	P27601;Q9D034	P27601	Guanine nucleotide-binding protein subunit	Gna13
27.42342	27.56819	27.60399	P31230;Q3UZG4;A0A0G2JDW6;A0A0G2	P31230;Q3UZG4;A0A0G2J	Aminoacyl tRNA synthase complex-interacti	Aimp1
26.78955	27.13124	27.05051	P31938	P31938	Dual specificity mitogen-activated protein ki	Map2k1
30.46295	30.52028	30.52962	P35979;F8VQK7	P35979	60S ribosomal protein L12	Rpl12
27.3662	27.51653	27.2803	P43276	P43276	Histone H1.5	Hist1h1b
28.37865	28.62506	28.55728	P45591;A0A1Y7VJ71	P45591	Cofilin-2	Cfl2
27.18896	27.16997	27.30661	P46664	P46664	Adenylosuccinate synthetase isozyme 2	Adss
26.2803	26.44727	26.21882	P47791	P47791	Glutathione reductase, mitochondrial	Gsr
29.73892	29.80655	29.62157	P51881	P51881	ADP/ATP translocase 2	Slc25a5
27.71293	27.73247	27.55362	P54116	P54116	Erythrocyte band 7 integral membrane prote	Stom
28.42742	28.34942	28.54995	P55258;Q9CXS2;Q3TYH2;A0A286YDR2;	P55258	Ras-related protein Rab-8A	Rab8a
29.74535	30.02168	29.79421	P59999;Q3TX55;E9PWA7	P59999	Actin-related protein 2/3 complex subunit 4	Arpc4
28.01903	27.94838	27.88577	P60229	P60229	Eukaryotic translation initiation factor 3 sub	Eif3e
29.15557	29.10649	29.02433	P60335	P60335	Poly(rC)-binding protein 1	Pcbp1
28.18896	28.22159	28.18423	P61089;A0A1W2P7Z3	P61089	Ubiquitin-conjugating enzyme E2 N	Ube2n
29.96774	29.98142	29.94	P62264;D3YVF4;D3Z7I1	P62264;D3YVF4;D3Z7I1	40S ribosomal protein S14;40S ribosomal pr	Rps14
29.96774	29.86378	29.98142	P62830;A2A6F8	P62830	60S ribosomal protein L23	Rpl23
28.24898	28.31527	28.43141	P62960;A2BGG7;A0A0A0MQD2;B2RUF0	P62960;A2BGG7	Nuclease-sensitive element-binding protein	Ybx1
26.86231	27.05051	27.04009	P70452;D6RJ29	P70452	Syntaxin-4	Stx4
26.08335	26.20769	26.01903	P70699;F6R5R5;A2AFL3;F6VEG4;A2AFL5	P70699	Lysosomal alpha-glucosidase	Gaa
28.93438	28.9428	28.89735	P99029;G3UZJ4;H3BJQ7	P99029;G3UZJ4;H3BJQ7	Peroxiredoxin-5, mitochondrial	Prdx5;Prd
26.53445	26.47383	26.31354	Q05D44	Q05D44	Eukaryotic translation initiation factor 5B	Eif5b
NaN	24.62087	24.86703	Q07113	Q07113	Cation-independent mannose-6-phosphate	Igf2r
27.54628	27.34942	27.41538	Q08093;D3Z7R6	Q08093;D3Z7R6	Calponin-2;Calponin	Cnn2
23.58692	24.86703	23.89504	Q3TBA3;P21958;F6QHF1	Q3TBA3;P21958	Antigen peptide transporter 1	Tap1
29.48039	29.56275	29.35783	Q3TLP8;P63001;P60764;A2AC13;Q0514	Q3TLP8;P63001;P60764	RAS-related C3 botulinum substrate 1, isofor	Rac1
27.10149	26.96499	27.07112	Q3TU36;E9Q912;E9Q6Q4;A0A0G2JGC8;	Q3TU36;E9Q912;E9Q6Q4;	RAP1, GTP-GDP dissociation stimulator 1	Rap1gds1
26.99766	26.85043	26.89735	Q3U367;Q9JLJ2	Q3U367;Q9JLJ2	4-trimethylaminobutyraldehyde dehydroger	Aldh9a1
26.2021	26.01052	25.91342	Q3U4F0;Q91V61	Q3U4F0;Q91V61	Sideroflexin-3	Sfxn3
27.52402	27.4073	27.41538	Q3UDE2;F2Z423	Q3UDE2	Tubulin--tyrosine ligase-like protein 12	Ttll12
27.55362	27.51653	27.43141	Q3UDS7;A0A1L1SSF2;Q8VDL4	Q3UDS7;A0A1L1SSF2;Q8V	ADP-dependent glucokinase	Adpgk
28.56092	28.50142	28.39509	Q5RKN9;P47753;A0A0G2JE27	Q5RKN9;P47753	Capping protein (Actin filament) muscle Z-lir	Capza1
27.0296	27.14102	27.07112	Q61166	Q61166	Microtubule-associated protein RP/EB famil	Mapre1
27.34942	27.56092	27.56819	Q61187;A0A1B0GS09;A0A1B0GS10;A0A	Q61187;A0A1B0GS09	Tumor susceptibility gene 101 protein;Tumo	Tsg101
23.79947	NaN	NaN	Q61576;A2A4H9;F6W360	Q61576;A2A4H9	Peptidyl-prolyl cis-trans isomerase FKBP10;P	Fkbp10
24.64306	24.45513	25.05672	Q61881;D3Z6N3;D3Z335;D3Z0J6;D3Z2K	Q61881;D3Z6N3	DNA replication licensing factor MCM7	Mcm7
27.74535	27.75812	27.82639	Q62371;A0A0A6YXY2	Q62371	Discoidin domain-containing receptor 2	Ddr2
25.68646	25.92025	25.96279	Q6NZD2;Q9WV80;D3YWH1	Q6NZD2;Q9WV80	Sorting nexin-1	Snx1
27.84446	27.87994	27.75812	Q6P069	Q6P069	Sorcin	Sri
26.20769	26.14686	26.21882	Q6VH22;A0A0J9YU41;A0A0J9YV04;A0A	Q6VH22;A0A0J9YU41	Intraflagellar transport protein 172 homolog	Ift172
27.19835	27.2803	26.92025	Q80X95;Q6NTA4;G3UYP1	Q80X95;Q6NTA4	Ras-related GTP-binding protein A;Ras-relate	Rraga;Rragb
27.87408	27.90311	28.12139	Q8BFW7	Q8BFW7	Lipoma-preferred partner homolog	Lpp
24.2535	24.11744	23.60964	Q8BGZ6;P51569;A2BDV6	Q8BGZ6;P51569	Alpha-galactosidase A	Gla
29.59513	29.87115	29.42142	Q8BMD8	Q8BMD8	Calcium-binding mitochondrial carrier prote	Slc25a24
27.79576	27.84446	27.97049	Q8BMS1	Q8BMS1	Trifunctional enzyme subunit alpha, mitoch	Hadha
22.83846	24.37782	22.31838	Q8BND5	Q8BND5	Sulfhydryl oxidase 1	Qsox1
NaN	NaN	23.03758	Q8BNY0;A0A0R4J026;P47931;A0A286YI	Q8BNY0;A0A0R4J026;P47	Follistatin	Fst
27.20769	27.22619	27.42342	Q8BP92	Q8BP92	Reticulocalbin-2	Rcn2
27.60399	27.61807	27.42342	Q8BWY3	Q8BWY3	Eukaryotic peptide chain release factor subu	Etf1
27.34942	27.30661	26.35448	Q8VBZ3	Q8VBZ3	Cleft lip and palate transmembrane protein	Clptm1
27.30661	26.63201	27.4073	Q921H8;H3BKL5;H3BJZ9;Q8VCH0;H3BK	Q921H8;H3BKL5;H3BJZ9;C	3-ketoacyl-CoA thiolase A, peroxisomal	Acaa1a
23.11902	23.49228	23.10629	Q99JB8;B1AW91;A6PWR1;B1AW94;B1A	Q99JB8;B1AW91;A6PWR1	Protein kinase C and casein kinase II substra	Pacsin3
26.71293	26.69975	26.76446	Q99KC8	Q99KC8	von Willebrand factor A domain-containing	Vwa5a
27.27142	27.16039	27.391	Q99LP6	Q99LP6	GrpE protein homolog 1, mitochondrial	Grpel1
27.71947	27.63201	27.57542	Q99LS3;A0A0J9YVH9;D3Z4T3;D3Z666	Q99LS3;A0A0J9YVH9;D3Z	Phosphoserine phosphatase;Phosphoserine	Psph;Ps
28.56456	28.61807	28.49762	Q99LX0;A2A813;A2A815;A2A817;A2A8	Q99LX0;A2A813;A2A815	Protein/nucleic acid deglycase DJ-1	Park7
25.33415	25.56092	25.54628	Q99P88	Q99P88	Nuclear pore complex protein Nup155	Nup155
29.94	29.89735	29.73409	Q9CQM8;O09167	Q9CQM8;O09167	60S ribosomal protein L21	Rpl21
28.39509	28.36202	28.30661	Q9CQQ7;A0A0G2JGX3	Q9CQQ7	ATP synthase F(0) complex subunit B1, mito	Atp5f1
26.43778	26.41538	26.509	Q9D071;F7C9N6;E9PW47;F6RGK4;F7A0	Q9D071;F7C9N6	MMS19 nucleotide excision repair protein h	Mms19
26.73892	26.83846	26.89735	Q9D2G2	Q9D2G2	Dihydrolipoyllysine-residue succinyltransfer	Dlst
22.62087	23.77957	22.90976	Q9D8T2	Q9D8T2	Gasdermin-D	Gsdmdc1
29.29132	29.19131	29.26919	Q9DB20;F6XVM5;F7D3P8	Q9DB20	ATP synthase subunit O, mitochondrial	Atp5o
26.63201	26.72598	26.63201	Q9DBV4;A2AD97	Q9DBV4;A2AD97	Matrix remodeling-associated protein 8;Mat	Mxra8
25.36119	25.44253	25.86703	Q9DC23	Q9DC23	DnaJ homolog subfamily C member 10	Dnajc10
26.38278	26.56674	26.3256	Q9EQ06;A8Y5N4;Q8VCR2	Q9EQ06	Estradiol 17-beta-dehydrogenase 11	Hsd17b11
32.18423	31.98142	32.07048	Q9ERK4;E9Q1T9;E9QAX7;F6ZEW4	Q9ERK4;E9Q1T9;E9QAX7;	Exportin-2;Exportin-2 (Fragment)	Cse1l
26.97596	27.06085	27.04009	Q9JIW9;F6QC68	Q9JIW9;F6QC68	Ras-related protein Ral-B;Ras-related protei	Ralb

28.20769	28.28472	28.18423	Q9JJ00	Q9JJ00	Phospholipid scramblase 1	Plscr1
27.92592	27.44727	27.43141	Q9JK48;A0A0G2JEC4;A0A0G2JE45;A0A0	Q9JK48;A0A0G2JEC4	Endophilin-B1	Sh3glb1
27.84446	27.61105	27.61807	Q9QXT0;A0A1W2P729	Q9QXT0;A0A1W2P729	Protein canopy homolog 2;Protein canopy h	Cnpy2
24.09346	23.72858	24.26069	Q9QZQ8;Q8CCK0	Q9QZQ8	Core histone macro-H2A.1	H2afy
28.54628	28.5862	28.4746	Q9R1P3	Q9R1P3	Proteasome subunit beta type-2	Psmb2
29.1795	29.14346	29.16758	Q9Z0J0	Q9Z0J0	Epididymal secretory protein E1	Npc2
28.58262	28.68979	28.54259	Q9Z127	Q9Z127	Large neutral amino acids transporter small	Slc7a5
26.69975	26.27853	26.86231	Z4YJW0;Q8C7X2;A0A1Y7VP81	Z4YJW0;Q8C7X2	ER membrane protein complex subunit 1	Emc1
32.20186	32.0927	32.11148	A0A0N4SV66;Q8R1M2	A0A0N4SV66;Q8R1M2	Histone H2A;Histone H2A.J	H2afj
25.78207	25.86467	25.8044	A0A0R4J107;Q8R146;A0A0A6YXV0	A0A0R4J107;Q8R146	Acylamino-acid-releasing enzyme (Fragment	Apeh
24.84805	24.73892	24.7024	A0A140T8V5;P17918	A0A140T8V5;P17918	Proliferating cell nuclear antigen	Pcna-ps2;Pcna
25.84566	25.85994	26.21327	A0A286YD12;Q91YP2;A0A286YD77	A0A286YD12;Q91YP2	Neurolysin, mitochondrial	Nln
29.92592	29.64581	29.79885	A2A547;P84099	A2A547;P84099	Ribosomal protein L19;60S ribosomal protei	Rpl19
27.63893	27.56092	27.61807	A2AH25;Q5FWK3	A2AH25;Q5FWK3	Rho GTPase-activating protein 1	Arhgap1
25.97596	26.0041	26.01478	B1ASP2;P52332	B1ASP2;P52332	Tyrosine-protein kinase;Tyrosine-protein kin	Jak1
25.58118	25.86467	25.44884	D3Z3E8;E9PW39;D3YVL0;P23249	D3Z3E8;E9PW39;D3YVL0;I	Putative helicase MOV-10	Mov10
26.72598	26.63201	26.60399	E9Q6R3;O08547;A0A0G2JF08;D6RES2	E9Q6R3;O08547	Vesicle-trafficking protein SEC22b	Sec22b
26.37451	26.61807	26.54185	G3UYQ2;A0A1B0GX81;G3UZT6;Q3UF95	G3UYQ2;A0A1B0GX81;G3	Large proline-rich protein BAG6;Large prolin	Bag6
24.26069	NaN	24.62087	O08538	O08538	Angiopoietin-1	Angpt1
24.44884	NaN	25.56964	O08797	O08797	SPI6	Serpinb9
27.90885	27.85638	27.79576	O54962	O54962	Barrier-to-autointegration factor	Banf1
28.23991	28.26249	28.23535	O55022	O55022	Membrane-associated progesterone recepto	Pgrmc1
26.42662	26.44095	26.34435	O70194	O70194	Eukaryotic translation initiation factor 3 sub	Eif3d
26.80193	26.77706	26.80193	O70252;D3YX62;D3YXN4;D3Z4A2	O70252;D3YX62	Heme oxygenase 2;Heme oxygenase 2 (Frag	Hmox2
25.97596	25.70504	25.91342	O70310	O70310	Glycylpeptide N-tetradecanoyltransferase 1	Nmt1
27.4073	27.43936	27.62506	P10404;P11370;B2RQY6	P10404	MLV-related proviral Env polyprotein	
30.21928	30.26472	30.30878	P14115	P14115	60S ribosomal protein L27a	Rpl27a
27.78332	27.80809	27.78332	P21460;A2APX3	P21460;A2APX3	Cystatin-C;Cystatin (Fragment)	Cst3
28.21697	28.30226	27.9428	P24668	P24668	Cation-dependent mannose-6-phosphate re	M6pr
27.51653	28.18896	28.16997	P26645	P26645	Myristoylated alanine-rich C-kinase substrat	Marcks
25.79699	25.97596	25.45513	P27612;F7D1R5	P27612	Phospholipase A-2-activating protein	Plaa
26.51803	26.69975	26.57542	P28063;G3UZW8	P28063;G3UZW8	Proteasome subunit beta type-8;Proteasome	Psmb8
NaN	NaN	23.91342	P28653	P28653	Biglycan	Bgn
29.06599	29.27808	29.15316	P35278;Q8C266	P35278;Q8C266	Ras-related protein Rab-5C	Rab5c
27.37451	27.63201	26.87408	P35951;A0A1L1SRE8	P35951;A0A1L1SRE8	Low-density lipoprotein receptor	Ldlr
31.03486	31.31419	31.14224	P35980;A0A1B0GSS8;A0A1B0GQU8;A0A	P35980;A0A1B0GSS8;A0A	60S ribosomal protein L18	Rpl18;Rpl
25.75939	25.64031	25.19084	P39053;A0A0J9YUE9;A0A0J9YUN4;F6W	P39053;A0A0J9YUE9;A0A0	Dynamin-1	Dnm1
26.34942	26.38607	26.71293	P42125	P42125	Enoyl-CoA delta isomerase 1, mitochondrial	Eci1
28.62506	28.98142	28.4512	P45952;D6RFD7	P45952	Medium-chain specific acyl-CoA dehydrogen	Acadm
26.39917	26.04009	25.85281	P46460;G3UX86	P46460	Vesicle-fusing ATPase	Nsf
27.38278	26.97596	27.49381	P49817;H3BKG0;D3Z148	P49817;H3BKG0;D3Z148	Caveolin-1;Caveolin (Fragment)	Cav1
27.08132	27.15074	27.19835	P52293;A2A600;A2A601;A6PW68;F2Z43	P52293	Importin subunit alpha-1	Kpna2
27.68646	27.43936	27.34096	P54227;D3Z1Z8;D3Z5N2	P54227;D3Z1Z8;D3Z5N2	Stathmin;Stathmin (Fragment)	Stmn1
27.77706	27.69312	27.66628	P54728	P54728	UV excision repair protein RAD23 homolog E	Rad23b
24.51653	24.67034	24.62087	P54754	P54754	Ephrin type-B receptor 3	Ephb3
26.48616	26.40243	26.61807	P55065;A2A5K2;A2A5K3;A2A5K4	P55065;A2A5K2	Phospholipid transfer protein	Pltp
30.27586	30.17236	30.40324	P62889	P62889	60S ribosomal protein L30	Rpl30
29.26919	29.18187	29.24898	P67984	P67984	60S ribosomal protein L22	Rpl22
26.51803	26.69975	26.60399	P68181;H6TMF5;A0A0G2JFT9	P68181	cAMP-dependent protein kinase catalytic su	Prkacb
28.63547	28.64924	28.69312	P70195	P70195	Proteasome subunit beta type-7	Psmb7
25.49838	25.5285	25.76699	P82198;Q3UXJ2	P82198;Q3UXJ2	Transforming growth factor-beta-induced pr	Tgfbi
30.76129	30.76129	30.80809	Q02013	Q02013	Aquaporin-1	Aqp1
26.60399	26.61807	26.75175	Q3TCN2	Q3TCN2	Putative phospholipase B-like 2	Plbd2
26.61807	26.75175	26.65949	Q3UE92;Q6P1B1;S4R1I3;S4R228	Q3UE92;Q6P1B1;S4R1I3	X-prolyl aminopeptidase (Aminopeptidase P	Xpnpep1
27.89157	27.48616	26.83846	Q52KR3;A0A286YDW6	Q52KR3	Protein prune homolog 2	Prune2
26.0041	25.99981	25.92931	Q60668;F6ZV59;G5E8G0;G3X9W0;E9Q5	Q60668;F6ZV59;G5E8G0;(	Heterogeneous nuclear ribonucleoprotein D	Hnrnpd
27.58978	27.13124	27.44727	Q61425	Q61425	Hydroxyacyl-coenzyme A dehydrogenase, m	Hadh
26.97596	26.97596	27.18896	Q62418	Q62418	Drebrin-like protein	Dbnl
29.05051	28.96499	28.97869	Q62426	Q62426	Cystatin-B	Cstb
26.68646	26.77706	26.80193	Q6P542	Q6P542	ATP-binding cassette sub-family F member 1	Abcf1
26.95394	26.86231	26.89735	Q6P5F9;F6YA11	Q6P5F9	Exportin-1	Xpo1
26.24082	26.26249	26.60399	Q6PDL0;A0A1D5RMC1;A0A1D5RM94	Q6PDL0;A0A1D5RMC1;A0	Cytoplasmic dynein 1 light intermediate cha	Dync1li2
26.63201	26.82639	26.77706	Q6PI17;P25785;B1AQJ3	Q6PI17;P25785;B1AQJ3	Metalloproteinase inhibitor 2;Metalloprotei	Timp2
32.16039	32.11148	32.04466	Q6ZWY9;Q64525;Q64478;P10854	Q6ZWY9;Q64525;Q64478;	Histone H2B type 1-C/E/G;Histone H2B type	Hist1h2bc;Hist2
27.76446	27.92592	27.84446	Q78ZM0;O70492;D3Z789;D3Z6Z0	Q78ZM0;O70492;D3Z789;	Sorting nexin 3, isoform CRA_b;Sorting nexir	Snx3
27.2535	27.5389	27.37451	Q7TPC1	Q7TPC1	Corneodesmosin	Cdsn
NaN	NaN	NaN	Q80VQ0;J3QMK6;E9Q3E1;F6QPV9	Q80VQ0	Aldehyde dehydrogenase family 3 member E	Aldh3b1
27.08132	27.23535	27.14102	Q8BGD9	Q8BGD9	Eukaryotic translation initiation factor 4B	Eif4b
26.16423	25.97596	25.87643	Q8BGH2	Q8BGH2	Sorting and assembly machinery component	Samm50
27.80809	27.79576	27.75175	Q8BJU0;A0A1W2P6P1;A0A1W2P7I5	Q8BJU0;A0A1W2P6P1;A0/	Small glutamine-rich tetratricopeptide repea	Sgta
26.51503	26.38278	26.14297	Q8C0Z1;F6SQM4;D3Z6P3	Q8C0Z1	Protein FAM234A	Fam234a
26.82639	26.29789	26.85043	Q8HWB2;A7VMS6;E9Q0G4;G3UXE9;V9(	Q8HWB2	Histocompatibility 2, Q region locus 4	H2-Q4
24.75939	24.48616	25.45826	Q8VI94	Q8VI94	2-5-oligoadenylate synthase-like protein 1	Oasl1
27.31527	27.00838	27.11148	Q91VA7;V9GXV0	Q91VA7	Isocitrate dehydrogenase [NAD] subunit, mit	Idh3b
25.06085	23.65403	25.90656	Q91VC3;A2AFK7;A0A0N4SVP8;E9PV04;/	Q91VC3;A2AFK7;A0A0N4S	Eukaryotic initiation factor 4A-III;Eukaryotic	Eif4a3
27.08132	27.11148	27.1795	Q91WK2	Q91WK2	Eukaryotic translation initiation factor 3 sub	Eif3h
25.30313	25.37119	25.06085	Q924C1	Q924C1	Exportin-5	Xpo5
26.26428	26.391	26.58978	Q99JY0;D3YXU1	Q99JY0;D3YXU1	Trifunctional enzyme subunit beta, mitochor	Hadhb
26.75175	26.83846	26.96499	Q99K48	Q99K48	Non-POU domain-containing octamer-bindir	Nono
26.48616	26.56384	26.93157	Q99KQ4	Q99KQ4	Nicotinamide phosphoribosyltransferase	Nampt
24.21697	24.391	24.30313	Q99PV0;B7ZC27	Q99PV0;B7ZC27	Pre-mRNA-processing-splicing factor 8;Pre-n	Prpf8
27.95394	28.0296	28.00838	Q9CPS5;Q9CX56;Q3TG45	Q9CPS5;Q9CX56;Q3TG45	26S proteasome non-ATPase regulatory sub	Psmd8
28.10649	28.18896	28.18423	Q9CQV8;A2A5N1	Q9CQV8;A2A5N1	14-3-3 protein beta/alpha;14-3-3 protein be	Ywhab
26.51051	26.69975	26.63201	Q9CR62;Q5SX46;Q5SX48	Q9CR62;Q5SX46	Mitochondrial 2-oxoglutarate/malate carrier	Slc25a11
26.99766	27.26249	27.00838	Q9CY27;G3UWE1;Q52L67;Q3TAN8;A0A	Q9CY27;G3UWE1;Q52L67	Very-long-chain enoyl-CoA reductase;MCG1	Tecr
27.85638	27.85043	27.81421	Q9CZ30;B1AYJ9	Q9CZ30	Obg-like ATPase 1	Ola1
27.07112	27.13124	27.19835	Q9CZ44;A2AT02	Q9CZ44;A2AT02	NSFL1 cofactor p47	Nsfl1c
28.30226	28.38278	28.33671	Q9D051	Q9D051	Pyruvate dehydrogenase E1 component sub	Pdhb

27.20769	27.10149	27.21697	Q9D0B6	Q9D0B6	Protein PBDC1	Pbdc1
27.10149	27.34942	27.43936	Q9D1Q6	Q9D1Q6	Endoplasmic reticulum resident protein 44	Erp44
26.80193	26.99766	26.97596	Q9D2Y4;D3YV42	Q9D2Y4	Mixed lineage kinase domain-like protein	Mlkl
26.04427	26.04843	26.40243	Q9D7N9	Q9D7N9	Adipocyte plasma membrane-associated pro	Apmap
27.73247	27.76446	27.79576	Q9D8B3;Q9D7F7	Q9D8B3	Charged multivesicular body protein 4b	Chmp4b
26.04427	26.20583	26.31872	Q9DB73;G3UZG6;G3UXV8;G3UZ57	Q9DB73;G3UZG6	NADH-cytochrome b5 reductase 1;Cytochro	Cyb5r1
24.08537	NaN	23.83846	Q9ESX5;B7ZCL7;F6YUI5;A2AN81;F6S1S5	Q9ESX5;B7ZCL7	H/ACA ribonucleoprotein complex subunit 4	Dkc1
28.45905	28.391	28.58978	Q9JIM1;E9PXM6;E9PWY7;E9Q221;E9Q5	Q9JIM1;E9PXM6	Equilibrative nucleoside transporter 1	Slc29a1
28.81421	28.70636	28.65267	Q9JJH1	Q9JJH1	Ribonuclease 4	Rnase4
26.76446	26.71293	26.63201	Q9JKZ2;V9GXD5	Q9JKZ2	Sodium/myo-inositol cotransporter	Slc5a3
26.4073	26.48769	26.61807	Q9QXB9	Q9QXB9	Developmentally-regulated GTP-binding pro	Drg2
27.61105	27.60399	27.69975	Q9QZD9;A2AE03	Q9QZD9;A2AE03	Eukaryotic translation initiation factor 3 sub	Eif3i
NaN	NaN	25.08132	Q9R002	Q9R002	Interferon-activable protein 202	Ifi202
27.87994	28.07112	27.88577	Q9R1R8;Q9QYF1	Q9R1R8;Q9QYF1	Retinol dehydrogenase 11	Rdh11
24.99766	24.74919	25.57831	Q9WUD1	Q9WUD1	STIP1 homology and U box-containing prote	Stub1
26.12534	26.56238	25.55215	Q9WUP7;A0A087WP81;A0A087WRL3	Q9WUP7;A0A087WP81	Ubiquitin carboxyl-terminal hydrolase isozyn	Uchl5
27.05051	27.24445	27.24445	Q9WVE8	Q9WVE8	Protein kinase C and casein kinase substrate	Pacsin2
26.55069	26.78955	26.73892	Q9WVJ3	Q9WVJ3	Carboxypeptidase Q	Cpq
24.1488	24.22435	23.5285	Q9WVL3	Q9WVL3	Solute carrier family 12 member 7	Slc12a7
25.73117	26.04843	26.04843	Q9Z1T1;Q9JME5	Q9Z1T1	AP-3 complex subunit beta-1	Ap3b1
28.97596	28.98142	29.05051	Q9Z2U1;A0A0G2JF97	Q9Z2U1	Proteasome subunit alpha type-5	Psma5
28.31095	28.21697	28.35363	Q9Z2X1;J3QM80;J3QMT0;J3QP45	Q9Z2X1	Heterogeneous nuclear ribonucleoprotein F	Hnrnpf
24.49228	24.44253	23.55215	A0A0A0MQ76;Q6DFW4;A0A087WQ46;	A0A0A0MQ76;Q6DFW4;A	Nucleolar protein 58;Nucleolar protein 58 (F	Nop58
26.78955	26.97596	26.55801	A0A0G2JGP4;Q9D091;P08556;A0A0G2J	A0A0G2JGP4;Q9D091;P08	GTPase NRas (Fragment);GTPase NRas	Nras
24.74407	25.00196	24.61526	A0A0R4J0D3;Q3TDQ1	A0A0R4J0D3;Q3TDQ1	Dolichyl-diphosphooligosaccharide--protein	Stt3b
26.11347	25.82639	25.65403	A0A2C9F2A2;Q921Q7;A0A286YCR5	A0A2C9F2A2;Q921Q7	Ras and Rab interactor 1	Rin1
24.94503	25.11744	25.29263	A8DIL0;Q9JL15;Q8C6H0	A8DIL0;Q9JL15;Q8C6H0	Galectin;Galectin-8	Lgals8
24.41054	24.40406	24.45513	B7ZWL1;A0A1D5RMJ8;Q6ZQ08;A0A1D5	B7ZWL1;A0A1D5RMJ8;Q6	CCR4-NOT transcription complex subunit 1	Cnot1
27.391	27.26249	27.67303	D3YWF6;Q7TQI3;D3Z7K0	D3YWF6;Q7TQI3;D3Z7K0	Ubiquitin thioesterase OTUB1;Ubiquitin thio	Otub1
NaN	NaN	NaN	D3Z598;E9QPS5;Q8K4G1;E9Q6B8;F6W6	D3Z598;E9QPS5;Q8K4G1;	Latent-transforming growth factor beta-bind	Ltbp4
27.79576	27.82031	27.69312	D3Z645;Q9QZ88;D3YYD5;D3YW98	D3Z645;Q9QZ88;D3YYD5;	Vacuolar protein sorting-associated protein	Vps29
27.10149	26.36119	26.92025	E9PV44;O35143	E9PV44;O35143	ATPase inhibitor, mitochondrial	Atpif1
27.86231	27.88577	28.09648	E9PYH2;Q91V12	E9PYH2;Q91V12	Cytosolic acyl coenzyme A thioester hydrola	Acot7
26.16615	26.2021	25.94503	E9Q1S3;Q01405	E9Q1S3;Q01405	Protein transport protein Sec23A	Sec23a
25.08132	25.22435	25.62366	E9Q405;A0A1C7ZN10;K3W4L0;B2RRE2;	E9Q405;A0A1C7ZN10;K3V	Unconventional myosin-XVIIIa	Myo18a
26.03591	25.87173	26.16423	F8WHM5;Q61543;E9PWD8;F6RSH1	F8WHM5;Q61543	Golgi apparatus protein 1 (Fragment);Golgi a	Glg1
25.1055	24.81909	25.93832	G3UXC7;G3UWL5	G3UXC7;G3UWL5	ADAMTS-like 3	Adamtsl3
27.76446	27.63893	27.38278	G3UXL2;Q9D7G0	G3UXL2;Q9D7G0	Phosphoribosyl pyrophosphate synthetase 1	Prps1l3;Prps1
25.27142	25.27498	25.65676	G3UY19;G3UW86;G3UWQ7;Q99K43;G3	G3UY19;G3UW86;G3UWC	Protein regulator of cytokinesis 1	Prc1
26.55215	26.90885	27.54628	G3UZ26;P50431;G3UYY1;G3UZZ9	G3UZ26;P50431;G3UYY1	Serine hydroxymethyltransferase, cytosolic (	Shmt1
25.85994	25.58692	25.83123	G3XA09;O88879;Q80VR5	G3XA09;O88879	Apoptotic protease-activating factor 1	Apaf1
NaN	24.46139	24.75939	G5E8J0;O35516	G5E8J0;O35516	Neurogenic locus notch homolog protein 2	Notch2
28.82031	28.93719	28.97049	H7BWZ3;Q9JM76;D3Z2F7;D3Z2F8;A0A0	H7BWZ3;Q9JM76;D3Z2F7	Actin-related protein 2/3 complex subunit 3	Arpc3
25.75685	25.81909	25.76192	H7BX95;Q6PDM2;F7AI47;Q9D0B0	H7BX95;Q6PDM2	Serine/arginine-rich-splicing factor 1;Serine/	Srsf1
23.65403	25.03171	25.391	O08599;A0A140LHJ4	O08599	Syntaxin-binding protein 1	Stxbp1
20.81127	21.10789	NaN	O09164	O09164	Extracellular superoxide dismutase [Cu-Zn]	Sod3
28.62854	28.53148	28.48616	O35114	O35114	Lysosome membrane protein 2	Scarb2
27.62506	27.89735	27.66628	O35593	O35593	26S proteasome non-ATPase regulatory sub	Psmd14
26.28736	26.21141	26.4186	O88207	O88207	Collagen alpha-1(V) chain	Col5a1
26.24989	26.81421	26.45826	O88958;D3Z0R5;D3YWR1;Q9CRC9	O88958;D3Z0R5	Glucosamine-6-phosphate isomerase 1;Gluc	Gnpda1
23.68646	23.79947	23.92252	P01027;H3BKW9	P01027	Complement C3	C3
29.25575	29.06856	29.39917	P01887	P01887	Beta-2-microglobulin	B2m
28.88285	28.54628	28.76129	P01901;Q3TH01;G3UXW2;A0A0B4J1G3	P01901;Q3TH01;G3UXW2	H-2 class I histocompatibility antigen, K-B alp	H2-K1
29.05827	29.0296	29.22619	P10639	P10639	Thioredoxin	Txn
28.61105	28.58978	28.54259	P11438	P11438	Lysosome-associated membrane glycoprote	Lamp1
25.68913	25.52253	25.87877	P13439;D6RJ62	P13439	Uridine 5-monophosphate synthase	Umps
29.26472	29.19131	29.29132	P17809;A0A140LIU2;G3UYL0;A2A7P3;G	P17809	Solute carrier family 2, facilitated glucose tra	Slc2a1
26.1776	26.24808	26.35279	P23242;A0A1W2P793;A0A1W2P8A3	P23242	Gap junction alpha-1 protein	Gja1
27.15074	26.95394	27.07112	P28658	P28658	Ataxin-10	Atxn10
NaN	22.39885	NaN	P30681;A0A1B0GQX9	P30681;A0A1B0GQX9	High mobility group protein B2;High mobility	Hmgb2
26.23169	25.7415	26.06085	P31324;H3BK84;A0A0A6YX73;Q8K1M3;	P31324;H3BK84	cAMP-dependent protein kinase type II-beta	Prkar2b
29.34308	29.37036	29.41337	P31786;M0QWU8;Q4VWZ5;D3Z563	P31786;M0QWU8;Q4VWZ	Acyl-CoA-binding protein	Dbi
29.07877	29.09396	29.12386	P34022;H7BX22	P34022;H7BX22	Ran-specific GTPase-activating protein	Ranbp1
26.02538	25.72858	25.6811	P35283;A2CG35	P35283;A2CG35	Ras-related protein Rab-12	Rab12
24.1946	24.82881	NaN	P35505;D3Z2R9	P35505	Fumarylacetoacetase	Fah
25.59833	26.239	25.73635	P35550;A0A140LIR6	P35550	rRNA 2-O-methyltransferase fibrillarin	Fbl
25.38113	25.20955	25.47383	P42227;B7ZC18	P42227;B7ZC18	Signal transducer and activator of transcripti	Stat3
27.51653	27.61105	27.47846	P45377;P21300	P45377	Aldose reductase-related protein 2	Akr1b8
23.51653	NaN	24.47383	P49717	P49717	DNA replication licensing factor MCM4	Mcm4
29.88285	29.83394	29.65096	P49722;Q8BKE0	P49722;Q8BKE0	Proteasome subunit alpha type-2;Proteasom	Psma2
26.12731	26.17379	26.76446	P50518;A0A0N4SW07;A0A0N4SWA3;A0	P50518;A0A0N4SW07	V-type proton ATPase subunit E 1;V-type pro	Atp6v1e1
31.72923	31.55728	31.44825	P50543	P50543	Protein S100-A11	S100a11
24.89966	25.1488	24.7543	P55264;A0A286YCD7;A0A286YCN3	P55264;A0A286YCD7	Adenosine kinase;Adenosine kinase (Fragme	Adk
27.14102	27.05051	27.11148	P57759;F8WJI4;F8WIM7;D6RG87	P57759	Endoplasmic reticulum resident protein 29	Erp29
26.88577	26.64581	26.48616	P61022;B0R091	P61022;B0R091	Calcineurin B homologous protein 1	Chp1
27.41538	27.4073	27.52402	P62071;A0A1B0GRT5	P62071	Ras-related protein R-Ras2	Rras2
30.18423	30.28692	30.23078	P62267	P62267	40S ribosomal protein S23	Rps23
25.97158	25.93382	26.16997	P62748;A0A1Y7VMK3;P84075;A2A7R5;	P62748;A0A1Y7VMK3	Hippocalcin-like protein 1;Hippocalcin-like p	Hpcal1
28.77706	28.97049	28.94559	P62900;A0A0A6YX26;A0A0A6YXL3	P62900;A0A0A6YX26	60S ribosomal protein L31	Rpl31
30.79266	30.96774	30.73731	P62983	P62983	Ubiquitin-40S ribosomal protein S27a	Rps27a
30.41337	30.37244	30.04791	P63028;D3YU75	P63028;D3YU75	Translationally-controlled tumor protein	Tpt1
27.63201	27.69312	27.83244	P63073;A0A0G2JGT5;A0A0G2JH04;A0A0	P63073;A0A0G2JGT5;A0A	Eukaryotic translation initiation factor 4E;Eu	Eif4e
27.53148	27.56092	27.66628	P63085;F6VEI7	P63085	Mitogen-activated protein kinase 1	Mapk1
24.76446	25.86467	25.35783	P70206;Q80UG2	P70206	Plexin-A1	Plxna1
26.18139	26.05466	25.79947	P70290;B7ZCL8;A2AN84;B7ZCL9;B7ZCM	P70290;B7ZCL8;A2AN84;B	55 kDa erythrocyte membrane protein	Mpp1
28.08132	28.06599	28.28912	P70404	P70404	Isocitrate dehydrogenase [NAD] subunit gam	Idh3g
27.41538	27.391	27.30661	P70677;A0A1B0GRX1	P70677	Caspase-3	Casp3
27.30661	27.43141	27.2803	P84096;A0A1B0GSL4	P84096	Rho-related GTP-binding protein RhoG	Rhog

27.38278	27.23535	27.21697	P97315	P97315	Cysteine and glycine-rich protein 1	Csrp1
NaN	NaN	NaN	P97857	P97857	A disintegrin and metalloproteinase with thr	Adamts1
NaN	NaN	NaN	Q05793	Q05793	Basement membrane-specific heparan sulfat	Hspg2
25.53148	26.01903	25.73635	Q3MIA8;G3UXW9;Q99LD4;A0A140LJB7	Q3MIA8;G3UXW9;Q99LD4	COP9 signalosome complex subunit 1	Gps1
NaN	NaN	NaN	Q3ULG5;P97311	Q3ULG5;P97311	DNA helicase;DNA replication licensing facto	Mcm6
NaN	NaN	NaN	Q3ULJ0;D3Z0L6;E0CXN5;P13707	Q3ULJ0	Glycerol-3-phosphate dehydrogenase 1-like	Gpd1l
27.10149	27.12139	27.3662	Q3UVK0;G8JL46;F6TQS9	Q3UVK0	Endoplasmic reticulum metallopeptidase 1	Ermp1
26.72598	25.77706	25.39754	Q4FJZ2;O35345	Q4FJZ2;O35345	Importin subunit alpha;Importin subunit alp	Kpna6
25.64856	25.36119	25.81421	Q5SF07;A6X8Z3	Q5SF07;A6X8Z3	Insulin-like growth factor 2 mRNA-binding p	Igf2bp2
29.14346	29.11396	29.1866	Q60692	Q60692	Proteasome subunit beta type-6	Psmb6
26.31354	26.61807	27.11148	Q60865;F6YLI0	Q60865	Caprin-1	Caprin1
27.86231	27.56092	27.63893	Q61081;A0A1L1STC0	Q61081;A0A1L1STC0	Hsp90 co-chaperone Cdc37;Hsp90 co-chape	Cdc37
25.07316	25.24627	25.32732	Q61655;Q8R3C7;Q8BZY3;Q9QY15	Q61655;Q8R3C7;Q8BZY3	ATP-dependent RNA helicase DDX19A;DEAD	Ddx19a;Ddx19b
22.99507	23.95837	NaN	Q64442	Q64442	Sorbitol dehydrogenase	Sord
24.6811	25.09748	25.50446	Q6NSR8;F6T2H5	Q6NSR8	Probable aminopeptidase NPEPL1	Npepl1
25.98468	25.84086	25.76699	Q6P2B1;F6TDZ2;A0A0U1RQ41;Q8K368	Q6P2B1	Transportin-3	Tnpo3
26.12731	26.11744	26.12928	Q80XR6;Q20BD0;Q99020	Q80XR6;Q20BD0;Q99020	Heterogeneous nuclear ribonucleoprotein A	Hnrnpab
25.68913	25.64306	25.84566	Q810B6	Q810B6	Rabankyrin-5	Ankfy1
26.89735	26.83846	26.87408	Q8BFY6	Q8BFY6	Peflin	Pef1
26.81421	26.92025	26.64581	Q8BFZ9;A0A1B0GT70;A0A1B0GSD8;A0A	Q8BFZ9;A0A1B0GT70;A0A	Erlin-2;Erlin-2 (Fragment)	Erlin2
26.75175	26.92025	26.71293	Q8BH95	Q8BH95	Enoyl-CoA hydratase, mitochondrial	Echs1
25.1332	25.84805	26.65949	Q8BJW6;D3YZZ6	Q8BJW6	Eukaryotic translation initiation factor 2A	Eif2a
24.33074	24.52253	24.51653	Q8BL66	Q8BL66	Early endosome antigen 1	Eea1
26.61807	27.14102	26.78955	Q8BLF1;Q8BYQ0	Q8BLF1	Neutral cholesterol ester hydrolase 1	Nceh1
26.1332	25.9157	26.18518	Q8BRF7;A0A1W2P6R7	Q8BRF7	Sec1 family domain-containing protein 1	Scfd1
25.21327	25.39754	24.53445	Q8BWB6;D3Z1C4;D3YUM6	Q8BWB6;D3Z1C4;D3YUM	Metalloreductase STEAP2;Metalloreductase	Steap2
24.86231	25.48308	24.6811	Q8BYA0;B1ATU0	Q8BYA0	Tubulin-specific chaperone D	Tbcd
26.31008	26.28383	26.32389	Q8C0E3	Q8C0E3	Tripartite motif-containing protein 47	Trim47
22.43237	24.71293	NaN	Q8CG16;Q8CFG9;Q3UZ09	Q8CG16;Q8CFG9	Complement C1r-A subcomponent;Complem	C1ra;C1rb
26.4567	26.27142	26.78955	Q8JZK9	Q8JZK9	Hydroxymethylglutaryl-CoA synthase, cytopl	Hmgcs1
26.01903	25.49838	25.67034	Q8JZN2;Q91W50;A0A0G2JF72	Q8JZN2;Q91W50;A0A0G2	Cold shock domain-containing protein E1	Csde1
26.08132	26.05466	26.42982	Q8JZZ5;P53811	Q8JZZ5;P53811	Phosphatidylinositol transfer protein beta is	Pitpnb
28.22159	28.53148	28.24898	Q8R010;Q8R3V2	Q8R010;Q8R3V2	Aminoacyl tRNA synthase complex-interacti	Aimp2
26.53594	26.20396	26.31872	Q8R016;E9PY26;E9Q6V3;E9PZH4;E9QA	Q8R016;E9PY26	Bleomycin hydrolase;Bleomycin hydrolase (F	Blmh
27.80193	27.96499	27.92025	Q8VBT0;F6V084	Q8VBT0	Thioredoxin-related transmembrane protein	Tmx1
NaN	21.23608	NaN	Q8VEB1;A0A286YE10;Q9EP84;O70293;A	Q8VEB1	G protein-coupled receptor kinase 5	Grk5
27.01903	26.97596	26.75175	Q91YR1	Q91YR1	Twinfilin-1	Twf1
26.47073	26.52552	27.21697	Q99JX4;A2A702;A2A701	Q99JX4;A2A702	Eukaryotic translation initiation factor 3 sub	Eif3m
22.92796	22.47507	23.32389	Q99KN1;A2AIS9;F7ANZ2;D6RDR8;F6Q4	Q99KN1;A2AIS9;F7ANZ2;	Arrestin domain-containing protein 1;Arrest	Arrdc1
21.8423	20.68859	NaN	Q9CQ65	Q9CQ65	S-methyl-5-thioadenosine phosphorylase	Mtap
27.24445	27.18896	27.39917	Q9CQA3	Q9CQA3	Succinate dehydrogenase [ubiquinone] iron-	Sdhb
29.54628	29.49381	29.53148	Q9CQX2	Q9CQX2	Cytochrome b5 type B	Cyb5b
22.54981	24.15653	NaN	Q9CYD3	Q9CYD3	Cartilage-associated protein	Crtap
27.20769	27.28912	27.16997	Q9CYN2;A0A140LJ01;A0A140LHG8;A0A	Q9CYN2;A0A140LJ01;A0A	Signal peptidase complex subunit 2;Signal pe	Spcs2
25.77203	25.67034	26.50142	Q9CYN9	Q9CYN9	Renin receptor	Atp6ap2
27.05051	26.65949	26.51051	Q9CZ04;D3Z440;D3YVI6;D3Z0S0	Q9CZ04;D3Z440;D3YVI6	COP9 signalosome complex subunit 7a;COP	Cops7a
28.84446	28.81421	28.93438	Q9CZT8;A2A7Z6	Q9CZT8;A2A7Z6	Ras-related protein Rab-3B	Rab3b
27.85043	28.06085	27.88577	Q9D1D4;A0A1Y7VM54	Q9D1D4;A0A1Y7VM54	Transmembrane emp24 domain-containing	Tmed10
29.52962	29.66797	29.61632	Q9D1R9;A0A0G2JEY6;A0A0G2JGY8	Q9D1R9;A0A0G2JEY6;A0A	60S ribosomal protein L34;60S ribosomal pr	Rpl34
23.80931	23.23169	25.19084	Q9DC53;Q9CYR4;Q9DB19	Q9DC53	Copine-8	Cpne8
25.48308	23.96719	24.64306	Q9EPE9	Q9EPE9	Manganese-transporting ATPase 13A1	Atp13a1
27.27142	27.26249	27.30661	Q9JHJ0;A0A1L1SQ12;A0A1L1SR42	Q9JHJ0	Tropomodulin-3	Tmod3
NaN	NaN	NaN	Q9JHU9	Q9JHU9	Inositol-3-phosphate synthase 1	Isyna1
27.45513	27.64581	27.47846	Q9QYA2;G3UY77	Q9QYA2	Mitochondrial import receptor subunit TOM	Tomm40
25.79452	25.97596	25.6513	Q9QYR9	Q9QYR9	Acyl-coenzyme A thioesterase 2, mitochond	Acot2
25.65676	25.59263	25.6971	Q9WTX5;E9PUV4	Q9WTX5	S-phase kinase-associated protein 1	Skp1
25.29263	24.78955	25.34773	Q9Z1Z0;A0A0J9YUG0	Q9Z1Z0	General vesicular transport factor p115	Uso1
25.48001	25.88344	25.95394	Q9Z2D3;D3YYP5;E9Q5V3;E9Q308	Q9Z2D3;D3YYP5;E9Q5V3	Gasdermin-E;Gasdermin E	Gsdme
26.38607	26.75175	26.44727	A0A0G2JEP0;Q61584;A0A0H2UH27;A0A	A0A0G2JEP0;Q61584;A0A	Fragile X mental retardation syndrome-relat	Fxr1
25.65403	25.90426	25.77203	A0A0G2JGQ4;P54729	A0A0G2JGQ4;P54729	NEDD8 ultimate buster 1	Nub1
25.58405	25.92705	26.16997	A0A0R3P9C8;Q9DC69	A0A0R3P9C8;Q9DC69	NADH dehydrogenase [ubiquinone] 1 alpha	Ndufa9
26.42342	26.13907	25.79947	A0A0R4J093;Q9DBP5	A0A0R4J093;Q9DBP5	UMP-CMP kinase	Cmpk1
24.54628	24.59263	24.87643	A0A0R4J0S4;Q80Y17	A0A0R4J0S4;Q80Y17	Lethal(2) giant larvae protein homolog 1	Llgl1
26.40892	26.58978	26.34096	A0A1L1SS44;Q60709;Q06335	A0A1L1SS44;Q60709;Q06	Amyloid-like protein 2 (Fragment);Amyloid-l	Aplp2
NaN	24.37119	24.17188	A0A1L1SVG6;Q8BY89	A0A1L1SVG6;Q8BY89	Choline transporter-like protein 2	Slc44a2
NaN	24.82881	24.61526	A2AFQ0;Q7TMY8;F6UYC1;F6XP90	A2AFQ0;Q7TMY8	E3 ubiquitin-protein ligase HUWE1	Huwe1
26.39591	26.58978	26.52402	A2AMH5;A2AMH3;A2AMH4;Q6X893;A2	A2AMH5;A2AMH3;A2AMH	Choline transporter-like protein 1	Slc44a1
25.96719	26.02538	25.9955	B1AQR8;G3X9T7;O08573	B1AQR8;G3X9T7;O08573	Galectin;Galectin-9	Lgals9
26.67303	26.35448	26.57254	B7ZC46;B1AQY9;B1AQZ0;Q8CHH9;E0CY	B7ZC46;B1AQY9;B1AQZ0;	Septin-8	Sept8;S
26.17188	25.2535	26.75175	B7ZNL2;Q78ZA7;A0A140LJB5;A0A140LJ	B7ZNL2;Q78ZA7	Nap1l4 protein;Nucleosome assembly prote	Nap1l4
25.24627	23.40406	23.59833	B7ZNR9;B2RUE8;A0A0A6YWR8;F8VPL5;	B7ZNR9;B2RUE8;A0A0A6Y	Map4k4 protein;Mitogen-activated protein	Map4k4
25.96719	26.28207	25.99766	B9EHJ3;P39447;A0A0U1RPW2;A0A0U1	B9EHJ3;P39447;A0A0U1R	Tight junction protein ZO-1	Tjp1
25.54038	25.08942	25.42662	D3Z390;Q921C5;A0A1Y7VIQ5	D3Z390;Q921C5	Bicaudal D homolog 2 (Drosophila), isoform	Bicd2
25.54333	25.59833	25.85994	D3Z3F8;Q8R1X6;D3Z2D6;D3Z1M5	D3Z3F8;Q8R1X6	Spastic paraplegia 20, spartin (Troyer syndro	Spg20;Spart
29.708	29.74535	29.42942	D3Z5I1;Q3UPF5;G3X9X5	D3Z5I1;Q3UPF5;G3X9X5	Zinc finger CCCH-type antiviral protein 1	Zc3hav1
26.76446	26.68646	26.65949	E9Q137	E9Q137	Testis-expressed gene 264	Tex264
NaN	22.02242	21.81909	E9Q6C2;Q8CG14;A0A1W2P7F1;Q8CFG8	E9Q6C2;Q8CG14;A0A1W2	Complement component 1, s subcomponent	C1s1;C1sa
26.92025	26.87408	26.98685	E9Q7G1;D3YZZ5	E9Q7G1;D3YZZ5	Transmembrane p24-trafficking protein 7	Tmed7
25.83364	26.12731	26.23535	E9QMK9;Q8BH86	E9QMK9;Q8BH86	D-glutamate cyclase, mitochondrial	Dglucy
26.4346	26.51653	26.45199	F8WI90;P05480;Q0VBH4;Q62270;Q922	F8WI90;P05480	Tyrosine-protein kinase;Neuronal proto-onc	Src
25.45199	25.71293	24.89041	G3X928;Q6NZC7	G3X928;Q6NZC7	SEC23-interacting protein	Sec23ip
24.79452	23.87643	NaN	G5DDB7;Q8R3B1;Q05DG3	G5DDB7;Q8R3B1;Q05DG3	Phosphoinositide phospholipase C;1-phosph	Plcd1
23.71816	23.84805	23.62087	O35286;Q497W9;A0A0G2JG10;A0A0G2	O35286;Q497W9;A0A0G2	Pre-mRNA-splicing factor ATP-dependent RN	Dhx15
25.91114	26.07725	26.06702	O35604	O35604	Niemann-Pick C1 protein	Npc1
25.45513	25.80686	25.47692	O54833;A0A1D5RLE4;A0A1D5RM74;A0	O54833;A0A1D5RLE4;A0A	Casein kinase II subunit alpha;Casein kinase	Csnk2a2
24.7024	24.86703	24.99766	O54988	O54988	STE20-like serine/threonine-protein kinase	Slk
26.17188	25.8044	25.20955	O88587;D3Z227	O88587	Catechol O-methyltransferase	Comt
24.96279	24.93157	25.05672	O88952;Q8JZS0;Q3TUM0;O88951	O88952	Protein lin-7 homolog C	Lin7c

NaN	NaN	NaN	P01897	P01897	H-2 class I histocompatibility antigen, L-D alp	H2-L
26.83846	26.87408	27.05051	P09671	P09671	Superoxide dismutase [Mn], mitochondrial	Sod2
23.20955	NaN	22.31562	P0DOV2;E9QKL6	P0DOV2;E9QKL6	Interferon-activable protein 204	Ifi204
23.78955	23.55215	23.07887	P10810	P10810	Monocyte differentiation antigen CD14	Cd14
25.85043	25.58978	25.66221	P14602;D3YZ06	P14602;D3YZ06	Heat shock protein beta-1	Hspb1
29.06343	29.09144	28.76762	P19157;P46425	P19157	Glutathione S-transferase P 1	Gstp1
27.10149	26.95394	27.11148	P20108	P20108	Thioredoxin-dependent peroxide reductase,	Prdx3
24.74919	25.06497	25.05672	P27046;F6QMB7	P27046	Alpha-mannosidase 2	Man2a1
23.31008	21.75939	23.77957	P29452	P29452	Caspase-1	Casp1
28.0296	28.13124	27.61105	P30412	P30412	Peptidyl-prolyl cis-trans isomerase C	Ppic
25.12534	25.24264	25.68913	P32067;A2AR07;D6RI87;F6SXM5	P32067;A2AR07	Lupus La protein homolog;Lupus La protein	Ssb
25.55215	25.4073	25.35111	P36916	P36916	Guanine nucleotide-binding protein-like 1	Gnl1
28.16039	27.99766	28.0296	P43274	P43274	Histone H1.4	Hist1h1e
26.97596	25.88577	25.93607	P46061	P46061	Ran GTPase-activating protein 1	Rangap1
27.1795	27.42342	27.35783	P47879;H3BLB7;H3BJT2	P47879;H3BLB7	Insulin-like growth factor-binding protein 4	Igfbp4
24.1332	24.17188	24.61526	P50428;F6QGM0	P50428	Arylsulfatase A	Arsa
26.45356	26.48769	26.54185	P54823	P54823	Probable ATP-dependent RNA helicase DDX6	Ddx6
25.59263	25.55801	25.52552	P55302;F6WMD1	P55302	Alpha-2-macroglobulin receptor-associated	Lrpap1
20.40665	NaN	21.51653	P57787;B1ATM1;B1ATM2	P57787;B1ATM1;B1ATM2	Monocarboxylate transporter 4;Monocarbox	Slc16a3
27.56092	27.55362	27.15074	P60521	P60521	Gamma-aminobutyric acid receptor-associat	Gabarapl2
26.95394	27.18896	27.05051	P61226	P61226	Ras-related protein Rap-2b	Rap2b
27.37451	27.27142	27.04009	P62331	P62331	ADP-ribosylation factor 6	Arf6
25.63478	25.96939	25.96058	P62897;CON__P62894;P00015	P62897	Cytochrome c, somatic	Cycs
28.30226	28.26696	28.35783	P63168;Q80ZS7	P63168	Dynein light chain 1, cytoplasmic	Dynll1
31.33565	31.31419	30.96086	P68433	P68433	Histone H3.1	Hist1h3a
24.51051	NaN	23.88577	P70193	P70193	Leucine-rich repeats and immunoglobulin-lik	Lrig1
28.73892	28.57903	28.65267	P70349;B0R1E3	P70349;B0R1E3	Histidine triad nucleotide-binding protein 1	Hint1
24.35111	24.33074	24.06085	P70372	P70372	ELAV-like protein 1	Elavl1
26.1055	26.01691	26.04218	P70460	P70460	Vasodilator-stimulated phosphoprotein	Vasp
28.68979	29.0531	28.84146	P97300;H3BIX4;Z4YLB7;H3BKA7;A0A0A	P97300;H3BIX4;Z4YLB7	Neuroplastin;Neuroplastin (Fragment)	Nptn
31.13614	31.10525	31.06085	P99024;G3UZR1	P99024	Tubulin beta-5 chain	Tubb5
28.00838	28.01372	28.01903	P99026	P99026	Proteasome subunit beta type-4	Psmb4
30.42342	30.51089	30.58441	P99027	P99027	60S acidic ribosomal protein P2	Rplp2
23.79947	23.82881	24.2535	Q00PI9	Q00PI9	Heterogeneous nuclear ribonucleoprotein U	Hnrnpul2
25.33756	25.36119	25.77706	Q08509;Q3UGL1;D3Z5I5;D3Z7F4	Q08509;Q3UGL1	Epidermal growth factor receptor kinase sub	Eps8
27.73247	27.75812	28.06599	Q09143;E9Q3N1;D3Z161	Q09143;E9Q3N1	High affinity cationic amino acid transporter	Slc7a1
26.00838	26.12337	25.90656	Q11136;G3UXC5	Q11136	Xaa-Pro dipeptidase	Pepd
26.14102	26.07928	26.15074	Q3TWV4;P84091	Q3TWV4;P84091	AP-2 complex subunit mu	Ap2m1
24.01903	25.51953	25.30661	Q52KC3;P49718	Q52KC3;P49718	DNA helicase;DNA replication licensing facto	Mcm5
26.38936	26.50142	26.20955	Q5SS83;Q60634	Q5SS83;Q60634	Flotillin 2, isoform CRA_a;Flotillin-2	Flot2
24.22435	NaN	23.81909	Q5SUR0;F6RUL9	Q5SUR0	Phosphoribosylformylglycinamidine synthase	Pfas
25.04009	24.96279	24.83364	Q5SX75;Q60716;B0QZH3;B0QZH1;Q5SX	Q5SX75;Q60716	Procollagen-proline, 2-oxoglutarate 4-dioxyg	P4ha2
NaN	NaN	NaN	Q61468	Q61468	Mesothelin	Msln
26.56529	26.57542	26.52552	Q62087;D6RJ12;H3BL07	Q62087	Serum paraoxonase/lactonase 3	Pon3
25.01052	24.64306	24.47383	Q62318	Q62318	Transcription intermediary factor 1-beta	Trim28
26.81421	28.42342	26.72598	Q63870	Q63870	Collagen alpha-1(VII) chain	Col7a1
26.61807	26.73892	26.69975	Q64191;B7ZNK6	Q64191;B7ZNK6	N(4)-(beta-N-acetylglucosaminyl)-L-asparagi	Aga
25.38772	25.42342	24.83364	Q64669	Q64669	NAD(P)H dehydrogenase [quinone] 1	Nqo1
26.27498	26.31872	26.60399	Q64735;A0A0A6YVT1;A0A0A6YXN9;A0A	Q64735;A0A0A6YVT1;A0A	Complement component receptor 1-like pro	Cr1l
25.44884	25.75939	26.2803	Q6A028	Q6A028	Switch-associated protein 70	Swap70
25.317	24.96279	24.98902	Q6NXL1	Q6NXL1	Sec24-related gene family, member D (S. cer	Sec24d
27.2803	26.67303	26.80193	Q6P5F6;A0A087WRC8;A0A087WQF7	Q6P5F6	Zinc transporter ZIP10	Slc39a10
26.88577	26.37285	26.72598	Q6P8X1;A0A1W2P701	Q6P8X1;A0A1W2P701	Sorting nexin-6	Snx6
26.85043	26.71293	27.15074	Q6ZQI3	Q6ZQI3	Malectin	Mlec
27.12139	26.87408	26.13907	Q76LS9	Q76LS9	Ubiquitin carboxyl-terminal hydrolase MIND	Mindy1
27.01903	26.98685	27.11148	Q791V5;A2AFW6;Q9D050;D6RCZ1	Q791V5;A2AFW6;Q9D050	Mitochondrial carrier homolog 2	Mtch2;
24.05258	22.70345	24.12534	Q80T21	Q80T21	ADAMTS-like protein 4	Adamtsl4
25.02749	24.76446	25.13712	Q80TM9;F7AFP3;F6PY46;F6YR29;F6ZL6	Q80TM9	Nischarin	Nisch
27.79576	27.47073	27.5969	Q8BK64	Q8BK64	Activator of 90 kDa heat shock protein ATPa	Ahsa1
27.11148	26.52253	26.54481	Q8BU14	Q8BU14	Translocation protein SEC62	Sec62
22.97246	22.8423	NaN	Q8BV57	Q8BV57	Soluble scavenger receptor cysteine-rich dor	Ssc5d
25.67303	24.84326	25.63201	Q8BWZ3;G3UZX9;G3UZ51;F6XQZ4	Q8BWZ3	N-alpha-acetyltransferase 25, NatB auxiliary	Naa25
25.72858	26.05466	25.90196	Q8C052;A0A1D5RLY6	Q8C052;A0A1D5RLY6	Microtubule-associated protein 1S	Map1s
25.55215	25.01052	24.49838	Q8C156	Q8C156	Condensin complex subunit 2	Ncaph
26.75175	26.95394	26.72598	Q8C2Q7;O35737	Q8C2Q7;O35737	Heterogeneous nuclear ribonucleoprotein H	Hnrnph1
28.07623	28.27586	28.20303	Q8C4U3	Q8C4U3	Secreted frizzled-related protein 1	Sfrp1
24.6811	24.21697	23.93157	Q8CBB7;P22892	Q8CBB7;P22892	AP-1 complex subunit gamma-1	Ap1g1
26.64581	26.85043	26.73892	Q8CFE6	Q8CFE6	Sodium-coupled neutral amino acid transpo	Slc38a2
25.38772	25.4073	25.53148	Q8CI19	Q8CI19	Platelet-derived growth factor C	Pdgfc
25.83364	25.32732	26.20023	Q8K124	Q8K124	Pleckstrin homology domain-containing fam	Plekho2
26.1946	25.94056	26.03591	Q8K2Q7;A0A0A6YWK4	Q8K2Q7	BRO1 domain-containing protein BROX	Brox
NaN	NaN	NaN	Q8K482	Q8K482	EMILIN-2	Emilin2
29.2957	29.27808	29.12633	Q8R143;E0CXB5;F6TFF2;E0CY65	Q8R143;E0CXB5	Pituitary tumor-transforming gene 1 protein	Pttg1ip
25.12534	25.45513	25.92252	Q8R3X6;Q9R087	Q8R3X6;Q9R087	Glypican-6	Gpc6
26.80193	26.72598	26.4008	Q8VDU3;Q8C7I4	Q8VDU3;Q8C7I4	Apol9a protein;Apolipoprotein L 9b	Apol9a;Apol9b
NaN	NaN	NaN	Q8VI78	Q8VI78	Phospholipase A1 member A	Pla1a
25.69179	25.43301	25.21327	Q8VIJ6	Q8VIJ6	Splicing factor, proline- and glutamine-rich	Sfpq
27.80193	27.58262	27.86821	Q91V89	Q91V89	Serine/threonine-protein phosphatase 2A 56	Ppp2r5d
25.51352	25.45826	25.55801	Q91VR8	Q91VR8	Protein BRICK1	Brk1
26.03171	25.97158	25.99118	Q91XH5;G3UXX3;Q64105;G3UZ79	Q91XH5;G3UXX3;Q64105;	Sepiapterin reductase	Spr
27.15074	27.23535	27.47846	Q921F2;A0A087WRZ5;Q8R0B4;Q8BLD4	Q921F2;A0A087WRZ5;Q8	TAR DNA-binding protein 43;MCG16669, iso	Tardbp
24.12534	NaN	NaN	Q921X9	Q921X9	Protein disulfide-isomerase A5	Pdia5
25.49534	25.1795	25.62087	Q922J9;D3Z5W6;D3Z4N9;D3Z072	Q922J9;D3Z5W6;D3Z4N9	Fatty acyl-CoA reductase 1;Fatty acyl-CoA re	Far1
23.93157	23.98468	23.60964	Q99KP6	Q99KP6	Pre-mRNA-processing factor 19	Prpf19
26.75175	27.06085	26.86231	Q99KV1	Q99KV1	DnaJ homolog subfamily B member 11	Dnajb11
27.58978	27.14102	27.5389	Q99L04	Q99L04	Dehydrogenase/reductase SDR family memb	Dhrs1
25.48308	25.42342	26.17379	Q99LF4	Q99LF4	tRNA-splicing ligase RtcB homolog	Rtcb
25.04843	24.92252	25.2021	Q99ME2	Q99ME2	WD repeat-containing protein 6	Wdr6

28.44332	28.73892	29.10899	Q99P91;Q8BVA0	Q99P91;Q8BVA0	Transmembrane glycoprotein NMB	Gpnmb
26.33245	26.32903	26.57542	Q9CQ62	Q9CQ62	2,4-dienoyl-CoA reductase, mitochondrial	Decr1
28.90311	28.68979	28.75175	Q9CQW2;A0A0N4SVB8	Q9CQW2;A0A0N4SVB8	ADP-ribosylation factor-like protein 8B	Arl8b
26.68646	26.36119	26.2589	Q9CR26;F6W5Q8;F8WJC2	Q9CR26;F6W5Q8	Vacuolar protein sorting-associated protein	Vta1
26.15074	26.11744	26.05672	Q9CWK8	Q9CWK8	Sorting nexin-2	Snx2
26.98685	27.06085	26.9428	Q9CX00	Q9CX00	IST1 homolog	Ist1
25.20583	25.03171	25.43619	Q9CX86	Q9CX86	Heterogeneous nuclear ribonucleoprotein A	Hnrnpa0
26.27498	26.2803	26.36786	Q9CXW3;A0A0A6YY29	Q9CXW3	Calcyclin-binding protein	Cacybp
24.70767	26.14686	26.30487	Q9CY64;A2ASB8;A2ASB7;A2ASB1	Q9CY64	Biliverdin reductase A	Blvra
26.28912	26.17188	26.09346	Q9D0F3	Q9D0F3	Protein ERGIC-53	Lman1
26.65949	26.48769	26.81421	Q9D1M4	Q9D1M4	Eukaryotic translation elongation factor 1 ep	Eef1e1
25.54333	25.44253	25.32045	Q9DBF1;G3UYR8;G3UY72;E9Q1H3;E9Q	Q9DBF1;G3UYR8;G3UY72	Alpha-aminoadipic semialdehyde dehydroge	Aldh7a1
27.35783	27.47073	27.54628	Q9DBG7;F6VF36	Q9DBG7	Signal recognition particle receptor subunit	Srpra
NaN	NaN	NaN	Q9EPK8;D3Z1H7;E9Q7L7;D3Z1H6	Q9EPK8;D3Z1H7;E9Q7L7;	Transient receptor potential cation channel	Trpv4
24.89041	25.10149	25.20955	Q9ET54;D3Z1J5;A0A1W2P7G1;Q5DTJ9	Q9ET54;D3Z1J5	Palladin;Palladin (Fragment)	Palld
27.52402	27.69312	27.5389	Q9JIZ9;Q5F284	Q9JIZ9;Q5F284	Phospholipid scramblase 3;Phospholipid scra	Plscr3
26.30661	26.11545	26.57542	Q9JLV1	Q9JLV1	BAG family molecular chaperone regulator 3	Bag3
24.56384	25.10149	24.85757	Q9QXX4	Q9QXX4	Calcium-binding mitochondrial carrier prote	Slc25a13
25.87643	26.0874	26.08335	Q9QZB7;A0A1Y7VL71;A0A1Y7VM21	Q9QZB7	Actin-related protein 10	Actr10
24.86703	24.90885	24.55215	Q9WTK5	Q9WTK5	Nuclear factor NF-kappa-B p100 subunit	Nfkb2
25.00624	24.98902	24.93607	Q9Z2I0	Q9Z2I0	Mitochondrial proton/calcium exchanger pr	Letm1
26.5285	26.67303	26.92025	Q9Z2I9	Q9Z2I9	Succinate--CoA ligase [ADP-forming] subunit	Sucla2
27.29789	27.27142	27.2535	A0A087WQE6;A0A087WNT1;P83940;A	A0A087WQE6;A0A087WN	Elongin-C (Fragment);Elongin-C	Eloc
25.94056	26.01265	26.09547	A0A0A6YVU8;Q9JKV1;D3YUD8	A0A0A6YVU8;Q9JKV1;D3Y	MCG119397;Proteasomal ubiquitin receptor	Gm9774;Adrm1
28.7357	28.71293	28.90311	A0A0G2JDW7;Q6ZWU9;A0A0G2JG29;A	A0A0G2JDW7;Q6ZWU9;A	40S ribosomal protein S27 (Fragment);40S ri	Rps27
25.98685	26.53742	26.46295	A0A0R4J0R1;O70404;A0A0U1RPE8	A0A0R4J0R1;O70404	Vesicle-associated membrane protein 8	Vamp8
25.76446	25.67034	25.43619	A0A0R4J1Z3;Q9CR67;Q9CZM3	A0A0R4J1Z3;Q9CR67	Transmembrane protein 33	Tmem33
25.97596	26.1055	26.317	A0A1B0GT92;Q9Z1E4;A0A1B0GR90	A0A1B0GT92;Q9Z1E4	Glycogen [starch] synthase;Glycogen [starch	Gys1
24.03591	24.2021	23.57542	A0A1D5RLL3;Q8BG67;Q8BWC5	A0A1D5RLL3;Q8BG67	Protein EFR3 homolog A	Efr3a
26.93157	27.15074	26.76446	A0A286YDJ7;A0A286YDX4;A0A286YCQ1	A0A286YDJ7;A0A286YDX4	Anion exchange protein;Anion exchange pro	Slc4a7
22.07235	21.66275	22.92615	A0A286YDT5;G5E8Q8;A0A286YD74	A0A286YDT5;G5E8Q8	Adhesion G protein-coupled receptor F5 (Fra	Adgrf5
25.49838	25.44253	26.07725	A2AUR7;Q9D031;Q01730	A2AUR7;Q9D031;Q01730	Ras suppressor protein 1	Rsu1
26.21512	26.56674	26.51202	A2BE93;Q9EQU5;A2BE92	A2BE93;Q9EQU5;A2BE92	Protein SET (Fragment);Protein SET	Set
26.99766	27.09144	26.88577	B1AT82;Q9D0M1;B1AT83;B1AT84	B1AT82;Q9D0M1	MCG6846, isoform CRA_c;Phosphoribosyl py	Prpsap1
24.15653	24.28207	25.2021	B1AZ46;Q8BKX1	B1AZ46;Q8BKX1	Brain-specific angiogenesis inhibitor 1-assoc	Baiap2
24.54038	24.46139	24.239	B2RUJ2;B7ZNX6;Q80TH2	B2RUJ2;B7ZNX6;Q80TH2	Erbb2ip protein;Erbin	Erbin
NaN	23.91342	NaN	D3YYI8;O09106;Q8BQ10;A0A0R4J008;P	D3YYI8;O09106;Q8BQ10;	Histone deacetylase;Histone deacetylase 1;H	Gm10093;Hdac
25.32389	25.08942	25.27853	E0CZ72;P28740;F8VQ42	E0CZ72;P28740;F8VQ42	Kinesin-like protein;Kinesin-like protein KIF2	Kif2a
25.29263	25.29263	25.46139	E9PWG6	E9PWG6	Non-SMC condensin I complex, subunit G	Ncapg
25.56674	26.77706	26.44253	E9PXB7;Q8CFI0;A0A286YDR6	E9PXB7;Q8CFI0	E3 ubiquitin-protein ligase NEDD4-like	Nedd4l
26.05466	25.60399	25.56674	E9PZJ8	E9PZJ8	Activating signal cointegrator 1 complex sub	Ascc3
NaN	NaN	NaN	E9Q748;P97430	E9Q748;P97430	Antileukoproteinase	Slpi
27.29789	27.38278	26.29088	F6VQ81;V9GWU5;Q3TUJ9;Q8BKP1;Q3T	F6VQ81;V9GWU5;Q3TUJ9	Tumor protein D54 (Fragment);Tumor prote	Tpd52l2
NaN	22.56151	22.08537	G3UZM9;Q8BWW9;G3UXH4;G3UYD6	G3UZM9;Q8BWW9;G3UX	Serine/threonine-protein kinase N2	Pkn2
24.317	24.239	24.81909	G3X9S2;A0A0R4J1Q7;P06802;E9QQ26	G3X9S2;A0A0R4J1Q7;P06	Ectonucleotide pyrophosphatase/phosphodi	Enpp1
25.98251	25.99118	25.92931	G3XA48;P58044;H3BLF8;H3BLP1;H3BK	G3XA48;P58044;H3BLF8	Isopentenyl-diphosphate Delta-isomerase 1	Idi1
27.98142	27.90311	27.97596	G5E850;P56395;E0CY88	G5E850;P56395	Cytochrome b-5, isoform CRA_a;Cytochrome	Cyb5a
27.85043	27.61807	27.75175	O35226	O35226	26S proteasome non-ATPase regulatory sub	Psmd4
27.01903	27.3662	27.32389	O35685	O35685	Nuclear migration protein nudC	Nudc
26.5389	26.87408	27.32389	O54774	O54774	AP-3 complex subunit delta-1	Ap3d1
24.92705	24.32389	24.63755	O88968	O88968	Transcobalamin-2	Tcn2
24.7543	25.08942	24.84805	P12399;A0A286YDF0;A0A286YD41;A0A	P12399;A0A286YDF0	Protein CTLA-2-alpha;Uncharacterized prote	Ctla2a
23.57542	24.24627	23.62087	P17426;A0A140LIG7	P17426	AP-2 complex subunit alpha-1	Ap2a1
27.65949	27.60399	27.58978	P21995	P21995	Embigin	Emb
25.85519	26.19272	26.30487	P23591	P23591	GDP-L-fucose synthase	Tsta3
25.05258	24.77455	24.84805	P24638;B7ZCF4;B7ZCF5	P24638	Lysosomal acid phosphatase	Acp2
25.7024	25.37119	26.01052	P32020	P32020	Non-specific lipid-transfer protein	Scp2
26.34266	26.18896	25.96939	P32233;F7BQ65	P32233	Developmentally-regulated GTP-binding pro	Drg1
30.40324	30.46295	30.40324	P34884	P34884	Macrophage migration inhibitory factor	Mif
30.18423	30.20769	30.06085	P41731;A0A1W2P8C6	P41731;A0A1W2P8C6	CD63 antigen;Tetraspanin (Fragment)	Cd63
27.98685	27.60399	27.87994	P42669	P42669	Transcriptional activator protein Pur-alpha	Pura
26.4346	26.417	26.77706	P46467	P46467	Vacuolar protein sorting-associated protein	Vps4b
24.74919	25.03591	24.84326	P58021;E9PZ69	P58021;E9PZ69	Transmembrane 9 superfamily member 2;Tr	Tm9sf2
29.70306	29.66628	29.64065	P60867	P60867	40S ribosomal protein S20	Rps20
26.89735	27.4073	27.86231	P61620;A0A0A6YVQ7	P61620	Protein transport protein Sec61 subunit alph	Sec61a1
26.49534	26.32217	26.35279	P62965	P62965	Cellular retinoic acid-binding protein 1	Crabp1
27.71947	27.64581	27.56092	P63024;B0QZN5;P63044;A0A0N4SUV3;	P63024;B0QZN5;P63044	Vesicle-associated membrane protein 3;Vesi	Vamp3;Vamp2
25.40406	25.62366	25.23169	P63328;E9Q6P2;G3X8U7;E0CZ78;P4845	P63328;E9Q6P2;G3X8U7;	Serine/threonine-protein phosphatase 2B ca	Ppp3ca;Ppp3cb
27.65949	27.47073	28.11644	P68037;D3YZS3	P68037;D3YZS3	Ubiquitin-conjugating enzyme E2 L3	Ube2l3
24.27498	NaN	23.65403	P97310	P97310	DNA replication licensing factor MCM2	Mcm2
26.07112	26.02749	26.16806	Q05186	Q05186	Reticulocalbin-1	Rcn1
25.59263	25.44884	25.64856	Q05BC8;A2A4N5;A2A4N8;A2A4N7;K3W	Q05BC8;A2A4N5;A2A4N8;	Nbr1 protein;Next to BRCA1 gene 1 protein	Nbr1
25.79452	25.79699	25.85994	Q0PD20;B1AQD3;Q64008;B1AQD4;F7B	Q0PD20;B1AQD3;Q64008;	Rab34;Ras-related protein Rab-34	Rab34
25.1488	25.55508	25.13712	Q2TBE6	Q2TBE6	Phosphatidylinositol 4-kinase type 2-alpha	Pi4k2a
NaN	NaN	NaN	Q3TJ55;Q80W45;Q00262;D3YY94;A0A0	Q3TJ55;Q80W45;Q00262;	Syntaxin-2;Syntaxin-2 (Fragment)	Stx2
25.7415	25.48922	25.09748	Q3TTF6;H3BKU1;G3UWS4;Q7TNP2;H3B	Q3TTF6;H3BKU1;G3UWS4	Serine/threonine-protein phosphatase 2A 6	Ppp2r1b
24.20955	24.46139	24.2535	Q3U0V2	Q3U0V2	Tumor necrosis factor receptor type 1-assoc	Tradd
29.95394	29.94	29.85935	Q3U8S1;A2APM5;A2APM3;A2APM4;E9	Q3U8S1;A2APM5;A2APM	CD44 antigen	Cd44
25.08537	25.06085	25.1488	Q3UKN6;P81117	Q3UKN6;P81117	Nucleobindin-2	Nucb2
26.08942	25.88809	26.22251	Q3UM45;A0A087WRA7;F6TGJ2	Q3UM45;A0A087WRA7	Protein phosphatase 1 regulatory subunit 7;	Ppp1r7
26.16806	26.24627	26.03381	Q5EG47;Q8BRK8	Q5EG47	5-AMP-activated protein kinase catalytic sub	Prkaa1
27.2803	26.55655	27.10149	Q5SSP3;O35874	Q5SSP3;O35874	Amino acid transporter;Neutral amino acid t	Slc1a4
28.43539	28.41538	28.27586	Q60817;P70670	Q60817;P70670	Nascent polypeptide-associated complex su	Naca
26.4073	26.64581	26.52552	Q60973;A2AFI9;A2AFJ1;F6ZLC6;F6U539	Q60973;A2AFI9;A2AFJ1;F	Histone-binding protein RBBP7	Rbbp7
27.85043	28.258	28.23991	Q61009;F7C5U2	Q61009	Scavenger receptor class B member 1	Scarb1
26.23535	26.48001	26.42822	Q61205;D3Z7E6;D3Z2X5;Q8CA83	Q61205;D3Z7E6;D3Z2X5;	Platelet-activating factor acetylhydrolase IB	Pafah1b3
26.96499	26.71293	27.47846	Q62086	Q62086	Serum paraoxonase/arylesterase 2	Pon2
26.1946	26.10149	26.32732	Q64133	Q64133	Amine oxidase [flavin-containing] A	Maoa

27.55362	27.5389	27.61807	Q64310;E0CXD9;F7CH13	Q64310;E0CXD9;F7CH13	Surfeit locus protein 4;Surfeit locus protein 4	Surf4
26.391	26.26607	26.67303	Q6P1B9;O08539	Q6P1B9;O08539	Bin1 protein;Myc box-dependent-interacting	Bin1
25.36453	25.45513	25.78207	Q6P9R2	Q6P9R2	Serine/threonine-protein kinase OSR1	Oxsr1
28.54628	28.70306	28.39509	Q6ZWZ4;P47964;D3YX71	Q6ZWZ4;P47964	60S ribosomal protein L36	Rpl36
22.75328	22.68859	23.50446	Q7TNC4;E9Q715;Q05CX5;D3Z765;A0A0	Q7TNC4;E9Q715;Q05CX5	Putative RNA-binding protein Luc7-like 2;Luc	Luc7l2
24.6811	24.56384	24.83846	Q80ZW2	Q80ZW2	Protein THEM6	Them6
25.92479	25.77957	25.38443	Q8BKE6	Q8BKE6	Cytochrome P450 20A1	Cyp20a1
25.54628	25.52552	25.76951	Q8BM72;D3Z0Y0	Q8BM72	Heat shock 70 kDa protein 13	Hspa13
25.97378	26.54628	25.83364	Q8BP48;A0A0G2JFL1	Q8BP48	Methionine aminopeptidase 1	Metap1
25.56384	25.24627	25.80193	Q8BQ47;D3Z0T5	Q8BQ47;D3Z0T5	Protein canopy homolog 4;Protein canopy h	Cnpy4
25.07316	25.46762	25.73117	Q8BT07	Q8BT07	Centrosomal protein of 55 kDa	Cep55
25.73376	25.91342	25.76699	Q8BYU6	Q8BYU6	Torsin-1A-interacting protein 2	Tor1aip2
26.86231	27.41538	27.22619	Q8C3X8	Q8C3X8	Lipase maturation factor 2	Lmf2
24.83364	24.91798	24.56964	Q8CCJ3	Q8CCJ3	E3 UFM1-protein ligase 1	Ufl1
25.76951	25.87643	26.16039	Q8CE08;A0A1L1SUT0	Q8CE08;A0A1L1SUT0	Prostatic acid phosphatase;Prostatic acid ph	Acpp
25.02749	24.93607	24.97158	Q8CIB5;A6X941;A6X940	Q8CIB5;A6X941	Fermitin family homolog 2;Fermitin family h	Fermt2
25.85994	26.1795	25.37451	Q8K0C9	Q8K0C9	GDP-mannose 4,6 dehydratase	Gmds
NaN	23.40406	NaN	Q8QZR4;A0A1L1SUK4	Q8QZR4	Out at first protein homolog	Oaf
NaN	NaN	23.68646	Q8R0Y6;A0A0N4SW56	Q8R0Y6	Cytosolic 10-formyltetrahydrofolate dehydro	Aldh1l1
27.90885	27.90885	27.39917	Q8R5C5;E0CZD4;E0CYB4	Q8R5C5	Beta-centractin	Actr1b
25.49838	25.59263	26.10149	Q8VHE0	Q8VHE0	Translocation protein SEC63 homolog	Sec63
25.44569	25.53148	25.85043	Q91WG4	Q91WG4	Elongator complex protein 2	Elp2
26.33926	26.65949	26.04009	Q91YN9;A0A087WNX9	Q91YN9	BAG family molecular chaperone regulator 2	Bag2
25.96058	26.07316	25.96499	Q91YZ8;A3KFU5;A3KFU8;Q6PHQ9;D3Z5	Q91YZ8;A3KFU5;A3KFU8;(	Polyadenylate-binding protein	Pabpc4;Pab
27.07112	27.04009	27.12139	Q91ZJ5	Q91ZJ5	UTP--glucose-1-phosphate uridylyltransferas	Ugp2
28.31095	28.14102	28.14589	Q921J2	Q921J2	GTP-binding protein Rheb	Rheb
26.77706	26.69975	26.82639	Q924B0;Q80ZJ2;O55023;A0A0A6YW07	Q924B0;Q80ZJ2;O55023	Inositol-1-monophosphatase;Inositol monop	Impa1
25.89966	26.06291	25.9955	Q99JR1	Q99JR1	Sideroflexin-1	Sfxn1
24.20955	25.01478	25.17188	Q99JY8	Q99JY8	Phospholipid phosphatase 3	Plpp3
25.67842	25.78706	25.83605	Q99K30	Q99K30	Epidermal growth factor receptor kinase sub	Eps8l2
26.56238	26.77706	26.55215	Q99KF1;A0A286YDS5	Q99KF1;A0A286YDS5	Transmembrane emp24 domain-containing	Tmed9
25.92025	25.93157	26.27142	Q99LE6	Q99LE6	ATP-binding cassette sub-family F member 2	Abcf2
24.82396	25.1946	24.90885	Q9CQ92;G3X9U9;D3YZ32	Q9CQ92;G3X9U9	Mitochondrial fission 1 protein	Fis1
26.67303	26.86231	26.67303	Q9CQD1;A2A5F5;A2A5F6	Q9CQD1	Ras-related protein Rab-5A	Rab5a
26.2589	26.1035	26.31527	Q9CQT1;F6YY88;F8WI95	Q9CQT1;F6YY88	Methylthioribose-1-phosphate isomerase;M	Mri1
27.05051	27.10149	27.16997	Q9CQU0	Q9CQU0	Thioredoxin domain-containing protein 12	Txndc12
30.61982	30.56638	30.55728	Q9CR57;A0A1L1SUF6	Q9CR57	60S ribosomal protein L14	Rpl14
24.64306	24.1795	24.69179	Q9CRB1;O54974;D3Z141	Q9CRB1;O54974;D3Z141	Galectin;Galectin-7;Galectin (Fragment)	Lgals7
28.43539	27.95394	27.83846	Q9CWF2	Q9CWF2	Tubulin beta-2B chain	Tubb2b
25.22435	24.97158	25.06497	Q9CY00;A2AKQ8;Q99J38	Q9CY00;A2AKQ8;Q99J38	Tetratricopeptide repeat protein 30B;Tetrat	Ttc30b;Ttc30a2
25.23169	25.5285	24.44253	Q9CZT5	Q9CZT5	Vasorin	Vasn
24.85757	24.97158	25.21697	Q9CZW5	Q9CZW5	Mitochondrial import receptor subunit TOM	Tomm70
25.7103	25.98033	25.80931	Q9CZX7	Q9CZX7	Type 2 phosphatidylinositol 4,5-bisphosphat	Pip4p2
26.54922	26.82639	26.76446	Q9CZY3;B7ZBY7;E9PY39;B2KF55;A6X92	Q9CZY3;B7ZBY7;E9PY39;B	Ubiquitin-conjugating enzyme E2 variant 1;P	Ube2v1
25.39428	25.32732	25.32732	Q9D024;B1AR93;F7C265	Q9D024	Coiled-coil domain-containing protein 47	Ccdc47
27.24445	27.12139	27.45513	Q9D0M3	Q9D0M3	Cytochrome c1, heme protein, mitochondria	Cyc1
NaN	NaN	NaN	Q9D1D6	Q9D1D6	Collagen triple helix repeat-containing prote	Cthrc1
25.95615	25.86231	25.98251	Q9D6U8	Q9D6U8	Protein FAM162A	Fam162a
28.01903	28.14102	28.1795	Q9D8L3;Q62186	Q9D8L3;Q62186	Signal sequence receptor, delta;Translocon-a	Ssr4
26.71293	26.48001	26.11545	Q9D8U8	Q9D8U8	Sorting nexin-5	Snx5
25.74407	25.69179	25.51953	Q9DBZ5;Q3TY56;A0A140LJ59	Q9DBZ5;Q3TY56	Eukaryotic translation initiation factor 3 sub	Eif3k
26.45826	26.51202	26.37451	Q9DC16;F6SLP4	Q9DC16;F6SLP4	Endoplasmic reticulum-Golgi intermediate c	Ergic1
NaN	NaN	NaN	Q9DCA5;F6WEP7	Q9DCA5;F6WEP7	Ribosome biogenesis protein BRX1 homolog	Brix1
24.47383	24.35783	24.8811	Q9EPL2	Q9EPL2	Calsyntenin-1	Clstn1
25.97815	26.09346	26.08942	Q9EPT5	Q9EPT5	Solute carrier organic anion transporter fam	Slco2a1
26.28207	25.85757	26.40243	Q9ER38;M0QWH2;A0A087WRX4;A0A0	Q9ER38;M0QWH2	Torsin-3A	Tor3a
26.31872	26.29439	26.78955	Q9ERE7;D3YVR4	Q9ERE7;D3YVR4	LRP chaperone MESD;LRP chaperone MESD	Mesd
23.74919	23.54038	22.93157	Q9JHI5	Q9JHI5	Isovaleryl-CoA dehydrogenase, mitochondria	Ivd
24.42342	24.30313	24.58118	Q9JI10;Q9JI11	Q9JI10;Q9JI11	Serine/threonine-protein kinase 3;Serine/th	Stk3;Stk4
25.69179	25.37782	25.64306	Q9JKB1;P58321	Q9JKB1;P58321	Ubiquitin carboxyl-terminal hydrolase isozyn	Uchl3;Uchl4
24.64306	24.89966	25.1795	Q9QXJ2;E9QJX9;Q9WVL2	Q9QXJ2;E9QJX9;Q9WVL2	Signal transducer and activator of transcript	Stat2
NaN	NaN	NaN	Q9R001	Q9R001	A disintegrin and metalloproteinase with thr	Adamts5
26.33926	26.13712	26.18707	Q9R0N0	Q9R0N0	Galactokinase	Galk1
27.97596	27.77077	27.49381	Q9R1P1;D3YUM8	Q9R1P1	Proteasome subunit beta type-3	Psmb3
25.54922	25.1946	26.19272	Q9WUK2	Q9WUK2	Eukaryotic translation initiation factor 4H	Eif4h
26.88577	27.04009	27.16997	Q9WUM3;D3YUG6	Q9WUM3	Coronin-1B	Coro1b
26.27498	26.35616	26.28912	Q9WUM5;A0A0N4SVU4	Q9WUM5	Succinate--CoA ligase [ADP/GDP-forming] su	Suclg1
24.69179	25.2571	24.60964	Q9Z175;E9Q0X7	Q9Z175;E9Q0X7	Lysyl oxidase homolog 3	Loxl3
26.10149	26.21512	26.44095	Q9Z1D1	Q9Z1D1	Eukaryotic translation initiation factor 3 sub	Eif3g
25.04843	25.02327	24.51051	Q9Z1G3	Q9Z1G3	V-type proton ATPase subunit C 1	Atp6v1c1
24.42982	24.06085	24.1488	A0A087WQF8;A0A087WP48;A0A087WS	A0A087WQF8;A0A087WP	Kinectin	Ktn1
26.01691	25.93607	25.92025	A0A087WQS2;Q9CQC6;A0A087WPF9;A	A0A087WQS2;Q9CQC6	Basic leucine zipper and W2 domain-contain	Bzw1
27.391	27.10149	27.22619	A0A0G2JDI4;O09172;H3BJA3;F6VNW5	A0A0G2JDI4;O09172;H3B	Glutamate--cysteine ligase regulatory subun	Gclm
24.78457	26.80193	25.57254	A0A0J9YUZ4;P63158;A0A0J9YUD8;D3YZ	A0A0J9YUZ4;P63158;A0A	High mobility group protein B1 (Fragment);H	Hmgb1
24.77957	25.12928	25.06497	A0A0N4SVL9;Q9CQR6;A0A0N4SVE2;A0/	A0A0N4SVL9;Q9CQR6	Serine/threonine-protein phosphatase;Serin	Ppp6c
27.15074	26.87408	26.87408	A0A0N4SW28;Q9DAS9;A0A0N4SVT3	A0A0N4SW28;Q9DAS9;A0	Guanine nucleotide-binding protein subunit	Gng12
26.85043	26.47228	25.80193	A0A0N4SW73;Q8R361	A0A0N4SW73;Q8R361	Rab11 family-interacting protein 5	Rab11fip5
25.2856	25.01903	25.03171	A0A0R4IZY0;Q8C1A5	A0A0R4IZY0;Q8C1A5	Thimet oligopeptidase	Thop1
26.11347	26.03591	25.78457	A0A0R4J005;E9QP62;Q99JW4;Q9D7B2;	A0A0R4J005;E9QP62;Q99	LIM and senescent cell antigen-like-containi	Lims1
25.1332	25.14492	24.65949	A0A0R4J034;Q99K01;D3YZA7	A0A0R4J034;Q99K01	MCG129810, isoform CRA_c;Pyridoxal-depe	Pdxdc1
24.56964	25.08132	24.70767	A0A0R4J1E3;Q9QXS6;F7CPL2	A0A0R4J1E3;Q9QXS6	Drebrin	Dbn1
24.79452	24.58692	24.75939	A0A140LHA2;Q9WVA3;A0A140LIM5;A0	A0A140LHA2;Q9WVA3;A0	Mitotic checkpoint protein BUB3	Bub3
27.15074	27.04009	26.98685	A0A1B0GS58;Q9CQM9;A0A1B0GSD3;A(	A0A1B0GS58;Q9CQM9	Glutaredoxin-3	Glrx3
25.03591	24.76446	24.60399	A0A1W2P7Q6;Q9CRT8;A0A1W2P7V1	A0A1W2P7Q6;Q9CRT8	Exportin-T	Xpot
25.17188	25.55215	25.5285	A2AUK7;A2AUK8;A2AUK6;A2AUK5;E9P\	A2AUK7;A2AUK8;A2AUK6	Band 4.1-like protein 1	Epb41l1
23.05092	NaN	23.391	A2AWF8;A2AWF9;E9Q4S7;Q64455	A2AWF8;A2AWF9;E9Q4S7	Receptor-type tyrosine-protein phosphatase	Ptprj
24.6811	24.85281	25.00196	B0R030;Q9D3L3;O09044;E9Q8A1;B0R0	B0R030;Q9D3L3;O09044;E	Synaptosomal-associated protein (Fragment	Snap23
24.93607	25.1055	25.30661	D3YU17;Q8VCM8	D3YU17;Q8VCM8	Nicalin	Ncln

25.75685	25.84086	26.20023	D3YUV1;Q99J45;A0A0J9YTX8;A0A0J9YU	D3YUV1;Q99J45	Nuclear receptor-binding protein	Nrbp1
NaN	NaN	NaN	D3YUY3;F8WI18;P70182;F8WHW6;O70	D3YUY3;F8WI18;P70182	Phosphatidylinositol 4-phosphate 5-kinase ty	Pip5k1a
25.48922	25.27853	25.59833	D3Z0F5;O88545;F6QK86	D3Z0F5;O88545	COP9 signalosome complex subunit 6	Cops6
24.62645	24.1795	23.83846	D3Z1U9;D3YUM1;Q91YT0;D3Z0K1;D3Y	D3Z1U9;D3YUM1;Q91YT0	NADH dehydrogenase [ubiquinone] flavopro	Ndufv1
28.35783	28.27142	28.31527	D3Z6I8;E9Q7Q3	D3Z6I8;E9Q7Q3	Tropomyosin alpha-3 chain	Tpm3
NaN	23.29614	NaN	D3Z6S1;Q8BM55	D3Z6S1;Q8BM55	Transmembrane protein 214	Tmem214
28.86821	29.0427	28.94	D3Z7C6;Q9R0Q7	D3Z7C6;Q9R0Q7	Prostaglandin E synthase 3	Ptges3
24.27498	24.61526	24.84805	D6REG4;Q91Z53;B1AXQ0	D6REG4;Q91Z53	Glyoxylate reductase/hydroxypyruvate redu	Grhpr
24.92705	24.75939	25.24627	E9PUF7;Q61210;F6ZN61	E9PUF7;Q61210;F6ZN61	Rho guanine nucleotide exchange factor 1	Arhgef1
25.37451	25.48616	25.12928	E9PUX0;Q80WJ7;F6ZQL0;F6QHD1;F6QF	E9PUX0;Q80WJ7	Protein LYRIC	Mtdh
25.31354	25.38113	25.69975	E9PV22;Q505F5	E9PV22;Q505F5	Leucine-rich repeat-containing protein 47	Lrrc47
25.26069	24.87643	24.36453	E9PVK4;Q0HA38	E9PVK4;Q0HA38	Tetratricopeptide repeat protein 21B	Ttc21b
24.62087	24.52253	24.74919	E9PZC3;Q923D2;E9PZC4;A0A0U1RPU7	E9PZC3;Q923D2;E9PZC4	Flavin reductase (NADPH)	Blvrb
NaN	NaN	NaN	E9Q151;Q8VBW1;F6UKB4;E9Q6T2	E9Q151;Q8VBW1;F6UKB4	Transporter;Sodium- and chloride-depender	Slc6a8;Slc6a
24.18707	24.32389	24.37119	E9Q1F5;A0A1L1SUF0	E9Q1F5	Myosin VC	Myo5c
25.16423	23.44253	25.62087	E9Q5L3;Q9DBL1;A0A140LHL4	E9Q5L3;Q9DBL1	Short/branched chain-specific acyl-CoA dehy	Acadsb
24.95837	24.417	25.01478	E9Q682;E9PY46	E9Q682;E9PY46	Intraflagellar transport protein 140 homolog	Ift140
28.15074	28.47073	28.41538	F6QL70;Q5M8M8;P47915;A0A1L1SS27;	F6QL70;Q5M8M8;P47915	60S ribosomal protein L29	Gm17669;Rpl29
25.46451	25.81176	25.80193	F7BX26;Q60676	F7BX26;Q60676	Serine/threonine-protein phosphatase (Frag	Ppp5c
24.12534	23.55215	NaN	G3UW30;F6ZHD8;Q9D6Y9	G3UW30;F6ZHD8;Q9D6Y9	1,4-alpha-glucan-branching enzyme	Gbe1
28.55362	28.54995	28.64238	G3UYV7;P62858	G3UYV7;P62858	40S ribosomal protein S28 (Fragment);40S ri	Rps28
26.08942	26.18329	26.24808	G3X977;Q61703;Q3UEG7	G3X977;Q61703;Q3UEG7	Inter-alpha trypsin inhibitor, heavy chain 2;I	Itih2
25.22435	24.67573	25.68913	G3X9J6;E9Q9M1;Q3V1L4	G3X9J6;E9Q9M1;Q3V1L4	5-nucleotidase, cytosolic II, isoform CRA_b;C	Nt5c2
27.44727	27.5969	27.48616	G5E8R1;G5E8R0;E9Q455	G5E8R1;G5E8R0;E9Q455	Tropomyosin 1, alpha, isoform CRA_j;Tropor	Tpm1
22.76344	NaN	NaN	G5E8R3;E9QPD7;Q05920	G5E8R3;E9QPD7;Q05920	Pyruvate carboxylase;Pyruvate carboxylase,	Pcx;Pc
26.2535	26.01265	26.29088	K9J7B2;Q64435;D3YZE6;D3Z7G0;D3YZ9	K9J7B2;Q64435;D3YZE6;D	UDP-glucuronosyltransferase;UDP-glucuron	Ugt1a6b;Ugt1a6
23.417	23.48001	23.81909	O08582	O08582	GTP-binding protein 1	Gtpbp1
26.46139	26.65949	26.86231	O35344	O35344	Importin subunit alpha-4	Kpna3
25.64581	25.55801	25.87173	O54984;A0A1B0GRE1	O54984	ATPase Asna1	Asna1
24.417	24.97158	24.60964	O70572;D6RGM4	O70572	Sphingomyelin phosphodiesterase 2	Smpd2
24.98902	NaN	NaN	O88531;B1B0P8;B1B0P9	O88531	Palmitoyl-protein thioesterase 1	Ppt1
27.38278	26.4999	27.20769	O88543;F6YCA7;D3Z036	O88543	COP9 signalosome complex subunit 3	Cops3
24.02749	25.45826	25.62087	O89017	O89017	Legumain	Lgmn
28.06599	28.40324	27.99226	P00405	P00405	Cytochrome c oxidase subunit 2	Mtco2
27.92592	28.04009	27.87408	P02463;A0A1B0GRC0;A0A1B0GSI7	P02463;A0A1B0GRC0;A0A	Collagen alpha-1(IV) chain;Collagen alpha-1(	Col4a1
NaN	NaN	NaN	P03975	P03975	IgE-binding protein	Iap
26.38607	26.61807	26.41054	P10922	P10922	Histone H1.0	H1f0
27.60399	27.21697	27.64581	P12787	P12787	Cytochrome c oxidase subunit 5A, mitochon	Cox5a
24.28207	24.12534	24.27498	P13011;Q99PL7;P13516;Q6T707	P13011;Q99PL7;P13516	Acyl-CoA desaturase 2;Acyl-CoA desaturase	Scd2;Scd3;Scd1
27.29789	27.3662	27.26249	P19783;M0QWX7	P19783	Cytochrome c oxidase subunit 4 isoform 1, n	Cox4i1
25.42662	25.48922	25.4008	P27048;P63163;A0A0G2JGN4	P27048;P63163;A0A0G2J(	Small nuclear ribonucleoprotein-associated	Snrpb;Snrpn
24.10149	NaN	23.31008	P28076;A0A0R4J256;G3UYK5	P28076;A0A0R4J256	Proteasome subunit beta type-9;Proteasom	Psmb9
NaN	NaN	NaN	P28301	P28301	Protein-lysine 6-oxidase	Lox
24.97158	25.18329	24.93607	P28571;E9Q517;E9Q3V0;E9QA02;E9Q3	P28571;E9Q517;E9Q3V0;E	Sodium- and chloride-dependent glycine tra	Slc6a9
NaN	NaN	NaN	P28667	P28667	MARCKS-related protein	Marcksl1
25.12928	25.23169	25.20955	P30285;E9Q9E4;E9PZX7;D3YWB0;D6RH	P30285;E9Q9E4;E9PZX7	Cyclin-dependent kinase 4;Cyclin-dependent	Cdk4
28.01372	28.09144	27.92592	P35456;A0A0U1RNN0	P35456;A0A0U1RNN0	Urokinase plasminogen activator surface rec	Plaur
26.80193	27.06085	26.99766	P39876;E9QAB2	P39876	Metalloproteinase inhibitor 3	Timp3
31.81955	31.72923	31.50142	P40240	P40240	CD9 antigen	Cd9
NaN	22.58233	22.46762	P43275	P43275	Histone H1.1	Hist1h1a
25.66763	25.98251	26.09949	P45878	P45878	Peptidyl-prolyl cis-trans isomerase FKBP2	Fkbp2
30.35153	30.13614	29.92592	P47955	P47955	60S acidic ribosomal protein P1	Rplp1
25.61246	25.72598	25.38113	P58742	P58742	Aladin	Aaas
26.28736	26.47537	26.61807	P59325;Q8BVV6;A0A1Y7VKT5;A0A1Y7V	P59325;Q8BVV6	Eukaryotic translation initiation factor 5	Eif5
25.239	25.04843	24.76951	P61211;F8WIB1;E9Q006	P61211;F8WIB1	ADP-ribosylation factor-like protein 1;ADP-ri	Arl1
29.17712	29.14346	29.1866	P61514;A0A1D5RL86	P61514;A0A1D5RL86	60S ribosomal protein L37a;60S ribosomal p	Rpl37a
27.24445	27.24445	27.42342	P61971	P61971	Nuclear transport factor 2	Nutf2
24.71293	24.76951	24.31008	P62317	P62317	Small nuclear ribonucleoprotein Sm D2	Snrpd2
28.75812	28.77392	28.83244	P62774;A0A0J9YV46	P62774	Myotrophin	Mtpn
29.41538	29.509	29.38895	P62849;A0A286YEB7	P62849;A0A286YEB7	40S ribosomal protein S24	Rps24
30.27586	30.28692	30.21928	P62855	P62855	40S ribosomal protein S26	Rps26
25.98468	25.78457	24.26785	P62869;A6PWE0	P62869	Elongin-B	Elob
26.95394	27.391	27.56092	P67871;G3UXG7;G3UXU2;G3UZJ5;G3UZ	P67871;G3UXG7;G3UXU2;	Casein kinase II subunit beta;Casein kinase II	Csnk2b
25.59263	25.34773	25.39428	P70122;D6REV5;F6TN03	P70122	Ribosome maturation protein SBDS	Sbds
23.94056	23.417	24.08537	P70441	P70441	Na(+)/H(+) exchange regulatory cofactor NH	Slc9a3r1
28.3452	27.87994	27.52402	P84084;D3YV25;E9Q2C2	P84084	ADP-ribosylation factor 5	Arf5
NaN	22.90976	NaN	Q00915	Q00915	Retinol-binding protein 1	Rbp1
25.23535	25.02749	24.95394	Q03350	Q03350	Thrombospondin-2	Thbs2
25.82639	25.74919	25.81421	Q07417	Q07417	Short-chain specific acyl-CoA dehydrogenase	Acads
25.19835	26.20396	25.89735	Q3TEW6;A0A0A6YWA2;A0A0A6YXX8;A(	Q3TEW6;A0A0A6YWA2	Myelin protein zero-like protein 1;Myelin pr	Mpzl1
24.26785	23.76951	24.09346	Q3TKX1;Q9R1Q9;B7FAU3;B7FAU7;F6X9	Q3TKX1;Q9R1Q9;B7FAU3;	ATPase, H+ transporting, lysosomal accesso	Atp6ap1
25.24264	NaN	NaN	Q3U0V1	Q3U0V1	Far upstream element-binding protein 2	Khsrp
25.01052	25.239	25.89504	Q3U816;Q9Z2G9	Q3U816;Q9Z2G9	Oxidoreductase HTATIP2	Htatip2
23.94056	24.45513	24.67034	Q3UA06;A0A1Y7VLR4	Q3UA06	Pachytene checkpoint protein 2 homolog	Trip13
27.71293	27.51653	27.5389	Q3UMR5	Q3UMR5	Calcium uniporter protein, mitochondrial	Mcu
26.2856	26.26964	26.30835	Q3UN02;B0V2Q9	Q3UN02	Lysocardiolipin acyltransferase 1	Lclat1
25.18329	NaN	23.96719	Q3UVG3	Q3UVG3	Protein FAM91A1	Fam91a1
24.10949	24.1795	25.06497	Q3UW66;Q99J99	Q3UW66;Q99J99	Sulfurtransferase;3-mercaptopyruvate sulfu	Mpst
28.01903	28.21697	28.4512	Q61990;E9Q7D8;P57722;A0A087WR61;	Q61990;E9Q7D8;P57722	Poly(rC)-binding protein 2;Poly(rC)-binding p	Pcbp2;Pcbp3
26.99766	27.47846	27.29789	Q62159;H3BL56;A0A0G2JEP8	Q62159;H3BL56;A0A0G2J	Rho-related GTP-binding protein RhoC	Rhoc
26.21141	26.37782	26.67303	Q62348	Q62348	Translin	Tsn
25.94726	26.20955	26.18518	Q62446;A0A1Y7VLK0;A0A1Y7VMJ9;A0A	Q62446;A0A1Y7VLK0	Peptidyl-prolyl cis-trans isomerase FKBP3;Pe	Fkbp3
25.37782	26.05879	25.7415	Q6PA06;E9QND8	Q6PA06;E9QND8	Atlastin-2	Atl2
25.81909	25.67034	25.82396	Q6PD26	Q6PD26	GPI transamidase component PIG-S	Pigs
24.76446	24.7543	24.71816	Q6PGL7;A0A0N4SUJ0;A0A0N4SV74	Q6PGL7;A0A0N4SUJ0;A0A	WASH complex subunit 2;WASH complex su	Washc2
24.08537	24.98902	24.77455	Q6ZPU9;H3BIY2;E0CXJ9;E0CXN7	Q6ZPU9	KIF1-binding protein	Kif1bp
28.88577	28.99226	29.02697	Q6ZWV7;G3UWD7	Q6ZWV7	60S ribosomal protein L35	Rpl35
25.32389	24.86703	25.2021	Q78HU3	Q78HU3	Multivesicular body subunit 12A	Mvb12a

25.72338	25.4008	25.82153	Q80U72;Q80VQ1	Q80U72	Protein scribble homolog	Scrib
NaN	24.1488	NaN	Q80W65	Q80W65	Proprotein convertase subtilisin/kexin type 9	Pcsk9
22.64966	23.94949	24.37119	Q8BGK6;A0A1D5RMA4;A0A1D5RMI7;A0	Q8BGK6;A0A1D5RMA4;A0	Y+L amino acid transporter 2	Slc7a6
25.77706	25.61526	25.61526	Q8BH43;B1AUN0;Q8R5H6	Q8BH43;B1AUN0	Wiskott-Aldrich syndrome protein family me	Wasf2
26.42342	26.57542	26.26069	Q8BH80;Q9QY76	Q8BH80;Q9QY76	Vesicle-associated membrane protein, assoc	Vapb
26.09346	25.49228	25.45826	Q8BIJ6;E9PWN3;E9PWN2	Q8BIJ6	Isoleucine--tRNA ligase, mitochondrial	Iars2
26.80193	26.93157	26.73892	Q8BMJ3;Q60872;Q3UTA4;Q3TQZ4;J3Q	Q8BMJ3;Q60872	Eukaryotic translation initiation factor 1A, X-	Eif1ax;Eif1a
25.94949	26.61807	26.89735	Q8BR63	Q8BR63	Protein FAM177A1	Fam177a1
26.33756	25.92705	26.44253	Q8BYH7;I6L974;A0A140LIS2;A0A140LHF	Q8BYH7;I6L974	TBC1 domain family member 17	Tbc1d17
25.64306	25.74407	26.01478	Q8C145;D3Z7N4	Q8C145	Zinc transporter ZIP6	Slc39a6
24.75939	24.59833	24.89041	Q8C9V5;Q8BGK0;Q61941;E9Q8F4	Q8C9V5;Q8BGK0;Q61941;	NAD(P) transhydrogenase, mitochondrial	Nnt
25.98251	25.73635	25.7543	Q8CAS9;H7BWY5	Q8CAS9;H7BWY5	Poly [ADP-ribose] polymerase 9	Parp9
25.46762	24.98468	25.94726	Q8CBY8	Q8CBY8	Dynactin subunit 4	Dctn4
23.65403	23.85757	23.391	Q8CG50;D3Z444;Q91Z34	Q8CG50;D3Z444;Q91Z34	Ras-related protein Rab-43;RIKEN cDNA 181	Rab43
23.79947	23.72858	23.21104	Q8CIH5	Q8CIH5	1-phosphatidylinositol 4,5-bisphosphate pho	Plcg2
26.22435	26.08537	25.96719	Q8JZU2;F6VVY4	Q8JZU2	Tricarboxylate transport protein, mitochond	Slc25a1
25.73892	26.16615	26.00838	Q8K094	Q8K094	Poliovirus receptor	Pvr
25.20583	25.1332	25.60399	Q8K2J7	Q8K2J7	RELT-like protein 1	Rell1
NaN	22.16116	NaN	Q8K2Y3	Q8K2Y3	Protein eva-1 homolog B	Eva1b
25.98685	25.85519	25.51352	Q8QZY6	Q8QZY6	Tetraspanin-14	Tspan14
28.72273	28.6764	28.78644	Q8R2Q8	Q8R2Q8	Bone marrow stromal antigen 2	Bst2
27.11148	27.58262	28.01372	Q8R2Y8	Q8R2Y8	Peptidyl-tRNA hydrolase 2, mitochondrial	Ptrh2
25.84805	25.88577	25.77455	Q8R4W6	Q8R4W6	Procollagen C-endopeptidase enhancer 2	Pcolce2
24.53445	24.30313	24.32389	Q8R502	Q8R502	Volume-regulated anion channel subunit LRI	Lrrc8c
24.90885	25.12139	25.417	Q8R574	Q8R574	Phosphoribosyl pyrophosphate synthase-ass	Prpsap2
24.55801	24.71293	24.10949	Q8R5F7	Q8R5F7	Interferon-induced helicase C domain-conta	Ifih1
25.09748	24.79452	25.66221	Q8R5H1	Q8R5H1	Ubiquitin carboxyl-terminal hydrolase 15	Usp15
27.51653	27.71947	27.43141	Q8R5J9	Q8R5J9	PRA1 family protein 3	Arl6ip5
26.99766	27.01903	26.83846	Q8R5L1;O35658	Q8R5L1;O35658	Complement component 1 Q subcomponent	C1qbp
26.26428	25.77455	26.35616	Q8VCN5	Q8VCN5	Cystathionine gamma-lyase	Cth
25.30313	25.09346	25.24989	Q8VEH6	Q8VEH6	COBW domain-containing protein 1	Cbwd1
25.50142	25.88809	25.94726	Q91VH6	Q91VH6	Protein MEMO1	Memo1
25.50142	25.92025	25.74663	Q91WS0	Q91WS0	CDGSH iron-sulfur domain-containing protei	Cisd1
23.35111	NaN	NaN	Q91XU3	Q91XU3	Phosphatidylinositol 5-phosphate 4-kinase t	Pip4k2c
25.67842	25.36786	24.94503	Q91YJ2	Q91YJ2	Sorting nexin-4	Snx4
24.49838	24.78457	24.21697	Q91YR9	Q91YR9	Prostaglandin reductase 1	Ptgr1
25.26069	25.77957	25.44884	Q91YS7;Q63932;M0QWN2;D3Z446;A0A	Q91YS7;Q63932;M0QWN	Dual-specificity mitogen-activated protein ki	Map2k2
25.49228	25.5285	25.65403	Q91YW3	Q91YW3	DnaJ homolog subfamily C member 3	Dnajc3
25.67842	25.68646	25.60116	Q91ZR1;P56371	Q91ZR1	Ras-related protein Rab-4B	Rab4b
25.49534	25.62645	25.42342	Q922Q4;Q922W5;A2ABZ2	Q922Q4	Pyrroline-5-carboxylate reductase 2	Pycr2
25.64581	25.56964	25.90426	Q923T9;A0A286YCW8;A0A286YCH4;A0A	Q923T9	Calcium/calmodulin-dependent protein kina	Camk2g
26.96499	27.1795	26.77706	Q924Z4;D3YTM0;D3Z0Z2;D3Z4M2	Q924Z4;D3YTM0;D3Z0Z2;	Ceramide synthase 2;Ceramide synthase 2 (F	Cers2
25.04843	24.87173	24.05258	Q99KN2;G3UYN9;G3UYR5;G3UYS4	Q99KN2;G3UYN9;G3UYR5	Probable cytosolic iron-sulfur protein assem	Ciao1
23.65403	23.85757	23.91342	Q99KW9	Q99KW9	T-cell immunomodulatory protein	Itfg1
26.01478	25.91114	26.16806	Q99LR1;D6RFU2;F7BHM8	Q99LR1;D6RFU2	Monoacylglycerol lipase ABHD12	Abhd12
23.71816	23.37782	23.91342	Q99M31	Q99M31	Heat shock 70 kDa protein 14	Hspa14
26.75175	26.86231	26.87408	Q9CQ22;A0A0A6YX02	Q9CQ22	Ragulator complex protein LAMTOR1	Lamtor1
25.61807	25.62087	26.13124	Q9CQ80;A8XY17;E9PXS9;A2A4K0;A2A4	Q9CQ80;A8XY17;E9PXS9;A	Vacuolar protein-sorting-associated protein	Vps25
NaN	NaN	24.33074	Q9CQB4;Q9D855	Q9CQB4;Q9D855	Cytochrome b-c1 complex subunit 7	Uqcrb
27.89735	27.85043	27.78332	Q9CQR2	Q9CQR2	40S ribosomal protein S21	Rps21
25.50446	25.2571	25.04009	Q9CQW1	Q9CQW1	Synaptobrevin homolog YKT6	Ykt6
29.20769	29.2535	29.08891	Q9CQW9	Q9CQW9	Interferon-induced transmembrane protein	Ifitm3
26.55508	26.83846	26.80193	Q9CR51	Q9CR51	V-type proton ATPase subunit G 1	Atp6v1g1
27.09144	27.18896	27.35783	Q9CR68	Q9CR68	Cytochrome b-c1 complex subunit Rieske, m	Uqcrfs1
25.39754	25.4008	25.77957	Q9CXE7;E9PXY3;D3Z615;Q8C5H9;Q3V4	Q9CXE7;E9PXY3	Transmembrane emp24 domain-containing	Tmed5
25.33074	25.2571	25.78457	Q9CZR8;Q9CX33	Q9CZR8	Elongation factor Ts, mitochondrial	Tsfm
23.99335	23.50446	23.62087	Q9CZV8;A2A536;A2A533;A2A550;Q8BH	Q9CZV8	F-box/LRR-repeat protein 20	Fbxl20
27.83244	27.12139	27.3662	Q9D1G1	Q9D1G1	Ras-related protein Rab-1B	Rab1b
25.51051	25.95172	25.67573	Q9D1M0	Q9D1M0	Protein SEC13 homolog	Sec13
26.71293	27.04009	26.77706	Q9D1M7	Q9D1M7	Peptidyl-prolyl cis-trans isomerase FKBP11	Fkbp11
26.01691	26.16039	26.30661	Q9D898	Q9D898	Actin-related protein 2/3 complex subunit 5-	Arpc5l
28.0296	28.33245	28.41941	Q9DBR7;A0A1W2P750	Q9DBR7	Protein phosphatase 1 regulatory subunit 12	Ppp1r12a
25.77706	25.52552	25.67034	Q9DC61;A2AIW9	Q9DC61;A2AIW9	Mitochondrial-processing peptidase subunit	Pmpca
27.71947	27.27142	27.34096	Q9DCT8;A0A0G2JF37	Q9DCT8	Cysteine-rich protein 2	Crip2
24.72858	25.11347	25.18707	Q9DCW2;D3Z7J3	Q9DCW2;D3Z7J3	Phospholipid scramblase 2;Phospholipid scra	Plscr2
23.28207	NaN	NaN	Q9EPR4;A2ANL1	Q9EPR4	Solute carrier family 23 member 2	Slc23a2
25.83846	25.45199	25.90196	Q9EQ80;D3Z0G0;E9PWJ6	Q9EQ80;D3Z0G0	NIF3-like protein 1	Nif3l1
28.34942	28.53519	29.24898	Q9EQC5	Q9EQC5	N-terminal kinase-like protein	Scyl1
24.78955	24.8044	24.74919	Q9JHK4	Q9JHK4	Geranylgeranyl transferase type-2 subunit a	Rabggta
NaN	NaN	22.44253	Q9JHW2	Q9JHW2	Omega-amidase NIT2	Nit2
25.82881	25.73892	26.51202	Q9JK38	Q9JK38	Glucosamine 6-phosphate N-acetyltransfera	Gnpnat1
25.29614	25.41054	24.77957	Q9JKB3	Q9JKB3	Y-box-binding protein 3	Ybx3
NaN	23.54038	NaN	Q9JLN9	Q9JLN9	Serine/threonine-protein kinase mTOR	Mtor
24.85281	24.48616	24.54038	Q9QZN4;A2A7H1;Z4YN00;A2A7G9	Q9QZN4;A2A7H1;Z4YN00;	F-box only protein 6;F-box only protein 6 (Fr	Fbxo6
24.37119	24.46139	25.01052	Q9R059;F2Z455	Q9R059;F2Z455	Four and a half LIM domains protein 3	Fhl3
NaN	NaN	NaN	Q9R061	Q9R061	Cytosolic Fe-S cluster assembly factor NUBP	Nubp2
25.6513	25.43936	25.52253	Q9WUL7	Q9WUL7	ADP-ribosylation factor-like protein 3	Arl3
NaN	NaN	NaN	Q9Z0J7	Q9Z0J7	Growth/differentiation factor 15	Gdf15
25.67034	25.66221	25.36453	Q9Z0L0	Q9Z0L0	Trophoblast glycoprotein	Tpbg
25.97158	25.94503	26.31181	A0A087WNP6;Q4VAA2;A0A087WRM0	A0A087WNP6;Q4VAA2	Protein CDV3	Cdv3
24.56964	25.239	24.68646	A0A087WQ01;Q69Z38	A0A087WQ01;Q69Z38	Pseudopodium-enriched atypical kinase 1	Peak1
23.98468	24.317	NaN	A0A087WSP7;A0A087WQZ1;Q8K310	A0A087WSP7;A0A087WQ	MCG121979, isoform CRA_a;Matrin-3	Matr3
26.12534	26.00838	25.98685	A0A0A0MQA5;P68368;A0A087WQS4;A0	A0A0A0MQA5;P68368	Tubulin alpha chain (Fragment);Tubulin alph	Tuba4a
23.68646	23.64306	24.08537	A0A0A0MQM7;O08746	A0A0A0MQM7;O08746	Matrilin-2	Matn2
24.63755	24.63755	24.95837	A0A0A6YXE3;A0A0A6YWB0;G3X9Q0;A0	A0A0A6YXE3;A0A0A6YWB	Muscleblind-like protein 1	Mbnl1
24.26069	24.1946	24.35783	A0A0F6AIX5;A0A1W2P7C8;Q8BVA5;A0A	A0A0F6AIX5;A0A1W2P7C8	Lipid droplet-associated hydrolase	Ldah
NaN	NaN	NaN	A0A0G2JEK2;P63254	A0A0G2JEK2;P63254	Cysteine-rich protein 1	Crip1
23.76951	24.7543	24.1946	A0A0G2JFP4;Q8K385	A0A0G2JFP4;Q8K385	Ferric-chelate reductase 1	Frrs1;FRRS1

27.49381	27.73892	27.62506	A0A0G2JFY0;A0A0A0MQ80;Q3UMC0;D	A0A0G2JFY0;A0A0A0MQ8	Spermatogenesis-associated protein 5	Spata5
23.58692	22.84038	22.50688	A0A0G2JGL8;Q99PU8	A0A0G2JGL8;Q99PU8	Putative ATP-dependent RNA helicase DHX3	Dhx30
25.03591	24.92705	25.07316	A0A0N4SVS6	A0A0N4SVS6	Cellular nucleic acid-binding protein	Cnbp
24.81909	25.39754	25.37782	A0A0R4J049;S4R295;F6QQQ6	A0A0R4J049	Protein arginine N-methyltransferase 5	Prmt5
24.40406	24.73376	24.31008	A0A0R4J138;P50429	A0A0R4J138;P50429	Arylsulfatase B	Arsb
24.87173	24.75939	24.94503	A0A0U1RNT6;A0A0U1RNK6;Q3THS6;A0	A0A0U1RNT6;A0A0U1RNK	S-adenosylmethionine synthase	Mat2a
25.239	25.63201	25.47073	A0A140LIT2;O88455;D3Z7G1;A0A140LI	A0A140LIT2;O88455;D3Z7	7-dehydrocholesterol reductase;7-dehydroc	Dhcr7
23.68646	NaN	23.44253	A0A1B0GRR8;Q62376;A0A1B0GR69;A0	A0A1B0GRR8;Q62376;A0A	U1 small nuclear ribonucleoprotein 70 kDa (	Snrnp70
26.34942	26.33756	26.47228	A0A1B0GS13;A0A1B0GT81;A0A1B0GTA	A0A1B0GS13;A0A1B0GT8	Apoptosis regulator BAX (Fragment);Apopto	Bax
24.20955	24.56964	23.74919	A0A1L1SRX2;O08739	A0A1L1SRX2;O08739	AMP deaminase;AMP deaminase 3	Ampd3
24.95837	24.80931	25.17188	A0A1L1SU22;Q9D1P4;A0A1L1STQ1;A0A	A0A1L1SU22;Q9D1P4;A0A	Cysteine and histidine-rich domain-containi	Chordc1
25.47383	25.47073	25.93382	A0A1Y7VN19;Q9CXR1	A0A1Y7VN19;Q9CXR1	Dehydrogenase/reductase SDR family memb	Dhrs7
NaN	23.91342	23.78955	A0A286YCV0;D3YV00;P98195;A0A286Y	A0A286YCV0;D3YV00;P98	Phospholipid-transporting ATPase	Atp9b
23.59833	23.66492	23.94056	A0A286YD87;Q01721	A0A286YD87;Q01721	Growth arrest-specific protein 1	Gas1
24.52253	24.56384	23.89504	A0A286YDB8;Q9CZX0	A0A286YDB8;Q9CZX0	Elongator complex protein 3	Elp3
24.36453	24.417	24.45513	A0A286YDI8;G3X972;A0A286YD08	A0A286YDI8;G3X972	Sec24 related gene family, member C (S. cer	Sec24c
25.52253	25.7024	24.78955	A0A286YED2;P53690	A0A286YED2;P53690	Matrix metalloproteinase;Matrix metallopro	Mmp14
24.44253	24.81909	24.57542	A2A7A7;Q8CFX1;A2A7A8	A2A7A7;Q8CFX1	GDH/6PGL endoplasmic bifunctional protein	H6pd
24.97596	24.89504	24.58692	A2AJH3;O70311;F7APP3	A2AJH3;O70311;F7APP3	Glycylpeptide N-tetradecanoyltransferase;G	Nmt2
25.07725	25.68378	25.47692	A2ASL8;Q7TQ95;A2ASM0;A2ASM1	A2ASL8;Q7TQ95;A2ASM0	Endoplasmic reticulum junction formation p	Lnpk
23.94949	24.08537	23.6971	A2AU61;A2AU62;Q64012;A2AU60	A2AU61;A2AU62;Q64012;	RNA-binding protein Raly (Fragment);RNA-b	Raly;Ra
24.317	NaN	NaN	A2AWI9;A2AWI7;Q8R3V5	A2AWI9;A2AWI7;Q8R3V5	Endophilin-B2	Sh3glb2
NaN	NaN	NaN	B1B1E3;Q8VDV0	B1B1E3;Q8VDV0	Integrin beta-like protein 1 (Fragment);Integ	Itgbl1
24.90426	24.98468	25.03171	B2RQS1;Q9ERG2	B2RQS1;Q9ERG2	Striatin-3	Strn3
27.0296	27.47073	26.22066	B2RXC1	B2RXC1	Trafficking protein particle complex subunit	Trappc11
30.29789	30.18423	30.28692	D3YTQ9;P62843	D3YTQ9;P62843	40S ribosomal protein S15	Rps15
25.59263	25.67842	25.68913	D3YWK1;Q80W47	D3YWK1;Q80W47	WD repeat domain phosphoinositide-intera	Wipi2
25.50749	25.69445	25.26069	D3Z0B9;A0A1B0GSU0;Q571I9;A0A1B0G	D3Z0B9;A0A1B0GSU0;Q5	Aldehyde dehydrogenase family 16 member	Aldh16a1
25.80686	25.72338	26.13712	D3Z783;P31750;D3YXX3;D3YYP9	D3Z783;P31750;D3YXX3	RAC-alpha serine/threonine-protein kinase	Akt1
25.2535	24.97158	24.84805	E9PUB7;D3YX87;Q2YDW2	E9PUB7;D3YX87;Q2YDW2	Protein misato homolog 1	Msto1
24.2535	24.53445	24.51051	E9PUQ9;F7AC58;E2JF22;F6PYU5;F6QY9	E9PUQ9;F7AC58;E2JF22;F	Piezo-type mechanosensitive ion channel co	Piezo1
23.6971	23.67573	23.48001	E9PXV7;E9PV41;O08808;F6XC54;D3Z07	E9PXV7;E9PV41;O08808;F	Protein diaphanous homolog 1	Diaph1
24.90426	25.2856	24.10949	E9PYX7;E9Q852;E9Q9C3;Q9QZQ1	E9PYX7;E9Q852;E9Q9C3;C	Afadin	Afdn
NaN	24.90426	NaN	E9Q0X4;Q8BLX7	E9Q0X4;Q8BLX7	Collagen alpha-1(XVI) chain	Col16a1
24.69179	24.78955	24.79452	E9Q6U4;Q3UWL8;E9Q4Q8;E9Q8R1;Q6F	E9Q6U4;Q3UWL8	Prefoldin subunit 4	Pfdn4
25.22066	25.60964	25.58692	E9Q855;Q3UXS0;O35609	E9Q855;Q3UXS0;O35609	Secretory carrier-associated membrane prot	Scamp3
23.80931	23.70767	24.18707	E9Q9G8;Q6NWV3	E9Q9G8;Q6NWV3	Intraflagellar transport protein 122 homolog	Ift122
NaN	NaN	NaN	E9QAM4;Q9Z184	E9QAM4;Q9Z184	Protein-arginine deiminase type-3	Padi3
25.50142	25.45199	25.64856	E9QAY5;A2RSX9;E9Q3G5;G5E8V9	E9QAY5;A2RSX9;E9Q3G5;	Predicted gene, 37240;ADP-ribosylation fact	Gm37240;Arfip
23.88577	23.49228	23.35111	E9QN99;Q8VCR7;A0A087WP24;A0A087	E9QN99;Q8VCR7;A0A087	Protein ABHD14B;Protein ABHD14B (Fragme	Abhd14b
23.62087	23.54038	NaN	F6TLV3;Q8BX90;E0CXY0	F6TLV3;Q8BX90	Fibronectin type-III domain-containing prote	Fndc3a
24.41054	24.34435	24.35111	F6UHR6;E9PXZ2;A0A0A6YXH3;A2A8Z1;	F6UHR6;E9PXZ2;A0A0A6Y	Oxysterol-binding protein	Osbpl9
NaN	23.33756	NaN	F7BPW6;E9QAT4;A2AIX1	F7BPW6;E9QAT4;A2AIX1	Protein transport protein sec16 (Fragment);	Sec16a
25.67303	25.09346	25.62366	F7CDT0;F6WMC0;P61082;G5E919	F7CDT0;F6WMC0;P61082;	NEDD8-conjugating enzyme Ubc12 (Fragmen	Ube2m
NaN	NaN	NaN	F8VQL0;P16056;D3YVY2;F6X333	F8VQL0;P16056	Hepatocyte growth factor receptor	Met
24.83846	24.82881	24.96279	F8WIE5;Q69ZR2	F8WIE5;Q69ZR2	E3 ubiquitin-protein ligase HECTD1	Hectd1
25.29614	25.2535	24.01052	H3BJJ6;H3BJD1;Q3TI69;H3BJQ3;H3BKE	H3BJJ6;H3BJD1;Q3TI69;H	DNA dC->dU-editing enzyme APOBEC-3 (Fra	Apobec3
24.60399	25.50749	25.18329	J3QQ30;J3QPW1;P53810;F8WGG5	J3QQ30;J3QPW1;P53810;	Phosphatidylinositol transfer protein alpha i	Pitpna
24.04427	24.42342	24.56964	O08915;D3YW40	O08915;D3YW40	AH receptor-interacting protein;AH receptor	Aip
24.74919	24.85757	24.2021	O35075	O35075	Down syndrome critical region protein 3 hor	Dscr3
22.63423	22.94771	22.64306	O35188;A0A1D5RMK8	O35188	Fractalkine	Cx3cl1
25.45513	25.72598	25.78706	O35215;G3UZN1;G3UYJ7	O35215;G3UZN1;G3UYJ7	D-dopachrome decarboxylase;Predicted gen	Ddt;Gm20441
NaN	NaN	NaN	O35640;Q921D0	O35640;Q921D0	Annexin A8;Annexin	Anxa8
NaN	NaN	NaN	O35857	O35857	Mitochondrial import inner membrane trans	Timm44
24.60964	NaN	24.11744	O35955	O35955	Proteasome subunit beta type-10	Psmb10
26.61807	26.71293	26.69975	O35988	O35988	Syndecan-4	Sdc4
26.16231	26.11347	25.87173	O54692	O54692	Centromere/kinetochore protein zw10 hom	Zw10
26.85043	26.87408	26.92025	O55135;A6PWZ2;D6RG53;D6RJJ3;B1AZ	O55135;A6PWZ2	Eukaryotic translation initiation factor 6;Euk	Eif6
24.92252	24.88577	24.92252	O70378;M0QWY0;M0QWP2	O70378;M0QWY0;M0QW	ER membrane protein complex subunit 8;ER	Emc8
29.30226	29.03223	29.31743	O70456	O70456	14-3-3 protein sigma	Sfn
24.78457	24.71816	24.63201	O88374;O35855;A0A1B0GX27;A0A1B0	O88374;O35855;A0A1B0	Branched-chain-amino-acid aminotransfera	Bcat2
27.91456	27.87408	27.90311	O88653;A0A0G2JGQ3	O88653;A0A0G2JGQ3	Ragulator complex protein LAMTOR3;Ragula	Lamtor3
26.81421	24.24627	24.1795	O88662	O88662	Epithelial membrane protein 2	Emp2
24.99766	24.80931	24.82396	O88696	O88696	ATP-dependent Clp protease proteolytic sub	Clpp
24.31008	NaN	NaN	O88792	O88792	Junctional adhesion molecule A	F11r
25.86703	26.33756	26.35783	O89023	O89023	Tripeptidyl-peptidase 1	Tpp1
25.28912	25.48616	25.34435	P00375	P00375	Dihydrofolate reductase	Dhfr
23.94056	24.16423	23.80931	P06795;A0A0G2JF49	P06795	Multidrug resistance protein 1B	Abcb1b
24.97596	25.47073	25.1946	P08074;F6YNX9	P08074;F6YNX9	Carbonyl reductase [NADPH] 2;Carbonyl red	Cbr2
25.59833	25.8044	25.75175	P10518	P10518	Delta-aminolevulinic acid dehydratase	Alad
24.52253	24.62645	24.33756	P11214	P11214	Tissue-type plasminogen activator	Plat
25.69179	25.37119	25.93607	P12032	P12032	Metalloproteinase inhibitor 1	Timp1
25.93382	26.01691	25.48616	P14576;E9PXC0;A0A1Y7VJJ0	P14576;E9PXC0	Signal recognition particle 54 kDa protein	Srp54;Srp54b
24.91342	25.06907	24.94949	P18406	P18406	Protein CYR61	Cyr61
26.40243	26.03171	26.20769	P19788	P19788	Matrix Gla protein	Mgp
25.61246	25.74663	25.64581	P21278	P21278	Guanine nucleotide-binding protein subunit	Gna11
25.38772	25.37782	25.79699	P22725;D3YTW9;A0A0R4J1M1;P24383;	P22725	Protein Wnt-5a	Wnt5a
26.17379	26.1332	26.34604	P26350;A0A087WP98;A0A087WPN6;A0	P26350	Prothymosin alpha	Ptma
27.5389	27.52402	27.42342	P26883;F6X9I3	P26883	Peptidyl-prolyl cis-trans isomerase FKBP1A	Fkbp1a
23.31008	22.10789	NaN	P28352;D3Z124;F6QA74	P28352;D3Z124;F6QA74	DNA-(apurinic or apyrimidinic site) lyase;DN	Apex1
23.83846	24.391	23.51653	P28867;Q1MX41;Q1MX43;Q1MX40;Q1	P28867;Q1MX41;Q1MX4	Protein kinase C delta type;Protein kinase C	Prkcd
23.28207	24.58692	23.70767	P35235	P35235	Tyrosine-protein phosphatase non-receptor	Ptpn11
26.69975	26.73892	26.80193	P40237;A2AIJ1	P40237;A2AIJ1	CD82 antigen;Tetraspanin (Fragment)	Cd82
23.88577	23.66492	23.14725	P41241;A0A1L1SR46;A0A1L1STA1;A0A1	P41241;A0A1L1SR46;A0A	Tyrosine-protein kinase CSK;Tyrosine-protei	Csk
26.63201	27.49381	27.27142	P48024;J3QN87;A0A1L1SSA3;Q9CXU9;J	P48024;J3QN87;A0A1L1S	Eukaryotic translation initiation factor 1;Euk	Eif1
NaN	NaN	NaN	P50228	P50228	C-X-C motif chemokine 5	Cxcl5
25.77706	25.417	25.68378	P51829;A0A1B0GT56	P51829;A0A1B0GT56	Adenylate cyclase type 7;Adenylate cyclase	Adcy7
24.12534	23.99335	24.10149	P51859;E0CXA0;E0CYW7	P51859;E0CXA0;E0CYW7	Hepatoma-derived growth factor;Hepatoma	Hdgf

24.33074	24.21697	24.35111	P54923	P54923	ADP-ribosylarginine] hydrolase	Adprh
26.08942	25.56964	25.77957	P57746	P57746	V-type proton ATPase subunit D	Atp6v1d
24.85281	24.83846	24.05258	P58389;B7ZDE0;A2AWF0;A2AWE9;F6Z6	P58389;B7ZDE0;A2AWF0;	Serine/threonine-protein phosphatase 2A ac	Ptpa
26.78955	26.54481	26.67303	P61021	P61021	Ras-related protein Rab-5B	Rab5b
27.18896	27.34096	27.26249	P61750;E9Q798;F6UFB9	P61750;E9Q798	ADP-ribosylation factor 4	Arf4
26.45199	26.4567	26.47537	P62320;A0A1W2P7K5	P62320;A0A1W2P7K5	Small nuclear ribonucleoprotein Sm D3;Sma	Snrpd3
27.18896	27.1795	27.2535	P62627;A2AVR9	P62627;A2AVR9	Dynein light chain roadblock-type 1;Dynein l	Dynlrb1
25.15267	25.43619	25.14492	P70202	P70202	Latexin	Lxn
25.74919	25.07316	25.78207	P70303	P70303	CTP synthase 2	Ctps2
25.48001	25.04009	24.93157	P70444	P70444	BH3-interacting domain death agonist	Bid
28.21234	28.20303	28.18896	P83882;A0A0A6YW33	P83882;A0A0A6YW33	60S ribosomal protein L36a;MCG18090	Rpl36a;Gm6525
NaN	22.52133	23.37782	P97742	P97742	Carnitine O-palmitoyltransferase 1, liver isof	Cpt1a
NaN	NaN	NaN	P97821;D3Z220	P97821	Dipeptidyl peptidase 1	Ctsc
24.67573	25.06907	24.90885	P97855	P97855	Ras GTPase-activating protein-binding prote	G3bp1
NaN	23.88577	23.96719	Q03963	Q03963	Interferon-induced, double-stranded RNA-ac	Eif2ak2
NaN	25.17188	24.28207	Q04447	Q04447	Creatine kinase B-type	Ckb
26.60399	26.68646	26.41054	Q06185;Q8BTB6	Q06185	ATP synthase subunit e, mitochondrial	Atp5i
23.63201	24.04427	24.43619	Q0KL02	Q0KL02	Triple functional domain protein	Trio
25.78955	26.21141	26.42021	Q31125	Q31125	Zinc transporter SLC39A7	Slc39a7
24.26785	23.79947	24.10949	Q3TCH7;E9PXT5;J3QJX0;E9PXY1;A2A43	Q3TCH7;E9PXT5;J3QJX0;E	Cullin-4A;Cullin-4B	Cul4a;Cul4b
22.44758	23.08699	NaN	Q3TET1;Q3UI47;B1AQZ2;P28741;F6V6Z	Q3TET1;Q3UI47;B1AQZ2;	Kinesin-like protein	Kif3a
27.43936	27.16039	26.93157	Q3THW5;P0C0S6;Q8R029;Q3UA95;G3U	Q3THW5;P0C0S6;Q8R029	Histone H2A.V;Histone H2A.Z;Histone H2A	H2afv;H2afz
25.63755	25.26069	25.44253	Q3TMN1;Q3UI33;O08663;M0QW69;M0	Q3TMN1;Q3UI33;O08663	Methionine aminopeptidase 2	Metap2
24.28912	NaN	NaN	Q3U741;Q501J6	Q3U741;Q501J6	DEAD (Asp-Glu-Ala-Asp) box polypeptide 17,	Ddx17
24.39754	24.07725	24.391	Q3U9G9;A0A0A6YXW3;A0A0A6YXT6;A0	Q3U9G9;A0A0A6YXW3;A0	Lamin-B receptor;Lamin-B receptor (Fragme	Lbr
24.48001	24.06907	23.92252	Q3UDC3;O88746;A0A1D5RM84;A0A1D	Q3UDC3;O88746;A0A1D5	Target of Myb protein 1;Target of Myb prote	Tom1
NaN	NaN	NaN	Q3UIR3	Q3UIR3	E3 ubiquitin-protein ligase DTX3L	Dtx3l
24.35783	24.48001	24.6811	Q3UTY6	Q3UTY6	Thrombospondin type-1 domain-containing	Thsd4
NaN	NaN	NaN	Q3V3R4	Q3V3R4	Integrin alpha-1	Itga1
24.96719	24.56384	25.04009	Q5SWD9	Q5SWD9	Pre-rRNA-processing protein TSR1 homolog	Tsr1
25.39428	25.09748	25.72858	Q61029;Q61033	Q61029;Q61033	Lamina-associated polypeptide 2, isoforms b	Tmpo
NaN	NaN	24.96279	Q61090	Q61090	Frizzled-7	Fzd7
NaN	NaN	23.95837	Q62181;E9Q0Z0	Q62181	Semaphorin-3C	Sema3c
25.64581	25.04009	25.57831	Q62419;A2ALV1;Q8BXU5;A2ALV3;Q624	Q62419	Endophilin-A2	Sh3gl1
25.18329	25.75175	25.30313	Q62422	Q62422	Osteoclast-stimulating factor 1	Ostf1
24.7543	24.68646	24.67573	Q6IRU5;F7BHJ0	Q6IRU5;F7BHJ0	Clathrin light chain B;Clathrin light chain (Fra	Cltb
NaN	NaN	NaN	Q6NS46	Q6NS46	Protein RRP5 homolog	Pdcd11
26.18329	25.29263	NaN	Q6P4T2	Q6P4T2	U5 small nuclear ribonucleoprotein 200 kDa	Snrnp200
NaN	NaN	NaN	Q6P9Q4	Q6P9Q4	FH1/FH2 domain-containing protein 1	Fhod1
24.83846	25.09346	25.24989	Q6PAR5;E9Q0D1;F7ADT6;F7ADQ2	Q6PAR5;E9Q0D1;F7ADT6;	GTPase-activating protein and VPS9 domain-	Gapvd1
NaN	23.51653	23.36453	Q6PB44	Q6PB44	Tyrosine-protein phosphatase non-receptor	Ptpn23
24.53445	24.49838	25.00196	Q6PGB6	Q6PGB6	N-alpha-acetyltransferase 50	Naa50
23.78955	24.1946	24.18707	Q6PGC1	Q6PGC1	ATP-dependent RNA helicase DHX29	Dhx29
NaN	NaN	NaN	Q6W5C0	Q6W5C0	C-X-C motif chemokine 3	Cxcl3
24.79452	24.7543	24.95394	Q6ZWQ5;Q3TGS7;Q3V2H3;O70493	Q6ZWQ5;Q3TGS7;Q3V2H	Sorting nexin 12, isoform CRA_c;Sorting nex	Snx12
27.13124	26.99766	27.08132	Q6ZWY8;CON__P21752;A0A0N4SVF0	Q6ZWY8	Thymosin beta-10	Tmsb10
27.58978	27.48616	27.5969	Q80SZ7;D3Z7Q3	Q80SZ7	Guanine nucleotide-binding protein G(I)/G(S	Gng5
26.86231	26.73892	26.98685	Q80UU9	Q80UU9	Membrane-associated progesterone recepto	Pgrmc2
24.64856	24.42342	24.68646	Q80WW9;B0R015	Q80WW9	DDRGK domain-containing protein 1	Ddrgk1
25.84566	25.97596	26.05672	Q80X71;D3Z191;D3Z0M2;D3Z6E0	Q80X71;D3Z191;D3Z0M2	Transmembrane protein 106B;Transmembra	Tmem106b
23.56384	23.71816	24.05258	Q80Y98;A0A1B0GSA5;A0A1B0GS27	Q80Y98;A0A1B0GSA5;A0A	Phospholipase DDHD2	Ddhd2
NaN	NaN	NaN	Q8BJ66	Q8BJ66	Kazal-type serine protease inhibitor domain-	Kazald1
23.391	22.90426	23.12218	Q8BJ71;A0A1D5RLQ0	Q8BJ71;A0A1D5RLQ0	Nuclear pore complex protein Nup93	Nup93
25.5285	25.33756	25.29964	Q8BJU2;D3YXN7;A0A0J9YTS4	Q8BJU2;D3YXN7;A0A0J9Y	Tetraspanin-9;Tetraspanin (Fragment)	Tspan9
25.29263	24.5285	25.20955	Q8BL97	Q8BL97	Serine/arginine-rich splicing factor 7	Srsf7
27.23535	27.10149	26.95394	Q8BQ46;F6QCI0	Q8BQ46;F6QCI0	TAF15 RNA polymerase II, TATA box binding	Taf15
24.7024	25.2021	24.82881	Q8BVU5;A2AH28;A2AH27;A2AH29	Q8BVU5;A2AH28;A2AH27	ADP-ribose pyrophosphatase, mitochondrial	Nudt9
25.5285	25.72078	25.73376	Q8C4F9;Q8CB78;O54965;A0A0G2JEM4;	Q8C4F9;Q8CB78;O54965;	E3 ubiquitin-protein ligase RNF13	Rnf13
25.72598	25.53445	25.59833	Q8C5N9;O88736	Q8C5N9;O88736	3-keto-steroid reductase	Hsd17b7
23.82881	23.78955	24.27498	Q8C788;Q91ZR2	Q8C788;Q91ZR2	Sorting nexin;Sorting nexin-18	Snx18
23.391	23.48001	23.5285	Q8CFI7	Q8CFI7	DNA-directed RNA polymerase II subunit RP	Polr2b
24.49838	23.29614	23.91342	Q8CH72;A2AGS0;A2AGS1	Q8CH72	E3 ubiquitin-protein ligase TRIM32	Trim32
24.39754	24.80931	NaN	Q8CJ53	Q8CJ53	Cdc42-interacting protein 4	Trip10
NaN	24.62087	24.35111	Q8JZN5;A0A0G2JDY4	Q8JZN5	Acyl-CoA dehydrogenase family member 9,	Acad9
23.83846	24.02749	23.45513	Q8JZQ2	Q8JZQ2	AFG3-like protein 2	Afg3l2
24.80931	24.67034	24.88577	Q8K1J6;A0A0N4SUW4;A0A0N4SV41	Q8K1J6;A0A0N4SUW4	CCA tRNA nucleotidyltransferase 1, mitocho	Trnt1
24.74919	24.64306	24.98468	Q8K268	Q8K268	ATP-binding cassette sub-family F member 3	Abcf3
23.63201	23.94949	23.66492	Q8R059	Q8R059	UDP-glucose 4-epimerase	Gale
23.86703	24.66492	24.84805	Q8R105	Q8R105	Vacuolar protein sorting-associated protein	Vps37c
25.54038	25.49838	25.30313	Q8R1G6;A0A087WPL1;F7C957;E9Q996	Q8R1G6;A0A087WPL1;F7	PDZ and LIM domain protein 2;PDZ and LIM	Pdlim2
25.32732	25.37782	25.32732	Q8R2K3;Q9CYR0	Q8R2K3;Q9CYR0	Single-stranded DNA binding protein 1;Singl	Ssbp1
24.49228	23.89504	25.00196	Q8R550;B0R0Y8;E9Q0C1	Q8R550;B0R0Y8;E9Q0C1	SH3 domain-containing kinase-binding prote	Sh3kbp1
23.49228	NaN	23.31008	Q8VCH6	Q8VCH6	Delta(24)-sterol reductase	Dhcr24
NaN	23.88577	NaN	Q8VEB4;A0A1D5RLZ5	Q8VEB4;A0A1D5RLZ5	Group XV phospholipase A2	Pla2g15
23.44253	23.46762	23.45513	Q8VH51;F7AA45;E9Q8F0;B7ZD61;B7ZD	Q8VH51;F7AA45;E9Q8F0;	RNA-binding protein 39;RNA-binding protein	Rbm39
NaN	NaN	NaN	Q91VJ2	Q91VJ2	Caveolae-associated protein 3	Cavin3
NaN	NaN	NaN	Q91VM5;A2AFI4;A2AFI3;Q9WV02	Q91VM5;A2AFI4;A2AFI3;Q	RNA binding motif protein, X-linked-like-1;R	Rbmxl1;Rbmx
25.22803	25.00624	25.60116	Q91W53;Q9D428	Q91W53	Golgin subfamily A member 7	Golga7
25.35111	NaN	25.21327	Q921W0	Q921W0	Charged multivesicular body protein 1a	Chmp1a
24.66492	24.71816	24.6811	Q921Y0;Q3UDM0;Q8BPB0	Q921Y0;Q3UDM0;Q8BPB0	MOB kinase activator 1A;MOB kinase activat	Mob1a;Mob1b
23.75939	23.85757	23.36453	Q922H4;D3Z3L0;D3Z3M0;D3Z2L8;D3YV	Q922H4	Mannose-1-phosphate guanyltransferase alp	Gmppa
25.10949	25.06497	24.47383	Q922Y1	Q922Y1	UBX domain-containing protein 1	Ubxn1
26.5285	26.56384	26.60399	Q99JB2;A2AG41;A2AG39;F6WI02	Q99JB2	Stomatin-like protein 2, mitochondrial	Stoml2
24.49228	24.8811	24.35111	Q99KE1	Q99KE1	NAD-dependent malic enzyme, mitochondri	Me2
24.1946	24.84805	24.50446	Q99KR3	Q99KR3	Endoribonuclease LACTB2	Lactb2
23.56384	23.33756	23.84805	Q99L13	Q99L13	3-hydroxyisobutyrate dehydrogenase, mitoc	Hibadh
24.42982	24.64306	24.64306	Q99L27;Q9DCZ1	Q99L27	GMP reductase 2	Gmpr2
23.29614	23.68646	23.79947	Q99LM2;D6RJ46;F2Z486	Q99LM2	CDK5 regulatory subunit-associated protein	Cdk5rap3

25.81421	25.85757	25.391	Q99M71;A0A1Y7VKS9	Q99M71	Mammalian ependymin-related protein 1	Epdr1
24.91798	25.10949	24.68646	Q99PG2	Q99PG2	Opioid growth factor receptor	Ogfr
25.04843	24.20955	24.68646	Q9CPP0	Q9CPP0	Nucleoplasmin-3	Npm3
27.05051	27.18896	26.68646	Q9CPQ1;D3Z6E1;A0A0G2JDA6	Q9CPQ1;D3Z6E1	Cytochrome c oxidase subunit 6C;Cytochrom	Cox6c
26.35951	26.51352	26.17188	Q9CPQ3	Q9CPQ3	Mitochondrial import receptor subunit TOM	Tomm22
26.45042	26.33756	26.57542	Q9CPQ8	Q9CPQ8	ATP synthase subunit g, mitochondrial	Atp5l
27.34942	27.509	27.54628	Q9CPT4	Q9CPT4	Myeloid-derived growth factor	Mydgf
28.29789	28.5389	28.41941	Q9CPW4;Q3UA72	Q9CPW4;Q3UA72	Actin-related protein 2/3 complex subunit 5	Arpc5
26.39591	26.26428	25.96279	Q9CQ43;Q8VCG1	Q9CQ43;Q8VCG1	Deoxyuridine triphosphatase	Dut
25.43936	24.46139	24.94949	Q9CQ45	Q9CQ45	Neudesin	Nenf
27.3662	26.83846	27.67976	Q9CQC9	Q9CQC9	GTP-binding protein SAR1b	Sar1b
24.8044	25.32045	24.73892	Q9CQD4;Q99LU0	Q9CQD4;Q99LU0	Charged multivesicular body protein 1b-2;Ch	Chmp1b2;Chmp
25.43936	25.34435	25.74407	Q9CQE8	Q9CQE8	RNA transcription, translation and transport	RTRAF
26.13907	26.05258	25.89273	Q9CQI6;A0A1D5RLP1	Q9CQI6;A0A1D5RLP1	Coactosin-like protein	Cotl1
25.59548	25.56384	25.63478	Q9CQM5	Q9CQM5	Thioredoxin domain-containing protein 17	Txndc17
29.0427	28.83846	28.93438	Q9CQV6;M0QWC2	Q9CQV6;M0QWC2	Microtubule-associated proteins 1A/1B light	Map1lc3b
23.45513	23.31008	23.417	Q9CR00;A0A0G2JGN6	Q9CR00;A0A0G2JGN6	26S proteasome non-ATPase regulatory sub	Psmd9
26.00838	26.57542	26.33586	Q9CRA4;D3Z458	Q9CRA4	Methylsterol monooxygenase 1	Msmo1
24.71816	24.65403	24.70767	Q9CRB9;S4R238;F6QFL0;Q9D9P1;D3Z0L	Q9CRB9;S4R238;F6QFL0;Q	MICOS complex subunit Mic19;MICOS comp	Chchd3
23.5285	NaN	22.55684	Q9CXP8;G3UZK1	Q9CXP8;G3UZK1	Guanine nucleotide-binding protein G(I)/G(S	Gng10;Gm2050
24.63201	23.84805	23.87643	Q9CXT8	Q9CXT8	Mitochondrial-processing peptidase subunit	Pmpcb
27.66628	27.61105	27.74535	Q9CY50;A0A286YDB7;A0A286YCT4;A0A	Q9CY50;A0A286YDB7;A0A	Translocon-associated protein subunit alpha	Ssr1
24.53445	24.85281	24.7543	Q9CYA0	Q9CYA0	Cysteine-rich with EGF-like domain protein 2	Creld2
25.1946	24.88577	25.03171	Q9CZQ6;Q9JI58;O08604;O08603;O0860	Q9CZQ6	Retinoic acid early-inducible protein 1-epsilo	Raet1e
25.07316	24.80931	24.87643	Q9D172;A0A1W2P870;A0A1W2P7B6	Q9D172;A0A1W2P870	ES1 protein homolog, mitochondrial;ES1 pro	D10Jhu81e
26.13907	26.15074	26.038	Q9D1A2	Q9D1A2	Cytosolic non-specific dipeptidase	Cndp2
25.36786	25.64306	25.93607	Q9D358;A0A1W2P7X3;Q561M1	Q9D358;A0A1W2P7X3;Q5	Low molecular weight phosphotyrosine prot	Acp1
24.17188	24.1332	24.74407	Q9D4H8;E0CYT5	Q9D4H8;E0CYT5	Cullin-2;Cullin-2 (Fragment)	Cul2
NaN	24.35111	NaN	Q9D4I9;P35288	Q9D4I9;P35288	RAB23, member RAS oncogene family, isofo	Rab23
25.44253	25.55801	25.59833	Q9D7H3;D3Z263;D6REI7;D6RD00	Q9D7H3;D3Z263	RNA 3-terminal phosphate cyclase;RNA 3-te	RtcA;Rtca
NaN	23.88577	24.10949	Q9D7J6	Q9D7J6	Deoxyribonuclease-1-like 1	Dnase1l1
25.35783	24.55215	25.67842	Q9D880	Q9D880	Mitochondrial import inner membrane trans	Timm50
26.18139	26.26428	26.24445	Q9D8V0;A3KGR9;Q6PGJ8	Q9D8V0;A3KGR9	Minor histocompatibility antigen H13;Histoc	Hm13;H13
NaN	NaN	24.09346	Q9DAR7;Q3TBW9	Q9DAR7;Q3TBW9	m7GpppX diphosphatase	Dcps
23.57542	23.51653	23.36453	Q9DAU1	Q9DAU1	Protein canopy homolog 3	Cnpy3
NaN	NaN	NaN	Q9DAU7;A2A5G4;A2A5G5;A2A5G6	Q9DAU7;A2A5G4;A2A5G5	WAP four-disulfide core domain protein 2	Wfdc2
NaN	NaN	NaN	Q9DAV6	Q9DAV6	R86	Serpinb9b
25.59833	25.04427	25.4073	Q9DB15	Q9DB15	39S ribosomal protein L12, mitochondrial	Mrpl12
25.239	25.32389	25.1055	Q9DB34;A0A1B0GT75;A0A1B0GR63;A0/	Q9DB34;A0A1B0GT75;A0/	Charged multivesicular body protein 2a	Chmp2a
24.64306	24.21697	23.79947	Q9DBE8	Q9DBE8	Alpha-1,3/1,6-mannosyltransferase ALG2	Alg2
26.92025	26.42342	26.83846	Q9DBG5	Q9DBG5	Perilipin-3	Plin3
25.65949	25.58978	25.79699	Q9DCC4	Q9DCC4	Pyrroline-5-carboxylate reductase 3	Pycr3
24.03591	23.83846	23.81909	Q9DCC5;P23198;D3Z1A9;D3Z313;Q7TPI	Q9DCC5;P23198	Cbx3 protein;Chromobox protein homolog 3	Cbx3
28.11148	28.16997	28.20769	Q9DCK3;D3Z0B7;D3Z0T3	Q9DCK3;D3Z0B7;D3Z0T3	Tetraspanin-4;Tetraspanin (Fragment)	Tspan4
25.27853	25.32732	25.27142	Q9DCM0	Q9DCM0	Persulfide dioxygenase ETHE1, mitochondria	Ethe1
24.38443	24.28207	24.17188	Q9EQQ9	Q9EQQ9	Protein O-GlcNAcase	Mgea5
24.71816	25.73635	24.7543	Q9ER39	Q9ER39	Torsin-1A	Tor1a
25.73892	25.81421	25.51653	Q9ER41;F6VK94;D6REF0;F6ZDX6	Q9ER41;F6VK94;D6REF0	Torsin-1B;Torsin-1B (Fragment)	Tor1b
25.81176	26.04843	25.90885	Q9ERG0	Q9ERG0	LIM domain and actin-binding protein 1	Lima1
30.23078	30.13614	30.06085	Q9ES97	Q9ES97	Reticulon-3	Rtn3
NaN	NaN	NaN	Q9ESM6	Q9ESM6	Glycerophosphoinositol inositolphosphodies	Gdpd2
25.58118	25.75175	25.28912	Q9EST5	Q9EST5	Acidic leucine-rich nuclear phosphoprotein 3	Anp32b
25.97158	25.92025	25.72858	Q9ET30	Q9ET30	Transmembrane 9 superfamily member 3	Tm9sf3
28.60399	28.62157	28.41135	Q9JJI8	Q9JJI8	60S ribosomal protein L38	Rpl38
25.99981	26.24445	26.14686	Q9JJU8	Q9JJU8	SH3 domain-binding glutamic acid-rich-like p	Sh3bgrl
24.35111	24.44253	24.02749	Q9JKY0	Q9JKY0	CCR4-NOT transcription complex subunit 9	Cnot9
NaN	25.1488	NaN	Q9QZ85;Q3UED7	Q9QZ85	Interferon-inducible GTPase 1	Iigp1
23.68646	22.0426	23.88577	Q9WTX6;Q3TPM3;A0A0N4SUW1;D3Z2H	Q9WTX6;Q3TPM3	Cullin-1;Cullin-1 (Fragment)	Cul1
NaN	NaN	NaN	Q9WTY4	Q9WTY4	Aquaporin-5	Aqp5
24.08537	23.80931	NaN	Q9Z0E6;E9Q2N7	Q9Z0E6	Guanylate-binding protein 2	Gbp2
23.81909	23.80931	24.15653	Q9Z0P5;A0A1L1SU53;A0A1L1STC8;A0A0	Q9Z0P5;A0A1L1SU53	Twinfilin-2;Twinfilin-2 (Fragment)	Twf2
24.37119	24.00196	24.06085	Q9Z0R9	Q9Z0R9	Fatty acid desaturase 2	Fads2
23.56384	23.21401	23.23754	Q9Z1F9;H3BLM2;H3BLR3	Q9Z1F9	SUMO-activating enzyme subunit 2	Uba2
24.1332	23.75939	24.29614	Q9Z2X2	Q9Z2X2	26S proteasome non-ATPase regulatory sub	Psmd10
24.66492	24.53445	NaN	S4R2T5;Q9D906;S4R2P7;S4R2D5;A0A0A	S4R2T5;Q9D906;S4R2P7;S	Ubiquitin-like modifier-activating enzyme AT	Atg7
23.0592	23.40406	NaN	A0A023T778;Q9CQL1;P61327;G3UZW7	A0A023T778;Q9CQL1;P61	Mago nashi protein;Protein mago nashi hom	Magohb;Magoh
NaN	NaN	NaN	A0A087WQT6;O89110	A0A087WQT6;O89110	Caspase-8	Casp8
24.64306	24.91342	25.24627	A0A087WR20;P49935;D3Z437	A0A087WR20;P49935	Pro-cathepsin H (Fragment);Pro-cathepsin H	Ctsh
25.13712	24.91798	24.80931	A0A087WRP7;A0A087WNZ5;P0CW02;P	A0A087WRP7;A0A087WN	Lymphocyte antigen 6C1 (Fragment);Lymph	Ly6c1;
23.6971	23.86703	24.09346	A0A0A0MQB0;Q4PJX1	A0A0A0MQB0;Q4PJX1	cDNA sequence BC003331;Protein odr-4 hom	BC003331;Odr4
22.71607	22.43746	22.41183	A0A0A6YXY1;A0A0A6YX97;A2APZ3;Q8R	A0A0A6YXY1;A0A0A6YX97	N-terminal Xaa-Pro-Lys N-methyltransferase	Ntmt1
NaN	NaN	NaN	A0A0B4J1E4;Q5U431	A0A0B4J1E4;Q5U431	G-protein-coupled receptor 39;G-protein co	Gpr39
22.58921	NaN	NaN	A0A0G2JG00;Q3TUE1;A0A0G2JGW9;A0	A0A0G2JG00;Q3TUE1;A0A	Far upstream element-binding protein 1	Fubp1
22.80735	22.75939	23.09989	A0A0H2UH17;A0A0G2JDV6;Q80X50;A0/	A0A0H2UH17;A0A0G2JDV	Ubiquitin associated protein 2-like, isoform (	Ubap2l
24.28912	23.93157	23.24336	A0A0N4SVQ1;Q62425	A0A0N4SVQ1;Q62425	Cytochrome c oxidase subunit NDUFA4	Ndufa4
23.81909	NaN	23.33756	A0A0R4J078;Q8VCH8;A0A087WSK5	A0A0R4J078;Q8VCH8	UBX domain containing 2, isoform CRA_a;UE	Ubxn4
23.417	24.1795	24.01903	A0A0R4J0G6;Q9CYK2	A0A0R4J0G6;Q9CYK2	Glutaminyl-peptide cyclotransferase	Qpct
24.22435	24.73376	24.80931	A0A0R4J0K1;Q9ERR7;A0A0G2JEF3;A0A1	A0A0R4J0K1;Q9ERR7;A0A	Selenoprotein F;Selenoprotein F (Fragment)	Selenof
25.26428	25.1757	25.2571	A0A0R4J0K4;Q9CRG1	A0A0R4J0K4;Q9CRG1	Transmembrane 7 superfamily member 3	Tm7sf3
23.67573	23.80931	23.93157	A0A0R4J150;B9EJ86	A0A0R4J150;B9EJ86	Oxysterol-binding protein;Oxysterol-binding	Osbpl8
25.75939	25.54922	26.20955	A0A0R4J1V1;Q75N73;D3Z6P5;D3Z354;D	A0A0R4J1V1;Q75N73;D3Z	Solute carrier family 39 (Zinc transporter), m	Slc39a14
20.43746	NaN	NaN	A0A0R4J268;G3X8V6;O70458	A0A0R4J268;G3X8V6;O70	Oncostatin-M-specific receptor subunit beta	Osmr
25.49228	25.391	25.52253	A0A0U1RP13;Q8VDP6;A0A0U1RP60;A0/	A0A0U1RP13;Q8VDP6	CDP-diacylglycerol--inositol 3-phosphatidylt	Cdipt
24.12534	24.16423	24.11744	A0A0U1RPF2;Q99P31	A0A0U1RPF2;Q99P31	Hsp70-binding protein 1 (Fragment);Hsp70-b	Hspbp1
24.16423	24.10949	24.71816	A0A1B0GRP7;Q9Z2Y8;A0A1B0GQY5;A0/	A0A1B0GRP7;Q9Z2Y8;A0A	Pyridoxal phosphate-binding protein (Fragm	Plpbp
NaN	NaN	NaN	A0A1D5RLS2;A0A1D5RM23;Q9CQF3;A0	A0A1D5RLS2;A0A1D5RM2	Cleavage and polyadenylation-specificity fac	Nudt21
NaN	NaN	NaN	A0A1D5RM88;A0A1D5RLE0;E9QMR2;A0	A0A1D5RM88;A0A1D5RLE	Dedicator of cytokinesis protein 9 (Fragment	Dock9
24.239	24.48001	NaN	A0A1L1SRG5;P23927	A0A1L1SRG5;P23927	Alpha-crystallin B chain (Fragment);Alpha-cr	Cryab

26.69975	26.43619	26.85043	A0A1L1SVK0;Q61206;A0A1L1SQ76;A0A	A0A1L1SVK0;Q61206;A0A	Platelet-activating factor acetylhydrolase IB	Pafah1b2
28.36202	28.14589	28.44727	A0A1W2P7X0;E9QMV2;Q4KML4	A0A1W2P7X0;E9QMV2;Q	Costars family protein ABRACL (Fragment);C	Abracl
24.62645	24.76951	24.2535	A0A1Y7VJ48;P48428	A0A1Y7VJ48;P48428	Tubulin-specific chaperone A	Tbca
24.70767	24.84326	24.66492	A0A1Y7VJW9;Q9WVH9	A0A1Y7VJW9;Q9WVH9	Fibulin-5	Fbln5
24.2535	24.46139	24.54038	A2AEX7;A2AEY1;A2AEX8;A2AEX6;A2AEY	A2AEX7;A2AEY1;A2AEX8;A	Four and a half LIM domains protein 1;Four	Fhl1
25.19835	24.72858	24.42982	A2AFP5;Q9R0M6;A2AFP4	A2AFP5;Q9R0M6	RAB9, member RAS oncogene family (Fragm	Rab9;Rab9a
23.5285	23.88577	23.33756	A2AHD1;Q9Z0G4	A2AHD1;Q9Z0G4	WNT1 inducible signaling pathway protein 2	Wisp2
25.74663	25.32389	25.60964	A2AUE1;G5E8T0;P60904	A2AUE1;G5E8T0;P60904	DnaJ homolog subfamily C member 5 (Fragm	Dnajc5
24.63201	24.5285	24.65949	A2CG44;E9Q3M0;Q8BUN5;Q62432;F7B	A2CG44;E9Q3M0;Q8BUN	Mothers against decapentaplegic homolog (	Smad3;Smad2
23.44253	23.67573	23.96719	B1ATZ0;B1ATZ1;Q3UMA3;Q99LI8;B1AT	B1ATZ0;B1ATZ1;Q3UMA3	Hepatocyte growth factor-regulated tyrosine	Hgs
NaN	NaN	NaN	B2RY04	B2RY04	Dedicator of cytokinesis protein 5	Dock5
24.32389	24.38443	23.70767	B7ZC19;Q8BXQ2	B7ZC19;Q8BXQ2	GPI transamidase component PIG-T	Pigt
NaN	22.70134	22.24481	B9EJ54;A0A0J9YUD5	B9EJ54;A0A0J9YUD5	MCG21756, isoform CRA_b;Nucleoporin 205	Nup205
NaN	21.47754	22.33211	C0HKG6;C0HKG5	C0HKG6;C0HKG5	Ribonuclease T2-B;Ribonuclease T2-A	Rnaset2b;Rnase
25.14492	25.36119	25.28207	D2DFA9;Q923Z0	D2DFA9;Q923Z0	G protein-coupled receptor family C group 5	Gprc5b
NaN	23.50446	23.73892	D3YUE7;Q8CB44;D6RET7	D3YUE7;Q8CB44;D6RET7	GRAM domain-containing protein 4	Gramd4
24.26069	23.79947	24.66492	D3YXG2;Q9D997;Q9QZ08	D3YXG2;Q9D997;Q9QZ08	N-acetyl-D-glucosamine kinase	Nagk
24.57542	23.81909	24.1332	D3YXT0;Q91WD5	D3YXT0;Q91WD5	NADH dehydrogenase [ubiquinone] iron-sulf	Ndufs2
27.30661	26.63201	27.2535	D3Z125;E9PUA7;D3Z7X7;F8WHQ1;Q62	D3Z125;E9PUA7;D3Z7X7;F	Tumor protein D52 (Fragment);Tumor prote	Tpd52
24.48001	23.92252	24.46139	D3Z375;Q62048	D3Z375;Q62048	Phosphoprotein-enriched in astrocytes 15A	Pea15a;Pea15
NaN	23.68646	23.35111	D3Z4D8;Z4YJY0;Q3TCJ1;D6RJ39	D3Z4D8;Z4YJY0;Q3TCJ1	BRISC complex subunit Abraxas 2 (Fragment	Abraxas2
25.53445	25.1757	25.47383	D3Z5U5;Q9Z257;Q9JJX6;D3YYR5;Q9Z25	D3Z5U5;Q9Z257;Q9JJX6;D	P2X purinoceptor;P2X purinoceptor 4	P2rx4
25.06085	25.32045	26.54628	D3Z7A7;Q6ZPF4	D3Z7A7;Q6ZPF4	Formin-like protein 3	Fmnl3
NaN	NaN	NaN	D3Z7D5;Q02788	D3Z7D5;Q02788	Collagen alpha-2(VI) chain	Col6a2
24.72858	24.36453	24.35783	D3Z7S0;D3Z4J5;Q9D1H7	D3Z7S0;D3Z4J5;Q9D1H7	Golgi to ER traffic protein 4 homolog (Fragm	Get4
24.61526	24.29614	23.49228	D6RDC7;Q9ERF3;D6RJ72;Q8BVQ0	D6RDC7;Q9ERF3;D6RJ72;	WD repeat-containing protein 61;WD repeat	Wdr61
27.52402	27.41538	27.58978	D6RFU9	D6RFU9	Synaptophysin-like protein	Sypl
25.54038	25.70504	24.94949	E0CY07;Q9CR64	E0CY07;Q9CR64	Protein kish;Protein kish-A	Tmem167;Tmer
23.18556	23.00539	22.8783	E0CYV2;F6TLH3;E0CXK8;E9PX53;E9QPR	E0CYV2;F6TLH3;E0CXK8;E	Serine/threonine-protein phosphatase 4 reg	Ppp4r1
24.02749	24.20955	24.11744	E0CZH6;Q922Q1	E0CZH6;Q922Q1	Mitochondrial amidoxime reducing compone	Marc2
26.67303	25.95394	25.95615	E9PUM4;A0A1L1SQ51;Q71LX4;Q8CDM	E9PUM4;A0A1L1SQ51;Q7	Talin-2	Tln2;T
NaN	NaN	NaN	E9PVM7;P48774;E9Q024;E9Q5L9;E9PV	E9PVM7;P48774;E9Q024	Glutathione S-transferase (Fragment);Glutat	Gstm5
NaN	23.51653	23.63201	E9PVP1;A0A0G2JG52	E9PVP1;A0A0G2JG52	Crystallin beta-gamma domain-containing 1	Crybg1
NaN	NaN	23.65403	E9PWK7;Q8C8K1;P54761;Q6PFV6;E9PU	E9PWK7;Q8C8K1;P54761	Ephrin type-B receptor 4	Ephb4
NaN	NaN	NaN	E9PX63;O19441	E9PX63;O19441	Histocompatibility 2, Q region locus 1	H2-Q1
24.35783	24.46139	25.07316	E9PYN1;Q8R5M8;E0CY16	E9PYN1;Q8R5M8;E0CY16	Cell adhesion molecule 1	Cadm1
NaN	22.1488	22.23023	E9Q0A7;Q4KMM3;B1H3M0	E9Q0A7;Q4KMM3	Oxidation resistance protein 1	Oxr1
NaN	NaN	NaN	E9Q109;Q9CXK8	E9Q109;Q9CXK8	60S ribosome subunit biogenesis protein NIP	Nip7
NaN	23.08861	22.91889	E9Q3G7;P98197;F6Z4J2;D6RII8	E9Q3G7;P98197;F6Z4J2	Phospholipid-transporting ATPase;Probable	Atp11a
24.2535	24.1332	24.97158	E9Q4K3;Q8CBC8;P24288;B2KFA9;B2KFA	E9Q4K3;Q8CBC8;P24288;	Branched-chain-amino-acid aminotransferas	Bcat1
25.09346	25.42342	25.14102	E9Q5H2;E9PZF5;P97822	E9Q5H2;E9PZF5;P97822	Acidic leucine-rich nuclear phosphoprotein 3	Anp32e
25.07316	25.09346	24.94503	E9QAQ5;Q9WV60	E9QAQ5;Q9WV60	Glycogen synthase kinase-3 beta	Gsk3b
26.04635	25.61246	26.21327	E9QQ10;Q70FJ1	E9QQ10;Q70FJ1	A-kinase anchor protein 9	Akap9
26.02327	25.55508	25.58692	F6UFG6;D3YYE1;D3Z7M9;O35381;Q64	F6UFG6;D3YYE1;D3Z7M9;	Acidic leucine-rich nuclear phosphoprotein 3	Anp32a
23.68646	23.67573	23.15499	F6UV57;F6YCM8;F8WIH0;D3Z7V3;H7BX	F6UV57;F6YCM8;F8WIH0;	Sarcolemmal membrane-associated protein	Slmap
25.239	25.49228	25.57831	F6V6T4;Q9R0Q3;Q8BPI2	F6V6T4;Q9R0Q3;Q8BPI2	Transmembrane emp24 domain-containing	Tmed2
24.11744	24.30313	23.94056	F6WV69;F7BIK4;Q80XR8;J3QK07;D3Z1I	F6WV69;F7BIK4;Q80XR8;	ARF GTPase-activating protein GIT2 (Fragme	Git2
24.71816	25.08942	25.37451	F6ZFT1;F8WJ64;Q9CR21	F6ZFT1;F8WJ64;Q9CR21	Acyl carrier protein (Fragment);Acyl carrier	Ndufab1
23.50446	23.07398	23.56384	F7AAE0;Q8K2J0	F7AAE0;Q8K2J0	Phosphoinositide phospholipase C (Fragmen	Plcd3
NaN	NaN	24.32389	F7BTP8;Q9QYI3	F7BTP8;Q9QYI3	DnaJ homolog subfamily C member 7 (Fragm	Dnajc7
22.63644	22.62756	22.63201	F7C528;E9QAS4;E9QAS5;E9Q614;B1AR	F7C528;E9QAS4;E9QAS5;	Chromodomain helicase DNA-binding protei	Chd3;Chd4
24.95394	25.42982	24.86231	F7CVJ5	F7CVJ5	AHNAK nucleoprotein 2 (Fragment)	Ahnak2
NaN	NaN	NaN	F7CVZ7;E9Q9N6;E9PYX3;A0A0R4J227;A	F7CVZ7;E9Q9N6;E9PYX3;A	Serine/threonine-protein kinase MARK2 (Fra	Mark2
25.00196	25.01478	24.58692	F7D432;E9PWE0;E0CYV0;P23506;F6V9F	F7D432;E9PWE0;E0CYV0;	Protein-L-isoaspartate O-methyltransferase	Pcmt1
NaN	24.09346	23.87643	F8VQD7;Q05909;E9Q6E7	F8VQD7;Q05909;E9Q6E7	Receptor-type tyrosine-protein phosphatase	Ptprg
22.73892	23.02242	23.19009	F8WH10;E9Q4N8;F8WH81;A0A1L1SVG	F8WH10;E9Q4N8;F8WH8	Vascular endothelial growth factor A	Vegfa
NaN	NaN	22.72025	F8WH20;D3Z194;D3YTT6;Q9DBS9;A0A	F8WH20;D3Z194;D3YTT6;	Oxysterol-binding protein	Osbpl3
25.391	25.36119	25.1055	F8WHW3;Q3TWL2;E0CYE6;E0CZF6;E0C	F8WHW3;Q3TWL2;E0CYE	Transmembrane protein 55b;Type 1 phosph	Tmem55b;Pip4
NaN	NaN	NaN	F8WIK5;Q9ERS5	F8WIK5;Q9ERS5	Pleckstrin homology domain-containing fam	Plekha2
NaN	NaN	NaN	F8WJG3;P62996	F8WJG3;P62996	Transformer-2 protein homolog beta	Tra2b
24.16423	24.51653	24.68646	G3UXT7;Q8CFQ9;P56959;G3UZD2;Q91	G3UXT7;Q8CFQ9;P56959	RNA-binding protein FUS (Fragment);Fusion,	Fus
25.00624	25.14492	25.32389	G3X9J4;E9QM90;Q148V7;A0A087WRM	G3X9J4;E9QM90;Q148V7;	RIKEN cDNA 2310035C23 gene	2310035C23Rik
NaN	NaN	22.16116	G5E8J3;Q8K1X1;A0A0U1RQ40	G5E8J3;Q8K1X1	Bromodomain and WD repeat domain conta	Wdr11
25.1795	23.97596	24.39754	G5E8Y6;E9QP49;Q99MS7	G5E8Y6;E9QP49;Q99MS7	EH domain-binding protein 1-like protein 1	Ehbp1l1
22.81323	23.40406	22.95482	H7BXB1;E9Q4G7;E9PWB2;Q8BK63;E9Q	H7BXB1;E9Q4G7;E9PWB2	Casein kinase I isoform alpha	Csnk1a1;C
23.51653	23.02918	23.72858	J3QN06;J3QMM7;K3W4M4;Q9CZ42	J3QN06;J3QMM7;K3W4M	ATP-dependent (S)-NAD(P)H-hydrate dehyd	Naxd
24.32389	24.64856	24.79947	O08688	O08688	Calpain-5	Capn5
23.97596	23.90426	23.07398	O08738	O08738	Caspase-6	Casp6
26.60399	26.58978	26.57542	O08997	O08997	Copper transport protein ATOX1	Atox1
24.7543	25.01478	26.89735	O09005	O09005	Sphingolipid delta(4)-desaturase DES1	Degs1
24.15653	25.4073	25.46762	O35316;A0A0U1RQA5;A0A0U1RP20;A0	O35316;A0A0U1RQA5;A0	Sodium- and chloride-dependent taurine tra	Slc6a6
25.15653	24.60964	24.11744	O54950	O54950	5-AMP-activated protein kinase subunit gam	Prkag1
NaN	NaN	NaN	O55091	O55091	Protein IMPACT	Impact
24.01903	24.78955	24.26785	O55242	O55242	Sigma non-opioid intracellular receptor 1	Sigmar1
23.28207	23.417	22.20658	O70152;F8WII3;A2BDX2	O70152;F8WII3;A2BDX2	Dolichol-phosphate mannosyltransferase su	Dpm1
24.09346	24.28207	24.74919	O70433	O70433	Four and a half LIM domains protein 2	Fhl2
26.02749	25.96939	25.98902	O70591;F8WJ30	O70591	Prefoldin subunit 2	Pfdn2
23.26785	NaN	NaN	O88200	O88200	C-type lectin domain family 11 member A	Clec11a
NaN	NaN	24.22435	O88413	O88413	Tubby-related protein 3	Tulp3
NaN	NaN	NaN	O88477	O88477	Insulin-like growth factor 2 mRNA-binding p	Igf2bp1
23.33756	NaN	NaN	O88668;K4DI63;J3QP41	O88668;K4DI63	Protein CREG1;Cellular repressor of E1A-stin	Creg1
23.07398	22.96543	NaN	P07742	P07742	Ribonucleoside-diphosphate reductase large	Rrm1
NaN	NaN	NaN	P11031	P11031	Activated RNA polymerase II transcriptional	Sub1
NaN	NaN	NaN	P13745;Q6P8Q0;D3Z6A6;D3YZV3;P1064	P13745;Q6P8Q0;D3Z6A6;	Glutathione S-transferase A1;Glutathione S-	Gsta1;Gsta
NaN	23.32389	22.94949	P14094;A0A0A6YX05	P14094;A0A0A6YX05	Sodium/potassium-transporting ATPase sub	Atp1b1
28.65267	28.78332	28.77077	P15532;Q5NC80;Q5NC79	P15532;Q5NC80;Q5NC79	Nucleoside diphosphate kinase A;Nucleoside	Nme1
26.0296	25.60116	25.24264	P17095	P17095	High mobility group protein HMG-I/HMG-Y	Hmga1
26.78955	26.75175	26.65949	P20065	P20065	Thymosin beta-4	Tmsb4x

24.18707	24.31008	24.59833	P25322	P25322	G1/S-specific cyclin-D1	Ccnd1
23.5285	24.44253	23.54038	P27790	P27790	Major centromere autoantigen B	Cenpb
23.95837	23.84805	23.46762	P33174	P33174	Chromosome-associated kinesin KIF4	Kif4
23.90426	23.89504	24.35783	P35585	P35585	AP-1 complex subunit mu-1	Ap1m1
30.6372	30.65438	30.59335	P35762;A0A140LJL0	P35762;A0A140LJL0	CD81 antigen;CD81 antigen (Fragment)	Cd81
NaN	NaN	NaN	P36552	P36552	Oxygen-dependent coproporphyrinogen-III	Cpox
NaN	NaN	NaN	P39688;D3YZ57	P39688;D3YZ57	Tyrosine-protein kinase Fyn;Tyrosine-protei	Fyn
24.36453	24.239	23.57542	P49769;A0A0R4J1F2;Q3U4P5;Q61144	P49769;A0A0R4J1F2;Q3U	Presenilin-1;Presenilin;Presenilin-2	Psen1;Psen2
26.02538	26.0874	26.00624	P49962;D3YZX8	P49962	Signal recognition particle 9 kDa protein	Srp9
26.78955	27.00838	26.72598	P50637;A0A140LIU9	P50637	Translocator protein	Tspo
26.78955	26.57542	26.71293	P51807	P51807	Dynein light chain Tctex-type 1	Dynlt1
22.96895	23.15808	23.68646	P52479	P52479	Ubiquitin carboxyl-terminal hydrolase 10	Usp10
23.94056	23.65403	24.42342	P52633;D3YU28	P52633	Signal transducer and transcription activator	Stat6
25.41377	25.46451	25.73635	P52927;Q6NSP9	P52927;Q6NSP9	High mobility group protein HMGI-C	Hmga2
26.90885	26.81421	26.82639	P56382	P56382	ATP synthase subunit epsilon, mitochondrial	Atp5e
NaN	25.00196	24.75939	P56389	P56389	Cytidine deaminase	Cda
25.60116	25.53445	25.46762	P56391;A0A140LIU3	P56391;A0A140LIU3	Cytochrome c oxidase subunit 6B1	Cox6b1
25.63755	25.59833	25.75175	P56812;D3Z7Q5	P56812;D3Z7Q5	Programmed cell death protein 5	Pdcd5
24.6811	24.28207	24.04427	P61028	P61028	Ras-related protein Rab-8B	Rab8b
25.80931	25.94726	25.90656	P61804	P61804	Dolichyl-diphosphooligosaccharide--protein	Dad1
24.88577	24.98033	24.94503	P61924	P61924	Coatomer subunit zeta-1	Copz1
22.08213	22.49473	22.59149	P61967;D3Z268;D3Z0D6;D3YXN0	P61967;D3Z268;D3Z0D6;D	AP-1 complex subunit sigma-1A;AP-1 comple	Ap1s1
27.26249	27.33245	27.50142	P62075;A0A1W2P756;A0A1W2P7H2	P62075;A0A1W2P756	Mitochondrial import inner membrane trans	Timm13
25.5285	25.32045	25.63478	P62141;A0A0J9YUU8;A0A0J9YUG2	P62141	Serine/threonine-protein phosphatase PP1-	Ppp1cb
25.81909	25.99766	25.7543	P62274	P62274	40S ribosomal protein S29	Rps29
24.94056	24.64306	24.86703	P62305;E9Q4F4;E9Q0W8	P62305;E9Q4F4	Small nuclear ribonucleoprotein E	Snrpe
26.47073	26.56238	25.97158	P62315	P62315	Small nuclear ribonucleoprotein Sm D1	Snrpd1
23.59833	24.26785	23.96719	P62746	P62746	Rho-related GTP-binding protein RhoB	Rhob
25.08132	24.66492	24.90426	P62878	P62878	E3 ubiquitin-protein ligase RBX1	Rbx1
26.15846	25.89966	26.22435	P63087;A0A0G2JGC1;A0A0G2JFF1	P63087;A0A0G2JGC1;A0A	Serine/threonine-protein phosphatase PP1-	Ppp1cc
25.65949	25.79203	26.10149	P63321;A0A1Y7VL93	P63321;A0A1Y7VL93	Ras-related protein Ral-A;Ras-related protei	Rala
30.68813	30.75334	30.42342	P68369;P05214;Q3UX10	P68369;P05214	Tubulin alpha-1A chain;Tubulin alpha-3 chai	Tuba1a;Tuba3a
24.49228	24.22435	24.18707	P70280	P70280	Vesicle-associated membrane protein 7	Vamp7
26.58978	26.54775	25.82396	P83741	P83741	Serine/threonine-protein kinase WNK1	Wnk1
23.72858	23.99335	24.10949	P83887;Q8VCK3	P83887;Q8VCK3	Tubulin gamma-1 chain;Tubulin gamma-2 ch	Tubg1;Tubg2
NaN	23.33756	23.31008	P84089;A0A1W2P7H9;A0A1W2P7T3;G3	P84089;A0A1W2P7H9;A0A	Enhancer of rudimentary homolog	Erh
25.91798	25.64031	25.66492	P84104;A2A4X6	P84104	Serine/arginine-rich splicing factor 3	Srsf3
NaN	NaN	NaN	P97348;G3X9Y4	P97348	Rho-related GTP-binding protein RhoD	Rhod
26.95394	26.90885	26.82639	P97450;E9QAD6	P97450	ATP synthase-coupling factor 6, mitochondri	Atp5j
23.21697	23.42982	24.06907	P97465	P97465	Docking protein 1	Dok1
NaN	NaN	NaN	P97470;A0A0U1RPR5;A0A0U1RQ12	P97470	Serine/threonine-protein phosphatase 4 cat	Ppp4c
23.99335	24.74407	24.84326	P97494	P97494	Glutamate--cysteine ligase catalytic subunit	Gclc
23.66492	24.07725	23.83846	P97770;D3YYB2	P97770	THUMP domain-containing protein 3	Thumpd3
NaN	NaN	NaN	P98064	P98064	Mannan-binding lectin serine protease 1	Masp1
24.90426	25.00624	24.78457	P99025	P99025	GTP cyclohydrolase 1 feedback regulatory p	Gchfr
24.37119	23.86703	23.20508	Q02257	Q02257	Junction plakoglobin	Jup
NaN	NaN	NaN	Q04690;Q5SYH9	Q04690	Neurofibromin	Nf1
NaN	NaN	NaN	Q04750	Q04750	DNA topoisomerase 1	Top1
24.66492	24.16423	23.56384	Q06138	Q06138	Calcium-binding protein 39	Cab39
NaN	23.391	23.83846	Q3B7Z2	Q3B7Z2	Oxysterol-binding protein 1	Osbp
24.33074	24.01903	24.59263	Q3TC93;A0A1W2P6R8	Q3TC93;A0A1W2P6R8	HCLS1-binding protein 3;HCLS1-binding prot	Hs1bp3
23.1991	22.83653	NaN	Q3TFD2	Q3TFD2	Lysophosphatidylcholine acyltransferase 1	Lpcat1
24.22435	23.01052	23.99335	Q3TL72;Q8C878	Q3TL72;Q8C878	NEDD8-activating enzyme E1 catalytic subun	Uba3
NaN	NaN	NaN	Q3TLP5;F6Z5N5;F6YTG0	Q3TLP5;F6Z5N5;F6YTG0	Enoyl-CoA hydratase domain-containing pro	Echdc2
NaN	22.69073	23.26785	Q3TYL7;Q9QYJ3	Q3TYL7;Q9QYJ3	DnaJ homolog subfamily B member 1	Dnajb1
24.22435	23.88577	23.75939	Q3U1V6;A0A140LHE8;A0A1B0GSG9	Q3U1V6	Ubiquitin-conjugating enzyme E2 variant 3	Uevld
NaN	NaN	NaN	Q3U1Z5;G3UYL4	Q3U1Z5	G-protein-signaling modulator 3	Gpsm3
25.83846	25.23535	25.71555	Q3UGC7;Q66JS6	Q3UGC7;Q66JS6	Eukaryotic translation initiation factor 3 sub	Eif3j1;Eif3j2
25.23535	24.96719	24.72858	Q3UGR5	Q3UGR5	Haloacid dehalogenase-like hydrolase doma	Hdhd2
25.47692	25.63201	25.56384	Q3UMB5	Q3UMB5	Guanine nucleotide exchange protein SMCR	Smcr8
NaN	NaN	NaN	Q3V1G4	Q3V1G4	Olfactomedin-like protein 2B	Olfml2b
NaN	NaN	NaN	Q4VBE8	Q4VBE8	WD repeat-containing protein 18	Wdr18
23.89504	23.62087	23.82881	Q544X6;P22315	Q544X6;P22315	Ferrochelatase;Ferrochelatase, mitochondri	Fech
23.07725	22.06907	23.67573	Q571E4;Q8CC47	Q571E4;Q8CC47	N-acetylgalactosamine-6-sulfatase	Galns
24.04427	23.96719	24.29614	Q5Y5T1	Q5Y5T1	Probable palmitoyltransferase ZDHHC20	Zdhhc20
NaN	NaN	NaN	Q61112	Q61112	45 kDa calcium-binding protein	Sdf4
NaN	23.5285	23.57542	Q61249	Q61249	Immunoglobulin-binding protein 1	Igbp1
25.41054	25.75175	25.2856	Q61469	Q61469	Phospholipid phosphatase 1	Plpp1
25.20583	24.43619	24.26069	Q61625	Q61625	Glutamate receptor ionotropic, delta-2	Grid2
24.17188	23.2006	23.16883	Q61635;A0A1L1SQM2	Q61635;A0A1L1SQM2	GTP-binding protein;Interferon gamma-indu	Ifi47
24.39754	24.5285	24.51051	Q61771;A0A1D5RLM0;A0A0R4J220;A2A	Q61771	Kinesin-like protein KIF3B	Kif3b
24.53445	25.23169	25.59833	Q62084	Q62084	Protein phosphatase 1 regulatory subunit 14	Ppp1r14b
21.95305	NaN	NaN	Q62093	Q62093	Serine/arginine-rich splicing factor 2	Srsf2
NaN	23.09507	23.18707	Q64010;Q3TQV3;Q8JZR2;Q5ND50	Q64010	Adapter molecule crk	Crk
27.28912	26.95394	27.75175	Q64152	Q64152	Transcription factor BTF3	Btf3
26.45356	26.75175	27.39917	Q64339	Q64339	Ubiquitin-like protein ISG15	Isg15
27.18896	27.24445	26.11347	Q64523;Q6GSS7;Q64522	Q64523;Q6GSS7	Histone H2A type 2-C;Histone H2A type 2-A	Hist2h2ac;Hist2
NaN	NaN	NaN	Q68FE6	Q68FE6	Rho family-interacting cell polarization regul	Ripor1
NaN	NaN	NaN	Q6DID7	Q6DID7	Protein wntless homolog	Wls
27.2535	25.92931	27.89157	Q6GV12	Q6GV12	3-ketodihydrosphingosine reductase	Kdsr
25.05258	25.09346	25.12928	Q6NS82;A0A087WNS5;A0A087WNV4;A	Q6NS82;A0A087WNS5;A0	Reticulophagy regulator 2;Reticulophagy reg	Retreg2
23.35111	23.417	23.87643	Q6P5B5;Q9WVR4	Q6P5B5;Q9WVR4	Fragile X mental retardation syndrome-relat	Fxr2
26.40406	26.60399	26.2535	Q6P5F7	Q6P5F7	Protein tweety homolog 3	Ttyh3
25.01903	25.18329	25.32045	Q6P5H2	Q6P5H2	Nestin	Nes
NaN	NaN	NaN	Q6PFB2;Q8VE37	Q6PFB2;Q8VE37	Rcc1 protein;Regulator of chromosome con	Rcc1
NaN	NaN	NaN	Q6PFQ7;F6WTE9	Q6PFQ7	Ras GTPase-activating protein 4	Rasa4
25.76446	25.71555	25.7415	Q6ZWQ7	Q6ZWQ7	Signal peptidase complex subunit 3	Spcs3
26.44411	26.038	26.27142	Q6ZWY3;D6RH49;D3YYB0	Q6ZWY3;D6RH49;D3YYB0	40S ribosomal protein S27-like;40S ribosoma	Rps27l

NaN	NaN	NaN	Q7M759	Q7M759	Protein ABHD17B	Abhd17b
NaN	NaN	NaN	Q7TMY7;B2KGD7	Q7TMY7;B2KGD7	Importin-8;Importin-8 (Fragment)	Ipo8
26.1035	26.81421	26.06085	Q80UP8	Q80UP8	Sodium-dependent phosphate transporter 2	Slc20a2
NaN	NaN	23.28207	Q80UW2	Q80UW2	F-box only protein 2	Fbxo2
24.12534	25.08132	24.77455	Q80VJ3	Q80VJ3	2-deoxynucleoside 5-phosphate N-hydrolase	Dnph1
22.48493	22.57311	22.86136	Q80YV4;F7B6K4	Q80YV4	Pantothenate kinase 4	Pank4
25.86703	25.75175	25.89966	Q8BG13;O89086;S4R2M6	Q8BG13;O89086	RNA-binding protein 3	Rbm3
NaN	NaN	NaN	Q8BG60	Q8BG60	Thioredoxin-interacting protein	Txnip
24.15653	24.49838	24.22435	Q8BH58;A0A0A6YY87	Q8BH58	TIP41-like protein	Tiprl
24.77957	24.79947	24.61526	Q8BH79;A0A1L1SS60;A0A1L1SR14	Q8BH79;A0A1L1SS60	Anoctamin-10;Anoctamin	Ano10
25.64306	25.97596	25.33756	Q8BHC4;B0QZX9	Q8BHC4;B0QZX9	Dephospho-CoA kinase domain-containing p	Dcakd
NaN	23.40406	23.77957	Q8BIW1	Q8BIW1	Exopolyphosphatase PRUNE1	Prune1
24.06907	24.00196	24.23169	Q8BJF9	Q8BJF9	Charged multivesicular body protein 2b	Chmp2b
24.28912	24.36453	25.33415	Q8BS45;E0CYE0;E0CXQ7;E0CX73	Q8BS45;E0CYE0	Intraflagellar transport protein 56;Intraflage	Ttc26
23.1047	23.04093	NaN	Q8BTS3	Q8BTS3	Guanylate-binding protein 9	Gbp9
23.44253	22.89689	22.89319	Q8BTZ7	Q8BTZ7	Mannose-1-phosphate guanyltransferase be	Gmppb
25.11744	25.04009	25.12139	Q8BVF2;A0A0A6YXV1	Q8BVF2	Phosducin-like protein 3	Pdcl3
24.90426	25.4073	25.35111	Q8BVG4	Q8BVG4	Dipeptidyl peptidase 9	Dpp9
25.71555	25.75175	25.97815	Q8BYI6	Q8BYI6	Lysophosphatidylcholine acyltransferase 2	Lpcat2
24.20955	24.20955	24.15653	Q8BYI8	Q8BYI8	Protein FAM234B	Fam234b
23.26785	22.87079	23.35111	Q8BYY4	Q8BYY4	Tetratricopeptide repeat protein 39B	Ttc39b
22.56384	NaN	NaN	Q8BZ03;D3Z6I0;Q5FWX6;Q8K1Y2;Q621	Q8BZ03	Serine/threonine-protein kinase D2	Prkd2
24.84326	24.80931	25.04427	Q8C1E7;D3Z0U3	Q8C1E7	Transmembrane protein 120A	Tmem120a
24.239	24.05258	24.32389	Q8C3Y4	Q8C3Y4	Kinetochore-associated protein 1	Kntc1
25.68646	25.83846	25.78457	Q8C7K6;A0A0A6YY26	Q8C7K6;A0A0A6YY26	Prenylcysteine oxidase-like	Pcyox1l
NaN	NaN	22.42214	Q8CBQ5	Q8CBQ5	Phosphatidylinositol 4-kinase type 2-beta	Pi4k2b
29.1795	28.66966	28.64924	Q8CCG1	Q8CCG1	Zinc finger C2HC domain-containing protein	Zc2hc1c
23.08537	23.29614	23.26785	Q8CCH2	Q8CCH2	NHL repeat-containing protein 3	Nhlrc3
23.2021	23.51653	23.04926	Q8CDM8	Q8CDM8	Protein FAM160B1	Fam160b1
23.60964	23.36453	23.28207	Q8K057;D3Z157;D3YWI8	Q8K057	Intraflagellar transport protein 80 homolog	Ift80
23.48001	23.40406	23.68646	Q8K298;A0A1L1SS34	Q8K298;A0A1L1SS34	Anillin;Anillin (Fragment)	Anln
19.20658	NaN	NaN	Q8K2B0	Q8K2B0	Endoplasmic reticulum protein SC65	P3h4
25.62923	25.42662	25.75175	Q8K2C9	Q8K2C9	Very-long-chain (3R)-3-hydroxyacyl-CoA deh	Hacd3
23.79947	23.82881	23.70767	Q8K2V6	Q8K2V6	Importin-11	Ipo11
27.88577	27.89735	27.86231	Q8K3G9	Q8K3G9	DCC-interacting protein 13-beta	Appl2
23.21697	24.417	NaN	Q8K4X7	Q8K4X7	1-acyl-sn-glycerol-3-phosphate acyltransfera	Agpat4
24.59833	24.76446	24.57542	Q8K4Z3	Q8K4Z3	NAD(P)H-hydrate epimerase	Naxe
23.66492	NaN	23.72858	Q8R0J7	Q8R0J7	Vacuolar protein sorting-associated protein	Vps37b
22.29894	23.42982	24.10949	Q8R2Y0	Q8R2Y0	Monoacylglycerol lipase ABHD6	Abhd6
25.28912	25.48922	25.46451	Q8VBV7;A0A087WPM5	Q8VBV7;A0A087WPM5	COP9 signalosome complex subunit 8;COP9	Cops8
NaN	25.32389	25.22803	Q8VC30	Q8VC30	Triokinase/FMN cyclase	Tkfc
NaN	NaN	NaN	Q8VCJ6	Q8VCJ6	Mas-related G-protein coupled receptor me	Mrgprf
NaN	NaN	NaN	Q8VDV8	Q8VDV8	MIT domain-containing protein 1	Mitd1
25.64856	25.27142	25.43936	Q8VE47	Q8VE47	Ubiquitin-like modifier-activating enzyme 5	Uba5
26.04218	26.02327	26.26964	Q8VEH3;F6QKK2	Q8VEH3;F6QKK2	ADP-ribosylation factor-like protein 8A;ADP-	Arl8a
24.37119	24.59833	24.03591	Q8VEK0;D3YVV1;Q8BHG3	Q8VEK0;D3YVV1	Cell cycle control protein 50A	Tmem30a
24.37119	24.27498	23.96719	Q8VHM5;F7B5B5;A2AW41	Q8VHM5;F7B5B5;A2AW41	Heterogeneous nuclear ribonucleoprotein R	Hnrnpr
24.51653	25.28207	24.96279	Q91V01;A0A0N4SUM3	Q91V01	Lysophospholipid acyltransferase 5	Lpcat3
NaN	NaN	NaN	Q91V08;A0A0N4SUR3;F5CSM7;Q9D676	Q91V08	C-type lectin domain family 2 member D	Clec2d
26.63201	26.69975	26.75175	Q91VW3;I7HPY0	Q91VW3	SH3 domain-binding glutamic acid-rich-like p	Sh3bgrl3
23.63201	NaN	NaN	Q91XH6;O88384;E0CYE5;F6UHS3	Q91XH6;O88384	Vesicle transport through interaction with t-	Vti1b
NaN	24.95394	NaN	Q91YT8;E0CX44;E0CXM3	Q91YT8	CSC1-like protein 1	Tmem63a
23.83846	22.7348	23.28207	Q923G2	Q923G2	DNA-directed RNA polymerases I, II, and III s	Polr2h
23.94949	23.46762	24.06085	Q925I1	Q925I1	ATPase family AAA domain-containing prote	Atad3
25.30661	25.05672	25.01903	Q99J09;F7D5L2	Q99J09;F7D5L2	Methylosome protein 50;Methylosome prot	Wdr77
25.08537	24.7024	24.91798	Q99J77	Q99J77	Sialic acid synthase	Nans
26.02749	26.12337	26.239	Q99JI6;A0A1W2P777	Q99JI6	Ras-related protein Rap-1b	Rap1b
23.81909	NaN	NaN	Q99KD5;D6RIN1;A0A0U1RNI4;D3YZN8	Q99KD5;D6RIN1	Protein unc-45 homolog A	Unc45a
23.93157	24.06085	24.01052	Q99KH8;A2AD84;Q99JT2;D3Z359;Q9Z2	Q99KH8;A2AD84;Q99JT2	Serine/threonine-protein kinase 24;Serine/t	Stk24;Stk26
26.82639	26.08335	25.6971	Q99LC3;A0A087WR38	Q99LC3	NADH dehydrogenase [ubiquinone] 1 alpha	Ndufa10
25.22066	25.1946	25.2021	Q99MB1	Q99MB1	Toll-like receptor 3	Tlr3
24.60964	24.58118	24.28912	Q99P58;Q9ERI2	Q99P58	Ras-related protein Rab-27B	Rab27b
23.78955	23.86703	24.03591	Q9CQ10;A0A0N4SVS3	Q9CQ10	Charged multivesicular body protein 3	Chmp3
25.28207	25.239	25.50446	Q9CQ48;E0CYQ2	Q9CQ48;E0CYQ2	NudC domain-containing protein 2	Nudcd2
25.42021	25.6971	25.36119	Q9CQ69	Q9CQ69	Cytochrome b-c1 complex subunit 8	Uqcrq
26.33415	26.60399	26.48769	Q9CQ88	Q9CQ88	Tetraspanin-31	Tspan31
24.89504	25.27853	24.79452	Q9CQF7;Q9CWM4	Q9CQF7;Q9CWM4	Prefoldin 1;Prefoldin subunit 1	Pfdn1
22.34299	NaN	NaN	Q9CQJ8	Q9CQJ8	NADH dehydrogenase [ubiquinone] 1 beta s	Ndufb9
NaN	NaN	NaN	Q9CRB3;A0A1B0GR99;A0A0A0MQC3;A(	Q9CRB3;A0A1B0GR99;A0A	5-hydroxyisourate hydrolase;5-hydroxyisour	Urah
24.71293	25.1332	24.64306	Q9CS42	Q9CS42	Ribose-phosphate pyrophosphokinase 2	Prps2
25.43301	24.98902	25.31354	Q9CX34	Q9CX34	Protein SGT1 homolog	Sugt1
24.86231	24.46139	24.64306	Q9CYG7	Q9CYG7	Mitochondrial import receptor subunit TOM	Tomm34
25.57542	25.44569	25.61526	Q9CYL5;B1AWD6	Q9CYL5;B1AWD6	Golgi-associated plant pathogenesis-related	Glipr2
24.43619	24.12534	24.76446	Q9CZE3;A0A1Y7VIZ0	Q9CZE3	Ras-related protein Rab-32	Rab32
25.84566	25.68913	25.75685	Q9D104;G5E8T3;Q9D7A6	Q9D104;G5E8T3;Q9D7A6	Signal recognition particle 19 kDa protein	Srp19
24.85757	24.97158	25.32389	Q9D1B1	Q9D1B1	Cystatin	Cst6
25.42662	24.56384	24.6811	Q9D281	Q9D281	Protein Noxp20	Fam114a1
NaN	23.72858	23.99335	Q9D554	Q9D554	Splicing factor 3A subunit 3	Sf3a3
24.58118	24.71293	25.2021	Q9D7M5	Q9D7M5	Dynactin-associated protein	Dynap
26.65949	26.92025	26.63201	Q9D7S7	Q9D7S7	60S ribosomal protein L22-like 1	Rpl22l1
25.04843	25.39428	25.18329	Q9D7S9	Q9D7S9	Charged multivesicular body protein 5	Chmp5
28.21234	26.41538	26.71293	Q9D823	Q9D823	60S ribosomal protein L37	Rpl37
27.34096	27.12139	27.26249	Q9D881;P19536;F7C106;A0A0A6YVR0	Q9D881;P19536	Cytochrome c oxidase subunit 5B, mitochon	Gm11273;Cox5l
23.74919	23.18404	23.21549	Q9D883;G3UW94	Q9D883;G3UW94	Splicing factor U2AF 35 kDa subunit;MCG14	U2af1
26.08537	26.34604	25.64581	Q9D8C4;D6RFB1	Q9D8C4	Interferon-induced 35 kDa protein homolog	Ifi35
25.55801	25.86231	25.85043	Q9D952	Q9D952	Envoplakin	Evpl
NaN	24.23169	NaN	Q9DB25;D6RCG2	Q9DB25;D6RCG2	Dolichyl-phosphate beta-glucosyltransferase	Alg5
22.94592	23.03591	22.87455	Q9DBD5;Q9D4T3	Q9DBD5	Proline-, glutamic acid- and leucine-rich prot	Pelp1

24.64306	24.391	24.417	Q9DCT2	Q9DCT2	NADH dehydrogenase [ubiquinone] iron-sulf	Ndufs3
26.34435	26.63201	26.2732	Q9DCW5;P43024	Q9DCW5;P43024	Cytochrome c oxidase subunit 6A, mitochon	Cox6a1
22.88017	22.29894	22.48247	Q9EP52	Q9EP52	Twisted gastrulation protein homolog 1	Twsg1
24.42982	24.29614	24.30313	Q9EPK2	Q9EPK2	Protein XRP2	Rp2
25.83364	26.45513	26.30487	Q9ER00	Q9ER00	Syntaxin-12	Stx12
23.35111	21.9566	NaN	Q9ERU9;Q99KR7	Q9ERU9	E3 SUMO-protein ligase RanBP2	Ranbp2
24.08537	NaN	23.31008	Q9ESW8;A0A1B0GSY1	Q9ESW8;A0A1B0GSY1	Pyroglutamyl-peptidase 1	Pgpep1
23.83846	23.57542	NaN	Q9ESY9	Q9ESY9	Gamma-interferon-inducible lysosomal thiol	Ifi30
NaN	NaN	NaN	Q9JHE3	Q9JHE3	Neutral ceramidase	Asah2
NaN	NaN	NaN	Q9JIG8;A0A140LJ36	Q9JIG8;A0A140LJ36	PRA1 family protein 2;PRA1 family protein	Praf2;Gm45208
24.30313	25.04843	24.56384	Q9JK92	Q9JK92	Heat shock protein beta-8	Hspb8
25.00196	25.239	24.8811	Q9JKX6;A0A0A6YVU1;A2ATT5	Q9JKX6;A0A0A6YVU1;A2A	ADP-sugar pyrophosphatase;ADP-sugar pyro	Nudt5
24.10949	24.08537	23.6971	Q9JLB0;E9PWC5	Q9JLB0	MAGUK p55 subfamily member 6	Mpp6
NaN	NaN	NaN	Q9JLQ0	Q9JLQ0	CD2-associated protein	Cd2ap
23.68646	23.89504	24.00196	Q9QYY7	Q9QYY7	Endothelial cell-specific molecule 1	Esm1
24.65403	24.94056	25.1946	Q9QZI8;E9Q553;E9PY81	Q9QZI8	Serine incorporator 1	Serinc1
23.04926	NaN	NaN	Q9R0H0;A2A848	Q9R0H0;A2A848	Peroxisomal acyl-coenzyme A oxidase 1;Pero	Acox1
25.30313	24.97158	24.54628	Q9WTP6;F7BP55	Q9WTP6	Adenylate kinase 2, mitochondrial	Ak2
23.92252	24.04427	24.31008	Q9WTX2	Q9WTX2	Interferon-inducible double-stranded RNA-d	Prkra
25.73892	25.50142	25.38772	Q9WU28;E9Q5Q8;E9Q093;H7BWX1;E9I	Q9WU28	Prefoldin subunit 5	Pfdn5
NaN	21.74304	NaN	Q9Z0M5	Q9Z0M5	Lysosomal acid lipase/cholesteryl ester hydr	Lipa
26.13907	26.18896	25.92931	Q9Z0Y1;E9Q919	Q9Z0Y1;E9Q919	Dynactin subunit 3	Dctn3
24.42982	24.54038	24.67573	Q9Z1J3;A6QRH3;F7CZD1;F6TXD3	Q9Z1J3;A6QRH3	Cysteine desulfurase, mitochondrial	Nfs1
23.76951	23.54038	23.76951	Q9Z1K5;H7BWY7;H9KV21	Q9Z1K5;H7BWY7;H9KV21	E3 ubiquitin-protein ligase ARIH1	Arih1
24.45513	23.64306	24.17188	Q9Z1M2;A0A140LIF8	Q9Z1M2;A0A140LIF8	Immunity-related GTPase family M member	Irgm2
23.32389	NaN	22.95127	Q9Z1N5;G3UXI6	Q9Z1N5	Spliceosome RNA helicase Ddx39b	Ddx39b
NaN	NaN	23.40406	S4R189;Q3UPH7;S4R2U9	S4R189;Q3UPH7;S4R2U9	Rho guanine nucleotide exchange factor 40	Arhgef40
23.82881	23.95837	24.00196	S4R1L5;S4R2P8;O88738;REV__Q5NCS9;	S4R1L5;S4R2P8;O88738	Baculoviral IAP repeat-containing protein 6	Birc6
23.391	22.77555	22.68003	Z4YK42;Q8C5H8	Z4YK42;Q8C5H8	NAD kinase 2, mitochondrial	Nadk2
23.08861	22.75735	23.29614	A0A087WNN8;Q61263;A0A087WSJ5	A0A087WNN8;Q61263;A0	Sterol O-acyltransferase 1 (Fragment);Sterol	Soat1
NaN	NaN	NaN	A0A087WNZ7;G5E870;A0A087WRV6;A0	A0A087WNZ7;G5E870;A0	E3 ubiquitin-protein ligase TRIP12	Trip12
NaN	22.70978	NaN	A0A087WQ65;A0A087WRZ2;A0A087WI	A0A087WQ65;A0A087WR	Lymphocyte antigen 6E (Fragment)	Ly6e
25.15653	24.86231	24.84326	A0A087WR57;Q91XC8	A0A087WR57;Q91XC8	Death-associated protein 1	Dap
NaN	24.71293	NaN	A0A087WRB8;A0A087WPR7;A0A087WS	A0A087WRB8;A0A087WP	Dystonin (Fragment);Dystonin	Dst
NaN	22.95127	23.42982	A0A0A6YW28;P35123;A0A0A6YVY7;Q9	A0A0A6YW28;P35123;A0	Ubiquitin carboxyl-terminal hydrolase 4	Usp4
NaN	NaN	NaN	A0A0A6YWM5;E9QKE4;Q8BMG7	A0A0A6YWM5;E9QKE4;Q	Rab3 GTPase-activating protein non-catalyti	Rab3gap2
26.35616	27.1795	27.19835	A0A0G2JE32;A0A0G2JGL0;P62838;P610	A0A0G2JE32;A0A0G2JGL0	Ubiquitin-conjugating enzyme E2 D3 (Fragm	Ube2d3
26.44411	25.01052	23.29614	A0A0G2JF44;Q9DAI2;A0A0H2UKB7;A0A	A0A0G2JF44;Q9DAI2;A0A	Intraflagellar transport protein 22 homolog	Ift22
23.23316	NaN	22.82104	A0A0G2JG35;A0A140T8T5;E9PZ43;P275	A0A0G2JG35;A0A140T8T5	Microtubule-associated protein (Fragment);	Map4
24.87173	24.26069	23.32389	A0A0J9YUN6;Q0VGU5;Q6TEK5	A0A0J9YUN6;Q0VGU5;Q6	Vitamin K epoxide reductase complex subun	Vkorc1l1
22.96895	NaN	NaN	A0A0J9YUR5;A0A0J9YUM4;A0A0J9YU62	A0A0J9YUR5;A0A0J9YUM	C-terminal-binding protein 1 (Fragment);C-te	Ctbp1
NaN	NaN	24.23169	A0A0J9YV30;F8VQ28;Q8VI36	A0A0J9YV30;F8VQ28;Q8V	Paxillin	Pxn
NaN	NaN	NaN	A0A0J9YVG0;Q61074	A0A0J9YVG0;Q61074	Protein phosphatase 1G	Ppm1g
24.23169	24.45513	24.08537	A0A0N4SUH8;Q9QZ23;D3Z285	A0A0N4SUH8;Q9QZ23;D3	NFU1 iron-sulfur cluster scaffold homolog, m	Nfu1
24.74919	24.53445	24.49838	A0A0N4SWD0;Q9CZC8	A0A0N4SWD0;Q9CZC8	Secernin-1 (Fragment);Secernin-1	Scrn1
NaN	NaN	NaN	A0A0R4J097;O88393	A0A0R4J097;O88393	Transforming growth factor beta receptor ty	Tgfbr3
NaN	NaN	NaN	A0A0R4J0T5;P28659;S4R2S7;A0A0R4J2E	A0A0R4J0T5;P28659;S4R2	CUG triplet repeat, RNA binding protein 1, is	Celf1
NaN	NaN	NaN	A0A0R4J126;Q8BS03	A0A0R4J126;Q8BS03	Peptidase inhibitor 15	Pi15
NaN	NaN	23.03086	A0A0R4J139;D3YUC9;Q8BU85	A0A0R4J139;D3YUC9;Q8B	Methionine sulfoxide reductase B3;Methion	Msrb3
23.95837	NaN	23.51653	A0A0R4J173;G5E8C4;Q8BRH0	A0A0R4J173;G5E8C4;Q8B	Transmembrane and TPR repeat-containing	Tmtc3
21.75532	NaN	NaN	A0A0R4J196;F8WH31;E9PUD1;Q58A65;	A0A0R4J196;F8WH31;E9P	C-Jun-amino-terminal kinase-interacting pro	Spag9
23.89504	23.60964	24.31008	A0A0R4J233;A0A1W2P6J7;Q8C650	A0A0R4J233;A0A1W2P6J7	Septin-10	Sept10
22.63423	22.47011	24.06907	A0A0R4J275;Q7TMF3	A0A0R4J275;Q7TMF3	NADH dehydrogenase [ubiquinone] 1 alpha	Ndufa12
24.45513	NaN	NaN	A0A140LHY2;Q7TMR0	A0A140LHY2;Q7TMR0	Lysosomal Pro-X carboxypeptidase	Prcp
23.37782	23.31008	23.78955	A0A140LIJ2;A0A140LJ29;A0A140LIQ6;E	A0A140LIJ2;A0A140LJ29;A	Transforming acidic coiled-coil-containing pr	Tacc2
25.73635	25.69445	25.60399	A0A140T8J4;Q9R257	A0A140T8J4;Q9R257	Heme-binding protein 1	Hebp1
23.391	23.42982	23.50446	A0A1D5RLY2;Q80WQ2	A0A1D5RLY2;Q80WQ2	Protein VAC14 homolog	Vac14
26.00838	26.00838	26.4008	A0A1L1SRJ4;Q9QY33;A0A1L1SQ19	A0A1L1SRJ4;Q9QY33;A0A	Tetraspanin;Tetraspanin-3;Tetraspanin (Frag	Tspan3
NaN	NaN	24.14102	A0A1L1SRR1;Q9ESD6	A0A1L1SRR1;Q9ESD6	CKLF-like MARVEL transmembrane domain-	Cmtm7
23.26785	23.65403	23.40406	A0A1L1SS10;Q9Z2X8	A0A1L1SS10;Q9Z2X8	Kelch-like ECH-associated protein 1	Keap1
22.39624	22.63201	22.54038	A0A1L1STF0;Q6PDI6	A0A1L1STF0;Q6PDI6	Ubiquitin carboxyl-terminal hydrolase MIND	Mindy2
NaN	NaN	NaN	A0A1N9M4K1;A0A1L1SU77;P70665;A0	A0A1N9M4K1;A0A1L1SU7	Sialate O-acetylesterase	Siae
NaN	NaN	NaN	A0A1W2P7L7;P59708	A0A1W2P7L7;P59708	Splicing factor 3B subunit 6 (Fragment);Splic	Sf3b6
23.94056	24.2021	23.94056	A0A1W2P7Z1;Q8VHI3;A0A1W2P6H4	A0A1W2P7Z1;Q8VHI3;A0	GDP-fucose protein O-fucosyltransferase 2 (	Pofut2
22.99335	22.87267	22.8839	A0A1W2P8A8;Q99KK1	A0A1W2P8A8;Q99KK1	Receptor expression-enhancing protein;Rec	Reep3
NaN	NaN	NaN	A0A1Y7VKX7;F8WJA0;Q9ESV0;A0A1Y7V	A0A1Y7VKX7;F8WJA0;Q9E	ATP-dependent RNA helicase DDX24	Ddx24
NaN	NaN	NaN	A0A1Y7VLW2;W4VSP4;F7B9A0;O08804	A0A1Y7VLW2;W4VSP4;F7	Serine (or cysteine) peptidase inhibitor, clad	Serpinb6c
25.30313	24.30313	25.96499	A0A1Y7VMA0;A0A1Y7VL95;Q9JI75	A0A1Y7VMA0;A0A1Y7VL9	Ribosyldihydronicotinamide dehydrogenase	Nqo2
24.05258	NaN	NaN	A0A1Y7VNI1;G3X9B1	A0A1Y7VNI1;G3X9B1	HEAT repeat-containing 1 (Fragment);HEAT	Heatr1
NaN	NaN	NaN	A2A5V3;A2A5V2;S4R2D3;P55194;S4R2T	A2A5V3;A2A5V2;S4R2D3;	SH3 domain-binding protein 1	Sh3bp1
22.32113	22.31562	22.11108	A2AA71;Q3U2P1	A2AA71;Q3U2P1	Protein transport protein Sec24A	Sec24a
24.53445	24.317	NaN	A2ACM0;Q8K4Q0	A2ACM0;Q8K4Q0	Putative uncharacterized protein;Regulatory	Rptor
24.44253	24.51653	24.42982	A2ADY9	A2ADY9	Protein DDI1 homolog 2	Ddi2
NaN	NaN	22.51893	A2AE27;Q9DBT5	A2AE27;Q9DBT5	AMP deaminase;AMP deaminase 2	Ampd2
24.44253	23.50446	24.70767	A2AF67;A2AF47	A2AF67;A2AF47	Dedicator of cytokinesis protein 11	Dock11
23.71816	23.63201	24.05258	A2AGA4	A2AGA4	Rhomboid-related protein 2	Rhbdl2
23.40406	NaN	NaN	A2AJ72;Q3TIX6;A0A0A6YVV5;A0A0A6Y	A2AJ72;Q3TIX6;A0A0A6Y	Far upstream element (FUSE)-binding protei	Fubp3
NaN	NaN	NaN	A2AM65;Z4YKA3;Z4YKB8;Q3TEA8	A2AM65;Z4YKA3;Z4YKB8;	Heterochromatin protein 1-binding protein	Hp1bp3
23.60964	23.92252	23.49228	A2AMC3;Q3UXG7;Q91ZW2;E9PZ15;E9	A2AMC3;Q3UXG7;Q91ZW	GDP-fucose protein O-fucosyltransferase 1	Pofut1
25.22435	NaN	24.01052	A2AMI7;Z4YKC4;A0A0R4J112;A2AMI2;A	A2AMI7;Z4YKC4;A0A0R4J1	Eukaryotic translation initiation factor 4 gam	Eif4g3
23.65403	25.79699	25.54333	A2AMQ5;Q99L43;Q6PBC0;F6S4G2	A2AMQ5;Q99L43;Q6PBC0	Phosphatidate cytidylyltransferase;Phospha	Cds2
26.60399	27.43141	27.20769	A2AQE2;Q6A098	A2AQE2;Q6A098	SECIS binding protein 2-like;Selenocysteine i	Secisbp2l
NaN	NaN	NaN	A2ARZ7;P35285	A2ARZ7;P35285	RAB22A, member RAS oncogene family, isof	Rab22a
23.15653	23.10789	22.72442	A2ATI8;A2ATI6;A2ATI9;Q99JX3	A2ATI8;A2ATI6;A2ATI9;Q	Golgi reassembly-stacking protein 2;Golgi re	Gorasp2
NaN	22.03255	NaN	A2ATU9;Q8BY71	A2ATU9;Q8BY71	Histone acetyltransferase type B catalytic su	Hat1
25.68378	25.79699	26.16806	A2AUM6;P16254	A2AUM6;P16254	Signal recognition particle 14 kDa protein	Srp14
NaN	NaN	NaN	A2AW05;Q08943	A2AW05;Q08943	FACT complex subunit SSRP1 (Fragment);FA	Ssrp1
NaN	NaN	NaN	A3KGB4	A3KGB4	TBC1 domain family member 8B	Tbc1d8b

23.1991	22.92976	21.86136	A6H5Z3	A6H5Z3	Exocyst complex component 6B	Exoc6b
26.26964	26.34604	26.18329	A6PWS5	A6PWS5	Gelsolin (Fragment)	Gsn
23.02918	22.36986	22.69922	B1ARW4;Q99LY9	B1ARW4;Q99LY9	NADH dehydrogenase [ubiquinone] iron-sulf	Ndufs5
22.5708	22.2006	NaN	B1AU74;Q9CWP6	B1AU74;Q9CWP6	Motile sperm domain-containing protein 2	Mospd2
25.63478	25.91342	25.92705	B1AV14;Q78IK4	B1AV14;Q78IK4	MICOS complex subunit;MICOS complex sub	Apool
NaN	21.2535	22.04593	B1AXY5;P15535	B1AXY5;P15535	Beta-1,4-galactosyltransferase 1	B4galt1
27.63201	27.78955	27.81421	B1AYG7;B1AYG6;Q9Z0T9	B1AYG7;B1AYG6;Q9Z0T9	Integrin beta (Fragment);Integrin beta;Integ	Itgb6
NaN	NaN	NaN	B1AZ26;B1AZ15;B1AZ14;Q3UMF0	B1AZ26;B1AZ15;B1AZ14;Q	Cordon-bleu protein-like 1 (Fragment);Cordo	Cobll1;C
24.10149	24.57542	24.32389	B1AZ42;P0C0A3;B1AZ41	B1AZ42;P0C0A3;B1AZ41	Charged multivesicular body protein 6 (Frag	Chmp6
NaN	NaN	NaN	B1B1A8;Q6PDN3	B1B1A8;Q6PDN3	Myosin light chain kinase, smooth muscle	Mylk
26.13124	25.54038	NaN	B2RXA1	B2RXA1	PI-PLC X domain-containing protein 2	Plcxd2
23.18556	22.67573	23.36453	B7ZWM8;B2RXP1;E9QAU9;Q8BVU0	B7ZWM8;B2RXP1;E9QAU9	Leucine-rich repeat and calponin homology	Lrch3
24.34435	24.15653	24.417	D3YTS4;Q9JHS3	D3YTS4;Q9JHS3	Ragulator complex protein LAMTOR2	Lamtor2
22.15499	22.23608	22.40406	D3YUP1;Q9WVG6	D3YUP1;Q9WVG6	Histone-arginine methyltransferase CARM1	Carm1
22.57311	22.32663	23.20955	D3YUU4;Q61124;D3Z448;Q6PAH4	D3YUU4;Q61124;D3Z448;	Battenin (Fragment);Battenin	Cln3
23.31008	23.45513	23.28207	D3YVN5;D3YVM9;Q9JJS0;Q68EF9;A2A4	D3YVN5;D3YVM9;Q9JJS0;	Signal peptide, CUB and EGF-like domain-co	Scube2
24.20955	24.02749	23.86703	D3YW20;D3YW19;I1E4X5;Q8BWQ6	D3YW20;D3YW19;I1E4X5;	RIKEN cDNA 9030624J02 gene (Fragment)	9030624J02Rik
23.46762	23.37782	NaN	D3YWD1;J3QQ16;A0A087WS16;E9PWQ	D3YWD1;J3QQ16;A0A087	Collagen, type VI, alpha 3 (Fragment);Collag	Col6a3
24.98902	25.28912	25.26428	D3YWT0;D3Z569;D3YTS1;Q9R0P6	D3YWT0;D3Z569;D3YTS1;	Signal peptidase complex catalytic subunit S	Sec11a
23.13477	22.9061	23.21549	D3YWU4;D3YWA9;D3YTR6;Q8R0G7;D6	D3YWU4;D3YWA9;D3YTR	Protein spinster homolog 1 (Fragment);Prot	Spns1;Spn
23.66492	23.83846	24.01903	D3YXP6;Q9D1G2	D3YXP6;Q9D1G2	Phosphomevalonate kinase	Pmvk
23.72858	23.65403	NaN	D3YXZ3;Q91YS4;O88448;D3Z5Y7	D3YXZ3;Q91YS4;O88448	Kinesin light chain 2	Klc2
NaN	23.26785	23.5285	D3YYT0;P15116	D3YYT0;P15116	Cadherin-2	Cdh2
NaN	NaN	23.60964	D3YZP9	D3YZP9	Coiled-coil domain-containing protein 6	Ccdc6
23.37782	NaN	24.66492	D3Z0V2;A0A0R4J0X8;Q9ES28	D3Z0V2;A0A0R4J0X8;Q9E	Rho guanine nucleotide exchange factor 7 (F	Arhgef7
22.18858	NaN	22.06579	D3Z101;S4R1X1;Q7TMQ7	D3Z101;S4R1X1;Q7TMQ7	WD repeat-containing protein 91 (Fragment	Wdr91
NaN	NaN	23.33756	D3Z1Y5;Q8BL80	D3Z1Y5;Q8BL80	Rho GTPase-activating protein 22	Arhgap22
23.68646	24.08537	23.72858	D3Z3J6;G3UYE5;F6Y616;Q8VE62;G3UZ6	D3Z3J6;G3UYE5;F6Y616;Q	Polyadenylate-binding protein-interacting p	Paip1
23.88577	24.01903	23.79947	D3Z3L3;D3YYU4;E9PV98	D3Z3L3;D3YYU4;E9PV98	Tripartite motif-containing 12C;Tripartite mo	Trim12c;Trim5;
NaN	NaN	21.98815	D3Z3T5;P49586;Q811Q9	D3Z3T5;P49586;Q811Q9	Choline-phosphate cytidylyltransferase A	Pcyt1a
25.34096	25.27142	25.04843	D3Z3X4;Q9CQB5	D3Z3X4;Q9CQB5	CDGSH iron-sulfur domain-containing protei	Cisd2
23.63201	24.84326	24.21697	D3Z4B2;Q9CWZ7	D3Z4B2;Q9CWZ7	Gamma-soluble NSF attachment protein (Fra	Napg
25.02749	25.01478	25.01903	D3Z6D5;Q6TYB5	D3Z6D5;Q6TYB5	Fasciculation and elongation protein zeta-2	Fez2
23.15499	22.71607	24.05258	D3Z6I4;Q921W4;D3YU21;F7BGV1;D3YZ	D3Z6I4;Q921W4;D3YU21;	Crystallin, zeta (Quinone reductase)-like 1, is	Cryzl1
23.62087	23.48001	23.20508	D3Z7F0;P16125;A0A0N4SVV8	D3Z7F0;P16125;A0A0N4S	L-lactate dehydrogenase (Fragment);L-lactat	Ldhb
26.33926	26.31008	26.75175	D6RFN5;D3YW25;O70131	D6RFN5;D3YW25;O70131	Ninjurin-1;Ninjurin 1, isoform CRA_a	Ninj1
24.06907	24.67573	24.33756	D6RI20;Q9CR20	D6RI20;Q9CR20	Haloacid dehalogenase-like hydrolase doma	Hdhd2;Ier3ip1
NaN	NaN	NaN	E0CX20	E0CX20	Protein BUD31 homolog	Bud31
28.86821	28.87994	29.42942	E0CYE2;F7DEU6;P50096;E0CXK0	E0CYE2;F7DEU6;P50096;E	Inosine-5-monophosphate dehydrogenase	Impdh1
24.02749	23.84805	24.27498	E0CYH4;Q8C6G8;F7DFQ2	E0CYH4;Q8C6G8;F7DFQ2	WD repeat-containing protein 26;WD repeat	Wdr26
29.10649	28.00838	27.65267	E9PU87;F6U8X4;F6U6U5;F6S7W6;Q6P4	E9PU87;F6U8X4;F6U6U5;	Serine/threonine-protein kinase SIK3;Serine	Sik3
23.2535	NaN	NaN	E9PUE7;Q5SSL4;H3BJY3;Q6PAJ1	E9PUE7;Q5SSL4;H3BJY3;Q	Active breakpoint cluster region-related pro	Abr
22.96543	NaN	23.19009	E9PUW7;Q9EPK7	E9PUW7;Q9EPK7	Exportin-7	Xpo7
25.48001	25.83364	25.72338	E9PVN6;Q9CR63;Q9D6K5	E9PVN6;Q9CR63;Q9D6K5	Predicted gene 20498;Cytochrome c oxidase	Gm20498;Cox1
23.42982	NaN	NaN	E9PW03;Q9WTK3;E9QA52;F6T7Y8	E9PW03;Q9WTK3;E9QA52	Glycosylphosphatidylinositol anchor attachn	Gpaa1
24.1946	24.22435	24.22435	E9PWW2;Q921T2	E9PWW2;Q921T2	Torsin-1A-interacting protein 1	Tor1aip1
23.74919	23.74919	23.40406	E9PY51;A0A286YCX6;E9PWG2;A0A286Y	E9PY51;A0A286YCX6;E9P	Trafficking protein particle complex 8	Trappc8
NaN	NaN	NaN	E9PZS4;Q9D0T1	E9PZS4;Q9D0T1	NHP2-like protein 1	Snu13
NaN	22.82881	23.00539	E9Q0T0;G5E8W7;F6SPQ1;O35448;E9PV	E9Q0T0;G5E8W7;F6SPQ1;	Lysosomal thioesterase PPT2 (Fragment)	Ppt2
23.70767	23.76951	24.44253	E9Q0U3;D3Z7P0;Q8K3W0;A0A0J9YV56	E9Q0U3;D3Z7P0;Q8K3W0	BRISC and BRCA1-A complex member 2	Babam2
22.75532	22.53326	22.25926	E9Q1T3;E9PX30;Q8BPM2	E9Q1T3;E9PX30;Q8BPM2	Mitogen-activated protein kinase kinase kin	Map4k5
NaN	23.02073	22.85567	E9Q2R3;Q9EQN9	E9Q2R3;Q9EQN9	Thiamine transporter 1	Slc19a2
22.76344	22.29053	22.75939	E9Q467;E9Q236	E9Q467;E9Q236	ATP-binding cassette, sub-family C (CFTR/M	Abcc4
24.11744	24.15653	24.18707	E9Q481;Q6P2K6	E9Q481;Q6P2K6	Serine/threonine-protein phosphatase 4 reg	Ppp4r3a
23.16269	NaN	NaN	E9Q4K7;A0A286YCV9	E9Q4K7;A0A286YCV9	Kinesin family member 13B	Kif13b
NaN	26.71293	NaN	E9Q4K9;E9PYU8;Q9CUN6;A2A5Z6;A0A1	E9Q4K9;E9PYU8;Q9CUN6;	E3 ubiquitin-protein ligase;E3 ubiquitin-prot	Smurf1;Smu
NaN	23.97596	NaN	E9Q4T8;Q9JLV5	E9Q4T8;Q9JLV5	Cullin-3	Cul3
NaN	NaN	NaN	E9Q5B5;O08528	E9Q5B5;O08528	Hexokinase-2	Hk2
NaN	NaN	NaN	E9Q5E3;Q6NZR1;P28047	E9Q5E3;Q6NZR1;P28047	Protein Wnt;Protein Wnt-7b	Wnt7b
NaN	NaN	NaN	E9Q5J9	E9Q5J9	Tropomyosin alpha-3 chain	Tpm3
24.28912	NaN	24.08537	E9Q7B0	E9Q7B0	Prolyl 4-hydroxylase subunit alpha-1	P4ha1
25.44884	25.45199	26.33586	E9Q8N1;E9Q8K5;A2ASS6	E9Q8N1;E9Q8K5;A2ASS6	Titin	Ttn
24.76951	24.36453	24.38443	E9Q9C5;P63082;D3Z3B2	E9Q9C5;P63082;D3Z3B2	V-type proton ATPase proteolipid subunit (F	Atp6v0c
NaN	NaN	NaN	E9Q9H2;E9Q9H3;P54103;Q3TRX6	E9Q9H2;E9Q9H3;P54103;	DnaJ homolog subfamily C member 2	Dnajc2
24.33756	24.48616	24.38443	E9QAN4;G3UW47;Q6TA13;E9Q9G6;A0A	E9QAN4;G3UW47;Q6TA1	Kinesin-like protein KIF1A;Kinesin family me	Kif1a
21.96367	21.73892	NaN	E9QAT0;D3Z6U8;E9QNF5;Q6AXB7;P359	E9QAT0;D3Z6U8;E9QNF5;	Synaptic functional regulator FMR1	Fmr1
24.10949	23.55215	24.14102	E9QKK8;F6Q8D3;Q9QZW0	E9QKK8;F6Q8D3;Q9QZW	Phospholipid-transporting ATPase;Phosphol	Atp11c
24.72338	24.33756	24.47383	E9QMH7;Q9DBZ1	E9QMH7;Q9DBZ1	Inhibitor of nuclear factor kappa-B kinase-in	Ikbip
NaN	21.0426	NaN	E9QMQ3;Q99KW3	E9QMQ3;Q99KW3	TRIO and F-actin-binding protein	Triobp
NaN	NaN	NaN	E9QN31;Q922K7	E9QN31;Q922K7	Probable 28S rRNA (cytosine-C(5))-methyltra	Nop2
24.14102	NaN	23.21104	E9QQ96;Z4YJE4;H7BX05;A2AAJ9;A0A1V	E9QQ96;Z4YJE4;H7BX05;A	Obscurin	Obscn;O
23.49228	23.73892	23.32389	F2Z4B7;G5E867;F2Z3U3;F2Z3X7	F2Z4B7;G5E867;F2Z3U3;F	Ras association (RalGDS/AF-6) and pleckstri	Raph1
23.20658	NaN	NaN	F6QH25;E9Q4G8;E9Q3Q6;Q61490	F6QH25;E9Q4G8;E9Q3Q6	CD166 antigen (Fragment);CD166 antigen	Alcam
NaN	NaN	22.52612	F6R220;Q3TZM9	F6R220;Q3TZM9	GDP-Man:Man(3)GlcNAc(2)-PP-Dol alpha-1,	Alg11
23.68646	23.87643	NaN	F6RCU2;D3Z3M7;D3Z2Z1;F8WIA1;Q922	F6RCU2;D3Z3M7;D3Z2Z1;	CAP-Gly domain-containing linker protein 1	Clip1
NaN	23.94949	NaN	F6SD17;Q810S1	F6SD17;Q810S1	Calcium uniporter regulatory subunit MCUb	Mcub
25.1332	25.28912	25.21697	F6VF29;Q91VL0;G5E8U9;O54940;Q8BH	F6VF29;Q91VL0;G5E8U9;	BCL2/adenovirus E1B 19 kDa protein-intera	Bnip2
NaN	NaN	NaN	F6XC25;Q8BRN9	F6XC25;Q8BRN9	Coiled-coil and C2 domain-containing protei	Cc2d1b
22.43237	22.66058	22.82104	F6ZKZ3;Q9R112	F6ZKZ3;Q9R112	Sulfide:quinone oxidoreductase, mitochond	Sqor
23.28207	22.98815	23.18101	F7ARZ1;Q3TI14;Q8VE22;A7M7Q8	F7ARZ1;Q3TI14;Q8VE22;A	28S ribosomal protein S23, mitochondrial (F	Mrps23
NaN	21.79551	NaN	F8VPK0	F8VPK0	Tetratricopeptide repeat domain 37	Ttc37
22.11426	22.40925	NaN	F8VPX1;E9PXY8;Q6A4J8	F8VPX1;E9PXY8;Q6A4J8	Ubiquitin carboxyl-terminal hydrolase 7	Usp7
26.23535	26.21327	26.23169	F8WHP8;P56135	F8WHP8;P56135	ATP synthase subunit f, mitochondrial	Atp5j2
NaN	NaN	NaN	F8WHU5;Q4VA53	F8WHU5;Q4VA53	Sister chromatid cohesion protein PDS5 hom	Pds5b
23.37782	23.44253	23.18707	F8WI14;D3YV64;D3Z772	F8WI14	Extracellular matrix protein 1	Ecm1
27.4073	26.93157	27.3662	F8WID5;B7ZNL3	F8WID5;B7ZNL3	Tropomyosin alpha-1 chain;Tpm1 protein	Tpm1
25.59263	25.71293	25.64306	F8WJ43;P46662;F7DBY2	F8WJ43;P46662;F7DBY2	Merlin (Fragment);Merlin	Nf2
NaN	NaN	NaN	G3UVU2;D3Z5A6;D3YW09;Q62203	G3UVU2;D3Z5A6;D3YW09	Splicing factor 3A subunit 2;Splicing factor 3	Sf3a2

25.239	25.07316	25.1946	G3UW70;Q9D1L9	G3UW70;Q9D1L9	MCG21719;Ragulator complex protein LAMT	Lamtor5
25.79203	25.74919	26.06702	G3UYF9;Q03958	G3UYF9;Q03958	Prefoldin subunit 6	Pfdn6
NaN	NaN	NaN	G3UZJ3;G3UXK0;G3UY34;Q8K0N0;Q8C8	G3UZJ3;G3UXK0;G3UY34;(	Dystrobrevin beta;Dystrobrevin	Dtnb
NaN	NaN	NaN	G3X8U3	G3X8U3	MCG6895	2210016F16Rik
24.27498	24.54038	24.58692	G3X8W7;A0A0R4J1W7;Q8BGZ4	G3X8W7;A0A0R4J1W7;Q8	CDC23 (Cell division cycle 23, yeast, homolog	Cdc23
NaN	NaN	NaN	G3X8Y7;O88561;F7CPA3	G3X8Y7;O88561;F7CPA3	Long-chain fatty acid transport protein 3	Slc27a3
23.49228	23.40406	23.44253	G5E884	G5E884	Serine/threonine-protein kinase PAK 1	Pak1
NaN	NaN	NaN	G5E898;Q9R269	G5E898;Q9R269	Periplakin	Ppl
23.98468	24.18707	23.78955	G5E8C3;Q8BHL4	G5E8C3;Q8BHL4	G protein-coupled receptor, family C, group	Gprc5a
23.94056	24.33756	23.85757	G8JL35;E0CYJ0;E0CXA9;Q6PEB6	G8JL35;E0CYJ0;E0CXA9;Q6	MOB-like protein phocein (Fragment);MOB-	Mob4;
23.32389	NaN	NaN	G8JL76;D3Z636;Q91VM9	G8JL76;D3Z636;Q91VM9	Inorganic pyrophosphatase 2, mitochondrial	Ppa2
23.90426	24.02749	24.57542	H3BJ37;Q8BIG7	H3BJ37;Q8BIG7	Catechol O-methyltransferase domain-conta	Comtd1
24.21697	24.37782	25.48308	H3BKD4;H3BKE6;E9QMI7;E9QMJ1;H3BI	H3BKD4;H3BKE6;E9QMI7;	Arf-GAP with SH3 domain, ANK repeat and P	Asap1
NaN	NaN	NaN	H3BKW0;H3BJ30;H3BJW3;Q6NVF9	H3BKW0;H3BJ30;H3BJW3;	Cleavage and polyadenylation-specificity fac	Cpsf6
26.78955	26.49686	26.4008	H3BKY1;H9H9R4;Q9JHJ3	H3BKY1;H9H9R4;Q9JHJ3	Glycosylated lysosomal membrane protein	Glmp
24.18707	NaN	NaN	H7BWY2;Q9JKC8;D6RI63;D3YWU3;D3Y	H7BWY2;Q9JKC8;D6RI63;I	AP-3 complex subunit mu-1	Ap3m1
26.01265	25.96719	25.90426	H7BX38;P40224	H7BX38;P40224	Stromal cell-derived factor 1	Cxcl12
NaN	NaN	NaN	H7BX44;D3YYN8;E9PVY0;A0A1D5RLQ9;	H7BX44;D3YYN8;E9PVY0;	Serine/threonine-protein kinase MRCK alpha	Cdc42bpa
23.97596	23.46762	23.93157	J3QJX3;Q9Z2G6	J3QJX3;Q9Z2G6	Protein sel-1 homolog 1	Sel1l
23.79947	24.1332	23.72858	J3QN51;J3QPT3;B0LM42;F7BGR7;E9QL1	J3QN51;J3QPT3;B0LM42;F	MCG8382, isoform CRA_d;RNA-binding prot	Gm21992;Rbm1
23.49228	24.32389	23.79947	J3QN85;P16092;A0A0J9YTQ5;Q61563;C	J3QN85;P16092;A0A0J9YT	Fibroblast growth factor receptor;Fibroblast	Fgfr1
24.26069	24.49228	24.2021	J3QP56;P97823;J3QQ63;D3Z269;D3YUG	J3QP56;P97823;J3QQ63;D	Acyl-protein thioesterase 1	Lypla1
22.10789	22.21252	21.24191	K4DI77;Q5ND34;F6XD87	K4DI77;Q5ND34;F6XD87	WD repeat-containing protein 81 (Fragment	Wdr81
22.15808	22.41183	22.31838	M9MMK0;Q62177	M9MMK0;Q62177	Semaphorin-3B	Sema3b
23.23462	NaN	NaN	O08796	O08796	Eukaryotic elongation factor 2 kinase	Eef2k
24.12534	24.391	24.08537	O08848	O08848	60 kDa SS-A/Ro ribonucleoprotein	Trove2
NaN	NaN	NaN	O09174	O09174	Alpha-methylacyl-CoA racemase	Amacr
23.239	22.97771	23.01903	O35127	O35127	Protein C10	Grcc10
24.43619	24.26785	24.72858	O35405	O35405	Phospholipase D3	Pld3
NaN	NaN	NaN	O35465;F6WP10	O35465;F6WP10	Peptidyl-prolyl cis-trans isomerase FKBP8;Pe	Fkbp8
23.88577	24.88577	24.47383	O35704	O35704	Serine palmitoyltransferase 1	Sptlc1
23.68646	24.15653	24.06085	O35864;A0A087WQA8	O35864;A0A087WQA8	COP9 signalosome complex subunit 5;COP9	Cops5
25.00196	24.97596	25.02749	O55013	O55013	Trafficking protein particle complex subunit	Trappc3
NaN	NaN	NaN	O70421	O70421	Frizzled-1	Fzd1
23.40406	23.29614	23.23023	O88441	O88441	Metaxin-2	Mtx2
26.55215	26.61807	26.53445	P03930	P03930	ATP synthase protein 8	Mtatp8
23.60964	24.06907	23.60964	P04925	P04925	Major prion protein	Prnp
24.32389	24.51051	24.58692	P05132	P05132	cAMP-dependent protein kinase catalytic su	Prkaca
NaN	NaN	NaN	P05533	P05533	Lymphocyte antigen 6A-2/6E-1	Ly6a
25.1795	25.12139	25.41054	P09041	P09041	Phosphoglycerate kinase 2	Pgk2
22.95837	23.33756	23.55215	P09470;F6QCP8	P09470;F6QCP8	Angiotensin-converting enzyme;Angiotensin	Ace
23.93157	23.75939	23.88577	P14719;G3UYU3	P14719;G3UYU3	Interleukin-1 receptor-like 1;Interleukin-1 re	Il1rl1
26.2589	26.32903	NaN	P17665	P17665	Cytochrome c oxidase subunit 7C, mitochon	Cox7c
24.26069	24.18707	24.72858	P18155	P18155	Bifunctional methylenetetrahydrofolate deh	Mthfd2
NaN	NaN	NaN	P18608;D3Z607	P18608;D3Z607	Non-histone chromosomal protein HMG-14	Hmgn1
NaN	23.25061	23.35111	P22935	P22935	Cellular retinoic acid-binding protein 2	Crabp2
25.72858	26.2021	25.80931	P24604;Q8CFK4;Q3U436	P24604;Q8CFK4;Q3U436	Tyrosine-protein kinase Tec;Tyrosine-protein	Tec
NaN	25.68378	NaN	P26450;Q80UI5	P26450;Q80UI5	Phosphatidylinositol 3-kinase regulatory sub	Pik3r1
NaN	NaN	NaN	P27661	P27661	Histone H2AX	H2afx
24.52253	25.00196	25.02749	P28481	P28481	Collagen alpha-1(II) chain	Col2a1
24.77455	24.89966	25.04427	P29387;A0A0A6YVN9;V9GWY1	P29387	Guanine nucleotide-binding protein subunit	Gnb4
26.71293	26.33245	25.89735	P29595	P29595	NEDD8	Nedd8
NaN	NaN	22.14258	P32261;A0A0A6YXS8;A0A0A6YX49;A0A	P32261;A0A0A6YXS8;A0A	Antithrombin-III	Serpinc1
NaN	NaN	NaN	P37172	P37172	Activin receptor type-1	Acvr1
25.73635	25.32389	25.28912	P43277;I7HFT9;Q07133	P43277	Histone H1.3	Hist1h1d
25.34096	24.42982	24.44884	P43407	P43407	Syndecan-2	Sdc2
24.06907	23.60964	NaN	P46737	P46737	Lys-63-specific deubiquitinase BRCC36	Brcc3
23.31008	23.54038	22.96895	P47968	P47968	Ribose-5-phosphate isomerase	Rpia
25.57254	25.74919	25.86703	P48771	P48771	Cytochrome c oxidase subunit 7A2, mitocho	Cox7a2
22.96895	22.66492	23.96719	P49615;A0A0G2JDL3	P49615;A0A0G2JDL3	Cyclin-dependent-like kinase 5	Cdk5
24.05258	23.60964	22.75328	P51432	P51432	1-phosphatidylinositol 4,5-bisphosphate pho	Plcb3
22.82104	22.56848	NaN	P51670	P51670	C-C motif chemokine 9	Ccl9
24.67034	24.72338	24.72338	P52196	P52196	Thiosulfate sulfurtransferase	Tst
22.47011	21.79154	22.46014	P52795	P52795	Ephrin-B1	Efnb1
24.67034	25.68378	25.79947	P56379;A0A1Y7VLP0	P56379;A0A1Y7VLP0	6.8 kDa mitochondrial proteolipid;RIKEN cD	Mp68;2010107I
23.36453	23.49228	23.49228	P58196	P58196	Phospholipid scramblase 4	Plscr4
23.10789	22.29894	23.22729	P59279;Q3TEG7	P59279;Q3TEG7	Ras-related protein Rab-2B	Rab2b
24.26785	24.6971	24.85757	P61294;A0A1L1SRS6	P61294;A0A1L1SRS6	Ras-related protein Rab-6B	Rab6b
NaN	NaN	NaN	P61793	P61793	Lysophosphatidic acid receptor 1	Lpar1
24.90885	24.77455	24.83364	P62309	P62309	Small nuclear ribonucleoprotein G	Snrpg
23.57542	23.46762	23.62087	P62715	P62715	Serine/threonine-protein phosphatase 2A ca	Ppp2cb
24.58118	24.75939	24.59833	P62743;A0A0U1RPS0;A0A0U1RQ06	P62743;A0A0U1RPS0;A0A	AP-2 complex subunit sigma	Ap2s1
25.15267	24.93607	24.77455	P63213	P63213	Guanine nucleotide-binding protein G(I)/G(S	Gng2
25.39428	25.44884	25.29263	P70245;A2AC29	P70245;A2AC29	3-beta-hydroxysteroid-Delta(8),Delta(7)-isor	Ebp
NaN	23.92252	NaN	P70333	P70333	Heterogeneous nuclear ribonucleoprotein H	Hnrnph2
NaN	NaN	NaN	P70663	P70663	SPARC-like protein 1	Sparcl1
22.31562	22.96719	22.30173	P97363	P97363	Serine palmitoyltransferase 2	Sptlc2
22.01223	22.99335	23.32389	P97379	P97379	Ras GTPase-activating protein-binding prote	G3bp2
23.89504	NaN	23.17646	P97930;D3Z3R3	P97930;D3Z3R3	Thymidylate kinase	Dtymk
26.3662	26.03381	26.48001	P99028	P99028	Cytochrome b-c1 complex subunit 6, mitoch	Uqcrh
25.22803	25.64856	25.19084	Q00780	Q00780	Collagen alpha-1(VIII) chain	Col8a1
NaN	22.17341	22.91707	Q01514;A4UUI2	Q01514;A4UUI2	Guanylate-binding protein 1;Guanylate bind	Gbp1;Gbp2b
NaN	NaN	NaN	Q04646;A0A1L1SUA9;A0A1L1SQP8	Q04646;A0A1L1SUA9;A0A	Sodium/potassium-transporting ATPase sub	Fxyd2
NaN	NaN	NaN	Q148V8	Q148V8	Protein FAM83H	Fam83h
NaN	NaN	NaN	Q3KQM4;Q80XR5;P26369	Q3KQM4;Q80XR5;P26369	U2 snRNP auxiliary factor large subunit	U2af2
25.391	24.84805	25.88344	Q3SXD3	Q3SXD3	HD domain-containing protein 2	Hddc2
24.85281	24.74407	25.06085	Q3THE2;Q9CQ19	Q3THE2	Myosin regulatory light chain 12B	Myl12b
24.15653	23.92252	24.38443	Q3THG9;A2A4P3;A2A4P4	Q3THG9;A2A4P3;A2A4P4	Alanyl-tRNA editing protein Aarsd1;Predicte	Aarsd1;Gm2702

21.39624	NaN	NaN	Q3TIR3;A0A1B0GSI8	Q3TIR3;A0A1B0GSI8	Synembryn-A;Synembryn-A (Fragment)	Ric8a
23.12218	23.40406	23.5285	Q3TJ22;J3QN89	Q3TJ22;J3QN89	Angio-associated migratory protein	Aamp
23.01393	23.1519	23.12692	Q3TJZ6	Q3TJZ6	Protein FAM98A	Fam98a
24.21697	24.28912	24.74919	Q3TR20;A0A0R4J0Q9;Q3UM29	Q3TR20;A0A0R4J0Q9;Q3U	Conserved oligomeric Golgi complex subunit	Cog7
23.31008	NaN	23.84805	Q3TSA8;Q8K021;D3YTP4	Q3TSA8;Q8K021;D3YTP4	Secretory carrier-associated membrane prot	Scamp1
23.1961	23.05755	23.71816	Q3TYX3	Q3TYX3	SET and MYND domain-containing protein 5	Smyd5
NaN	NaN	NaN	Q3U3G8;Q9EPB5	Q3U3G8;Q9EPB5	Serine hydrolase-like protein	Serhl
23.1379	22.91342	22.6231	Q3UE37	Q3UE37	Ubiquitin-conjugating enzyme E2 Z	Ube2z
NaN	NaN	NaN	Q3UEB4;Q9R008;A0A0G2JEG0;A0A0G2	Q3UEB4;Q9R008;A0A0G2	Mevalonate kinase	Mvk
23.62087	23.56384	23.48001	Q3UF75;Q9EPC1	Q3UF75;Q9EPC1	Alpha-parvin	Parva
22.48247	22.66709	22.34028	Q3UGB5;Q9JII5;D3Z4J1	Q3UGB5;Q9JII5;D3Z4J1	DAZ associated protein 1, isoform CRA_b;DA	Dazap1
NaN	23.16576	22.84613	Q3UGF1	Q3UGF1	WD repeat-containing protein 19	Wdr19
24.63201	24.67034	24.74919	Q3UGN9;O88811;P70297;B0R0N7;A2AF	Q3UGN9;O88811;P70297;	Signal transducing adapter molecule 1;Signa	Stam;Stam2
24.26069	24.65403	24.2021	Q3UHX2	Q3UHX2	28 kDa heat- and acid-stable phosphoprotei	Pdap1
22.09507	NaN	NaN	Q3UKJ7	Q3UKJ7	WD40 repeat-containing protein SMU1	Smu1
24.36453	25.18707	23.81909	Q3UN10;P56695	Q3UN10;P56695	Wolframin	Wfs1
NaN	NaN	NaN	Q3UPH1	Q3UPH1	Protein PRRC1	Prrc1
23.75939	23.36453	NaN	Q3UPZ0;Q62217	Q3UPZ0;Q62217	Sema domain, seven thrombospondin repea	Sema5a
NaN	NaN	NaN	Q3UTR7;P11859	Q3UTR7;P11859	Angiotensinogen	Agt
NaN	23.68646	23.80931	Q4FCQ7;J3QNU1;A0A1L1SQ41;Q9Z2A5;	Q4FCQ7;J3QNU1;A0A1L1S	Arginyl-tRNA--protein transferase 1	Ate1
28.41135	28.61456	28.509	Q4LFA9	Q4LFA9	Semaphorin-3G	Sema3g
NaN	NaN	NaN	Q505D1	Q505D1	Serine/threonine-protein phosphatase 6 reg	Ankrd28
23.63201	23.64306	23.94056	Q5F2E8;F6RXB5;A0A0R4J1T3;Q8BYC6	Q5F2E8;F6RXB5;A0A0R4J1	Serine/threonine-protein kinase TAO1;Serin	Taok1;Taok3
NaN	NaN	NaN	Q5SPX8;P58802	Q5SPX8;P58802	TBC1 domain family member 10A	Tbc1d10a
23.70767	23.68646	23.74919	Q5SSZ5	Q5SSZ5	Tensin-3	Tns3
22.14569	NaN	22.61189	Q5SUH7;Q5SUH6;Q99KN9	Q5SUH7;Q5SUH6;Q99KN9	Clathrin interactor 1	Clint1
23.09023	23.26785	23.42982	Q5SUT0;Q5SUS9;Q61545	Q5SUT0;Q5SUS9;Q61545	RNA-binding protein EWS	Ewsr1
23.76951	23.73892	23.91342	Q5SVG5;Q5SVG4;O35643	Q5SVG5;Q5SVG4;O35643	AP complex subunit beta;AP-1 complex subu	Ap1b1
23.79947	23.85757	23.86703	Q5SX40;B1AR69;G3UW82;Q5SX39;P135	Q5SX40;B1AR69;G3UW82	Myosin-1;Myosin, heavy polypeptide 13, ske	Myh1;Myh13;M
23.86703	23.44253	23.62087	Q5SXA5;Q5SXA4;Q5SRX1	Q5SXA5;Q5SXA4;Q5SRX1	TOM1-like protein 2	Tom1l2
24.63201	24.49838	24.28912	Q5U4H9;E9Q0D2;E9Q2H3	Q5U4H9;E9Q0D2;E9Q2H3	Nuclear receptor coactivator 4;Nuclear rece	Ncoa4
23.51653	23.49228	23.02411	Q5XKN4;A0A0N4SV89	Q5XKN4;A0A0N4SV89	Protein jagunal homolog 1	Jagn1
25.23169	26.1546	25.06907	Q60772	Q60772	Cyclin-dependent kinase 4 inhibitor C	Cdkn2c
24.62087	25.12534	25.1757	Q60960;G3UXF0	Q60960;G3UXF0	Importin subunit alpha-5;Importin subunit a	Kpna1
23.57542	23.66492	23.72858	Q60972	Q60972	Histone-binding protein RBBP4	Rbbp4
27.71947	27.41538	27.43936	Q61735	Q61735	Leukocyte surface antigen CD47	Cd47
22.96895	23.54038	23.21401	Q62011;A8Y5F6	Q62011;A8Y5F6	Podoplanin	Pdpn
24.33756	24.31008	24.38443	Q62074;F6QWV4	Q62074;F6QWV4	Protein kinase C iota type;Protein kinase C i	Prkci
24.54628	25.04843	NaN	Q62266	Q62266	Cornifin-A	Sprr1a
22.54981	22.31008	22.74919	Q62273;E9PZ06;F8WI08	Q62273;E9PZ06;F8WI08	Sulfate transporter;Sulfate transporter (Frag	Slc26a2
25.1488	24.78457	25.05672	Q63810	Q63810	Calcineurin subunit B type 1	Ppp3r1
24.391	24.28207	24.67034	Q63850	Q63850	Nuclear pore glycoprotein p62	Nup62
23.97596	23.57542	23.391	Q63ZW6;F7CK55	Q63ZW6;F7CK55	Col4a5 protein;Collagen, type IV, alpha 5 (Fr	Col4a5
23.02918	23.37782	23.99335	Q64261;A0A0G2JGA8	Q64261	Cyclin-dependent kinase 6	Cdk6
26.63201	26.49381	26.05879	Q642K5;P62862	Q642K5;P62862	40S ribosomal protein S30	Fau
23.76951	NaN	NaN	Q64345;E9PV48	Q64345;E9PV48	Interferon-induced protein with tetratricope	Ifit3;Ifit3b
NaN	NaN	NaN	Q64429	Q64429	Cytochrome P450 1B1	Cyp1b1
NaN	23.5285	23.16883	Q64437	Q64437	Alcohol dehydrogenase class 4 mu/sigma ch	Adh7
NaN	NaN	NaN	Q64695;A2AUV5	Q64695;A2AUV5	Endothelial protein C receptor;Endothelial p	Procr
21.66709	NaN	NaN	Q69ZF7;Q3TWN3	Q69ZF7;Q3TWN3	Metal transporter CNNM4;Metal transporte	Cnnm4;Cnnm2
NaN	NaN	22.39885	Q6KAU4	Q6KAU4	Multivesicular body subunit 12B	Mvb12b
25.10149	24.6971	24.32389	Q6XLQ8	Q6XLQ8	Calumenin	Calu
22.91525	22.89874	23.49228	Q6ZPE2;F7AWK3;D6RG52	Q6ZPE2;F7AWK3;D6RG52	Myotubularin-related protein 5;Myotubulari	Sbf1
NaN	22.75124	22.74919	Q76N33	Q76N33	AMSH-like protease	Stambpl1
24.91798	25.00624	24.92705	Q78IK2	Q78IK2	Up-regulated during skeletal muscle growth	Usmg5
22.82687	22.5545	22.96543	Q78IS1	Q78IS1	Transmembrane emp24 domain-containing	Tmed3
28.16997	28.20303	28.29351	Q7M6W1;A3QM89;Q8K0T0	Q7M6W1;A3QM89;Q8K0T	Reticulon;Reticulon (Fragment);Reticulon-1	Rtn1
NaN	NaN	24.72858	Q7TS64;Q99MK8;F7AEX1;F6Y9P3;F6QY	Q7TS64;Q99MK8;F7AEX1;	G protein-coupled receptor kinase;Beta-adre	Grk2
NaN	22.87267	NaN	Q80TA1;D6RE20;Q8CET7	Q80TA1;D6RE20;Q8CET7	Ethanolaminephosphotransferase 1;DNA seg	Selenoi
21.66275	21.40665	21.8423	Q80TA9	Q80TA9	Ectopic P granules protein 5 homolog	Epg5
NaN	NaN	21.64966	Q80U95;A0A0G2JDR9	Q80U95;A0A0G2JDR9	Ubiquitin-protein ligase E3C	Ube3c
24.17188	24.11744	23.82881	Q80UM7	Q80UM7	Mannosyl-oligosaccharide glucosidase	Mogs
23.80931	23.51653	23.33756	Q80UY2;A0A0U1RNG8;A0A0N4SV15	Q80UY2;A0A0U1RNG8;A0	E3 ubiquitin-protein ligase KCMF1	Kcmf1
NaN	22.17035	22.94414	Q80VD1	Q80VD1	Protein FAM98B	Fam98b
23.03591	22.58463	22.65622	Q80W68	Q80W68	Kin of IRRE-like protein 1	Kirrel1
NaN	22.60286	23.09507	Q80WQ6	Q80WQ6	Inactive rhomboid protein 2	Rhbdf2
NaN	22.95305	23.49228	Q80XI4;F6RJE8;O70172	Q80XI4;F6RJE8;O70172	Phosphatidylinositol 5-phosphate 4-kinase t	Pip4k2b;Pip4k2
24.07725	24.74407	24.33074	Q80Y14;A0A1Y7VN70	Q80Y14;A0A1Y7VN70	Glutaredoxin-related protein 5, mitochondri	Glrx5
27.09144	25.56674	25.66221	Q80Y44	Q80Y44	Probable ATP-dependent RNA helicase DDX1	Ddx10
24.42342	23.77957	24.02749	Q80ZX0;F6VJC5	Q80ZX0;F6VJC5	Sec24-related gene family, member B (S. cer	Sec24b
NaN	NaN	NaN	Q8BGT5	Q8BGT5	Alanine aminotransferase 2	Gpt2
24.46139	24.8811	23.91342	Q8BH24	Q8BH24	Transmembrane 9 superfamily member 4	Tm9sf4
NaN	NaN	24.62645	Q8BK72	Q8BK72	28S ribosomal protein S27, mitochondrial	Mrps27
24.54628	24.87173	NaN	Q8BND3	Q8BND3	WD repeat-containing protein 35	Wdr35
NaN	NaN	NaN	Q8BPM0	Q8BPM0	Disheveled-associated activator of morphog	Daam1
23.60964	22.55918	22.42726	Q8BTU6;P10630;E9Q561	Q8BTU6;P10630;E9Q561	Eukaryotic initiation factor 4A-II	Eif4a2
25.77957	25.56964	25.77706	Q8BU31;Q80ZJ1	Q8BU31;Q80ZJ1	Ras-related protein Rap-2c;Ras-related prote	Rap2c;Rap2a
24.27498	24.02749	NaN	Q8BVQ5;A0A140LI84	Q8BVQ5;A0A140LI84	Protein phosphatase methylesterase 1;Prote	Ppme1
NaN	NaN	NaN	Q8BX70;A0A1L1SS63	Q8BX70;A0A1L1SS63	Vacuolar protein sorting-associated protein	Vps13c
NaN	NaN	NaN	Q8BXV2	Q8BXV2	BRI3-binding protein	Bri3bp
22.03255	21.29894	21.70134	Q8BZW8	Q8BZW8	NHL repeat-containing protein 2	Nhlrc2
NaN	24.87643	24.69179	Q8C048;A2ALL9;Q0VF55;S4R1C4	Q8C048;A2ALL9;Q0VF55;S	Calcium-transporting ATPase	Atp2b3
24.62645	24.61526	24.417	Q8C5G6;Q9QZ06;A9JEI5;F7AT44	Q8C5G6;Q9QZ06;A9JEI5;F	Toll-interacting protein;Toll interacting prote	Tollip;Toll
24.48616	24.74919	25.1332	Q8C6U2;Q3UU83;A0A1Y7VLY9	Q8C6U2;Q3UU83;A0A1Y7	PQ-loop repeat-containing protein 3	Pqlc3
NaN	20.37518	NaN	Q8CCF0	Q8CCF0	U4/U6 small nuclear ribonucleoprotein Prp3	Prpf31
27.05051	26.5285	26.69975	Q8CFU8;G3UXZ2	Q8CFU8;G3UXZ2	Tumor protein p53-inducible nuclear protein	Tp53inp2;Trp53
NaN	NaN	NaN	Q8CG70;D6RHQ3	Q8CG70;D6RHQ3	Prolyl 3-hydroxylase 3	P3h3
NaN	24.46762	NaN	Q8CG76	Q8CG76	Aflatoxin B1 aldehyde reductase member 2	Akr7a2

NaN	NaN	NaN	Q8CGC6	Q8CGC6	RNA-binding protein 28	Rbm28
NaN	NaN	NaN	Q8CHK3	Q8CHK3	Lysophospholipid acyltransferase 7	Mboat7
23.66492	23.67573	22.87455	Q8CHP8	Q8CHP8	Glycerol-3-phosphate phosphatase	Pgp
23.32389	23.54038	23.391	Q8CIH9	Q8CIH9	Amidophosphoribosyltransferase	Ppat
24.33074	24.43619	24.42982	Q8CJ40	Q8CJ40	Rootletin	Crocc
22.77957	22.51171	22.58921	Q8K0D5	Q8K0D5	Elongation factor G, mitochondrial	Gfm1
24.81421	24.93157	24.79452	Q8K183	Q8K183	Pyridoxal kinase	Pdxk
NaN	NaN	NaN	Q8K224	Q8K224	RNA cytidine acetyltransferase	Nat10
NaN	NaN	NaN	Q8K363;F6ZX28	Q8K363;F6ZX28	ATP-dependent RNA helicase DDX18;ATP-de	Ddx18
23.19009	23.08699	23.2535	Q8K3H0	Q8K3H0	DCC-interacting protein 13-alpha	Appl1
24.04427	24.02749	24.06907	Q8K3J1	Q8K3J1	NADH dehydrogenase [ubiquinone] iron-sulf	Ndufs8
NaN	NaN	NaN	Q8K4Z5	Q8K4Z5	Splicing factor 3A subunit 1	Sf3a1
23.23608	23.56384	23.70767	Q8N7N5	Q8N7N5	DDB1- and CUL4-associated factor 8	Dcaf8
NaN	NaN	NaN	Q8QZY9	Q8QZY9	Splicing factor 3B subunit 4	Sf3b4
22.40406	22.67788	NaN	Q8R0G9	Q8R0G9	Nuclear pore complex protein Nup133	Nup133
NaN	NaN	NaN	Q8R116;B7ZCA6	Q8R116;B7ZCA6	Palmitoleoyl-protein carboxylesterase NOTU	Notum
23.0258	23.21401	23.71816	Q8R127;J3QNN5;F6S0R8	Q8R127;J3QNN5;F6S0R8	Saccharopine dehydrogenase-like oxidoredu	Sccpdh
26.07725	26.13516	26.1795	Q8R1I1	Q8R1I1	Cytochrome b-c1 complex subunit 9	Uqcr10
21.2764	21.43746	19.74222	Q8R1N4;A0A0A0MQL8	Q8R1N4;A0A0A0MQL8	NudC domain-containing protein 3;NudC do	Nudcd3
NaN	NaN	NaN	Q8R1V4;Q5SVW9	Q8R1V4;Q5SVW9	Transmembrane emp24 domain-containing	Tmed4
NaN	NaN	NaN	Q8R2G6	Q8R2G6	Coiled-coil domain-containing protein 80	Ccdc80
23.46762	23.68646	23.417	Q8R2U6;P0C028;P0C027	Q8R2U6;P0C028;P0C027	Diphosphoinositol polyphosphate phosphoh	Nudt4;Nudt11
22.15808	22.01563	22.0258	Q8R317	Q8R317	Ubiquilin-1	Ubqln1
NaN	NaN	NaN	Q8R323	Q8R323	Replication factor C subunit 3	Rfc3
22.97596	NaN	22.64526	Q8R349	Q8R349	Cell division cycle protein 16 homolog	Cdc16
29.65438	NaN	29.71129	Q8R480	Q8R480	Nuclear pore complex protein Nup85	Nup85
23.91342	24.67034	24.49228	Q8VCB1;J3QPB5	Q8VCB1;J3QPB5	Nucleoporin NDC1	Ndc1
NaN	NaN	NaN	Q8VCF1	Q8VCF1	Soluble calcium-activated nucleotidase 1	Cant1
24.58692	24.54038	24.20955	Q8VCR4;Q9DCG9	Q8VCR4;Q9DCG9	0610038D11Rik protein;Multifunctional met	Trmt112
NaN	NaN	NaN	Q8VCW8	Q8VCW8	Acyl-CoA synthetase family member 2, mitoc	Acsf2
25.19084	25.50446	25.43936	Q8VD65	Q8VD65	Phosphoinositide 3-kinase regulatory subuni	Pik3r4
NaN	NaN	NaN	Q8VDD8	Q8VDD8	WASH complex subunit 1	Washc1
NaN	NaN	NaN	Q8VDP4	Q8VDP4	Cell cycle and apoptosis regulator protein 2	Ccar2
24.44253	24.50446	24.30313	Q8VDZ4	Q8VDZ4	Palmitoyltransferase ZDHHC5	Zdhhc5
21.92071	21.77153	21.8423	Q8VE88	Q8VE88	Protein FAM114A2	Fam114a2
24.84805	24.94056	24.17188	Q8VE91	Q8VE91	Reticulophagy regulator 1	Retreg1d
22.74098	23.46762	23.50446	Q8VE98;A0A1L1SUD4	Q8VE98;A0A1L1SUD4	CD276 antigen;CD276 antigen (Fragment)	Cd276
NaN	NaN	NaN	Q8VHB5;F6XXU0	Q8VHB5;F6XXU0	Carbonic anhydrase 9;Carbonic anhydrase 9	Ca9;Car9
24.99766	26.22066	25.05258	Q8VHE6	Q8VHE6	Dynein heavy chain 5, axonemal	Dnah5
24.1332	23.45513	24.63201	Q91V64	Q91V64	Isochorismatase domain-containing protein	Isoc1
26.34773	26.23535	26.1035	Q91VR7	Q91VR7	Microtubule-associated proteins 1A/1B light	Map1lc3a
25.96279	NaN	25.92705	Q91W18;F6ZI20;E9Q6T6	Q91W18;F6ZI20;E9Q6T6	Tudor domain-containing protein 3;Tudor do	Tdrd3
NaN	NaN	21.87267	Q91WR3	Q91WR3	Activating signal cointegrator 1 complex sub	Ascc2
22.44	22.41442	NaN	Q91XD6	Q91XD6	Vacuolar protein-sorting-associated protein	Vps36
25.11347	25.11347	24.36453	Q91XX1;A0A0A6YXZ9;A0A0A6YVS5;Q91	Q91XX1;A0A0A6YXZ9;A0A	Protocadherin gamma C3;Protocadherin gan	Pcdhgc3;Pcdhgc
NaN	21.58692	21.58692	Q91YR5	Q91YR5	Methyltransferase-like protein 13	Mettl13
22.11426	21.44758	20.96719	Q91YR7	Q91YR7	Pre-mRNA-processing factor 6	Prpf6
24.83364	24.63201	24.80931	Q91YS8;D3Z368	Q91YS8;D3Z368	Calcium/calmodulin-dependent protein kina	Camk1
26.58978	26.39264	26.87408	Q920Q2	Q920Q2	DNA repair protein REV1	Rev1
23.09507	22.93157	NaN	Q921I1;D3YYR8	Q921I1	Serotransferrin	Tf
21.6584	NaN	NaN	Q921I2;G3UZG5;G3UZ63;G3X961	Q921I2;G3UZG5;G3UZ63;(	Kelch domain-containing protein 4	Klhdc4
23.14102	22.77756	23.2535	Q921U7;Q8C281;Q8CE04;Q8CE80;P511	Q921U7;Q8C281;Q8CE04;	Calpastatin	Cast
23.75939	23.72858	24.03591	Q922H1	Q922H1	Protein arginine N-methyltransferase 3	Prmt3
NaN	NaN	NaN	Q99J23	Q99J23	GH3 domain-containing protein	Ghdc
25.1488	25.35448	25.50142	Q99J56	Q99J56	Derlin-1	Derl1
23.82881	NaN	23.37782	Q99J87	Q99J87	Probable ATP-dependent RNA helicase DHX5	Dhx58
NaN	23.35111	23.24191	Q99LB6;E0CYU5	Q99LB6;E0CYU5	Methionine adenosyltransferase 2 subunit b	Mat2b
22.84613	22.99852	23.24916	Q99LC8;A0A0G2JEF1;D3YZ05;D3Z151	Q99LC8;A0A0G2JEF1;D3YZ	Translation initiation factor eIF-2B subunit a	Eif2b1
24.72858	NaN	24.06907	Q99LT0	Q99LT0	Protein dpy-30 homolog	Dpy30
NaN	NaN	NaN	Q99M20	Q99M20	Kallikrein j	Klk10
23.31008	23.26785	21.96367	Q99M87	Q99M87	DnaJ homolog subfamily A member 3, mitoc	Dnaja3
22.17035	NaN	22.23023	Q99MR3	Q99MR3	Solute carrier family 12 member 9	Slc12a9
22.7348	22.56384	22.71398	Q9CPX6	Q9CPX6	Ubiquitin-like-conjugating enzyme ATG3	Atg3
24.14102	24.36453	24.35111	Q9CQ75	Q9CQ75	NADH dehydrogenase [ubiquinone] 1 alpha s	Ndufa2
22.35917	22.69498	22.08537	Q9CQA1	Q9CQA1	Trafficking protein particle complex subunit	Trappc5
25.04843	24.78955	25.33415	Q9CQH7	Q9CQH7	Transcription factor BTF3 homolog 4	Btf3l4
NaN	NaN	NaN	Q9CQI3;D3YY93	Q9CQI3;D3YY93	Glia maturation factor beta;Glia maturation	Gmfb
24.90426	25.81176	22.96014	Q9CQM2	Q9CQM2	ER lumen protein-retaining receptor 2	Kdelr2
24.2535	24.10949	23.99335	Q9CQN6	Q9CQN6	Transmembrane protein 14C	Tmem14c
24.82881	24.63201	24.09346	Q9CQR4	Q9CQR4	Acyl-coenzyme A thioesterase 13	Acot13
25.21697	25.28912	25.45199	Q9CQS8;E9PW43	Q9CQS8;E9PW43	Protein transport protein Sec61 subunit beta	Sec61b;Gm1032
23.96719	24.35783	24.02749	Q9CQU3	Q9CQU3	Protein RER1	Rer1
NaN	22.01563	22.48247	Q9CQY1	Q9CQY1	Ubiquitin-like protein ATG12	Atg12
23.2535	23.1946	NaN	Q9CR41	Q9CR41	Huntingtin-interacting protein K	Hypk
23.75939	24.391	24.78457	Q9CR60	Q9CR60	Vesicle transport protein GOT1B	Golt1b
24.69179	24.05258	24.07725	Q9CR86	Q9CR86	Calcium-regulated heat stable protein 1	Carhsp1
25.80193	26.08537	25.28912	Q9CRC0	Q9CRC0	Vitamin K epoxide reductase complex subun	Vkorc1
24.57542	24.7543	24.57542	Q9CRD2	Q9CRD2	ER membrane protein complex subunit 2	Emc2
22.75735	24.7543	NaN	Q9CWU9	Q9CWU9	Nucleoporin Nup37	Nup37
NaN	NaN	NaN	Q9CXP4	Q9CXP4	Rho GTPase-activating protein 8	Arhgap8
NaN	NaN	NaN	Q9CXT7;A0A1B0GS44	Q9CXT7;A0A1B0GS44	Transmembrane protein 192	Tmem192
23.35111	23.23023	22.92976	Q9CXY6	Q9CXY6	Interleukin enhancer-binding factor 2	Ilf2
22.91342	23.04593	22.92976	Q9CZH3	Q9CZH3	Proteasome assembly chaperone 3	Psmg3
NaN	NaN	NaN	Q9D0F6;D3Z1Y6	Q9D0F6;D3Z1Y6	Replication factor C subunit 5	Rfc5
NaN	NaN	24.26069	Q9D0J4	Q9D0J4	ADP-ribosylation factor-like protein 2	Arl2
25.69975	25.67573	25.60399	Q9D0J8	Q9D0J8	Parathymosin	Ptms
23.76951	23.79947	22.99852	Q9D0M5;D6RIN4	Q9D0M5	Dynein light chain 2, cytoplasmic	Dynll2
23.55215	NaN	23.58692	Q9D0P8	Q9D0P8	Intraflagellar transport protein 27 homolog	Ift27

24.26785	23.99335	24.1332	Q9D0S9	Q9D0S9	Histidine triad nucleotide-binding protein 2,	Hint2
24.28207	23.83846	23.31008	Q9D0W5	Q9D0W5	Peptidyl-prolyl cis-trans isomerase-like 1	Ppil1
24.97596	24.60399	25.11744	Q9D187;A0A1D5RMI0;F8WHV1	Q9D187;A0A1D5RMI0;F8W	Mitotic spindle-associated MMXD complex s	Fam96b
NaN	NaN	NaN	Q9D1E8;F8WGD9	Q9D1E8;F8WGD9	1-acyl-sn-glycerol-3-phosphate acyltransfera	Agpat5
NaN	NaN	NaN	Q9D1J3;A0A1W2P7G2;A0A1W2P6N2	Q9D1J3;A0A1W2P7G2;A0	SAP domain-containing ribonucleoprotein	Sarnp
24.90885	24.98902	24.85757	Q9D1T2;Q9CZH7	Q9D1T2;Q9CZH7	Matrix-remodeling-associated protein 7	Mxra7
29.01903	28.97869	28.97323	Q9D305	Q9D305	THAP domain-containing protein 2	Thap2
27.67303	27.60399	27.50142	Q9D3D9	Q9D3D9	ATP synthase subunit delta, mitochondrial	Atp5d
NaN	NaN	24.35111	Q9D4H1	Q9D4H1	Exocyst complex component 2	Exoc2
22.64306	22.4996	NaN	Q9D5T0	Q9D5T0	ATPase family AAA domain-containing prote	Atad1
24.8044	24.90426	24.56964	Q9D6J6	Q9D6J6	NADH dehydrogenase [ubiquinone] flavopro	Ndufv2
21.94235	22.20658	21.74714	Q9D771	Q9D771	Transmembrane protein 206	Tmem206
NaN	NaN	24.35111	Q9D7B7	Q9D7B7	Probable glutathione peroxidase 8	Gpx8
22.46513	23.391	NaN	Q9D832	Q9D832	DnaJ homolog subfamily B member 4	Dnajb4
NaN	23.26785	23.18858	Q9D892	Q9D892	Inosine triphosphate pyrophosphatase	Itpa
22.70134	22.30452	22.50688	Q9D8N2;D6RIQ6;D3YUG1;Q3TH34	Q9D8N2;D6RIQ6;D3YUG1	Protein FAM45A;Protein FAM45A (Fragment	Fam45a
NaN	NaN	NaN	Q9D8S5;O35326	Q9D8S5;O35326	MCG7614, isoform CRA_c;Serine/arginine-ri	Srsf5
22.90426	23.36453	23.48001	Q9DB07	Q9DB07	Intraflagellar transport protein 46 homolog	Ift46
23.92252	23.92252	22.75124	Q9DB10;E0CZF0	Q9DB10;E0CZF0	Essential MCU regulator, mitochondrial	Smdt1
23.04093	23.00881	23.6971	Q9DBL7	Q9DBL7	Bifunctional coenzyme A synthase	Coasy
NaN	NaN	NaN	Q9DC29	Q9DC29	ATP-binding cassette sub-family B member 6	Abcb6
25.35448	25.46762	25.03171	Q9DC37	Q9DC37	Major facilitator superfamily domain-contai	Mfsd1
NaN	NaN	NaN	Q9DC42;A0A0D9SEG8;E9QMJ5;E9QJS7;	Q9DC42;A0A0D9SEG8;E9	Adhesion G protein-coupled receptor E5	Adgre5
24.01052	22.47754	23.67573	Q9DC70	Q9DC70	NADH dehydrogenase [ubiquinone] iron-sulf	Ndufs7
21.96719	22.29053	22.239	Q9DCE6;E0CXT5;E9PX84;Q3TRE6;E9QL3	Q9DCE6;E0CXT5;E9PX84;Q	Disabled homolog 2	Dab2
23.87643	23.5285	23.72858	Q9DCJ5	Q9DCJ5	NADH dehydrogenase [ubiquinone] 1 alpha	Ndufa8
23.42982	23.29614	23.5285	Q9DCS3;A2A845	Q9DCS3;A2A845	Enoyl-[acyl-carrier-protein] reductase, mitoc	Mecr
NaN	22.48738	NaN	Q9EQI8	Q9EQI8	39S ribosomal protein L46, mitochondrial	Mrpl46
23.63201	23.60964	23.63201	Q9ERS2	Q9ERS2	NADH dehydrogenase [ubiquinone] 1 alpha	Ndufa13
NaN	23.36453	NaN	Q9ESP1	Q9ESP1	Stromal cell-derived factor 2-like protein 1	Sdf2l1
NaN	NaN	NaN	Q9EST4;REV__A0A0A6YWX5	Q9EST4;REV__A0A0A6YW	Proteasome assembly chaperone 2;Voltage-	Psmg2;Cacnb2
NaN	NaN	NaN	Q9ET22	Q9ET22	Dipeptidyl peptidase 2	Dpp7
22.3073	23.29614	NaN	Q9JIP6	Q9JIP6	Frizzled-2	Fzd2
NaN	NaN	NaN	Q9JJA4;D3Z369	Q9JJA4;D3Z369	Ribosome biogenesis protein WDR12;Ribosc	Wdr12
24.73892	25.16806	25.09748	Q9JJF9	Q9JJF9	Signal peptide peptidase-like 2A	Sppl2a
23.94949	24.28912	24.04427	Q9JKN1	Q9JKN1	Zinc transporter 7	Slc30a7
27.93719	27.85638	27.99226	Q9JKW0;A0A0U1RPY6	Q9JKW0;A0A0U1RPY6	ADP-ribosylation factor-like protein 6-intera	Arl6ip1
NaN	NaN	NaN	Q9JM51	Q9JM51	Prostaglandin E synthase	Ptges
NaN	NaN	NaN	Q9JMK0	Q9JMK0	Beta-1,4-galactosyltransferase 5	B4galt5
NaN	NaN	NaN	Q9QXK7;A0A1Y7VIZ9;A0A1Y7VJ97;A0A1	Q9QXK7;A0A1Y7VIZ9;A0A	Cleavage and polyadenylation specificity fac	Cpsf3
NaN	NaN	NaN	Q9QZC7	Q9QZC7	Pleckstrin homology domain-containing fam	Plekhb2
22.01903	22.48247	22.33756	Q9QZD8	Q9QZD8	Mitochondrial dicarboxylate carrier	Slc25a10
24.65403	24.84326	24.41054	Q9QZI9	Q9QZI9	Serine incorporator 3	Serinc3
24.91798	24.56384	NaN	Q9QZM0	Q9QZM0	Ubiquilin-2	Ubqln2
24.32389	24.42982	25.01903	Q9QZM4	Q9QZM4	Tumor necrosis factor receptor superfamily	Tnfrsf10b
NaN	23.1991	NaN	Q9R078;S4R2R1	Q9R078;S4R2R1	5-AMP-activated protein kinase subunit beta	Prkab1
NaN	NaN	22.86325	Q9R099	Q9R099	Transducin beta-like protein 2	Tbl2
25.26069	25.03171	25.16423	Q9R1Q6;A0A0N4SUM7;A0A0N4SUY1;A0	Q9R1Q6;A0A0N4SUM7;A0	Transmembrane protein 176B;Transmembra	Tmem176b
28.27142	28.51653	28.74855	Q9R1Q7	Q9R1Q7	Proteolipid protein 2	Plp2
NaN	NaN	20.81127	Q9R1X5	Q9R1X5	Multidrug resistance-associated protein 5	Abcc5
NaN	NaN	NaN	Q9WTR6;A0A0B4J1P7	Q9WTR6;A0A0B4J1P7	Cystine/glutamate transporter	Slc7a11
NaN	NaN	23.417	Q9WUN2;A0A1W2P835	Q9WUN2;A0A1W2P835	Serine/threonine-protein kinase TBK1;Serine	Tbk1
24.79947	24.91342	25.16039	Q9WV27	Q9WV27	Sodium/potassium-transporting ATPase sub	Atp1a4
23.50446	23.66492	23.36453	Q9WV95	Q9WV95	Pleckstrin homology-like domain family A m	Phlda3
25.74407	26.10149	26.05466	Q9WV98;A0A1Y7VL11;A0A1Y7VIZ6	Q9WV98;A0A1Y7VL11;A0	Mitochondrial import inner membrane trans	Timm9
23.57542	23.417	23.58692	Q9WVA2;Q4FZG7	Q9WVA2;Q4FZG7	Mitochondrial import inner membrane trans	Timm8a1;Timm
24.52253	24.01052	24.44884	Q9WVC3	Q9WVC3	Caveolin-2	Cav2
24.14102	23.78955	23.85757	Q9Z0G0	Q9Z0G0	PDZ domain-containing protein GIPC1	Gipc1
25.04009	24.95837	25.01478	Q9Z0L8	Q9Z0L8	Gamma-glutamyl hydrolase	Ggh
NaN	NaN	NaN	Q9Z0P4;Q3UZP7;A0A1W2P831	Q9Z0P4;Q3UZP7;A0A1W2	Paralemmin-1;Paralemmin-1 (Fragment)	Palm
24.53445	24.56964	24.58118	Q9Z160	Q9Z160	Conserved oligomeric Golgi complex subunit	Cog1
23.18707	23.1916	23.28207	Q9Z179;A0A140LHA7	Q9Z179;A0A140LHA7	SHC SH2 domain-binding protein 1;SHC SH2	Shcbp1
24.14102	24.32389	24.07725	Q9Z1A1;B8JJG9;B8JJG7;B8JJG8;B8JJG6;F	Q9Z1A1;B8JJG9;B8JJG7;B8	TFG protein;Trk-fused gene (Fragment)	Tfg
22.86703	22.95305	23.16423	Q9Z1W9	Q9Z1W9	STE20/SPS1-related proline-alanine-rich pro	Stk39
24.17188	24.1332	23.46762	Q9Z247	Q9Z247	Peptidyl-prolyl cis-trans isomerase FKBP9	Fkbp9
23.0258	NaN	NaN	Q9Z2L6	Q9Z2L6	Multiple inositol polyphosphate phosphatas	Minpp1
NaN	23.57542	23.64306	Q9Z2N8;D3YVN1;A0A0A6YWG8	Q9Z2N8;D3YVN1;A0A0A6	Actin-like protein 6A;Actin-like protein 6A (F	Actl6a
NaN	NaN	20.56384	Q9Z329;B2KF89	Q9Z329;B2KF89	Inositol 1,4,5-trisphosphate receptor type 2;	Itpr2
NaN	NaN	NaN	S4R2E1;A0A0R4J0B6;Q7TMS5;D3YZX5;A	S4R2E1;A0A0R4J0B6;Q7TI	ATP-binding cassette sub-family G member 2	Abcg2
NaN	NaN	22.88762	V9GXY4;P58801	V9GXY4;P58801	Receptor-interacting serine/threonine-prote	Ripk2
23.09023	24.06085	NaN	Z4YK56;Q8K135;A2A786;A2A787;A2A78	Z4YK56;Q8K135;A2A786;A	Expressed sequence AU040320;Dyslexia-ass	AU040320;Kiaa0
26.56092	26.56674	26.71293	Z4YKQ9;Q4QY64	Z4YKQ9;Q4QY64	ATPase family AAA domain-containing prote	Atad5
23.44253	23.97596	NaN	Z4YKT6;Q99J47	Z4YKT6;Q99J47	Dehydrogenase/reductase SDR family memb	Dhrs7b
22.63423	22.08213	NaN	A0A087WPH7	A0A087WPH7	26S proteasome regulatory subunit 6A (Frag	Psmc3
22.72234	22.02918	21.71816	A0A087WQ48;H7BWZ9;Q8BL99	A0A087WQ48;H7BWZ9;Q	Protein dopey-1	Dopey1
25.50142	25.61246	NaN	A0A087WRM2;Q91VK4	A0A087WRM2;Q91VK4	Integral membrane protein 2C	Itm2c
26.51803	26.52253	26.34096	A0A087WRZ7;A0A0G2JDM3;E9PWG4;A	A0A087WRZ7;A0A0G2JDN	Myosin light chain 1/3, skeletal muscle isofo	Myl1;Myl3
26.90885	27.16997	27.19835	A0A087WSN6;B9EHT6;B7ZNJ1	A0A087WSN6;B9EHT6;B7	Fibronectin	Fn1
NaN	NaN	25.77706	A0A0A0MQ90;P97352	A0A0A0MQ90;P97352	Protein S100-A13	S100a13
NaN	NaN	NaN	A0A0A0MQN1;Q5RL79	A0A0A0MQN1;Q5RL79	Keratinocyte associated protein 2, isoform C	Krtcap2
25.00624	24.48001	25.34435	A0A0A6YVS2;Q921L3	A0A0A6YVS2;Q921L3	Calcium load-activated calcium channel (Fra	Tmco1
24.94056	24.86703	24.89966	A0A0A6YVX4;Q8CII2	A0A0A6YVX4;Q8CII2	Cell division cycle protein 123 homolog (Fra	Cdc123
24.55215	24.81909	NaN	A0A0A6YW87;Q9CX13	A0A0A6YW87;Q9CX13	Cornichon homolog 4 (Drosophila), isoform	Cnih4
NaN	NaN	NaN	A0A0A6YXC8;E9QKR1;A0A0A6YW06;J3C	A0A0A6YXC8;E9QKR1;A0A	Protein enabled homolog	Enah
NaN	NaN	NaN	A0A0A6YXG8;A0A0A6YWJ7;B2RXV4	A0A0A6YXG8;A0A0A6YWJ	Feline leukemia virus subgroup C receptor-re	Flvcr1
NaN	NaN	NaN	A0A0G2JDX4;A0A0G2JEZ7;Q9D1X8;Q92	A0A0G2JDX4;A0A0G2JEZ7	Tetraspanin-2 (Fragment);Tetraspanin	Tspan2
23.13163	22.90058	22.8423	A0A0G2JE26;Q6DFX2	A0A0G2JE26;Q6DFX2	Anthrax toxin receptor 2	Antxr2
21.77153	22.0258	22.14258	A0A0G2JEC8;Q8C0M2;Q3U7M5;Q8VD6	A0A0G2JEC8;Q8C0M2;Q3	Protein ABHD4 (Fragment);Protein ABHD4	Abhd4

NaN	NaN	NaN	A0A0G2JEE5	A0A0G2JEE5	T cell receptor alpha joining 50 (Fragment)	Traj50
24.36453	24.48001	24.70767	A0A0G2JEW0;A0A0G2JH13;Q8CJ26	A0A0G2JEW0;A0A0G2JH1	Death domain-containing membrane protei	Nradd
NaN	22.80538	22.03926	A0A0G2JF52;Q9R0Q6;D3YVI5	A0A0G2JF52;Q9R0Q6;D3Y	Actin related protein 2/3 complex, subunit 1	Arpc1a
NaN	22.69285	NaN	A0A0G2JGA4;A0A0G2JDI9;P55096	A0A0G2JGA4;A0A0G2JDI9	ATP-binding cassette sub-family D member 3	Abcd3
21.48738	NaN	NaN	A0A0G2JGP6;A0A0G2JEW6;D6RHK5;A0	A0A0G2JGP6;A0A0G2JEW	Cyclin-G-associated kinase	Gak;Ga
NaN	NaN	NaN	A0A0G2JGP8;Q9D7I0	A0A0G2JGP8;Q9D7I0	Protein shisa-5	Shisa5
NaN	NaN	NaN	A0A0G2JGR9;Q80VB6;A0A0G2JGQ6;Q9	A0A0G2JGR9;Q80VB6;A0A	Serine/threonine-protein kinase DCLK1 (Frag	Dclk1
26.65949	26.75175	26.92025	A0A0G2JGU1;Q64302	A0A0G2JGU1;Q64302	Transmembrane 4 L6 family member 1 (Frag	Tm4sf1
22.89319	22.95837	22.90242	A0A0G2JGY0;D3YWR7;A0A0G2JGJ1;Q8	A0A0G2JGY0;D3YWR7;A0	Dihydropteridine reductase	Qdpr;
22.81714	22.46513	22.90793	A0A0J9YTY5;D3YY98;Q8VDS8	A0A0J9YTY5;D3YY98;Q8V	Syntaxin-18 (Fragment);Syntaxin-18	Stx18
24.46762	24.33756	23.83846	A0A0J9YUQ8;A0A0J9YUJ8	A0A0J9YUQ8	Gelsolin (Fragment)	Gsn
24.14102	24.2021	24.1488	A0A0J9YUR9;Q3V3R8;A0A0J9YUI1;A0A	A0A0J9YUR9;Q3V3R8;A0A	Ubiquitin-conjugating enzyme E2 K (Fragme	Ube2k
22.34028	NaN	22.25061	A0A0J9YUT8;Q8R3C6	A0A0J9YUT8;Q8R3C6	Probable RNA-binding protein 19	Rbm19
NaN	19.5545	NaN	A0A0N4SUH4;Q8BKS9	A0A0N4SUH4;Q8BKS9	Pumilio homolog 3	Pum3
23.44253	23.60964	23.01903	A0A0N4SUT8;A0A0N4SVH5;A0A0N4SV	A0A0N4SUT8;A0A0N4SVH	Deoxyribose-phosphate aldolase (Fragment)	Dera
22.64526	22.23608	22.74714	A0A0N4SUZ3;Q6ZWM4	A0A0N4SUZ3;Q6ZWM4	U6 snRNA-associated Sm-like protein LSm8	Lsm8
NaN	NaN	NaN	A0A0N4SVC2;E9QP00;Q6PFR5	A0A0N4SVC2;E9QP00;Q6	Transformer-2 protein homolog alpha	Tra2a
21.55918	21.89874	22.21845	A0A0N4SVT9;H3BKK8;F7CDA2;H3BK97;	A0A0N4SVT9;H3BKK8;F7C	FERM domain-containing protein 4B (Fragm	Frmd4b;Frmd4a
NaN	NaN	NaN	A0A0R4IZW9;Q9DCT5	A0A0R4IZW9;Q9DCT5	Stromal cell-derived factor 2	Sdf2
NaN	NaN	NaN	A0A0R4J086;Q8BK62	A0A0R4J086;Q8BK62	Olfactomedin-like protein 3	Olfml3
NaN	NaN	NaN	A0A0R4J0T2;Q504P2	A0A0R4J0T2;Q504P2	C-type lectin domain family 12 member A	Clec12a
22.69498	22.47754	NaN	A0A0R4J0T8;Q99K28;Q9D8S3	A0A0R4J0T8;Q99K28;Q9D	ADP-ribosylation factor GTPase-activating p	Arfgap3;Arfgap
22.56848	22.75328	22.36185	A0A0R4J0U7;Q8R395	A0A0R4J0U7;Q8R395	COMM domain-containing protein 5	Commd5
24.01052	24.21697	NaN	A0A0R4J0W6;Q9CRC8	A0A0R4J0W6;Q9CRC8	Leucine rich repeat containing 40, isoform C	Lrrc40
22.33484	NaN	22.13946	A0A0R4J131;Q8CIF4	A0A0R4J131;Q8CIF4	Biotinidase	Btd
NaN	NaN	NaN	A0A0R4J1K4;A0A0R4J1K9;A0A0R4J101;	A0A0R4J1K4;A0A0R4J1K9	Anion exchange protein	Slc4a2;
24.81421	25.21327	25.07316	A0A0R4J1L2;F6ZFU0;D3Z7N2	A0A0R4J1L2;F6ZFU0;D3Z7	Elongation factor 1-delta;Elongation factor 1	Eef1d
NaN	NaN	NaN	A0A0R4J2D3;Q8BI84	A0A0R4J2D3;Q8BI84	Transport and Golgi organization protein 1 h	Mia3
20.09507	19.86816	NaN	A0A0U1RNZ3;Q8C5X1	A0A0U1RNZ3;Q8C5X1	RIKEN cDNA 4931406P16 gene	4931406P16Rik
NaN	NaN	NaN	A0A0U1RP00;D6RGU1;A0A0U1RPB0;Q9	A0A0U1RP00;D6RGU1;A0	Nucleolysin TIAR;Nucleolysin TIAR (Fragmen	Tial1
22.58003	23.0476	NaN	A0A140LJC5;A0A140LHQ1;A0A140LHM	A0A140LJC5;A0A140LHQ1	Gamma-secretase subunit PEN-2 (Fragment)	Psenen
NaN	NaN	NaN	A0A1B0GSZ1;O35912	A0A1B0GSZ1;O35912	Epithelial membrane protein 3 (Fragment);E	Emp3
23.29614	22.89134	NaN	A0A1B0GSZ9;O35972	A0A1B0GSZ9;O35972	39S ribosomal protein L23, mitochondrial	Mrpl23
NaN	NaN	NaN	A0A1C7CYU4;A0A1B0GRC2;Q64704	A0A1C7CYU4;A0A1B0GRC	Syntaxin-3	Stx3
21.0559	20.75532	20.65403	A0A1D5RLK1;Q8C3S2	A0A1D5RLK1;Q8C3S2	Transport and Golgi organization protein 6 h	Tango6
24.2021	NaN	NaN	A0A1D5RLZ6;Q9DCC8	A0A1D5RLZ6;Q9DCC8	Mitochondrial import receptor subunit TOM	Tomm20
NaN	NaN	NaN	A0A1D5RM41;A0A0A6YXX3;A0A0A6YXL	A0A1D5RM41;A0A0A6YX	Lipopolysaccharide-responsive and beige-lik	Lrba
NaN	NaN	NaN	A0A1L1SQA2;Q9CQV1	A0A1L1SQA2;Q9CQV1	Predicted gene 9803;Mitochondrial import i	Gm9803;Pam16
NaN	NaN	NaN	A0A1L1SQC5;F7ATH5;Q8R2Y2	A0A1L1SQC5;F7ATH5;Q8R	Cell surface glycoprotein MUC18 (Fragment)	Mcam
NaN	NaN	NaN	A0A1L1SS76;Q8BH57	A0A1L1SS76;Q8BH57	WD repeat-containing protein 48 (Fragment	Wdr48
NaN	NaN	NaN	A0A1L1SSA8;Q91XE8	A0A1L1SSA8;Q91XE8	Transmembrane protein 205 (Fragment);Tra	Tmem205
23.42982	23.00539	23.45513	A0A1L1SSV6;A0A1L1SST5;Q9CQC8	A0A1L1SSV6;A0A1L1SST5	Maspardin (Fragment);Maspardin	Spg21
25.26069	25.46762	25.29263	A0A1L1SUU4;Q9ERN0	A0A1L1SUU4;Q9ERN0	Secretory carrier-associated membrane prot	Scamp2
NaN	24.94056	25.71293	A0A1W2P6G5	A0A1W2P6G5	Myosin light polypeptide 6	Myl6
28.68979	27.95947	26.19648	A0A1W2P768;P84228	A0A1W2P768;P84228	Histone H3.2	Hist2h3c1;Hist1
20.28771	NaN	NaN	A0A1W2P7G3;P23950	A0A1W2P7G3;P23950	mRNA decay activator protein ZFP36L1	Zfp36l1
22.81519	23.03086	23.46762	A0A1Y7VM38;P52503	A0A1Y7VM38;P52503	NADH dehydrogenase [ubiquinone] iron-sul	Ndufs6
NaN	NaN	NaN	A0A1Y7VMF7;O89016	A0A1Y7VMF7;O89016	ATP-binding cassette sub-family D member	Abcd4
NaN	NaN	NaN	A0A286YD04;A0A286YE98;A0A286YDT8	A0A286YD04;A0A286YE98	Tight junction-associated protein 1 (Fragme	Tjap1
NaN	NaN	NaN	A0A286YDS2;A0A286YDF6;B0V2N1	A0A286YDS2;A0A286YDF	Receptor-type tyrosine-protein phosphatas	Ptprs
NaN	NaN	NaN	A2A4J3;A2A4J1;P61290	A2A4J3;A2A4J1;P61290	Proteasome activator complex subunit 3 (Fr	Psme3
23.2535	22.87643	22.84805	A2A6M1;Q9CZ28	A2A6M1;Q9CZ28	Vacuolar-sorting protein SNF8	Snf8
27.60399	27.56819	27.55362	A2A934;A2A930;A2A931;A2A933;A2A9	A2A934;A2A930;A2A931;	PR domain zinc finger protein 16	Prdm16
NaN	23.59833	23.50446	A2A9X5;Q9JM14	A2A9X5;Q9JM14	5(3)-deoxyribonucleotidase, cytosolic type	Nt5c
NaN	23.46762	23.6971	A2AD03;Q9QXG2	A2AD03;Q9QXG2	Rab proteins geranylgeranyltransferase com	Chm
22.17341	23.55215	22.31008	A2AEG3;A2AEG6;P35803	A2AEG3;A2AEG6;P35803	Glycoprotein m6b, isoform CRA_a;Glycoprot	Gpm6b
NaN	NaN	NaN	A2AQJ7;A2AQJ5;A2AQJ6;Q8BXN9	A2AQJ7;A2AQJ5;A2AQJ6;	Transmembrane protein 87A	Tmem87a
23.28207	23.2535	22.91707	A2ASW6;Q9ESL4	A2ASW6;Q9ESL4	Mitogen-activated protein kinase kinase kin	Map3k20
NaN	NaN	25.26428	A2AUF7;B0R0S4	A2AUF7;B0R0S4	Rab9 effector protein with kelch motifs	Rabepk
NaN	NaN	NaN	A6H5X4	A6H5X4	PHD finger protein 11	Phf11
NaN	NaN	22.50203	A6H630	A6H630	Protein-glutamate O-methyltransferase	Armt1
22.97771	NaN	22.98989	A7YY80;Q9WV92;A0A286YDY4;A0A286	A7YY80;Q9WV92;A0A286	130kDa Protein 4.1B MEF cell isoform;Band	Epb41l3
23.57542	23.56384	23.1946	A8Y5H7	A8Y5H7	SEC14-like protein 1	Sec14l1
NaN	NaN	NaN	B1AR50;Q9DCD6	B1AR50;Q9DCD6	Gamma-aminobutyric acid receptor-associat	Gabarap
23.01733	NaN	22.45764	B1ASW5;B1ASW6;Q5NCF2	B1ASW5;B1ASW6;Q5NCF	Trafficking protein particle complex 1, isofor	Trappc1
23.68646	NaN	NaN	B1AUY9;B1AUZ1;Q3V4D5;B1AUY7;Q9Q	B1AUY9;B1AUZ1;Q3V4D5	N-alpha-acetyltransferase 10	Naa10
NaN	NaN	23.417	B1AVZ0	B1AVZ0	Uracil phosphoribosyltransferase homolog	Uprt
NaN	NaN	NaN	B1B0C7	B1B0C7	Basement membrane-specific heparan sulfa	Hspg2
24.93607	24.26069	24.65403	B2RXS8;B9EKR1	B2RXS8;B9EKR1	Ptprz1 protein;Receptor-type tyrosine-prote	Ptprz1
NaN	23.65403	NaN	B7ZCB8;F7CJY7;F6X4N5;Q8BVI5	B7ZCB8;F7CJY7;F6X4N5;Q	Syntaxin-16 (Fragment);Syntaxin-16	Stx16
NaN	NaN	NaN	B8ZXI1	B8ZXI1	Queuine tRNA-ribosyltransferase accessory	Qtrt2
24.20955	23.67573	23.50446	D3YUT6;Q91V77	D3YUT6;Q91V77	Protein S100-A1;Protein S100	S100a1
24.49838	24.73892	NaN	D3YV69	D3YV69	Ras-related protein Rab-6A	Rab6a
NaN	NaN	NaN	D3YVH4;E9Q4Q2;D3YZC9;D3YZD0;Q642	D3YVH4;E9Q4Q2;D3YZC9;	Splicing factor 1 (Fragment);Splicing factor 1	Sf1
23.17493	23.49228	23.01393	D3YVQ6;P35292	D3YVQ6;P35292	Ras-related protein Rab-17 (Fragment);Ras-	Rab17
NaN	NaN	NaN	D3YVV4;Q99KR8	D3YVV4;Q99KR8	Plasma alpha-L-fucosidase (Fragment);Plasm	Fuca2
NaN	NaN	NaN	D3YXA7;Q9CXK4	D3YXA7;Q9CXK4	Sugar transporter SWEET1	Slc50a1
NaN	NaN	NaN	D3YZC1;E9Q1J7;Q99MN9	D3YZC1;E9Q1J7;Q99MN9	Propionyl-CoA carboxylase beta chain, mito	Pccb
23.51653	23.59833	NaN	D3Z0J2;P51637	D3Z0J2	Caveolin (Fragment)	Cav1
23.50446	23.01563	23.00367	D3Z0Z6;D3Z136;Q8BHI7	D3Z0Z6;D3Z136;Q8BHI7	Elongation of very long chain fatty acids pro	Elovl5
22.239	22.09828	21.76344	D3Z1H8;D3Z1H9;Q9JL62	D3Z1H8;D3Z1H9;Q9JL62	Glycolipid transfer protein	Gltp
NaN	23.94949	25.26428	D3Z494;G5E895;S4R2G9	D3Z494;G5E895	Aldo-keto reductase family 1, member B10 (	Akr1b10
23.95837	23.29614	NaN	D3Z568;F6Y6V5;D3YX99;D3Z6W9;Q9C	D3Z568;F6Y6V5;D3YX99;	NADH dehydrogenase (Ubiquinone) 1 beta s	Ndufb5
22.97946	23.09184	23.13477	D3Z5Y5;D3YX12;A0A0A6YXR0;Q8BGS7	D3Z5Y5;D3YX12;A0A0A6Y	Choline/ethanolaminephosphotransferase 1	Cept1
NaN	NaN	NaN	D3Z795;Q9JK23	D3Z795;Q9JK23	Proteasome assembly chaperone 1	Psmg1
22.15808	22.7265	22.97771	D3Z7E5;Q2NL51	D3Z7E5;Q2NL51	Glycogen synthase kinase-3 alpha	Gsk3a
23.50446	23.21697	23.51653	D3Z7J6;D3Z487;Q99LD9	D3Z7J6;D3Z487;Q99LD9	Translation initiation factor eIF-2B subunit	Eif2b2
22.71607	23.1916	23.78955	D3Z7J7;D3Z6Y9;Q99LN9	D3Z7J7;D3Z6Y9;Q99LN9	Deoxyhypusine hydroxylase (Fragment);Deo	Dohh

24.71293	24.45513	24.42342	D3Z7P2;D3Z3Z2;D3YX08;D3Z0I8;Q3UBX	D3Z7P2;D3Z3Z2;D3YX08;D	Transmembrane protein 109 (Fragment);Tra	Tmem109
NaN	NaN	22.94235	D6RGM3;E9QK48;Q7TNG5	D6RGM3;E9QK48;Q7TNG5	Echinoderm microtubule-associated protein	Eml2
22.09184	22.00196	21.89134	D6RGR3;D3Z0L3;Q3T9A5;Q8BZM1	D6RGR3;D3Z0L3;Q3T9A5;	Glomulin;Glomulin (Fragment)	Glmn
NaN	NaN	NaN	D6RI83;D3Z0S6;Q62189	D6RI83;D3Z0S6;Q62189	U1 small nuclear ribonucleoprotein A;U1 sm	Snrpa
NaN	NaN	NaN	D6RIP0;D6RHN4;Q3UXD9;Q99MZ7	D6RIP0;D6RHN4;Q3UXD9;	Peroxisomal trans-2-enoyl-CoA reductase	Pecr
23.64306	24.1332	24.57542	E0CXB9;Q61301;Q8BS72	E0CXB9;Q61301;Q8BS72	Catenin alpha-2;Catenin (Cadherin associate	Ctnna2;
21.54981	NaN	NaN	E0CXM9;Q9DB43	E0CXM9;Q9DB43	Zinc finger protein-like 1 (Fragment);Zinc fin	Zfpl1
20.98815	20.62756	NaN	E0CXW7;E9QMF4;A0A0R4J1Y3	E0CXW7;E9QMF4;A0A0R4	Probable tRNA N6-adenosine threonylcarba	Osgep
NaN	NaN	NaN	E0CYI5;Q6S9I0;Q6S9I2;Q6S9I3	E0CYI5;Q6S9I0;Q6S9I2;Q6	Kininogen 2;HMW-kininogen-II variant	Kng2
22.61863	22.43492	21.6584	E0CYQ4;E0CX64;H3BK26;P49442	E0CYQ4;E0CX64;H3BK26;P	Inositol polyphosphate 1-phosphatase (Frag	Inpp1
27.87408	27.99226	26.09144	E0CZ27;P84244;P02301;F8WI35;E0CYN	E0CZ27;P84244;P02301;F	Histone H3 (Fragment);Histone H3.3;Histone	H3f3a;H3f3c
NaN	23.36453	23.391	E9PYB0	E9PYB0	AHNAK nucleoprotein 2 (Fragment)	Ahnak2
NaN	NaN	NaN	E9PYD5;P10711	E9PYD5;P10711	Transcription elongation factor A protein 1	Tcea1
25.29964	25.60682	25.60682	E9PZD8;Q61147	E9PZD8;Q61147	Ceruloplasmin	Cp
NaN	NaN	NaN	E9Q066;G3X9Q6;Q8BWW4	E9Q066;G3X9Q6;Q8BWW	La-related protein 4	Larp4
23.50446	23.21401	23.66492	E9Q0G1;Q9CQJ6	E9Q0G1;Q9CQJ6	Density-regulated protein (Fragment);Densit	Denr
NaN	23.29614	NaN	E9Q0T4;E9PWQ9;Q91YZ2;P56546	E9Q0T4;E9PWQ9;Q91YZ2;	C-terminal-binding protein 2 (Fragment)	Ctbp2
NaN	NaN	NaN	E9Q1Y3;E9Q414	E9Q1Y3;E9Q414	Apolipoprotein B-100 (Fragment);Apolipopro	Apob
22.80931	23.29614	22.94771	E9Q2H8;E9PYA3;A0A0R4J052;G5E8T9;C	E9Q2H8;E9PYA3;A0A0R4J	Hydroxyacylglutathione hydrolase, mitochor	Hagh
NaN	26.11942	NaN	E9Q3T0	E9Q3T0	Predicted pseudogene 10073	Gm10073
23.13477	22.59377	NaN	E9Q3X0;D3Z2N7	E9Q3X0	Major vault protein	Mvp
21.36986	22.13946	NaN	E9Q411	E9Q411	Neuroblastoma-amplified sequence	Nbas
NaN	NaN	NaN	E9Q6P5;A0A1Y7VL44	E9Q6P5;A0A1Y7VL44	Tetratricopeptide repeat protein 7B	Ttc7b
23.95837	23.55215	23.75939	E9Q704;F8VQA4;P97467	E9Q704;F8VQA4;P97467	Peptidyl-glycine alpha-amidating monooxyge	Pam
NaN	NaN	NaN	E9Q8N5;Q08EB6;Q08EB5;F7DCH5;Q8BR	E9Q8N5;Q08EB6;Q08EB5;	CLIP-associating protein 2	Clasp2
NaN	NaN	NaN	E9Q9A9	E9Q9A9	2-5-oligoadenylate synthase 2	Oas2
NaN	NaN	NaN	E9QAY1;Q8K4F6	E9QAY1;Q8K4F6	Probable 28S rRNA (cytosine-C(5))-methyltra	Nsun5
23.36453	23.417	23.62087	E9QLL2;Q8BZ98;D6RH60;F2Z460;E0CXZ	E9QLL2;Q8BZ98	Dynamin-3	Dnm3
24.53445	NaN	NaN	E9QLR3	E9QLR3	Lysosomal-associated transmembrane prote	Laptm4a
NaN	NaN	22.12692	E9QLZ0;Q8BMC3;P98083	E9QLZ0;Q8BMC3;P98083	MCG18215;SHC-transforming protein 2;SHC	Shc2;Shc1
NaN	NaN	NaN	F6SFF5;Q8K1Z0	F6SFF5;Q8K1Z0	Ubiquinone biosynthesis protein COQ9, mito	Coq9
NaN	24.239	NaN	F6TWM7;F8WJA1;P97808	F6TWM7;F8WJA1;P97808	FXYD domain-containing ion transport regul	Fxyd5
21.57773	21.45764	21.06907	F6ULQ4;Q3U141;A0A0R4J0T4;Q5D0E0;	F6ULQ4;Q3U141;A0A0R4J	Inhibitor of nuclear factor kappa-B kinase su	Ikbkb
24.32389	24.78457	24.46762	F6VRP8	F6VRP8	Galectin-3-binding protein (Fragment)	Lgals3bp
25.82153	26.27853	25.92479	F6VSK8	F6VSK8	Integrin alpha-6 (Fragment)	Itga6
NaN	NaN	NaN	F6XLZ3;D3Z3S1;Q9WUQ2	F6XLZ3;D3Z3S1;Q9WUQ2	Prolactin regulatory element-binding protei	Preb
NaN	NaN	NaN	F6Z9X8;Q8CIE4	F6Z9X8;Q8CIE4	Poly [ADP-ribose] polymerase (Fragment);Po	Parp10
NaN	NaN	21.48738	F6ZZ61;Q3URQ0	F6ZZ61;Q3URQ0	Testis-expressed protein 10 (Fragment);Test	Tex10
NaN	NaN	24.03591	F7AI87	F7AI87	26S proteasome non-ATPase regulatory sub	Psmd4
21.66275	20.90976	21.35917	F7B209;Q80UP5;Q6PD24	F7B209;Q80UP5;Q6PD24	Ankyrin repeat domain-containing protein 1	Ankrd13a
24.09346	24.10949	24.06907	F7BWT7	F7BWT7	Tetraspanin-15	Tspan15
23.46762	23.6971	23.44253	F8WGB3;Q9CZB0	F8WGB3;Q9CZB0	Succinate dehydrogenase cytochrome b560	Sdhc
NaN	NaN	NaN	F8WHG5;Q60823	F8WHG5;Q60823	RAC-beta serine/threonine-protein kinase	Akt2
24.36453	24.57542	24.28207	F8WHU8;Q9D8T7	F8WHU8;Q9D8T7	SRA stem-loop-interacting RNA-binding prot	Slirp
23.31008	24.74919	23.37782	F8WI55;Q8VE70	F8WI55;Q8VE70	Programmed cell death protein 10	Pdcd10
NaN	22.35649	22.22435	F8WI56;D3Z7D3;Q8BWT5	F8WI56;D3Z7D3;Q8BWT5	Disco-interacting protein 2 homolog A	Dip2a
24.239	24.28912	24.53445	G3UX99;G3UZY2;A2A439;P97493	G3UX99;G3UZY2;A2A439;	Thioredoxin, mitochondrial (Fragment);Thio	Txn2
23.21845	23.07725	22.94414	G3UXB5;G3XA75;G3UXX9;G3XA66;D3Z3	G3UXB5;G3XA75;G3UXX9;	Metaxin-1 (Fragment)	Mtx1
21.77555	NaN	NaN	G3UYD5;G3UX23;Q3UW64;Q91WG8	G3UYD5;G3UX23;Q3UW6	Bifunctional UDP-N-acetylglucosamine 2-epi	Gne
23.45513	23.40406	23.48001	G3UZX6;H7BWX9;D3Z794;G3UZ60;G3U	G3UZX6;H7BWX9;D3Z794	Small ubiquitin-related modifier 3 (Fragmen	Sumo3;Sumo2
20.90976	20.92434	20.92434	G3X8P9;O54754	G3X8P9;O54754	Aldehyde oxidase 1	Aox1
21.75532	21.29894	NaN	G3X8S1;Q9CQV7	G3X8S1;Q9CQV7	DnaJ (Hsp40) homolog, subfamily C, membe	Dnajc19
23.46762	NaN	23.60964	G3X909;G3UY21;A0A140T8T2;G3UYX7;	G3X909;G3UY21;A0A140T	Slit homolog 2 protein	Slit2
NaN	NaN	NaN	G3X9I1;G5E911;Q91VU0	G3X9I1;G5E911;Q91VU0	DNA segment, Chr 6, Wayne State University	Fam3c
22.15808	21.9061	21.83846	G3X9I4;Q9JJW6;O08583	G3X9I4;Q9JJW6;O08583	Aly/REF export factor 2;THO complex subun	Alyref2;Alyref
NaN	NaN	NaN	G5E814;Q9D8B4	G5E814;Q9D8B4	MCG5603;NADH dehydrogenase [ubiquinon	Ndufa11
24.78955	25.37119	25.43936	G5E8N3;D3YYC3;O88974	G5E8N3;D3YYC3;O88974	Histone-lysine N-methyltransferase	Setdb1
23.70767	23.5285	23.11902	G5E8R4;Q922D4	G5E8R4;Q922D4	SAPS domain family, member 3, isoform CRA	Ppp6r3
NaN	NaN	NaN	H3BJI7;Q8C6B0;Q76I24;Q5I0W6;Q76I26	H3BJI7;Q8C6B0;Q76I24;Q	Methyltransferase hypoxia-inducible domai	Methig1;Mettl7
NaN	NaN	23.54038	H3BJY1;H3BJ78;H3BKF4;H3BIZ8;B0QZP8	H3BJY1;H3BJ78;H3BKF4;H	Synaptogyrin-2;Synaptogyrin-2 (Fragment);	Syngr2;Gm2070
22.20359	22.47259	23.29614	H3BK73;P0C7W3;Q8R1J9	H3BK73;P0C7W3;Q8R1J9	Prosalusin (Fragment);Prosalusin;Torsin-2A	Tor2a
24.33074	24.26069	24.30313	H3BLI9;D3YW86;D3Z0V7;E9QLZ1;P6250	H3BLI9;D3YW86;D3Z0V7;	TSC22 domain family protein 1	Tsc22d1;T
24.26785	24.34435	24.1488	H7BWZ1;D3YW97;P61961	H7BWZ1;D3YW97;P61961	Ubiquitin-fold modifier 1	Ufm1
NaN	NaN	NaN	H9KV05;Q99JN2	H9KV05;Q99JN2	Kelch-like protein 22	Klhl22
NaN	NaN	23.37782	I7HJS1;O08579	I7HJS1;O08579	Emerin	Emd
22.9619	23.21252	23.0476	J3QMV5;Q9Z2L7	J3QMV5;Q9Z2L7	Cytokine receptor-like factor 3	Crlf3
23.59833	23.26785	23.66492	J3QNW4;Q3TIR1	J3QNW4;Q3TIR1	Trafficking protein particle complex subunit	Trappc13
24.01052	23.68646	24.1332	J3QNY6;Q9QY30	J3QNY6;Q9QY30	Bile salt export pump	Abcb11
28.30226	28.21234	28.15557	K3W4R2;Q6URW6;A0A140LI60	K3W4R2;Q6URW6	Myosin-14	Myh14
NaN	23.26785	23.1961	M0QWJ2;A0A087WQM3;M0QWQ0;B2K	M0QWJ2;A0A087WQM3;	Ribulose-phosphate 3-epimerase	Rpe
24.42982	24.88577	25.72858	O08792	O08792	Transcription factor COE2	Ebf2
23.56384	24.21697	23.79947	O09117	O09117	Synaptophysin-like protein 1	Sypl1
22.99162	23.417	22.73066	O09118	O09118	Netrin-1	Ntn1
NaN	NaN	NaN	O35657	O35657	Sialidase-1	Neu1
NaN	NaN	NaN	O35841	O35841	Apoptosis inhibitor 5	Api5
NaN	24.26069	23.85757	O35901;O35900	O35901;O35900	U6 snRNA-associated Sm-like protein LSm2	Lsm2
21.71398	NaN	NaN	O35954	O35954	Membrane-associated phosphatidylinositol	Pitpnm1
26.22986	26.2553	27.13124	O55112	O55112	AF4/FMR2 family member 2	Aff2
NaN	NaN	NaN	O55188	O55188	Dentin matrix acidic phosphoprotein 1	Dmp1
25.16039	24.79452	24.96279	O70274	O70274	Protein tyrosine phosphatase type IVA 2	Ptp4a2
NaN	NaN	NaN	O70514	O70514	Fibroblast growth factor-binding protein 1	Fgfbp1
NaN	NaN	NaN	P04223	P04223	H-2 class I histocompatibility antigen, K-K alp	H2-K1
27.33245	27.32389	27.24445	P11276	P11276	Fibronectin	Fn1
NaN	NaN	NaN	P14824	P14824	Annexin A6	Anxa6
22.96367	22.52612	22.13006	P15208	P15208	Insulin receptor	Insr
29.17236	28.50142	29.15557	P15919	P15919	V(D)J recombination-activating protein 1	Rag1
23.59833	23.40406	24.12534	P24549;O35945;A0A286YDG6;A0A286Y	P24549;O35945;A0A286Y	Retinal dehydrogenase 1;Aldehyde dehydrog	Aldh1a1;Aldh1a
24.08537	24.08537	NaN	P31650	P31650	Sodium- and chloride-dependent GABA tran	Slc6a11

NaN	NaN	NaN	P32037	P32037	Solute carrier family 2, facilitated glucose tra	Slc2a3
26.32732	26.22619	26.1488	P35276;D3YWL1;D3YW33	P35276;D3YWL1;D3YW33	Ras-related protein Rab-3D;Ras-related prot	Rab3d
23.58692	23.80931	23.82881	P35821	P35821	Tyrosine-protein phosphatase non-receptor	Ptpn1
24.78955	24.23169	25.13712	P47757;A0A0A0MQI9;F6YHZ8	P47757;A0A0A0MQI9;F6Y	F-actin-capping protein subunit beta;F-actin-	Capzb
21.47754	21.59605	22.86703	P53395	P53395	Lipoamide acyltransferase component of bra	Dbt
NaN	NaN	NaN	P54763	P54763	Ephrin type-B receptor 2	Ephb2
25.23535	25.31354	25.31008	P55821	P55821	Stathmin-2	Stmn2
NaN	NaN	NaN	P56564	P56564	Excitatory amino acid transporter 1	Slc1a3
22.75939	22.66709	24.49838	P58854	P58854	Gamma-tubulin complex component 3	Tubgcp3
NaN	22.93697	22.49228	P60060	P60060	Protein transport protein Sec61 subunit gam	Sec61g
29.19835	29.05827	28.79266	P60710;E9Q1F2;E9Q5F4;G3UZ07;E9Q6	P60710;E9Q1F2;E9Q5F4;G	Actin, cytoplasmic 1;Actin, cytoplasmic 1 (Fr	Actb
23.31008	23.04427	NaN	P61166	P61166	Transmembrane protein 258	Tmem258
22.98989	22.74919	22.56384	P62307	P62307	Small nuclear ribonucleoprotein F	Snrpf
23.99335	23.91342	23.79947	P62737;P63268;D3YZY0;D3Z2K3;A0A0U	P62737;P63268;D3YZY0	Actin, aortic smooth muscle;Actin, gamma-e	Acta2;Actg2
24.60399	24.27498	24.55215	P62821	P62821	Ras-related protein Rab-1A	Rab1A
NaN	25.64581	25.79203	P62892	P62892	60S ribosomal protein L39	Rpl39
24.62087	24.77957	24.42982	P63011;D3YZP5	P63011;D3YZP5	Ras-related protein Rab-3A;Ras-related prot	Rab3a
25.74919	25.77957	25.54333	P68373;Q9JJZ2	P68373;Q9JJZ2	Tubulin alpha-1C chain;Tubulin alpha-8 chair	Tuba1c;Tuba8
22.94056	22.77555	22.91889	P97346	P97346	Nucleoredoxin	Nxn
21.47754	NaN	18.08472	Q31093	Q31093	Histocompatibility 2, M region locus 3	H2-M3
NaN	21.44253	NaN	Q3TAA8	Q3TAA8	Phosphatidylinositol glycan anchor biosynth	Pigu
NaN	NaN	NaN	Q3TBT3	Q3TBT3	Stimulator of interferon genes protein	Tmem173
24.99335	25.22435	25.48308	Q3TIR6;P61759	Q3TIR6;P61759	Prefoldin subunit 3	Vbp1
NaN	NaN	NaN	Q3TSS7;P52019	Q3TSS7;P52019	Squalene monooxygenase	Sqle
26.69975	26.78955	24.76446	Q3TYJ3;D3Z0M6;O88485	Q3TYJ3;D3Z0M6;O88485	Cytoplasmic dynein 1 intermediate chain 1	Dync1i1
24.11744	24.01903	23.57542	Q3TYS2	Q3TYS2	Uncharacterized protein C17orf62 homolog	
25.5285	25.56384	NaN	Q3U0J8	Q3U0J8	TBC1 domain family member 2B	Tbc1d2b
23.64306	23.99335	23.88577	Q3U3W2	Q3U3W2	Transmembrane protein 181A	Tmem181a
NaN	NaN	NaN	Q3U687;E9PXF7;D3Z6F0	Q3U687;E9PXF7;D3Z6F0	Interferon-induced protein with tetratricope	Ifit1bl2;Ifit1bl1
26.76446	26.68646	26.57109	Q3UHD9	Q3UHD9	Arf-GAP with GTPase, ANK repeat and PH do	Agap2
22.29334	22.6006	NaN	Q3UHH1	Q3UHH1	Zinc finger SWIM domain-containing protein	Zswim8
NaN	24.21697	NaN	Q3UI43	Q3UI43	BRISC and BRCA1-A complex member 1	Babam1
23.70767	23.63201	NaN	Q3UI84;Q99J62	Q3UI84;Q99J62	Replication factor C subunit 4	Rfc4
22.86136	NaN	22.55918	Q3UN00;F8WI93;Q9Z1M0	Q3UN00;F8WI93;Q9Z1M0	P2X purinoceptor;p2X purinoceptor;P2X pur	P2rx7
22.37782	22.23608	NaN	Q3UNN4;Q3UID0;P97496;Q6PDG5	Q3UNN4;Q3UID0;P97496;	SWI/SNF complex subunit SMARCC1;SWI/SN	Smarcc1;Smarcc
NaN	NaN	NaN	Q45VK5;A0A1L1STE4;Q9Z1X4	Q45VK5;A0A1L1STE4;Q9Z	Interleukin enhancer-binding factor 3	Ilf3
NaN	NaN	NaN	Q4PZA2	Q4PZA2	Endothelin-converting enzyme 1	Ece1
NaN	21.80735	NaN	Q52KR6;B8JJ92;B8JJ91;F6RJ39;Q9JIX8	Q52KR6;B8JJ92;B8JJ91;F6	Acin1 protein;Apoptotic chromatin condensa	Acin1
NaN	NaN	NaN	Q5F258;Q68FF6	Q5F258;Q68FF6	ARF GTPase-activating protein GIT1	Git1
NaN	NaN	23.29614	Q5SQX6;F6QD74;G3UZI5	Q5SQX6;F6QD74	Cytoplasmic FMR1-interacting protein 2;Cyto	Cyfip2
23.48001	23.64306	22.97946	Q60876	Q60876	Eukaryotic translation initiation factor 4E-bin	Eif4ebp1
NaN	NaN	NaN	Q61165	Q61165	Sodium/hydrogen exchanger 1	Slc9a1
23.97596	23.82881	23.93157	Q61390;B1AT05	Q61390;B1AT05	T-complex protein 1 subunit zeta-2	Cct6b
21.75124	21.0625	19.39833	Q61609	Q61609	Sodium-dependent phosphate transporter 1	Slc20a1
NaN	NaN	NaN	Q61823	Q61823	Programmed cell death protein 4	Pdcd4
22.79947	22.69922	NaN	Q62241	Q62241	U1 small nuclear ribonucleoprotein C	Snrpc
NaN	NaN	NaN	Q62312	Q62312	TGF-beta receptor type-2	Tgfbr2
23.79947	24.65403	24.37119	Q63918	Q63918	Caveolae-associated protein 2	Cavin2
NaN	22.59377	NaN	Q6NVE8	Q6NVE8	WD repeat-containing protein 44	Wdr44
23.65403	NaN	24.391	Q6PAV2	Q6PAV2	Probable E3 ubiquitin-protein ligase HERC4	Herc4
NaN	NaN	NaN	Q6PFD6	Q6PFD6	Kinesin-like protein KIF18B	Kif18b
22.46513	22.62979	22.81714	Q6PIP5	Q6PIP5	NudC domain-containing protein 1	Nudcd1
NaN	NaN	24.32389	Q6QI06	Q6QI06	Rapamycin-insensitive companion of mTOR	Rictor
25.42982	25.317	25.30313	Q78XF5	Q78XF5	Oligosaccharyltransferase complex subunit O	Ostc
26.60399	26.86231	26.75175	Q78YZ6	Q78YZ6	Short coiled-coil protein	Scoc
23.51653	23.391	23.54038	Q7TMM9	Q7TMM9	Tubulin beta-2A chain	Tubb2a
23.22435	23.63201	23.74919	Q7TSV4	Q7TSV4	Phosphoglucomutase-2	Pgm2
NaN	NaN	NaN	Q80U93	Q80U93	Nuclear pore complex protein Nup214	Nup214
23.239	22.90976	NaN	Q80UW5	Q80UW5	Serine/threonine-protein kinase MRCK gamn	Cdc42bpg
23.03086	22.73686	23.02749	Q80WS3	Q80WS3	rRNA/tRNA 2-O-methyltransferase fibrillarin	Fbll1
25.61246	NaN	24.51051	Q80YQ1	Q80YQ1	Thrombospondin-1	Thbs1
25.39754	25.33074	25.80193	Q810Q5	Q810Q5	Normal mucosa of esophagus-specific gene 1	Nmes1
25.32389	25.34435	25.37451	Q8BGB7	Q8BGB7	Enolase-phosphatase E1	Enoph1
NaN	NaN	NaN	Q8BGS0	Q8BGS0	Protein MAK16 homolog	Mak16
24.48616	23.96719	23.55215	Q8BGX0	Q8BGX0	E3 ubiquitin-protein ligase TRIM23	Trim23
24.14102	24.24627	24.08537	Q8BH40;O70439	Q8BH40;O70439	Syntaxin-7	Stx7
NaN	NaN	NaN	Q8BHX6;J3QK52;Q9WV70	Q8BHX6;J3QK52;Q9WV70	Nucleolar complex protein 2 homolog	Noc2l
21.25926	NaN	NaN	Q8BK80;Q9DBN5	Q8BK80;Q9DBN5	Lon protease homolog 2, peroxisomal	Lonp2
22.63423	22.44506	22.88204	Q8BML1	Q8BML1	MICAL2	Mical2
22.82493	22.31008	22.23608	Q8BNU0	Q8BNU0	Armadillo repeat-containing protein 6	Armc6
22.53326	21.52612	NaN	Q8BTY8	Q8BTY8	Sec1 family domain-containing protein 2	Scfd2
NaN	22.88948	NaN	Q8BUE4	Q8BUE4	Apoptosis-inducing factor 2	Aifm2
NaN	NaN	NaN	Q8BZ20	Q8BZ20	Poly [ADP-ribose] polymerase 12	Parp12
21.99852	20.93157	22.21549	Q8C079;Q8C9H6	Q8C079;Q8C9H6	Striatin-interacting protein 1;Striatin-interac	Strip1;Strip2
NaN	NaN	21.34841	Q8C0D5	Q8C0D5	Elongation factor-like GTPase 1	Efl1
NaN	NaN	NaN	Q8C1Y8	Q8C1Y8	Vacuolar fusion protein CCZ1 homolog	Ccz1
NaN	NaN	NaN	Q8C561	Q8C561	LMBR1 domain-containing protein 2	Lmbrd2
25.68646	25.63478	25.79452	Q8C5R8	Q8C5R8	Phosphoribosyl pyrophosphate synthetase 1	Prps1l1
24.60964	24.1488	24.27498	Q8C7E4	Q8C7E4	Ribonuclease 4	Rnase4
23.72858	23.83846	22.30452	Q8CF66	Q8CF66	Ragulator complex protein LAMTOR4	Lamtor4
23.68646	23.68646	23.88577	Q8CFV9	Q8CFV9	Riboflavin kinase	Rfk
NaN	NaN	NaN	Q8CFZ0;G3UYP0;P63280	Q8CFZ0;G3UYP0;P63280	SUMO-conjugating enzyme UBC9;SUMO-cor	Ube2i
NaN	NaN	NaN	Q8CGA0	Q8CGA0	Protein phosphatase 1F	Ppm1f
NaN	NaN	NaN	Q8CHT0	Q8CHT0	Delta-1-pyrroline-5-carboxylate dehydrogen	Aldh4a1
24.42342	24.28207	24.36453	Q8K0B2	Q8K0B2	Probable lysosomal cobalamin transporter	Lmbrd1
NaN	23.03423	NaN	Q8K0C1	Q8K0C1	Importin-13	Ipo13
NaN	23.05258	NaN	Q8K0P3	Q8K0P3	TLD domain-containing protein 1	Tldc1
22.6006	23.16576	22.79749	Q8K1S3	Q8K1S3	Netrin receptor UNC5B	Unc5b

NaN	NaN	25.18707	Q8K2I9	Q8K2I9	F-box DNA helicase 1	Fbxo18
28.60045	28.41135	28.36202	Q8R015	Q8R015	Biogenesis of lysosome-related organelles co	Bloc1s5
NaN	20.48738	20.2764	Q8R1L4	Q8R1L4	ER lumen protein-retaining receptor 3	Kdelr3
23.86703	23.88577	24.10949	Q8R1T1	Q8R1T1	Charged multivesicular body protein 7	Chmp7
23.54038	22.80341	23.36453	Q8R527	Q8R527	Rho-related GTP-binding protein RhoQ	Rhoq
NaN	21.85377	22.00881	Q8VBZ0	Q8VBZ0	Dehydrogenase/reductase SDR family memb	Dhrsx
23.391	NaN	NaN	Q8VEJ9;Q8BPY9	Q8VEJ9;Q8BPY9	Vacuolar protein sorting-associated protein	Vps4a;Fignl1
22.31838	21.78357	21.38047	Q8VHK9	Q8VHK9	ATP-dependent RNA helicase DHX36	Dhx36
NaN	NaN	NaN	Q8VI93	Q8VI93	2-5-oligoadenylate synthase 3	Oas3
NaN	NaN	21.7348	Q91VB8;P01942	Q91VB8;P01942	Alpha globin 1;Hemoglobin subunit alpha	Hba-a1;Hba
22.7348	22.69922	22.5545	Q91VE0	Q91VE0	Long-chain fatty acid transport protein 4	Slc27a4
23.46762	NaN	NaN	Q91VJ4	Q91VJ4	Serine/threonine-protein kinase 38	Stk38
23.2006	23.1946	23.33756	Q91X96	Q91X96	Guanine nucleotide exchange factor MSS4	Rabif
21.53089	21.18253	21.27071	Q920I9	Q920I9	WD repeat-containing protein 7	Wdr7
26.95394	26.82639	26.35448	Q922R8	Q922R8	Protein disulfide-isomerase A6	Pdia6
NaN	NaN	NaN	Q923D4	Q923D4	Splicing factor 3B subunit 5	Sf3b5
NaN	22.68432	NaN	Q923F1	Q923F1	Chloride channel, nucleotide-sensitive, 1A	Clns1a
NaN	NaN	22.94949	Q924T2	Q924T2	28S ribosomal protein S2, mitochondrial	Mrps2
22.73892	22.85377	22.94414	Q99J27	Q99J27	Acetyl-coenzyme A transporter 1	Slc33a1
23.54038	23.55215	23.57542	Q99J83	Q99J83	Autophagy protein 5	Atg5
NaN	NaN	NaN	Q99J93;A0A1B0GT68	Q99J93;A0A1B0GT68	Interferon-induced transmembrane protein	Ifitm2;Gm4571
23.13946	22.67142	21.77555	Q99K23	Q99K23	Ufm1-specific protease 2	Ufsp2
NaN	NaN	NaN	Q99KI3	Q99KI3	ER membrane protein complex subunit 3	Emc3
NaN	NaN	NaN	Q99M28	Q99M28	RNA-binding protein with serine-rich domai	Rnps1
23.391	23.50446	22.62756	Q99P72;Q8BHF5	Q99P72;Q8BHF5	Reticulon-4;Reticulon	Rtn4
23.77957	23.90426	23.77957	Q9CPN9	Q9CPN9	RIKEN cDNA 2210010C04 gene	2210010C04Rik
24.49228	24.59263	24.44253	Q9CPP6	Q9CPP6	NADH dehydrogenase [ubiquinone] 1 alpha	Ndufa5
NaN	NaN	22.67573	Q9CPR5	Q9CPR5	39S ribosomal protein L15, mitochondrial	Mrpl15
22.92976	22.41957	22.42214	Q9CPS7	Q9CPS7	RNA-binding protein PNO1	Pno1
NaN	NaN	NaN	Q9CPZ6	Q9CPZ6	ORM1-like protein 3	Ormdl3
23.32389	NaN	22.47754	Q9CQ28	Q9CQ28	Diphthine--ammonia ligase	Dph6
23.80931	23.66492	23.62087	Q9CQ54	Q9CQ54	NADH dehydrogenase [ubiquinone] 1 subun	Ndufc2
23.46762	23.33756	23.94056	Q9CQS5	Q9CQS5	Serine/threonine-protein kinase RIO2	Riok2
23.88577	NaN	23.58692	Q9CQV4	Q9CQV4	Reticulophagy regulator 3	Retreg3
22.29894	23.2535	22.55684	Q9CR09	Q9CR09	Ubiquitin-fold modifier-conjugating enzyme	Ufc1
NaN	NaN	NaN	Q9CRB2	Q9CRB2	H/ACA ribonucleoprotein complex subunit 2	Nhp2
NaN	NaN	NaN	Q9CT10	Q9CT10	Ran-binding protein 3	Ranbp3
NaN	23.57542	23.32389	Q9CY52	Q9CY52	Probable tRNA(His) guanylyltransferase	Thg1l
NaN	NaN	NaN	Q9CZG3	Q9CZG3	COMM domain-containing protein 8	Commd8
21.58692	NaN	NaN	Q9D1H6	Q9D1H6	NADH dehydrogenase [ubiquinone] 1 alpha	Ndufaf4
24.32389	24.44253	24.47383	Q9D1Q4	Q9D1Q4	Dolichol-phosphate mannosyltransferase su	Dpm3
23.03423	23.73892	22.96543	Q9D7X8	Q9D7X8	Gamma-glutamylcyclotransferase	Ggct
25.1946	25.51653	25.42662	Q9D958	Q9D958	Signal peptidase complex subunit 1	Spcs1
23.94056	23.70767	23.6971	Q9DAK9	Q9DAK9	14 kDa phosphohistidine phosphatase	Phpt1
NaN	NaN	NaN	Q9DAW9	Q9DAW9	Calponin-3	Cnn3
21.9566	20.91707	NaN	Q9DB82;P61600	Q9DB82;P61600	N-acetyltransferase 5 (ARD1 homolog, S. cer	Naa20
23.26785	NaN	23.36453	Q9DBG9	Q9DBG9	Tax1-binding protein 3	Tax1bp3
NaN	NaN	NaN	Q9DCE9	Q9DCE9	Interferon gamma-induced GTPase	Igtp
25.42662	25.1757	25.04843	Q9DCF9	Q9DCF9	Translocon-associated protein subunit gamm	Ssr3
NaN	NaN	22.27071	Q9EPK6	Q9EPK6	Nucleotide exchange factor SIL1	Sil1
NaN	NaN	NaN	Q9EQC7	Q9EQC7	Follistatin-related protein 3	Fstl3
23.01733	22.64966	NaN	Q9EQG9	Q9EQG9	Collagen type IV alpha-3-binding protein	Col4a3bp
24.33756	24.317	21.91707	Q9EQQ2	Q9EQQ2	Protein YIPF5	Yipf5
NaN	NaN	NaN	Q9ER71	Q9ER71	Rho-related GTP-binding protein RhoJ	Rhoj
23.64306	23.94056	24.35783	Q9ERD7;A0A1D5RM76	Q9ERD7	Tubulin beta-3 chain	Tubb3
22.73892	23.59833	NaN	Q9ERY9	Q9ERY9	Probable ergosterol biosynthetic protein 28	Erg28
22.72234	22.38311	22.92615	Q9JIK9	Q9JIK9	28S ribosomal protein S34, mitochondrial	Mrps34
NaN	NaN	NaN	Q9JK53	Q9JK53	Prolargin	Prelp
24.05258	24.06907	24.54628	Q9JKV5	Q9JKV5	Secretory carrier-associated membrane prot	Scamp4
24.89966	24.44884	24.06085	Q9JLJ5	Q9JLJ5	Elongation of very long chain fatty acids pro	Elovl1
24.417	24.55215	25.08942	Q9JLZ6	Q9JLZ6	Hypermethylated in cancer 2 protein	Hic2
24.27498	NaN	23.11744	Q9QUR7	Q9QUR7	Peptidyl-prolyl cis-trans isomerase NIMA-int	Pin1
NaN	24.31008	NaN	Q9QYS9	Q9QYS9	Protein quaking	Qki
NaN	21.2006	NaN	Q9R1T2	Q9R1T2	SUMO-activating enzyme subunit 1	Sae1
22.07561	22.29334	22.73892	Q9WU60	Q9WU60	Attractin	Atrn
23.24916	NaN	NaN	Q9WVK0	Q9WVK0	Type-1 angiotensin II receptor-associated pr	Agtrap
NaN	NaN	NaN	Q9Z1R9	Q9Z1R9	MCG124046	Prss1
NaN	NaN	NaN	Q9Z277	Q9Z277	Tyrosine-protein kinase BAZ1B	Baz1b
NaN	NaN	NaN	Q9Z2H7	Q9Z2H7	PDZ domain-containing protein GIPC2	Gipc2
NaN	24.59833	24.61526	S4R1E5;F7A8H6;O70325	S4R1E5;F7A8H6;O70325	Glutathione peroxidase;Phospholipid hydrop	Gpx4
25.09748	24.80931	24.89966	S4R1K7;S4R1P7;G3UWG7;S4R287;Q9CR	S4R1K7;S4R1P7;G3UWG7;	WASH complex subunit 3;WASH complex su	Washc3
NaN	NaN	NaN	S4R1M2;D3YXK2	S4R1M2;D3YXK2	Scaffold attachment factor B1	Safb
24.18707	23.82881	NaN	V9GXI0;V9GXT7;Q9Z266	V9GXI0;V9GXT7;Q9Z266	SNARE-associated protein Snapin	Snapin
NaN	NaN	NaN	Z4YJT3;Q6ZQ58	Z4YJT3;Q6ZQ58	La-related protein 1	Larp1

Supplemental table 2: Student's T-test between EMT/6 Control and EMT/6 RT

LQF Intensity EMT/6 Control1	LQF Intensity EMT/6 Control2	LQF Intensity EMT/6 Control3	LQF Intensity EMT/6 RT1	LQF Intensity EMT/6 RT2	LQF Intensity EMT/6 RT3	-Log t-test p value EMT/6 Control vs	t-test Difference EMT/6 Control vs	Cluster	Protein IDs	Majority protein IDs
25.42342	26.26964	25.82396	21.91707	19.74878	21.98815	2.402024177	-4.62100474	Higher in EMT/6 Control	P97298;B7ZC25;F6	P97298
23.92252	24.23169	23.81909	21.18253	21.66275	21.65403	3.61071201	-2.4913311	Higher in EMT/6 Control	Q52KR3;A0A286Y	Q52KR3
27.61807	27.77077	27.4073	25.33756	25.18329	25.81176	3.242294781	-2.154510498	Higher in EMT/6 Control	Q99KN1;A2AIS9;F	Q99KN1;A2AIS9;F
27.77077	28.15074	26.19272	25.16039	25.53742	25.16806	1.564964956	-2.082789103	Higher in EMT/6 Control	E9Q6C2;Q8CG14;A	E9Q6C2;Q8CG14;A
25.9955	26.07928	26.2021	24.33074	24.30313	23.51653	2.764132719	-2.042161306	Higher in EMT/6 Control	E9Q7U2;Q8CGU1	E9Q7U2;Q8CGU1
27.54628	27.80193	27.68646	25.4008	25.67573	25.84086	3.792397013	-2.03909111	Higher in EMT/6 Control	P97499	P97499
28.58262	28.52776	28.27142	26.60399	26.65949	26.42982	4.053206897	-1.896165212	Higher in EMT/6 Control	O09164	O09164
28.37036	NaN	28.29351	26.44884	26.57542	NaN	2.782184501	-1.819802284	Higher in EMT/6 Control	Q6P5F7	Q6P5F7
28.48616	26.71293	25.98902	25.27853	25.14492	25.32732	1.14598295	-1.812449137	Higher in EMT/6 Control	S4R1E5;F7A8H6;O	S4R1E5;F7A8H6;O
24.10149	22.53801	24.08537	21.7348	21.53564	22.09184	1.519521145	-1.787533442	Higher in EMT/6 Control	Q3UZ39;A0A087W	Q3UZ39
26.52253	27.11148	27.58262	25.86938	24.28912	25.70767	1.412212853	-1.783484141	Higher in EMT/6 Control	Q9CY64;A2ASB8;A	Q9CY64
27.509	27.53148	27.63201	25.7543	25.37782	26.2021	2.744684966	-1.779421488	Higher in EMT/6 Control	Q9D8T2	Q9D8T2
29.84146	29.56638	30.08639	28.23991	27.63893	28.54995	2.274859318	-1.688479106	Higher in EMT/6 Control	E9Q8I0;P06909;D6	E9Q8I0;P06909;D6
24.92705	25.72338	24.2535	23.24045	23.23169	23.54038	1.69373538	-1.63046964	Higher in EMT/6 Control	Q8VHB5;F6XXU0	Q8VHB5;F6XXU0
27.07112	26.93157	27.52402	25.44569	25.55215	25.81176	2.774803021	-1.572367986	Higher in EMT/6 Control	O35188;A0A1D5R	O35188
28.92309	29.03486	28.76446	27.391	27.39917	27.33245	4.333893692	-1.533260981	Higher in EMT/6 Control	Q8R366;G3UYZ1;A	Q8R366;G3UYZ1;A
28.39917	28.23535	28.67976	27.20769	27.1795	26.34435	2.095712076	-1.527581533	Higher in EMT/6 Control	Q9CRB1;O54974;D	Q9CRB1;O54974;D
24.20955	23.32389	24.48001	23.417	21.94235	22.09184	1.222555596	-1.520752589	Higher in EMT/6 Control	F6ULQ4;Q3U141;A	F6ULQ4;Q3U141;A
27.53148	28.13124	28.60752	26.85043	25.99981	26.92025	1.600893218	-1.499917348	Higher in EMT/6 Control	Q3UHK6;B7ZNJ5;Q	Q3UHK6
26.17188	25.97815	26.4073	24.82396	24.92252	24.33756	2.611936497	-1.491096497	Higher in EMT/6 Control	P52927;Q6NSP9	P52927;Q6NSP9
23.92252	24.45513	23.80931	23.12692	22.68003	21.98468	1.712096156	-1.465113322	Higher in EMT/6 Control	P32261;A0A0A6YX	P32261;A0A0A6YX
28.87994	28.89735	29.14831	27.42342	27.58978	27.67976	3.600509863	-1.410879135	Higher in EMT/6 Control	P43277;I7HFT9;Q	P43277
26.16615	25.24264	25.27142	24.59833	23.50446	24.48616	1.380340378	-1.363753637	Higher in EMT/6 Control	Q5SUR0;F6RUL9	Q5SUR0
31.19013	31.11768	31.08639	29.92592	29.31743	30.07368	2.36295719	-1.35905838	Higher in EMT/6 Control	Q99K41	Q99K41
27.78332	27.74535	27.93157	26.21141	26.64581	26.60399	3.05741097	-1.333005269	Higher in EMT/6 Control	P43275	P43275
25.16039	24.58118	25.31008	23.83846	23.58692	23.65403	2.313794333	-1.324079514	Higher in EMT/6 Control	F8WH10;E9Q4N8;	F8WH10;E9Q4N8;
28.09144	28.9428	28.01372	26.89735	26.82639	27.41538	1.686827046	-1.302944183	Higher in EMT/6 Control	Q8VD65	Q8VD65
24.8044	24.6971	24.91798	23.87643	23.50446	23.1946	2.459421755	-1.28133138	Higher in EMT/6 Control	Q7TNC4;E9Q715;Q	Q7TNC4;E9Q715;Q
24.96279	25.76951	25.63201	23.93157	23.99335	24.72338	1.595737041	-1.238670349	Higher in EMT/6 Control	Q9EP52	Q9EP52
25.32732	25.66492	25.2535	24.47383	23.82881	24.32389	2.18322177	-1.206403732	Higher in EMT/6 Control	P19788	P19788
28.68646	28.69975	28.90885	27.67303	27.48616	27.5969	3.705421735	-1.179653168	Higher in EMT/6 Control	P10922	P10922
26.03591	25.96058	25.7543	24.42342	24.74919	25.04427	2.39682586	-1.177967072	Higher in EMT/6 Control	Q8K482	Q8K482
27.27142	27.73892	27.46295	26.54628	26.13907	26.26249	2.532766587	-1.175153097	Higher in EMT/6 Control	Q8QZY6	Q8QZY6
29.30226	29.56092	29.30878	28.23991	28.16039	28.24898	3.706662442	-1.174227397	Higher in EMT/6 Control	Q04857	Q04857
26.11347	25.68378	26.44253	24.56964	25.51653	24.64306	1.449055604	-1.170180639	Higher in EMT/6 Control	P17095	P17095
25.18329	25.26785	25.57542	24.34435	23.99335	24.239	2.7212314	-1.149957657	Higher in EMT/6 Control	P50228	P50228
28.27142	28.29351	28.07623	26.77706	27.07112	27.391	2.400376697	-1.133993149	Higher in EMT/6 Control	P97857	P97857
27.0296	27.2803	27.20769	26.69975	25.62366	25.79699	1.529161193	-1.132394155	Higher in EMT/6 Control	Q91VR8	Q91VR8
31.08005	31.07368	31.16639	29.88577	30.03486	30.00838	4.486372237	-1.130366643	Higher in EMT/6 Control	P43274	P43274
29.70306	29.85638	29.5862	28.52402	28.68311	28.60752	3.591866442	-1.110326767	Higher in EMT/6 Control	B1AWE0;Q6PFA2;	B1AWE0;Q6PFA2;I
29.31095	29.42342	29.26696	28.12139	28.32817	28.2803	3.819759531	-1.090488434	Higher in EMT/6 Control	O54962	O54962
28.99496	28.61807	28.98956	27.54628	28.00838	27.77706	2.402849383	-1.090290705	Higher in EMT/6 Control	P05063	P05063
30.86083	30.55728	30.59335	29.72923	29.23078	29.78176	2.259212867	-1.08989652	Higher in EMT/6 Control	Q62000	Q62000
24.98468	25.13712	25.00624	23.94949	23.86703	24.06907	3.857845617	-1.080816905	Higher in EMT/6 Control	G3X9I4;Q9JJW6;O	G3X9I4;Q9JJW6;O
27.81421	27.76446	27.63893	26.50749	26.60399	26.89735	2.946277206	-1.069587708	Higher in EMT/6 Control	P28301	P28301
30.51089	30.71293	30.59335	29.58441	29.54995	29.49572	4.11045004	-1.062358856	Higher in EMT/6 Control	P43276	P43276
32.34625	32.35941	32.38278	31.25912	31.31419	31.33565	5.720961495	-1.059825897	Higher in EMT/6 Control	P15864	P15864
26.64581	27.05051	26.87408	25.75175	25.73376	25.92025	2.884662709	-1.05488205	Higher in EMT/6 Control	A0A087WNM1;Q9	A0A087WNM1;Q9
27.26249	27.20769	27.2803	26.05879	26.18707	26.40568	3.24544909	-1.032979329	Higher in EMT/6 Control	Q00915	Q00915
35.16414	35.14148	35.08718	34.05924	34.09899	34.15285	5.073150266	-1.027236938	Higher in EMT/6 Control	A0A0N4SV66;Q8R	A0A0N4SV66;Q8R
33.20624	33.19601	33.26192	32.2106	32.2106	32.21639	5.985302437	-1.008861542	Higher in EMT/6 Control	Q9EQK5	Q9EQK5
30.23078	30.33031	30.2535	29.34942	29.12139	29.32603	3.671393042	-1.005913417	Higher in EMT/6 Control	Q64523;Q6GSS7;Q	Q64523;Q6GSS7
25.99335	25.15653	26.29789	25.11347	24.60399	24.7543	1.247973273	-0.992003759	Higher in EMT/6 Control	O35295	O35295
25.08132	25.1055	24.27498	23.6971	23.64306	24.26785	1.316871906	-0.951256434	Higher in EMT/6 Control	F7BPW6;E9QAT4;A	F7BPW6;E9QAT4;A
25.27498	25.8044	25.89735	24.72858	24.73892	24.66492	2.081019106	-0.948099136	Higher in EMT/6 Control	P84089;A0A1W2P	P84089;A0A1W2P
25.77957	26.06702	25.7024	25.1946	24.89966	24.66492	2.099407882	-0.929932912	Higher in EMT/6 Control	Q8R105	Q8R105
34.19014	34.36984	34.36333	33.38278	33.31689	33.44579	3.733322404	-0.925949097	Higher in EMT/6 Control	P68433	P68433
29.42542	29.56456	29.40324	28.70636	28.45905	28.45905	3.172522682	-0.922928492	Higher in EMT/6 Control	Q3THW5;P0C0S6;Q	Q3THW5;P0C0S6;Q
27.91456	27.22619	27.44727	26.20955	27.06085	26.60399	1.327708798	-0.904539744	Higher in EMT/6 Control	P28653	P28653
33.63071	33.72517	33.68395	32.79653	32.7692	32.76525	5.194464467	-0.902950287	Higher in EMT/6 Control	Q8C4U8;O35474;Q	Q8C4U8;O35474;Q
25.48922	25.89966	25.8044	24.66492	25.07316	24.74919	2.166281754	-0.902001063	Higher in EMT/6 Control	Q9EPL2	Q9EPL2
23.99335	24.23169	24.14102	22.88017	23.40406	23.37782	2.093782255	-0.901336034	Higher in EMT/6 Control	Q8R0J7	Q8R0J7
25.27853	25.36786	24.77455	24.36453	24.48616	23.86703	1.56669818	-0.901074092	Higher in EMT/6 Control	F8WJG3;P62996	F8WJG3;P62996
26.72598	26.80193	26.47537	25.69975	25.92931	25.69179	2.685347678	-0.894145966	Higher in EMT/6 Control	Q9ESM6	Q9ESM6
26.47383	26.69975	26.15653	25.79452	25.34096	25.51953	1.911601251	-0.891702016	Higher in EMT/6 Control	Q6W5C0	Q6W5C0
27.06085	27.52402	27.0296	26.52552	26.26607	26.15074	1.991842784	-0.890717824	Higher in EMT/6 Control	Q8BH43;B1AUN0;	Q8BH43;B1AUN0
27.07112	26.68646	26.67303	26.02749	26.17188	25.57542	1.787525308	-0.885270437	Higher in EMT/6 Control	Q99KP6	Q99KP6
28.94	28.37451	29.0296	28.03486	27.51653	28.14102	1.457578247	-0.883899689	Higher in EMT/6 Control	F8WH23;E9Q5D9;	F8WH23;E9Q5D9;Q
23.12376	23.86703	23.35111	22.60964	22.52373	NaN	1.267398606	-0.880615552	Higher in EMT/6 Control	Q8BR63	Q8BR63
NaN	24.72338	24.88577	23.95837	23.84805	24.00196	2.705167861	-0.868449847	Higher in EMT/6 Control	O89023	O89023
28.37451	28.26249	28.391	27.29789	27.55362	27.57542	3.048250192	-0.867020289	Higher in EMT/6 Control	P30681;A0A1B0G	P30681;A0A1B0G
26.50749	26.27142	26.75175	25.28912	25.74407	25.92252	1.668635367	-0.858314514	Higher in EMT/6 Control	Q9CXW3;A0A0A6	Q9CXW3
NaN	25.43301	25.34773	24.64856	24.391	24.56964	2.430209122	-0.853969256	Higher in EMT/6 Control	A0A1B0GRR8;Q62	A0A1B0GRR8;Q62
28.13614	28.24898	28.01903	27.1795	27.31527	27.35783	3.24275804	-0.850516001	Higher in EMT/6 Control	O35598;E9PYF2;D	O35598;E9PYF2
28.82941	29.04009	29.06856	27.89157	28.48999	28.01903	1.895857352	-0.84582456	Higher in EMT/6 Control	Q9WVJ3	Q9WVJ3
26.20955	26.26069	26.64581	25.77957	25.04843	25.76699	1.407798524	-0.840358734	Higher in EMT/6 Control	A2AUK7;A2AUK8;	A2AUK7;A2AUK8;A
24.27498	24.40406	24.92705	23.6971	23.77957	23.64306	1.820083732	-0.828784943	Higher in EMT/6 Control	O88200	O88200
35.09899	35.02663	35.17905	34.22647	34.21928	34.38278	3.549162828	-0.82537969	Higher in EMT/6 Control	Q6ZWY9;Q64525;	Q6ZWY9;Q64525;
26.75175	26.64581	25.94726	25.64581	25.39754	25.82881	1.367017024	-0.82421875	Higher in EMT/6 Control	P24604;Q8CFK4;Q	P24604;Q8CFK4;Q
27.67303	28.4073	28.20303	27.58978	27.16997	27.07112	1.408182586	-0.817498525	Higher in EMT/6 Control	O09159	O09159
33.64151	33.69437	33.58665	32.80424	32.81574	32.85712	4.69594023	-0.815141042	Higher in EMT/6 Control	Q5SXR6;Q68FD5;F	Q5SXR6;Q68FD5
29.57542	29.66966	29.40933	28.87408	28.60045	28.7357	2.75603297	-0.81472524	Higher in EMT/6 Control	P47879;H3BLB7;H	P47879;H3BLB7
24.54038	25.19084	24.89966	24.18707	24.00196	24.01903	1.826763218	-0.807607015	Higher in EMT/6 Control	Q9CQF7;Q9CWM4	Q9CQF7;Q9CWM4
27.14102	26.53148	27.07112	26.04427	25.89041	26.4073	1.504544329	-0.80054601	Higher in EMT/6 Control	Q8CG16;Q8CFG9;Q	Q8CG16;Q8CFG9
24.00196	24.44884	23.71816	23.18253	23.1961	23.45513	1.555419469	-0.778401693	Higher in EMT/6 Control	J3QNY6;Q9QY30	J3QNY6;Q9QY30
27.2535	27.62506	27.27142	26.56674	26.54628	26.71293	2.377346872	-0.774674098	Higher in EMT/6 Control	Q8BND5	Q8BND5
30.62854	30.82335	30.62854	29.88867	29.87555	29.99496	3.299505577	-0.773747126	Higher in EMT/6 Control	Q9WV91	Q9WV91
29.44332	29.56819	29.37865	28.77077	28.66966	28.64924	3.47490225	-0.766829809	Higher in EMT/6 Control	G5E8D6;Q9WVJ9;	G5E8D6;Q9WVJ9;I
23.57542	23.72858	23.83846	22.95837	22.79154	23.09989	2.54287212	-0.764223099	Higher in EMT/6 Control	D6RI83;D3Z0S6;Q	D6RI83;D3Z0S6;Q
24.239	24.53445	24.28912	23.40406	23.73892	23.63201	2.32133273	-0.76252683	Higher in EMT/6 Control	Q9JIP6	Q9JIP6
35.27239	35.20478	35.2931	34.38921	34.60885	34.48832	3.419093682	-0.761301676	Higher in EMT/6 Control	P62806	P62806
29.54075	29.78799	29.51465	29.06343	28.75175	28.75494	2.30346551	-0.757761637	Higher in EMT/6 Control	Q6P069	Q6P069
24.37119	NaN	24.65949	23.87643	23.65403	23.75939	1.936078412	-0.752057076	Higher in EMT/6 Control	D3YUT6;Q91V77	D3YUT6;Q91V77
25.15653	25.27142	25.35111	24.417	24.5285	24.58118	3.266072281	-0.750792185	Higher in EMT/6 Control	A0A0G2JGP8;Q9D	A0A0G2JGP8;Q9D
26.20583	26.07112	26.417	25.58978	25.60399	25.2535	2.098076456	-0.748891195	Higher in EMT/6 Control	P51859;E0CXA0;E	P51859;E0CXA0;E
28.34096	28.34096	28.34942	27.62506	27.54628	27.62506	5.026983685	-0.744983673	Higher in EMT/6 Control	B7ZCU2;B7ZCU5;B	B7ZCU2;B7ZCU5;B
24.04427	23.6971	24.00196	23.37782	23.07071	23.09184	2.118481252	-0.73431778	Higher in EMT/6 Control	Q91V08;A0A0N4S	Q91V08
23.57542	23.56384	NaN	22.69285	22.9812	NaN	1.435685335	-0.732604027	Higher in EMT/6 Control	G3UZ26;P50431;G	G3UZ26;P50431;G
25.45513	25.33756	25.46451	24.76446	NaN	24.60964	2.588665872	-0.732017199	Higher in EMT/6 Control	Q9Z2D3;D3YYP5;E	Q9Z2D3;D3YYP5;E
NaN	24.59263	24.5285	23.83846	23.86703	23.78955	3.518357616	-0.728889783	Higher in EMT/6 Control	Q6ZPU9;H3BIY2;E	Q6ZPU9
27.55362	27.33245	27.58262	26.88577	26.71293	26.71293	2.739579611	-0.719022115	Higher in EMT/6 Control	Q64669	Q64669
32.73933	32.52729	32.81765	31.86083	32.0414	32.03813	2.609406755	-0.714635849	Higher in EMT/6 Control	P08207	P08207
26.9428	26.81421	26.90885	26.22803	26.28207	26.01903	2.879574467	-0.712244034	Higher in EMT/6 Control	Q5FWI3	Q5FWI3
27.51653	27.96499	27.9428	27.41538	27.0296	26.85043	1.48413193	-0.709634781	Higher in EMT/6 Control	Q3UH60;B2RQC7;	Q3UH60;B2RQC7;I
30.44332	30.6372	30.50142	29.85192	29.69644	29.91171	2.921635978	-0.707292557	Higher in EMT/6 Control	P48678;D3YUF7	P48678
26.39754	26.33756	25.91114	25.75685	25.51953	25.2535	1.540510327	-0.705455144	Higher in EMT/6 Control	Q01279;Q9WVF5;	Q01279;Q9WVF5
25.1757	25.62366	25.16423	24.95837	24.54038	24.35783	1.402482832	-0.702334722	Higher in EMT/6 Control	Q9Z0E6;E9Q2N7	Q9Z0E6
27.56092	28.15074	27.80193	27.18896	27.1795	27.05051	1.772798634	-0.698210398	Higher in EMT/6 Control	Q9DAU7;A2A5G4;	Q9DAU7;A2A5G4;A
28.1795	27.97596	27.92025	27.0296	27.39917	27.5969	1.686627859	-0.683345159	Higher in EMT/6 Control	Q61739	Q61739

28.87701	29.05051	28.94838	28.31527	28.23535	28.29351	3.580388975	-0.677254995	Higher in EMT/6 Control	G3UXZ5;P97371;G	G3UXZ5;P97371;G
27.72598	27.71947	27.5969	27.01903	27.06085	26.93157	3.550251828	-0.676969528	Higher in EMT/6 Control	Q8BGZ6;P51569;A	Q8BGZ6;P51569
26.77706	26.89735	27.24445	26.36786	26.36119	26.1757	1.922094115	-0.671372732	Higher in EMT/6 Control	Q64442	Q64442
27.1795	27.42342	27.22619	26.55947	26.61807	26.64581	2.972069398	-0.668585459	Higher in EMT/6 Control	A0A0G2JEK2;P632	A0A0G2JEK2;P632
28.76762	28.84146	28.84146	28.18423	28.10149	28.20303	4.098033225	-0.653926849	Higher in EMT/6 Control	Q9WVA4;A0A0A6	Q9WVA4;A0A0A6Y
30.45317	30.45317	30.36202	29.78332	29.73409	29.79266	4.293305565	-0.652766546	Higher in EMT/6 Control	P47880	P47880
25.73117	NaN	25.81665	25.18329	25.11744	25.06907	2.926793142	-0.650648753	Higher in EMT/6 Control	Q9WTP6;F7BP55	Q9WTP6
24.54628	24.83846	24.96279	24.10149	24.1795	24.11744	2.176403437	-0.649699529	Higher in EMT/6 Control	A0A0A0MQM7;O0	A0A0A0MQM7;O0
22.33756	22.20955	22.21549	NaN	21.67573	21.54981	2.535361647	-0.64143308	Higher in EMT/6 Control	B1ASW5;B1ASW6;	B1ASW5;B1ASW6;
27.07112	27.33245	26.98685	26.51352	26.40568	26.55508	2.314060995	-0.638710022	Higher in EMT/6 Control	Q8CG19;B1B1E2	Q8CG19;B1B1E2
28.64238	28.94	28.70965	28.16519	27.98142	28.23535	2.247496732	-0.636688232	Higher in EMT/6 Control	Q6PI17;P25785;B1	Q6PI17;P25785;B1
29.22389	29.46879	29.20769	28.70965	28.62157	28.70636	2.649255904	-0.620933533	Higher in EMT/6 Control	P37889	P37889
23.88577	24.2535	24.00196	23.23169	23.54038	23.55215	1.796866098	-0.605665207	Higher in EMT/6 Control	Q9WVA2;Q4FZG7	Q9WVA2;Q4FZG7
24.84805	24.5285	24.93607	23.96719	24.32389	24.22435	1.670310521	-0.599063238	Higher in EMT/6 Control	Q8CCH2	Q8CCH2
29.1866	29.35993	29.0531	28.63547	28.65949	28.509	2.401650727	-0.598557154	Higher in EMT/6 Control	P11688	P11688
25.42982	25.12534	25.18329	24.66492	24.54038	24.74919	2.227822378	-0.59465154	Higher in EMT/6 Control	A0A1N9M4K1;A0A	A0A1N9M4K1;A0A
29.62506	29.64924	29.69644	29.03748	29.08639	29.06856	4.701501834	-0.592772166	Higher in EMT/6 Control	A2AS98;P28660;Q	A2AS98;P28660
28.02433	28.05569	27.9428	27.33245	27.60399	27.31527	2.395060368	-0.590366364	Higher in EMT/6 Control	P70349;B0R1E3	P70349;B0R1E3
23.93157	23.90426	NaN	23.35111	23.46762	23.16423	1.848848092	-0.590262413	Higher in EMT/6 Control	O70421	O70421
28.34942	28.4512	28.2535	27.75175	27.65949	27.87994	2.625657287	-0.587649663	Higher in EMT/6 Control	Q9D0F9;A2CEK3	Q9D0F9;A2CEK3
32.49905	32.53659	32.51559	31.90813	31.91171	31.9746	4.764627743	-0.58559672	Higher in EMT/6 Control	G3X9T8;G3X8Q5;Q	G3X9T8;G3X8Q5
25.90426	25.97596	25.76951	25.19084	25.28207	25.42021	2.539825845	-0.585538864	Higher in EMT/6 Control	Q9WTX5;E9PUV4	Q9WTX5
26.86231	26.87408	26.97596	26.41538	26.31354	26.22803	3.072210652	-0.585133235	Higher in EMT/6 Control	Q8BGD9	Q8BGD9
25.13712	25.43936	25.49228	24.84326	24.60399	24.87173	1.857299287	-0.583258311	Higher in EMT/6 Control	P56812;D3Z7Q5	P56812;D3Z7Q5
25.41377	25.86703	25.37782	24.94056	24.94949	25.02327	1.66038175	-0.581769943	Higher in EMT/6 Control	P97821;D3Z220	P97821
27.76446	28.07112	28.06085	27.23535	27.30661	27.61105	1.719018723	-0.581141154	Higher in EMT/6 Control	A0A1W2P6L9;Q3U	A0A1W2P6L9;Q3U
30.33031	30.34096	30.23078	29.80655	29.68311	29.67472	3.331316275	-0.579221725	Higher in EMT/6 Control	Q7TMB8;A0A0R4J	Q7TMB8;A0A0R4J
31.35153	31.2814	31.39816	30.69644	30.72923	30.87555	3.058085042	-0.576622009	Higher in EMT/6 Control	Q60963;E9Q330;E	Q60963;E9Q330
27.58262	27.86821	27.77077	27.09144	27.27142	27.15074	2.341342815	-0.56933403	Higher in EMT/6 Control	D3Z3G6;Q63844;A	D3Z3G6;Q63844;A
29.29351	29.258	29.59156	29.04531	28.82941	28.56092	1.500174306	-0.569141388	Higher in EMT/6 Control	Q9DCK3;D3Z0B7;D	Q9DCK3;D3Z0B7;D
27.04009	26.64581	27.0296	26.40406	26.26964	26.33586	1.863418878	-0.568651835	Higher in EMT/6 Control	Q8BJU2;D3YXN7;A	Q8BJU2;D3YXN7;A
30.65438	30.59335	30.64581	30.06085	30.07368	30.06085	5.072144227	-0.566053391	Higher in EMT/6 Control	P34884	P34884
33.68395	33.33031	33.73529	33.06728	33.07846	32.90992	1.820513572	-0.564633687	Higher in EMT/6 Control	Q07797;E9Q5X5	Q07797
26.71293	26.81421	26.82639	26.27675	26.09748	26.29614	2.817765791	-0.561050415	Higher in EMT/6 Control	P13745;Q6P8Q0;D	P13745;Q6P8Q0;D
27.76446	27.99226	27.73247	27.34096	27.34096	27.13124	2.18375626	-0.558676402	Higher in EMT/6 Control	P61089;A0A1W2P	P61089
29.54995	29.79885	29.58082	29.04791	29.13858	29.07112	2.593310815	-0.557336807	Higher in EMT/6 Control	Q99LB4;D3YTL5;P	Q99LB4;D3YTL5;P2
31.27031	31.38792	31.16639	30.70471	30.70471	30.74535	2.98042727	-0.556615829	Higher in EMT/6 Control	P60766;G3UZM2;I	P60766
27.52402	27.73247	27.45513	26.97596	26.97596	27.09144	2.425620289	-0.556084951	Higher in EMT/6 Control	Q9WVE8	Q9WVE8
25.82396	25.58692	25.76699	25.22803	25.03171	25.24989	2.297346552	-0.556083043	Higher in EMT/6 Control	Q6NXL1	Q6NXL1
23.67573	23.56384	23.86703	22.91159	23.20658	23.35111	1.596941791	-0.545773188	Higher in EMT/6 Control	Q3UGB5;Q9JII5;D3	Q3UGB5;Q9JII5;D3
27.49381	27.20769	27.52402	26.92025	26.85043	26.82639	2.18221965	-0.542819341	Higher in EMT/6 Control	P26883;F6X9I3	P26883
27.90885	28.12139	27.97596	27.38278	27.47846	27.51653	2.729249016	-0.54281044	Higher in EMT/6 Control	Q61187;A0A1B0G	Q61187;A0A1B0G
28.15557	28.30226	28.05569	27.75175	27.56819	27.56819	2.344075962	-0.541794459	Higher in EMT/6 Control	Q9CY58;A0A0N4S	Q9CY58;A0A0N4S
29.83244	29.99496	29.84146	29.36411	29.27808	29.40324	2.959415105	-0.54114151	Higher in EMT/6 Control	Q00493	Q00493
28.96224	29.17474	28.95947	28.46684	28.54628	28.47073	2.679359802	-0.537534078	Higher in EMT/6 Control	Q9JIZ9;Q5F284	Q9JIZ9;Q5F284
29.13614	29.23763	29.27808	28.66289	28.51653	28.86526	2.091304007	-0.53572464	Higher in EMT/6 Control	P48759	P48759
28.95671	29.18896	28.97049	28.45905	28.48616	28.56819	2.541524105	-0.534252167	Higher in EMT/6 Control	Q5EBP8;P49312	Q5EBP8;P49312
30.41337	30.60222	30.52962	30.00838	29.95394	29.98142	3.134299048	-0.533825556	Higher in EMT/6 Control	Q3UW53;E9PYV4;	Q3UW53;E9PYV4
25.90885	25.76192	25.87877	25.44884	25.27853	25.22435	2.555059327	-0.532606125	Higher in EMT/6 Control	Q62422	Q62422
27.12139	27.13124	27.04009	26.51051	26.51503	26.67303	3.026398694	-0.531385422	Higher in EMT/6 Control	O88207	O88207
25.08942	25.1757	25.01903	24.50446	24.51653	24.67034	2.789816234	-0.53094101	Higher in EMT/6 Control	G5E8J0;O35516	G5E8J0;O35516
24.74407	24.7024	24.58692	24.09346	24.09346	24.26069	2.716212037	-0.528593063	Higher in EMT/6 Control	Q5SUT0;Q5SUS9;Q	Q5SUT0;Q5SUS9;Q
28.54259	28.42742	28.62157	28.06085	28.04009	27.90885	2.693112541	-0.527263641	Higher in EMT/6 Control	Q62433;E9Q514;E	Q62433
29.8279	30.00838	29.80347	29.31527	29.45905	29.29351	2.49408173	-0.523976644	Higher in EMT/6 Control	P23780;A0A1L1SS	P23780
31.74134	31.91883	31.75732	31.31419	31.24785	31.28692	3.023565862	-0.522843043	Higher in EMT/6 Control	Q61508;D3Z0U9;D	Q61508
31.81192	31.88285	31.7692	31.34096	31.2535	31.30334	3.629758574	-0.522055308	Higher in EMT/6 Control	Q9DBJ1;O70250	Q9DBJ1
29.7973	29.95394	29.79421	29.41941	29.26024	29.30443	2.735975783	-0.520452499	Higher in EMT/6 Control	P70296;D3Z1V4;D	P70296;D3Z1V4;D
26.89735	27.04009	26.89735	26.43141	26.31527	26.54185	2.507076178	-0.515418371	Higher in EMT/6 Control	A6PWS5	A6PWS5
32.98142	32.8792	33.07208	32.45807	32.48951	32.44825	3.068970382	-0.512289683	Higher in EMT/6 Control	P09411;S4R2M7	P09411;S4R2M7
27.80193	27.87408	27.78332	27.27142	27.2535	27.39917	3.173477262	-0.511747996	Higher in EMT/6 Control	Q80XR6;Q20BD0;Q	Q80XR6;Q20BD0;Q
31.94349	32.04466	32.06085	31.48712	31.51089	31.52496	3.729131621	-0.508681615	Higher in EMT/6 Control	Q9WU78	Q9WU78
23.97596	23.93157	NaN	23.42982	23.44253	23.48001	3.543877242	-0.502981186	Higher in EMT/6 Control	D3Z3J6;G3UYE5;F	D3Z3J6;G3UYE5;F6
30.44332	30.57542	30.52028	30.00838	30.02168	30.00838	3.690713861	-0.500192642	Higher in EMT/6 Control	Q99K85;Q3U6K9;E	Q99K85;Q3U6K9
32.5038	32.48472	32.42342	31.89735	32.08004	31.93649	2.927123781	-0.499348958	Higher in EMT/6 Control	P17742;A0A1L1SS	P17742;A0A1L1SS
27.89157	28.04531	27.89735	27.31527	27.47073	27.55362	2.35351801	-0.498203913	Higher in EMT/6 Control	O88325	O88325
32.13001	32.28967	32.23934	31.74134	31.61982	31.80809	2.623568365	-0.496592204	Higher in EMT/6 Control	M0QWP1;Z4YK85;	M0QWP1;Z4YK85;
29.03486	29.25124	29.06085	28.63201	28.6764	28.56092	2.533273289	-0.492537816	Higher in EMT/6 Control	Q8R1F1;A2ARS6	Q8R1F1
31.24218	31.23649	31.32496	30.83846	30.77706	30.71293	3.356817171	-0.491727829	Higher in EMT/6 Control	P10649;A2AE89;F	P10649;A2AE89;F6
24.239	24.317	24.05258	23.62087	23.79947	23.72858	2.177782646	-0.486554464	Higher in EMT/6 Control	E9QN99;Q8VCR7;A	E9QN99;Q8VCR7;A
31.76129	31.81574	31.90096	31.38792	31.31959	31.33565	3.331379886	-0.478277206	Higher in EMT/6 Control	Q02819;A0A1C7C	Q02819;A0A1C7C
32.85527	32.91705	33.00336	32.4919	32.4007	32.44825	3.163047011	-0.478275299	Higher in EMT/6 Control	P05064;A6ZI44;D3	P05064;A6ZI44;D3
28.68646	28.88577	28.72923	28.37451	28.21697	28.28472	2.479759354	-0.475084941	Higher in EMT/6 Control	P97372;G3X9V0;E	P97372;G3X9V0
26.92025	26.96499	26.64581	26.37617	26.26249	26.46918	1.820706744	-0.474410375	Higher in EMT/6 Control	Q61205;D3Z7E6;D	Q61205;D3Z7E6;D
32.04791	32.08639	32.03157	31.59779	31.55728	31.59335	4.660933437	-0.47248586	Higher in EMT/6 Control	E9Q616;G5E8K8	E9Q616
32.37762	32.41589	32.34096	31.79266	32.00169	31.92945	2.712796802	-0.470221837	Higher in EMT/6 Control	P17751;H7BXC3	P17751;H7BXC3
25.88809	25.98468	25.87643	25.39428	25.62923	25.317	2.030358211	-0.469563802	Higher in EMT/6 Control	Q9CQM5	Q9CQM5
27.81421	27.9428	27.87994	27.47846	27.45513	27.29789	2.638675294	-0.468486786	Higher in EMT/6 Control	Q9CQ60;Q8CBG6;	Q9CQ60;Q8CBG6;I
32.90992	32.96774	32.71088	32.3956	32.27864	32.52262	1.938829659	-0.463891347	Higher in EMT/6 Control	E9PX70;Q60847;F	E9PX70;Q60847
27.67303	27.98685	27.89735	27.50142	27.30661	27.3662	1.864294849	-0.46100235	Higher in EMT/6 Control	Q9D1C8	Q9D1C8
24.87643	25.04427	24.94503	24.42342	24.42342	24.64856	2.157530846	-0.456774394	Higher in EMT/6 Control	P70441	P70441
26.60399	26.52999	26.49228	26.24445	26.06085	25.95172	2.121898322	-0.456417084	Higher in EMT/6 Control	Q8CI19	Q8CI19
28.37036	28.37865	28.33245	27.87408	27.89157	27.94838	4.170111475	-0.455809911	Higher in EMT/6 Control	P12023	P12023
26.95394	26.75175	26.78955	26.50749	26.32732	26.29789	2.135555328	-0.454179128	Higher in EMT/6 Control	Q99JB8;B1AW91;A	Q99JB8;B1AW91;A
29.73731	29.72923	29.77706	29.47846	29.29789	29.10899	1.864939242	-0.452753703	Higher in EMT/6 Control	E9Q748;P97430	E9Q748;P97430
29.16997	29.09144	29.34731	28.61456	28.74855	28.89446	1.816653382	-0.450381597	Higher in EMT/6 Control	Q8R143;E0CXB5;F	Q8R143;E0CXB5
29.14589	29.23535	29.34731	28.80809	28.67976	28.89446	2.20305461	-0.448745092	Higher in EMT/6 Control	O88569;A0A0N4S	O88569;A0A0N4S
27.66628	27.89735	27.70636	27.32389	27.32389	27.2803	2.449763045	-0.447305679	Higher in EMT/6 Control	O08917;G3UYU4;Q	O08917;G3UYU4
29.53705	29.71293	29.509	29.04009	29.104	29.27586	2.030262196	-0.446340561	Higher in EMT/6 Control	Q9JJH1	Q9JJH1
27.91456	27.78332	27.76446	27.43141	27.39917	27.29789	2.697997066	-0.444619497	Higher in EMT/6 Control	A0A1B0GR11;Q93	A0A1B0GR11;Q93
25.59833	25.83605	25.84086	25.23535	25.36119	25.34773	2.11320619	-0.443657557	Higher in EMT/6 Control	Q9Z1D1	Q9Z1D1
28.50142	28.68311	28.52028	28.20769	28.16039	28.02433	2.270856343	-0.43747139	Higher in EMT/6 Control	Q9CPU0	Q9CPU0
33.35678	33.38278	33.48232	33.05278	32.96086	32.90455	2.778659542	-0.434563955	Higher in EMT/6 Control	Q564E2;P06151;A	Q564E2;P06151;A
24.64856	24.71293	24.76446	24.23169	24.12534	24.46762	1.81782629	-0.433764776	Higher in EMT/6 Control	P62743;A0A0U1R	P62743;A0A0U1R
27.99766	28.07112	28.08132	27.47073	27.75812	27.62506	2.113002567	-0.432064056	Higher in EMT/6 Control	P54822;E9Q242;E	P54822;E9Q242;E
28.20769	28.258	28.12139	27.80193	27.68646	27.80809	2.806130732	-0.430202484	Higher in EMT/6 Control	Q61235;B7ZNU9	Q61235;B7ZNU9
30.59335	30.62854	30.80039	30.27586	30.23078	30.24218	2.537316856	-0.424484253	Higher in EMT/6 Control	Q9DCD0	Q9DCD0
29.94	30.11148	29.94	29.59513	29.52402	29.60752	2.591224922	-0.421597799	Higher in EMT/6 Control	Q8BH78	Q8BH78
27.61807	27.80809	27.63893	27.28912	27.23535	27.2803	2.595195169	-0.420105616	Higher in EMT/6 Control	P50396;B7FAU8;D	P50396
27.63893	27.63201	27.62506	27.11148	27.24445	27.28912	2.83507519	-0.416980743	Higher in EMT/6 Control	Q60668;F6ZV59;G	Q60668;F6ZV59;G
27.34942	27.35783	27.39917	27.01903	26.92025	26.92025	3.477003487	-0.415629705	Higher in EMT/6 Control	P40237;A2AIJ1	P40237;A2AIJ1
24.92705	25.04843	24.71293	24.47383	24.50446	24.46762	1.861567821	-0.414169312	Higher in EMT/6 Control	Q921Y0;Q3UDM0	Q921Y0;Q3UDM0;
29.86821	29.71457	29.68479	29.31743	29.27808	29.44332	2.250130342	-0.409575144	Higher in EMT/6 Control	F2Z4A3;A0A087W	F2Z4A3;A0A087W
29.31527	29.35363	29.36411	28.94	28.90885	28.96774	4.247954201	-0.405476888	Higher in EMT/6 Control	Q9QYB1;E0CY42;Q	Q9QYB1
27.06085	26.99766	26.95394	26.61807	26.53297	26.64581	3.038029254	-0.405197779	Higher in EMT/6 Control	F8WHM5;Q61543	F8WHM5;Q61543
24.90885	24.82396	25.04843	24.54628	24.59263	24.43619	2.129203616	-0.402045568	Higher in EMT/6 Control	P02463;A0A1B0G	P02463;A0A1B0G
30.26472	30.45317	30.36202	29.92592	29.94	30.02168	2.515174974	-0.397438049	Higher in EMT/6 Control	Q8BT60;Q9D6C8;Q	Q8BT60
26.86231	26.82639	26.96499	26.51202	26.54628	26.42021	2.656653405	-0.391726176	Higher in EMT/6 Control	O08997	O08997
30.00838	30.19601	30.20769	29.71457	29.68646	29.83846	2.096052234	-0.390868505	Higher in EMT/6 Control	Q920E5;A0A0G2JE	Q920E5;A0A0G2JE
30.83092	30.9746	30.87555	30.48232	30.46295	30.56638	2.739532704	-0.389808655	Higher in EMT/6 Control	Q64727;A0A286Y	Q64727
29.02168	28.99496	29.10899	28.7162	28.56092	28.68311	2.575252027	-0.388463974	Higher in EMT/6 Control	Q8VCQ8;E9QA16;	Q8VCQ8;E9QA16;
30.35153	30.35153	30.40324	30.03486	30.03486	29.87848	2.666367014	-0.386037191	Higher in EMT/6 Control	Q99PT1	Q99PT1
31.68395	31.76525	31.62418	31.33031	31.25912	31.33031	2.905875726	-0.384545008	Higher in EMT/6 Control	Q9QXS1;E9Q3W4;	Q9QXS1;E9Q3W4
33.38278	33.51794	33.54121	33.0927	33.16339	33.06085	2.532044681	-0.374994914	Higher in EMT/6 Control	P17182;Q6PHC1;E	P17182;Q6PHC1

33.00838	33.05924	33.15285	32.66713	32.74535	32.70058	2.813250172	-0.369141897	Higher in EMT/6 Control	P16045	P16045
31.94349	32.09899	32.08322	31.68395	31.66289	31.69229	2.709067312	-0.362191518	Higher in EMT/6 Control	D3Z2H9;D3YVR0	D3Z2H9
31.50142	31.61982	31.54352	31.19601	31.20186	31.18423	3.300082717	-0.360883713	Higher in EMT/6 Control	P40142;A0A286YE	P40142;A0A286YE
30.19601	30.26472	30.11148	29.83996	29.80655	29.84446	2.847286833	-0.360414505	Higher in EMT/6 Control	Q8C253;P16110	Q8C253;P16110
29.99496	29.98142	30.16039	29.64065	29.6981	29.71947	2.352647898	-0.359516144	Higher in EMT/6 Control	Q3TMX0;O08992;	Q3TMX0;O08992;
29.64238	29.80193	29.68646	29.41135	29.31095	29.33458	2.500587753	-0.357962926	Higher in EMT/6 Control	P58242	P58242
28.04009	28.22159	28.04009	27.75175	27.80809	27.67976	2.121700934	-0.354059219	Higher in EMT/6 Control	Q91V35;P18052;A	Q91V35;P18052
28.74535	28.91456	28.75494	28.52402	28.41941	28.41538	2.239832464	-0.352010091	Higher in EMT/6 Control	D3Z6I8;E9Q7Q3	D3Z6I8;E9Q7Q3
31.61982	31.65864	31.54352	31.21928	31.28692	31.2814	2.994725581	-0.344790141	Higher in EMT/6 Control	A0A2C9F2D2;Q07	A0A2C9F2D2;Q07
33.87004	33.78488	33.91528	33.49429	33.50616	33.5527	2.885901927	-0.339014689	Higher in EMT/6 Control	P20152;A0A0A6YV	P20152;A0A0A6YV
27.79576	27.90311	27.87994	27.58978	27.45513	27.52402	2.571949004	-0.336622874	Higher in EMT/6 Control	Q80X71;D3Z191;D	Q80X71;D3Z191;D
30.96774	31.0414	31.0544	30.70471	30.70471	30.67135	3.445334323	-0.327591578	Higher in EMT/6 Control	Q6IRU2	Q6IRU2
26.65949	26.76446	26.82639	26.39428	26.41377	26.48155	2.35102111	-0.320246379	Higher in EMT/6 Control	A0A0G2JDI4;O091	A0A0G2JDI4;O091
28.84446	28.94559	28.83244	28.60399	28.51653	28.54259	2.710450474	-0.319787979	Higher in EMT/6 Control	Q6ZQ38;D3YWC5	Q6ZQ38
30.16039	30.11148	30.24218	29.78488	29.87262	29.89735	2.475748262	-0.319733302	Higher in EMT/6 Control	Q8BH64;D3Z7U7	Q8BH64
29.10149	29.19131	29.15074	28.84745	28.83545	28.81421	3.46815651	-0.315477371	Higher in EMT/6 Control	A0A0A6YY47;P135	A0A0A6YY47;P135
30.60222	30.72923	30.66289	30.37244	30.37244	30.30878	2.749924628	-0.313562393	Higher in EMT/6 Control	P08228	P08228
31.82335	31.80424	31.84221	31.49667	31.54812	31.49667	3.962175428	-0.30945015	Higher in EMT/6 Control	F8WIT2	F8WIT2
31.91171	31.99833	31.86821	31.63288	31.65438	31.61544	2.73216509	-0.291851044	Higher in EMT/6 Control	Q64277;A0A0R4J1	Q64277;A0A0R4J1
28.85341	28.81115	28.79885	28.5862	28.509	28.50522	3.117597195	-0.287663142	Higher in EMT/6 Control	Q06890;E9PUU2;E	Q06890;E9PUU2;E
29.09396	29.01903	29.02697	28.81115	28.76762	28.72273	2.880986205	-0.279485703	Higher in EMT/6 Control	E9PYT3;Q91YH5;C	E9PYT3;Q91YH5
32.2814	32.26752	32.35678	32.58442	32.58889	32.57768	3.265392649	0.281758626	Higher in EMT/6 RT	P08113;F7C312	P08113;F7C312
28.62157	28.67303	28.64924	28.87994	28.95116	28.98685	2.95504581	0.291366577	Higher in EMT/6 RT	P61161	P61161
33.35678	33.34361	33.40831	33.59779	33.69437	33.70471	2.776810938	0.296053569	Higher in EMT/6 RT	P20029	P20029
30.18423	30.13614	30.2535	30.5389	30.46295	30.46295	2.658596144	0.296976089	Higher in EMT/6 RT	Q99JY9;A0A087W	Q99JY9
28.56819	28.65949	28.61456	28.91171	28.91456	28.93438	3.443336421	0.306136449	Higher in EMT/6 RT	Q8K2B3;A0A1Y7V	Q8K2B3
28.29351	28.42742	28.35783	28.70636	28.67976	28.61456	2.537833234	0.307303747	Higher in EMT/6 RT	Q80ZP8;Q3TMX5;	Q80ZP8;Q3TMX5;
29.32603	29.33031	29.36202	29.61456	29.60399	29.7597	2.466519535	0.319965998	Higher in EMT/6 RT	Q9D1R9;A0A0G2J	Q9D1R9;A0A0G2J
27.18896	27.08132	27.12139	27.43141	27.42342	27.50142	2.885815299	0.321531296	Higher in EMT/6 RT	Q9CYN9	Q9CYN9
27.05051	27.1795	27.04009	27.391	27.43141	27.42342	2.657708925	0.325245539	Higher in EMT/6 RT	A2AL50;Q8C0I1;H	A2AL50;Q8C0I1;H
30.42342	30.42342	30.47267	30.73731	30.7692	30.79266	3.848769638	0.326551437	Higher in EMT/6 RT	P61358;A2A4Q0	P61358;A2A4Q0
26.42502	26.54038	26.55801	26.92025	26.77706	26.82639	2.31115308	0.333429337	Higher in EMT/6 RT	E9Q175;E9Q3L1;E	E9Q175;E9Q3L1;E
30.90455	30.95394	30.94	31.2814	31.22504	31.29242	3.702943192	0.333459218	Higher in EMT/6 RT	P62754	P62754
31.06085	31.10525	31.06085	31.44825	31.40324	31.38278	3.795094962	0.335776011	Higher in EMT/6 RT	P62281;A0A1B0G	P62281;A0A1B0G
31.28692	31.35153	31.31959	31.61544	31.66289	31.69229	3.498492261	0.337527593	Higher in EMT/6 RT	P62908;D3YV43;A	P62908;D3YV43;A
33.78488	33.69437	33.71498	34.05924	34.12232	34.02663	3.001069784	0.337991079	Higher in EMT/6 RT	P05213	P05213
31.61982	31.67976	31.6501	32.04466	32.01837	31.90455	2.732226803	0.339297613	Higher in EMT/6 RT	Q9D8E6	Q9D8E6
27.60399	27.58262	27.45513	27.80193	27.95394	27.90885	2.205927724	0.340990702	Higher in EMT/6 RT	P51655	P51655
31.13614	31.27586	31.22504	31.4919	31.61544	31.5527	2.486290052	0.341000875	Higher in EMT/6 RT	P12970;A0A140T8	P12970;A0A140T8
28.5969	28.54995	28.70965	29.01372	28.88285	28.98685	2.280686944	0.342305501	Higher in EMT/6 RT	A0A0G2JDW7;Q6	A0A0G2JDW7;Q6
31.47267	31.59779	31.47267	31.8347	31.85341	31.88285	2.833531561	0.342611949	Higher in EMT/6 RT	P25444;A0A140T8	P25444;A0A140T8
29.01105	29.21234	29.17236	29.4453	29.49381	29.4919	2.253748407	0.345085144	Higher in EMT/6 RT	O54734	O54734
29.27586	29.43936	29.30661	29.77077	29.71129	29.60045	2.124393059	0.35356013	Higher in EMT/6 RT	Q99KI0	Q99KI0
29.2803	29.38278	29.22389	29.68144	29.5862	29.69312	2.468897916	0.357931773	Higher in EMT/6 RT	P49817;H3BKG0;D	P49817;H3BKG0;D
28.51653	28.31527	28.33245	28.74214	28.78955	28.70965	2.19906437	0.359027863	Higher in EMT/6 RT	O88685;A2AGN7;	O88685;A2AGN7;
30.55728	30.6372	30.5389	30.95394	30.89012	30.96774	3.135287922	0.359474182	Higher in EMT/6 RT	P62717;A0A1D5R	P62717;A0A1D5R
24.50446	24.75939	24.65403	24.98468	25.03171	24.99766	2.080080795	0.365388234	Higher in EMT/6 RT	A0A1L1SUU4;Q9E	A0A1L1SUU4;Q9E
33.74535	33.80424	33.66289	34.09899	34.08322	34.14528	2.929916158	0.371667226	Higher in EMT/6 RT	E9PZ16	E9PZ16
27.26249	27.47073	27.22619	27.72598	27.66628	27.69975	2.073404477	0.377538045	Higher in EMT/6 RT	Q8BH95	Q8BH95
31.45317	31.30334	31.45807	31.81955	31.74535	31.80809	2.641339231	0.386135737	Higher in EMT/6 RT	P27659;A0A087W	P27659
30.33031	30.33031	30.4334	30.67135	30.71293	30.86821	2.29931721	0.386150996	Higher in EMT/6 RT	Q8BMK4	Q8BMK4
27.93157	28.18423	28.07112	28.43936	28.4746	28.44332	2.209075183	0.390120824	Higher in EMT/6 RT	Q9D051	Q9D051
31.54812	31.50142	31.51089	31.94349	31.87188	31.92592	3.965751565	0.393623988	Higher in EMT/6 RT	P47911	P47911
29.13858	29.11892	29.17712	29.46684	29.59513	29.56819	3.137159757	0.398513158	Higher in EMT/6 RT	P54071;D6RIL6;A	P54071
27.11148	26.99766	27.11148	27.391	27.56819	27.46295	2.479953848	0.400510788	Higher in EMT/6 RT	P57759;F8WJI4;F	P57759
29.81879	29.88867	29.92592	30.28692	30.2535	30.29789	3.52569325	0.401642481	Higher in EMT/6 RT	P62889	P62889
27.23535	27.13124	27.07112	27.52402	27.55362	27.56819	2.89920849	0.402709961	Higher in EMT/6 RT	E9Q800;Q8CAQ8;	E9Q800;Q8CAQ8;
28.52028	28.45513	28.48232	28.78644	29.0296	28.85341	2.241784193	0.403905869	Higher in EMT/6 RT	P46978;D3YZN5	P46978
30.34096	30.47267	30.40324	30.85341	30.73731	30.83846	2.80850175	0.404103597	Higher in EMT/6 RT	P35564	P35564
27.82639	28.00838	27.96499	28.24898	28.47073	28.29351	2.011755229	0.404486338	Higher in EMT/6 RT	Q9CY50;A0A286Y	Q9CY50;A0A286Y
31.39305	31.34096	31.40324	31.77706	31.75732	31.84595	3.631742089	0.414361318	Higher in EMT/6 RT	P97351	P97351
30.29789	30.29789	30.40324	30.79266	30.76129	30.69644	3.118541945	0.41711998	Higher in EMT/6 RT	P10605	P10605
28.77392	29.00035	28.98414	29.33671	29.26696	29.4073	2.126990583	0.417523066	Higher in EMT/6 RT	P62852;A0A1L1S	P62852;A0A1L1S
30.52028	30.56638	30.61982	30.94698	30.99496	31.03486	3.416362307	0.423440933	Higher in EMT/6 RT	P62918	P62918
27.29789	27.2803	27.44727	27.76446	27.73892	27.80809	2.785424938	0.428670883	Higher in EMT/6 RT	Q8BMS1	Q8BMS1
26.92025	26.82639	27.04009	27.29789	27.42342	27.35783	2.413467303	0.430805206	Higher in EMT/6 RT	Q91XV3	Q91XV3
30.67976	30.72923	30.75334	31.04791	31.17831	31.23649	2.715728086	0.433458964	Higher in EMT/6 RT	P14131	P14131
29.66797	29.46879	29.68646	29.94	30.12386	30.06085	2.101718594	0.433832169	Higher in EMT/6 RT	Q9CQM8;O09167	Q9CQM8;O09167
27.33245	27.3662	27.10149	27.64581	27.77706	27.67976	2.037103574	0.434165955	Higher in EMT/6 RT	E9Q8Z4;E9Q8Z9;D	E9Q8Z4;E9Q8Z9;D
26.48308	26.58978	26.4346	26.97596	26.95394	26.89735	2.987821743	0.439929326	Higher in EMT/6 RT	Q9WUM5;A0A0N	Q9WUM5
33.05278	33.09428	33.08163	33.51794	33.52962	33.51794	5.380074373	0.445603689	Higher in EMT/6 RT	P27773;F6Q404	P27773
30.17236	30.16039	30.23078	30.61105	30.52962	30.76129	2.479013909	0.446146011	Higher in EMT/6 RT	P41105;F6Z0X0	P41105
31.78097	31.78488	31.77313	32.21928	32.2135	32.25068	5.504936437	0.448156357	Higher in EMT/6 RT	P62702;V9GWY0	P62702
30.21928	30.06085	30.20769	30.64581	30.62854	30.56638	2.877142856	0.450968424	Higher in EMT/6 RT	P62245;F8WJ41;D	P62245;F8WJ41;D
29.68979	29.68144	29.87262	30.14831	30.19601	30.2535	2.539982671	0.451324463	Higher in EMT/6 RT	O55142	O55142
27.53148	27.70636	27.391	27.97049	28.04531	27.97049	2.058297344	0.452482859	Higher in EMT/6 RT	Q8QZT1	Q8QZT1
29.104	29.2007	29.0296	29.53148	29.55545	29.60929	2.941586968	0.453975677	Higher in EMT/6 RT	O70503	O70503
26.27142	26.44727	26.23535	26.69975	26.89735	26.72598	2.144771112	0.456350962	Higher in EMT/6 RT	P14602;D3YZ06	P14602;D3YZ06
29.95394	29.88722	29.86231	30.2535	30.37244	30.45317	2.694041274	0.458547592	Higher in EMT/6 RT	Q9CQX2	Q9CQX2
30.16039	30.20769	30.06085	30.66289	30.56638	30.57542	3.005012607	0.458586375	Higher in EMT/6 RT	Q60930;G3UX26;A	Q60930;G3UX26;A
25.13712	25.317	24.95837	25.60682	25.62923	25.56384	1.929530466	0.462467194	Higher in EMT/6 RT	D3YU17;Q8VCM8	D3YU17;Q8VCM8
27.43936	27.5969	27.26249	27.94838	27.83846	27.90311	1.985138526	0.463731766	Higher in EMT/6 RT	P51660	P51660
26.72598	26.75175	26.71293	27.16039	27.05051	27.37451	2.079606848	0.464916229	Higher in EMT/6 RT	H3BJU7;H3BJX8;H	H3BJU7;H3BJX8;H
27.13124	27.22619	27.06085	27.57542	27.63201	27.62506	3.114699712	0.47140185	Higher in EMT/6 RT	Q8BWT1	Q8BWT1
27.68646	27.75175	27.71293	28.17474	28.14589	28.24898	3.70785455	0.472826004	Higher in EMT/6 RT	Q8R0X7;D6REF7;D	Q8R0X7;D6REF7
26.80193	26.96499	26.95394	27.2803	27.53148	27.33245	2.158663362	0.474454244	Higher in EMT/6 RT	G3XA21;E0CZ22;D	G3XA21;E0CZ22;D
27.13124	27.18896	27.20769	27.68646	27.70636	27.56092	3.131750222	0.475282033	Higher in EMT/6 RT	P97807;H3BKG7	P97807
25.79947	25.91342	25.87173	26.2571	26.27675	26.49228	2.366378416	0.480504354	Higher in EMT/6 RT	Q06185;Q8BTB6	Q06185
22.46513	22.41442	NaN	22.80931	23.03255	22.92976	1.963610611	0.484100024	Higher in EMT/6 RT	A0A0R4J275;Q7TN	A0A0R4J275;Q7TN
30.70471	30.55728	30.67976	31.08005	31.13614	31.18423	3.055852825	0.486223857	Higher in EMT/6 RT	Q6ZWV3;I7HLV2;P	Q6ZWV3;I7HLV2;P
34.21205	34.31689	34.28967	34.76525	34.76525	34.75035	3.975965801	0.487415314	Higher in EMT/6 RT	O35639;Q3TET3;A	O35639
26.02538	25.62645	25.72858	26.2571	26.31181	26.27853	1.810869016	0.48900795	Higher in EMT/6 RT	D3Z783;P31750;D	D3Z783;P31750;D
27.66628	27.98142	27.69312	28.10649	28.32817	28.37451	1.700986459	0.489454905	Higher in EMT/6 RT	Q9JJI8	Q9JJI8
26.20769	26.20769	25.93832	26.58978	26.61807	26.61807	2.254923632	0.490738551	Higher in EMT/6 RT	Q99JY0;D3YXU1	Q99JY0;D3YXU1
29.80501	29.73409	29.94	30.17236	30.41337	30.37244	2.1688202	0.49302419	Higher in EMT/6 RT	P62830;A2A6F8	P62830
29.82941	29.7973	29.83996	30.33031	30.31959	30.29789	5.183937518	0.493703842	Higher in EMT/6 RT	P14115	P14115
26.45199	26.30835	26.37285	26.69975	26.88577	27.0296	2.045309425	0.493976593	Higher in EMT/6 RT	Q8JZK9	Q8JZK9
27.43936	27.35783	27.53148	27.81421	27.81421	28.19366	1.665377159	0.497802099	Higher in EMT/6 RT	E9PV44;O35143	E9PV44;O35143
30.02168	29.77549	30.07368	30.4919	30.36202	30.51089	2.071472708	0.497987111	Higher in EMT/6 RT	Q8VEM8;G5E902	Q8VEM8;G5E902
28.35363	28.51277	28.42742	28.90024	28.87701	29.01372	2.876798951	0.499046326	Higher in EMT/6 RT	Q8CGK3	Q8CGK3
29.77549	29.77235	30.00838	30.28692	30.27586	30.4919	2.048164329	0.499488195	Higher in EMT/6 RT	P62267	P62267
27.21697	27.44727	26.98685	27.65949	27.67976	27.82031	1.617941173	0.502824783	Higher in EMT/6 RT	Q8BH59;V9GXX9	Q8BH59
24.75939	24.58692	24.66492	25.42982	25.08537	25.00624	1.648609938	0.50340271	Higher in EMT/6 RT	E9Q151;Q8VBW1;	E9Q151;Q8VBW1;
29.11644	29.35363	29.29132	29.71947	29.75812	29.80347	2.600409314	0.506555557	Higher in EMT/6 RT	Q8BP67;F6RSK3	Q8BP67
30.77706	30.76129	30.72923	31.27031	31.27586	31.26472	5.431888378	0.514437358	Higher in EMT/6 RT	P38647	P38647
28.0296	28.34096	27.84446	28.57903	28.55362	28.62854	1.610718836	0.515389125	Higher in EMT/6 RT	Q9R045;A0A0A6Y	Q9R045
27.26249	27.15074	26.85043	27.69975	27.60399	27.51653	1.746488684	0.518874486	Higher in EMT/6 RT	A0A0A6YX18;Q8B	A0A0A6YX18;Q8B
29.49953	29.30443	29.31527	29.85341	29.91171	29.91171	2.844986962	0.5191981	Higher in EMT/6 RT	Q9CPX4;P29391;A	Q9CPX4;P29391;A
29.03223	28.86231	28.81726	29.35153	29.40527	29.52962	2.474601233	0.524873734	Higher in EMT/6 RT	Q9Z110;D3Z0B4;H	Q9Z110
25.1055	25.52253	25.1795	25.82153	25.66221	25.90426	1.638541797	0.526825587	Higher in EMT/6 RT	Q9CZ04;D3Z440;D	Q9CZ04;D3Z440;D
28.10149	27.69975	28.11644	28.44332	28.49762	28.55728	1.701193025	0.526844025	Higher in EMT/6 RT	Q9D8L3;Q62186	Q9D8L3;Q62186
26.16615	25.98468	26.37451	26.64581	26.67303	26.78955	1.919628068	0.527686437	Higher in EMT/6 RT	Q8JZU2;F6VVY4	Q8JZU2
23.09346	23.32389	23.391	23.67573	23.76951	23.94949	1.925638617	0.528795878	Higher in EMT/6 RT	Q9CXP4	Q9CXP4

32.4775	32.57768	32.65651	33.11613	33.08797	33.10212	3.273141499	0.531511943	Higher in EMT/6 RT	P14211	P14211
26.89735	27.10149	26.61807	27.35783	27.5389	27.31527	1.567632632	0.531696955	Higher in EMT/6 RT	K3W4T3;Q9Z1G4;	K3W4T3;Q9Z1G4
26.99766	27.22619	27.2803	27.57542	27.83244	27.69312	2.019775773	0.532278697	Higher in EMT/6 RT	P32921	P32921
27.49381	27.509	27.3662	27.9428	28.01372	28.01372	3.32419945	0.533742269	Higher in EMT/6 RT	P83882;A0A0A6YV	P83882;A0A0A6YV
23.88577	24.31008	24.17188	24.76446	24.54628	24.65949	1.72537795	0.5341657	Higher in EMT/6 RT	A0A140LIT2;O884	A0A140LIT2;O884
27.48616	27.35783	27.4073	27.92025	28.03486	27.90311	3.18352488	0.535642624	Higher in EMT/6 RT	Q9R0X4;Q32MW3	Q9R0X4;Q32MW3
25.51051	25.60116	25.47383	26.06291	25.99981	26.13712	3.218660528	0.538113912	Higher in EMT/6 RT	Q8C5N9;O88736	Q8C5N9;O88736
29.3662	29.61632	29.391	30.03486	29.95394	30.02168	2.549282546	0.54565239	Higher in EMT/6 RT	Q8BMD8	Q8BMD8
27.0296	27.33245	27.09144	27.91456	27.58262	27.60399	1.748488771	0.54922994	Higher in EMT/6 RT	P50518;A0A0N4S\	P50518;A0A0N4S\
28.82941	29.14346	28.89446	29.58441	29.48808	29.46879	2.26245363	0.557979584	Higher in EMT/6 RT	P62814;Q91YH6;A	P62814
26.07725	25.87643	25.90196	26.68646	26.38607	26.46295	2.153090174	0.559948603	Higher in EMT/6 RT	P56382	P56382
27.58262	28.0296	27.92592	28.60752	28.33245	28.2803	1.531386993	0.560708364	Higher in EMT/6 RT	P62900;A0A0A6Y)	P62900;A0A0A6Y
29.77392	29.91171	29.64238	30.2535	30.47267	30.28692	2.253874916	0.561693192	Higher in EMT/6 RT	P62855	P62855
25.12928	24.94503	24.90885	25.61246	25.52552	25.53148	2.797556992	0.562100728	Higher in EMT/6 RT	Q3U0V2	Q3U0V2
27.5969	27.53148	27.68646	28.14589	28.15074	28.20769	3.481541563	0.563158671	Higher in EMT/6 RT	A0A0R4J083;P511	A0A0R4J083;P511
30.28692	30.44332	30.4334	30.98821	30.94698	30.91883	3.308576212	0.563458761	Higher in EMT/6 RT	P16460	P16460
28.4512	28.79266	28.34096	29.09648	29.07368	29.11644	1.847586924	0.567258199	Higher in EMT/6 RT	P51863	P51863
25.65949	25.38443	25.22435	26.15846	25.93157	25.8811	1.684604461	0.567622503	Higher in EMT/6 RT	Q8BLF1;Q8BYQ0	Q8BLF1
31.29242	31.09899	31.27586	31.64581	31.86452	31.86821	2.393553175	0.570425034	Higher in EMT/6 RT	P99024;G3UZR1	P99024
24.79452	24.90885	24.48001	25.23535	25.23169	25.42982	1.780712873	0.571163813	Higher in EMT/6 RT	O09005	O09005
27.63201	27.56819	27.51653	28.02433	28.09144	28.31527	2.430334401	0.571435928	Higher in EMT/6 RT	P35550;A0A140LI	P35550
27.43936	27.14102	27.50142	27.83846	28.04009	27.92025	1.983144384	0.572331746	Higher in EMT/6 RT	Q9CPW4;Q3UA72	Q9CPW4;Q3UA72
36.12809	36.19564	36.08124	36.69177	36.63882	36.79217	3.290670955	0.572594961	Higher in EMT/6 RT	A0A087WR50;Q3	A0A087WR50;Q3
30.82335	30.93298	30.69644	31.38278	31.39305	31.39816	2.954298805	0.573737462	Higher in EMT/6 RT	P47738;A0A0G2J	P47738;A0A0G2J
25.92705	25.77203	26.00838	26.56674	26.48616	26.391	2.596486344	0.578810374	Higher in EMT/6 RT	P61211;F8WIB1;E	P61211;F8WIB1
28.05569	28.20769	27.94838	28.64924	28.59335	28.70636	2.673496365	0.579062144	Higher in EMT/6 RT	Q99LC5	Q99LC5
29.76762	29.33671	29.77077	30.13614	30.18423	30.31959	1.723863835	0.588286082	Higher in EMT/6 RT	P62911;P17932	P62911
27.45513	27.49381	27.2535	27.94838	27.93719	28.08639	2.577514907	0.589839935	Higher in EMT/6 RT	Q9CQN1	Q9CQN1
26.63201	26.5389	26.60399	26.87408	27.30661	27.3662	1.700233627	0.590662638	Higher in EMT/6 RT	P57746	P57746
29.96774	30.19601	30.09899	30.61982	30.71293	30.71293	2.905692086	0.594312032	Higher in EMT/6 RT	P50516;D3Z1B9;D	P50516
24.58692	24.67573	24.51051	25.26785	25.06085	25.23169	2.766167559	0.595746358	Higher in EMT/6 RT	P58021;E9PZ69	P58021;E9PZ69
25.77706	25.89966	25.85757	26.57254	NaN	26.31527	1.954069877	0.599142075	Higher in EMT/6 RT	Q78XF5	Q78XF5
27.53148	27.58978	27.53148	27.99226	28.04009	28.42342	1.918808172	0.601011912	Higher in EMT/6 RT	Q9CQQ7;A0A0G2	Q9CQQ7
25.89504	25.85519	26.21512	26.52701	26.54922	26.69975	2.057758939	0.603542328	Higher in EMT/6 RT	Q6GQT9	Q6GQT9
30.48232	30.68813	30.48232	31.16639	31.18423	31.12386	2.991136501	0.607241948	Higher in EMT/6 RT	Q3TFD0;Q9CZN7	Q3TFD0;Q9CZN7
28.37865	28.52776	28.06085	29.02433	29.00571	28.76129	1.704165061	0.608022054	Higher in EMT/6 RT	Q3V3R1	Q3V3R1
25.239	25.53148	25.42982	26.14686	25.92025	25.96279	2.280480249	0.609867732	Higher in EMT/6 RT	F8WHP8;P56135	F8WHP8;P56135
26.35111	26.06907	26.2535	26.88577	26.58978	27.0296	1.782900815	0.610490163	Higher in EMT/6 RT	Q8R5L1;O35658	Q8R5L1;O35658
27.14102	27.39917	27.11148	27.74535	27.83846	27.90311	2.40704768	0.611749649	Higher in EMT/6 RT	Q8C2Q8;Q91VR2;	Q8C2Q8;Q91VR2;
26.09346	26.1776	25.85043	26.52701	26.67303	26.76446	2.161755947	0.614339828	Higher in EMT/6 RT	Q9D2G2	Q9D2G2
28.31095	28.34096	28.11148	28.76129	29.00303	28.87408	2.471044835	0.625006994	Higher in EMT/6 RT	Q99N15;A2AFQ2;	Q99N15;A2AFQ2;
27.38278	27.391	27.09144	27.95947	27.95394	27.90885	2.54800563	0.652345657	Higher in EMT/6 RT	Q8BMF4	Q8BMF4
23.81909	24.20955	23.92252	24.55215	24.59263	24.78457	2.066793399	0.659395218	Higher in EMT/6 RT	Q9CZW5	Q9CZW5
25.98685	25.7103	25.87643	26.44253	26.53594	26.58978	2.726216536	0.664889018	Higher in EMT/6 RT	O54984;A0A1B0G	O54984
28.08639	28.21697	28.13614	28.88577	28.76129	28.79576	3.636538051	0.667772293	Higher in EMT/6 RT	A0A1L1STE6;Q9D	A0A1L1STE6;Q9D
25.52253	25.83605	26.21697	26.32903	26.61807	26.63201	1.3920395	0.66785113	Higher in EMT/6 RT	O08797	O08797
25.76446	25.61246	25.52253	26.29439	26.56092	26.05258	1.831680628	0.669485092	Higher in EMT/6 RT	E9QMK9;Q8BH86	E9QMK9;Q8BH86
29.61456	29.34096	29.63893	30.16039	30.23078	30.21928	2.625986178	0.671997706	Higher in EMT/6 RT	Q6ZWZ7;Q9CPR4;	Q6ZWZ7;Q9CPR4
31.51559	31.44825	31.44332	32.14224	32.13919	32.15738	5.028986854	0.677215576	Higher in EMT/6 RT	Q03265;D3Z6F5;D	Q03265;D3Z6F5
30.47267	30.58441	30.62854	31.20769	31.28692	31.24218	3.722270527	0.683726629	Higher in EMT/6 RT	P05202	P05202
24.83846	24.83364	24.76446	25.47692	25.48616	25.53445	4.676647999	0.686990102	Higher in EMT/6 RT	P48771	P48771
23.35111	23.01563	23.29614	23.57542	24.00196	24.16423	1.562741631	0.692910512	Higher in EMT/6 RT	Q9DBL7	Q9DBL7
31.48712	31.55728	31.57091	32.13919	32.26472	32.29242	3.680056086	0.693677266	Higher in EMT/6 RT	P56480	P56480
31.80809	31.84969	31.84221	32.48712	32.50142	32.59557	4.355034047	0.694709142	Higher in EMT/6 RT	P63038;D3Z2F2;D	P63038
26.17188	26.63201	26.06497	27.09144	26.9428	26.92025	1.725860316	0.695210775	Higher in EMT/6 RT	P97450;E9QAD6	P97450
31.18423	31.34096	31.16639	31.93649	31.92592	31.91528	3.622790242	0.69536972	Higher in EMT/6 RT	P48962;Q3V132	P48962
NaN	24.34435	24.52253	25.15267	25.31354	24.92252	1.643231763	0.696139336	Higher in EMT/6 RT	Q6PD26	Q6PD26
25.89735	25.89041	25.99981	27.01903	26.24082	26.61807	1.425588285	0.696781794	Higher in EMT/6 RT	P50637;A0A140LI	P50637
26.36453	26.317	25.87408	27.21697	26.72598	26.71293	1.429620395	0.700091044	Higher in EMT/6 RT	Q8C3X8	Q8C3X8
30.07368	30.28692	30.06085	30.82335	30.89735	30.81574	3.085233161	0.704998652	Higher in EMT/6 RT	Q9CZU6;Q80X68	Q9CZU6
29.18896	29.26919	29.21697	29.96774	29.91171	29.91171	4.710677732	0.705347697	Higher in EMT/6 RT	P26443;F7CFA5	P26443
28.52776	28.75494	28.40324	29.22849	29.23991	29.34308	2.535933267	0.708511353	Higher in EMT/6 RT	P51881	P51881
26.85043	26.57109	26.93157	27.34096	27.61807	27.53148	2.192383422	0.712472916	Higher in EMT/6 RT	P35486	P35486
24.77455	24.55801	24.34435	25.16806	25.33074	25.317	2.213878015	0.712964376	Higher in EMT/6 RT	Q8C9V5;Q8BGK0;	Q8C9V5;Q8BGK0;
25.56384	25.7024	25.69975	26.28912	26.38113	26.44253	3.441813702	0.715597153	Higher in EMT/6 RT	P03930	P03930
28.74855	28.93157	28.83244	29.60222	29.53148	29.52962	3.607540187	0.716923396	Higher in EMT/6 RT	O08749	O08749
25.77957	26.35783	26.33586	26.77706	27.05051	26.82639	1.60797637	0.726898829	Higher in EMT/6 RT	A0A0A0MQA5;P6	A0A0A0MQA5;P6
24.89504	24.91798	24.96279	25.46139	25.90656	25.59548	2.264472395	0.729206085	Higher in EMT/6 RT	P17665	P17665
28.55728	28.58978	28.48999	29.32174	29.27586	29.22849	4.281162518	0.729679108	Higher in EMT/6 RT	P29758	P29758
29.13124	29.04009	29.10649	29.80039	29.77392	29.89735	4.025560425	0.731277466	Higher in EMT/6 RT	O35129;F6QPR1;F	O35129;F6QPR1
24.65403	25.27142	24.71293	25.78955	25.6811	25.39754	1.502750915	0.743270874	Higher in EMT/6 RT	A0A0N4SVQ1;Q62	A0A0N4SVQ1;Q62
24.26069	NaN	23.80931	24.70767	24.67034	24.95837	1.444521164	0.743789355	Higher in EMT/6 RT	Q6PGL7;A0A0N4S	Q6PGL7;A0A0N4S
25.92931	26.07928	25.68913	26.87408	26.46607	26.58978	1.961291715	0.744070053	Higher in EMT/6 RT	P61804	P61804
24.33756	24.79947	24.417	24.90885	25.64031	25.239	1.363951983	0.744710286	Higher in EMT/6 RT	Q8R1I1	Q8R1I1
28.46295	28.258	28.49381	29.11644	29.08132	29.258	2.909748576	0.746999741	Higher in EMT/6 RT	Q9DB20;F6XVM5;	Q9DB20
30.19601	30.33031	30.36202	31.00169	31.0544	31.0927	3.713638203	0.753481547	Higher in EMT/6 RT	Q64433	Q64433
27.2803	27.35783	27.39917	28.11148	28.07623	28.11644	4.4658218	0.755614599	Higher in EMT/6 RT	G3X9L6;Q9DCX2;E	G3X9L6;Q9DCX2;B
27.41538	27.38278	27.18896	28.11644	28.06599	28.07623	3.32736543	0.757181168	Higher in EMT/6 RT	Q99LP6	Q99LP6
25.2856	25.38113	25.2021	26.13712	26.07928	25.95394	3.292398781	0.767170588	Higher in EMT/6 RT	Q9Z1G3	Q9Z1G3
30.62854	30.59335	30.46295	31.2814	31.34625	31.37244	3.758695449	0.771753947	Higher in EMT/6 RT	Q9QZF2	Q9QZF2
24.55215	24.98033	24.49838	25.6971	25.1946	25.46139	1.672428934	0.774076462	Higher in EMT/6 RT	Q9WTK5	Q9WTK5
24.41054	24.12534	24.80931	25.1055	24.98902	25.58405	1.350518412	0.777792613	Higher in EMT/6 RT	A0A0A6YVX4;Q8C	A0A0A6YVX4;Q8C
31.69229	31.75732	31.82714	32.55728	32.56865	32.5038	4.244060828	0.784320831	Higher in EMT/6 RT	P08249;A0A0G2J	P08249
29.45317	29.45905	29.40121	30.19601	30.30878	30.18423	4.261518366	0.791866302	Higher in EMT/6 RT	P18242;F8WIR1;F	P18242;F8WIR1;F
25.66492	25.66221	26.46918	26.64581	26.69975	26.83846	1.354700896	0.795906703	Higher in EMT/6 RT	A0A0R4J1V1;Q75	A0A0R4J1V1;Q75
22.58463	NaN	22.59605	NaN	23.51653	23.26785	1.633417397	0.801851273	Higher in EMT/6 RT	Q925I1	Q925I1
29.74053	29.66628	29.42142	30.44332	30.42342	30.38278	2.925476817	0.807098389	Higher in EMT/6 RT	P67778;Q5SQG5	P67778;Q5SQG5
25.86938	26.05466	25.55215	26.1488	26.77706	26.97596	1.309417414	0.808543523	Higher in EMT/6 RT	Q61655;Q8R3C7;(	Q61655;Q8R3C7;C
25.10949	NaN	25.61807	26.22803	26.1055	26.18707	1.594536096	0.809752782	Higher in EMT/6 RT	Q9QZM4	Q9QZM4
24.06085	NaN	24.08537	25.09346	24.98033	24.58118	1.564972128	0.811875979	Higher in EMT/6 RT	E9Q9C5;P63082;D	E9Q9C5;P63082;D
24.23169	24.51051	24.62087	25.01052	25.39428	25.39754	2.031496237	0.813090007	Higher in EMT/6 RT	J3QN51;J3QPT3;B	J3QN51;J3QPT3;B
25.85994	26.20396	25.89735	26.90885	26.87408	26.63201	2.374360997	0.817893982	Higher in EMT/6 RT	P28481	P28481
25.13712	25.16806	24.90426	25.82881	25.86938	26.00624	2.958057748	0.831666311	Higher in EMT/6 RT	Q922Q4;Q922W5	Q922Q4
25.33074	24.95837	25.10149	25.87877	25.92479	26.10149	2.551693591	0.838149389	Higher in EMT/6 RT	Q9DCW5;P43024	Q9DCW5;P43024
26.04427	25.55801	25.36786	26.56238	26.49076	26.44884	1.840487929	0.843946457	Higher in EMT/6 RT	Z4YJW0;Q8C7X2;A	Z4YJW0;Q8C7X2
23.04593	23.65403	23.49228	24.16423	24.33074	24.24627	1.971460101	0.849660873	Higher in EMT/6 RT	G3UX99;G3UZY2;A	G3UX99;G3UZY2;A
27.76446	28.29789	27.84446	29.28251	28.54628	28.63201	1.389971941	0.851328582	Higher in EMT/6 RT	P61620;A0A0A6Y\	P61620
25.55508	25.60399	25.64856	26.41054	26.73892	26.21512	2.271165322	0.852315267	Higher in EMT/6 RT	Q9Z2I9	Q9Z2I9
26.82639	26.77706	27.19835	27.28912	28.18423	27.88577	1.351614869	0.85243988	Higher in EMT/6 RT	H3BKY1;H9H9R4;(	H3BKY1;H9H9R4;C
25.56092	25.66221	25.6513	26.52103	26.60399	26.33586	3.264083832	0.862150828	Higher in EMT/6 RT	Q9CPQ1;D3Z6E1;A	Q9CPQ1;D3Z6E1
29.07368	28.32817	28.88285	29.63201	29.57181	29.67303	1.730620815	0.864051819	Higher in EMT/6 RT	Q9D0K2;Q3UJQ9	Q9D0K2;Q3UJQ9
26.61807	26.45042	26.32045	27.44727	27.1795	27.4073	2.739592937	0.881711324	Higher in EMT/6 RT	Q91YZ8;A3KFU5;A	Q91YZ8;A3KFU5;A
23.01733	23.1991	22.89874	24.01903	23.77957	24.01903	2.796465806	0.900817871	Higher in EMT/6 RT	Q9CQR4	Q9CQR4
NaN	23.417	23.49228	24.33756	24.26785	24.48001	2.749758131	0.907166799	Higher in EMT/6 RT	Q99MR3	Q99MR3
26.69975	27.00838	27.39917	27.56819	28.08639	28.22619	1.502627985	0.924485524	Higher in EMT/6 RT	P70404	P70404
22.0559	22.91525	21.79154	23.33756	23.04926	23.15962	1.246707179	0.927920024	Higher in EMT/6 RT	Q8R1L4	Q8R1L4
27.2803	26.76446	27.34096	27.90311	28.20769	28.08639	2.002611733	0.93715922	Higher in EMT/6 RT	Q8BFR5;A0A0U1R	Q8BFR5
28.00303	28.12633	28.02433	28.95947	28.92592	29.11148	3.798183545	0.947730382	Higher in EMT/6 RT	P09528	P09528
26.31354	26.42982	26.32389	27.13124	27.16039	27.63201	2.334876521	0.952128728	Higher in EMT/6 RT	Q9DB77;A0A140L	Q9DB77
24.48001	24.09346	24.10949	25.16039	24.65949	25.72598	1.340700443	0.954304377	Higher in EMT/6 RT	G5E8R3;E9QPD7;(	G5E8R3;E9QPD7;C
26.04427	26.08335	26.16423	26.9428	27.14102	27.11148	3.77221932	0.967817307	Higher in EMT/6 RT	Q791V5;A2AFW6;	Q791V5;A2AFW6;
24.44884	23.83846	24.33756	25.2535	25.02749	25.2535	2.061704514	0.969873428	Higher in EMT/6 RT	Q91V01;A0A0N4S	Q91V01
23.20359	23.28207	NaN	23.84805	24.63755	24.18707	1.342446955	0.981390953	Higher in EMT/6 RT	Q9DBD5;Q9D4T3	Q9DBD5

NaN	24.65403	24.86703	25.48308	25.98251	25.76446	1.775597059	0.98281765	Higher in EMT/6 RT	O88696	O88696
24.97596	24.57542	25.08132	25.76446	25.65403	26.20396	1.922683293	0.996583939	Higher in EMT/6 RT	Q9QXX4	Q9QXX4
30.09899	30.17236	30.14831	31.0927	31.19013	31.15436	5.023851988	1.005846024	Higher in EMT/6 RT	Q99JR5;H3BJ97;H	Q99JR5;H3BJ97
25.74919	25.78207	26.23717	27.22619	26.75175	26.85043	2.054166853	1.01997757	Higher in EMT/6 RT	Q8R2Y8	Q8R2Y8
23.88577	24.26785	23.42982	25.04009	25.21697	24.39754	1.375857128	1.023722967	Higher in EMT/6 RT	Q9DCA5;F6WEP7	Q9DCA5;F6WEP7
25.13712	25.08942	24.50446	26.11545	25.92705	25.76192	1.9660559	1.024478277	Higher in EMT/6 RT	Q9DC61;A2AIW9	Q9DC61;A2AIW9
26.37782	26.86231	26.56092	27.55362	27.74535	27.61807	2.619338814	1.038661321	Higher in EMT/6 RT	Q9CZ13;A0A0A6Y	Q9CZ13
25.47692	26.09748	25.4073	26.49228	26.60399	27.0296	1.729665848	1.048058192	Higher in EMT/6 RT	H3BKN0;Q1HFZ0	H3BKN0;Q1HFZ0
23.56384	24.42982	NaN	25.08537	24.99335	25.07725	1.32243386	1.055160522	Higher in EMT/6 RT	A0A0R3P9C8;Q9D	A0A0R3P9C8;Q9D
26.60399	25.90196	26.80193	27.60399	27.42342	27.47073	1.726524251	1.06341807	Higher in EMT/6 RT	Q8CFE6	Q8CFE6
23.97596	24.03591	24.1488	25.12534	25.02327	25.20583	3.886201747	1.064589183	Higher in EMT/6 RT	A0A087WSN6;B9E	A0A087WSN6;B9E
24.98902	25.20583	25.57542	26.42502	26.14492	26.4008	2.279492986	1.066823324	Higher in EMT/6 RT	Q9CPQ8	Q9CPQ8
24.96719	24.82396	24.35783	26.1488	26.04635	25.15653	1.366730784	1.06756719	Higher in EMT/6 RT	Q9CR60	Q9CR60
25.46139	26.17188	25.46139	26.85043	26.18707	27.27142	1.273694637	1.071419398	Higher in EMT/6 RT	Q62086	Q62086
23.11744	22.42214	NaN	23.59833	23.70767	24.24627	1.222774309	1.080967585	Higher in EMT/6 RT	Q6NS46	Q6NS46
25.04009	25.1946	25.36786	26.50446	25.97378	26.38278	2.364892644	1.086151123	Higher in EMT/6 RT	Q8BGH2	Q8BGH2
26.14686	26.63201	26.48001	27.4073	27.5389	27.57542	2.693872825	1.087584813	Higher in EMT/6 RT	Q6PB66	Q6PB66
25.72858	25.30661	25.73117	26.56529	26.48462	27.07112	2.072656488	1.118221919	Higher in EMT/6 RT	P19783;M0QWX7	P19783
25.82881	25.35448	26.40568	27.09144	26.9428	26.95394	1.674614125	1.133069356	Higher in EMT/6 RT	P12787	P12787
25.05258	25.38113	24.47383	26.03591	26.24627	26.05051	1.853141206	1.14171346	Higher in EMT/6 RT	Q921L6;Q60598	Q921L6;Q60598
28.87408	28.69975	28.78955	29.92592	29.91171	29.99496	4.472373206	1.156403859	Higher in EMT/6 RT	E9Q5G3;A0A1L1SI	E9Q5G3;A0A1L1SI
25.58405	25.43619	25.79699	26.13124	26.98685	27.16997	1.578154533	1.156942368	Higher in EMT/6 RT	Q9DC53;Q9CYR4;	Q9DC53
24.49838	24.65403	24.71293	25.76446	25.66492	25.91342	3.560342003	1.159151713	Higher in EMT/6 RT	O35988	O35988
27.66628	27.67303	27.90885	28.92875	29.0427	28.88867	3.703335334	1.203986486	Higher in EMT/6 RT	Q9WVM1;E9Q9N	Q9WVM1
23.04427	23.32389	24.61526	24.67034	25.00624	25.05672	1.178179265	1.249961217	Higher in EMT/6 RT	P10404;P11370;B	P10404
23.93157	NaN	24.17188	25.33415	25.54038	25.04009	2.03958097	1.253149668	Higher in EMT/6 RT	O88958;D3Z0R5;D	O88958;D3Z0R5
25.04009	24.62645	25.19084	26.20583	26.30487	26.16997	2.738036211	1.274429321	Higher in EMT/6 RT	Q64133	Q64133
25.52552	25.24627	25.2571	26.90885	26.57109	26.64581	3.242003561	1.365622838	Higher in EMT/6 RT	Q9D0M3	Q9D0M3
25.76446	24.40406	25.60682	26.35111	26.82639	26.81421	1.43006845	1.405456543	Higher in EMT/6 RT	Q9D881;P19536;F	Q9D881;P19536
22.2006	21.64966	21.70978	23.50446	22.66275	23.63201	1.803420463	1.413060506	Higher in EMT/6 RT	E9Q411	E9Q411
23.70767	24.39754	23.82881	25.48922	25.50142	25.41377	2.647364465	1.490131378	Higher in EMT/6 RT	E9Q3T3;L0N7N1;A	E9Q3T3;L0N7N1
28.18896	27.41538	27.16997	29.18423	29.13614	29.23991	2.17739265	1.595322291	Higher in EMT/6 RT	E9Q600;Q924C6;E	E9Q600;Q924C6
24.59263	24.64306	24.84326	26.76446	26.1946	25.90656	2.42589401	1.595553716	Higher in EMT/6 RT	D3Z7P3;D3Z7P4;F	D3Z7P3;D3Z7P4
25.78457	24.89041	25.99118	26.99766	27.16997	27.28912	1.993964218	1.596862793	Higher in EMT/6 RT	Q9CQV6;M0QWC	Q9CQV6;M0QWC
24.54628	23.77957	24.35783	25.71293	25.93607	26.26249	2.466515884	1.74260203	Higher in EMT/6 RT	P99028	P99028
26.24082	24.57542	24.32389	26.82639	26.99766	26.64581	1.360847577	1.77657636	Higher in EMT/6 RT	Q9QYA2;G3UY77	Q9QYA2
28.51653	28.69644	28.40324	30.30878	30.45317	30.18423	3.983051305	1.776655833	Higher in EMT/6 RT	Q64337;D3YZJ1;F	Q64337;D3YZJ1
23.06579	NaN	22.17035	25.22803	24.26069	23.96719	1.28748164	1.867230733	Higher in EMT/6 RT	A0A0F6AIX5;A0A1	A0A0F6AIX5;A0A1
23.29614	22.26499	24.47383	25.50749	25.26069	25.62645	1.513985594	2.119889577	Higher in EMT/6 RT	Q9CXF4	Q9CXF4
24.6971	23.33756	24.1332	26.44095	26.4567	26.239	2.356925379	2.322925568	Higher in EMT/6 RT	P52293;A2A600;A	P52293
21.85377	NaN	21.21252	24.5285	23.391	23.87643	1.792560608	2.398832003	Higher in EMT/6 RT	Q8VDU3;Q8C7I4	Q8VDU3;Q8C7I4

Protein names	Gene names	GOBP name	GOMF name	GOCC name	KEGG name
Pigment epithelium-deriv	Serpinf1	biological regulation;negative regulation of angi	endopeptidase inhibitor activity;endopeptidase	cell part;cytoplasmic membrane-bounded vesicl	
Protein prune homolog 2	Prune2	apoptosis;cell death;cellular process;death;prog	catalytic activity;hydrolase activity;hydrolase ac	cell part;cytoplasmic part;Golgi apparatus;intrac	
Arrestin domain-containin	Arrdc1				
Complement component	C1s1;C1sa	activation of immune response;biological regula	binding;calcium ion binding;catalytic activity;cat	extracellular region	Complement and coagulation cascades;Staphylc
Calcium-binding and coile	Calcoco1	biological regulation;biosynthetic process;cell su	binding;chromatin binding;DNA binding;ligand-	cell part;chromatin;chromosomal part;cytoplasn	
Telomerase protein comp	Tep1	anatomical structure homeostasis;biological reg	adenyl nucleotide binding;adenyl ribonucleotide	cell part;chromosomal part;chromosome, telom	
Extracellular superoxide d	Sod3	cellular metabolic process;cellular process;cellu	antioxidant activity;binding;catalytic activity;cat	cell part;cytoplasmic part;cytosol;extracellular n	
Protein tweety homolog 3	Ttyh3		anion channel activity;anion transmembrane tra	cell part;chloride channel complex;ion channel c	
Glutathione peroxidase;Pl	Gpx4	cellular component organization;cellular compo	antioxidant activity;catalytic activity;glutathione	cell part;cytoplasmic part;cytosol;envelope;intra	Glutathione metabolism
Leucine-rich repeat flightl	Lrrfip1	biological regulation;biosynthetic process;cell su	binding;DNA binding;nucleic acid binding	cell part;cytoplasm;intracellular membrane-bou	
Biliverdin reductase A	Blvra	catabolic process;cellular catabolic process;cellu	biliverdin reductase activity;binding;catalytic ac	cell part;cytoplasm;intracellular part	Porphyrin and chlorophyll metabolism
Gasdermin-D	Gsdmdc1	cell communication;cellular process;cellular resp			
Complement factor H	Cfh	activation of immune response;biological regula	binding;carbohydrate binding;glycosaminoglyca	extracellular region	Complement and coagulation cascades;Staphylc
Carbonic anhydrase 9;Car	Ca9;Car9	anatomical structure morphogenesis;cellular me	binding;carbonate dehydratase activity;carbon-	cell part;cell projection membrane;cell projectio	Nitrogen metabolism
Fractalkine	Cx3cl1	anatomical structure formation involved in mor	binding;chemokine activity;chemokine receptor	cell part;cell surface;extracellular region part;ex	Chemokine signaling pathway;Cytokine-cytokine
Immunoglobulin superfam	Igsf8			cell part;integral to membrane;intrinsic to mem	
Galectin;Galectin-7;Galect	Lgals7	apoptosis;cell death;cellular process;death;prog	binding;carbohydrate binding	cell part;cytoplasm;extracellular region;intracell	
Inhibitor of nuclear factor	Ikbkb	B cell homeostasis;biological regulation;homeos	adenyl nucleotide binding;adenyl ribonucleotide	CD40 receptor complex;cell part;cytoplasm;inte	Acute myeloid leukemia;Adipocytokine signaling
Teneurin-4	Tenm4	anatomical structure formation involved in mor	binding;identical protein binding;protein binding	axon;cell part;cell projection;cytoplasm;cytoplas	
High mobility group prote	Hmga2	adipose tissue development;adrenal gland deve	5'-deoxyribose-5-phosphate lyase activity;AT DN	cell part;chromatin;chromosomal part;chromoso	
Antithrombin-III	Serpinc1	biological regulation;blood coagulation;coagulat	binding;carbohydrate binding;endopeptidase in	extracellular region;extracellular region part;ext	Complement and coagulation cascades
Histone H1.3	Hist1h1d	binding of sperm to zona pellucida;cell different	binding;DNA binding;nucleic acid binding	cell part;chromatin;chromosomal part;chromoso	
Phosphoribosylformylglyc	Pfas	amine metabolic process;biosynthetic process;c	adenyl nucleotide binding;adenyl ribonucleotide	cell part;cytoplasm;intracellular part	Purine metabolism
EMILIN-1	Emilin1	biological adhesion;biological regulation;cell adh	binding;extracellular matrix constituent conferri	collagen;extracellular matrix;extracellular matrix	
Histone H1.1	Hist1h1a	cellular component assembly;cellular componen	binding;DNA binding;nucleic acid binding	cell part;chromatin;chromosomal part;intracellu	
Vascular endothelial grow	Vegfa	activation of protein kinase activity;adaptive im	binding;carbohydrate binding;chemoattractant	basement membrane;cell part;cell surface;cytop	Bladder cancer;Cytokine-cytokine receptor inter
Phosphoinositide 3-kinase	Pik3r4		adenyl nucleotide binding;adenyl ribonucleotide	cell part;cytoplasmic part;endosome;intracellula	Regulation of autophagy
Putative RNA-binding prot	Luc7l2	biological regulation;negative regulation of biolo		cell part;intracellular membrane-bounded orgar	
Twisted gastrulation prote	Twsg1	anatomical structure development;anatomical s		extracellular region part;extracellular space	
Matrix Gla protein	Mgp	anatomical structure development;biological re	binding;calcium ion binding;cation binding;ion b	extracellular matrix;extracellular region;extracel	
Histone H1.0	H1f0	cellular component assembly;cellular componen	binding;DNA binding;nucleic acid binding	cell part;chromatin;chromosomal part;cytoplasr	
EMILIN-2	Emilin2	biological adhesion;cell adhesion;cellular proces		collagen;extracellular matrix part;extracellular r	
Tetraspanin-14	Tspan14			cell part;integral to membrane;intrinsic to mem	
Collagen alpha-1(VI) chain	Col6a1	biological adhesion;cell adhesion;cellular compo		cell part;collagen;extracellular matrix;extracellu	ECM-receptor interaction;Focal adhesion;Protei
High mobility group prote	Hmga1	aging;biological regulation;biosynthetic process;	binding;DNA binding;hormone receptor binding	cell part;chromatin;chromosomal part;heteroch	
C-X-C motif chemokine 5	Cxcl5	biological regulation;chemotaxis;defense respor		extracellular region part;extracellular space	Chemokine signaling pathway;Cytokine-cytokine
A disintegrin and metallop	Adamts1	anatomical structure development;anatomical s	binding;carbohydrate binding;catalytic activity;c	basement membrane;cell part;cytoplasmic part;	
Protein BRICK1	Brk1	actin cytoskeleton organization;actin filament-b		cell part;cytoplasm;cytoskeleton;intracellular nc	Regulation of actin cytoskeleton
Histone H1.4	Hist1h1e	cellular component assembly;cellular componen	binding;DNA binding;nucleic acid binding	cell part;chromosomal part;intracellular membr	
Clathrin light chain	Clta	cellular component organization;cellular compo	binding;peptide binding;structural molecule acti	cell part;clathrin coat;clathrin coat of coated pit	Bacterial invasion of epithelial cells;Endocrine ar
Barrier-to-autointegration	Banf1	cellular macromolecule metabolic process;cellul	binding;DNA binding;nucleic acid binding	cell part;chromosome;cytoplasm;intracellular m	
Fructose-bisphosphate ald	Aldoc	aging;alcohol catabolic process;alcohol metabol	aldehyde-lyase activity;carbon-carbon lyase acti	axon;cell part;cell projection;cytoplasmic part;ir	Carbon fixation in photosynthetic organisms;Fru
Mimecan	Ogn			extracellular matrix;extracellular region part;ext	
Aly/REF export factor 2;TH	Alyref2;Alyref	cellular macromolecule metabolic process;cellul	binding;DNA binding;nucleic acid binding;nuclec	cell part;cytoplasm;intracellular membrane-bou	mRNA surveillance pathway;RNA transport;Splic
Protein-lysine 6-oxidase	Lox	anatomical structure development;blood vessel	binding;catalytic activity;cation binding;copper i	cell part;collagen;extracellular matrix part;extra	
Histone H1.5	Hist1h1b	anatomical structure development;cellular com	binding;DNA binding;nucleic acid binding	cell part;chromosomal part;intracellular membr	
Histone H1.2	Hist1h1c	cellular component assembly;cellular componen	binding;DNA binding;nucleic acid binding	cell part;chromosomal part;intracellular membr	
SLIT-ROBO Rho GTPase-ac	Srgap2	actin filament severing;actin filament-based pro	binding;enzyme activator activity;enzyme bindir	cell junction;cell part;cell projection;cytoplasmi	Axon guidance
Retinol-binding protein 1	Rbp1	biological regulation;biosynthetic process;carbo	binding;isoprenoid binding;lipid binding;retinal	cell body;cell part;cytoplasmic part;cytosol;intra	
Histone H2A;Histone H2A	H2afj	cellular component assembly;cellular componen	binding;DNA binding;nucleic acid binding	cell part;chromosomal part;intracellular membr	Systemic lupus erythematosus
Major vault protein	Mvp	cell proliferation;macromolecule metabolic proc		cell part;cytoplasm;extracellular membrane-bou	
Histone H2A type 2-C;Hist	Hist2h2ac;Hist	cellular component assembly;cellular componen	binding;DNA binding;nucleic acid binding	cell part;chromosomal part;intracellular membr	Systemic lupus erythematosus
Transcriptional activator p	Purb	apoptosis;biological regulation;biosynthetic pro	binding;DNA binding;double-stranded DNA bind	cell part;chromosomal part;DNA replication fact	
Protein transport protein	Sec16a	cellular component organization;cellular compo		cell part;cytoplasmic part;cytosol;intracellular p	
Enhancer of rudimentary	Erh	cell cycle;cellular process			
Vacuolar protein sorting-a	Vps37c	establishment of localization;establishment of p		cell part;cytoplasmic part;endosomal part;endo	Endocytosis
Histone H3.1	Hist1h3a	biological regulation;cellular component assem	binding;DNA binding;nucleic acid binding	cell part;chromosomal part;intracellular organel	Systemic lupus erythematosus
Histone H2A.V;Histone H2	H2afv;H2afz	cellular component assembly;cellular componen	binding;DNA binding;nucleic acid binding	cell part;chromosomal part;intracellular membr	Systemic lupus erythematosus
Biglycan	Bgn	amine metabolic process;aminoglycan metaboli	binding;carbohydrate binding;extracellular matr	cell part;cell surface;cytoplasmic membrane-bou	
EGF-like repeat and discoi	Edil3	biological adhesion;biological regulation;cell adh	binding;calcium ion binding;cation binding;ion b	extracellular membrane-bounded organelle;extr	
Calsyntenin-1	Clstn1	biological adhesion;biological regulation;calcium	binding;calcium ion binding;cation binding;ion b	cell junction;cell part;cell projection;cytoplasmi	
Vacuolar protein sorting-a	Vps37b	establishment of localization;establishment of p		cell part;cytoplasmic part;endosomal part;endo	Endocytosis
Transformer-2 protein ho	Tra2b	biological regulation;cellular macromolecule me	binding;mRNA binding;nucleic acid binding;nucl	cell part;intracellular membrane-bounded orgar	Spliceosome
Glycerophosphoinositol in	Gdpd2	alcohol metabolic process;alditol metabolic pro	binding;catalytic activity;cation binding;glycerop	cell part;cytoplasm;cytoskeleton;integral to mer	
C-X-C motif chemokine 3	Cxcl3	biological regulation;calcium ion homeostasis;ca	binding;chemokine activity;chemokine receptor	extracellular region part;extracellular space	
Wiskott-Aldrich syndrome	Wasf2	actin cytoskeleton organization;actin filament-b		cell part;cell projection;cytoplasmic part;cytosk	Adherens junction;Bacterial invasion of epithelia
Pre-mRNA-processing fact	Prpf19	biological regulation;biosynthetic process;cell pr	acid-amino acid ligase activity;binding;catalytic a	cell part;cytoplasmic part;cytoskeletal part;intra	Spliceosome;Ubiquitin mediated proteolysis
Insulin-like growth factor-	Igfbp7	anatomical structure development;biological ad		extracellular matrix;extracellular region;extracel	
Protein FAM177A1	Fam177a1				
Tripeptidyl-peptidase 1	Tpp1	anatomical structure development;anatomical s	binding;catalytic activity;cation binding;endopep	cell part;cytoplasmic membrane-bounded vesicl	Lysosome
High mobility group prote	Hmgb2	anatomical structure development;base-excisio	binding;chemoattractant activity;damaged DNA	cell part;chromosome;condensed chromosome;	
Calcyclin-binding protein	Cacybp	aging;biological regulation;cardiac cell differenti		beta-catenin destruction complex;cell body;cell	Wnt signaling pathway
U1 small nuclear ribonucle	Snrnp70	biological regulation;cellular macromolecule me	binding;nucleic acid binding;nucleotide binding;	cell part;intracellular organelle part;intracellular	Spliceosome
Disintegrin and metallopro	Adam10	biological regulation;cell activation;cell surface r	binding;catalytic activity;cation binding;endopep	cell part;cell surface;cytoplasmic membrane-bou	Alzheimer's disease;Epithelial cell signaling in He
Carboxypeptidase Q	Cpq	alcohol metabolic process;amine metabolic proc	binding;carboxypeptidase activity;catalytic activ	cell part;cytoplasmic part;endoplasmic reticulun	
Band 4.1-like protein 1	Epb41l1	actin cytoskeleton organization;actin filament-b	1-phosphatidylinositol binding;actin binding;bin	actin cytoskeleton;cell cortex part;cell part;corti	Tight junction
C-type lectin domain fami	Clec11a	biological regulation;positive regulation of biolo	binding;carbohydrate binding;growth factor acti	cell part;centrosome;cytoplasm;cytoplasmic par	
Histone H2B type 1-C/E/G	Hist1h2bc;Hist	cellular component assembly;cellular componen	binding;DNA binding;nucleic acid binding	cell part;chromosomal part;intracellular membr	Systemic lupus erythematosus
Tyrosine-protein kinase Te	Tec	activation of immune response;antigen receptor	adenyl nucleotide binding;adenyl ribonucleotide	cell part;cytoplasm;cytoskeleton;intracellular nc	Osteoclast differentiation;T cell receptor signalir
Lysosomal alpha-mannosi	Man2b1	alcohol metabolic process;behavior;carbohydrat	alpha-mannosidase activity;binding;carbohydrat	cell part;cytoplasmic part;intracellular membrar	Lysosome;Other glycan degradation
Clathrin heavy chain;Clath	Cltc	cation transport;cell cycle process;cellular comp	structural molecule activity	cell part;chromosomal part;clathrin coat;clathrir	Bacterial invasion of epithelial cells;Endocrine ar
Insulin-like growth factor-	Igfbp4	biological regulation;cell proliferation;defense re		extracellular region part;extracellular space	
Prefoldin 1;Prefoldin subu	Pfdn1	actin cytoskeleton organization;actin filament-b		cell part;cytoplasmic part;cytosolic part;intracel	
Complement C1r-A subco	C1ra;C1rb	activation of immune response;biological regula	binding;calcium ion binding;catalytic activity;cat	extracellular region;extracellular region part;ext	Complement and coagulation cascades;Phagoso
Bile salt export pump	Abcb11		active transmembrane transporter activity;aden	apical plasma membrane;cell junction;cell part;c	ABC transporters;Bile secretion
Sulfhydryl oxidase 1	Qsox1	biological regulation;cell redox homeostasis;cell	catalytic activity;flavin-linked sulfhydryl oxidase	cell part;cytoplasmic part;endoplasmic reticulun	
Prostaglandin F2 receptor	Ptgfrn	cellular component organization;cellular compo		cell part;cytoplasmic part;endoplasmic reticulun	
EGF-containing fibulin-like	Efemp2	biological regulation;blood coagulation;coagulat	binding;calcium ion binding;cation binding;ion b	cell part;extracellular matrix;extracellular regior	
U1 small nuclear ribonucle	Snrpa	cellular macromolecule metabolic process;cellul	binding;nucleic acid binding;nucleotide binding;	cell part;intracellular organelle part;intracellular	Spliceosome
Frizzled-2	Fzd2	anatomical structure development;anatomical s	binding;G-protein coupled receptor activity;mol	apical part of cell;cell part;cell projection memb	Basal cell carcinoma;Melanogenesis;Pathways ir
Histone H4	Hist1h4a	biological regulation;cellular component assem	binding;DNA binding;nucleic acid binding	actin cytoskeleton;cell part;chromosomal part;c	Systemic lupus erythematosus
Sorcin	Sri	biological regulation;calcium ion transport;catio	binding;calcium ion binding;cation binding;ion b	cell part;chromaffin granule membrane;contract	
Protein S100-A1;Protein S	S100a1	biological regulation;negative regulation of biolo	binding;calcium ion binding;cation binding;ion b	cell part;cytoplasmic part;endoplasmic reticulun	
Protein shisa-5	Shisa5	biological regulation;cellular process;cellular res	molecular transducer activity;signal transducer a	cell part;cytoplasmic part;endoplasmic reticulun	p53 signaling pathway
Hepatoma-derived growth	Hdgf	biological regulation;biosynthetic process;cellula	binding;carbohydrate binding;DNA binding;glyc	cell part;cytoplasm;extracellular region part;ext	
Abl interactor 1	Abi1	anatomical structure development;anatomical s	enzyme activator activity;enzyme regulator acti	cell junction;cell part;cell projection;cell projecti	
C-type lectin domain fami	Clec2d	biological regulation;cellular defense response;c	binding;carbohydrate binding;molecular transdu	cell part;external side of plasma membrane;extr	
Serine hydroxymethyltran	Shmt1	amine biosynthetic process;amine catabolic pro	aldehyde-lyase activity;amine binding;amino aci	cell part;cytoplasmic part;cytosol;intracellular m	Cyanoamino acid metabolism;Glycine, serine an
Gasdermin-E;Gasdermin E	Gsdme	apoptosis;biological regulation;cell death;cell di		cell part;cytoplasm;intracellular part	
KIF1-binding protein	Kif1bp	anatomical structure development;cell different		cell part;cytoplasmic part;intracellular membrar	
NAD(P)H dehydrogenase [	Nqo1	aging;biological regulation;cellular metabolic pr	antioxidant activity;catalytic activity;NAD(P)H de	cell body;cell part;cytoplasmic part;cytosol;intra	
Protein S100-A10	S100a10	biological regulation;cellular process;cellular res	binding;calcium ion binding;cation binding;ion b	cell part;extrinsic to membrane;extrinsic to plas	
Cell surface hyaluronidase	Tmem2	developmental process;multicellular organismal		cell part;integral to membrane;intrinsic to mem	
Disco-interacting protein 2	Dip2b		catalytic activity	cell part;intracellular membrane-bounded orgar	
Prelamin-A/C	Lmna	anatomical structure development;biological re	structural molecule activity	cell part;cytoplasmic part;cytoskeletal part;enve	Arrhythmogenic right ventricular cardiomyopath
Epidermal growth factor r	Egfr	ameboidal cell migration;anatomical structure d	adenyl nucleotide binding;adenyl ribonucleotide	apical plasma membrane;basolateral plasma me	Adherens junction;Bladder cancer;Calcium signa
Guanylate-binding proteir	Gbp2	adhesion to other organism involved in symbiot	binding;catalytic activity;GTP binding;GTPase ac	cell part;cytoplasmic part;cytoplasmic vesicle;ex	
WAP four-disulfide core d	Wfdc2	biological regulation;negative regulation of cata	endopeptidase inhibitor activity;endopeptidase	extracellular region	
Integrin alpha-6	Itga6	anatomical structure morphogenesis;biological a	binding;cation binding;ion binding;metal ion bin	basal plasma membrane;basement membrane;c	

Proteasome activator com	Psme1	antigen processing and presentation;antigen pro	endopeptidase activator activity;endopeptidase	cell part;intracellular part;macromolecular com	Antigen processing and presentation;Proteasom
Alpha-galactosidase A	Gla	biological regulation;carbohydrate catabolic p	alpha-galactosidase activity;binding;carbohydra	cell part;cytoplasmic part;extracellular region;G	Galactose metabolism;Glycerolipid metabolism;
Sorbitol dehydrogenase	Sord	alcohol biosynthetic process;alcohol catabolic p	binding;catalytic activity;cation binding;coenzy	cell part;cell projection;cilium;cytoplasmic part;	Fructose and mannose metabolism
Cysteine-rich protein 1	Crip1	biological regulation;cellular process;cellular res	AT DNA binding;binding;cation binding;DNA ber	cell part;cytoplasm;intracellular part	
Transgelin-2;Transgelin-2	Tagln2	anatomical structure development;biological reg		cell part;intracellular membrane-bounded organ	
Insulin-like growth factor-	Igfbp6	biological regulation;regulation of biological pro		cell part;cytoplasmic part;extracellular region pa	
Adenylate kinase 2, mitoc	Ak2	2'-deoxyribonucleotide metabolic process;ADP b	adenyl nucleotide binding;adenyl ribonucleotide	cell part;cytoplasmic part;cytosol;intracellular o	Purine metabolism
Matrilin-2	Matn2	anatomical structure morphogenesis;axon guida	binding;calcium ion binding;cation binding;ion b	basement membrane;extracellular matrix part;e	
Trafficking protein particle	Trappc1	cellular process;ER to Golgi vesicle-mediated tra		cell part;cis-Golgi network;cytoplasmic part;end	
Latent-transforming grow	Ltbp1	biological regulation;cell surface receptor linked	binding;calcium ion binding;cation binding;ion b	extracellular matrix;extracellular region part;pro	
Metalloproteinase inhibito	Timp2	aging;anatomical structure development;biologi	binding;cation binding;endopeptidase inhibitor	basement membrane;cell body;cell part;cell pro	
Fibulin-2	Fbln2	biological regulation;positive regulation of biolo	binding;calcium ion binding;cation binding;extra	extracellular matrix;extracellular region part;pro	
Mitochondrial import inne	Timm8a1;Timn	cellular component organization;cellular compo	binding;cation binding;ion binding;metal ion bin	cell part;cytoplasmic part;intracellular membran	
NHL repeat-containing pro	Nhlrc3				
Integrin alpha-5	Itga5	behavior;biological adhesion;biological regulatio	binding;cation binding;ion binding;metal ion bin	adherens junction;anchoring junction;cell juncti	Arrhythmogenic right ventricular cardiomyopath
Sialate O-acetylesterase	Siae		acetylesterase activity;carboxylesterase activity;	cell part;cytoplasmic part;intracellular membran	
Nck-associated protein 1	Nckap1	actin cytoskeleton organization;actin filament-b	binding;enzyme activator activity;enzyme regula	cell part;cell projection;cell projection membran	Regulation of actin cytoskeleton
Histidine triad nucleotide-	Hint1	biological regulation;biosynthetic process;catab	binding;catalytic activity;hydrolase activity;nucle	cell part;chromatin remodeling complex;cytopla	
Frizzled-1	Fzd1	anatomical structure development;anatomical s	binding;G-protein coupled receptor activity;mol	apical part of cell;cell part;cell projection memb	Basal cell carcinoma;Melanogenesis;Pathways in
Phosphoglucomutase-1;Pl	Pgm1;Pgm2	alcohol catabolic process;alcohol metabolic proc	binding;catalytic activity;cation binding;intramo	actin cytoskeleton;cell part;cytoplasmic part;cyt	Amino sugar and nucleotide sugar metabolism;G
Ceruloplasmin	Cp	biological regulation;cation homeostasis;cation	binding;catalytic activity;cation binding;copper i	extracellular region part;extracellular space	Porphyrin and chlorophyll metabolism
S-phase kinase-associated	Skp1	catabolic process;cellular catabolic process;cellu	acid-amino acid ligase activity;catalytic activity;l	cell part;Cul7-RING ubiquitin ligase complex;cull	Cell cycle;Cell cycle - yeast;Circadian rhythm - m
Eukaryotic translation init	Eif4b		binding;nucleic acid binding;nucleotide binding;		mTOR signaling pathway;RNA transport
Programmed cell death pr	Pdcd5	apoptosis;cell death;cellular process;death;prog	binding;DNA binding;nucleic acid binding	cell part;cytoplasm;intracellular membrane-bou	
Dipeptidyl peptidase 1	Ctsc	adaptive immune response;adaptive immune re	anion binding;binding;catalytic activity;chloride	cell part;cytoplasmic part;endoplasmic reticulum	Lysosome
Peroxidasin homolog	Pxdn	catabolic process;cellular catabolic process;cellu	antioxidant activity;binding;catalytic activity;cat	cell part;cytoplasmic part;endoplasmic reticulum	
Cytoplasmic FMR1-interac	Cyfip1	axon extension;biological regulation;cell growth	actin binding;actin filament binding;binding;cyto	cell junction;cell part;cell projection;cytoplasmi	Regulation of actin cytoskeleton
Platelet-activating factor a	Pla2g7	biological regulation;catabolic process;cellular c	1-alkyl-2-acetylglycerophosphocholine esterase	extracellular region part;low-density lipoprotein	Ether lipid metabolism
Mitogen-activated protein	Mapk3	anatomical structure development;anatomical s	adenyl nucleotide binding;adenyl ribonucleotide	adherens junction;anchoring junction;caveola;c	Acute myeloid leukemia;Adherens junction;Aldo
Tetraspanin-4;Tetraspanin	Tspan4			cell part;integral to membrane;intrinsic to mem	
Tetraspanin-9;Tetraspanin	Tspan9			cell part;integral to membrane;intrinsic to mem	
Macrophage migration inh	Mif	aging;biological regulation;biosynthetic process;	binding;catalytic activity;chemoattractant activi	cell part;cell surface;cytoplasm;extracellular reg	Phenylalanine metabolism;Tyrosine metabolism
Galectin-3-binding protein	Lgals3bp	biological adhesion;cell adhesion;cellular proces	cargo receptor activity;receptor activity;scaveng	cell part;extracellular matrix;extracellular memb	
Glutathione S-transferase	Gsta1;Gsta	amine metabolic process;anatomical structure d	catalytic activity;glutathione transferase activity	cell part;cytoplasm;intracellular part	Drug metabolism - cytochrome P450;Glutathion
Ubiquitin-conjugating enz	Ube2n	activation of immune response;antigen recepto	acid-amino acid ligase activity;adenyl nucleotide	cell part;cytoplasm;intracellular membrane-bou	Ubiquitin mediated proteolysis
Capping protein (Actin fila	Capg	actin filament capping;biological regulation;cell		cell part;cytoplasm;cytoplasmic membrane-bou	
Cell division control prote	Cdc42	actin cytoskeleton organization;actin filament b	binding;catalytic activity;GTP binding;GTPase ac	actin filament;apical part of cell;cell body;cell pa	Adherens junction;Axon guidance;Bacterial inva
Protein kinase C and casei	Pacsin2	anatomical structure morphogenesis;biological	binding;cytoskeletal protein binding;lipid bindin	caveola;cell part;cell projection membrane;cell	
Sec24-related gene family	Sec24d	cellular process;ER to Golgi vesicle-mediated tra	binding;cation binding;ion binding;metal ion bin	cell part;COPII vesicle coat;cytoplasmic part;cyto	Protein processing in endoplasmic reticulum
DAZ associated protein 1,	Dazap1	anatomical structure development;cell different	binding;nucleic acid binding;nucleotide binding;	cell part;cytoplasm;intracellular membrane-bou	mRNA surveillance pathway
Peptidyl-prolyl cis-trans is	Fkbp1a	anatomical structure formation involved in mor	binding;catalytic activity;cis-trans isomerase act	axon;axon part;axon terminus;cell part;cell proje	
Tumor susceptibility gene	Tsg101	biological regulation;catabolic process;cell cycle	protein binding transcription factor activity;tran	cell part;cytoplasmic part;early endosome;endo	Endocytosis
Plasminogen activator inh	Serbp1		binding;mRNA 3'-UTR binding;mRNA binding;nu	cell part;cytoplasm;cytoplasmic part;intracellula	
Carboxypeptidase E	Cpe	anatomical structure morphogenesis;biological	binding;carboxypeptidase activity;catalytic activ	cell part;cytoplasmic part;cytoplasmic vesicle m	Type I diabetes mellitus
Phospholipid scramblase 3	Plscr3	apoptosis;biological regulation;carbohydrate ho		cell part;cytoplasmic part;integral to membrane	
Pentraxin-related protein	Ptx3	biological regulation;immune effector process;in	1,3-beta-D-glucan binding;binding;carbohydrate	extracellular region	
Heterogeneous nuclear rib	Hnrnpa1	alternative nuclear mRNA splicing, via spliceoso	binding;DNA binding;nucleic acid binding;nucle	cell part;cytoplasm;intracellular membrane-bou	Spliceosome
Protein Niban	Fam129a	biological regulation;cellular process;cellular res		cell part;cytoplasm;intracellular membrane-bou	
Osteoclast-stimulating fac	Ostf1		binding;protein binding;protein domain specific	cell part;cytoplasm;intracellular part	
Collagen alpha-1(V) chain	Col5a1	anatomical structure development;anatomical s	binding;carbohydrate binding;extracellular matr	basement membrane;collagen;collagen type V;e	Amoebiasis;ECM-receptor interaction;Focal adh
Neurogenic locus notch ho	Notch2	anatomical structure development;anatomical s	binding;calcium ion binding;cation binding;ion b	cell part;cell surface;cytoplasmic part;cytosol;ex	Dorso-ventral axis formation;Notch signaling pa
RNA-binding protein EWS	Ewsr1	biological regulation;biosynthetic process;cellula	binding;cation binding;ion binding;metal ion bin	cell part;cytoplasm;intracellular membrane-bou	
Protein NDRG1	Ndrg1	biological regulation;cell activation;cellular com		adherens junction;anchoring junction;cell juncti	
Beta-galactosidase	Glb1	alcohol catabolic process;alcohol metabolic proc	beta-galactosidase activity;binding;carbohydrate	cell part;cytoplasmic part;intracellular membran	Galactose metabolism;Glycosaminoglycan degra
Extracellular matrix prote	Ecm1	anatomical structure development;anatomical s	molecular transducer activity;signal transducer	extracellular matrix;extracellular region part;ext	
Phosphoglycerate mutase	Pgam1	alcohol biosynthetic process;alcohol catabolic p	2,3-bisphosphoglycerate-dependent phosphogly	cell part;cytoplasmic part;cytosol;intracellular m	Glycolysis / Gluconeogenesis;Methane metaboli
Phosphatidylethanolamin	Pebp1	aging;anatomical structure development;behavi	adenyl nucleotide binding;adenyl ribonucleotide	apical part of cell;axon part;axon terminus;cell b	
Gelsolin (Fragment)	Gsn	actin cytoskeleton organization;actin filament o		actin cytoskeleton;cell part;cell projection;cytop	
Phosphoglycerate kinase 1	Pgk1	alcohol catabolic process;alcohol metabolic proc	adenyl nucleotide binding;adenyl ribonucleotide	cell part;cytoplasm;intracellular part	Carbon fixation in photosynthetic organisms;Gly
Heterogeneous nuclear rib	Hnrnpab	anatomical structure morphogenesis;biological	binding;DNA binding;nucleic acid binding;nucle	cell part;cytoplasm;intracellular membrane-bou	
Programmed cell death 6-	Pdcd6ip	apoptosis;cell cycle;cell death;cell division;cellul		cell part;cytoplasmic membrane-bounded vesicl	Endocytosis
Polyadenylate-binding pro	Paip1	cellular macromolecule metabolic process;cellul	binding;DNA binding;nucleic acid binding;RNA b	cell part;cytoplasm;intracellular part	RNA transport
Phosphoserine aminotran	Psat1	amine biosynthetic process;amine metabolic pro	binding;catalytic activity;cofactor binding;O-pho		Glycine, serine and threonine metabolism;Meth
Peptidyl-prolyl cis-trans is	Ppia	biological regulation;cellular macromolecule me	binding;catalytic activity;cis-trans isomerase act	cell part;cytoplasmic part;cytosol;extracellular r	
Alpha-N-acetylglucosamin	Naglu	anatomical structure development;anatomical s			Glycosaminoglycan degradation;Lysosome
Agrin	Agrn	biological regulation;cell surface receptor linked	acetylcholine receptor regulator activity;binding	basal lamina;cell junction;cell part;cell surface;c	ECM-receptor interaction
Niban-like protein 1	Fam129b	biological regulation;negative regulation of apo	binding;lipid binding;phospholipid binding	adherens junction;anchoring junction;cell juncti	
Glutathione S-transferase	Gstm1	benzene-containing compound metabolic proce	binding;catalytic activity;glutathione transferase	cell part;cytoplasm;cytoplasmic part;cytosol;intr	Drug metabolism - cytochrome P450;Glutathion
Protein ABHD14B;Protein	Abhd14b		catalytic activity;hydrolase activity	cell part;cytoplasm;intracellular membrane-bou	
Nucleobindin-1;Nucleobin	Nucb1		binding;calcium ion binding;cation binding;DNA	cell part;cytoplasmic part;cytoskeleton;endoplas	
Fructose-bisphosphate ald	Aldoa	alcohol catabolic process;alcohol metabolic proc	aldehyde-lyase activity;binding;carbohydrate bi	cell part;centriolar satellite;contractile fiber par	Carbon fixation in photosynthetic organisms;Fru
Proteasome activator com	Psme2			cell part;intracellular part;macromolecular com	Antigen processing and presentation;Proteasom
Platelet-activating factor a	Pafah1b3	anatomical structure development;brain develo	1-alkyl-2-acetylglycerophosphocholine esterase	cell part;cytoplasm;cytoplasmic part;cytosol;intr	Ether lipid metabolism
AHNAK nucleoprotein (de	Ahnak			cell junction;cell-cell junction	
Triosephosphate isomeras	Tpi1	alcohol biosynthetic process;alcohol catabolic p	catalytic activity;intramolecular oxidoreductase		Carbon fixation in photosynthetic organisms;Fru
Thioredoxin domain-conta	Txndc17	biological regulation;cell surface receptor linked	antioxidant activity;catalytic activity;electron ca	cell part;cytoplasmic part;cytosol;intracellular pa	
6-phosphogluconolactona	Pgls	alcohol catabolic process;alcohol metabolic proc	6-phosphogluconolactonase activity;binding;car	cell part;cytoplasm;intracellular part	Pentose phosphate pathway
Collagen alpha-1(XII) chair	Col12a1	biological adhesion;cell adhesion;cellular proces		collagen;extracellular matrix part;extracellular r	
Vacuolar protein sorting-a	Vps28	biological regulation;catabolic process;cellular c		cell part;cytoplasmic part;cytosol;endosomal pa	Endocytosis
Na(+)/H(+) exchange regu	Slc9a3r1	acid secretion;actin cytoskeleton organization;a	binding;channel regulator activity;chloride chan	apical plasma membrane;brush border membra	
Platelet-derived growth fa	Pdgfc	activation of protein kinase activity;activation of	binding;growth factor receptor binding;platelet-	cell part;cytoplasmic part;extracellular region;extracel	Cytokine-cytokine receptor interaction;Focal adh
Amyloid-beta A4 protein	App	adult behavior;adult locomotory behavior;alcoh	binding;carbohydrate binding;cation binding;DN	apical part of cell;axon;cell part;cell projection;c	Alzheimer's disease
Protein kinase C and casei	Pacsin3	biological regulation;cellular component organiz	binding;calcium channel inhibitor activity;calciu	cell part;cytoplasm;intracellular part;membrane	
Antileukoproteinase	Slpi	biological regulation;negative regulation of bind	endopeptidase inhibitor activity;endopeptidase	extracellular matrix;extracellular region;extracel	
Pituitary tumor-transform	Pttg1ip	establishment of localization;establishment of lo		cell part;cytoplasm;integral to membrane;intrac	
Heterogeneous nuclear rib	Hnrnpa2b1	biological regulation;cellular macromolecule me	binding;DNA binding;nucleic acid binding;nucle	catalytic step 2 spliceosome;cell part;cytoplasm;	
Flotillin-1	Flot1	anatomical structure morphogenesis;axonogene		cell part;centriolar satellite;cytoplasmic membra	Insulin signaling pathway
Ribonuclease 4	Rnase4	cellular metabolic process;cellular nitrogen com	binding;catalytic activity;endonuclease activity;e	extracellular region	
Transaldolase	Taldo1	alcohol metabolic process;carbohydrate metabo	binding;carbohydrate binding;catalytic activity;n	cell part;cytoplasmic part;cytosol;intracellular m	Pentose phosphate pathway
Eukaryotic translation init	Eif3g	biological regulation;cellular component assemb	binding;nucleic acid binding;nucleotide binding;	cell part;cytoplasmic part;eukaryotic 43S preinit	RNA transport
Lactoylglutathione lyase	Glo1	amine metabolic process;biological regulation;ca	binding;carbon-sulfur lyase activity;catalytic acti		MAPK signaling pathway - yeast;Pyruvate metab
L-lactate dehydrogenase;L	Ldha	alcohol catabolic process;alcohol metabolic proc	catalytic activity;lactate dehydrogenase activity;	cell part;cell projection;cilium;cytoplasm;cytopla	Cysteine and methionine metabolism;Glycolysis
AP-2 complex subunit sigr	Ap2s1	cellular process;establishment of localization;es	protein transporter activity;substrate-specific tr	AP-2 adaptor complex;AP-type membrane coat	Endocrine and other factor-regulated calcium re
Adenylosuccinate lyase	Adsl	aerobic respiration;AMP biosynthetic process;Al	(S)-2-(5-amino-1-(5-phospho-D-ribosyl)imidazol	cell part;cytoplasmic part;cytosol;intracellular m	Alanine, aspartate and glutamate metabolism;P
Beta-2-syntrophin	Sntb2		binding;lipid binding;phospholipid binding	cell junction;cell part;cytoplasmic part;cytoplasr	
6-phosphogluconate dehy	Pgd	alcohol biosynthetic process;alcohol catabolic p	binding;catalytic activity;coenzyme binding;cofa	cell part;cytoplasm;intracellular part	Glutathione metabolism;Pentose phosphate pat
Reticulon	Rtn4	anatomical structure development;anatomical s		cell body;cell part;cell projection;cytoplasmic pa	
Rab GDP dissociation inhil	Gdi1	biological regulation;establishment of localizatio	enzyme activator activity;enzyme regulator activ	cell part;cell projection;cytoplasm;intracellular p	
Heterogeneous nuclear rib	Hnrnpd	biological regulation;biosynthetic process;cellula	binding;DNA binding;nucleic acid binding;nucle	cell part;cytoplasm;intracellular membrane-bou	
CD82 antigen;Tetraspanin	Cd82			cell part;integral to membrane;intrinsic to mem	p53 signaling pathway
MOB kinase activator 1A;M	Mob1a;Mob1b	biological regulation;cellular macromolecule me	binding;cation binding;enzyme activator activity	cell part;cytoplasm;intracellular membrane-bou	Cell cycle - yeast
FAT atypical cadherin 1	Fat1	actin cytoskeleton organization;actin filament o	binding;calcium ion binding;cation binding;ion b	cell junction;cell part;cell projection;cell-cell jun	
Chloride intracellular char	Clic4	anatomical structure formation involved in mor	anion channel activity;anion transmembrane tra	actin cytoskeleton;apical part of cell;cell junctio	
Golgi apparatus protein 1	Glg1	anatomical structure morphogenesis;biological		cell part;cytoplasmic part;extracellular matrix;ex	Cell adhesion molecules (CAMs)
Collagen alpha-1(IV) chain	Col4a1	anatomical structure formation involved in mor	extracellular matrix structural constituent;struct	collagen;collagen type IV;extracellular matrix pa	Amoebiasis;ECM-receptor interaction;Focal adh
Copine-3	Cpne3		binding;catalytic activity;kinase activity;lipid bin	axon;cell part;cell projection;cytoplasmic part;cy	
Copper transport protein	Atox1	biological regulation;cation homeostasis;cation	binding;cation binding;copper chaperone activit		Mineral absorption
Farnesyl pyrophosphate s	Fdps;Gm4373	alcohol biosynthetic process;alcohol metabolic p	binding;catalytic activity;cation binding;dimethy	cell part;cytoplasmic part;intracellular membran	Terpenoid backbone biosynthesis
Vinculin	Vcl	adherens junction assembly;adherens junction o	structural molecule activity	actin cytoskeleton;adherens junction;anchoring	Adherens junction;Amoebiasis;Bacterial invasion
Caldesmon 1	Cald1	biological regulation;multicellular organismal pr	actin binding;binding;cytoskeletal protein bindir	actin cap;cell cortex part;cell part;cytoplasmic p	Vascular smooth muscle contraction
Rho GDP-dissociation inhi	Arhgdia	biological regulation;cellular process;cellular res	enzyme activator activity;enzyme regulator activ	cell part;cytoplasm;immunological synapse;intra	Neurotrophin signaling pathway;Vasopressin-re
Plectin	Plec	cell junction assembly;cell junction organization		adherens junction;anchoring junction;cell juncti	
Alpha-enolase	Eno1	alcohol catabolic process;alcohol metabolic proc	binding;carbon-oxygen lyase activity;catalytic ac	cell part;cytoplasm;cytoplasmic part;cytosolic p	Glycolysis / Gluconeogenesis;Methane metaboli

Galectin-1	Lgals1	B cell activation;B cell activation involved in imm	binding;carbohydrate binding;disaccharide bind	cell part;cell surface;cytoplasm;extracellular ma	
Tropomyosin 3, related se	Tpm3-rs7	anatomical structure development;brain develo		actin filament bundle;actomyosin;cell cortex pa	
Transketolase;Transketola	Tkt	biological regulation;regulation of biological pro	binding;catalytic activity;cation binding;ion bind	cell part;intracellular membrane-bounded orga	Biosynthesis of ansamycins;Carbon fixation in ph
Galectin;Galectin-3	Lgals3	anatomical structure development;cell different	binding;carbohydrate binding	cell part;cytoplasm;cytoplasmic part;extracellula	
MCG4375, isoform CRA_b	Sdcbp	biological regulation;cellular process;cellular res	binding;cytokine receptor binding;growth factor	cell part;cytoplasm;cytoplasmic membrane-bou	
Acid sphingomyelinase-lik	Smpdl3b	catabolic process;cellular catabolic process;cellu	catalytic activity;hydrolase activity;hydrolase ac	extracellular region part;extracellular space	
Receptor-type tyrosine-pr	Ptpra	biological regulation;cell surface receptor linked	binding;catalytic activity;hydrolase activity;hydr	cell part;cytoplasm;cytoskeleton;integral to me	
Tropomyosin alpha-3 chai	Tpm3			actin filament bundle;actomyosin;cell cortex pa	
Annexin A7	Anxa7	behavior;behavioral interaction between organi	binding;calcium ion binding;calcium-dependent	cell part;chromaffin granule membrane;cytoplas	
Vimentin	Vim	anatomical structure development;anatomical s	structural constituent of cytoskeleton;structural	adherens junction;anchoring junction;axon;axon	Amyotrophic lateral sclerosis (ALS);Arrhythmoge
Transmembrane protein 1	Tmem106b			cell part;cytoplasmic part;endosomal part;endos	
Tropomyosin alpha-4 chai	Tpm4	response to chemical stimulus;response to oxida	binding;cation binding;ion binding;metal ion bin	actin filament bundle;actomyosin;cell cortex pa	Cardiac muscle contraction;Dilated cardiomyopa
Glutamate--cysteine ligase	Gclm	amine biosynthetic process;amine metabolic pro	acid-amino acid ligase activity;binding;catalytic a	cell part;cytoplasmic part;glutamate-cysteine lig	Glutathione metabolism
Cullin-associated NEDD8-d	Cand1	biological regulation;cell differentiation;cellular		cell part;cullin-RING ubiquitin ligase complex;cy	
EH domain-containing pro	Ehd2	actin cytoskeleton organization;actin filament-b	adenyl nucleotide binding;adenyl ribonucleotide	cell part;cytoplasmic part;endosomal part;endo	Endocytosis
Neural cell adhesion mole	Ncam1	biological adhesion;biological regulation;cell adh	binding;carbohydrate binding;glycosaminoglyca	anchored to membrane;axon;cell body;cell part	Cell adhesion molecules (CAMs);Prion diseases
Superoxide dismutase [Cu	Sod1	activation of MAPK activity;aging;amine metabo	antioxidant activity;binding;catalytic activity;cat	cell body;cell part;cell projection cytoplasm;cell	Amyotrophic lateral sclerosis (ALS);Huntington's
Annexin	Anxa6	biological regulation;calcium ion transport;catio	binding;calcium ion binding;calcium-dependent	cell part;cytoplasmic part;intracellular part;peri	
ADP-ribosyl cyclase/cyclic	Bst1		catalytic activity;hydrolase activity;hydrolase ac	anchored to membrane;cell part;intrinsic to me	Calcium signaling pathway;Nicotinate and nicoti
Clusterin;Clusterin (Fragm	Clu	biological regulation;cell death;cellular macrom	binding;enzyme binding;misfolded protein bindi	cell part;chromaffin granule;cytoplasmic membr	
Atlastin-3	Atl3	anatomical structure morphogenesis;axonogene	binding;catalytic activity;GTP binding;GTPase ac	axon;cell part;cell projection;cytoplasmic part;e	
Endoplasmin;Endoplasmin	Hsp90b1	actin cytoskeleton organization;actin filament o	adenyl nucleotide binding;adenyl ribonucleotide	cell part;cytoplasmic membrane-bounded vesic	NOD-like receptor signaling pathway;Pathways i
Actin-related protein 2	Actr2	actin cytoskeleton organization;actin filament o	adenyl nucleotide binding;adenyl ribonucleotide	actin cap;Arp2/3 protein complex;cell cortex pa	
78 kDa glucose-regulated	Hspa5	activation of signaling protein activity involved i	adenyl nucleotide binding;adenyl ribonucleotide	cell part;cell surface;cytoplasmic membrane-bo	Prion diseases;Protein export;Protein processing
Actin-related protein 3	Actr3	actin cytoskeleton organization;actin filament o	adenyl nucleotide binding;adenyl ribonucleotide	Arp2/3 protein complex;cell junction;cell part;c	
Succinate dehydrogenase	Sdha	acetyl-CoA catabolic process;acetyl-CoA metabo	binding;catalytic activity;coenzyme binding;cofa	cell part;cytoplasmic part;fumarate reductase c	Alzheimer's disease;Citrate cycle (TCA cycle);Hu
Armet protein;Arginine-ri	Manf	biological regulation;circulatory system process;		cell part;cytoplasmic part;extracellular region;ex	
60S ribosomal protein L34	Rpl34	biosynthetic process;cellular biosynthetic proces	structural constituent of ribosome;structural m	cell part;cytoplasmic part;intracellular non-mem	Ribosome
Renin receptor	Atp6ap2	angiotensin maturation;biological regulation;ho	receptor activity	cell body;cell part;cell projection;external side o	
Alkylglycerone-phosphate	Agps	biosynthetic process;cellular biosynthetic proces	alkylglycerone-phosphate synthase activity;bind	cell part;cytoplasmic part;intracellular membra	Ether lipid metabolism;Peroxisome
60S ribosomal protein L27	Rpl27	biosynthetic process;cellular biosynthetic proces	structural constituent of ribosome;structural m	cell part;cytoplasmic part;cytosolic large riboso	Ribosome
Unconventional myosin-V	Myo6	anatomical structure development;anatomical s	actin binding;actin filament binding;adenyl nucl	axon;cell body;cell part;cell projection;clathrin-c	
40S ribosomal protein S6	Rps6	biological regulation;biosynthetic process;carbo	structural constituent of ribosome;structural m	cell part;intracellular non-membrane-bounded o	Insulin signaling pathway;mTOR signaling pathw
40S ribosomal protein S11	Rps11	biosynthetic process;cellular biosynthetic proces	binding;nucleic acid binding;RNA binding;rRNA b	cell part;cytosolic small ribosomal subunit;intra	Ribosome
40S ribosomal protein S3	Rps3	biosynthetic process;cellular biosynthetic proces	binding;nucleic acid binding;RNA binding;struct	cell part;cytoplasm;intracellular organelle part;i	Ribosome
Tubulin alpha-1B chain	Tuba1b	cellular component assembly;cellular componen	binding;catalytic activity;GTP binding;GTPase ac	cell part;cytoplasmic microtubule;cytoplasmic p	Gap junction;Pathogenic Escherichia coli infecti
60S ribosomal protein L4	Rpl4	biosynthetic process;cellular biosynthetic proces	structural constituent of ribosome;structural m	cell part;cytosolic large ribosomal subunit;intra	Ribosome
Glypican-4	Gpc4			anchored to membrane;cell part;cytoplasmic pa	
60S ribosomal protein L7a	Rpl7a;Rpl7a-p	cellular component biogenesis;cellular compone		cell part;cytoplasmic part;intracellular non-mem	Ribosome
40S ribosomal protein S27	Rps27	biosynthetic process;cellular biosynthetic proces	binding;cation binding;ion binding;metal ion bin	cell part;cytosolic small ribosomal subunit;intra	Ribosome
40S ribosomal protein S2;	Rps2;Rps2-ps6	biosynthetic process;cellular biosynthetic proces	binding;nucleic acid binding;RNA binding;struct	cell part;intracellular organelle part;intracellula	Ribosome
Dolichyl-diphosphooligosa	Ddost	carbohydrate metabolic process;cell activation;c	catalytic activity;dolichyl-diphosphooligosacchar	cell part;cytoplasmic part;endoplasmic reticulu	N-Glycan biosynthesis;Protein processing in end
Aconitate hydratase, mito	Aco2	acetyl-CoA catabolic process;acetyl-CoA metab	3 iron, 4 sulfur cluster binding;4 iron, 4 sulfur cl	cell part;cytoplasmic part;intracellular membra	Carbon fixation pathways in prokaryotes;Citrate
Caveolin-1;Caveolin (Frag	Cav1	acylglycerol metabolic process;anatomical struc	enzyme activator activity;enzyme regulator acti	acrosomal membrane;apical plasma membrane	Bacterial invasion of epithelial cells;Endocytosis;
26S proteasome regulator	Psmc3	anatomical structure development;blastocyst de	adenyl nucleotide binding;adenyl ribonucleotide	cell part;cytoplasm;cytoplasmic mRNA processin	Proteasome
60S ribosomal protein L18	Rpl18a	biosynthetic process;cellular biosynthetic proces	structural constituent of ribosome;structural m	cell part;cytosolic large ribosomal subunit;intra	Ribosome
Secretory carrier-associat	Scamp2	establishment of localization;establishment of p		cell part;cytoplasmic part;endosomal part;endo	
Basement membrane-spe	Hspg2	anatomical structure development;anatomical s		basal lamina;extracellular matrix part;extracellu	ECM-receptor interaction
Enoyl-CoA hydratase, mito	Echs1	carboxylic acid catabolic process;carboxylic acid	carbon-oxygen lyase activity;catalytic activity;en	cell part;cytoplasmic part;intracellular membra	Aminobenzoate degradation;Benzoate degradat
60S ribosomal protein L3	Rpl3	biosynthetic process;cellular biosynthetic proces	structural constituent of ribosome;structural m	cell part;cytosolic large ribosomal subunit;intra	Ribosome
Cytoskeleton-associated p	Ckap4			cell part;cytoplasmic part;cytoskeleton;endopla	Protein processing in endoplasmic reticulum
Pyruvate dehydrogenase	Pdhb	acetyl-CoA biosynthetic process;acetyl-CoA bios	catalytic activity;oxidoreductase activity;oxidore	cell part;cytoplasmic part;intracellular membra	Butanoate metabolism;Citrate cycle (TCA cycle);
60S ribosomal protein L6	Rpl6	biosynthetic process;cellular biosynthetic proces	structural constituent of ribosome;structural m	cell part;cytoplasm;cytoplasmic part;intracellula	Ribosome
Isocitrate dehydrogenase	Idh2	2-oxoglutarate metabolic process;acetyl-CoA ca	binding;catalytic activity;cation binding;coenzym	cell part;cytoplasmic part;intracellular organelle	Carbon fixation pathways in prokaryotes;Citrate
Endoplasmic reticulum re	Erp29	cellular process;establishment of localization;es		cell part;cell surface;cytoplasmic membrane-bo	Protein processing in endoplasmic reticulum
60S ribosomal protein L30	Rpl30	biosynthetic process;cellular biosynthetic proces	structural constituent of ribosome;structural m	cell part;cytosolic large ribosomal subunit;intra	Ribosome
MICOS complex subunit M	Immt	biological regulation;calcium ion homeostasis;ca		cell part;cytoplasmic part;integral to membrane	
Dolichyl-diphosphooligosa	Stt3a	carbohydrate metabolic process;cellular carboh	catalytic activity;dolichyl-diphosphooligosacchar	cell part;cytoplasmic part;endoplasmic reticulu	N-Glycan biosynthesis;Protein processing in end
Calnexin	Canx	aging;cellular component organization;cellular c	binding;calcium ion binding;cation binding;ion b	axon;cell body;cell part;cell projection;cell proje	Antigen processing and presentation;Phagosom
Translocon-associated pro	Ssr1			cell part;cytoplasmic part;endoplasmic reticulu	Protein processing in endoplasmic reticulum
40S ribosomal protein S3a	Rps3a	biosynthetic process;cell differentiation;cellular	structural constituent of ribosome;structural m	cell part;cytoplasmic part;cytosol;cytosolic small	Ribosome
Cathepsin B	Ctsb	biological regulation;cellular process;cellular res	catalytic activity;cysteine-type endopeptidase a	cell part;cytoplasmic membrane-bounded vesic	Antigen processing and presentation;Lysosome
40S ribosomal protein S25	Rps25	cellular component assembly;cellular componen	structural constituent of ribosome;structural m	cell part;cytosolic small ribosomal subunit;intra	Ribosome
60S ribosomal protein L8	Rpl8	biosynthetic process;cellular biosynthetic proces	binding;nucleic acid binding;RNA binding;rRNA b	cell part;cytosolic large ribosomal subunit;intra	Ribosome
Trifunctional enzyme subu	Hadha	carboxylic acid catabolic process;carboxylic acid	3-hydroxyacyl-CoA dehydrogenase activity;acety	cell part;cytoplasmic part;fatty acid beta-oxidati	Aminobenzoate degradation;Benzoate degradat
Brain acid soluble protein	Basp1	biological regulation;cell differentiation;cell diffe	binding;DNA binding;nucleic acid binding;protei	cell part;cell projection part;cytoplasm;growth c	
40S ribosomal protein S16	Rps16	biosynthetic process;cellular biosynthetic proces	binding;nucleic acid binding;RNA binding;struct	cell part;cytosolic small ribosomal subunit;intra	Ribosome
60S ribosomal protein L21	Rpl21	biosynthetic process;cellular biosynthetic proces	structural constituent of ribosome;structural m	cell part;cytoplasmic part;intracellular non-mem	Ribosome
Catenin delta-1	Ctnnd1	anatomical structure morphogenesis;biological a		adherens junction;anchoring junction;cell juncti	Adherens junction;Leukocyte transendothelial m
Succinate--CoA ligase [AD	Suclg1	acetyl-CoA catabolic process;acetyl-CoA metab	acid-thiol ligase activity;ATP citrate synthase act	cell part;cytoplasmic part;intracellular organelle	Citrate cycle (TCA cycle);Propanoate metabolism
Protein disulfide-isomeras	Pdia3	biological regulation;cell redox homeostasis;cell	catalytic activity;disulfide oxidoreductase activit	cell part;cell surface;cytoplasmic membrane-bo	Antigen processing and presentation;Protein pro
60S ribosomal protein L28	Rpl28	biosynthetic process;cellular biosynthetic proces	structural constituent of ribosome;structural m	cell part;cytoplasmic part;intracellular non-mem	Ribosome
40S ribosomal protein S4,	Rps4x	biological regulation;biosynthetic process;cellula	binding;nucleic acid binding;RNA binding;rRNA b	cell part;intracellular organelle part;intracellula	Ribosome
40S ribosomal protein S15	Rps15a	biological regulation;biosynthetic process;cellula	structural constituent of ribosome;structural m	cell part;cytosolic small ribosomal subunit;intra	Ribosome
60S ribosomal protein L35	Rpl35a	biosynthetic process;cellular biosynthetic proces	structural constituent of ribosome;structural m	cell part;cytosolic large ribosomal subunit;intra	Ribosome
Acetyl-CoA acetyltransfera	Acat1	adipose tissue development;anatomical structur	acetyl-CoA C-acetyltransferase activity;acetyl-Co	cell part;cytoplasmic part;intracellular organelle	Benzoate degradation;Butanoate metabolism;Ca
Very-long-chain 3-oxoacyl	Hsd17b12	biological regulation;biosynthetic process;carbo	binding;carbohydrate binding;catalytic activity;c	cell part;cytoplasmic part;endoplasmic reticulu	Biosynthesis of unsaturated fatty acids;Steroid h
Heat shock protein beta-1	Hspb1	biological regulation;cell surface receptor linked	binding;enzyme binding;enzyme inhibitor activit	cell part;cell surface;contractile fiber part;cytop	Amoebiasis;MAPK signaling pathway;VEGF signa
Cytochrome b5 type B	Cyb5b	cellular metabolic process;cellular process;elect	binding;cation binding;enzyme activator activity	cell part;cytoplasmic part;integral to membrane	
Voltage-dependent anion-	Vdac2	biological regulation;cellular process;establishm	anion channel activity;anion transmembrane tra	cell part;cytoplasmic part;intracellular non-mem	Calcium signaling pathway;Huntington's disease
Nicalin	Ncln	biological regulation;macromolecule metabolic	catalytic activity;hydrolase activity;peptidase ac	cell part;cytoplasmic part;endoplasmic reticulu	
Peroxisomal multifunction	Hsd17b4	anatomical structure development;carboxylic ac	3alpha,7alpha,12alpha-trihydroxy-5beta-cholest	cell part;cytoplasmic part;intracellular membra	Peroxisome;Primary bile acid biosynthesis
Rho guanine nucleotide e	Arhgef2	actin cytoskeleton organization;actin filament o	binding;cation binding;cytoskeletal protein bind	cell body;cell junction;cell part;cell projection m	Pathogenic Escherichia coli infection
3-ketoacyl-CoA thiolase, m	Acaa2	biological regulation;carboxylic acid metabolic p	acetyl-CoA C-acyltransferase activity;C-acyltrans	cell part;cytoplasmic part;intracellular organelle	Benzoate degradation;Fatty acid elongation in m
Sphingosine-1-phosphate	Sgpl1	ameboidal cell migration;anatomical structure d	aldehyde-lyase activity;binding;carbon-carbon l	cell part;cytoplasmic part;endoplasmic reticulu	Sphingolipid metabolism
MCG134445, isoform CRA	Mroh1				
Fumarate hydratase, mito	Fh	acetyl-CoA catabolic process;acetyl-CoA metabo	carbon-oxygen lyase activity;catalytic activity;fu	cell part;cytoplasmic part;intracellular membra	Carbon fixation pathways in prokaryotes;Citrate
ATP synthase subunit e, m	Atp5i	ATP biosynthetic process;ATP metabolic process	ATPase activity;catalytic activity;cation transme	cell part;cytoplasmic part;intracellular membra	Oxidative phosphorylation
NADH dehydrogenase [ub	Ndufa12	cellular metabolic process;cellular process;elect	catalytic activity;electron carrier activity;NADH	cell part;cytoplasmic part;intracellular membra	Alzheimer's disease;Huntington's disease;Oxidat
60S ribosomal protein L10	Rpl10;Rpl1	biosynthetic process;cellular biosynthetic proces	structural constituent of ribosome;structural m	cell part;cytoplasmic part;cytosolic large riboso	Ribosome
Annexin A3	Anxa3	biological regulation;cell activation;cell activatio	binding;calcium ion binding;calcium-dependent	cell part;cytoplasmic membrane-bounded vesic	
RAC-alpha serine/threonin	Akt1	activation-induced cell death of T cells;aging;alc	adenyl nucleotide binding;adenyl ribonucleotide	cell part;cytoplasm;cytoplasmic part;cytoskeleta	Acute myeloid leukemia;Adipocytokine signaling
60S ribosomal protein L38	Rpl38	90S preribosome assembly;anatomical structure	structural constituent of ribosome;structural m	cell part;cytoplasmic part;cytosolic large riboso	Ribosome
Trifunctional enzyme subu	Hadhb	carboxylic acid catabolic process;carboxylic acid	3-hydroxyacyl-CoA dehydrogenase activity;acety	cell part;cytoplasmic part;endoplasmic reticulu	Benzoate degradation;Fatty acid elongation in m
60S ribosomal protein L23	Rpl23	biosynthetic process;cellular biosynthetic proces	structural constituent of ribosome;structural m	cell part;cytoplasmic part;cytosolic large riboso	Ribosome
60S ribosomal protein L27	Rpl27a	biosynthetic process;cellular biosynthetic proces	structural constituent of ribosome;structural m	cell part;cytoplasmic part;intracellular non-mem	Ribosome
Hydroxymethylglutaryl-Co	Hmgcs1	alcohol metabolic process;anatomical structure	binding;catalytic activity;drug binding;hydroxym	cell part;cytoplasmic part;cytosol;intracellular m	Butanoate metabolism;Synthesis and degradatio
ATPase inhibitor, mitocho	Atpif1	biological regulation;biosynthetic process;cell di	angiostatin binding;ATPase binding;ATPase inhib	cell part;cell surface;cytoplasmic part;intracellu	
Phosphate carrier protein	Slc25a3	establishment of localization;transport	active transmembrane transporter activity;seco	cell part;cytoplasmic part;integral to membrane	
Lon protease homolog, m	Lonp1	aging;biological regulation;catabolic process;cel	adenyl nucleotide binding;adenyl ribonucleotide	cell part;cytoplasmic part;intracellular non-mem	
40S ribosomal protein S23	Rps23	biosynthetic process;cellular biosynthetic proces	structural constituent of ribosome;structural m	cell part;cytosolic small ribosomal subunit;intra	Ribosome
Calcium-binding mitochon	Slc25a12	establishment of localization;establishment of lo	acidic amino acid transmembrane transporter a	cell part;cytoplasmic part;integral to membrane	
Transporter;Sodium- and	Slc6a8;Slc6a		active transmembrane transporter activity;amin	cell part;integral to membrane;integral to plasm	
60S ribosomal protein L24	Rpl24			cell part;cytoplasmic part;cytosolic large riboso	Ribosome
Stress-70 protein, mitocho	Hspa9	cellular macromolecule metabolic process;cellul	adenyl nucleotide binding;adenyl ribonucleotide	cell part;cell surface;cytoplasmic part;intracellul	ko05152;RNA degradation
Angiopoietin-related prot	Angptl2	anatomical structure formation involved in mor		cell part;cytoplasmic membrane-bounded vesic	
V-type proton ATPase sub	Atp6v1h	ATP hydrolysis coupled proton transport;cation	active transmembrane transporter activity;ATPa	cell part;cytoplasmic part;intracellular organelle	Epithelial cell signaling in Helicobacter pylori inf
Ferritin;Ferritin light chain	Ftl1	biological regulation;cation homeostasis;cation	binding;catalytic activity;cation binding;ferric ir	cell part;ferritin complex;intracellular ferritin co	
Delta-1-pyrroline-5-carbo	Aldh18a1	amine biosynthetic process;amine metabolic pr	adenyl nucleotide binding;adenyl ribonucleotide	cell part;cytoplasmic part;intracellular membra	Arginine and proline metabolism
COP9 signalosome comple	Cops7a	cellular macromolecule metabolic process;cellul		cell part;cytoplasm;intracellular organelle part;i	
Signal sequence receptor,	Ssr4			cell part;cytoplasmic part;endoplasmic reticulu	Protein processing in endoplasmic reticulum
Tricarboxylate transport p	Slc25a1	cellular process;establishment of localization;tra		cell part;cytoplasmic part;integral to membrane	
Rho GTPase-activating pro	Arhgap8	biological regulation;cellular process;cellular res	enzyme activator activity;enzyme regulator acti	cell part;intracellular	

Calreticulin	Calr	actin cytoskeleton organization;actin filament-b	binding;calcium ion binding;carbohydrate bindir	acrosomal vesicle;cell part;cytoplasmic membra	Antigen processing and presentation;Chagas dis
V-type proton ATPase sub	Atp6v0a1	ATP hydrolysis coupled proton transport;cation	cation transmembrane transporter activity;hydr	cell part;cytoplasm;cytoplasmic membrane-bou	Collecting duct acid secretion;Epithelial cell sign
Tryptophan--tRNA ligase,	Wars	amine metabolic process;amino acid activation;	adenyl nucleotide binding;adenyl ribonucleotide	cell part;cytoplasm;intracellular part	Aminoacyl-tRNA biosynthesis;Tryptophan metal
60S ribosomal protein L36	Rpl36a;Gm652	biosynthetic process;cellular biosynthetic proces	structural constituent of ribosome;structural mo	cell part;cytoplasmic part;intracellular non-mem	Ribosome
7-dehydrocholesterol redu	Dhcr7	alcohol metabolic process;anatomical structure	7-dehydrocholesterol reductase activity;catalyti	cell part;cytoplasmic part;endoplasmic reticulum	Steroid biosynthesis
Acyl-coenzyme A thioeste	Acot9;Acot10	acyl-CoA metabolic process;cellular metabolic p	acetyl-CoA hydrolase activity;acyl-CoA hydrolase	cell part;cytoplasmic part;intracellular membrar	
3-keto-steroid reductase	Hsd17b7	alcohol metabolic process;biological regulation;	3-keto sterol reductase activity;catalytic activity	cell part;cytoplasmic part;endoplasmic reticulum	Steroid biosynthesis;Steroid hormone biosynthe
Calcium-binding mitochon	Slc25a24	cellular process;establishment of localization;tra	binding;calcium ion binding;cation binding;ion b	cell part;cytoplasmic part;integral to membrane	
V-type proton ATPase sub	Atp6v1e1	ATP hydrolysis coupled proton transport;cation	active transmembrane transporter activity;ATPa	acrosomal vesicle;apical plasma membrane;cell	Collecting duct acid secretion;Epithelial cell sign
V-type proton ATPase sub	Atp6v1b2	ATP hydrolysis coupled proton transport;ATP me	adenyl nucleotide binding;adenyl ribonucleotide	apical plasma membrane;basolateral plasma me	Collecting duct acid secretion;Epithelial cell sign
ATP synthase subunit epsi	Atp5e	ATP biosynthetic process;ATP metabolic process	active transmembrane transporter activity;ATPa	cell part;cytoplasmic part;intracellular organelle	Alzheimer's disease;Huntington's disease;Oxidat
60S ribosomal protein L31	Rpl31	biosynthetic process;cellular biosynthetic proces	structural constituent of ribosome;structural mo	cell part;cytosolic large ribosomal subunit;intrac	Ribosome
40S ribosomal protein S26	Rps26	biological regulation;biosynthetic process;cellula	binding;mRNA binding;nucleic acid binding;RNA	cell part;cytosolic small ribosomal subunit;intrac	Ribosome
Tumor necrosis factor rec	Tradd	biological regulation;induction of apoptosis;indu	binding;binding, bridging;molecular transducer a	cell part;cytoplasm;cytoskeleton;intracellular m	Adipocytokine signaling pathway;Apoptosis;Hep
Long-chain-specific acyl-C	Acadl	amine catabolic process;amine metabolic proces	acyl-CoA dehydrogenase activity;acyl-CoA oxida	cell part;cytoplasmic part;intracellular membrar	Fatty acid metabolism;PPAR signaling pathway
Argininosuccinate synthas	Ass1	amide biosynthetic process;amine biosynthetic	adenyl nucleotide binding;adenyl ribonucleotide	cell part;cytoplasmic part;intracellular membrar	Alanine, aspartate and glutamate metabolism;A
V-type proton ATPase sub	Atp6v0d1	anatomical structure development;anatomical s	active transmembrane transporter activity;ATPa	apical plasma membrane;axon part;axon termin	Collecting duct acid secretion;Epithelial cell sign
Neutral cholesterol ester	Nceh1	catabolic process;cellular macromolecule metab	anion binding;binding;carboxylesterase activity;	cell part;cytoplasmic part;endoplasmic reticulum	Bile secretion
Tubulin beta-5 chain	Tubb5	cellular component assembly;cellular componer	binding;catalytic activity;GTP binding;GTPase ac	cell body;cell part;cytoplasm;cytoplasmic part;c	Gap junction;Pathogenic Escherichia coli infectio
Sphingolipid delta(4)-desa	Degs1	biosynthetic process;carboxylic acid biosyntheti	catalytic activity;oxidoreductase activity;oxidore	cell part;cytoplasmic part;endoplasmic reticulum	Sphingolipid metabolism
rRNA 2-O-methyltransfera	Fbl	cellular macromolecule metabolic process;cellul	binding;catalytic activity;methyltransferase activ	cell part;cytoplasmic part;intracellular membrar	Ribosome biogenesis in eukaryotes
Actin-related protein 2/3	Arpc5	biological regulation;cellular component organiz		Arp2/3 protein complex;cell part;cell projection	Bacterial invasion of epithelial cells;Fc gamma R
Fibronectin	Fn1	biological adhesion;calcium-independent cell-m	enzyme activator activity;enzyme regulator activ	apical plasma membrane;basement membrane;	Amoebiasis;Bacterial invasion of epithelial cells;
Aldehyde dehydrogenase,	Aldh2	9-cis-retinoic acid biosynthetic process;9-cis-reti	3-chloroallyl aldehyde dehydrogenase activity;a	cell part;cytoplasm;cytoplasmic part;cytosol;intr	Arginine and proline metabolism;Ascorbate and
ADP-ribosylation factor-lik	Arl1	activation of phospholipase D activity;biological	binding;catalytic activity;cation binding;enzyme	cell part;cytoplasmic part;Golgi apparatus;Golgi	
Electron transfer flavopro	Etfa	cellular metabolic process;cellular process;elect	binding;catalytic activity;coenzyme binding;cofa	cell part;cytoplasmic part;electron transfer flavo	
60S ribosomal protein L32	Rpl32	biosynthetic process;cellular biosynthetic proces	structural constituent of ribosome;structural mo	cell part;cytoplasmic part;cytosolic large ribosor	Ribosome
Heat shock protein 75 kDa	Trap1	cellular macromolecule metabolic process;cellul	adenyl nucleotide binding;adenyl ribonucleotide	cell part;cytoplasmic part;intracellular membrar	
V-type proton ATPase sub	Atp6v1d	anatomical structure formation involved in mor	active transmembrane transporter activity;ATPa	cell part;cytoplasmic part;intracellular organelle	Collecting duct acid secretion;Epithelial cell sign
V-type proton ATPase cata	Atp6v1a	ATP hydrolysis coupled proton transport;ATP me	active transmembrane transporter activity;aden	apical plasma membrane;cell part;cell projectio	Collecting duct acid secretion;Epithelial cell sign
Transmembrane 9 superfa	Tm9sf2			cell part;cytoplasmic part;endosomal part;endos	
Oligosaccharyltransferase	Ostc			cell part;integral to membrane;intrinsic to mem	
ATP synthase F(0) comple	Atp5f1	ATP biosynthetic process;ATP metabolic process	cation transmembrane transporter activity;hydr	cell part;cytoplasmic part;intracellular organelle	Alzheimer's disease;Huntington's disease;Oxidat
Nodal modulator 1	Nomo1		binding;carbohydrate binding	cell part;integral to membrane;intrinsic to mem	
Serine hydroxymethyltran	Shmt2	amine biosynthetic process;amine metabolic pro	aldehyde-lyase activity;amine binding;amino aci	cell part;cytoplasmic part;cytoskeleton;intracell	Cyanoamino acid metabolism;Glycine, serine an
Monofunctional C1-tetrah	Mthfd1l	10-formyltetrahydrofolate biosynthetic process;	adenyl nucleotide binding;adenyl ribonucleotide	cell part;cytoplasmic part;intracellular membrar	One carbon pool by folate
ATP synthase subunit f, m	Atp5j2	ATP biosynthetic process;ATP metabolic process	ATPase activity;catalytic activity;hydrolase activ	cell part;cytoplasmic part;integral to membrane	Oxidative phosphorylation
Complement component	C1qbp	activation of immune response;apoptosis;biolog	adrenergic receptor binding;binding;carbohydra	cell part;cell surface;cytoplasmic part;cytosol;ex	
ATP synthase subunit gam	Atp5c1	ATP biosynthetic process;ATP metabolic process	active transmembrane transporter activity;ATPa	cell part;cytoplasmic part;intracellular organelle	Alzheimer's disease;Huntington's disease;Oxidat
Dihydrolipoyllysine-residu	Dlst	acetyl-CoA catabolic process;acetyl-CoA metabo	catalytic activity;dihydrolipoyllysine-residue suc	cell part;cytoplasmic part;intracellular membrar	Citrate cycle (TCA cycle);Lysine degradation
17beta-hydroxysteroid de	Hsd17b10	cellular macromolecule metabolic process;cellul	3-hydroxy-2-methylbutyryl-CoA dehydrogenase	cell part;cytoplasmic part;endoplasmic reticulum	Alzheimer's disease;Valine, leucine and isoleucir
Dihydrolipoyllysine-residu	Dlat	acetyl-CoA biosynthetic process;acetyl-CoA bios	acetyltransferase activity;catalytic activity;dihyd	cell part;cytoplasmic part;intracellular organelle	Citrate cycle (TCA cycle);Glycolysis / Gluconeoge
Mitochondrial import rece	Tomm70			cell part;cytoplasmic part;integral to membrane	
ATPase Asna1	Asna1	ATP catabolic process;ATP metabolic process;ca	adenyl nucleotide binding;adenyl ribonucleotide	cell part;cytoplasmic part;endoplasmic reticulum	
Isocitrate dehydrogenase	Idh3a	acetyl-CoA catabolic process;acetyl-CoA metabo	binding;catalytic activity;cation binding;coenzy	cell part;cytoplasmic part;intracellular membrar	Citrate cycle (TCA cycle)
SPI6	Serpinb9	acute inflammatory response;acute inflammato	endopeptidase inhibitor activity;endopeptidase	cell part;cytoplasm;intracellular part	Amoebiasis
D-glutamate cyclase, mito	Dglucy			cell part;cytoplasmic part;intracellular membrar	
60S ribosomal protein L17	Rpl17	biosynthetic process;cellular biosynthetic proces	structural constituent of ribosome;structural mo	cell part;intracellular organelle part;intracellular	Ribosome
ATP synthase subunit alph	Atp5a1	ATP biosynthetic process;ATP hydrolysis couple	active transmembrane transporter activity;aden	cell part;cytoplasmic part;intracellular organelle	Alzheimer's disease;Huntington's disease;Oxidat
Aspartate aminotransfera	Got2	2-oxoglutarate metabolic process;amine biosyn	binding;catalytic activity;cofactor binding;kynur	cell part;cytoplasmic part;intracellular organelle	Alanine, aspartate and glutamate metabolism;A
Cytochrome c oxidase sub	Cox7a2		catalytic activity;cation transmembrane transpo	cell part;cytoplasmic part;intracellular organelle	Alzheimer's disease;Cardiac muscle contraction;
Bifunctional coenzyme A	Coasy	biosynthetic process;cellular biosynthetic proces	adenyl nucleotide binding;adenyl ribonucleotide	cell part;cytoplasm;intracellular membrane-bou	Pantothenate and CoA biosynthesis
ATP synthase subunit beta	Atp5b	anatomical structure formation involved in mor	active transmembrane transporter activity;aden	cell part;cell surface;cytoplasmic part;intracell	Alzheimer's disease;Huntington's disease;Oxidat
60 kDa heat shock protein	Hspd1	activation of caspase activity;activation of immu	adenyl nucleotide binding;adenyl ribonucleotide	cell part;cell surface;coated pit;coated vesicle;c	ko05152;RNA degradation;Type I diabetes mellit
ATP synthase-coupling fac	Atp5j	ATP biosynthetic process;ATP metabolic process	ATPase activity;catalytic activity;cation transme	cell part;cytoplasmic part;intracellular organelle	Alzheimer's disease;Huntington's disease;Oxidat
ADP/ATP translocase 1	Slc25a4	apoptotic mitochondrial changes;biological regu	transporter activity	cell part;cell projection;cilium;cytoplasmic part;	Calcium signaling pathway;Huntington's disease
GPI transamidase compor	Pigs	attachment of GPI anchor to protein;cellular ma		cell part;cytoplasmic part;endoplasmic reticulum	Glycosylphosphatidylinositol(GPI)-anchor biosyr
Translocator protein	Tspo	adrenal gland development;aging;anatomical st	androgen binding;benzodiazepine receptor activ	cell part;cytoplasmic part;integral to membrane	Neuroactive ligand-receptor interaction
Lipase maturation factor 2	Lmf2			cell part;cytoplasmic part;endoplasmic reticulum	
Citrate synthase, mitocho	Cs	acetyl-CoA catabolic process;acetyl-CoA metabo	catalytic activity;citrate (Si)-synthase activity;tra	cell part;cytoplasmic part;intracellular membrar	Citrate cycle (TCA cycle);Glyoxylate and dicarbox
Glutamate dehydrogenase	Glud1	amine metabolic process;behavior;biological reg	adenyl nucleotide binding;adenyl ribonucleotide	cell part;cytoplasmic part;intracellular organelle	Alanine, aspartate and glutamate metabolism;A
ADP/ATP translocase 2	Slc25a5	biological regulation;cellular process;chromosor	transporter activity	cell part;cytoplasmic part;cytoskeletal part;integ	Calcium signaling pathway;Huntington's disease
Pyruvate dehydrogenase	Pdha1	acetyl-CoA biosynthetic process;acetyl-CoA bios	catalytic activity;oxidoreductase activity;oxidore	cell part;cytoplasmic part;intracellular membrar	Butanoate metabolism;Citrate cycle (TCA cycle);
NAD(P) transhydrogenase	Nnt	cation transport;cellular metabolic process;cellu	binding;catalytic activity;coenzyme binding;cofa	cell part;cytoplasmic part;integral to membrane	
ATP synthase protein 8	Mtatp8	ATP biosynthetic process;ATP metabolic process	ATPase activity;catalytic activity;cation transme	cell part;cytoplasmic part;integral to membrane	Alzheimer's disease;Huntington's disease;Oxidat
Dihydrolipoyl dehydrogen	Dld	anatomical structure morphogenesis;biological	binding;catalytic activity;coenzyme binding;cofa	acrosomal matrix;cell part;cell projection;cilium	Citrate cycle (TCA cycle);Glycine, serine and thre
Tubulin alpha chain (Frag	Tuba4a	cellular component assembly;cellular componer	binding;catalytic activity;GTP binding;GTPase ac	cell part;cytoplasm;cytoskeletal part;intracellula	Gap junction;Pathogenic Escherichia coli infectic
Cytochrome c oxidase sub	Cox7c		catalytic activity;cation transmembrane transpo	cell part;cytoplasmic part;integral to membrane	Alzheimer's disease;Cardiac muscle contraction;
Ornithine aminotransfera	Oat	amine biosynthetic process;amine metabolic pro	binding;catalytic activity;cofactor binding;ornith	cell part;cytoplasmic part;intracellular membrar	Arginine and proline metabolism
Prohibitin-2;Prohibitin-2 (	Phb2	anatomical structure development;anatomical s		cell part;cytoplasmic part;intracellular membrar	
Cytochrome c oxidase sub	Ndufa4	cellular metabolic process;cellular process;elect	catalytic activity;cation transmembrane transpo	cell part;cytoplasmic part;intracellular organelle	Alzheimer's disease;Huntington's disease;Oxidat
WASH complex subunit 2;	Washc2	cellular process;endosome transport;establishm		cell part;cytoplasmic part;early endosome;early	
Dolichyl-diphosphooligosa	Dad1	anatomical structure development;apoptosis;bi	catalytic activity;dolichyl-diphosphooligosacchar	cell part;cytoplasmic part;endoplasmic reticulum	N-Glycan biosynthesis;Protein processing in end
Cytochrome b-c1 complex	Uqcr10	cellular metabolic process;cellular process;elect		cell part;cytoplasmic part;intracellular organelle	Alzheimer's disease;Cardiac muscle contraction;
ATP synthase subunit O, m	Atp5o	ATP biosynthetic process;ATP metabolic process	binding;cation transmembrane transporter activ	cell part;cytoplasmic part;intracellular organelle	Alzheimer's disease;Huntington's disease;Oxidat
10 kDa heat shock protein	Hspe1	cellular macromolecule metabolic process;cellul	adenyl nucleotide binding;adenyl ribonucleotide	cell part;cytoplasmic part;intracellular membrar	
ATP synthase subunit d, m	Gm10250;Atp	ATP biosynthetic process;ATP metabolic process	cation transmembrane transporter activity;hydr	cell part;cytoplasmic part;intracellular membrar	Alzheimer's disease;Huntington's disease;Oxidat
GrpE protein homolog 1, r	Grpel1	cellular macromolecule metabolic process;cellul	adenyl nucleotide binding;adenyl-nucleotide exc	cell part;cytoplasmic part;intracellular membrar	
V-type proton ATPase sub	Atp6v1c1	ATP hydrolysis coupled proton transport;cation	active transmembrane transporter activity;ATPa	apical part of cell;cell part;cytoplasmic part;cyto	Collecting duct acid secretion;Epithelial cell sign
Glypican-1	Gpc1	anatomical structure formation involved in mor	binding;cation binding;copper ion binding;extrac	anchored to membrane;cell part;cytoplasmic pa	
Nuclear factor NF-kappa-E	Nfkb2	adaptive immune response;adaptive immune re	binding;DNA binding;nucleic acid binding;nuclei	Bcl3/NF-kappaB2 complex;cell part;cytoplasm;I-	MAPK signaling pathway;Osteoclast differentiati
Cell division cycle protein	Cdc123	cell cycle;cell division;cellular process		cell part;cytoplasm;intracellular part	
Malate dehydrogenase, m	Mdh2	acetyl-CoA catabolic process;acetyl-CoA metabo	catalytic activity;L-malate dehydrogenase activit	cell part;cytoplasmic part;intracellular membrar	Carbon fixation in photosynthetic organisms;Citr
Cathepsin D;Uncharacteri	Ctsd	autophagic vacuole assembly;cellular componer	aspartic-type endopeptidase activity;aspartic-ty	cell part;cytoplasmic membrane-bounded vesicl	ko05152;Lysosome
Solute carrier family 39 (Z	Slc39a14	biological regulation;cation homeostasis;cation	cation transmembrane transporter activity;dival	cell part;cytoplasmic part;endoplasmic reticulum	
ATPase family AAA domai	Atad3		adenyl nucleotide binding;adenyl ribonucleotide	cell part;cytoplasmic part;integral to membrane	
Prohibitin;Prohibitin (Frag	Phb	biological regulation;biosynthetic process;cellula	binding;DNA binding;nucleic acid binding;nuclei	cell part;cytoplasmic part;integral to membrane	
ATP-dependent RNA helic	Ddx19a;Ddx19	anatomical structure development;biological reg	adenyl nucleotide binding;adenyl ribonucleotide	cell part;chromatoid body;cytoplasm;cytoplasm	
Tumor necrosis factor rec	Tnfrsf10b	apoptosis;apoptosis in response to endoplasmic		cell part;integral to membrane;intrinsic to mem	Apoptosis;Cytokine-cytokine receptor interactio
V-type proton ATPase pro	Atp6v0c	ATP hydrolysis coupled proton transport;biologi	active transmembrane transporter activity;ATPa	cell part;cytoplasmic part;integral to membrane	Collecting duct acid secretion;Epithelial cell sign
MCG8382, isoform CRA_d	Gm21992;Rbm	biological regulation;biosynthetic process;cellula	binding;cation binding;ion binding;ligand-depen	cell part;intracellular non-membrane-bounded o	
Collagen alpha-1(II) chain	Col2a1	anatomical structure development;anatomical s	binding;cation binding;extracellular matrix struc	basement membrane;cell part;collagen;collager	Amoebiasis;ECM-receptor interaction;Focal adh
Pyrroline-5-carboxylate re	Pycr2	amine biosynthetic process;amine metabolic pro	catalytic activity;oxidoreductase activity;oxidore	cell part;cytoplasm;cytoplasmic part;intracellula	Arginine and proline metabolism
Cytochrome c oxidase sub	Cox6a1		catalytic activity;cation transmembrane transpo	cell part;cytoplasmic part;intracellular membrar	Alzheimer's disease;Cardiac muscle contraction;
ER membrane protein con	Emc1			ER membrane protein complex;macromolecular	
Thioredoxin, mitochondria	Txn2	biological regulation;cell communication;cell rec	catalytic activity;disulfide oxidoreductase activit	cell body;cell part;cell projection;cytoplasmic pa	
Protein transport protein	Sec61a1	establishment of localization;establishment of p	binding;ribonucleoprotein binding;ribosome bin	cell part;cytoplasmic part;cytoplasmic vesicle m	Phagosome;Protein export;Protein processing ir
Succinate--CoA ligase [AD	Sucla2	acetyl-CoA catabolic process;acetyl-CoA metabo	acid-thiol ligase activity;adenyl nucleotide bindi	cell part;cytoplasmic part;intracellular membrar	Citrate cycle (TCA cycle);Propanoate metabolism
Glycosylated lysosomal m	Glmp	biological regulation;positive regulation of biolo	binding;DNA binding;ligand-dependent nuclear	cell part;cytoplasmic part;cytosol;integral to me	
Cytochrome c oxidase sub	Cox6c		catalytic activity;cation transmembrane transpo	cell part;cytoplasmic part;integral to membrane	Alzheimer's disease;Cardiac muscle contraction;
Succinyl-CoA:3-ketoacid c	Oxct1	adipose tissue development;anatomical structur	3-oxoacid CoA-transferase activity;catalytic activ	cell part;cytoplasmic part;intracellular membrar	Butanoate metabolism;Synthesis and degradatic
Polyadenylate-binding pro	Pabpc4;Pab		binding;nucleic acid binding;nucleotide binding;		mRNA surveillance pathway;RNA degradation;R
Acyl-coenzyme A thioeste	Acot13	cellular component assembly;cellular componer	acyl-CoA hydrolase activity;catalytic activity;CoA	cell part;cytoplasmic part;intracellular membrar	
Solute carrier family 12 m	Slc12a9	cellular process;establishment of localization;tra		cell part;integral to membrane;intrinsic to mem	
Isocitrate dehydrogenase	Idh3g	acetyl-CoA catabolic process;acetyl-CoA metabo	adenyl nucleotide binding;adenyl ribonucleotide	cell part;cytoplasmic part;intracellular membrar	Citrate cycle (TCA cycle)
ER lumen protein-retainin	Kdelr3	biological regulation;cellular process;establishm	binding;ER retention sequence binding;peptide	cell part;cytoplasmic part;endoplasmic reticulum	Vibrio cholerae infection
Elongation factor Tu, mito	Tufm	catabolic process;cellular catabolic process;cellu	binding;catalytic activity;GTP binding;GTPase ac	cell part;cytoplasmic part;intracellular organelle	Plant-pathogen interaction
Ferritin heavy chain	Fth1	biological regulation;cation homeostasis;cation	binding;catalytic activity;cation binding;ferric irc	cell part;cytoplasmic part;intracellular membrar	Mineral absorption;Porphyrin and chlorophyll m
Cytochrome b-c1 complex	Uqcrc2	cellular metabolic process;cellular process;elect	binding;catalytic activity;cation binding;endopep	cell part;cytoplasmic part;intracellular organelle	Alzheimer's disease;Cardiac muscle contraction;
Pyruvate carboxylase;Pyru	Pcx;Pc	alcohol biosynthetic process;alcohol metabolic p	adenyl nucleotide binding;adenyl ribonucleotide	cell part;cytoplasmic part;intracellular membrar	Citrate cycle (TCA cycle);Pyruvate metabolism
Mitochondrial carrier hom	Mtch2;	establishment of localization;transport		cell part;cytoplasmic part;integral to membrane	
Lysophospholipid acyltran	Lpcat3	biological regulation;biosynthetic process;cellula	1-acylglycerophosphocholine O-acyltransferase	cell part;cytoplasmic part;endoplasmic reticulum	Glycerophospholipid metabolism
Proline-, glutamic acid- an	Pelp1	biosynthetic process;cellular biosynthetic proces		cell part;cytoplasm;histone methyltransferase c	

ATP-dependent Clp protea	Clpp	macromolecule metabolic process;metabolic pr	adenyl nucleotide binding;adenyl ribonucleotide	cell part;cytoplasmic part;intracellular membran	Cell cycle - Caulobacter
Calcium-binding mitochon	Slc25a13	ATP biosynthetic process;ATP metabolic process	acidic amino acid transmembrane transporter a	cell part;cytoplasmic part;integral to membrane	
Tubulointerstitial nephriti	Tinagl1	immune response;immune system process;mac	binding;carbohydrate binding;cargo receptor ac	cell part;cytoplasm;extracellular matrix;extracel	
Peptidyl-tRNA hydrolase 2	Ptrh2	biological regulation;negative regulation of anoi	aminoacyl-tRNA hydrolase activity;carboxylic es	cell part;cytoplasmic part;cytosol;intracellular m	
Ribosome biogenesis prot	Brix1	cellular component biogenesis;cellular compone		cell part;intracellular non-membrane-bounded	
Mitochondrial-processing	Pmpca	macromolecule metabolic process;metabolic pr	binding;catalytic activity;cation binding;endope	cell part;cytoplasmic part;intracellular membran	
Cytochrome b-c1 complex	Uqcrc1	cellular metabolic process;cellular process;elect	binding;catalytic activity;cation binding;ion bind	cell part;cytoplasmic part;intracellular organelle	Alzheimer's disease;Cardiac muscle contraction;
tRNA (cytosine(34)-C(5))-r	Nsun2	cell cycle phase;cell cycle process;cell division;ce	binding;catalytic activity;methyltransferase activ	cell part;cytoplasm;cytoskeletal part;intracellula	
NADH dehydrogenase [ub	Ndufa9	cellular metabolic process;cellular process;elect		cell part;cytoplasmic part;intracellular organelle	Alzheimer's disease;Huntington's disease;Oxidat
Sodium-coupled neutral a	Slc38a2	cation transport;establishment of localization;io	active transmembrane transporter activity;amin	cell part;integral to membrane;intrinsic to mem	Protein digestion and absorption
Fibronectin	Fn1	biological adhesion;calcium-independent cell-m	enzyme activator activity;enzyme regulator activ	apical plasma membrane;basement membrane;	Amoebiasis;Bacterial invasion of epithelial cells;
ATP synthase subunit g, m	Atp5l	ATP biosynthetic process;ATP metabolic process	cation transmembrane transporter activity;hydr	cell part;cytoplasmic part;intracellular organelle	Oxidative phosphorylation
Vesicle transport protein (	Golt1b	biological regulation;cellular process;establishm	molecular transducer activity;signal transducer a	cell part;cytoplasmic part;endoplasmic reticulun	
Serum paraoxonase/aryle	Pon2	aromatic compound catabolic process;catabolic	arylesterase activity;binding;carboxylic ester hyd	cell part;cytoplasmic part;extracellular region;in	Aminobenzoate degradation;Bisphenol degrada
Protein RRP5 homolog	Pdcd11	apoptosis;biological regulation;cell death;cellula	binding;nucleic acid binding;RNA binding	cell part;cytoplasmic part;cytosol;intracellular n	
Sorting and assembly mac	Samm50	cellular component organization;cellular compo		cell part;cytoplasmic part;integral to membrane	
Leucine-rich PPR motif-co	Lrpprc	biological regulation;biosynthetic process;cellula	beta-tubulin binding;binding;cytoskeletal protei	cell part;chromosome;condensed chromosome;	
Cytochrome c oxidase sub	Cox4i1		catalytic activity;cation transmembrane transpo	cell part;cytoplasmic part;intracellular organelle	Alzheimer's disease;Cardiac muscle contraction;
Cytochrome c oxidase sub	Cox5a		binding;catalytic activity;cation binding;cation tr	cell part;cytoplasmic part;intracellular organelle	Alzheimer's disease;Cardiac muscle contraction;
Cortactin, isoform CRA_a;	Cttn			cell cortex;cell part;cell projection;cell projectio	Bacterial invasion of epithelial cells;Pathogenic E
Kinesin-like protein KIF23;	Kif23	cell cycle process;cellular component assembly;	adenyl nucleotide binding;adenyl ribonucleotide	cell part;centralspindlin complex;centrosome;cy	
Copine-8	Cpne8				
Syndecan-4	Sdc4	anatomical structure development;biological re	molecular transducer activity;receptor activity;s	adherens junction;anchoring junction;cell juncti	Cell adhesion molecules (CAMs);ECM-receptor i
Rac GTPase-activating pro	Racgap1	actin cytoskeleton organization;actin filament-b	alpha-tubulin binding;beta-tubulin binding;bindi	acrosomal vesicle;cell division site part;cell part;	
MLV-related proviral Env polyprotein		reproductive process;viral infectious cycle;viral r	binding;cation binding;ion binding;metal ion bin	cell part;integral to membrane;intrinsic to mem	
Glucosamine-6-phosphate	Gnpda1	acrosome reaction;alcohol biosynthetic process;	catalytic activity;deaminase activity;glucosamine	cell part;cytoplasm;intracellular part	Amino sugar and nucleotide sugar metabolism
Amine oxidase [flavin-con	Maoa	alcohol catabolic process;alcohol metabolic proc	catalytic activity;oxidoreductase activity	cell part;cytoplasmic part;integral to membrane	Arginine and proline metabolism;Drug metaboli
Cytochrome c1, heme pro	Cyc1	cellular metabolic process;cellular process;elect	binding;cation binding;electron carrier activity;h	cell junction;cell part;cytoplasmic part;integral t	Alzheimer's disease;Cardiac muscle contraction;
Cytochrome c oxidase sub	Gm11273;Cox		binding;catalytic activity;cation binding;cation tr	cell part;cytoplasmic part;envelope;intracellular	Alzheimer's disease;Cardiac muscle contraction;
Neuroblastoma-amplified	Nbas				
Kinesin-like protein KIF14	Kif14	cellular component movement;cellular process;	adenyl nucleotide binding;adenyl ribonucleotide	cell part;cytoskeletal part;intracellular organelle	
Lysyl oxidase homolog 4	Loxl4		binding;cargo receptor activity;catalytic activity;	cell part;extracellular region;extracellular region	
Glutaminase kidney isofor	Gls	amine biosynthetic process;amine catabolic proc	catalytic activity;glutaminase activity;hydrolase	cell part;cytoplasmic part;cytosol;intracellular m	Alanine, aspartate and glutamate metabolism;A
Microtubule-associated p	Map1lc3b	autophagy;catabolic process;cellular catabolic p		autophagic vacuole;autophagic vacuole membra	
Cytochrome b-c1 complex	Uqcrh	cellular component assembly;cellular componen	catalytic activity;cation transmembrane transpo	cell part;cytoplasmic part;intracellular organelle	Alzheimer's disease;Cardiac muscle contraction;
Mitochondrial import rece	Tomm40	cellular process;establishment of localization;es	channel activity;passive transmembrane transpo	cell part;cytoplasmic part;integral to membrane	Amyotrophic lateral sclerosis (ALS)
Sequestosome-1	Sqstm1	apoptosis;autophagy;biological regulation;catab	binding;cation binding;enzyme binding;identical	aggresome;autophagic vacuole;cell part;cytopla	Osteoclast differentiation
Lipid droplet-associated h	Ldah				
TBC1 domain family mem	Tbc1d15		enzyme activator activity;enzyme regulator activ	cell part;cytoplasmic part;extracellular region;in	
Importin subunit alpha-1	Kpna2	establishment of localization;establishment of lo	protein transporter activity;substrate-specific tr	cell part;cytoplasm;intracellular membrane-bou	
Apol9a protein;Apolipopr	Apol9a;Apol9b	cellular macromolecule metabolic process;cellul	binding;lipid binding	extracellular region	

Supplemental table 3: Student's T-test between PyMT Control and PyMT RT

LQF Intensity PYMT Control1	LQF Intensity PYMT Control2	LQF Intensity PYMT Control3	LQF Intensity PYMT RT1	LQF Intensity PYMT RT2	LQF Intensity PYMT RT3	-Log t-test p value PyMT Control vs RT	t-test Difference PyMT Control vs RT	Cluster	Protein IDs	Majority protein IDs
28.12139	29.96774	29.104	24.70767	26.14686	26.30487	1.979008172	-3.344577789	Higher in PyMT Control	Q9CY64;A2ASB8;A2	Q9CY64
31.29789	31.22504	31.51089	27.60399	28.14102	28.27142	3.947404622	-3.339127223	Higher in PyMT Control	P97298;B7ZC25;F6S	P97298
26.38772	25.67303	27.07112	23.06579	23.51653	23.17798	2.735926848	-3.123858134	Higher in PyMT Control	E9Q8I0;P06909;D6F	E9Q8I0;P06909;D6
26.99766	25.98251	26.45356	23.94056	24.23169	23.71816	2.803659625	-2.514437358	Higher in PyMT Control	M0QWP1;Z4YK85;A	M0QWP1;Z4YK85;
24.08537	22.89874	21.83461	20.47754	20.68859	NaN	1.16688226	-2.356508573	Higher in PyMT Control	F8WH23;E9Q5D9;Q	F8WH23;E9Q5D9;
24.98033	24.14102	24.67573	NaN	22.58233	22.46762	2.12926211	-2.074049632	Higher in PyMT Control	P43275	P43275
25.73892	25.1332	24.84805	23.03758	22.94414	23.55215	2.507115294	-2.062098821	Higher in PyMT Control	O09159	O09159
25.19084	24.56964	25.391	23.09507	22.93157	NaN	2.086430946	-2.037177086	Higher in PyMT Control	Q921I1;D3YYR8	Q921I1
25.32732	NaN	26.37451	24.37119	23.86703	23.20508	1.397549321	-2.036479314	Higher in PyMT Control	Q02257	Q02257
25.57831	25.59833	25.83123	23.75939	23.85757	23.36453	3.520822866	-2.008792241	Higher in PyMT Control	Q922H4;D3Z3L0;D3	Q922H4
29.13369	28.74535	29.48999	27.39917	27.07112	27.38278	2.80641957	-1.838652929	Higher in PyMT Control	E9Q035;P47758;F7	E9Q035
29.23306	28.97596	29.13614	27.58978	27.07112	27.1795	3.331600956	-1.834923426	Higher in PyMT Control	Q9QUR8	Q9QUR8
26.73892	26.38607	26.71293	25.33074	NaN	24.33074	1.675314645	-1.781902313	Higher in PyMT Control	Q9WUU7	Q9WUU7
24.20955	24.42982	25.64581	22.75328	22.68859	23.50446	1.579193116	-1.779619853	Higher in PyMT Control	Q7TNC4;E9Q715;Q	Q7TNC4;E9Q715;
26.19272	26.86231	26.57542	24.7543	25.08942	24.84805	2.782136599	-1.646229426	Higher in PyMT Control	P12399;A0A286YDF	P12399;A0A286YD
31.80039	31.80809	31.80039	30.12386	30.21928	30.18423	6.280760193	-1.627163569	Higher in PyMT Control	P06797;A0A1Y7VN	P06797;A0A1Y7VN
24.66492	NaN	24.5285	NaN	23.32389	22.94949	1.741903791	-1.460024834	Higher in PyMT Control	P14094;A0A0A6YX0	P14094;A0A0A6YX
25.09748	25.27498	25.08132	23.83846	23.57542	NaN	2.840269096	-1.444316228	Higher in PyMT Control	Q9ESY9	Q9ESY9
26.85043	26.99766	26.78955	25.73635	25.32389	25.28912	3.099943172	-1.429428736	Higher in PyMT Control	P43277;I7HFT9;Q07	P43277
26.72598	27.1795	27.2535	26.0296	25.60116	25.24264	2.1513055	-1.42852656	Higher in PyMT Control	P17095	P17095
25.75175	25.11744	25.38113	24.2535	24.11744	23.60964	2.214301543	-1.423247019	Higher in PyMT Control	Q8BGZ6;P51569;A2	Q8BGZ6;P51569
24.78955	25.1946	25.00196	23.31008	NaN	23.84805	1.958029909	-1.416307767	Higher in PyMT Control	Q3TSA8;Q8K021;D3	Q3TSA8;Q8K021;D
28.97869	30.36202	30.40324	28.41135	28.61456	28.509	1.387320775	-1.403016408	Higher in PyMT Control	Q4LFA9	Q4LFA9
27.33245	27.3662	27.29789	26.40406	25.59263	25.84086	2.344985299	-1.386327744	Higher in PyMT Control	P07141;D3Z090;D3	P07141;D3Z090
25.73635	25.29614	25.44253	23.58692	24.86703	23.89504	1.556270295	-1.375341415	Higher in PyMT Control	Q3TBA3;P21958;F6	Q3TBA3;P21958
25.44884	25.01478	25.53742	24.08537	NaN	23.83846	2.03677549	-1.371764819	Higher in PyMT Control	Q9ESX5;B7ZCL7;F6Y	Q9ESX5;B7ZCL7
32.67766	32.75334	32.81	31.51559	31.36202	31.36202	4.505391367	-1.333791097	Higher in PyMT Control	P10923;F8WIP8;D3	P10923;F8WIP8
28.7162	28.78644	28.65949	27.3662	27.51653	27.2803	4.158469462	-1.333034515	Higher in PyMT Control	P43276	P43276
30.40324	30.38278	30.91171	29.25575	29.06856	29.39917	2.588120991	-1.324750264	Higher in PyMT Control	P01887	P01887
26.33245	26.27853	26.30835	25.29263	24.5285	25.20955	2.228442429	-1.296209335	Higher in PyMT Control	Q8BL97	Q8BL97
29.62506	29.52962	29.47846	28.76129	28.06085	28.04531	2.195038447	-1.255228043	Higher in PyMT Control	Q61391;A0A0A6YY	Q61391
24.10149	25.02749	24.21697	23.77957	22.9566	22.85567	1.411672976	-1.251374563	Higher in PyMT Control	H3BKN0;Q1HFZ0	H3BKN0;Q1HFZ0
29.05051	28.95394	29.12139	27.78332	27.80809	27.78332	4.845652845	-1.250372569	Higher in PyMT Control	P21460;A2APX3	P21460;A2APX3
24.23169	23.95837	24.49838	22.73892	23.02242	23.19009	2.438440466	-1.245669683	Higher in PyMT Control	F8WH10;E9Q4N8;F	F8WH10;E9Q4N8;
26.63201	26.24989	26.05258	25.23169	25.5285	24.44253	1.563760399	-1.243917465	Higher in PyMT Control	Q9CZT5	Q9CZT5
28.88867	29.13614	28.85638	27.79576	27.78955	27.5969	3.444998778	-1.232993444	Higher in PyMT Control	Q5EBP8;P49312	Q5EBP8;P49312
32.3802	32.51559	32.33565	31.33565	31.31419	30.96086	3.08733757	-1.206914902	Higher in PyMT Control	P68433	P68433
25.24989	25.71816	25.56384	24.1332	24.17188	24.61526	2.36047199	-1.203845978	Higher in PyMT Control	P50428;F6QGM0	P50428
25.27853	24.91342	25.2856	24.27498	NaN	23.65403	1.624270996	-1.194676399	Higher in PyMT Control	P97310	P97310
25.92025	25.79452	25.71555	24.92705	24.32389	24.63755	2.517527221	-1.180610657	Higher in PyMT Control	O88968	O88968
25.17188	25.45513	25.37119	24.49228	24.44253	23.55215	1.679269659	-1.170412064	Higher in PyMT Control	A0A0A0MQ76;Q6D	A0A0A0MQ76;Q6
25.02749	25.56674	24.78457	23.79947	23.82881	24.2535	1.883606421	-1.165676753	Higher in PyMT Control	Q00PI9	Q00PI9
31.17831	31.24218	31.22504	30.02168	30.07368	30.07368	5.834133316	-1.158833186	Higher in PyMT Control	P10605	P10605
24.43619	24.72858	24.24627	NaN	23.33756	23.31008	2.096683558	-1.146525701	Higher in PyMT Control	P84089;A0A1W2P7	P84089;A0A1W2P
29.21466	29.23763	29.14346	28.16039	27.99766	28.0296	4.418519532	-1.136033376	Higher in PyMT Control	P43274	P43274
33.21928	33.33031	33.13155	32.16039	32.11148	32.04466	4.13796141	-1.121537526	Higher in PyMT Control	Q6ZWY9;Q64525;Q	Q6ZWY9;Q64525;
33.21928	33.30334	33.24785	32.20186	32.0927	32.11148	4.941672453	-1.121477763	Higher in PyMT Control	A0A0N4SV66;Q8R1	A0A0N4SV66;Q8R
29.17712	29.13614	28.87115	27.72598	28.14589	27.95947	2.710071765	-1.117687225	Higher in PyMT Control	O88569;A0A0N4SU	O88569;A0A0N4SU
28.51277	28.5969	27.70636	27.15074	27.01903	27.34942	1.668692687	-1.098946889	Higher in PyMT Control	Q64449;A2AAA9	Q64449;A2AAA9
27.3662	27.2803	27.2803	26.40243	26.03171	26.20769	3.22998325	-1.094986598	Higher in PyMT Control	P19788	P19788
33.49429	33.51794	33.50616	32.44085	32.43589	32.35941	5.639639399	-1.094081879	Higher in PyMT Control	P62806	P62806
28.22159	28.28912	28.29351	27.43936	27.16039	26.93157	2.736256517	-1.090967178	Higher in PyMT Control	Q3THW5;P0C0S6;Q	Q3THW5;P0C0S6;
30.47267	30.52962	30.4919	29.3869	29.47267	29.36828	5.136210477	-1.088781993	Higher in PyMT Control	P15864	P15864
27.81421	27.71293	27.74535	26.61807	26.71293	26.69975	4.863946818	-1.080579758	Higher in PyMT Control	O35988	O35988
26.63201	26.48616	26.43141	25.69179	25.43301	25.21327	2.68314164	-1.070505142	Higher in PyMT Control	Q8VIJ6	Q8VIJ6
23.75939	23.74919	23.92252	22.63423	22.94771	22.64306	3.093322461	-1.068702062	Higher in PyMT Control	O35188;A0A1D5RM	O35188
27.50142	27.3662	27.61105	26.21512	26.56674	26.51202	2.909861976	-1.061597188	Higher in PyMT Control	A2BE93;Q9EQU5;A	A2BE93;Q9EQU5;
25.97815	26.30661	25.89504	25.04009	24.95837	25.01478	2.928942366	-1.055518468	Higher in PyMT Control	Q9Z0L8	Q9Z0L8
25.86467	25.71816	25.63755	24.417	24.55215	25.08942	2.0891465	-1.05393664	Higher in PyMT Control	Q9JLZ6	Q9JLZ6
24.37782	24.05258	24.1332	23.32389	NaN	22.95127	1.948053965	-1.050291061	Higher in PyMT Control	Q9Z1N5;G3UXI6	Q9Z1N5
26.55362	26.44411	26.76446	25.41377	25.46451	25.73635	2.801957276	-1.049188614	Higher in PyMT Control	P52927;Q6NSP9	P52927;Q6NSP9
26.75175	26.69975	26.86231	25.91798	25.64031	25.66492	3.285971292	-1.030198415	Higher in PyMT Control	P84104;A2A4X6	P84104
26.48769	26.54185	26.42822	25.02327	25.59833	25.77455	1.949885526	-1.020542781	Higher in PyMT Control	Q8BK67;A2AWQ2	Q8BK67
25.76951	25.83123	25.27498	NaN	24.59833	24.61526	1.683166267	-1.018443743	Higher in PyMT Control	S4R1E5;F7A8H6;O7	S4R1E5;F7A8H6;O
26.27853	26.32732	25.85519	25.37119	24.74407	25.30313	1.819320811	-1.014217377	Higher in PyMT Control	P48759	P48759
26.86231	27.13124	27.01903	25.52253	26.23535	26.21327	1.831752117	-1.013811111	Higher in PyMT Control	G5E866;Q99NB9	G5E866;Q99NB9
28.68311	28.509	28.63893	27.43936	27.70636	27.64581	3.336810543	-1.013167699	Higher in PyMT Control	G3X8T3;P16675;A2	G3X8T3;P16675
26.76446	26.78955	26.78955	25.75685	25.81909	25.76192	5.885579943	-1.001897176	Higher in PyMT Control	H7BX95;Q6PDM2;F	H7BX95;Q6PDM2
25.34773	25.1488	25.43936	23.75939	24.391	24.78457	1.491026756	-1.000313441	Higher in PyMT Control	Q9CR60	Q9CR60
24.32389	24.34435	24.1795	22.95837	23.33756	23.55215	2.278765773	-0.999883652	Higher in PyMT Control	P09470;F6QCP8	P09470;F6QCP8
25.79452	25.41054	25.49228	24.47383	24.35783	24.8811	2.140762638	-0.994857788	Higher in PyMT Control	Q9EPL2	Q9EPL2
27.47073	27.78332	27.77077	26.55069	26.78955	26.73892	2.843019073	-0.98188591	Higher in PyMT Control	Q9WVJ3	Q9WVJ3
25.34773	24.7024	25.10149	24.12534	23.99335	24.10149	2.149845451	-0.977148692	Higher in PyMT Control	P51859;E0CXA0;E0	P51859;E0CXA0;E
24.47383	24.85757	23.91342	23.44253	23.46762	23.45513	1.603909661	-0.959845861	Higher in PyMT Control	Q8VH51;F7AA45;E9	Q8VH51;F7AA45;E
24.89966	25.26428	24.90885	23.94949	24.28912	24.04427	2.390515364	-0.929968516	Higher in PyMT Control	Q9JKN1	Q9JKN1
27.46295	27.45513	27.22619	26.16997	26.47692	26.71293	2.213858652	-0.928148905	Higher in PyMT Control	Q8BTS0;Q61656;S4	Q8BTS0;Q61656;S
27.67976	27.90885	27.30661	26.95394	26.82639	26.35448	1.656743096	-0.920140584	Higher in PyMT Control	Q922R8	Q922R8
27.11148	26.83846	26.87408	26.038	25.97378	26.1035	3.191375042	-0.902915319	Higher in PyMT Control	Q9Z204;A0A1D5RL	Q9Z204
26.69975	26.58978	26.39428	25.69975	25.67573	25.60399	3.182697942	-0.901444117	Higher in PyMT Control	Q9D0J8	Q9D0J8
27.53148	27.21697	27.34942	26.38607	26.61807	26.41054	2.800909651	-0.894396464	Higher in PyMT Control	P10922	P10922
26.5285	25.85757	26.40892	25.19084	25.50446	25.43936	1.754259077	-0.886777242	Higher in PyMT Control	Q8VD65	Q8VD65
27.88577	28.09144	27.58978	27.06085	27.10149	26.75175	2.075432506	-0.884297689	Higher in PyMT Control	Q9D0E1;B8JK33;B8	Q9D0E1;B8JK33;B
26.34773	25.81665	26.12534	25.20583	25.03171	25.43619	1.968017211	-0.872000376	Higher in PyMT Control	Q9CX86	Q9CX86
25.73635	25.91798	26.61807	25.09346	25.42342	25.14102	1.410366682	-0.871498108	Higher in PyMT Control	E9Q5H2;E9PZF5;P9	E9Q5H2;E9PZF5;P
29.71947	29.76288	29.66119	28.88285	28.76762	28.88577	4.23350196	-0.869100571	Higher in PyMT Control	Q8VEK3	Q8VEK3
24.18707	24.67034	24.48616	23.5285	23.88577	23.33756	1.808126543	-0.863911311	Higher in PyMT Control	A2AHD1;Q9Z0G4	A2AHD1;Q9Z0G4
24.14102	24.48616	24.30313	23.45513	23.40406	23.48001	2.969498518	-0.863705317	Higher in PyMT Control	G3UZX6;H7BWX9;D	G3UZX6;H7BWX9;
26.26428	26.48155	26.46139	25.58118	25.75175	25.28912	2.32292774	-0.861720403	Higher in PyMT Control	Q9EST5	Q9EST5
26.61807	26.58978	26.13712	25.46139	25.56964	25.73117	2.105684349	-0.860919952	Higher in PyMT Control	P25206	P25206
25.33415	25.82639	25.52552	24.64306	24.45513	25.05672	1.679833133	-0.843714396	Higher in PyMT Control	Q61881;D3Z6N3;D3	Q61881;D3Z6N3
25.77455	25.61526	26.29263	24.85757	24.97158	25.32389	1.564025296	-0.843135834	Higher in PyMT Control	Q9D1B1	Q9D1B1
24.10149	24.08537	24.12534	23.09023	23.26785	23.42982	2.982634811	-0.84143575	Higher in PyMT Control	Q5SUT0;Q5SUS9;Q	Q5SUT0;Q5SUS9;
30.74535	30.52962	30.62854	29.74695	29.72273	29.91171	3.210342037	-0.840705236	Higher in PyMT Control	P20060	P20060
27.57542	27.75175	27.73247	26.75175	26.83846	26.96499	3.252388297	-0.834813436	Higher in PyMT Control	Q99K48	Q99K48
31.4919	31.57542	31.48712	30.64581	30.68813	30.7211	4.684325941	-0.833133062	Higher in PyMT Control	G3X9T8;G3X8Q5;G3	G3X9T8;G3X8Q5
27.13124	27.38278	26.97596	26.47073	26.56238	25.97158	1.715593855	-0.828430176	Higher in PyMT Control	P62315	P62315
27.43936	27.24445	27.391	26.61807	26.61807	26.37451	2.919733572	-0.82138888	Higher in PyMT Control	F8VQJ3;P02468;F6T	F8VQJ3;P02468
25.93832	25.44569	25.87643	25.09748	24.80931	24.89966	2.007723504	-0.817993164	Higher in PyMT Control	S4R1K7;S4R1P7;G3	S4R1K7;S4R1P7;G3
29.41337	29.33031	29.48424	28.56456	28.71947	28.51277	3.349898295	-0.810371399	Higher in PyMT Control	Q02819;A0A1C7CY	Q02819;A0A1C7CY
25.38443	25.81421	25.49838	24.84805	24.73892	24.7024	2.386923124	-0.802551905	Higher in PyMT Control	A0A140T8V5;P1791	A0A140T8V5;P179
26.1035	26.01052	25.77706	25.239	25.07316	25.1946	2.725428978	-0.794770559	Higher in PyMT Control	G3UW70;Q9D1L9	G3UW70;Q9D1L9
26.64581	26.56092	26.73892	25.59833	26.239	25.73635	1.765791143	-0.790665309	Higher in PyMT Control	P35550;A0A140LIR	P35550

26.72598	26.68646	26.78955	25.90426	26.06085	25.87173	3.559707271	-0.788380941	Higher in PyMT Control	P12265;Q99KJ6;D3Y	P12265
27.07112	27.08132	27.12139	26.28736	26.21141	26.4186	3.638838067	-0.78548495	Higher in PyMT Control	O88207	O88207
26.9428	26.61807	26.71293	26.0041	25.99981	25.92931	2.846598869	-0.78019015	Higher in PyMT Control	Q60668;F6ZV59;G5	Q60668;F6ZV59;G
27.63201	27.37451	27.63201	26.71293	26.80193	26.80193	2.982837689	-0.773910522	Higher in PyMT Control	A0A1B0GR11;Q930	A0A1B0GR11;Q93
25.2571	25.391	25.46451	24.71293	24.76951	24.31008	2.103904792	-0.773362478	Higher in PyMT Control	P62317	P62317
24.42342	24.49228	23.83846	23.57542	23.51653	23.36453	1.617174647	-0.7658933	Higher in PyMT Control	Q9DAU1	Q9DAU1
25.89273	25.7543	25.92252	25.08537	25.06085	25.1488	3.701766927	-0.758174896	Higher in PyMT Control	Q3UKN6;P81117	Q3UKN6;P81117
24.75939	25.06497	25.34773	24.34435	24.15653	24.417	1.80072737	-0.751402537	Higher in PyMT Control	D3YTS4;Q9JHS3	D3YTS4;Q9JHS3
27.28912	27.16997	26.80193	26.60399	26.27498	26.16806	1.700545466	-0.737998327	Higher in PyMT Control	Q8VDW0;D6RHT5	Q8VDW0;D6RHT5
28.64581	29.08132	28.76129	28.16997	28.00838	28.09648	2.224993882	-0.737862905	Higher in PyMT Control	E9Q668;O08665;O8	E9Q668;O08665
23.29614	23.32389	23.24916	22.88017	22.29894	22.48247	1.882713928	-0.735870997	Higher in PyMT Control	Q9EP52	Q9EP52
27.63201	27.63201	27.3662	26.75175	26.95394	26.72598	2.518243546	-0.732848485	Higher in PyMT Control	Q8C2Q7;O35737	Q8C2Q7;O35737
27.01903	26.85043	26.68646	26.12731	26.11744	26.12928	2.787097169	-0.727299372	Higher in PyMT Control	Q80XR6;Q20BD0;Q	Q80XR6;Q20BD0;
25.34773	25.78706	25.62923	25.04843	25.02327	24.51051	1.542734798	-0.727272034	Higher in PyMT Control	Q9Z1G3	Q9Z1G3
23.87643	24.01903	23.99335	22.96895	23.54038	23.21401	1.868807281	-0.721822103	Higher in PyMT Control	Q62011;A8Y5F6	Q62011;A8Y5F6
26.77706	26.63201	27.06085	26.08335	26.20769	26.01903	2.195875059	-0.719949722	Higher in PyMT Control	P70699;F6R5R5;A2	P70699
25.69445	25.78207	25.70504	24.72858	25.11347	25.18707	2.111447122	-0.71747907	Higher in PyMT Control	Q9DCW2;D3Z7J3	Q9DCW2;D3Z7J3
27.34096	27.44727	27.39917	26.61807	26.73892	26.69975	3.950437934	-0.71021843	Higher in PyMT Control	Q64191;B7ZNK6	Q64191;B7ZNK6
27.58978	27.85043	27.62506	26.83846	27.10149	26.99766	2.498845013	-0.709218979	Higher in PyMT Control	Q7TPV4	Q7TPV4
27.11148	27.391	27.11148	26.39591	26.58978	26.52402	2.520162198	-0.701412837	Higher in PyMT Control	A2AMH5;A2AMH3;	A2AMH5;A2AMH3
25.33756	25.19084	24.93157	24.16423	24.51653	24.68646	1.639346273	-0.697584788	Higher in PyMT Control	G3UXT7;Q8CFQ9;P	G3UXT7;Q8CFQ9;
24.91342	24.37119	24.46762	23.79947	24.1332	23.72858	1.541524083	-0.696994781	Higher in PyMT Control	J3QN51;J3QPT3;B0	J3QN51;J3QPT3;B
24.66492	24.58118	24.5285	24.03591	23.83846	23.81909	3.014919457	-0.693717321	Higher in PyMT Control	Q9DCC5;P23198;D3	Q9DCC5;P23198
26.82639	26.86231	26.9428	26.28912	26.17188	26.09346	3.319163431	-0.692343394	Higher in PyMT Control	Q9D0F3	Q9D0F3
29.7162	29.95394	29.89446	29.1795	29.14346	29.16758	3.177244391	-0.691355387	Higher in PyMT Control	Q9Z0J0	Q9Z0J0
25.61526	25.62923	25.27142	24.94056	24.64306	24.86703	2.022658439	-0.688421885	Higher in PyMT Control	P62305;E9Q4F4;E9	P62305;E9Q4F4
28.95394	29.15557	28.81726	28.31095	28.21697	28.35363	2.518591384	-0.681739807	Higher in PyMT Control	Q9Z2X1;J3QM80;J3	Q9Z2X1
29.62854	29.4512	29.57903	28.80809	28.88867	28.92025	3.393930467	-0.680585861	Higher in PyMT Control	Q5NCU4;A0A1L1SS	Q5NCU4;A0A1L1S
26.33245	26.38278	26.38607	25.81421	25.85757	25.391	1.977320465	-0.679506938	Higher in PyMT Control	Q99M71;A0A1Y7VK	Q99M71
29.67472	29.66119	29.67472	28.98685	28.97049	29.01903	5.849189112	-0.678087234	Higher in PyMT Control	Q61398;D3YUE2;A	Q61398;D3YUE2;A
26.12139	26.07521	25.87408	25.12534	25.24264	25.68913	1.632921071	-0.671194077	Higher in PyMT Control	P32067;A2AR07;D6	P32067;A2AR07
29.77706	29.65096	29.6764	28.98414	28.93157	29.1795	2.859233792	-0.669739405	Higher in PyMT Control	P48678;D3YUF7	P48678
29.96774	29.94	30.00838	29.24672	29.32603	29.34308	4.314409394	-0.666765849	Higher in PyMT Control	P18242;F8WIR1;F6	P18242;F8WIR1;F
24.49838	24.55801	24.50446	23.68646	23.89504	24.00196	2.652305045	-0.659130732	Higher in PyMT Control	Q9QYY7	Q9QYY7
27.98685	27.92025	28.0296	27.1795	27.42342	27.35783	2.935609042	-0.658650716	Higher in PyMT Control	P47879;H3BLB7;H3	P47879;H3BLB7
30.90455	31.00169	30.83092	30.28692	30.26472	30.21928	3.599666221	-0.655412674	Higher in PyMT Control	J3QNY1;B2RXM2;A	J3QNY1;B2RXM2;
28.3869	28.21234	28.31527	27.67976	27.66628	27.61105	3.543648571	-0.65247345	Higher in PyMT Control	O88325	O88325
24.59263	24.54038	24.93157	24.17188	24.11744	23.82881	1.790809888	-0.648817698	Higher in PyMT Control	Q80UM7	Q80UM7
27.84446	27.89157	28.15074	27.41538	27.46295	27.07112	1.843093644	-0.645771027	Higher in PyMT Control	P29416	P29416
24.41054	24.8811	24.46762	23.72858	23.99335	24.10949	1.584311897	-0.642616908	Higher in PyMT Control	P83887;Q8VCK3	P83887;Q8VCK3
30.4334	30.36202	30.41337	29.78644	29.82639	29.68479	3.758283834	-0.637062073	Higher in PyMT Control	P24369	P24369
28.42342	28.49762	28.45905	27.75175	27.87408	27.87994	3.733981094	-0.624774933	Higher in PyMT Control	Q640N1;A0A0R4J0	Q640N1
29.06599	28.92025	29.19835	28.33671	28.51653	28.45905	2.535411152	-0.624105453	Higher in PyMT Control	Q61937;Q9DAY9;Q	Q61937;Q9DAY9;
27.97049	28.14589	28.3662	27.46295	27.53148	27.63893	2.099241718	-0.616404851	Higher in PyMT Control	P23780;A0A1L1SSJ	P23780
26.89735	26.52103	26.46139	25.98685	26.14492	25.90426	1.79230616	-0.614582062	Higher in PyMT Control	Q921M3	Q921M3
28.63547	28.56456	28.57903	28.09648	27.96499	27.87994	3.110055414	-0.612552643	Higher in PyMT Control	A0A087WPL5;E9QN	A0A087WPL5;E9Q
27.08132	27.00838	27.15074	26.56384	26.49686	26.34604	2.878204765	-0.611232758	Higher in PyMT Control	G5E924;Q8R081;G3	G5E924;Q8R081;G
25.1946	25.14492	24.94056	24.40406	24.73376	24.31008	1.816583985	-0.610726674	Higher in PyMT Control	A0A0R4J138;P5042	A0A0R4J138;P504
22.56384	22.69073	22.44506	21.94235	22.20658	21.74714	1.78713016	-0.601184209	Higher in PyMT Control	Q9D771	Q9D771
28.39917	28.35363	28.45513	27.84446	27.76446	27.80809	4.047011259	-0.596978505	Higher in PyMT Control	Q8BFR4;A0A1W2P8	Q8BFR4
33.7751	33.79459	33.78488	33.1916	33.15436	33.21784	5.190204464	-0.596921285	Higher in PyMT Control	Q01853	Q01853
26.92025	27.18896	26.82639	26.55655	26.29439	26.29439	1.89118564	-0.596756617	Higher in PyMT Control	G3XA21;E0CZ22;D3	G3XA21;E0CZ22;D
24.91342	25.32732	24.87643	24.33074	24.52253	24.51653	1.679220449	-0.582454681	Higher in PyMT Control	Q8BL66	Q8BL66
27.83244	27.89735	27.86821	27.15074	27.23535	27.47846	2.351061334	-0.577814738	Higher in PyMT Control	Q921F2;A0A087WR	Q921F2;A0A087W
26.52552	26.71293	26.81421	26.04218	26.02327	26.26964	2.107796077	-0.572524389	Higher in PyMT Control	Q8VEH3;F6QKK2	Q8VEH3;F6QKK2
25.85281	26.07521	26.1035	25.42662	25.48922	25.4008	2.622899227	-0.571622213	Higher in PyMT Control	P27048;P63163;A0	P27048;P63163;A
24.42982	24.02749	24.22435	23.57542	23.66492	23.72858	1.995699399	-0.570910772	Higher in PyMT Control	Q60972	Q60972
26.81421	26.92025	26.53297	26.04427	26.20583	26.31872	1.804817821	-0.566203435	Higher in PyMT Control	Q9DB73;G3UZG6;G	Q9DB73;G3UZG6
25.37119	25.41377	25.58692	25.05258	24.77455	24.84805	2.223582957	-0.565568288	Higher in PyMT Control	P24638;B7ZCF4;B72	P24638
25.9428	25.74663	26.05051	25.42982	25.317	25.30313	2.349682029	-0.563330968	Higher in PyMT Control	Q78XF5	Q78XF5
26.78955	26.65949	26.54628	26.16615	26.2021	25.94503	2.201293182	-0.560680389	Higher in PyMT Control	E9Q1S3;Q01405	E9Q1S3;Q01405
28.53519	28.46295	28.4746	27.85043	28.06085	27.88577	2.902433797	-0.55856514	Higher in PyMT Control	Q9D1D4;A0A1Y7VN	Q9D1D4;A0A1Y7V
27.39917	27.34942	27.42342	26.81421	26.85043	26.85043	4.611408735	-0.552314123	Higher in PyMT Control	P17439;A0A0G2JDK	P17439;A0A0G2JD
26.90885	27.12139	26.97596	26.45199	26.4567	26.47537	2.991126054	-0.540714264	Higher in PyMT Control	P62320;A0A1W2P7	P62320;A0A1W2P
27.4073	27.54628	27.15074	26.75175	26.86231	26.87408	1.934680559	-0.538727442	Higher in PyMT Control	Q9CQ22;A0A0A6YX	Q9CQ22
26.20583	26.21327	26.15267	25.45513	25.72598	25.78706	2.171407418	-0.534531275	Higher in PyMT Control	O35215;G3UZN1;G	O35215;G3UZN1;
25.86231	25.89504	25.81421	25.37451	25.48616	25.12928	2.08898416	-0.527204514	Higher in PyMT Control	E9PUX0;Q80WJ7;F6	E9PUX0;Q80WJ7
29.28692	29.16279	29.21002	28.49762	28.77077	28.81115	2.131921851	-0.526727041	Higher in PyMT Control	E9QN70;P02469	E9QN70;P02469
29.58978	29.708	29.6372	29.11892	29.13614	29.12139	3.932400706	-0.519510269	Higher in PyMT Control	P61979;B2M1R6;A	P61979;B2M1R6;
24.8811	24.87173	25.00624	24.33756	24.48616	24.38443	2.955431925	-0.516978582	Higher in PyMT Control	E9QAN4;G3UW47;	E9QAN4;G3UW47
26.04635	26.0041	26.06907	25.39754	25.4008	25.77957	1.795133644	-0.513872147	Higher in PyMT Control	Q9CXE7;E9PXY3;D3	Q9CXE7;E9PXY3
28.20303	28.1795	28.12633	27.71293	27.73247	27.55362	2.928729717	-0.503278732	Higher in PyMT Control	P54116	P54116
27.13124	26.99766	27.34942	26.60399	26.61807	26.75175	1.947440606	-0.501502991	Higher in PyMT Control	Q3TCN2	Q3TCN2
29.34308	29.21002	29.391	28.71293	28.81726	28.91171	2.500476726	-0.500731786	Higher in PyMT Control	P37889	P37889
27.2803	27.07112	27.00838	26.56238	26.77706	26.55215	1.946622027	-0.489401499	Higher in PyMT Control	Q99KF1;A0A286YD	Q99KF1;A0A286Y
28.20303	28.01372	28.37036	27.73892	27.69975	27.71293	2.00391063	-0.478501638	Higher in PyMT Control	P12023	P12023
28.78019	28.91741	28.74535	28.391	28.3452	28.28472	2.833605662	-0.474013011	Higher in PyMT Control	Q60963;E9Q330;E9	Q60963;E9Q330
38.95535	39.01109	39.06477	38.50468	38.59432	38.51206	3.424183516	-0.473379771	Higher in PyMT Control	A0A087WR50;Q3U	A0A087WR50;Q3
26.09144	26.11744	26.06907	25.76446	25.67034	25.43619	2.049392163	-0.468988419	Higher in PyMT Control	A0A0R4J1Z3;Q9CR	A0A0R4J1Z3;Q9C
26.61807	26.65949	26.56384	26.09144	26.07928	26.26428	2.686674225	-0.468798319	Higher in PyMT Control	Q61235;B7ZNU9	Q61235;B7ZNU9
27.56819	27.56092	27.58978	27.05051	27.10149	27.16997	3.701290722	-0.465640386	Higher in PyMT Control	Q9CQU0	Q9CQU0
23.96719	23.77957	23.90426	23.46762	23.37782	NaN	2.004130625	-0.460949898	Higher in PyMT Control	D3YWD1;J3QQ16;A	D3YWD1;J3QQ16;
27.56092	27.47846	27.69975	27.15074	27.13124	27.13124	2.614580512	-0.441973368	Higher in PyMT Control	G5E8D6;Q9WVJ9;E	G5E8D6;Q9WVJ9;
25.74663	25.75939	25.77455	25.21697	25.28912	25.45199	2.488790422	-0.440828959	Higher in PyMT Control	Q9CQS8;E9PW43	Q9CQS8;E9PW43
31.76129	31.78097	31.90813	31.31419	31.39305	31.45807	2.637020667	-0.4283638	Higher in PyMT Control	E9PZ00;Q8BFQ1;K3	E9PZ00;Q8BFQ1;K
26.49381	26.47228	26.31354	25.96058	26.07316	25.96499	2.490044767	-0.426965714	Higher in PyMT Control	Q91YZ8;A3KFU5;A3	Q91YZ8;A3KFU5;A
29.10899	29.07368	29.08639	28.60752	28.62854	28.76762	2.92405072	-0.421789805	Higher in PyMT Control	P09405	P09405
30.81574	30.57542	30.81574	30.31959	30.33031	30.34096	2.13935466	-0.405347824	Higher in PyMT Control	Q99JR5;H3BJ97;H3	Q99JR5;H3BJ97
35.83045	35.8186	35.75782	35.39879	35.4427	35.36006	3.593167862	-0.401772817	Higher in PyMT Control	E9PZ16	E9PZ16
28.83545	28.93438	28.74535	28.50522	28.34096	28.4746	2.232301617	-0.398136139	Higher in PyMT Control	Q8CGK3	Q8CGK3
30.11148	30.02168	30.11148	29.71293	29.71293	29.62506	3.164378434	-0.397907257	Higher in PyMT Control	A0A0R4J0Z1;P0800	A0A0R4J0Z1;P080
26.83846	26.86231	26.83846	26.57542	26.43301	26.33756	2.334437697	-0.397743861	Higher in PyMT Control	Q60770;A0A0G2JE	Q60770
30.66289	30.61105	30.67976	30.20769	30.36202	30.21928	2.710004386	-0.388234456	Higher in PyMT Control	O35887;G3UWV3;G	O35887;G3UWV3;
29.42142	29.53519	29.49381	29.07623	29.21928	29.0296	2.323914823	-0.375104904	Higher in PyMT Control	Q8R180;A0A1Y7VJN	Q8R180
30.4334	30.33031	30.4334	30.00838	30.04791	30.02168	3.297456741	-0.373048147	Higher in PyMT Control	Q3U9N4;P28798;H	Q3U9N4;P28798;
25.15267	25.1055	25.08537	24.76446	24.7543	24.71816	3.952775939	-0.368873596	Higher in PyMT Control	Q6PGL7;A0A0N4SU	Q6PGL7;A0A0N4S
30.67976	30.61105	30.60222	30.33031	30.28692	30.18423	2.730182931	-0.363855362	Higher in PyMT Control	P97384;D3Z7U0	P97384;D3Z7U0
31.46782	31.48712	31.53427	31.0927	31.19013	31.14224	3.303736739	-0.354707718	Higher in PyMT Control	Q9QZF2	Q9QZF2
33.35678	33.36984	33.38278	33.07368	33.02993	32.96258	3.329740214	-0.347738902	Higher in PyMT Control	P20029	P20029
32.75732	32.80424	32.89374	32.47266	32.51559	32.46782	2.832712767	-0.333080292	Higher in PyMT Control	F8WIT2	F8WIT2
33.33031	33.33031	33.24785	33.01339	33.01172	32.93473	2.952184517	-0.316210429	Higher in PyMT Control	P08113;F7C312	P08113;F7C312
31.36724	31.39816	31.40832	31.12386	31.10525	31.0544	3.598140177	-0.296735128	Higher in PyMT Control	Q07797;E9Q5X5	Q07797
36.36496	36.38439	36.36169	36.0852	36.11846	36.09113	4.58565455	-0.272085826	Higher in PyMT Control	P07356;B0V2N5;B0	P07356;B0V2N5;B
30.14831	30.18423	30.17236	30.42342	30.50142	30.45317	3.5026188	0.29103597	Higher in PyMT RT	Q8BP47	Q8BP47
30.78488	30.82335	30.79266	31.11148	31.08639	31.0927	4.537818298	0.296559016	Higher in PyMT RT	Q9DBJ1;O70250	Q9DBJ1

28.44727	28.41538	28.43936	28.72598	28.70306	28.76446	3.895835385	0.297161738	Higher in PyMT RT	A0A0R4IZW8;O884	A0A0R4IZW8;O884
32.89374	32.91171	32.83281	33.13614	33.18719	33.21928	3.048744402	0.30144755	Higher in PyMT RT	Q9JHU4;F6ZX84	Q9JHU4
30.74535	30.71293	30.72923	30.96774	31.08639	31.04791	2.96406423	0.304843903	Higher in PyMT RT	P62754	P62754
28.77706	28.78019	28.82335	29.07877	29.09396	29.12386	3.972297781	0.305330276	Higher in PyMT RT	P34022;H7BX22	P34022;H7BX22
29.92592	29.86673	29.91171	30.23078	30.23078	30.17236	3.520237613	0.309848785	Higher in PyMT RT	Q99K85;Q3U6K9;E9	Q99K85;Q3U6K9
30.62854	30.57542	30.57542	30.83846	30.94	30.95394	2.846801529	0.317668915	Higher in PyMT RT	Q8CGC7;A0A0A6YW	Q8CGC7
31.50616	31.5389	31.4919	31.87921	31.81955	31.79266	3.399870808	0.318150202	Higher in PyMT RT	P40124;B1ARS0	P40124
27.90885	27.93157	27.86231	28.14589	28.27142	28.24898	2.738859445	0.321188609	Higher in PyMT RT	P00493	P00493
34.03485	34.00169	34.02663	34.38921	34.33031	34.31013	3.630534177	0.322157542	Higher in PyMT RT	P10126;D3YZ68;D3	P10126;D3YZ68;D3
31.08005	31.02828	31.0927	31.31959	31.44825	31.41337	2.786517114	0.326725642	Higher in PyMT RT	Q61753;F6ZSB7	Q61753
29.69644	29.67303	29.79885	30.03486	30.09899	30.02168	2.715396135	0.329067866	Higher in PyMT RT	O70503	O70503
28.69644	28.64238	28.76129	29.04009	29.0531	29.0296	3.204035092	0.340895971	Higher in PyMT RT	Q99MN1;Q8R2P8	Q99MN1;Q8R2P8
27.52402	27.51653	27.45513	27.85638	27.85043	27.81421	3.747058473	0.341779073	Higher in PyMT RT	Q9CZ30;B1AYJ9	Q9CZ30
32.69851	32.74735	32.71088	33.14224	33.02003	33.02828	2.917179632	0.344601949	Higher in PyMT RT	P18760;F8WGL3	P18760;F8WGL3
29.98142	30.00838	29.91171	30.31959	30.27586	30.34096	3.245630401	0.344966253	Higher in PyMT RT	Q99JZ4;P36536;A0A	Q99JZ4;P36536
30.36202	30.26472	30.33031	30.67976	30.67976	30.64581	3.463093462	0.349426905	Higher in PyMT RT	Q9CZD3	Q9CZD3
28.64238	28.66289	28.63201	29.05051	28.96499	28.97869	3.637258563	0.352308273	Higher in PyMT RT	Q62426	Q62426
32.0414	32.11148	32.2106	32.48712	32.42842	32.52262	2.506351304	0.358229319	Higher in PyMT RT	P17751;H7BXC3	P17751;H7BXC3
30.4334	30.41337	30.55728	30.87555	30.80809	30.81574	2.734607933	0.365107854	Higher in PyMT RT	E9Q5G3;A0A1L1SR	E9Q5G3;A0A1L1SR
28.90885	28.80501	28.87994	29.26919	29.18187	29.24898	3.08619317	0.368747711	Higher in PyMT RT	P67984	P67984
31.26472	31.20769	31.12386	31.5527	31.55728	31.59335	2.999124015	0.369016647	Higher in PyMT RT	P68040	P68040
27.86821	28.02433	27.90311	28.30226	28.26696	28.35783	2.649380856	0.377134323	Higher in PyMT RT	P63168;Q80ZS7	P63168
29.69478	29.71457	29.76604	30.12386	30.13614	30.07368	3.760834301	0.386096319	Higher in PyMT RT	Q3TGU7;P50580	Q3TGU7;P50580
32.31149	32.44825	32.36984	32.85155	32.69851	32.73933	2.510997374	0.386601766	Higher in PyMT RT	P14069	P14069
27.79576	27.73892	27.90885	28.21234	28.20303	28.18896	2.810222805	0.386933645	Higher in PyMT RT	P83882;A0A0A6YW	P83882;A0A0A6YW
29.46684	29.48808	29.34731	29.82031	29.81268	29.84296	3.025525403	0.391241709	Higher in PyMT RT	Q00612;A3KG36;G3	Q00612;A3KG36
29.66966	29.57001	29.61456	29.94	29.98142	30.11148	2.573879266	0.392887115	Higher in PyMT RT	Q61768;E9QAK5;P3	Q61768;E9QAK5
29.30878	29.19131	29.20536	29.58978	29.64238	29.66966	3.091847426	0.398787816	Higher in PyMT RT	Q9Z0N1;A2AAW9;Q	Q9Z0N1;A2AAW9;
28.75494	28.77392	28.78332	29.0531	29.20303	29.26249	2.513705316	0.4021492	Higher in PyMT RT	P70698	P70698
28.01903	28.06599	27.97596	28.4073	28.45905	28.41135	3.713481075	0.405569077	Higher in PyMT RT	Q99KJ8	Q99KJ8
29.30226	29.15074	29.33245	29.70306	29.66628	29.64065	2.638323036	0.40818278	Higher in PyMT RT	P60867	P60867
31.88649	31.75732	31.84595	32.25912	32.2535	32.20478	3.212125635	0.409210841	Higher in PyMT RT	P35700;B1AXW5;B1	P35700;B1AXW5;B
28.20769	28.24445	28.20303	28.61807	28.61105	28.70306	3.715640354	0.425666173	Higher in PyMT RT	E9Q586;E9Q3M3;O	E9Q586;E9Q3M3;
29.62157	29.52776	29.509	29.94	30.04791	29.95394	3.037279767	0.427841187	Higher in PyMT RT	Q9R0P5	Q9R0P5
30.14831	30.08639	30.14831	30.42342	30.65438	30.59335	2.398665921	0.429377874	Higher in PyMT RT	P63325;Q3UW83	P63325;Q3UW83
30.4919	30.39305	30.50142	30.87555	30.91883	30.88285	3.498747311	0.430287043	Higher in PyMT RT	P35279	P35279
29.99496	29.95394	29.84595	30.35153	30.33031	30.41337	2.980117498	0.43345197	Higher in PyMT RT	Q8C483;P26638;A2	Q8C483;P26638;A
29.37036	29.32174	29.27808	29.7162	29.7162	29.85935	2.890570138	0.440524419	Higher in PyMT RT	Q61171;D3Z4A4	Q61171;D3Z4A4
28.74855	28.66289	28.76446	29.17712	29.14346	29.1866	3.693828572	0.443759918	Higher in PyMT RT	P61514;A0A1D5RL8	P61514;A0A1D5RL
28.23535	28.07623	27.97049	28.60399	28.62157	28.41135	1.937507981	0.451615016	Higher in PyMT RT	Q9JJI8	Q9JJI8
28.43539	28.41941	28.4746	28.77706	28.97049	28.94559	2.71048939	0.454577764	Higher in PyMT RT	P62900;A0A0A6YX2	P62900;A0A0A6YX
25.1488	25.317	25.23169	25.73892	25.81421	25.51653	1.964795016	0.457391739	Higher in PyMT RT	Q9ER41;F6VK94;D6	Q9ER41;F6VK94;D
31.41337	31.32496	31.30334	31.79653	31.88649	31.73731	2.953273454	0.459553401	Higher in PyMT RT	Q62465	Q62465
30.67135	30.55728	30.61105	31.04791	31.08639	31.08639	3.697221709	0.460336685	Higher in PyMT RT	P35441;E9PWF0;Q	P35441
27.92592	27.85043	28.09144	28.41538	28.35363	28.48616	2.335760507	0.462460836	Higher in PyMT RT	P21550;Q5SX59;J3	P21550
27.5969	27.42342	27.42342	27.86231	27.88577	28.09648	2.110977253	0.466932933	Higher in PyMT RT	E9PYH2;Q91V12	E9PYH2;Q91V12
28.42342	28.4073	28.35783	28.6764	28.88577	29.0531	1.896144484	0.475570679	Higher in PyMT RT	Q8C522;A0A1L1SSA	Q8C522
23.85757	23.70767	23.75939	24.26785	24.34435	24.1488	2.578182096	0.478791555	Higher in PyMT RT	H7BWZ1;D3YW97;	H7BWZ1;D3YW97;
27.95947	27.71947	27.91456	28.29789	28.26696	28.48616	2.072516795	0.485833486	Higher in PyMT RT	Q5UE59;E9Q7C9;Q	Q5UE59;E9Q7C9;Q
33.72517	33.74535	33.80424	34.25491	34.26192	34.22647	4.323851927	0.489513397	Higher in PyMT RT	P58252	P58252
24.79452	24.55801	24.80931	25.07316	25.24627	25.32732	1.956585975	0.494969686	Higher in PyMT RT	Q61655;Q8R3C7;Q	Q61655;Q8R3C7;Q
29.96774	30.02168	29.95394	30.52962	30.45317	30.45317	3.95749681	0.497533162	Higher in PyMT RT	Q6ZWZ6;F7AEH4;A	Q6ZWZ6;F7AEH4;A
25.06907	25.27498	25.12139	25.67842	25.68646	25.60116	2.751993248	0.500198364	Higher in PyMT RT	Q91ZR1;P56371	Q91ZR1
29.55179	29.44332	29.52589	30.02168	29.98142	30.02168	3.842693563	0.50125885	Higher in PyMT RT	P61164	P61164
28.52028	28.41538	28.53519	28.99226	28.99496	28.99766	3.743153475	0.504674911	Higher in PyMT RT	Q9ER72;A0A140LIB	Q9ER72
24.64856	24.77455	24.79452	25.15267	25.43619	25.14492	2.050553259	0.505385081	Higher in PyMT RT	P70202	P70202
28.34096	28.15557	28.07112	28.63547	28.67303	28.79576	2.271588675	0.512203852	Higher in PyMT RT	Q9D819	Q9D819
27.38278	27.47073	27.65267	27.87994	28.05569	28.12633	2.062513737	0.518592199	Higher in PyMT RT	P28474;A0A0G2JGS	P28474
23.64306	23.68646	23.66492	24.20955	24.20955	24.15653	4.771368553	0.527065913	Higher in PyMT RT	Q8BYI8	Q8BYI8
21.63201	NaN	21.44253	22.15808	22.01563	22.0258	1.979250947	0.529236158	Higher in PyMT RT	Q8R317	Q8R317
27.79576	27.81421	27.92592	28.43539	28.41538	28.27586	2.92104831	0.530251185	Higher in PyMT RT	Q60817;P70670	Q60817;P70670
24.16423	23.89504	24.12534	24.74919	24.53445	24.49838	2.010809178	0.53247261	Higher in PyMT RT	A0A0N4SWD0;Q9C	A0A0N4SWD0;Q9C
29.4174	29.17236	29.31095	29.87994	29.89735	29.73892	2.462740937	0.538502375	Higher in PyMT RT	F7DBB3;Q3URZ6	F7DBB3
24.42342	24.14102	24.42342	24.90426	25.00624	24.78457	2.124822427	0.569068909	Higher in PyMT RT	P99025	P99025
24.83846	24.81421	24.67573	25.54038	25.08942	25.42662	1.786588263	0.576007843	Higher in PyMT RT	D3Z390;Q921C5;A0	D3Z390;Q921C5
28.47846	28.21697	28.56092	28.88867	29.06856	29.05827	2.103669742	0.586381912	Higher in PyMT RT	P63005;Q5SW16	P63005
22.36185	22.49228	NaN	23.07398	22.96543	NaN	1.701431534	0.592638016	Higher in PyMT RT	P07742	P07742
27.98142	28.16039	27.81421	28.55362	28.54995	28.64238	2.333455408	0.596645355	Higher in PyMT RT	G3UYV7;P62858	G3UYV7;P62858
24.28207	24.239	24.33756	25.08132	24.66492	24.90426	2.068703106	0.597291946	Higher in PyMT RT	P62878	P62878
24.67034	24.80931	24.58692	25.23535	25.31354	25.31008	2.992618165	0.597468058	Higher in PyMT RT	P55821	P55821
24.50446	24.31008	24.42982	24.85757	24.97158	25.21697	2.124413004	0.600589116	Higher in PyMT RT	Q9CZW5	Q9CZW5
28.10149	28.19366	28.06599	28.65267	28.78332	28.77077	3.39715848	0.615202586	Higher in PyMT RT	P15532;Q5NC80;Q5	P15532;Q5NC80;Q
29.65267	29.53148	29.73085	30.29789	30.18423	30.28692	3.081118912	0.618015925	Higher in PyMT RT	D3YTQ9;P62843	D3YTQ9;P62843
25.43301	25.15267	25.42662	26.01691	25.93607	25.92025	2.510765724	0.620309194	Higher in PyMT RT	A0A087WQS2;Q9C	A0A087WQS2;Q9C
27.28912	27.16997	27.00838	27.74535	27.75812	27.82639	2.725494293	0.620791753	Higher in PyMT RT	Q62371;A0A0A6YX	Q62371
26.96499	26.82639	26.93157	27.42342	27.56819	27.60399	3.077122953	0.624221166	Higher in PyMT RT	P31230;Q3UZG4;A0	P31230;Q3UZG4;A
26.34266	25.86231	26.01478	26.76446	26.68646	26.65949	1.912233715	0.630219777	Higher in PyMT RT	E9Q137	E9Q137
24.91798	25.1757	24.82396	25.44569	25.53148	25.85043	1.767696348	0.636655807	Higher in PyMT RT	Q91WG4	Q91WG4
28.15557	28.07623	28.18896	28.65949	28.82639	28.88867	2.988798456	0.651262919	Higher in PyMT RT	Q9CY58;A0A0N4SV	Q9CY58;A0A0N4SV
26.50597	25.99118	26.48001	26.99766	27.09144	26.88577	1.699293374	0.665899913	Higher in PyMT RT	B1AT82;Q9D0M1;B	B1AT82;Q9D0M1
26.18139	25.98251	26.06497	27.05051	26.5285	26.69975	1.850709969	0.683300018	Higher in PyMT RT	Q8CFU8;G3UXZ2	Q8CFU8;G3UXZ2
22.92252	NaN	22.85186	23.417	23.48001	23.81909	1.606544701	0.684838931	Higher in PyMT RT	O08582	O08582
30.08639	29.91171	30.20769	30.7211	30.65438	30.90455	2.429433343	0.691413244	Higher in PyMT RT	P14873;A2ARP8;Q9	P14873
24.91342	25.07316	25.05258	25.37782	26.05879	25.7415	1.608493495	0.71298027	Higher in PyMT RT	Q6PA06;E9QND8	Q6PA06;E9QND8
27.93157	27.48616	27.87408	28.48999	28.58978	28.43141	2.132154841	0.73979187	Higher in PyMT RT	Q3UZ39;A0A087WS	Q3UZ39
22.36986	22.58003	NaN	NaN	23.26785	23.18858	1.667234912	0.753268242	Higher in PyMT RT	Q9D892	Q9D892
24.01903	24.01903	NaN	24.78955	24.8044	24.74919	4.318884787	0.762014389	Higher in PyMT RT	Q9JHK4	Q9JHK4
27.20769	26.77706	26.93157	27.90885	27.90885	27.39917	1.652874322	0.766849518	Higher in PyMT RT	Q8R5C5;E0CZD4;E0	Q8R5C5
NaN	23.16269	22.97071	23.75939	23.72858	24.03591	1.891684322	0.774592082	Higher in PyMT RT	Q922H1	Q922H1
27.85043	27.47073	28.05569	28.27142	28.88285	28.56819	1.473727665	0.781871796	Higher in PyMT RT	O55234	O55234
24.05258	24.28912	24.36453	25.02749	25.01478	25.01903	2.948508824	0.785024643	Higher in PyMT RT	D3Z6D5;Q6TYB5	D3Z6D5;Q6TYB5
26.57398	26.65949	26.71293	27.83244	27.12139	27.3662	1.690440429	0.791208903	Higher in PyMT RT	Q9D1G1	Q9D1G1
26.2553	25.76951	26.28383	26.80193	27.06085	26.99766	2.004559269	0.850599925	Higher in PyMT RT	P39876;E9QAB2	P39876
26.82639	27.15074	26.95394	27.65267	27.89735	28.10149	2.316526509	0.90681839	Higher in PyMT RT	Q5PRF0	Q5PRF0
25.98033	26.12534	26.1795	26.55215	26.90885	27.54628	1.423010561	0.907372157	Higher in PyMT RT	G3UZ26;P50431;G3	G3UZ26;P50431;G
25.59548	25.24989	25.1055	26.2589	26.1035	26.31527	2.338538362	0.908934275	Higher in PyMT RT	Q9CQT1;F6YY88;F8	Q9CQT1;F6YY88
26.32732	26.20583	26.23535	27.20769	27.10149	27.21697	4.218686267	0.919220606	Higher in PyMT RT	Q9D0B6	Q9D0B6
24.46139	23.23169	24.02749	25.04009	24.96279	24.83364	1.331348297	1.038649877	Higher in PyMT RT	Q5SX75;Q60716;B0	Q5SX75;Q60716
24.94503	24.417	24.5285	NaN	25.64581	25.79203	1.830725846	1.088747978	Higher in PyMT RT	P62892	P62892
23.68646	22.97946	22.71188	23.94056	24.79947	24.03591	1.331809821	1.132712682	Higher in PyMT RT	Q3UHK6;B7ZNJ5;G3	Q3UHK6
25.97158	27.34942	26.71293	27.89735	27.85043	27.78332	1.363179937	1.165722529	Higher in PyMT RT	Q9CQR2	Q9CQR2
25.98251	26.78955	25.90426	27.89157	27.48616	26.83846	1.323750165	1.179957708	Higher in PyMT RT	Q52KR3;A0A286YD	Q52KR3
22.52373	23.0592	22.81127	23.63201	24.04427	24.43619	1.946405384	1.239421209	Higher in PyMT RT	Q0KL02	Q0KL02
26.12337	26.20583	27.09144	27.63201	27.69312	27.83244	1.773607334	1.245641708	Higher in PyMT RT	P63073;A0A0G2JGT	P63073;A0A0G2JG
24.52253	24.46139	24.59833	25.81909	25.99766	25.7543	4.054812	1.329601924	Higher in PyMT RT	P62274	P62274

23.96719	24.10949	24.28207	25.46762	24.98468	25.94726	2.000751876	1.346939723	Higher in PyMT RT	Q8CBY8	Q8CBY8
23.87643	22.9566	24.72858	25.22803	25.64856	25.19084	1.321056069	1.501939774	Higher in PyMT RT	Q00780	Q00780
24.90885	23.0258	24.26069	25.83605	26.34435	26.35783	1.664205218	2.114297231	Higher in PyMT RT	F2Z4A3;A0A087WR	F2Z4A3;A0A087W

Protein names	Gene names	GOBP name	GOMF name	GOCC name	KEGG name
Biliverdin reductase A	Blvra	catabolic process;cellular catabolic process;cell	biliverdin reductase activity;binding;catalytic ac	cell part;cytoplasm;intracellular part	Porphyrin and chlorophyll metabolism
Pigment epithelium-de	Serpinf1	biological regulation;negative regulation of ang	endopeptidase inhibitor activity;endopeptidase	cell part;cytoplasmic membrane-bounded vesic	
Complement factor H	Cfh	activation of immune response;biological regul	binding;carbohydrate binding;glycosaminoglyca	extracellular region	Complement and coagulation cascades;Staphyl
Agrin	Agrn	biological regulation;cell surface receptor linke	acetylcholine receptor regulator activity;bindin	basal lamina;cell junction;cell part;cell surface;	ECM-receptor interaction
Insulin-like growth fact	Igfbp7	anatomical structure development;biological ad		extracellular matrix;extracellular region;extrace	
Histone H1.1	Hist1h1a	cellular component assembly;cellular compone	binding;DNA binding;nucleic acid binding	cell part;chromatin;chromosomal part;intracell	
Lysosomal alpha-mann	Man2b1	alcohol metabolic process;behavior;carbohydra	alpha-mannosidase activity;binding;carbohydra	cell part;cytoplasmic part;intracellular membra	Lysosome;Other glycan degradation
Serotransferrin	Tf	actin cytoskeleton organization;actin filament c	binding;cation binding;ferric iron binding;ion bi	cell part;coated pit;cytoplasmic membrane-bou	Mineral absorption
Junction plakoglobin	Jup	anatomical structure development;anatomical	binding;cadherin binding;cell adhesion molecul	actin cytoskeleton;adherens junction;anchoring	Acute myeloid leukemia;Arrhythmogenic right v
Mannose-1-phosphate	Gmppa	alcohol biosynthetic process;alcohol metabolic	binding;catalytic activity;GTP binding;guanyl nu		Amino sugar and nucleotide sugar metabolism;
Predicted gene 20425	Gm20425	biological regulation;cation homeostasis;cation	binding;cation binding;enzyme inhibitor activity	cell part;coated pit;cytoplasmic membrane-bou	Protein export
Semaphorin-7A	Sema7a	biological regulation;cell surface receptor linke	receptor activity	anchored to membrane;cell part;external side c	Axon guidance
Cathepsin Z	Ctsz	anatomical structure morphogenesis;branching	catalytic activity;cysteine-type peptidase activit	cell part;cytoplasmic part;endoplasmic reticulu	Lysosome
Putative RNA-binding p	Luc7l2	biological regulation;negative regulation of bio		cell part;intracellular membrane-bounded orga	
Protein CTLA-2-alpha;U	Ctla2a	biological regulation;negative regulation of bio	cysteine-type endopeptidase inhibitor activity;c	cell part;cell projection;dendrite;extracellular r	
Cathepsin L1;Cathepsin	Ctsl	aging;anatomical structure development;autop	aminopeptidase activity;binding;catalytic activi	cell part;cell projection;cytoplasmic part;extern	Antigen processing and presentation;Lysosome
Sodium/potassium-tra	Atp1b1	biological adhesion;cell adhesion;cellular proce	active transmembrane transporter activity;ATP	apical plasma membrane;basolateral plasma m	Aldosterone-regulated sodium reabsorption;Bil
Gamma-interferon-ind	Ifi30	antigen processing and presentation;antigen pr	catalytic activity;disulfide oxidoreductase activi	cell junction;cell part;cytoplasmic part;extracel	Antigen processing and presentation
Histone H1.3	Hist1h1d	binding of sperm to zona pellucida;cell differen	binding;DNA binding;nucleic acid binding	cell part;chromatin;chromosomal part;chromos	
High mobility group pr	Hmga1	aging;biological regulation;biosynthetic process	binding;DNA binding;hormone receptor binding	cell part;chromatin;chromosomal part;heteroc	
Alpha-galactosidase A	Gla	biological regulation;carbohydrate catabolic pr	alpha-galactosidase activity;binding;carbohydra	cell part;cytoplasmic part;extracellular region;G	Galactose metabolism;Glycerolipid metabolism
Secretory carrier-assoc	Scamp1	cellular component organization;cellular compo		cell part;clathrin coated vesicle membrane;coa	
Semaphorin-3G	Sema3g	developmental process;multicellular organisma	receptor activity	cell part;extracellular region;membrane	Axon guidance
Macrophage colony-sti	Csf1	adipose tissue development;anatomical structu		cell part;cytoplasmic part;extracellular region p	Cytokine-cytokine receptor interaction;Hematc
Antigen peptide transp	Tap1	antigen processing and presentation;antigen pr	active transmembrane transporter activity;ade	cell part;cytoplasmic part;cytoplasmic vesicle m	ABC transporters;Antigen processing and prese
H/ACA ribonucleoprote	Dkc1	cellular macromolecule metabolic process;cellu	binding;carbon-oxygen lyase activity;catalytic a	Cajal body;cell part;intracellular non-membran	Ribosome biogenesis in eukaryotes
Osteopontin	Spp1	anatomical structure development;biological a	binding;cytokine activity;extracellular matrix bi	apical part of cell;cell part;cell projection;cytop	ECM-receptor interaction;Focal adhesion;Toll-li
Histone H1.5	Hist1h1b	anatomical structure development;cellular com	binding;DNA binding;nucleic acid binding	cell part;chromosomal part;intracellular memb	
Beta-2-microglobulin	B2m	antigen processing and presentation;antigen pr		cell part;cytoplasmic part;cytoplasmic vesicle m	Antigen processing and presentation
Serine/arginine-rich sp	Srsf7	biological regulation;cellular macromolecule m	binding;cation binding;ion binding;metal ion bi	cell part;intracellular membrane-bounded orga	Spliceosome
Neprilysin	Mme	aging;amine metabolic process;anatomical stru	binding;catalytic activity;cation binding;endope	axon;brush border;cell part;cell projection;cell	Alzheimer's disease;Hematopoietic cell lineage;
tRNA (cytosine(34)-C(5	Nsun2	cell cycle phase;cell cycle process;cell division;c	binding;catalytic activity;methyltransferase act	cell part;cytoplasm;cytoskeletal part;intracellul	
Cystatin-C;Cystatin (Fra	Cst3	anatomical structure development;apoptosis;b	beta-amyloid binding;binding;cysteine-type en	basement membrane;cell body;cell part;cell pr	Salivary secretion
Vascular endothelial gr	Vegfa	activation of protein kinase activity;adaptive im	binding;carbohydrate binding;chemoattractant	basement membrane;cell part;cell surface;cyto	Bladder cancer;Cytokine-cytokine receptor inte
Vasorin	Vasn	cellular process;cellular response to chemical st		cell part;cytoplasmic part;integral to membrane	
Heterogeneous nuclea	Hnrnpa1	alternative nuclear mRNA splicing, via spliceoso	binding;DNA binding;nucleic acid binding;nucle	cell part;cytoplasm;intracellular membrane-bou	Spliceosome
Histone H3.1	Hist1h3a	biological regulation;cellular component assem	binding;DNA binding;nucleic acid binding	cell part;chromosomal part;intracellular organe	Systemic lupus erythematosus
Arylsulfatase A	Arsa	binding of sperm to zona pellucida;cell recognit	binding;calcium ion binding;catalytic activity;ca	cell part;cytoplasmic part;integral to membrane	Lysosome;Sphingolipid metabolism
DNA replication licensi	Mcm2	cell cycle;cellular component assembly;cellular	adenyl nucleotide binding;adenyl ribonucleotid	cell part;chromatin;chromosomal part;intracell	Cell cycle;Cell cycle - yeast;DNA replication;Mei
Transcobalamin-2	Tcn2	cation transport;cellular metabolic process;cell	binding;cation binding;cobalamin binding;ion b	extracellular region	Vitamin digestion and absorption
Nucleolar protein 58;N	Nop58	cellular component biogenesis;cellular compon	binding;nucleic acid binding;ribonucleoprotein	Cajal body;cell part;cytoplasm;intracellular non	Ribosome biogenesis in eukaryotes
Heterogeneous nuclea	Hnrnpul2		binding;nucleic acid binding	cell part;intracellular membrane-bounded orga	
Cathepsin B	Ctsb	biological regulation;cellular process;cellular re	catalytic activity;cysteine-type endopeptidase a	cell part;cytoplasmic membrane-bounded vesic	Antigen processing and presentation;Lysosome
Enhancer of rudimenta	Erh	cell cycle;cellular process			
Histone H1.4	Hist1h1e	cellular component assembly;cellular compone	binding;DNA binding;nucleic acid binding	cell part;chromosomal part;intracellular memb	
Histone H2B type 1-C/E	Hist1h2bc;Hist	cellular component assembly;cellular compone	binding;DNA binding;nucleic acid binding	cell part;chromosomal part;intracellular memb	Systemic lupus erythematosus
Histone H2A;Histone H	H2afj	cellular component assembly;cellular compone	binding;DNA binding;nucleic acid binding	cell part;chromosomal part;intracellular memb	Systemic lupus erythematosus
Heterogeneous nuclea	Hnrnpa2b1	biological regulation;cellular macromolecule m	binding;DNA binding;nucleic acid binding;nucle	catalytic step 2 spliceosome;cell part;cytoplasm	
C-type mannose recept	Mrc2	cellular component organization;cellular compo	binding;carbohydrate binding;collagen binding;	cell part;cell surface;integral to membrane;intr	ko05152;Phagosome
Matrix Gla protein	Mgp	anatomical structure development;biological re	binding;calcium ion binding;cation binding;ion	extracellular matrix;extracellular region;extrace	
Histone H4	Hist1h4a	biological regulation;cellular component assem	binding;DNA binding;nucleic acid binding	actin cytoskeleton;cell part;chromosomal part;	Systemic lupus erythematosus
Histone H2A.V;Histone	H2afv;H2afz	cellular component assembly;cellular compone	binding;DNA binding;nucleic acid binding	cell part;chromosomal part;intracellular memb	Systemic lupus erythematosus
Histone H1.2	Hist1h1c	cellular component assembly;cellular compone	binding;DNA binding;nucleic acid binding	cell part;chromosomal part;intracellular memb	
Syndecan-4	Sdc4	anatomical structure development;biological re	molecular transducer activity;receptor activity;	adherens junction;anchoring junction;cell junct	Cell adhesion molecules (CAMs);ECM-receptor
Splicing factor, proline-	Sfpq	alternative nuclear mRNA splicing, via spliceoso	binding;DNA binding;nucleic acid binding;nucle	cell part;cytoplasm;intracellular organelle part;	
Fractalkine	Cx3cl1	anatomical structure formation involved in mo	binding;chemokine activity;chemokine recepto	cell part;cell surface;extracellular region part;e	Chemokine signaling pathway;Cytokine-cytokin
Protein SET (Fragment)	Set	biological regulation;cellular component assem	binding;DNA binding;nucleic acid binding	cell part;cytoplasmic part;cytosol;endoplasmic	
Gamma-glutamyl hydro	Ggh	amine metabolic process;carboxylic acid metab	catalytic activity;exopeptidase activity;gamma-	cell part;cytoplasmic membrane-bounded vesic	Folate biosynthesis
Hypermethylated in ca	Hic2	biological regulation;biosynthetic process;cellu	binding;cation binding;DNA binding;ion binding	adherens junction;anchoring junction;cell junct	
Spliceosome RNA helic	Ddx39b	cellular component assembly;cellular compone	adenyl nucleotide binding;adenyl ribonucleotid	cell part;cytoplasm;intracellular organelle part;	mRNA surveillance pathway;RNA transport;Spli
High mobility group pr	Hmga2	adipose tissue development;adrenal gland deve	5'-deoxyribose-5-phosphate lyase activity;AT D	cell part;chromatin;chromosomal part;chromos	
Serine/arginine-rich sp	Srsf3	biological regulation;cell surface receptor linke	binding;nucleic acid binding;nucleotide binding	cell part;intracellular organelle part;intracellula	Spliceosome
Protein RCC2	Rcc2	cell cycle phase;cell cycle process;cell division;c		cell part;chromosomal part;chromosome, centr	
Glutathione peroxidase	Gpx4	cellular component organization;cellular compo	antioxidant activity;catalytic activity;glutathion	cell part;cytoplasmic part;cytosol;envelope;intr	Glutathione metabolism
Pentraxin-related prote	Ptx3	biological regulation;immune effector process;	1,3-beta-D-glucan binding;binding;carbohydrat	extracellular region	
Splicing factor 3B subu	Sf3b1	anterior/posterior pattern specification;cellular	binding;chromatin binding	catalytic step 2 spliceosome;cell part;intracellu	Spliceosome
Carboxypeptidase;Lyso	Ctsa	macromolecule metabolic process;metabolic p	carboxypeptidase activity;catalytic activity;exo	cell part;cytoplasmic part;intracellular membra	Lysosome;Renin-angiotensin system
Serine/arginine-rich-sp	Srsf1	biological regulation;cardiac muscle contractio	binding;nucleic acid binding;nucleotide binding	catalytic step 2 spliceosome;cell part;cytoplasm	Spliceosome
Vesicle transport prote	Golt1b	biological regulation;cellular process;establishn	molecular transducer activity;signal transducer	cell part;cytoplasmic part;endoplasmic reticulu	
Angiotensin-converting	Ace	aging;anatomical structure development;anato	binding;carboxypeptidase activity;catalytic acti	cell part;extracellular region;integral to membr	Chagas disease (American trypanosomiasis);Hy
Calsyntenin-1	Clstn1	biological adhesion;biological regulation;calciu	binding;calcium ion binding;cation binding;ion	cell junction;cell part;cell projection;cytoplasmi	
Carboxypeptidase Q	Cpq	alcohol metabolic process;amine metabolic pro	binding;carboxypeptidase activity;catalytic acti	cell part;cytoplasmic part;endoplasmic reticulu	
Hepatoma-derived gro	Hdgf	biological regulation;biosynthetic process;cellu	binding;carbohydrate binding;DNA binding;gly	cell part;cytoplasm;extracellular region part;ex	
RNA-binding protein 39	Rbm39	biological regulation;biosynthetic process;cellu	binding;nucleic acid binding;nucleotide binding	cell part;centrosome;cytoplasmic part;cytoskel	
Zinc transporter 7	Slc30a7	biological regulation;cation transport;divalent i	binding;cation binding;cation transmembrane t	cell part;cytoplasmic membrane-bounded vesic	
DEAD (Asp-Glu-Ala-Asp	Ddx5	biological regulation;biosynthetic process;cellu	adenyl nucleotide binding;adenyl ribonucleotid	catalytic step 2 spliceosome;cell part;intracellu	Spliceosome
Protein disulfide-isome	Pdia6	biological adhesion;biological regulation;cell ad	catalytic activity;disulfide oxidoreductase activi	cell part;cytoplasmic membrane-bounded vesic	Protein processing in endoplasmic reticulum
Heterogeneous nuclea	Hnrnpc	cellular macromolecule metabolic process;cellu	binding;mRNA binding;nucleic acid binding;nuc	catalytic step 2 spliceosome;cell part;intracellu	Spliceosome
Parathymosin	Ptms				
Histone H1.0	H1f0	cellular component assembly;cellular compone	binding;DNA binding;nucleic acid binding	cell part;chromatin;chromosomal part;cytoplas	
Phosphoinositide 3-kin	Pik3r4		adenyl nucleotide binding;adenyl ribonucleotid	cell part;cytoplasmic part;endosome;intracellu	Regulation of autophagy
Heterogeneous nuclea	Hnrnpm	alternative nuclear mRNA splicing, via spliceoso	binding;nucleic acid binding;nucleotide binding	catalytic step 2 spliceosome;cell part;intracellu	Spliceosome
Heterogeneous nuclea	Hnrnpa0	3'-UTR-mediated mRNA stabilization;biological	AU-rich element binding;binding;nucleic acid bi	cell part;intracellular membrane-bounded orga	
Acidic leucine-rich nucl	Anp32e		enzyme inhibitor activity;enzyme regulator acti	cell part;cytoplasmic membrane-bounded vesic	
Heterogeneous nuclea	Hnrnpu	biological regulation;cellular macromolecule m	adenyl nucleotide binding;adenyl ribonucleotid	catalytic step 2 spliceosome;cell part;cell surfac	Spliceosome
WNT1 inducible signali	Wisp2	biological adhesion;biological regulation;cell ad	binding;growth factor activity;protein binding;r	cell part;extracellular region;extracellular regio	
Small ubiquitin-related	Sumo3;Sumo2	biological regulation;cellular localization;cellula	acid-amino acid ligase activity;binding;catalytic	cell part;cytoplasm;intracellular organelle part;	RNA transport
Acidic leucine-rich nucl	Anp32b	anatomical structure development;developmen		cell part;intracellular membrane-bounded orga	
DNA replication licensi	Mcm3	cell cycle;cellular macromolecule metabolic pro	adenyl nucleotide binding;adenyl ribonucleotid	cell part;centrosome;chromosomal part;cytopla	Cell cycle;Cell cycle - yeast;DNA replication;Mei
DNA replication licensi	Mcm7	biological regulation;cell cycle;cell proliferation	adenyl nucleotide binding;adenyl ribonucleotid	cell part;chromosomal part;cytoplasmic part;cy	Cell cycle;Cell cycle - yeast;DNA replication;Mei
Cystatin	Cst6	anatomical structure development;developmen	cysteine-type endopeptidase inhibitor activity;e	cell part;cornified envelope;cytoskeleton;intrac	
RNA-binding protein EV	Ewsr1	biological regulation;biosynthetic process;cellu	binding;cation binding;ion binding;metal ion bi	cell part;cytoplasm;intracellular membrane-bou	
Beta-hexosaminidase s	Hexb	amine metabolic process;aminoglycan metabol	beta-N-acetylhexosaminidase activity;binding;c	acrosomal vesicle;cell part;cytoplasmic membr	Amino sugar and nucleotide sugar metabolism;
Non-POU domain-cont	Nono	biological regulation;biosynthetic process;cellu	binding;DNA binding;nucleic acid binding;nucle	cell part;intracellular non-membrane-bounded	
Ceruloplasmin	Cp	biological regulation;cation homeostasis;cation	binding;catalytic activity;cation binding;copper	extracellular region part;extracellular space	Porphyrin and chlorophyll metabolism
Small nuclear ribonucle	Snrpd1	cellular macromolecule metabolic process;cellu		catalytic step 2 spliceosome;cell part;cytoplasm	Spliceosome;Systemic lupus erythematosus
Laminin subunit gamm	Lamc1	anatomical structure morphogenesis;biological	binding;extracellular matrix structural constitue	extracellular matrix part;extracellular membran	Amoebiasis;ECM-receptor interaction;Focal adh
WASH complex subunit	Washc3			cell part;cytoplasmic part;intracellular part;mac	
Nucleobindin-1;Nucleo	Nucb1		binding;calcium ion binding;cation binding;DNA	cell part;cytoplasmic part;cytoskeleton;endopla	
Proliferating cell nuclea	Pcna-ps2;Pcna	base-excision repair, gap-filling;biological regul	binding;DNA binding;DNA polymerase processi	cell part;chromosomal part;cyclin-dependent p	Base excision repair;Cell cycle;DNA replication;
MCG21719;Ragulator c	Lamtor5	biological regulation;cellular component organ	enzyme regulator activity;GTPase regulator act	cell part;cytoplasmic part;cytosol;endosomal pa	
rRNA 2-O-methyltransf	Fbl	cellular macromolecule metabolic process;cellu	binding;catalytic activity;methyltransferase act	Cajal body;cell part;dense fibrillar component;g	Ribosome biogenesis in eukaryotes

Beta-glucuronidase	Gusb	carbohydrate metabolic process;metabolic pro	beta-glucuronidase activity;catalytic activity;hy	cell part;cytoplasmic part;endoplasmic reticulu	Drug metabolism - other enzymes;Flavone and
Collagen alpha-1(V) ch	Col5a1	anatomical structure development;anatomical	binding;carbohydrate binding;extracellular mat	basement membrane;collagen;collagen type V;	Amoebiasis;ECM-receptor interaction;Focal adh
Heterogeneous nuclea	Hnrnpd	biological regulation;biosynthetic process;cellu	binding;DNA binding;nucleic acid binding;nucle	cell part;cytoplasm;intracellular membrane-bou	
Transaldolase	Taldo1	alcohol metabolic process;carbohydrate metab	binding;carbohydrate binding;catalytic activity;	cell part;cytoplasmic part;cytosol;intracellular r	Pentose phosphate pathway
Small nuclear ribonucle	Snrpd2	cellular macromolecule metabolic process;cellu		catalytic step 2 spliceosome;cell part;intracellu	Spliceosome
Protein canopy homolo	Cnpy3	defense response;immune response;immune s		cell part;cytoplasmic part;endoplasmic reticulu	
Nucleobindin-2	Nucb2	biological regulation;calcium ion homeostasis;c	binding;calcium ion binding;cation binding;DNA	cell part;cytoplasmic part;endoplasmic reticulu	
Ragulator complex pro	Lamtor2	activation of MAPKK activity;activation of prote	enzyme regulator activity;GTPase regulator act	cell part;cytoplasmic part;endosomal part;endo	
ATP-dependent RNA he	Ddx39a;Ddx39	cellular macromolecule metabolic process;cellu	adenyl nucleotide binding;adenyl ribonucleotid	cell part;cytoplasm;intracellular membrane-bou	
Semaphorin-3A (Fragm	Sema3a	ameboidal cell migration;anatomical structure	chemorepellent activity;receptor activity	axon;cell part;cell projection;dendrite;extracell	Axon guidance
Twisted gastrulation pr	Twsg1	anatomical structure development;anatomical		extracellular region part;extracellular space	
Heterogeneous nuclea	Hnrnph1	biological regulation;cellular macromolecule m	binding;nucleic acid binding;nucleotide binding	actin cytoskeleton;catalytic step 2 spliceosome	
Heterogeneous nuclea	Hnrnpab	anatomical structure morphogenesis;biological	binding;DNA binding;nucleic acid binding;nucle	cell part;cytoplasm;intracellular membrane-bou	
V-type proton ATPase s	Atp6v1c1	ATP hydrolysis coupled proton transport;cation	active transmembrane transporter activity;ATP	apical part of cell;cell part;cytoplasmic part;cyt	Collecting duct acid secretion;Epithelial cell sig
Podoplanin	Pdpn	anatomical structure development;anatomical		apical plasma membrane;cell part;cell projectic	
Lysosomal alpha-gluco	Gaa	anatomical structure development;anatomical	alpha-glucosidase activity;binding;carbohydrat	cell part;cytoplasmic part;intracellular membra	Galactose metabolism;Lysosome;Starch and su
Phospholipid scrambla	Plscr2	cellular process;cellular response to biotic stim	binding;cation binding;ion binding;metal ion bi	cell part;cytoplasm;integral to membrane;intra	
N(4)-(beta-N-acetylglu	Aga	cellular macromolecule metabolic process;cellu	asparaginase activity;catalytic activity;hydrolas	cell part;cytoplasmic part;endoplasmic reticulu	Lysosome;Other glycan degradation
Myb-binding protein 1	Mybbp1a	biological regulation;biosynthetic process;cell c	binding;catalytic activity;cation binding;DNA bi	cell part;cytoplasm;intracellular non-membran	
Choline transporter-lik	Slc44a1		active transmembrane transporter activity;ami	cell part;cytoplasmic part;integral to membran	
RNA-binding protein FU	Fus		binding;cation binding;DNA binding;ion binding	cell part;cytoplasm;intracellular membrane-bou	
MCG8382, isoform CRA	Gm21992;Rbn	biological regulation;biosynthetic process;cellu	binding;cation binding;ion binding;ligand-depe	cell part;intracellular non-membrane-bounded	
Cbx3 protein;Chromob	Cbx3	biological regulation;biosynthetic process;cellu	binding;chromatin binding;histone binding;met	cell part;centromeric heterochromatin;chroma	
Protein ERGIC-53	Lman1	biological regulation;cellular component organ	binding;cation binding;ion binding;metal ion bi	cell part;contractile fiber part;cytoplasmic part	Protein processing in endoplasmic reticulum
Epididymal secretory p	Npc2	alcohol metabolic process;biological regulation	binding;cholesterol binding;lipid binding;steroi	cell part;cytoplasmic part;endoplasmic reticulu	Lysosome
Small nuclear ribonucle	Snrpe	cellular macromolecule metabolic process;cellu	binding;nucleic acid binding;RNA binding	catalytic step 2 spliceosome;cell part;intracellu	Spliceosome
Heterogeneous nuclea	Hnrnpf	biological regulation;cellular macromolecule m	binding;nucleic acid binding;nucleotide binding	cell part;intracellular organelle part;intracellula	
SPARC	Sparc	anatomical structure development;biological re	binding;calcium ion binding;cation binding;extr	basement membrane;cell part;cytoplasm;extra	
Mammalian ependymi	Epdr1	biological adhesion;cell adhesion;cell-matrix ad	binding;calcium ion binding;cation binding;ion	cell part;cytoplasmic part;extracellular region;i	
Procollagen C-endopep	Pcolce	macromolecule metabolic process;metabolic p	binding;carbohydrate binding;collagen binding;	extracellular matrix;extracellular membrane-bo	
Lupus La protein homo	Ssb	cellular macromolecule metabolic process;cellu	binding;nucleic acid binding;nucleotide binding	cell part;intracellular membrane-bounded orga	Systemic lupus erythematosus
Prelamin-A/C	Lmna	anatomical structure development;biological re	structural molecule activity	cell part;cytoplasmic part;cytoskeletal part;env	Arrhythmogenic right ventricular cardiomyopat
Cathepsin D;Uncharact	Ctsd	autophagic vacuole assembly;cellular compone	aspartic-type endopeptidase activity;aspartic-ty	cell part;cytoplasmic membrane-bounded vesic	ko05152;Lysosome
Endothelial cell-specifi	Esm1	anatomical structure formation involved in mo		extracellular region	
Insulin-like growth fact	Igfbp4	biological regulation;cell proliferation;defense		extracellular region part;extracellular space	
Predicted pseudogene	Gm9242;Gm6	cellular macromolecule metabolic process;cellu	binding;nucleic acid binding;nucleotide binding	catalytic step 2 spliceosome;cell part;intracellu	Spliceosome
Alpha-N-acetylglucosar	Naglu	anatomical structure development;anatomical			Glycosaminoglycan degradation;Lysosome
Mannosyl-oligosacchar	Mogs	carbohydrate metabolic process;metabolic pro	catalytic activity;glucosidase activity;hydrolase	cell part;cytoplasmic part;endoplasmic reticulu	N-Glycan biosynthesis;Protein processing in en
Beta-hexosaminidase s	Hexa	adult behavior;adult locomotory behavior;adul	beta-N-acetylhexosaminidase activity;catalytic	cell part;cytoplasmic part;intracellular membra	Amino sugar and nucleotide sugar metabolism;
Tubulin gamma-1 chair	Tubg1;Tubg2	biological regulation;cell cycle process;cellular	binding;catalytic activity;GTP binding;GTPase a	apical part of cell;cell leading edge;cell part;cel	
Peptidyl-prolyl cis-tran	Ppib	cellular macromolecule metabolic process;cellu	binding;catalytic activity;cis-trans isomerase ac	cell part;cytoplasmic membrane-bounded vesic	
Adipocyte enhancer-bi	Aebp1	biological adhesion;biological regulation;biosyr	binding;carboxypeptidase activity;catalytic acti	cell part;cytoplasm;extracellular matrix;extrace	
Nucleophosmin	Npm1	aging;biological regulation;cell aging;cell cycle	binding;DNA binding;enzyme inhibitor activity;	cell part;centrosome;cytoplasmic part;cytoske	
Beta-galactosidase	Glb1	alcohol catabolic process;alcohol metabolic pro	beta-galactosidase activity;binding;carbohydra	cell part;cytoplasmic part;intracellular membra	Galactose metabolism;Glycosaminoglycan degr
Splicing factor 3B subu	Sf3b3	cellular macromolecule metabolic process;cellu	binding;nucleic acid binding	catalytic step 2 spliceosome;cell part;intracellu	Spliceosome
ATP-dependent RNA he	Dhx9	biological regulation;cellular macromolecule m	adenyl nucleotide binding;adenyl ribonucleotid	cell part;centrosome;CRD-mediated mRNA stab	
Heterogeneous nuclea	Hnrnpl	cellular macromolecule metabolic process;cellu	binding;DNA binding;nucleic acid binding;nucle	cell part;cytoplasm;intracellular membrane-bou	
Arylsulfatase B	Arsb	anatomical structure development;autophagy;	arylsulfatase activity;binding;catalytic activity;c	cell part;cytoplasmic part;endoplasmic reticulu	Glycosaminoglycan degradation;Lysosome
Transmembrane protei	Tmem206			cell part;integral to membrane;intrinsic to mem	
N-acetylglucosamine-6	Gns	amine metabolic process;aminoglycan metabol	binding;catalytic activity;cation binding;hydrola	cell part;cytoplasmic part;intracellular membra	Glycosaminoglycan degradation;Lysosome
Transitional endoplasm	Vcp	activation of caspase activity;aggresome assem	adenyl nucleotide binding;adenyl ribonucleotid	cell part;chromosomal part;cytoplasmic part;cy	Protein processing in endoplasmic reticulum
MCG134445, isoform C	Mroh1				
Early endosome antige	Eea1	cellular component organization;cellular comp	1-phosphatidylinositol binding;binding;cation b	cell part;cytoplasmic part;cytosol;early endoso	Endocytosis;ko05152;Phagosome
TAR DNA-binding prote	Tardbp	3'-UTR-mediated mRNA stabilization;biological	binding;DNA binding;double-stranded DNA bin	cell part;intracellular membrane-bounded orga	
ADP-ribosylation factor	Arl8a	biological regulation;cell cycle phase;cell cycle	binding;GTP binding;guanyl nucleotide binding;	cell part;cytoplasmic part;cytoskeletal part;end	
Small nuclear ribonucle	Snrpb;Snrpn	cellular macromolecule metabolic process;cellu	binding;histone pre-mRNA DCP binding;nucleic	catalytic step 2 spliceosome;cell part;cytoplasm	Spliceosome;Systemic lupus erythematosus
Histone-binding protei	Rbbp4	biological regulation;biosynthetic process;cell c	ATPase activity;ATPase activity, coupled;cataly	CAF-1 complex;cell part;chromatin assembly co	
NADH-cytochrome b5 r	Cyb5r1	alcohol metabolic process;biosynthetic process	catalytic activity;cytochrome-b5 reductase acti	cell part;cytoplasmic part;integral to membran	Amino sugar and nucleotide sugar metabolism
Lysosomal acid phosph	Acp2	anatomical structure development;cellular com	acid phosphatase activity;catalytic activity;hydr	cell part;cytoplasmic part;integral to membran	Riboflavin metabolism
Oligosaccharyltransfera	Ostc			cell part;integral to membrane;intrinsic to mem	
Protein transport prote	Sec23a	cellular process;ER to Golgi vesicle-mediated tr	binding;cation binding;ion binding;metal ion bi	cell part;COPII vesicle coat;cytoplasmic part;cyt	Protein processing in endoplasmic reticulum
Transmembrane emp2	Tmed10	amyloid precursor protein catabolic process;am		cell part;cis-Golgi network;clathrin-coated vesic	
Glucosylceramidase	Gba	alcohol biosynthetic process;alcohol metabolic	catalytic activity;ceramidase activity;glucosylce	cell part;cytoplasmic part;intracellular organell	Lysosome;Other glycan degradation;Sphingolip
Small nuclear ribonucle	Snrpd3	cellular macromolecule metabolic process;cellu	binding;histone pre-mRNA DCP binding;nucleic	catalytic step 2 spliceosome;cell part;cytoplasm	Spliceosome;Systemic lupus erythematosus
Ragulator complex pro	Lamtor1	biological regulation;cell growth;cellular compo	enzyme regulator activity;GTPase regulator act	cell part;cytoplasmic part;endosomal part;Golg	
D-dopachrome decarbo	Ddt;Gm20441	biosynthetic process;cellular biosynthetic proce	carbon-carbon lyase activity;carboxy-lyase activ	cell part;cytoplasm;intracellular part	
Protein LYRIC	Mtdh	biological regulation;cell junction assembly;cell	binding;NF-kappaB binding;protein binding;pro	apical plasma membrane;cell junction;cell part;	
Laminin subunit beta-1	Lamb1	anatomical structure morphogenesis;behavior;	binding;extracellular matrix structural constitue	cell part;cytoplasmic part;extracellular matrix p	Amoebiasis;ECM-receptor interaction;Focal adh
Heterogeneous nuclea	Hnrnpk	biological regulation;cellular macromolecule m	binding;core promoter proximal region DNA bi	catalytic step 2 spliceosome;cell junction;cell p	Spliceosome
Kinesin-like protein KIF	Kif1a	anterograde axon cargo transport;axon cargo t	adenyl nucleotide binding;adenyl ribonucleotid	cell body;cell part;cell projection;cytoplasm;cyt	
Transmembrane emp2	Tmed5	cellular component organization;cellular comp		cell part;cis-Golgi network;cytoplasmic part;enc	
Erythrocyte band 7 inte	Stom	cellular component assembly;cellular compone		cell part;cytoplasmic membrane-bounded vesic	
Putative phospholipase	Plbd2	catabolic process;lipid catabolic process;lipid m	catalytic activity;hydrolase activity	cell part;cytoplasmic part;intracellular membra	
Fibulin-2	Fbln2	biological regulation;positive regulation of biol	binding;calcium ion binding;cation binding;extr	extracellular matrix;extracellular region part;pr	
Transmembrane emp2	Tmed9	biological regulation;cellular component organi		cell part;cytoplasmic part;endoplasmic reticulu	
Amyloid-beta A4 prote	App	adult behavior;adult locomotory behavior;alco	binding;carbohydrate binding;cation binding;D	apical part of cell;axon;cell part;cell projection;	Alzheimer's disease
Platelet-activating fact	Pla2g7	biological regulation;catabolic process;cellular	1-alkyl-2-acetylglycerophosphocholine esterase	extracellular region part;low-density lipoprotei	Ether lipid metabolism
Fibronectin	Fn1	biological adhesion;calcium-independent cell-m	enzyme activator activity;enzyme regulator act	apical plasma membrane;basement membrane	Amoebiasis;Bacterial invasion of epithelial cells
Transmembrane protei	Tmem33			cell part;cytoplasmic membrane-bounded vesic	
Beta-2-syntrophin	Sntb2		binding;lipid binding;phospholipid binding	cell junction;cell part;cytoplasmic part;cytoplas	
Thioredoxin domain-co	Txndc12	biological regulation;cell redox homeostasis;ce	catalytic activity;oxidoreductase activity;oxidor	cell part;cytoplasmic part;endoplasmic reticulu	Glutathione metabolism
Collagen, type VI, alpha	Col6a3		endopeptidase inhibitor activity;endopeptidase	cell part;extracellular matrix;extracellular regio	ECM-receptor interaction;Focal adhesion;Prote
EGF-containing fibulin-	Efemp2	biological regulation;blood coagulation;coagula	binding;calcium ion binding;cation binding;ion	cell part;extracellular matrix;extracellular regio	
Protein transport prote	Sec61b;Gm10	catabolic process;cellular catabolic process;cell	binding;ribonucleoprotein binding;ribosome bi	cell part;cytoplasmic part;endoplasmic reticulu	Phagosome;Protein export;Protein processing i
Prosaposin	Psap	biological regulation;cell differentiation;cellular		cell part;cytoplasmic part;extracellular region p	Lysosome
Polyadenylate-binding	Pabpc4;Pab		binding;nucleic acid binding;nucleotide binding		mRNA surveillance pathway;RNA degradation;F
Nucleolin	Ncl	anatomical structure formation involved in mo	binding;DNA binding;nucleic acid binding;nucle	cell cortex;cell part;cytoplasmic part;intracellul	Pathogenic Escherichia coli infection
Tubulointerstitial neph	Tinagl1	immune response;immune system process;ma	binding;carbohydrate binding;cargo receptor a	cell part;cytoplasm;extracellular matrix;extrace	
Basement membrane-s	Hspg2	anatomical structure development;anatomical		basal lamina;extracellular matrix part;extracell	ECM-receptor interaction
Lon protease homolog,	Lonp1	aging;biological regulation;catabolic process;ce	adenyl nucleotide binding;adenyl ribonucleotid	cell part;cytoplasmic part;intracellular non-me	
Protein disulfide-isome	Pdia4	biological regulation;cell redox homeostasis;ce	catalytic activity;disulfide oxidoreductase activi	cell part;cell surface;cytoplasmic membrane-bo	Protein processing in endoplasmic reticulum;Vi
Syntaxin-binding prote	Stxbp3	anatomical structure development;biological re	binding;protein binding;SNARE binding;syntaxi	apical plasma membrane;cell part;cytoplasmic	
Calumenin;Calumenin (	Calu		binding;calcium ion binding;cation binding;ion	cell part;cytoplasmic membrane-bounded vesic	
ERO1-like protein alpha	Ero1a	apoptosis;apoptosis in response to endoplasmi	binding;catalytic activity;coenzyme binding;cof	cell part;cytoplasmic part;endoplasmic reticulu	Protein processing in endoplasmic reticulum;Vi
Granulins;Granulins (Fr	Grn	biological regulation;blastocyst hatching;develo		cell part;cytoplasmic part;extracellular region p	
WASH complex subunit	Washc2	cellular process;endosome transport;establishn		cell part;cytoplasmic part;early endosome;early	
Annexin A11;Annexin	Anxa11	cellular component organization;cellular comp	binding;calcium ion binding;calcium-dependent	azurophil granule;cell part;cytoplasm;cytoplasn	
Glypican-1	Gpc1	anatomical structure formation involved in mo	binding;cation binding;copper ion binding;extra	anchored to membrane;cell part;cytoplasmic p	
78 kDa glucose-regulat	Hspa5	activation of signaling protein activity involved	adenyl nucleotide binding;adenyl ribonucleotid	cell part;cell surface;cytoplasmic membrane-bo	Prion diseases;Protein export;Protein processin
Annexin	Anxa6	biological regulation;calcium ion transport;cati	binding;calcium ion binding;calcium-dependent	cell part;cytoplasmic part;intracellular part;per	
Endoplasmin;Endoplas	Hsp90b1	actin cytoskeleton organization;actin filament c	adenyl nucleotide binding;adenyl ribonucleotid	cell part;cytoplasmic membrane-bounded vesic	NOD-like receptor signaling pathway;Pathways
Galectin-3-binding prot	Lgals3bp	biological adhesion;cell adhesion;cellular proce	cargo receptor activity;receptor activity;scaven	cell part;extracellular matrix;extracellular mem	
Annexin A2;Annexin (F	Anxa2	anatomical structure formation involved in mo	binding;calcium ion binding;calcium-dependent	basement membrane;cell part;cytoplasmic mer	
Asparagine--tRNA ligas	Nars	amine metabolic process;amino acid activation	adenyl nucleotide binding;adenyl ribonucleotid	cell part;cytoplasmic part;intracellular membra	Aminoacyl-tRNA biosynthesis
Phosphoglycerate mut	Pgam1	alcohol biosynthetic process;alcohol catabolic p	2,3-bisphosphoglycerate-dependent phosphog	cell part;cytoplasmic part;cytosol;intracellular r	Glycolysis / Gluconeogenesis;Methane metabo

Calpain small subunit 1	Capns1		binding;calcium ion binding;calcium-dependen	cell part;cytoplasm;intracellular part;membran	
Cytoplasmic dynein 1 h	Dync1h1	ATP catabolic process;ATP metabolic process;c	adenyl nucleotide binding;adenyl ribonucleotid	cell part;centrosome;cytoplasmic part;cytoskel	Phagosome;Vasopressin-regulated water reabs
40S ribosomal protein	Rps6	biological regulation;biosynthetic process;carb	structural constituent of ribosome;structural m	cell part;intracellular non-membrane-bounded	Insulin signaling pathway;mTOR signaling pathw
Ran-specific GTPase-ac	Ranbp1	biological regulation;cellular component organ	enzyme activator activity;enzyme regulator act	cell part;centrosome;cytoplasm;cytoplasmic pa	
Phosphoserine aminot	Psat1	amine biosynthetic process;amine metabolic p	binding;catalytic activity;cofactor binding;O-ph		Glycine, serine and threonine metabolism;Meth
Bifunctional glutamate	Eprs	amine metabolic process;amino acid activation	adenyl nucleotide binding;adenyl ribonucleotid	cell part;cytoplasm;intracellular part;macromol	Aminoacyl-tRNA biosynthesis;Porphyrin and ch
Adenylyl cyclase-assoc	Cap1	actin cytoskeleton organization;actin filament-		cell cortex part;cell part;cortical actin cytoskele	
Hypoxanthine-guanine	Hprt1	adenine biosynthetic process;adenine metabol	binding;catalytic activity;cation binding;guanin	cell part;cytoplasmic part;cytosol;intracellular p	Drug metabolism - other enzymes;Purine meta
Elongation factor 1-alp	Eef1a1	biological regulation;catabolic process;cellular	binding;catalytic activity;GTP binding;GTPase a	cell body;cell part;cytoplasm;cytoplasmic part;	RNA transport
D-3-phosphoglycerate	Phgdh	amine biosynthetic process;amine metabolic p	binding;catalytic activity;coenzyme binding;cof		Glycine, serine and threonine metabolism;Meth
Very-long-chain 3-oxoa	Hsd17b12	biological regulation;biosynthetic process;carb	binding;carbohydrate binding;catalytic activity;	cell part;cytoplasmic part;endoplasmic reticulu	Biosynthesis of unsaturated fatty acids;Steroid
Lysine--tRNA ligase	Kars	amine metabolic process;amino acid activation	adenyl nucleotide binding;adenyl ribonucleotid	aminoacyl-tRNA synthetase multienzyme comp	Aminoacyl-tRNA biosynthesis
Obg-like ATPase 1	Ola1	ATP catabolic process;ATP metabolic process;c	adenyl nucleotide binding;adenyl ribonucleotid	cell part;cytoplasm;intracellular non-membran	
Cofilin-1	Cfl1	actin cytoskeleton organization;actin filament		cell cortex part;cell part;cell projection;cell pro	Axon guidance;Fc gamma R-mediated phagocyt
GTP-binding protein SA	Sar1a	cellular process;establishment of localization;e	binding;GTP binding;guanyl nucleotide binding;	cell part;cytoplasmic part;endoplasmic reticulu	Protein processing in endoplasmic reticulum
Glycine--tRNA ligase	Gars	amine metabolic process;amino acid activation	adenyl nucleotide binding;adenyl ribonucleotid	cell part;cytoplasmic membrane-bounded vesic	Aminoacyl-tRNA biosynthesis
Cystatin-B	Cstb	adult behavior;adult locomotory behavior;beha	cysteine-type endopeptidase inhibitor activity;	cell part;cytoplasm;intracellular non-membran	
Triosephosphate isome	Tpi1	alcohol biosynthetic process;alcohol catabolic	catalytic activity;intramolecular oxidoreductase		Carbon fixation in photosynthetic organisms;Fr
Kinesin-like protein KIF	Kif23	cell cycle process;cellular component assembly	adenyl nucleotide binding;adenyl ribonucleotid	cell part;centralspindlin complex;centrosome;c	
60S ribosomal protein	Rpl22	alpha-beta T cell activation;alpha-beta T cell dif	binding;carbohydrate binding;glycosaminoglyca	cell part;cytoplasmic part;cytosolic large riboso	Ribosome
Receptor of activated p	Rack1	anatomical structure morphogenesis;biological	apoptotic protease activator activity;binding;ca	cell body;cell part;cell projection;cytoplasmic p	Measles
Dynein light chain 1, cy	Dynll1	apoptosis;biological regulation;biosynthetic pro	catalytic activity;enzyme regulator activity;hyd	cell part;centrosome;chromosomal part;cytopla	Vasopressin-regulated water reabsorption
Proliferation-associate	Pa2g4	biological regulation;biosynthetic process;cellu	binding;DNA binding;nucleic acid binding;nucle	cell part;cytoplasmic part;intracellular membrane-bou	
Protein S100-A6	S100a6		binding;calcium ion binding;cation binding;ion	cell part;cell projection;cytoplasmic part;cytoso	
60S ribosomal protein	Rpl36a;Gm652	biosynthetic process;cellular biosynthetic proce	structural constituent of ribosome;structural m	cell part;cytoplasmic part;intracellular non-mem	Ribosome
Glucose-6-phosphate 1	G6pdx	alcohol biosynthetic process;alcohol catabolic p	binding;catalytic activity;coenzyme binding;cof		Glutathione metabolism;Pentose phosphate pa
Kinesin-1 heavy chain;K	Kif5b	biological regulation;cellular chemical homeost	adenyl nucleotide binding;adenyl ribonucleotid	cell part;cell projection;cell projection part;cilia	
Eukaryotic translation i	Eif2s3x	catabolic process;cellular catabolic process;cell	binding;catalytic activity;GTP binding;GTPase a		RNA transport
CTP synthase 1	Ctps1	amine metabolic process;biosynthetic process;	adenyl nucleotide binding;adenyl ribonucleotid		Pyrimidine metabolism
Dynactin subunit 2	Dctn2	cell cycle phase;cell cycle process;cell proliferat	catalytic activity;hydrolase activity;hydrolase a	cell part;cell projection part;centrosome;chrom	Huntington's disease;Vasopressin-regulated wa
40S ribosomal protein	Rps20	biosynthetic process;cellular biosynthetic proce	binding;nucleic acid binding;RNA binding;struct	cell part;cytosolic small ribosomal subunit;intra	Ribosome
Peroxiredoxin-1;Peroxi	Prdx1	biological regulation;catabolic process;cell killin	antioxidant activity;binding;catalytic activity;ca	cell part;chromatin;chromosomal part;cytoplas	Peroxisome
Dynactin subunit 1	Dctn1	establishment of localization;transport	catalytic activity;hydrolase activity;hydrolase a	cell part;cell projection part;centrosome;chrom	Huntington's disease;Vasopressin-regulated wa
Destrin	Dstn	actin cytoskeleton organization;actin filament		cell cortex part;cell part;cortical actin cytoskele	
40S ribosomal protein	Rps10	cellular component assembly;cellular compone	structural constituent of ribosome;structural m	cell part;cytosolic small ribosomal subunit;intra	Ribosome
Ras-related protein Ra	Rab6a	biological regulation;cellular macromolecule m	ATPase activator activity;ATPase regulator activ	cell part;cytoplasmic part;Golgi apparatus;Golg	
Serine--tRNA ligase, cy	Sars	amine metabolic process;amino acid activation	adenyl nucleotide binding;adenyl ribonucleotid	cell part;cytoplasmic part;intracellular membra	Aminoacyl-tRNA biosynthesis
Peroxiredoxin-2;Peroxi	Prdx2	activation of MAPK activity;anatomical structur	antioxidant activity;binding;catalytic activity;ox	cell part;cytoplasmic part;intracellular membra	
60S ribosomal protein	Rpl37a	biosynthetic process;cellular biosynthetic proce	binding;cation binding;ion binding;metal ion bi	cell part;cytosolic large ribosomal subunit;intra	Ribosome
60S ribosomal protein	Rpl38	90S preribosome assembly;anatomical structur	structural constituent of ribosome;structural m	cell part;cytoplasmic part;cytosolic large riboso	Ribosome
60S ribosomal protein	Rpl31	biosynthetic process;cellular biosynthetic proce	structural constituent of ribosome;structural m	cell part;cytosolic large ribosomal subunit;intra	Ribosome
Torsin-1B;Torsin-1B (Fr	Tor1b	cellular component assembly;cellular compone	adenyl nucleotide binding;adenyl ribonucleotid	cell part;cytoplasmic part;endoplasmic reticulu	
Synaptic vesicle memb	Vat1	biological regulation;negative regulation of bio	binding;catalytic activity;cation binding;ion bin	cell part;cytoplasmic part;intracellular organell	
Thrombospondin-1	Thbs1	anatomical structure development;anatomical	binding;calcium ion binding;carbohydrate bind	cell part;cytoplasmic part;extracellular region;e	ECM-receptor interaction;Focal adhesion;Malar
Beta-enolase	Eno3	aging;alcohol catabolic process;alcohol metabo	binding;carbon-oxygen lyase activity;catalytic a	cell part;cytoplasmic part;cytosolic part;intrace	Glycolysis / Gluconeogenesis;Methane metabo
Cytosolic acyl coenzym	Acot7	carboxylic acid catabolic process;carboxylic aci	acyl-CoA thioesterase activity;carboxylesterase	cell body;cell part;cell projection;cytoplasmic p	Biosynthesis of unsaturated fatty acids
Endonuclease domain-	Endod1	cellular metabolic process;cellular nitrogen con	binding;catalytic activity;cation binding;endonu	extracellular region	Apoptosis
Ubiquitin-fold modifier	Ufm1	cellular macromolecule metabolic process;cellu		cell part;cytoplasm;intracellular membrane-bou	
Kinesin light chain 1	Klc1	axon cargo transport;cellular component disass	catalytic activity;hydrolase activity;hydrolase a	axon;cell body;cell part;cell projection;cell proj	
Elongation factor 2	Eef2	biosynthetic process;cellular biosynthetic proce	binding;catalytic activity;GTP binding;GTPase a	cell part;cytoplasm;intracellular part;macromol	
ATP-dependent RNA he	Ddx19a;Ddx19	anatomical structure development;biological re	adenyl nucleotide binding;adenyl ribonucleotid	cell part;chromatoid body;cytoplasm;cytoplasm	
40S ribosomal protein	Rps12-ps3;Rps	biosynthetic process;cellular biosynthetic proce	structural constituent of ribosome;structural m	cell part;cytoplasmic part;cytosolic small riboso	Ribosome
Ras-related protein Ra	Rab4b	biological regulation;cellular process;cellular re	binding;catalytic activity;GDP binding;GTP bind	cell part;cytoplasmic part;cytoplasmic vesicle m	Endocytosis
Alpha-centractin	Actr1a		adenyl nucleotide binding;adenyl ribonucleotid	cell part;centrosome;cytoplasm;cytoplasmic pa	
Cysteine--tRNA ligase,	Cars	amine metabolic process;amino acid activation	adenyl nucleotide binding;adenyl ribonucleotid	cell part;cytoplasmic part;cytosol;intracellular p	Aminoacyl-tRNA biosynthesis
Latexin	Lxn	defense response;detection of abiotic stimulus	binding;carbohydrate binding;endopeptidase in	cell part;cytoplasm;intracellular part	
Inorganic pyrophospha	Ppa1	cellular metabolic process;cellular process;met	binding;catalytic activity;cation binding;hydrola	cell part;cytoplasm;intracellular part	Oxidative phosphorylation
Alcohol dehydrogenase	Adh5	aging;alcohol catabolic process;alcohol metabo	alcohol dehydrogenase (NAD) activity;binding;	cell part;cytoplasmic part;intracellular membra	Chloroalkane and chloroalkene degradation;Dr
Protein FAM234B	Fam234b			cell part;integral to membrane;intrinsic to mem	
Ubiquilin-1	Ubqln1	biological regulation;cellular process;cellular re	binding;enzyme binding;intermediate filament	cell part;cytoplasmic part;endoplasmic reticulu	Protein processing in endoplasmic reticulum
Nascent polypeptide-a	Naca	anatomical structure development;anatomical	binding;DNA binding;nucleic acid binding;prote	cell part;cytoplasm;intracellular membrane-bou	
Secernin-1 (Fragment);	Scrn1	cellular process;establishment of localization;e	catalytic activity;dipeptidase activity;exopeptid	cell part;cytoplasm;intracellular organelle part;	
AHNAK nucleoprotein	Ahnak2		binding;nucleic acid binding		
GTP cyclohydrolase 1 f	Gchfr	biological regulation;cellular component assem	amine binding;amino acid binding;binding;carb	cell part;cell projection;cytoplasmic membrane	
Bicaudal D homolog 2	Bicd2	biological regulation;cellular component mover		cell part;cytoplasmic part;cytoplasmic vesicle;c	
Platelet-activating fact	Pafah1b1	acrosome assembly;actin cytoskeleton organiza	binding;cytoskeletal protein binding;microtubu	astral microtubule;cell body;cell cortex;cell par	Ether lipid metabolism
Ribonucleoside-diphos	Rrm1	biosynthetic process;cellular biosynthetic proce	adenyl nucleotide binding;adenyl ribonucleotid	cell part;cytoplasmic part;intracellular part;mac	Glutathione metabolism;Purine metabolism;Py
40S ribosomal protein	Rps28	biosynthetic process;cellular biosynthetic proce	structural constituent of ribosome;structural m	cell part;cytosolic small ribosomal subunit;intra	Ribosome
E3 ubiquitin-protein lig	Rbx1	catabolic process;cellular catabolic process;cell	acid-amino acid ligase activity;binding;catalytic	anaphase-promoting complex;cell part;Cul2-RI	Cell cycle;Cell cycle - yeast;Circadian rhythm - m
Stathmin-2	Stmn2	biological regulation;negative regulation of bio		axon;cell part;cell projection;cell projection par	
Mitochondrial import r	Tomm70			cell part;cytoplasmic part;integral to membran	
Nucleoside diphosphat	Nme1	anatomical structure development;biological re	adenyl nucleotide binding;adenyl ribonucleotid	cell part;centrosome;cytoplasmic part;cytoskel	Purine metabolism;Pyrimidine metabolism
40S ribosomal protein	Rps15	biosynthetic process;cellular biosynthetic proce	binding;nucleic acid binding;RNA binding;struct	cell part;intracellular organelle part;intracellula	Ribosome
Basic leucine zipper an	Bzw1	biological regulation;biosynthetic process;cellu		cell part;cytoplasm;intracellular part	
Discoidin domain-conta	Ddr2	anatomical structure development;biological a	adenyl nucleotide binding;adenyl ribonucleotid	cell part;integral to membrane;intrinsic to mem	
Aminoacyl tRNA syntha	Aimp1	alcohol metabolic process;anatomical structure	binding;cytokine activity;identical protein bindi	aminoacyl-tRNA synthetase multienzyme comp	
Testis-expressed gene	Tex264				
Elongator complex pro	Elp2	biological regulation;biosynthetic process;cellu	binding;DNA binding;endopeptidase activator a	cell part;cytoplasm;Elongator holoenzyme com	
Plasminogen activator	Serbp1		binding;mRNA 3'-UTR binding;mRNA binding;n	cell part;cytoplasm;cytoplasmic part;intracellul	
MCG6846, isoform CRA	Prpsap1	biological regulation;biosynthetic process;cellu	binding;catalytic activity;cation binding;diphos	macromolecular complex;protein complex;ribo	
Tumor protein p53-ind	Tp53inp2;Trp5	autophagic vacuole assembly;biological regulat		autophagic vacuole;cell part;cytoplasmic part;c	
GTP-binding protein 1	Gtpbp1	biological regulation;positive regulation of biol	binding;catalytic activity;GTP binding;GTPase a	cell part;cytoplasmic exosome (RNase complex	
Microtubule-associated	Map1b	anatomical structure development;axon extens	binding;catalytic activity;cytoskeletal protein b	cell junction;cell part;cell projection;cell project	
Atlastin-2	Atl2	catabolic process;cellular catabolic process;cell	binding;catalytic activity;GTP binding;GTPase a	cell part;cytoplasmic part;endoplasmic reticulu	
Leucine-rich repeat flig	Lrrfip1	biological regulation;biosynthetic process;cell s	binding;DNA binding;nucleic acid binding	cell part;cytoplasm;intracellular membrane-bou	
Inosine triphosphate p	Itpa	catabolic process;cellular catabolic process;cell	binding;catalytic activity;cation binding;dITP di	cell part;cytoplasm;intracellular part	Drug metabolism - other enzymes;Purine meta
Geranylgeranyl transfe	Rabggta	cellular macromolecule metabolic process;cellu	binding;catalytic activity;cation binding;ion bin		
Beta-centractin	Actr1b		adenyl nucleotide binding;adenyl ribonucleotid	cell part;centrosome;cytoplasm;cytoplasmic pa	
Protein arginine N-met	Prmt3	cellular macromolecule metabolic process;cellu	arginine N-methyltransferase activity;binding;c	cell part;cytoplasmic part;cytosol;intracellular p	
Proteasome subunit be	Psmb5	catabolic process;cellular catabolic process;cell	catalytic activity;endopeptidase activity;hydrol	cell part;cytoplasmic part;cytosol;intracellular r	Proteasome
Fasciculation and elong	Fez2				
Ras-related protein Ra	Rab1b	biological regulation;cellular process;cellular re	binding;GTP binding;guanyl nucleotide binding;	cell part;cytoplasmic part;Golgi apparatus;intra	
Metalloproteinase inhi	Timp3	biological regulation;cellular process;cellular re	binding;cation binding;endopeptidase inhibitor	basement membrane;extracellular matrix part;	
HEAT repeat-containin	Heatr5a				
Serine hydroxymethylt	Shmt1	amine biosynthetic process;amine catabolic pro	aldehyde-lyase activity;amine binding;amino ac	cell part;cytoplasmic part;cytosol;intracellular r	Cyanoamino acid metabolism;Glycine, serine ar
Methylthioribose-1-ph	Mri1	amine biosynthetic process;amine metabolic p	catalytic activity;intramolecular oxidoreductase	cell part;cytoplasm;intracellular membrane-bo	Cysteine and methionine metabolism
Protein PBDC1	Pbdc1				
Procollagen-proline, 2-	P4ha2	4-hydroxyproline metabolic process;amine met	binding;carboxylic acid binding;catalytic activity	cell part;collagen;cytoplasmic part;endoplasmi	Arginine and proline metabolism
60S ribosomal protein	Rpl39	biosynthetic process;cellular biosynthetic proce	structural constituent of ribosome;structural m	cell part;cytoplasmic part;intracellular non-mem	Ribosome
Teneurin-4	Tenm4	anatomical structure formation involved in mo	binding;identical protein binding;protein bindin	axon;cell part;cell projection;cytoplasm;cytopla	
40S ribosomal protein	Rps21	biosynthetic process;cellular biosynthetic proce	binding;ribonucleoprotein binding;ribosome bi	cell part;cytosolic small ribosomal subunit;intra	Ribosome
Protein prune homolog	Prune2	apoptosis;cell death;cellular process;death;pro	catalytic activity;hydrolase activity;hydrolase a	cell part;cytoplasmic part;Golgi apparatus;intra	
Triple functional doma	Trio	biological regulation;regulation of biological pr	adenyl nucleotide binding;adenyl ribonucleotid	cell part;cytoplasm;intracellular part	
Eukaryotic translation i	Eif4e	anatomical structure development;biological re	binding;nucleic acid binding;RNA binding;trans	cell part;chromatoid body;cytoplasmic mRNA p	Insulin signaling pathway;mTOR signaling pathw
40S ribosomal protein	Rps29	biosynthetic process;cellular biosynthetic proce	binding;cation binding;ion binding;metal ion bi	cell part;cytosolic small ribosomal subunit;intra	Ribosome

Dynactin subunit 4	Dctn4			cell part;centrosome;contractile fiber part;cyto	Huntington's disease;Vasopressin-regulated wa
Collagen alpha-1(VIII) c	Col8a1	anatomical structure formation involved in mor		basement membrane;collagen;extracellular ma	
FAT atypical cadherin 1	Fat1	actin cytoskeleton organization;actin filament c	binding;calcium ion binding;cation binding;ion	cell junction;cell part;cell projection;cell-cell jur	

Supplemental table 4: Immune system-related proteins in EMT/6

Selection value	-Log t-test P-value EMT/6 Control vs RT	t-test difference EMT/6 Control vs RT	Protein name	Gene name
Overexpressed in EMT/6 Control group	1.564964956	-2.082789103	Complement component 1, s subcomponent 1;Complement C1s-A	C1s1;C1sa
Overexpressed in EMT/6 Control group	2.274859318	-1.688479106	Complement factor H	Cfh
Overexpressed in EMT/6 Control group	2.774803021	-1.572367986	Fractalkine	Cx3cl1
Overexpressed in EMT/6 Control group	1.222555596	-1.520752589	Inhibitor of nuclear factor kappa-B kinase subunit beta (Fragment	Ikbkb
Overexpressed in EMT/6 Control group	2.313794333	-1.324079514	Vascular endothelial growth factor A	Vegfa
Overexpressed in EMT/6 Control group	2.7212314	-1.149957657	C-X-C motif chemokine 5	Cxcl5
Overexpressed in EMT/6 Control group	1.911601251	-0.891702016	C-X-C motif chemokine 3	Cxcl3
Overexpressed in EMT/6 Control group	1.64024887	-0.467979431	Osteopontin	Spp1
Overexpressed in EMT/6 Control group	3.24275804	-0.850516001	Disintegrin and metalloproteinase domain-containing protein 10	Adam10
Overexpressed in EMT/6 Control group	1.367017024	-0.82421875	Tyrosine-protein kinase Tec;Tyrosine-protein kinase	Tec
Overexpressed in EMT/6 Control group	1.826763218	-0.807607015	Prefoldin 1;Prefoldin subunit 1	Pfdn1
Overexpressed in EMT/6 Control group	1.504544329	-0.80054601	Complement C1r-A subcomponent;Complement C1r-B subcompo	C1ra;C1rb
Overexpressed in EMT/6 Control group	2.118481252	-0.73431778	C-type lectin domain family 2 member D	Clec2d
Overexpressed in EMT/6 Control group	1.540510327	-0.705455144	Epidermal growth factor receptor	Egfr
Overexpressed in EMT/6 Control group	1.686627859	-0.683345159	Integrin alpha-6	Itga6
Overexpressed in EMT/6 Control group	3.580388975	-0.677254995	Proteasome activator complex subunit 1 (Fragment);Proteasome	Psme1
Overexpressed in EMT/6 Control group	4.701501834	-0.592772166	Nck-associated protein 1	Nckap1
Overexpressed in EMT/6 Control group	1.66038175	-0.581769943	Dipeptidyl peptidase 1	Ctsc
Overexpressed in EMT/6 Control group	5.072144227	-0.566053391	Macrophage migration inhibitory factor	Mif
Overexpressed in EMT/6 Control group	2.18375626	-0.558676402	Ubiquitin-conjugating enzyme E2 N	Ube2n
Overexpressed in EMT/6 Control group	2.18221965	-0.542819341	Peptidyl-prolyl cis-trans isomerase FKBP1A	Fkbp1a
Overexpressed in EMT/6 Control group	2.091304007	-0.53572464	Pentraxin-related protein PTX3	Ptx3
Overexpressed in EMT/6 Control group	2.789816234	-0.53094101	Neurogenic locus notch homolog protein 2	Notch2
Overexpressed in EMT/6 Control group	2.693112541	-0.527263641	Protein NDRG1	Ndrg1
Overexpressed in EMT/6 Control group	2.813250172	-0.369141897	Galectin-1	Lgals1
Overexpressed in EMT/6 Control group	2.847286833	-0.360414505	Galectin;Galectin-3	Lgals3
Overexpressed in EMT/6 Control group	2.749924628	-0.313562393	Superoxide dismutase [Cu-Zn]	Sod1
Overexpressed in EMT/6 RT group	2.253748407	0.345085144	Dolichyl-diphosphooligosaccharide--protein glycosyltransferase 48	Ddost
Overexpressed in EMT/6 RT group	2.468897916	0.357931773	Caveolin-1;Caveolin (Fragment)	Cav1
Overexpressed in EMT/6 RT group	3.975965801	0.487415314	Annexin A3	Anxa3
Overexpressed in EMT/6 RT group	1.810869016	0.48900795	RAC-alpha serine/threonine-protein kinase	Akt1
Overexpressed in EMT/6 RT group	1.782900815	0.610490163	Complement component 1 Q subcomponent-binding protein, mit	C1qbp
Overexpressed in EMT/6 RT group	1.3920395	0.66785113	SPI6	Serpinb9
Overexpressed in EMT/6 RT group	4.355034047	0.694709142	60 kDa heat shock protein, mitochondrial	Hspd1
Overexpressed in EMT/6 RT group	1.672428934	0.774076462	Nuclear factor NF-kappa-B p100 subunit	Nfkb2
Overexpressed in EMT/6 RT group	3.798183545	0.947730382	Ferritin heavy chain	Fth1
Overexpressed in EMT/6 RT group	5.023851988	1.005846024	Tubulointerstitial nephritis antigen-like	Tinagl1

Supplemental table 5: Immune system-related proteins in PyMT

Selection value	-Log t-test P-value PyMT Control vs RT	t-test difference PyMT Control vs RT	Protein name	Gene name
Overexpressed in EMT/6 Control group	2.735926848	-3.123858134	Complement factor H	Cfh
Overexpressed in EMT/6 Control group	2.782136599	-1.646229426	Protein CTLA-2-alpha;Uncharacterized protein	Ctla2a
Overexpressed in EMT/6 Control group	2.840269096	-1.444316228	Gamma-interferon-inducible lysosomal thiol re	Ifi30
Overexpressed in EMT/6 Control group	2.344985299	-1.386327744	Macrophage colony-stimulating factor 1;Macro	Csf1
Overexpressed in EMT/6 Control group	1.556270295	-1.375341415	Antigen peptide transporter 1	Tap1
Overexpressed in EMT/6 Control group	4.505391367	-1.333791097	Osteopontin	Spp1
Overexpressed in EMT/6 Control group	2.588120991	-1.324750264	Beta-2-microglobulin	B2m
Overexpressed in EMT/6 Control group	2.438440466	-1.245669683	Vascular endothelial growth factor A	Vegfa
Overexpressed in EMT/6 Control group	3.093332461	-1.068702062	Fractalkine	Cx3cl1
Overexpressed in EMT/6 Control group	1.819320811	-1.014217377	Pentraxin-related protein PTX3	Ptx3
Overexpressed in EMT/6 Control group	2.278765773	-0.999883652	Angiotensin-converting enzyme;Angiotensin-co	Ace
Overexpressed in EMT/6 Control group	1.617174647	-0.7658933	Protein canopy homolog 3	Cnpy3
Overexpressed in EMT/6 Control group	2.13935466	-0.405347824	Tubulointerstitial nephritis antigen-like	Tinagl1
Overexpressed in EMT/6 RT group	2.738859445	0.321188609	Hypoxanthine-guanine phosphoribosyltransfera	Hprt1
Overexpressed in EMT/6 RT group	3.08619317	0.368747711	60S ribosomal protein L22	Rpl22
Overexpressed in EMT/6 RT group	3.212125635	0.409210841	Peroxiredoxin-1;Peroxiredoxin-1 (Fragment)	Prdx1
Overexpressed in EMT/6 RT group	2.890570138	0.440524419	Peroxiredoxin-2;Peroxiredoxin-2 (Fragment)	Prdx2
Overexpressed in EMT/6 RT group	3.077122953	0.624221166	Aminoacyl tRNA synthase complex-interacting	Aimp1

Supplemental table 6: Fisher exact test for the comparison between EMT/6 Control and EMT/6 RT. FDR threshold: 0.02

Selection value	Category column	Category value	Total size	Selection size	Category size	Intersection size	Enrichment factor	P value	Benj. Hoch. FDR
Enriched in EMT/6 RT group	GOBP name	acetyl-CoA catabolic process	2975	246	22	14	7.6959	8.22E-11	1.20E-07
Enriched in EMT/6 RT group	GOBP name	acetyl-CoA metabolic process	2975	246	27	14	6.2707	3.42E-09	2.13E-06
Enriched in EMT/6 Control group	GOMF name	adenyl nucleotide binding	2975	243	395	13	0.40293	1.60E-05	0.0023883
Enriched in EMT/6 Control group	GOMF name	adenyl ribonucleotide binding	2975	243	393	13	0.40498	1.80E-05	0.0024033
Enriched in EMT/6 Control group	GOBP name	alcohol catabolic process	2975	243	43	12	3.4166	8.36E-05	0.018551
Enriched in EMT/6 RT group	KEGG name	Alzheimer's disease	2975	246	80	26	3.9304	2.27E-10	1.92E-08
Enriched in EMT/6 RT group	KEGG name	Arginine and proline metabolism	2975	246	22	9	4.9473	2.65E-05	0.0010322
Enriched in EMT/6 Control group	GOMF name	ATP binding	2975	243	390	13	0.40809	2.16E-05	0.0026815
Enriched in EMT/6 RT group	GOBP name	ATP biosynthetic process	2975	246	19	13	8.2745	1.04E-10	1.24E-07
Enriched in EMT/6 RT group	GOBP name	ATP hydrolysis coupled proton transport	2975	246	13	10	9.3027	2.81E-09	2.16E-06
Enriched in EMT/6 RT group	GOBP name	ATP metabolic process	2975	246	39	16	4.9614	1.76E-08	9.21E-06
Enriched in EMT/6 RT group	GOBP name	ATP synthesis coupled proton transport	2975	246	13	11	10.233	6.63E-11	1.24E-07
Enriched in EMT/6 RT group	GOMF name	ATPase activity, coupled to movement of substances	2975	246	40	11	3.3257	0.0002089	0.018119
Enriched in EMT/6 RT group	GOMF name	ATPase activity, coupled to transmembrane movement of ions	2975	246	26	10	4.6513	1.77E-05	0.0024451
Enriched in EMT/6 RT group	GOMF name	ATPase activity, coupled to transmembrane movement of substances	2975	246	40	11	3.3257	0.0002089	0.017707
Enriched in EMT/6 RT group	KEGG name	Benzoate degradation	2975	246	7	5	8.6382	6.60E-05	0.002386
Enriched in EMT/6 RT group	GOMF name	binding	2975	246	1700	112	0.79675	3.62E-05	0.00397
Enriched in EMT/6 RT group	GOBP name	biological regulation	2975	246	1348	79	0.70874	3.54E-06	0.0013232
Enriched in EMT/6 RT group	GOBP name	biosynthetic process	2975	246	496	76	1.853	3.27E-09	2.14E-06
Enriched in EMT/6 RT group	KEGG name	Butanoate metabolism	2975	246	11	7	7.6959	5.76E-06	0.00029144
Enriched in EMT/6 Control group	GOMF name	carbohydrate binding	2975	243	86	22	3.1319	5.23E-07	9.29E-05
Enriched in EMT/6 Control group	GOBP name	carbohydrate catabolic process	2975	243	44	13	3.6172	2.23E-05	0.0059565
Enriched in EMT/6 RT group	KEGG name	Cardiac muscle contraction	2975	246	29	12	5.0042	1.02E-06	6.42E-05
Enriched in EMT/6 Control group	GOMF name	catalytic activity	2975	243	1230	76	0.75647	0.0001927	0.017115
Enriched in EMT/6 RT group	GOMF name	cation transmembrane transporter activity	2975	246	81	30	4.4791	1.71E-13	1.28E-10
Enriched in EMT/6 RT group	GOBP name	cation transport	2975	246	88	27	3.7105	4.35E-10	4.06E-07
Enriched in EMT/6 RT group	GOMF name	cation-transporting ATPase activity	2975	246	16	7	5.2909	0.0001311	0.012539
Enriched in EMT/6 RT group	GOCC name	cell part	2975	246	2689	238	1.0704	4.73E-05	0.0020885
Enriched in EMT/6 Control group	GOCC name	cell part	2975	243	2689	203	0.92424	0.0001635	0.0067148
Enriched in EMT/6 RT group	GOBP name	cellular biosynthetic process	2975	246	469	74	1.9081	1.47E-09	1.28E-06
Enriched in EMT/6 Control group	GOBP name	cellular carbohydrate catabolic process	2975	243	40	13	3.9789	7.17E-06	0.0024679
Enriched in EMT/6 Control group	GOBP name	cellular component assembly at cellular level	2975	243	231	36	1.908	4.03E-05	0.009955
Enriched in EMT/6 RT group	GOBP name	cellular ketone metabolic process	2975	246	207	33	1.9279	6.83E-05	0.015414
Enriched in EMT/6 RT group	GOBP name	cellular macromolecule biosynthetic process	2975	246	237	39	1.9901	7.21E-06	0.0024186
Enriched in EMT/6 RT group	GOBP name	cellular metabolic process	2975	246	1279	143	1.3521	2.25E-07	9.48E-05
Enriched in EMT/6 Control group	GOBP name	chromatin assembly	2975	243	6	5	10.202	1.93E-05	0.0054954
Enriched in EMT/6 Control group	GOBP name	chromatin organization	2975	243	56	17	3.7166	8.19E-07	0.00032464
Enriched in EMT/6 Control group	GOCC name	chromosomal part	2975	243	98	18	2.2487	0.0004841	0.016764
Enriched in EMT/6 RT group	GOCC name	chromosomal part	2975	246	98	0	0	0.0001831	0.0073477
Enriched in EMT/6 Control group	GOBP name	chromosome organization	2975	243	75	18	2.9383	1.45E-05	0.0042238
Enriched in EMT/6 RT group	KEGG name	Citrate cycle (TCA cycle)	2975	246	26	16	7.4422	7.14E-12	9.03E-10
Enriched in EMT/6 RT group	GOBP name	coenzyme catabolic process	2975	246	24	14	7.0545	4.28E-10	4.31E-07
Enriched in EMT/6 RT group	GOBP name	coenzyme metabolic process	2975	246	72	18	3.0234	9.49E-06	0.0030303
Enriched in EMT/6 RT group	GOBP name	cofactor catabolic process	2975	246	26	14	6.5119	1.79E-09	1.46E-06
Enriched in EMT/6 RT group	GOBP name	cofactor metabolic process	2975	246	89	19	2.5818	5.52E-05	0.013132
Enriched in EMT/6 RT group	KEGG name	Collecting duct acid secretion	2975	246	12	8	8.0623	6.97E-07	5.04E-05
Enriched in EMT/6 RT group	GOMF name	cytochrome-c oxidase activity	2975	246	10	8	9.6748	7.49E-08	2.33E-05
Enriched in EMT/6 RT group	GOCC name	cytoplasm	2975	246	679	27	0.48089	3.49E-07	2.05E-05
Enriched in EMT/6 RT group	GOCC name	cytoplasmic part	2975	246	1672	186	1.3453	2.22E-11	3.27E-09
Enriched in EMT/6 Control group	GOCC name	cytoplasmic part	2975	243	1672	114	0.83474	0.0005405	0.018356
Enriched in EMT/6 RT group	GOCC name	cytosolic large ribosomal subunit	2975	246	26	13	6.0467	2.25E-08	1.65E-06
Enriched in EMT/6 RT group	GOCC name	cytosolic small ribosomal subunit	2975	246	24	8	4.0312	0.0003782	0.013359
Enriched in EMT/6 RT group	GOBP name	dicarboxylic acid metabolic process	2975	246	19	8	5.092	5.92E-05	0.013823
Enriched in EMT/6 Control group	GOMF name	DNA binding	2975	243	164	32	2.3888	1.03E-06	0.00016771
Enriched in EMT/6 RT group	GOBP name	energy coupled proton transport, against electrochemical gradient	2975	246	13	10	9.3027	2.81E-09	2.04E-06
Enriched in EMT/6 RT group	GOBP name	energy coupled proton transport, down electrochemical gradient	2975	246	13	11	10.233	6.63E-11	1.09E-07
Enriched in EMT/6 RT group	KEGG name	Epithelial cell signaling in Helicobacter pylori infection	2975	246	28	9	3.8872	0.0002228	0.0070459
Enriched in EMT/6 Control group	GOCC name	ESCRT I complex	2975	243	4	4	12.243	4.35E-05	0.0019702
Enriched in EMT/6 Control group	GOCC name	extracellular matrix	2975	243	61	20	4.014	2.04E-08	1.56E-06
Enriched in EMT/6 Control group	GOCC name	extracellular matrix part	2975	243	55	13	2.8938	0.0002533	0.0093208
Enriched in EMT/6 Control group	GOCC name	extracellular region	2975	243	121	21	2.1248	0.000376	0.013551
Enriched in EMT/6 Control group	GOCC name	extracellular region part	2975	243	235	57	2.9695	1.54E-15	3.89E-13
Enriched in EMT/6 Control group	GOCC name	extracellular space	2975	243	127	32	3.0848	1.80E-09	1.77E-07
Enriched in EMT/6 RT group	GOMF name	heme-copper terminal oxidase activity	2975	246	10	8	9.6748	7.49E-08	2.15E-05
Enriched in EMT/6 RT group	GOBP name	heterocycle biosynthetic process	2975	246	79	18	2.7555	3.56E-05	0.0089494
Enriched in EMT/6 Control group	GOBP name	hexose catabolic process	2975	243	30	11	4.489	1.01E-05	0.0031469
Enriched in EMT/6 RT group	KEGG name	Huntington's disease	2975	246	82	28	4.1295	1.15E-11	1.16E-09
Enriched in EMT/6 RT group	GOMF name	hydrogen ion transmembrane transporter activity	2975	246	38	28	8.911	2.46E-23	9.17E-20
Enriched in EMT/6 RT group	GOMF name	hydrogen ion transporting ATP synthase activity, rotational mechanism	2975	246	8	6	9.0701	7.15E-06	0.0011108
Enriched in EMT/6 RT group	GOBP name	hydrogen transport	2975	246	27	22	9.854	3.41E-20	4.47E-16
Enriched in EMT/6 RT group	GOMF name	hydrogen-exporting ATPase activity, phosphorylative mechanism	2975	246	6	5	10.078	2.05E-05	0.0026403
Enriched in EMT/6 Control group	GOMF name	hydrolase activity, acting on acid anhydrides	2975	243	263	6	0.2793	2.27E-05	0.0027294
Enriched in EMT/6 RT group	GOMF name	hydrolase activity, acting on acid anhydrides, catalyzing transmembrane movement of substance	2975	246	41	12	3.5396	5.71E-05	0.0059204
Enriched in EMT/6 Control group	GOMF name	hydrolase activity, acting on acid anhydrides, in phosphorus-containing anhydrides	2975	243	260	6	0.28253	2.78E-05	0.0032445
Enriched in EMT/6 RT group	GOMF name	inorganic cation transmembrane transporter activity	2975	246	57	30	6.365	1.09E-18	1.35E-15
Enriched in EMT/6 RT group	GOCC name	intracellular organelle lumen	2975	246	105	29	3.3401	1.49E-09	1.54E-07
Enriched in EMT/6 RT group	GOCC name	intracellular organelle part	2975	246	1383	176	1.539	6.29E-17	2.22E-14
Enriched in EMT/6 Control group	GOCC name	intracellular organelle part	2975	243	1383	85	0.75245	4.28E-05	0.0019868
Enriched in EMT/6 RT group	GOCC name	intracellular part	2975	246	2443	226	1.1188	3.18E-06	0.00017522
Enriched in EMT/6 RT group	GOBP name	ion transmembrane transport	2975	246	35	20	6.9106	1.06E-13	2.31E-10
Enriched in EMT/6 RT group	GOMF name	ion transmembrane transporter activity	2975	246	98	31	3.8255	8.25E-12	5.13E-09
Enriched in EMT/6 RT group	GOBP name	ion transport	2975	246	100	29	3.5071	4.21E-10	4.59E-07
Enriched in EMT/6 RT group	GOCC name	large ribosomal subunit	2975	246	31	14	5.4616	3.26E-08	2.22E-06
Enriched in EMT/6 RT group	GOCC name	macromolecular complex	2975	246	994	112	1.3626	1.12E-05	0.00054819
Enriched in EMT/6 RT group	GOBP name	macromolecule biosynthetic process	2975	246	243	39	1.9409	1.30E-05	0.0038654
Enriched in EMT/6 RT group	GOCC name	membrane part	2975	246	916	100	1.3203	0.0001568	0.0065918
Enriched in EMT/6 RT group	GOCC name	membrane-enclosed lumen	2975	246	125	31	2.9992	6.72E-09	5.65E-07
Enriched in EMT/6 RT group	GOBP name	metabolic process	2975	246	1428	154	1.3042	5.43E-07	0.00022203
Enriched in EMT/6 RT group	GOCC name	mitochondrial inner membrane	2975	246	109	48	5.3256	3.58E-25	3.16E-22
Enriched in EMT/6 RT group	GOCC name	mitochondrial matrix	2975	246	48	21	5.2909	2.14E-11	3.44E-09
Enriched in EMT/6 RT group	GOCC name	mitochondrial membrane	2975	246	156	53	4.1087	2.16E-21	9.51E-19
Enriched in EMT/6 RT group	GOCC name	mitochondrial membrane part	2975	246	55	24	5.2772	7.82E-13	1.53E-10
Enriched in EMT/6 RT group	GOCC name	mitochondrial nucleoid	2975	246	18	10	6.7186	2.84E-07	1.73E-05
Enriched in EMT/6 RT group	GOCC name	mitochondrial part	2975	246	239	83	4.1998	3.51E-35	6.19E-32
Enriched in EMT/6 RT group	GOCC name	mitochondrial proton-transporting ATP synthase complex	2975	246	15	12	9.6748	2.83E-11	3.84E-09
Enriched in EMT/6 RT group	GOCC name	mitochondrial proton-transporting ATP synthase complex, coupling factor F(o)	2975	246	7	7	12.093	2.44E-08	1.73E-06
Enriched in EMT/6 RT group	GOCC name	mitochondrial respiratory chain complex III	2975	246	6	4	8.0623	0.0005785	0.018919
Enriched in EMT/6 RT group	GOCC name	mitochondrion	2975	246	330	54	1.9789	1.22E-07	7.72E-06
Enriched in EMT/6 Control group	GOMF name	monosaccharide binding	2975	243	20	8	4.8971	8.32E-05	0.0083826
Enriched in EMT/6 Control group	GOBP name	monosaccharide catabolic process	2975	243	32	11	4.2085	2.02E-05	0.0055191
Enriched in EMT/6 RT group	GOMF name	monovalent inorganic cation transmembrane transporter activity	2975	246	51	29	6.8767	2.39E-19	4.46E-16
Enriched in EMT/6 RT group	GOBP name	monovalent inorganic cation transport	2975	246	39	23	7.1321	5.09E-16	1.66E-12
Enriched in EMT/6 RT group	GOCC name	nucleoid	2975	246	19	10	6.365	5.51E-07	3.14E-05
Enriched in EMT/6 RT group	GOBP name	nucleoside triphosphate biosynthetic process	2975	246	28	13	5.6148	6.86E-08	3.33E-05
Enriched in EMT/6 RT group	GOBP name	nucleoside triphosphate metabolic process	2975	246	71	17	2.8956	3.01E-05	0.0077233
Enriched in EMT/6 Control group	GOMF name	nucleoside-triphosphatase activity	2975	243	248	5	0.24683	1.61E-05	0.0023119
Enriched in EMT/6 Control group	GOCC name	nucleosome	2975	243	17	12	8.642	2.82E-10	3.11E-08
Enriched in EMT/6 Control group	GOBP name	nucleosome assembly	2975	243	21	12	6.9959	9.68E-09	5.51E-06

Enriched in EMT/6 Control group	GOBP name	nucleosome organization	2975	243	24	12	6.1214	6.97E-08	3.26E-05
Enriched in EMT/6 Control group	GOMF name	nucleotide binding	2975	243	656	33	0.61587	0.0001699	0.015456
Enriched in EMT/6 RT group	GOCC name	nucleus	2975	246	625	32	0.61919	0.0002525	0.0094869
Enriched in EMT/6 RT group	GOCC name	organelle inner membrane	2975	246	120	49	4.9382	6.84E-24	4.03E-21
Enriched in EMT/6 RT group	GOCC name	organelle lumen	2975	246	106	29	3.3086	1.89E-09	1.76E-07
Enriched in EMT/6 RT group	GOCC name	organelle membrane	2975	246	585	81	1.6745	1.03E-07	6.74E-06
Enriched in EMT/6 RT group	GOCC name	organelle part	2975	246	1407	176	1.5128	5.39E-16	1.59E-13
Enriched in EMT/6 Control group	GOCC name	organelle part	2975	243	1407	86	0.74832	2.65E-05	0.0012671
Enriched in EMT/6 RT group	KEGG name	Oxidative phosphorylation	2975	246	70	37	6.3923	5.52E-23	2.79E-20
Enriched in EMT/6 RT group	GOMF name	oxidoreductase activity	2975	246	230	41	2.1558	5.10E-07	9.51E-05
Enriched in EMT/6 RT group	GOMF name	oxidoreductase activity, acting on a heme group of donors	2975	246	11	8	8.7953	2.52E-07	4.96E-05
Enriched in EMT/6 RT group	GOMF name	oxidoreductase activity, acting on a heme group of donors, oxygen as acceptor	2975	246	10	8	9.6748	7.49E-08	1.99E-05
Enriched in EMT/6 RT group	KEGG name	Parkinson's disease	2975	246	65	28	5.2095	1.26E-14	2.13E-12
Enriched in EMT/6 Control group	KEGG name	Pentose phosphate pathway	2975	243	20	7	4.285	0.0005898	0.016579
Enriched in EMT/6 RT group	KEGG name	Phagosome	2975	246	72	15	2.5195	0.0004161	0.012385
Enriched in EMT/6 RT group	GOMF name	protein binding	2975	246	376	13	0.41813	3.75E-05	0.0039975
Enriched in EMT/6 Control group	GOCC name	proteinaceous extracellular matrix	2975	243	35	11	3.8477	5.12E-05	0.0022039
Enriched in EMT/6 Control group	GOCC name	protein-DNA complex	2975	243	20	13	7.9578	2.35E-10	2.76E-08
Enriched in EMT/6 Control group	GOBP name	protein-DNA complex assembly	2975	243	21	12	6.9959	9.68E-09	5.28E-06
Enriched in EMT/6 Control group	GOBP name	protein-DNA complex subunit organization	2975	243	24	12	6.1214	6.97E-08	3.15E-05
Enriched in EMT/6 RT group	GOBP name	proton transport	2975	246	27	22	9.854	3.41E-20	2.23E-16
Enriched in EMT/6 RT group	GOCC name	proton-transporting ATP synthase complex	2975	246	15	12	9.6748	2.83E-11	3.57E-09
Enriched in EMT/6 RT group	GOCC name	proton-transporting ATP synthase complex, catalytic core F(1)	2975	246	5	4	9.6748	0.0002099	0.008239
Enriched in EMT/6 RT group	GOCC name	proton-transporting ATP synthase complex, coupling factor F(o)	2975	246	8	8	12.093	1.97E-09	1.74E-07
Enriched in EMT/6 RT group	GOMF name	proton-transporting ATPase activity, rotational mechanism	2975	246	9	7	9.4061	7.43E-07	0.00012602
Enriched in EMT/6 RT group	GOCC name	proton-transporting two-sector ATPase complex	2975	246	19	15	9.5475	1.08E-13	2.39E-11
Enriched in EMT/6 RT group	GOCC name	proton-transporting two-sector ATPase complex, catalytic domain	2975	246	11	9	9.8947	7.35E-09	5.90E-07
Enriched in EMT/6 RT group	GOCC name	proton-transporting two-sector ATPase complex, proton-transporting domain	2975	246	12	11	11.086	1.11E-11	1.96E-09
Enriched in EMT/6 RT group	GOCC name	proton-transporting V-type ATPase, V1 domain	2975	246	5	4	9.6748	0.0002099	0.0080599
Enriched in EMT/6 RT group	GOBP name	purine nucleoside triphosphate biosynthetic process	2975	246	24	13	6.5506	6.36E-09	3.78E-06
Enriched in EMT/6 RT group	GOBP name	purine nucleoside triphosphate metabolic process	2975	246	65	17	3.1629	8.88E-06	0.0029051
Enriched in EMT/6 Control group	GOMF name	purine nucleotide binding	2975	243	531	16	0.3689	7.62E-08	1.90E-05
Enriched in EMT/6 RT group	GOBP name	purine nucleotide biosynthetic process	2975	246	43	13	3.6562	1.94E-05	0.005409
Enriched in EMT/6 Control group	GOMF name	purine ribonucleoside triphosphate binding	2975	243	525	16	0.37311	1.14E-07	2.35E-05
Enriched in EMT/6 RT group	GOBP name	purine ribonucleoside triphosphate biosynthetic process	2975	246	23	13	6.8355	3.16E-09	2.18E-06
Enriched in EMT/6 RT group	GOBP name	purine ribonucleoside triphosphate metabolic process	2975	246	63	17	3.2633	5.67E-06	0.0020621
Enriched in EMT/6 Control group	GOMF name	purine ribonucleotide binding	2975	243	529	16	0.37029	8.71E-08	2.03E-05
Enriched in EMT/6 RT group	GOBP name	purine ribonucleotide biosynthetic process	2975	246	39	13	4.0312	6.01E-06	0.0021243
Enriched in EMT/6 Control group	GOMF name	pyrophosphatase activity	2975	243	260	6	0.28253	2.78E-05	0.0031462
Enriched in EMT/6 RT group	GOCC name	pyruvate dehydrogenase complex	2975	246	3	3	12.093	0.0005591	0.018629
Enriched in EMT/6 RT group	KEGG name	Pyruvate metabolism	2975	246	26	8	3.7211	0.000679	0.018083
Enriched in EMT/6 RT group	GOBP name	regulation of biological process	2975	246	1279	69	0.65242	1.72E-07	7.49E-05
Enriched in EMT/6 Control group	GOBP name	regulation of cell aging	2975	243	4	4	12.243	4.35E-05	0.010544
Enriched in EMT/6 RT group	GOBP name	regulation of cellular process	2975	246	1169	65	0.67244	3.39E-06	0.0013062
Enriched in EMT/6 RT group	GOCC name	respiratory chain complex III	2975	246	6	4	8.0623	0.0005785	0.018575
Enriched in EMT/6 RT group	KEGG name	Rheumatoid arthritis	2975	246	18	9	6.0467	3.62E-06	0.00020359
Enriched in EMT/6 RT group	GOCC name	ribonucleoprotein complex	2975	246	243	40	1.9907	5.43E-06	0.00029068
Enriched in EMT/6 RT group	GOBP name	ribonucleoside triphosphate biosynthetic process	2975	246	26	13	6.0467	2.25E-08	1.13E-05
Enriched in EMT/6 RT group	GOBP name	ribonucleoside triphosphate metabolic process	2975	246	66	17	3.115	1.10E-05	0.0033535
Enriched in EMT/6 Control group	GOMF name	ribonucleotide binding	2975	243	529	16	0.37029	8.71E-08	1.91E-05
Enriched in EMT/6 RT group	GOBP name	ribonucleotide biosynthetic process	2975	246	48	13	3.2753	6.77E-05	0.015545
Enriched in EMT/6 RT group	KEGG name	Ribosome	2975	246	79	33	5.0517	1.37E-16	3.46E-14
Enriched in EMT/6 RT group	GOCC name	ribosome	2975	246	39	13	4.0312	6.01E-06	0.00031197
Enriched in EMT/6 RT group	GOBP name	small molecule metabolic process	2975	246	472	62	1.5886	2.60E-05	0.0068058
Enriched in EMT/6 RT group	GOCC name	small ribosomal subunit	2975	246	34	12	4.2683	7.09E-06	0.00035784
Enriched in EMT/6 RT group	GOMF name	structural constituent of ribosome	2975	246	83	31	4.5168	4.99E-14	4.65E-11
Enriched in EMT/6 RT group	GOMF name	structural molecule activity	2975	246	159	37	2.8142	1.40E-09	5.78E-07
Enriched in EMT/6 RT group	GOMF name	substrate-specific transmembrane transporter activity	2975	246	125	34	3.2894	8.05E-11	3.75E-08
Enriched in EMT/6 RT group	GOMF name	substrate-specific transporter activity	2975	246	167	35	2.5346	6.88E-08	2.33E-05
Enriched in EMT/6 Control group	GOMF name	sugar binding	2975	243	20	8	4.8971	8.32E-05	0.008162
Enriched in EMT/6 Control group	KEGG name	Systemic lupus erythematosus	2975	243	20	8	4.8971	8.32E-05	0.002805
Enriched in EMT/6 RT group	GOMF name	transferase activity, transferring phosphorus-containing groups	2975	246	173	3	0.20971	0.0001526	0.014228
Enriched in EMT/6 RT group	GOBP name	translation	2975	246	81	31	4.6284	2.27E-14	5.94E-11
Enriched in EMT/6 RT group	GOBP name	transmembrane transport	2975	246	54	27	6.0467	3.63E-16	1.58E-12
Enriched in EMT/6 RT group	GOMF name	transmembrane transporter activity	2975	246	141	37	3.1735	3.44E-11	1.83E-08
Enriched in EMT/6 RT group	GOMF name	transporter activity	2975	246	198	40	2.4431	2.16E-08	8.07E-06
Enriched in EMT/6 RT group	GOBP name	tricarboxylic acid cycle	2975	246	22	14	7.6959	8.22E-11	1.08E-07
Enriched in EMT/6 RT group	KEGG name	Valine, leucine and isoleucine degradation	2975	246	27	10	4.4791	2.59E-05	0.0010904
Enriched in EMT/6 RT group	KEGG name	Vibrio cholerae infection	2975	246	29	11	4.5872	7.81E-06	0.0003594

Supplemental table 7: Fisher exact test for the comparison between PyMT Control and PyMT RT. FDR threshold: 0.02

Selection value	Category column	Category value	Total size	Selection size	Category size	Intersection size	Enrichment factor	P value	Benj. Hoch. FDR
Enriched in PyMT RT group	KEGG name	Aminoacyl-tRNA biosynthesis	2975	110	21	6	7.7273	7.10E-05	0.0044887
Enriched in PyMT RT group	GOBP name	biosynthetic process	2975	110	496	38	2.072	1.70E-06	0.0014827
Enriched in PyMT Control group	GOCC name	catalytic step 2 spliceosome	2975	205	33	16	7.0362	5.59E-11	1.97E-08
Enriched in PyMT RT group	GOBP name	cellular biosynthetic process	2975	110	469	38	2.1913	4.10E-07	0.00038329
Enriched in PyMT RT group	GOBP name	cellular macromolecule biosynthetic process	2975	110	237	23	2.6247	7.32E-06	0.005039
Enriched in PyMT RT group	GOCC name	centrosome	2975	110	73	11	4.0753	4.54E-05	0.0040123
Enriched in PyMT Control group	GOBP name	chromatin assembly	2975	205	6	6	14.512	9.99E-08	0.00010898
Enriched in PyMT Control group	GOBP name	chromatin assembly or disassembly	2975	205	8	6	10.884	2.44E-06	0.0019921
Enriched in PyMT Control group	GOBP name	chromatin organization	2975	205	56	19	4.9238	1.43E-09	2.08E-06
Enriched in PyMT Control group	GOCC name	chromosomal part	2975	205	98	21	3.1098	1.25E-06	0.0001703
Enriched in PyMT Control group	GOBP name	chromosome organization	2975	205	75	19	3.6764	2.75E-07	0.0002773
Enriched in PyMT RT group	GOCC name	cytosolic small ribosomal subunit	2975	110	24	6	6.7614	0.000158	0.010339
Enriched in PyMT Control group	GOMF name	DNA binding	2975	205	164	40	3.5396	1.31E-13	4.88E-10
Enriched in PyMT Control group	GOCC name	extracellular matrix	2975	205	61	13	3.0928	0.000141	0.0099514
Enriched in PyMT Control group	GOCC name	extracellular region	2975	205	121	27	3.2383	1.43E-08	2.82E-06
Enriched in PyMT Control group	GOCC name	extracellular region part	2975	205	235	39	2.4084	5.03E-08	8.08E-06
Enriched in PyMT Control group	GOCC name	extracellular space	2975	205	127	28	3.1995	1.01E-08	2.22E-06
Enriched in PyMT Control group	KEGG name	Glycosaminoglycan degradation	2975	205	8	7	12.698	5.00E-08	8.44E-06
Enriched in PyMT Control group	GOMF name	hydrolase activity, acting on glycosyl bonds	2975	205	22	9	5.9368	6.06E-06	0.0037653
Enriched in PyMT Control group	GOMF name	hydrolase activity, hydrolyzing O-glycosyl compounds	2975	205	17	9	7.6829	4.17E-07	0.00051865
Enriched in PyMT RT group	GOCC name	integral to membrane	2975	110	568	7	0.33331	7.94E-05	0.0058458
Enriched in PyMT RT group	GOCC name	intrinsic to membrane	2975	110	586	7	0.32307	4.49E-05	0.0041737
Enriched in PyMT Control group	KEGG name	Lysosome	2975	205	57	20	5.092	2.60E-10	1.31E-07
Enriched in PyMT Control group	GOCC name	lysosome	2975	205	83	28	4.8957	1.59E-13	2.81E-10
Enriched in PyMT Control group	GOBP name	lysosome organization	2975	205	9	6	9.6748	6.82E-06	0.0049558
Enriched in PyMT Control group	GOCC name	lytic vacuole	2975	205	83	28	4.8957	1.59E-13	1.41E-10
Enriched in PyMT RT group	GOBP name	macromolecule biosynthetic process	2975	110	243	23	2.5599	1.10E-05	0.0072078
Enriched in PyMT Control group	GOCC name	melanosome	2975	205	71	15	3.066	4.94E-05	0.0041531
Enriched in PyMT RT group	GOCC name	membrane part	2975	110	916	11	0.32478	9.98E-08	1.47E-05
Enriched in PyMT RT group	KEGG name	Methane metabolism	2975	110	16	6	10.142	1.25E-05	0.00090547
Enriched in PyMT RT group	GOCC name	microtubule associated complex	2975	110	33	9	7.376	1.56E-06	0.00019724
Enriched in PyMT Control group	GOCC name	mitochondrial part	2975	205	239	3	0.18216	2.15E-05	0.002114
Enriched in PyMT Control group	GOBP name	mRNA metabolic process	2975	205	100	31	4.4988	9.78E-14	1.28E-09
Enriched in PyMT Control group	GOBP name	mRNA processing	2975	205	85	27	4.6098	2.31E-12	1.51E-08
Enriched in PyMT Control group	GOCC name	nuclear body	2975	205	55	14	3.694	9.89E-06	0.0010912
Enriched in PyMT Control group	GOCC name	nuclear part	2975	205	430	58	1.9575	4.43E-08	7.82E-06
Enriched in PyMT Control group	GOMF name	nucleic acid binding	2975	205	408	67	2.3831	2.43E-13	4.54E-10
Enriched in PyMT Control group	GOBP name	nucleic acid metabolic process	2975	205	351	52	2.15	1.13E-08	1.34E-05
Enriched in PyMT RT group	GOBP name	nucleobase-containing small molecule metabolic process	2975	110	147	17	3.1277	1.38E-05	0.0085845
Enriched in PyMT Control group	GOBP name	nucleobase-containing small molecule metabolic process	2975	205	147	0	0	2.10E-05	0.012482
Enriched in PyMT Control group	GOCC name	nucleoplasm part	2975	205	106	19	2.6012	5.44E-05	0.0041767
Enriched in PyMT Control group	GOCC name	nucleosome	2975	205	17	11	9.3902	1.06E-09	3.13E-07
Enriched in PyMT Control group	GOBP name	nucleosome assembly	2975	205	21	13	8.9837	6.54E-11	1.71E-07
Enriched in PyMT Control group	GOBP name	nucleosome organization	2975	205	24	13	7.8608	6.56E-10	1.23E-06
Enriched in PyMT Control group	GOCC name	organelle	2975	205	1600	134	1.2154	0.000141	0.0095764
Enriched in PyMT Control group	KEGG name	Other glycan degradation	2975	205	9	6	9.6748	6.82E-06	0.00057489
Enriched in PyMT Control group	GOCC name	pigment granule	2975	205	71	15	3.066	4.94E-05	0.0039643
Enriched in PyMT Control group	GOCC name	protein-DNA complex	2975	205	20	13	9.4329	2.66E-11	1.18E-08
Enriched in PyMT Control group	GOBP name	protein-DNA complex assembly	2975	205	21	13	8.9837	6.54E-11	1.43E-07
Enriched in PyMT Control group	GOBP name	protein-DNA complex subunit organization	2975	205	24	13	7.8608	6.56E-10	1.07E-06
Enriched in PyMT RT group	GOCC name	ribonucleoprotein complex	2975	110	243	20	2.226	0.000281	0.017695
Enriched in PyMT RT group	KEGG name	Ribosome	2975	110	79	14	4.7929	5.54E-07	7.00E-05
Enriched in PyMT Control group	GOMF name	RNA binding	2975	205	266	38	2.0732	3.53E-06	0.002637
Enriched in PyMT Control group	GOBP name	RNA metabolic process	2975	205	273	45	2.3921	4.85E-09	6.35E-06
Enriched in PyMT Control group	GOBP name	RNA processing	2975	205	117	30	3.7211	5.54E-11	1.81E-07
Enriched in PyMT Control group	GOBP name	RNA splicing	2975	205	75	25	4.8374	4.79E-12	2.09E-08
Enriched in PyMT RT group	GOCC name	small ribosomal subunit	2975	110	34	9	7.1591	2.05E-06	0.00024177
Enriched in PyMT Control group	GOCC name	spliceosomal complex	2975	205	61	19	4.5202	7.20E-09	1.82E-06
Enriched in PyMT Control group	KEGG name	Spliceosome	2975	205	54	18	4.8374	5.45E-09	1.38E-06
Enriched in PyMT RT group	GOMF name	structural constituent of ribosome	2975	110	83	14	4.5619	1.03E-06	0.00095875
Enriched in PyMT Control group	KEGG name	Systemic lupus erythematosus	2975	205	20	9	6.5305	2.35E-06	0.00023743
Enriched in PyMT RT group	GOBP name	translation	2975	110	81	13	4.3406	4.50E-06	0.0034664
Enriched in PyMT Control group	GOCC name	U12-type spliceosomal complex	2975	205	14	7	7.2561	1.42E-05	0.0014721
Enriched in PyMT Control group	GOCC name	vacuole	2975	205	89	28	4.5656	1.14E-12	6.72E-10

Supplemental table 8: T cell suppressing effect of TMPs extracted from the irradiated cells, as presented by a ratio between PD-L1-positive TMPs and T cell activation

	EMT/6	*4T1*
Control	**0.073** *(2.39% PD-L1$^+$ TMPs / 3.27% CD8$^+$CD25$^+$)*	**0.157** *(4.91% PD-L1$^+$ TMPs / 3.12% CD8$^+$CD25$^+$)*
2 Gy	**0.479** *(9.22% PD-L1$^+$ TMPs / 1.93% CD8$^+$CD25$^+$)*	**0.176** *(5.61% PD-L1$^+$ TMPs / 3.17% CD8$^+$CD25$^+$)*
Fold change	**6.56**	**1.12**
